# Checklist of newly-vouchered annelid taxa from the Clarion-Clipperton Zone, central Pacific Ocean, based on morphology and genetic delimitation

**DOI:** 10.3897/BDJ.11.e86921

**Published:** 2023-09-15

**Authors:** Helena Wiklund, Muriel Rabone, Adrian G Glover, Guadalupe Bribiesca-Contreras, Regan Drennan, Eva C D Stewart, Corie M Boolukos, Lucas D King, Emma Sherlock, Craig R Smith, Thomas G Dahlgren, Lenka Neal

**Affiliations:** 1 Gothenburg Global Biodiversity Centre, Gothenburg, Sweden Gothenburg Global Biodiversity Centre Gothenburg Sweden; 2 Natural History Museum, London, United Kingdom Natural History Museum London United Kingdom; 3 University of Gothenburg, Gothenburg, Sweden University of Gothenburg Gothenburg Sweden; 4 University of Southampton, Southampton, United Kingdom University of Southampton Southampton United Kingdom; 5 University of Hawaii, Honolulu, United States of America University of Hawaii Honolulu United States of America; 6 NORCE Norwegian Research Centre, Bergen, Norway NORCE Norwegian Research Centre Bergen Norway

**Keywords:** taxonomy, DNA barcoding, biodiversity, conservation, deep-sea mining, abyssal Pacific, environmental impact

## Abstract

**Background:**

We present a checklist of annelids from recent United Kingdom Seabed Resources (UKSR) expeditions (Abyssal Baseline - ABYSSLINE project) to the eastern abyssal Pacific Clarion-Clipperton Zone (CCZ) polymetallic nodule fields, based on DNA species delimitation, including imagery of voucher specimens, Darwin Core (DwC) data and links to vouchered specimen material and new GenBank sequence records. This paper includes genetic and imagery data for 129 species of annelids from 339 records and is restricted to material that is, in general, in too poor a condition to describe formally at this time, but likely contains many species new to science. We make these data available both to aid future taxonomic studies in the CCZ that will be able to link back to these genetic data and specimens and to better underpin ongoing ecological studies of potential deep-sea mining impacts using the principles of FAIR (Findable, Accessible, Interoperable, Reusuable) data and specimens that will be available for all.

**New information:**

We include genetic, imagery and all associated metadata in Darwin Core format for 129 species of annelids from the Clarion-Clipperton Zone, eastern abyssal Pacific, with 339 records.

## Introduction

We present a checklist of annelid fauna from recent UKSR expeditions (ABYSSLINE project) to the CCZ the equatorial abyssal Pacific, based on DNA species delimitation, including imagery of voucher specimens, Darwin Core data (DwC) and links to vouchered specimen material and new GenBank sequence records. This is a region undergoing significant exploration for mineral resources in the form of polymetallic nodules at the seafloor (Hein et al. 2013) and, as such, future environmental monitoring of these areas will require access to information on the biodiversity of animals at the seafloor, in particular based on sound taxonomic sources (Glover et al. 2018, Rabone et al. 2023a, Rabone et al. 2023b).

Surveying of this region over the the past 20 years resulted in a large collection of often very small, morphologically sub-optimally preserved and difficult to identify annelids, despite the best efforts at collecting animals directly from samples at sea using cold-chain protocols (Glover et al. 2015). Typically, the specimens can be identifed to family or genus at best. Although good quality DNA sequences were obtained in most cases, there are few genetic matches to sequences available on public databases (Suppl. material 1), most being from other studies in the CCZ (Janssen et al. 2015, Janssen et al. 2019, Bonifácio and Menot 2019, Bonifacio et al. 2020, Bonifácio et al. 2021) or abyssal Atlantic (Boggemann 2009, Boggemann 2016). Based on detailed taxonomic studies that also incorporated molecular data (e.g. Wiklund et al. 2019, Bonifácio and Menot 2019, Neal et al. 2022a, Neal et al. 2022b, Bonifácio et al. 2021), over 80% of the CCZ-collected annelids are likely new to science (Rabone et al. 2023b). To date, publications from the areas targeted in this study have yielded 60 annelid species from 327 specimen records, of which only 18 species have been formalised as new (Wiklund et al. 2019, Drennan et al. 2021, Neal et al. 2022a, Neal et al. 2022b). However, we are left with a problem in that many specimens are in sub-optimal morphological condition and unable, therefore, to be formally described. Therefore, in order to ensure that this material is accessible and useful as more comparative material is recovered in future surveys and that the DNA sequences obtained are linked transparently to openly-available molecular tissue/DNA and specimen vouchers, we are making these data and materials available in the form of this checklist paper. This has two major advantages: firstly, it enables future taxonomic work with better materials to use and to link to these data and potentially describe more of these specimens; secondly, it provides openly available data on the actual species hypotheses used to support ecological papers describing the biodiversity of the CCZ (Rabone et al. 2023b), which clearly suffer from a lack of quality control in the specimen identifications available, as has been recently acknowledged (Bianchi and Gonçalves 2021, Washburn et al. 2021). This checklist also provides supporting data to Stewart et al. (2023) describing the biodiversity and connectivity of annelids across the entire eastern CCZ region.

This paper includes a checklist of 129 annelid species, with 339 records from 18 polychaete families and four species of Sipuncula. Excluded from this study are several annelid families which are common in the CCZ, but are either ones that have already been studied (Wiklund et al. 2019, Drennan et al. 2021, Neal et al. 2022a, Neal et al. 2022b) or are currently in preparation in separate detailed taxonomic papers (e.g. order Phyllodocida and families Acrocirridae and Serpulidae).

## Materials and methods

### Sampling

**Figure 1.**
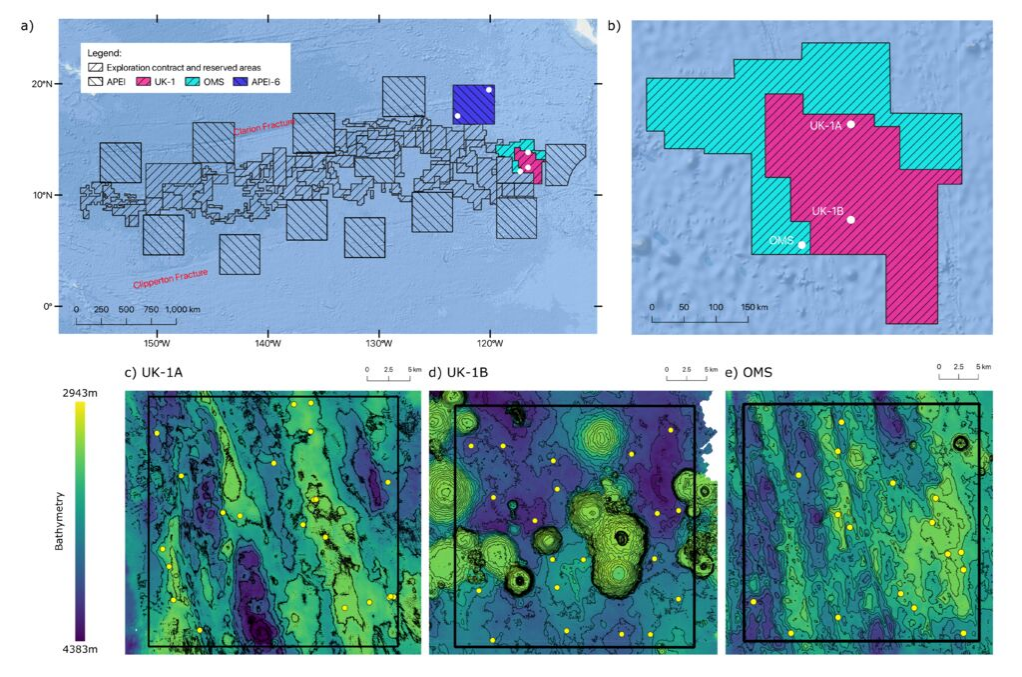
Map of ABYSSLINE sampling locations used in this study. a) The Clarion-Clipperton Zone, central Pacific Ocean, with contract areas and Areas of Particular Environmental Interest highlighted; b) UK-1 and OMS-1 sampling locations used in this study; c-e) Bathymetric data for sites in UK-1 and OMS-1 claim areas, with yellow circles showing sampling locations.

The first UKSR ABYSSLINE cruise (AB01), sampling the UK-1 exploration contract area, took place in October 2013 aboard the RV *Melville* and the second cruise (AB02), sampling the UK-1 and OMS-1 (Ocean Minerals Singapore) exploration contract areas and one Area of Particular Environmental Interest (APEI), APEI-6, took place in February-March 2015 onboard RV *Thomas G. Thompson.* Sampling locations are as indicated in Fig. 1.

A comprehensive description of our methods is provided in Glover et al. (2015). In summary, deep-sea benthic specimens from the targeted CCZ areas were collected using a range of oceanographic sampling gear including box core, epibenthic sledge (EBS), ROV and multiple core. Geographic data from sampling activities were recorded on a central GIS database. Live-sorting of specimen samples was carried out aboard both vessels in a ‘cold-chain’ pipeline, in which material was constantly maintained in chilled, filtered seawater held at 2–4°C. Specimens were preliminarily identified at sea and imaged live using stereomicroscopes with attached digital cameras. Only annelid specimens with a visible head end were counted and included in taxonomic and biodiversity analyses. The specimens were then stored in individual microtube vials containing an aqueous solution of 80% non-denatured ethanol, numbered and barcoded into a database and kept chilled until return to the Natural History Museum (NHM), London, UK.

### Laboratory Work

In the laboratory, specimens were re-examined using stereomicroscopes, identified to the best possible taxonomic level and a small tissue sample was taken for DNA extraction. In most of the specimens in this study, the material was too poor or degraded to make species-level identifications.

Extraction of DNA was done with DNeasy Blood and Tissue Kit (Qiagen) using a Hamilton Microlab STAR Robotic Workstation. About 450 bp of 16S and 650 bp of cytochrome c oxidase subunit I (COI) were amplified using primers listed in Table 2. PCR mixtures contained 1 μl of each primer (10 μM), 2 μl template DNA and 21 μl of Red Taq DNA Polymerase 1.1X MasterMix (VWR) in a mixture of total 25 μl. The PCR amplification profile consisted of initial denaturation at 95°C for 5 min, 35 cycles of denaturation at 94°C for 45 s, annealing at 55°C for 45 s, extension at 72°C for 2 min and a final extension at 72°C for 10 min. PCR products were purified using Millipore Multiscreen 96-well PCR Purification System and sequencing was performed on an ABI 3730XL DNA Analyser (Applied Biosystems) at NHM Sequencing Facility, using the same primers as in the PCR reactions (Table 2). Overlapping sequence fragments were merged into consensus sequences using Geneious (Kearse et al. 2012) and aligned using MAFFT (Katoh 2002) for 16S and MUSCLE (Edgar 2004) for COI.

### Species delimitation and taxonomic assignments

Future studies of biogeographic and bathymetric ranges, gene-flow, extinction risks, natural history, reproductive ecology, functional ecology and geochemical interactions of CCZ species are dependent on accurate taxonomic identifications. Our more recent taxonomic studies (e.g. Glover et al. (2016), Dahlgren et al. (2016), Wiklund et al. (2017), Wiklund et al. (2019), Drennan et al. (2021), Neal et al. (2022a), Neal et al. (2022b)), applied an integrative DNA taxonomy approach, with species delimitation principally based on a phylogenetic species concept, *sensu* Donoghue (1985) coupled with morphological interpretations. However, in this broader study of all of the annelids, including many poorly-preserved or fragmented individuals, we have relied more heavily on genetic data.

**Taxonomic assignments and nomenclature**

Almost all species discriminated by the analyses could be identified to family level from morphology and this was cross-checked with the genetic data through a BLAST search. Given the poor preservation of morphology and the goals of this paper to make specimen and genetic data rapidly available for any future studies, we did not attempt to force species into genera in most instances and used an open nomenclature whereby the species are named with the best taxonomic level and a species epithet that refers to the best voucher specimen of that species. For example, Pilargidae sp. (NHM_015) is the informal species name for the species that is represented by two specimens, specimen NHM_015 being the better representative (an informal type specimen). This avoids confusion with the use of sp. A, B, C etc. where informal and confusing synonyms can easily arise.

Species were delimited, based on well-supported clades recovered from phylogenetic trees estimated separately for 16S and COI sequences. Best nucleotide substitution models for each alignment (i.e. 16S and COI) and best-fit partitions were inferred using PartitionFinder 2.0 (Lanfear et al. 2017), utilising a greedy clustering algorithm (Lanfear et al. 2012) and PhyML v.3 (Guindon et al. 2010). Phylogenetic trees were estimated using Bayesian Inference in BEAST v.2.6.2 (Bouckaert et al. 2019) under a Yule speciation model, uncorrelated relaxed clock, GTR+I+G substitution model for each partition of COI (codon positions 1, 2 and 3) and for 16S and a chain length of 100 M, sampling every 10,000. The MCMC runs were inspected convergence in Tracer v.1.7.1. The first 50 M states were discarded as burn-in and a consensus median heights tree was generated from the remaining posterior trees. Trees were visualised using FigTree v.1.4.4 and species clusters were mostly defined from very similar sequences recovered in monophyletic clades with a posterior probability of 1.

### Data Handling

The field and laboratory work created a series of databases and sample sets that are integrated into a data-management pipeline. This includes the transfer and management of data and samples between a central collections database, a molecular collections database and external repositories (GenBank, WoRMS, OBIS, GBIF, GGBN, ZooBank) through DwC archives. This provides a robust data framework to support DNA taxonomy, in which openly available data and voucher material is key to quality data standards. All DwC data present in the taxon treatments of the checklist herein are also provided as a supplementary data file to allow for ease of usage (Suppl. material 2). A further elaboration of the data pipeline is published in Glover et al. (2015).

### List of Species

A list of the species in this checklist and number of records is provided here in Table 1.

## Checklists

### Ampharetidae Malmgren, 1866

#### Ampharetidae sp. (NHM_015)

4CC11D91-A5EF-5F7E-A6F5-A53AE78E6FC0

##### Materials

- **Type status:** Other material. **Occurrence:** catalogNumber: NHMUK ANEA 2023.309; recordNumber: NHM_1893; recordedBy: Adrian Glover | Helena Wiklund | Thomas Dahlgren | Madeleine Brasier; individualCount: 1; preparations: specimen stored in 80% non-denatured ethanol aqueous solution | DNA voucher stored in buffer; otherCatalogNumbers: 0174126196; associatedSequences: OQ746731 (16S); occurrenceID: 0A7FA264-84FE-574B-BF4C-026E6BE55820; **Taxon:** taxonConceptID: Ampharetidae sp. (NHM_015); scientificName: Ampharetidae; kingdom: Animalia; phylum: Annelida; class: Polychaeta; order: Terebellida; family: Ampharetidae; taxonRank: family; scientificNameAuthorship: Malmgren, 1866; **Location:** waterBody: Pacific; stateProvince: Clarion Clipperton Zone; locality: Ocean Mineral Singapore exploration claim Stratum A; verbatimLocality: OMS Stratum A; maximumDepthInMeters: 4094; locationRemarks: Deployment EB11; at Station S10; from R/V Thomas G. Thompson Cruise no. TN319; verbatimLatitude: 12°02.49’; verbatimLongitude: 117°13.03’; decimalLatitude: 12.0415; decimalLongitude: -117.21717; geodeticDatum: WGS84; **Identification:** identifiedBy: Helena Wiklund | Lenka Neal | Thomas Dahlgren | Adrian Glover | Madeleine Brasier | Regan Drennan | Eva Stewart; dateIdentified: 2021-04-20; identificationRemarks: identified by DNA and morphology; **Event:** eventID: OMS1_AB02_EB11; samplingProtocol: Brenke Epibenthic Sledge; eventDate: 2015-03-13; habitat: Abyssal plain; fieldNotes: Collected from nodule (on the epibenthic sledge); **Record Level:** language: en; institutionCode: NHMUK; collectionCode: ZOO; datasetName: ABYSSLINE; basisOfRecord: PreservedSpecimen
- **Type status:** Other material. **Occurrence:** catalogNumber: NHMUK ANEA 2023.308; recordNumber: NHM_0015; recordedBy: Adrian Glover | Helena Wiklund | Thomas Dahlgren | Magdalena Georgieva; individualCount: 1; preparations: specimen stored in 80% non-denatured ethanol aqueous solution | DNA voucher stored in buffer; otherCatalogNumbers: 0174127342; associatedSequences: OQ746466 (16S) | OQ746789 (18S) | OQ738494 (COI); occurrenceID: 667791B3-AD99-567E-A080-F41C3123770A; **Taxon:** taxonConceptID: Ampharetidae sp. (NHM_015); scientificName: Ampharetidae; kingdom: Animalia; phylum: Annelida; class: Polychaeta; order: Terebellida; family: Ampharetidae; taxonRank: family; scientificNameAuthorship: Malmgren, 1866; **Location:** waterBody: Pacific; stateProvince: Clarion Clipperton Zone; locality: UK Seabed Resources Ltd exploration area UK-1 Stratum A; verbatimLocality: UK1 Stratum A; maximumDepthInMeters: 4336; locationRemarks: Deployment EB01; at Station B-K-E; from R/V Melville Cruise no. MV1313; verbatimLatitude: 13°50.232; verbatimLongitude: 116°33.506; decimalLatitude: 13.8372; decimalLongitude: -116.55843; geodeticDatum: WGS84; **Identification:** identifiedBy: Helena Wiklund | Lenka Neal | Thomas Dahlgren | Adrian Glover | Madeleine Brasier | Regan Drennan | Eva Stewart; dateIdentified: 2021-04-20; identificationRemarks: identified by DNA and morphology; **Event:** eventID: UK1_AB01_EB01; samplingProtocol: Brenke Epibenthic Sledge; eventDate: 2013-10-09; eventTime: 10:26; habitat: Abyssal plain; fieldNotes: Collected from epi net (on the epibenthic sledge); **Record Level:** language: en; institutionCode: NHMUK; collectionCode: ZOO; datasetName: ABYSSLINE; basisOfRecord: PreservedSpecimen

##### Distribution

Eastern Clarion-Clipperton Zone, central Pacific ocean.

##### Diagnosis

Damaged specimens (Fig. 2) consistent with placement within family Ampharetidae, based on morphology and DNA.

#### Ampharetidae sp. (NHM_044)

E5CD50BD-0B90-569D-8472-EFEE536636C8

##### Materials

- **Type status:** Other material. **Occurrence:** catalogNumber: NHMUK ANEA 2023.311; recordNumber: NHM_1947I; recordedBy: Adrian Glover | Helena Wiklund | Thomas Dahlgren | Madeleine Brasier; individualCount: 1; preparations: specimen stored in 80% non-denatured ethanol aqueous solution | DNA voucher stored in buffer; otherCatalogNumbers: 0174126173; associatedSequences: OQ746738 (16S); occurrenceID: 65B5ECB4-3223-5FDE-A716-44F1E8C3A680; **Taxon:** taxonConceptID: Ampharetidae sp. (NHM_044); scientificName: Ampharetidae; kingdom: Animalia; phylum: Annelida; class: Polychaeta; order: Terebellida; family: Ampharetidae; taxonRank: family; scientificNameAuthorship: Malmgren, 1866; **Location:** waterBody: Pacific; stateProvince: Clarion Clipperton Zone; locality: Ocean Mineral Singapore exploration claim Stratum A; verbatimLocality: OMS Stratum A; maximumDepthInMeters: 4094; locationRemarks: Deployment EB11; at Station S10; from R/V Thomas G. Thompson Cruise no. TN319; verbatimLatitude: 12°02.49’; verbatimLongitude: 117°13.03’; decimalLatitude: 12.0415; decimalLongitude: -117.21717; geodeticDatum: WGS84; **Identification:** identifiedBy: Helena Wiklund | Lenka Neal | Thomas Dahlgren | Adrian Glover | Madeleine Brasier | Regan Drennan | Eva Stewart; dateIdentified: 2021-04-20; identificationRemarks: identified by DNA and morphology; **Event:** eventID: OMS1_AB02_EB11; samplingProtocol: Brenke Epibenthic Sledge; eventDate: 2015-03-13; habitat: Abyssal plain; fieldNotes: Collected from epi net (on the epibenthic sledge); **Record Level:** language: en; institutionCode: NHMUK; collectionCode: ZOO; datasetName: ABYSSLINE; basisOfRecord: PreservedSpecimen
- **Type status:** Other material. **Occurrence:** catalogNumber: NHMUK ANEA 2023.312; recordNumber: NHM_2769; recordedBy: Adrian Glover | Helena Wiklund | Thomas Dahlgren | Madeleine Brasier; individualCount: 1; preparations: specimen stored in 80% non-denatured ethanol aqueous solution | DNA voucher stored in buffer; otherCatalogNumbers: 0174126158; associatedSequences: OQ746783 (16S); occurrenceID: 8DEE847A-E775-5B20-B2F7-52219A9BCE98; **Taxon:** taxonConceptID: Ampharetidae sp. (NHM_044); scientificName: Ampharetidae; kingdom: Animalia; phylum: Annelida; class: Polychaeta; order: Terebellida; family: Ampharetidae; taxonRank: family; scientificNameAuthorship: Malmgren, 1866; **Location:** waterBody: Pacific; stateProvince: Clarion Clipperton Zone; locality: Ocean Mineral Singapore exploration claim Stratum A; verbatimLocality: OMS Stratum A; maximumDepthInMeters: 4100; locationRemarks: Deployment EB05; at Station S2; from R/V Thomas G. Thompson Cruise no. TN319; verbatimLatitude: 12'06.93; verbatimLongitude: 117'09.87; decimalLatitude: 12.1155; decimalLongitude: -117.1645; geodeticDatum: WGS84; **Identification:** identifiedBy: Helena Wiklund | Lenka Neal | Thomas Dahlgren | Adrian Glover | Madeleine Brasier | Regan Drennan | Eva Stewart; dateIdentified: 2021-04-20; identificationRemarks: identified by DNA and morphology; **Event:** eventID: OMS1_AB02_EB05; samplingProtocol: Brenke Epibenthic Sledge; eventDate: 2015-02-26; eventTime: 21:29; habitat: Abyssal plain; fieldNotes: Collected from supra net (on the epibenthic sledge); **Record Level:** language: en; institutionCode: NHMUK; collectionCode: ZOO; datasetName: ABYSSLINE; basisOfRecord: PreservedSpecimen
- **Type status:** Other material. **Occurrence:** catalogNumber: NHMUK ANEA 2023.310; recordNumber: NHM_0044; recordedBy: Adrian Glover | Helena Wiklund | Thomas Dahlgren | Magdalena Georgieva; individualCount: 1; preparations: specimen stored in 80% non-denatured ethanol aqueous solution | DNA voucher stored in buffer; otherCatalogNumbers: 0174127318; associatedSequences: OQ746470 (16S) | OQ746793 (18S); occurrenceID: EB0ED06F-AEFF-5006-8181-F1C25FE55726; **Taxon:** taxonConceptID: Ampharetidae sp. (NHM_044); scientificName: Ampharetidae; kingdom: Animalia; phylum: Annelida; class: Polychaeta; order: Terebellida; family: Ampharetidae; taxonRank: family; scientificNameAuthorship: Malmgren, 1866; **Location:** waterBody: Pacific; stateProvince: Clarion Clipperton Zone; locality: UK Seabed Resources Ltd exploration area UK-1 Stratum A; verbatimLocality: UK1 Stratum A; maximumDepthInMeters: 4336; locationRemarks: Deployment EB01; at Station B-K-E; from R/V Melville Cruise no. MV1313; verbatimLatitude: 13°50.232; verbatimLongitude: 116°33.506; decimalLatitude: 13.8372; decimalLongitude: -116.55843; geodeticDatum: WGS84; **Identification:** identifiedBy: Helena Wiklund | Lenka Neal | Thomas Dahlgren | Adrian Glover | Madeleine Brasier | Regan Drennan | Eva Stewart; dateIdentified: 2021-04-20; identificationRemarks: identified by DNA and morphology; **Event:** eventID: UK1_AB01_EB01; samplingProtocol: Brenke Epibenthic Sledge; eventDate: 2013-10-09; eventTime: 10:26; habitat: Abyssal plain; fieldNotes: Collected from epi net (on the epibenthic sledge); **Record Level:** language: en; institutionCode: NHMUK; collectionCode: ZOO; datasetName: ABYSSLINE; basisOfRecord: PreservedSpecimen

##### Distribution

Eastern Clarion-Clipperton Zone, central Pacific ocean.

##### Diagnosis

Damaged specimens (Fig. 3) consistent with Ampharetidae, based on morphology and DNA.

#### Ampharetidae sp. (NHM_062)

519EC066-3EBC-58E5-9686-9BCD223ACFF4

##### Materials

- **Type status:** Other material. **Occurrence:** catalogNumber: NHMUK ANEA 2023.314; recordNumber: NHM_2541; recordedBy: Adrian Glover | Helena Wiklund | Thomas Dahlgren | Madeleine Brasier; individualCount: 1; preparations: specimen stored in 80% non-denatured ethanol aqueous solution | DNA voucher stored in buffer; otherCatalogNumbers: 0174126183; associatedSequences: OQ746779 (16S) | OQ738616 (COI); occurrenceID: 9883D76D-3710-56C8-B231-665FDB2833BA; **Taxon:** taxonConceptID: Ampharetidae sp. (NHM_062); scientificName: Ampharetidae; kingdom: Animalia; phylum: Annelida; class: Polychaeta; order: Terebellida; family: Ampharetidae; taxonRank: family; scientificNameAuthorship: Malmgren, 1866; **Location:** waterBody: Pacific; stateProvince: Clarion Clipperton Zone; locality: UK Seabed Resources Ltd exploration area UK-1 Stratum B; verbatimLocality: UK1 Stratum B; maximumDepthInMeters: 4233; locationRemarks: Deployment EB09; at Station U1; from R/V Thomas G. Thompson Cruise no. TN319; verbatimLatitude: 12'21.81; verbatimLongitude: 116'40.86; decimalLatitude: 12.3635; decimalLongitude: -116.681; geodeticDatum: WGS84; **Identification:** identifiedBy: Helena Wiklund | Lenka Neal | Thomas Dahlgren | Adrian Glover | Madeleine Brasier | Regan Drennan | Eva Stewart; dateIdentified: 2021-04-20; identificationRemarks: identified by DNA and morphology; **Event:** eventID: UK1_AB02_EB09; samplingProtocol: Brenke Epibenthic Sledge; eventDate: 2015-03-10; eventTime: 10:46; habitat: Abyssal plain; fieldNotes: Collected from supra net (on the epibenthic sledge); **Record Level:** language: en; institutionCode: NHMUK; collectionCode: ZOO; datasetName: ABYSSLINE; basisOfRecord: PreservedSpecimen
- **Type status:** Other material. **Occurrence:** catalogNumber: NHMUK ANEA 2023.313; recordNumber: NHM_0062; recordedBy: Adrian Glover | Helena Wiklund | Thomas Dahlgren | Magdalena Georgieva; individualCount: 1; preparations: specimen stored in 80% non-denatured ethanol aqueous solution | DNA voucher stored in buffer; otherCatalogNumbers: 0174127389; associatedSequences: OQ746474 (16S) | OQ738499 (COI); occurrenceID: D15497A0-76F6-523E-A84B-77C3F4685510; **Taxon:** taxonConceptID: Ampharetidae sp. (NHM_062); scientificName: Ampharetidae; kingdom: Animalia; phylum: Annelida; class: Polychaeta; order: Terebellida; family: Ampharetidae; taxonRank: family; scientificNameAuthorship: Malmgren, 1866; **Location:** waterBody: Pacific; stateProvince: Clarion Clipperton Zone; locality: UK Seabed Resources Ltd exploration area UK-1 Stratum A; verbatimLocality: UK1 Stratum A; maximumDepthInMeters: 4108; locationRemarks: Deployment BC04; at Station B; from R/V Melville Cruise no. MV1313; verbatimLatitude: 13°50.993; verbatimLongitude: 116°38.697; decimalLatitude: 13.84988; decimalLongitude: -116.64495; geodeticDatum: WGS84; **Identification:** identifiedBy: Helena Wiklund | Lenka Neal | Thomas Dahlgren | Adrian Glover | Madeleine Brasier | Regan Drennan | Eva Stewart; dateIdentified: 2021-04-20; identificationRemarks: identified by DNA and morphology; **Event:** eventID: UK1_AB01_BC04; samplingProtocol: USNEL Box Core; eventDate: 2013-10-09; eventTime: 17:34; habitat: Abyssal plain; fieldNotes: Collected from 0-2 cm layer of box core using a 300 micron sieve; **Record Level:** language: en; institutionCode: NHMUK; collectionCode: ZOO; datasetName: ABYSSLINE; basisOfRecord: PreservedSpecimen

##### Distribution

Eastern Clarion-Clipperton Zone, central Pacific Ocean.

##### Diagnosis

**Figure 4.**
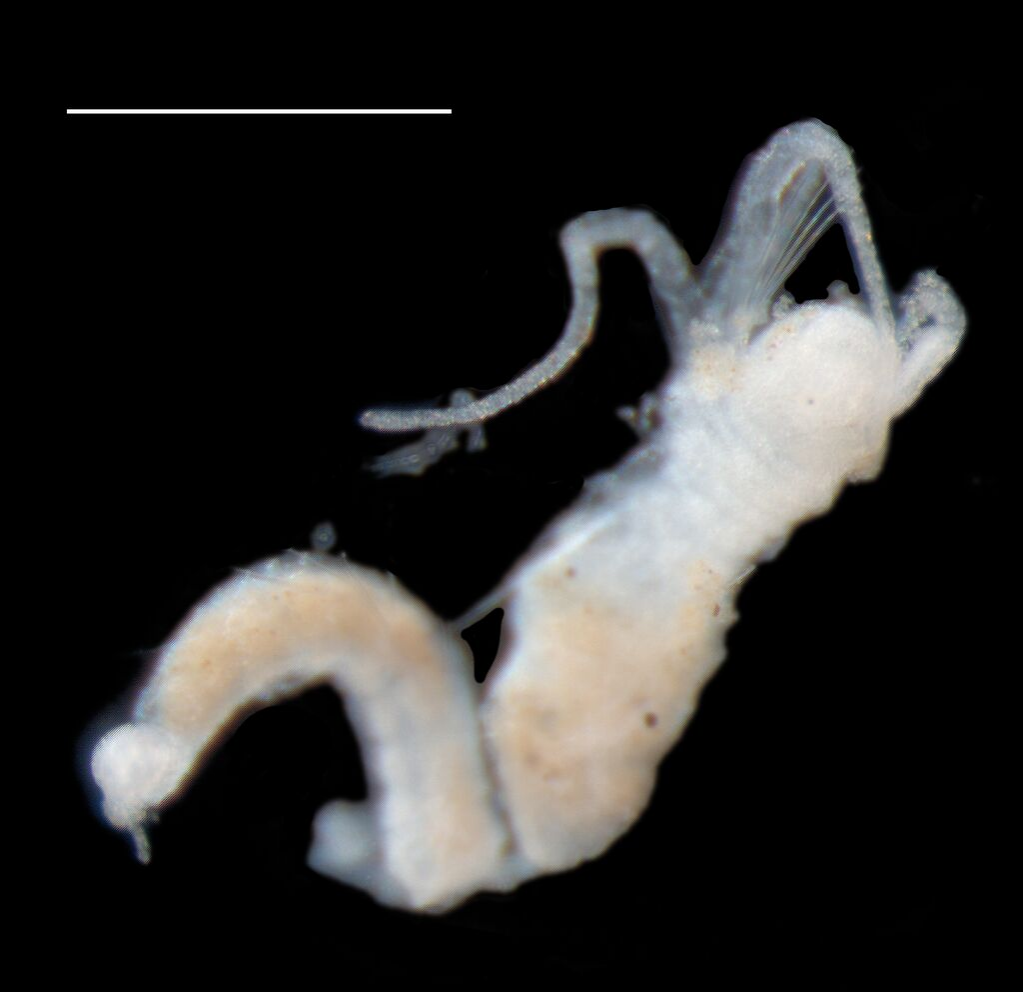
Ampharetidae sp. (NHM_062), specimen NHM_062 in lateral view. Scale bar 500 µm.

Damaged specimen (Fig. 4) consistent with placement within family Ampharetidae, based on morphology and DNA.

#### Ampharetidae sp. (NHM_1338)

BCF47C41-4013-51BA-B802-8FB801D19176

##### Materials

- **Type status:** Other material. **Occurrence:** catalogNumber: NHMUK ANEA 2023.317; recordNumber: NHM_1338; recordedBy: Adrian Glover | Helena Wiklund | Thomas Dahlgren | Madeleine Brasier; individualCount: 1; preparations: specimen stored in 80% non-denatured ethanol aqueous solution | DNA voucher stored in buffer; otherCatalogNumbers: 0174126541; associatedSequences: OQ746662 (16S) | OQ746890 (18S); occurrenceID: 8198ADBC-A581-5F1A-AEE6-C1FEC1F8F6E2; **Taxon:** taxonConceptID: Ampharetidae sp. (NHM_1338); scientificName: Ampharetidae; kingdom: Animalia; phylum: Annelida; class: Polychaeta; order: Terebellida; family: Ampharetidae; taxonRank: family; scientificNameAuthorship: Malmgren, 1866; **Location:** waterBody: Pacific; stateProvince: Clarion Clipperton Zone; locality: Ocean Mineral Singapore exploration claim Stratum A; verbatimLocality: OMS Stratum A; maximumDepthInMeters: 4302; locationRemarks: Deployment EB06; at Station S5; from R/V Thomas G. Thompson Cruise no. TN319; verbatimLatitude: 12'15.44; verbatimLongitude: 117'18.13; decimalLatitude: 12.25733; decimalLongitude: -117.30217; geodeticDatum: WGS84; **Identification:** identifiedBy: Helena Wiklund | Lenka Neal | Thomas Dahlgren | Adrian Glover | Madeleine Brasier | Regan Drennan | Eva Stewart; dateIdentified: 2021-04-20; identificationRemarks: identified by DNA and morphology; **Event:** eventID: OMS1_AB02_EB06; samplingProtocol: Brenke Epibenthic Sledge; eventDate: 2015-03-01; eventTime: 04:02; habitat: Abyssal plain; fieldNotes: Collected from epi net (on the epibenthic sledge); **Record Level:** language: en; institutionCode: NHMUK; collectionCode: ZOO; datasetName: ABYSSLINE; basisOfRecord: PreservedSpecimen
- **Type status:** Other material. **Occurrence:** catalogNumber: NHMUK ANEA 2023.318; recordNumber: NHM_1347H; recordedBy: Adrian Glover | Helena Wiklund | Thomas Dahlgren | Madeleine Brasier; individualCount: 1; preparations: specimen stored in 80% non-denatured ethanol aqueous solution | DNA voucher stored in buffer; otherCatalogNumbers: 0174126538; associatedSequences: OQ746666 (16S); occurrenceID: D26AEACE-F406-5672-B690-5BF0ADB022F8; **Taxon:** taxonConceptID: Ampharetidae sp. (NHM_1338); scientificName: Ampharetidae; kingdom: Animalia; phylum: Annelida; class: Polychaeta; order: Terebellida; family: Ampharetidae; taxonRank: family; scientificNameAuthorship: Malmgren, 1866; **Location:** waterBody: Pacific; stateProvince: Clarion Clipperton Zone; locality: Ocean Mineral Singapore exploration claim Stratum A; verbatimLocality: OMS Stratum A; maximumDepthInMeters: 4302; locationRemarks: Deployment EB06; at Station S5; from R/V Thomas G. Thompson Cruise no. TN319; verbatimLatitude: 12'15.44; verbatimLongitude: 117'18.13; decimalLatitude: 12.25733; decimalLongitude: -117.30217; geodeticDatum: WGS84; **Identification:** identifiedBy: Helena Wiklund | Lenka Neal | Thomas Dahlgren | Adrian Glover | Madeleine Brasier | Regan Drennan | Eva Stewart; dateIdentified: 2021-04-20; identificationRemarks: identified by DNA and morphology; **Event:** eventID: OMS1_AB02_EB06; samplingProtocol: Brenke Epibenthic Sledge; eventDate: 2015-03-01; eventTime: 04:02; habitat: Abyssal plain; fieldNotes: Collected from epi net (on the epibenthic sledge); **Record Level:** language: en; institutionCode: NHMUK; collectionCode: ZOO; datasetName: ABYSSLINE; basisOfRecord: PreservedSpecimen
- **Type status:** Other material. **Occurrence:** catalogNumber: NHMUK ANEA 2023.319; recordNumber: NHM_1480I; recordedBy: Adrian Glover | Helena Wiklund | Thomas Dahlgren | Madeleine Brasier; individualCount: 1; preparations: specimen stored in 80% non-denatured ethanol aqueous solution | DNA voucher stored in buffer; otherCatalogNumbers: 0174126167; associatedSequences: OQ746686 (16S); occurrenceID: EAE6F368-F2E1-56C4-9EF4-94BCCCF2E699; **Taxon:** taxonConceptID: Ampharetidae sp. (NHM_1338); scientificName: Ampharetidae; kingdom: Animalia; phylum: Annelida; class: Polychaeta; order: Terebellida; family: Ampharetidae; taxonRank: family; scientificNameAuthorship: Malmgren, 1866; **Location:** waterBody: Pacific; stateProvince: Clarion Clipperton Zone; locality: UK Seabed Resources Ltd exploration area UK-1 Stratum B; verbatimLocality: UK1 Stratum B; maximumDepthInMeters: 4137; locationRemarks: Deployment EB07; at Station U7; from R/V Thomas G. Thompson Cruise no. TN319; verbatimLatitude: 12'27.26; verbatimLongitude: 116'36.77; decimalLatitude: 12.45433; decimalLongitude: -116.61283; geodeticDatum: WGS84; **Identification:** identifiedBy: Helena Wiklund | Lenka Neal | Thomas Dahlgren | Adrian Glover | Madeleine Brasier | Regan Drennan | Eva Stewart; dateIdentified: 2021-04-20; identificationRemarks: identified by DNA and morphology; **Event:** eventID: UK1_AB02_EB07; samplingProtocol: Brenke Epibenthic Sledge; eventDate: 2015-03-03; eventTime: 20:40; habitat: Abyssal plain; fieldNotes: Collected from epi net (on the epibenthic sledge); **Record Level:** language: en; institutionCode: NHMUK; collectionCode: ZOO; datasetName: ABYSSLINE; basisOfRecord: PreservedSpecimen

##### Distribution

Eastern Clarion-Clipperton Zone, central Pacific Ocean.

##### Diagnosis

**Figure 5.**
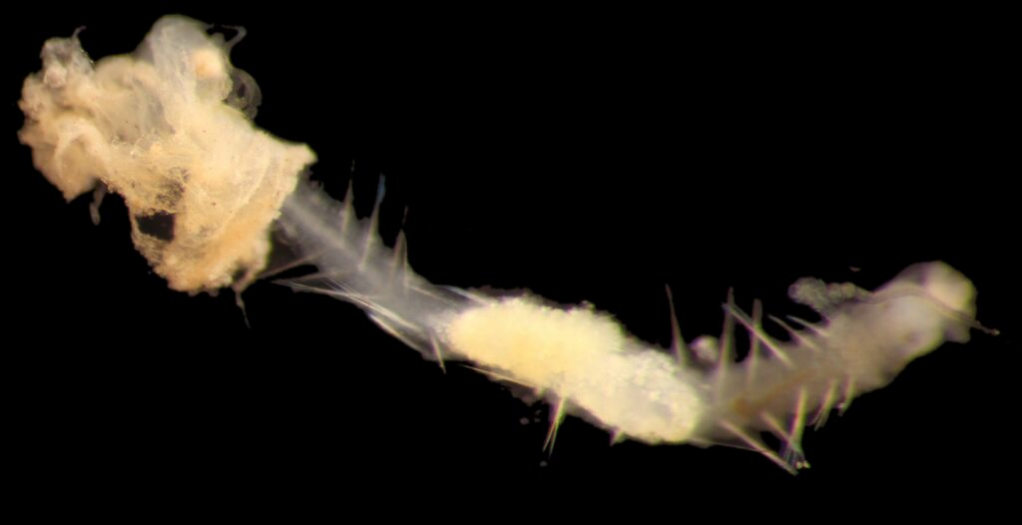
Ampharetidae sp. (NHM_1338), live specimen NHM_1338.

Damaged specimen (Fig. 5) consistent with placement within family Ampharetidae, based on morphology and DNA.

#### Ampharetidae sp. (NHM_1161)

61E657FC-E790-5737-84A2-2AF22D2AB620

##### Materials

- **Type status:** Other material. **Occurrence:** catalogNumber: NHMUK ANEA 2023.316; recordNumber: NHM_1161; recordedBy: Adrian Glover | Helena Wiklund | Thomas Dahlgren | Madeleine Brasier; individualCount: 1; preparations: specimen stored in 80% non-denatured ethanol aqueous solution | DNA voucher stored in buffer; otherCatalogNumbers: 0174126544; associatedSequences: OQ746629 (16S); occurrenceID: FACA35E6-347A-54CA-8D19-6A9C5CA0D813; **Taxon:** taxonConceptID: Ampharetidae sp. (NHM_1161); scientificName: Ampharetidae; kingdom: Animalia; phylum: Annelida; class: Polychaeta; order: Terebellida; family: Ampharetidae; taxonRank: family; scientificNameAuthorship: Malmgren, 1866; **Location:** waterBody: Pacific; stateProvince: Clarion Clipperton Zone; locality: Ocean Mineral Singapore exploration claim Stratum A; verbatimLocality: OMS Stratum A; maximumDepthInMeters: 4100; locationRemarks: Deployment EB05; at Station S2; from R/V Thomas G. Thompson Cruise no. TN319; verbatimLatitude: 12'06.93; verbatimLongitude: 117'09.87; decimalLatitude: 12.1155; decimalLongitude: -117.1645; geodeticDatum: WGS84; **Identification:** identifiedBy: Helena Wiklund | Lenka Neal | Thomas Dahlgren | Adrian Glover | Madeleine Brasier | Regan Drennan | Eva Stewart; dateIdentified: 2021-04-20; identificationRemarks: identified by DNA and morphology; **Event:** eventID: OMS1_AB02_EB05; samplingProtocol: Brenke Epibenthic Sledge; eventDate: 2015-02-26; eventTime: 21:29; habitat: Abyssal plain; fieldNotes: Collected from epi net (on the epibenthic sledge); **Record Level:** language: en; institutionCode: NHMUK; collectionCode: ZOO; datasetName: ABYSSLINE; basisOfRecord: PreservedSpecimen

##### Distribution

Eastern Clarion-Clipperton Zone, central Pacific Ocean.

##### Diagnosis

**Figure 6.**
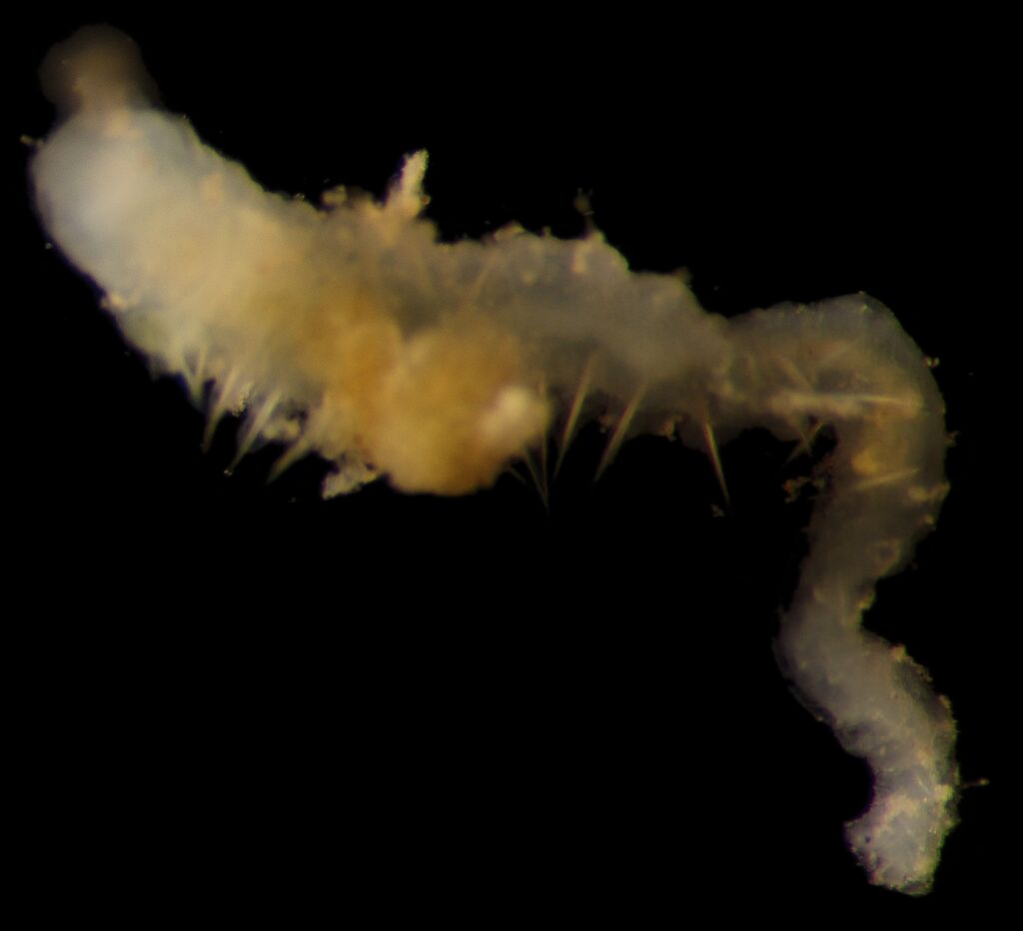
Ampharetidae sp. (NHM_1161), live specimen NHM_1661 in dorsal view.

Damaged specimen (Fig. 6) consistent with placement within family Ampharetidae, based on morphology and DNA.

#### Ampharetidae sp. (NHM_789)

DF581B50-21BD-56EF-A49D-DBD780AB3539

##### Materials

- **Type status:** Other material. **Occurrence:** recordNumber: NHM_1177; recordedBy: Adrian Glover | Helena Wiklund | Thomas Dahlgren | Madeleine Brasier; individualCount: 1; preparations: Tissue voucher stored in 80% non-denatured ethanol aqueous solution | DNA voucher stored in buffer; otherCatalogNumbers: 0109405353 | 0174126590; associatedSequences: OQ746639 (16S) | OQ738569 (COI); occurrenceID: 8C5D805A-89AF-58C2-A177-310CBF2D9BD6; **Taxon:** taxonConceptID: Ampharetidae sp. (NHM_789); scientificName: Ampharetidae; kingdom: Animalia; phylum: Annelida; class: Polychaeta; order: Terebellida; family: Ampharetidae; taxonRank: family; scientificNameAuthorship: Malmgren, 1866; **Location:** waterBody: Pacific; stateProvince: Clarion Clipperton Zone; locality: Ocean Mineral Singapore exploration claim Stratum A; verbatimLocality: OMS Stratum A; maximumDepthInMeters: 4100; locationRemarks: Deployment EB05; at Station S2; from R/V Thomas G. Thompson Cruise no. TN319; verbatimLatitude: 12'06.93; verbatimLongitude: 117'09.87; decimalLatitude: 12.1155; decimalLongitude: -117.1645; geodeticDatum: WGS84; **Identification:** identifiedBy: Helena Wiklund | Lenka Neal | Thomas Dahlgren | Adrian Glover | Madeleine Brasier | Regan Drennan | Eva Stewart; dateIdentified: 2021-04-20; identificationRemarks: identified by DNA and morphology; **Event:** eventID: OMS1_AB02_EB05; samplingProtocol: Brenke Epibenthic Sledge; eventDate: 2015-02-26; eventTime: 21:29; habitat: Abyssal plain; fieldNotes: Collected from epi net (on the epibenthic sledge); **Record Level:** language: en; institutionCode: NHMUK; collectionCode: ZOO; datasetName: ABYSSLINE; basisOfRecord: PreservedSpecimen
- **Type status:** Other material. **Occurrence:** catalogNumber: NHMUK ANEA 2023.332; recordNumber: NHM_0870; recordedBy: Adrian Glover | Helena Wiklund | Thomas Dahlgren | Madeleine Brasier; individualCount: 1; preparations: specimen stored in 80% non-denatured ethanol aqueous solution | DNA voucher stored in buffer; otherCatalogNumbers: 0174126619; associatedSequences: OQ746569 (16S); occurrenceID: 0DE37950-8866-5263-AB15-201BEBA06625; **Taxon:** taxonConceptID: Ampharetidae sp. (NHM_789); scientificName: Ampharetidae; kingdom: Animalia; phylum: Annelida; class: Polychaeta; order: Terebellida; family: Ampharetidae; taxonRank: family; scientificNameAuthorship: Malmgren, 1866; **Location:** waterBody: Pacific; stateProvince: Clarion Clipperton Zone; locality: UK Seabed Resources Ltd exploration area UK-1 Stratum B; verbatimLocality: UK1 Stratum B; maximumDepthInMeters: 4237; locationRemarks: Deployment BC06; at Station U6; from R/V Thomas G. Thompson Cruise no. TN319; verbatimLatitude: 12'34.742; verbatimLongitude: 116'41.218; decimalLatitude: 12.57903; decimalLongitude: -116.68697; geodeticDatum: WGS84; **Identification:** identifiedBy: Helena Wiklund | Lenka Neal | Thomas Dahlgren | Adrian Glover | Madeleine Brasier | Regan Drennan | Eva Stewart; dateIdentified: 2021-04-20; identificationRemarks: identified by DNA and morphology; **Event:** eventID: UK1_AB02_BC06; samplingProtocol: USNEL Box Core; eventDate: 2015-02-22; eventTime: 05:08; habitat: Abyssal plain; fieldNotes: Collected from nodule in box core sample; **Record Level:** language: en; institutionCode: NHMUK; collectionCode: ZOO; datasetName: ABYSSLINE; basisOfRecord: PreservedSpecimen
- **Type status:** Other material. **Occurrence:** catalogNumber: NHMUK ANEA 2023.331; recordNumber: NHM_0789; recordedBy: Adrian Glover | Helena Wiklund | Thomas Dahlgren | Madeleine Brasier; individualCount: 1; preparations: specimen stored in 80% non-denatured ethanol aqueous solution | DNA voucher stored in buffer; otherCatalogNumbers: 0174126596; associatedSequences: OQ746561 (16S) | OQ746860 (18S); occurrenceID: D791260E-EDCE-5FF5-9BF5-9D7223F1C860; **Taxon:** taxonConceptID: Ampharetidae sp. (NHM_789); scientificName: Ampharetidae; kingdom: Animalia; phylum: Annelida; class: Polychaeta; order: Terebellida; family: Ampharetidae; taxonRank: family; scientificNameAuthorship: Malmgren, 1866; **Location:** waterBody: Pacific; stateProvince: Clarion Clipperton Zone; locality: UK Seabed Resources Ltd exploration area UK-1 Stratum B; verbatimLocality: UK1 Stratum B; maximumDepthInMeters: 4425; locationRemarks: Deployment EB02; at Station U5; from R/V Thomas G. Thompson Cruise no. TN319; verbatimLatitude: 12'32.23; verbatimLongitude: 116'36.25; decimalLatitude: 12.53717; decimalLongitude: -116.60417; geodeticDatum: WGS84; **Identification:** identifiedBy: Helena Wiklund | Lenka Neal | Thomas Dahlgren | Adrian Glover | Madeleine Brasier | Regan Drennan | Eva Stewart; dateIdentified: 2021-04-20; identificationRemarks: identified by DNA and morphology; **Event:** eventID: UK1_AB02_EB02; samplingProtocol: Brenke Epibenthic Sledge; eventDate: 2015-02-20; eventTime: 06:24; habitat: Abyssal plain; fieldNotes: Collected from epi net (on the epibenthic sledge); **Record Level:** language: en; institutionCode: NHMUK; collectionCode: ZOO; datasetName: ABYSSLINE; basisOfRecord: PreservedSpecimen
- **Type status:** Other material. **Occurrence:** catalogNumber: NHMUK ANEA 2023.333; recordNumber: NHM_1631; recordedBy: Adrian Glover | Helena Wiklund | Thomas Dahlgren | Madeleine Brasier; individualCount: 1; preparations: specimen stored in 80% non-denatured ethanol aqueous solution | DNA voucher stored in buffer; otherCatalogNumbers: 0174126237; associatedSequences: OQ746698 (16S); occurrenceID: 664C4AA4-243C-54FF-A390-80608E891287; **Taxon:** taxonConceptID: Ampharetidae sp. (NHM_789); scientificName: Ampharetidae; kingdom: Animalia; phylum: Annelida; class: Polychaeta; order: Terebellida; family: Ampharetidae; taxonRank: family; scientificNameAuthorship: Malmgren, 1866; **Location:** waterBody: Pacific; stateProvince: Clarion Clipperton Zone; locality: UK Seabed Resources Ltd exploration area UK-1 Stratum B; verbatimLocality: UK1 Stratum B; maximumDepthInMeters: 4258; locationRemarks: Deployment BC20; at Station U13; from R/V Thomas G. Thompson Cruise no. TN319; verbatimLatitude: 12'35.813; verbatimLongitude: 116'29.614; decimalLatitude: 12.59688; decimalLongitude: -116.49357; geodeticDatum: WGS84; **Identification:** identifiedBy: Helena Wiklund | Lenka Neal | Thomas Dahlgren | Adrian Glover | Madeleine Brasier | Regan Drennan | Eva Stewart; dateIdentified: 2021-04-20; identificationRemarks: identified by DNA and morphology; **Event:** eventID: UK1_AB02_BC20; samplingProtocol: USNEL Box Core; eventDate: 2015-03-09; habitat: Abyssal plain; fieldNotes: Collected from nodule in box core sample; **Record Level:** language: en; institutionCode: NHMUK; collectionCode: ZOO; datasetName: ABYSSLINE; basisOfRecord: PreservedSpecimen

##### Distribution

Eastern Clarion-Clipperton Zone, central Pacific Ocean.

##### Diagnosis

**Figure 7.**
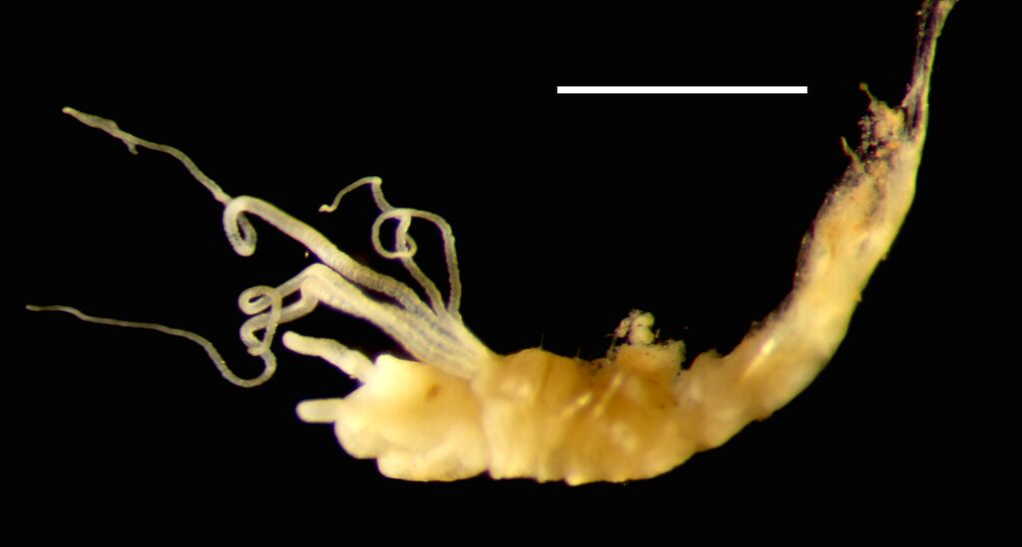
Ampharetidae sp. (NHM_789), live specimen NHM_870 in lateral view. Scale bar: 1 mm.

Damaged specimen (Fig. 7) consistent with placement within family Ampharetidae, based on morphology and DNA.

#### Ampharetidae sp. (NHM_265)

3A7A1259-2554-5C7E-BC0D-8BF622848A04

##### Materials

- **Type status:** Other material. **Occurrence:** catalogNumber: NHMUK ANEA 2023.325; recordNumber: NHM_0265; recordedBy: Adrian Glover | Helena Wiklund | Thomas Dahlgren | Magdalena Georgieva; individualCount: 1; preparations: specimen stored in 80% non-denatured ethanol aqueous solution | DNA voucher stored in buffer; otherCatalogNumbers: 0174127346; associatedSequences: OQ746490 (16S) | OQ746809 (18S) | OQ738508 (COI); occurrenceID: E9B2998D-6DE9-5E70-BAA7-3027D067D1A4; **Taxon:** taxonConceptID: Ampharetidae sp. (NHM_265); scientificName: Ampharetidae; kingdom: Animalia; phylum: Annelida; class: Polychaeta; order: Terebellida; family: Ampharetidae; taxonRank: family; scientificNameAuthorship: Malmgren, 1866; **Location:** waterBody: Pacific; stateProvince: Clarion Clipperton Zone; locality: UK Seabed Resources Ltd exploration area UK-1 Stratum A; verbatimLocality: UK1 Stratum A; maximumDepthInMeters: 4128; locationRemarks: Deployment EB04; at Station G-I; from R/V Melville Cruise no. MV1313; verbatimLatitude: 13°45.21N; verbatimLongitude: 116°29.12W; decimalLatitude: 13.75583; decimalLongitude: -116.48667; geodeticDatum: WGS84; **Identification:** identifiedBy: Helena Wiklund | Lenka Neal | Thomas Dahlgren | Adrian Glover | Madeleine Brasier | Regan Drennan | Eva Stewart; dateIdentified: 2021-04-20; identificationRemarks: identified by DNA and morphology; **Event:** eventID: UK1_AB01_EB04; samplingProtocol: Brenke Epibenthic Sledge; eventDate: 2013-10-17; eventTime: 01:50; habitat: Abyssal plain; fieldNotes: Collected from epi net (on the epibenthic sledge); **Record Level:** language: en; institutionCode: NHMUK; collectionCode: ZOO; datasetName: ABYSSLINE; basisOfRecord: PreservedSpecimen

##### Distribution

Eastern Clarion-Clipperton Zone, central Pacific Ocean.

##### Diagnosis

**Figure 8.**
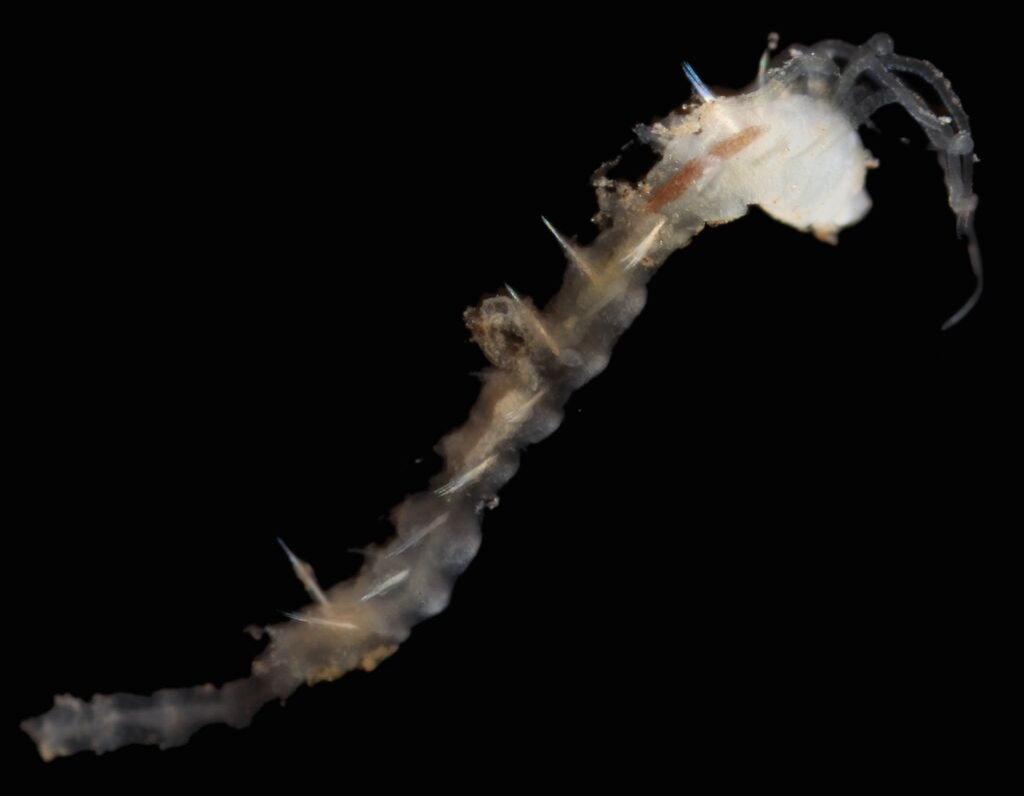
Ampharetidae sp. (NHM_265), live specimen NHM_265 in lateral view.

Damaged specimen (Fig. 8) consistent with placement within family Ampharetidae, based on morphology and DNA.

#### Ampharetidae sp. (NHM_292)

F2754E2F-8042-5314-AA05-860FFE48D98B

##### Materials

- **Type status:** Other material. **Occurrence:** catalogNumber: NHMUK ANEA 2023.328; recordNumber: NHM_1865; recordedBy: Adrian Glover | Helena Wiklund | Thomas Dahlgren | Madeleine Brasier; individualCount: 1; preparations: specimen stored in 80% non-denatured ethanol aqueous solution | DNA voucher stored in buffer; otherCatalogNumbers: 0174126147; associatedSequences: OQ746726 (16S); occurrenceID: 4250E561-2280-534F-8A33-80C168B029EB; **Taxon:** taxonConceptID: Ampharetidae sp. (NHM_292); scientificName: Ampharetidae; kingdom: Animalia; phylum: Annelida; class: Polychaeta; order: Terebellida; family: Ampharetidae; taxonRank: family; scientificNameAuthorship: Malmgren, 1866; **Location:** waterBody: Pacific; stateProvince: Clarion Clipperton Zone; locality: Ocean Mineral Singapore exploration claim Stratum A; verbatimLocality: OMS Stratum A; maximumDepthInMeters: 4094; locationRemarks: Deployment EB11; at Station S10; from R/V Thomas G. Thompson Cruise no. TN319; verbatimLatitude: 12°02.49’; verbatimLongitude: 117°13.03’; decimalLatitude: 12.0415; decimalLongitude: -117.21717; geodeticDatum: WGS84; **Identification:** identifiedBy: Helena Wiklund | Lenka Neal | Thomas Dahlgren | Adrian Glover | Madeleine Brasier | Regan Drennan | Eva Stewart; dateIdentified: 2021-04-20; identificationRemarks: identified by DNA and morphology; **Event:** eventID: OMS1_AB02_EB11; samplingProtocol: Brenke Epibenthic Sledge; eventDate: 2015-03-13; habitat: Abyssal plain; fieldNotes: Collected from epi net (on the epibenthic sledge); **Record Level:** language: en; institutionCode: NHMUK; collectionCode: ZOO; datasetName: ABYSSLINE; basisOfRecord: PreservedSpecimen
- **Type status:** Other material. **Occurrence:** catalogNumber: NHMUK ANEA 2023.329; recordNumber: NHM_1947B; recordedBy: Adrian Glover | Helena Wiklund | Thomas Dahlgren | Madeleine Brasier; individualCount: 1; preparations: specimen stored in 80% non-denatured ethanol aqueous solution | DNA voucher stored in buffer; otherCatalogNumbers: 0174126187; associatedSequences: OQ746735 (16S); occurrenceID: BD75E160-CE38-5DC9-BBE5-52BE9AE3AE52; **Taxon:** taxonConceptID: Ampharetidae sp. (NHM_292); scientificName: Ampharetidae; kingdom: Animalia; phylum: Annelida; class: Polychaeta; order: Terebellida; family: Ampharetidae; taxonRank: family; scientificNameAuthorship: Malmgren, 1866; **Location:** waterBody: Pacific; stateProvince: Clarion Clipperton Zone; locality: Ocean Mineral Singapore exploration claim Stratum A; verbatimLocality: OMS Stratum A; maximumDepthInMeters: 4094; locationRemarks: Deployment EB11; at Station S10; from R/V Thomas G. Thompson Cruise no. TN319; verbatimLatitude: 12°02.49’; verbatimLongitude: 117°13.03’; decimalLatitude: 12.0415; decimalLongitude: -117.21717; geodeticDatum: WGS84; **Identification:** identifiedBy: Helena Wiklund | Lenka Neal | Thomas Dahlgren | Adrian Glover | Madeleine Brasier | Regan Drennan | Eva Stewart; dateIdentified: 2021-04-20; identificationRemarks: identified by DNA and morphology; **Event:** eventID: OMS1_AB02_EB11; samplingProtocol: Brenke Epibenthic Sledge; eventDate: 2015-03-13; habitat: Abyssal plain; fieldNotes: Collected from epi net (on the epibenthic sledge); **Record Level:** language: en; institutionCode: NHMUK; collectionCode: ZOO; datasetName: ABYSSLINE; basisOfRecord: PreservedSpecimen
- **Type status:** Other material. **Occurrence:** catalogNumber: NHMUK ANEA 2023.327; recordNumber: NHM_1056; recordedBy: Adrian Glover | Helena Wiklund | Thomas Dahlgren | Madeleine Brasier; individualCount: 1; preparations: specimen stored in 80% non-denatured ethanol aqueous solution | DNA voucher stored in buffer; otherCatalogNumbers: 0174126534; associatedSequences: OQ746610 (16S); occurrenceID: 93C0FFA4-4E5E-585B-BCA8-61D51E4947DE; **Taxon:** taxonConceptID: Ampharetidae sp. (NHM_292); scientificName: Ampharetidae; kingdom: Animalia; phylum: Annelida; class: Polychaeta; order: Terebellida; family: Ampharetidae; taxonRank: family; scientificNameAuthorship: Malmgren, 1866; **Location:** waterBody: Pacific; stateProvince: Clarion Clipperton Zone; locality: Ocean Mineral Singapore exploration claim Stratum A; verbatimLocality: OMS Stratum A; maximumDepthInMeters: 4070; locationRemarks: Deployment BC09; at Station S2; from R/V Thomas G. Thompson Cruise no. TN319; verbatimLatitude: 12'04.914; verbatimLongitude: 117'10.691; decimalLatitude: 12.0819; decimalLongitude: -117.19283; geodeticDatum: WGS84; **Identification:** identifiedBy: Helena Wiklund | Lenka Neal | Thomas Dahlgren | Adrian Glover | Madeleine Brasier | Regan Drennan | Eva Stewart; dateIdentified: 2021-04-20; identificationRemarks: identified by DNA and morphology; **Event:** eventID: OMS1_AB02_BC09; samplingProtocol: USNEL Box Core; eventDate: 2015-02-26; eventTime: 00:37; habitat: Abyssal plain; fieldNotes: Collected from 0-2 cm layer of box core using a 300 micron sieve; **Record Level:** language: en; institutionCode: NHMUK; collectionCode: ZOO; datasetName: ABYSSLINE; basisOfRecord: PreservedSpecimen
- **Type status:** Other material. **Occurrence:** catalogNumber: NHMUK ANEA 2023.326; recordNumber: NHM_0292; recordedBy: Adrian Glover | Helena Wiklund | Thomas Dahlgren | Magdalena Georgieva; individualCount: 1; preparations: specimen stored in 80% non-denatured ethanol aqueous solution | DNA voucher stored in buffer; otherCatalogNumbers: 0174127370; associatedSequences: OQ746493 (16S) | OQ746812 (18S) | OQ738511 (COI); occurrenceID: DBD48590-7B08-583C-AD7E-97B0364CFA1A; **Taxon:** taxonConceptID: Ampharetidae sp. (NHM_292); scientificName: Ampharetidae; kingdom: Animalia; phylum: Annelida; class: Polychaeta; order: Terebellida; family: Ampharetidae; taxonRank: family; scientificNameAuthorship: Malmgren, 1866; **Location:** waterBody: Pacific; stateProvince: Clarion Clipperton Zone; locality: UK Seabed Resources Ltd exploration area UK-1 Stratum A; verbatimLocality: UK1 Stratum A; maximumDepthInMeters: 4128; locationRemarks: Deployment EB04; at Station G-I; from R/V Melville Cruise no. MV1313; verbatimLatitude: 13°45.21N; verbatimLongitude: 116°29.12W; decimalLatitude: 13.75583; decimalLongitude: -116.48667; geodeticDatum: WGS84; **Identification:** identifiedBy: Helena Wiklund | Lenka Neal | Thomas Dahlgren | Adrian Glover | Madeleine Brasier | Regan Drennan | Eva Stewart; dateIdentified: 2021-04-20; identificationRemarks: identified by DNA and morphology; **Event:** eventID: UK1_AB01_EB04; samplingProtocol: Brenke Epibenthic Sledge; eventDate: 2013-10-17; eventTime: 01:50; habitat: Abyssal plain; fieldNotes: Collected from epi net (on the epibenthic sledge); **Record Level:** language: en; institutionCode: NHMUK; collectionCode: ZOO; datasetName: ABYSSLINE; basisOfRecord: PreservedSpecimen

##### Distribution

Eastern Clarion-Clipperton Zone, central Pacific Ocean.

##### Diagnosis

**Figure 9.**
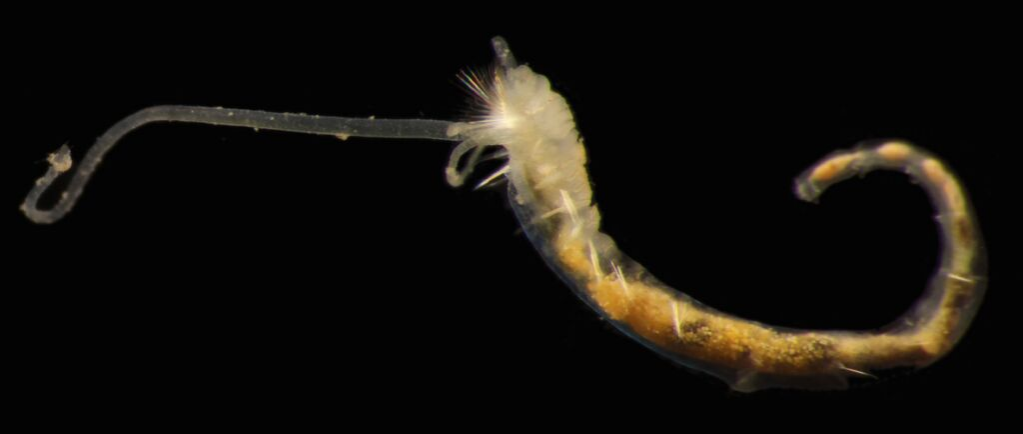
Ampharetidae sp. (NHM_292), live specimen NHM_1865 in lateral view.

Damaged specimens (Fig. 9) consistent with placement within family Ampharetidae, based on morphology and DNA.

#### Ampharetidae sp. (NHM_774)

D5309916-F346-5909-AC36-E6DC1D44F915

##### Materials

- **Type status:** Other material. **Occurrence:** catalogNumber: NHMUK ANEA 2023.330; recordNumber: NHM_0774; recordedBy: Adrian Glover | Helena Wiklund | Thomas Dahlgren | Madeleine Brasier; individualCount: 1; preparations: specimen stored in 80% non-denatured ethanol aqueous solution | DNA voucher stored in buffer; otherCatalogNumbers: 0174126620; associatedSequences: OQ746558 (16S) | OQ746859 (18S); occurrenceID: A2AB0A12-F120-5C81-BF8E-A33B95008AF1; **Taxon:** taxonConceptID: Ampharetidae sp. (NHM_774); scientificName: Ampharetidae; kingdom: Animalia; phylum: Annelida; class: Polychaeta; order: Terebellida; family: Ampharetidae; taxonRank: family; scientificNameAuthorship: Malmgren, 1866; **Location:** waterBody: Pacific; stateProvince: Clarion Clipperton Zone; locality: UK Seabed Resources Ltd exploration area UK-1 Stratum B; verbatimLocality: UK1 Stratum B; maximumDepthInMeters: 4425; locationRemarks: Deployment EB02; at Station U5; from R/V Thomas G. Thompson Cruise no. TN319; verbatimLatitude: 12'32.23; verbatimLongitude: 116'36.25; decimalLatitude: 12.53717; decimalLongitude: -116.60417; geodeticDatum: WGS84; **Identification:** identifiedBy: Helena Wiklund | Lenka Neal | Thomas Dahlgren | Adrian Glover | Madeleine Brasier | Regan Drennan | Eva Stewart; dateIdentified: 2021-04-20; identificationRemarks: identified by DNA and morphology; **Event:** eventID: UK1_AB02_EB02; samplingProtocol: Brenke Epibenthic Sledge; eventDate: 2015-02-20; eventTime: 06:24; habitat: Abyssal plain; fieldNotes: Collected from epi net (on the epibenthic sledge); **Record Level:** language: en; institutionCode: NHMUK; collectionCode: ZOO; datasetName: ABYSSLINE; basisOfRecord: PreservedSpecimen

##### Distribution

Eastern Clarion-Clipperton Zone, central Pacific Ocean.

##### Diagnosis

**Figure 10.**
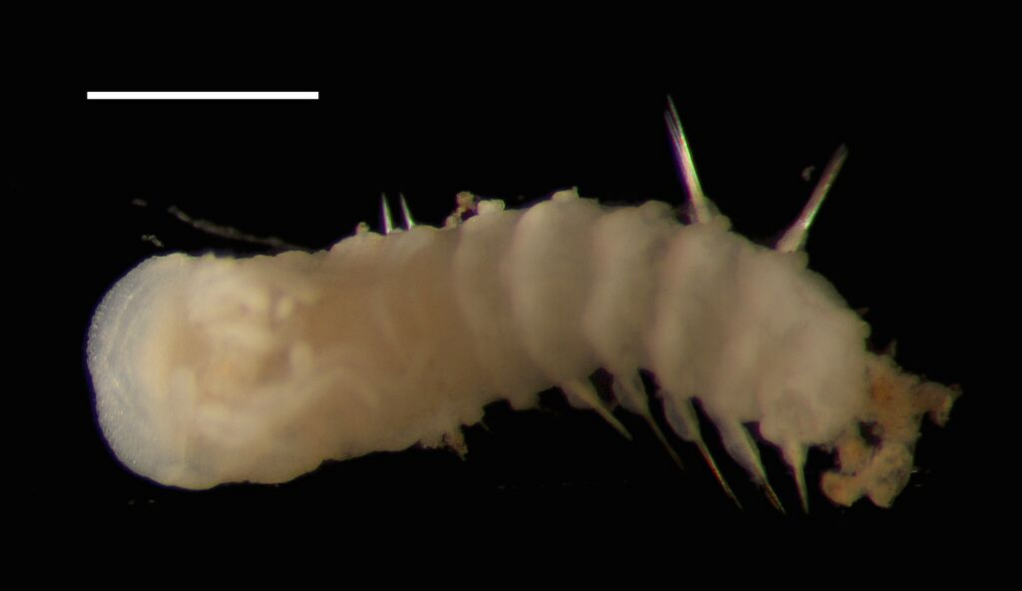
Ampharetidae sp. (NHM_774), posteriorly incomplete specimen NHM_774. Scale bar: 1 mm.

Damaged specimen (Fig. 10) consistent with placement within family Ampharetidae, based on morphology and DNA.

#### Ampharetidae sp. (NHM_1082)

1BB4DDFA-32C9-56C3-A208-8D5DAC1BB8D9

##### Materials

- **Type status:** Other material. **Occurrence:** catalogNumber: NHMUK ANEA 2023.315; recordNumber: NHM_1082; recordedBy: Adrian Glover | Helena Wiklund | Thomas Dahlgren | Madeleine Brasier; individualCount: 1; preparations: specimen stored in 80% non-denatured ethanol aqueous solution | DNA voucher stored in buffer; otherCatalogNumbers: 0174126583; associatedSequences: OQ746614 (16S) | OQ746874 (18S); occurrenceID: D14E9925-C50B-5EB8-808B-0DF8AF255839; **Taxon:** taxonConceptID: Ampharetidae sp. (NHM_1082); scientificName: Ampharetidae; kingdom: Animalia; phylum: Annelida; class: Polychaeta; order: Terebellida; family: Ampharetidae; taxonRank: family; scientificNameAuthorship: Malmgren, 1866; **Location:** waterBody: Pacific; stateProvince: Clarion Clipperton Zone; locality: Ocean Mineral Singapore exploration claim Stratum A; verbatimLocality: OMS Stratum A; maximumDepthInMeters: 4100; locationRemarks: Deployment EB05; at Station S2; from R/V Thomas G. Thompson Cruise no. TN319; verbatimLatitude: 12'06.93; verbatimLongitude: 117'09.87; decimalLatitude: 12.1155; decimalLongitude: -117.1645; geodeticDatum: WGS84; **Identification:** identifiedBy: Helena Wiklund | Lenka Neal | Thomas Dahlgren | Adrian Glover | Madeleine Brasier | Regan Drennan | Eva Stewart; dateIdentified: 2021-04-20; identificationRemarks: identified by DNA and morphology; **Event:** eventID: OMS1_AB02_EB05; samplingProtocol: Brenke Epibenthic Sledge; eventDate: 2015-02-26; eventTime: 21:29; habitat: Abyssal plain; fieldNotes: Collected from epi net (on the epibenthic sledge); **Record Level:** language: en; institutionCode: NHMUK; collectionCode: ZOO; datasetName: ABYSSLINE; basisOfRecord: PreservedSpecimen

##### Distribution

Eastern Clarion-Clipperton Zone, central Pacific Ocean.

##### Diagnosis

**Figure 11.**
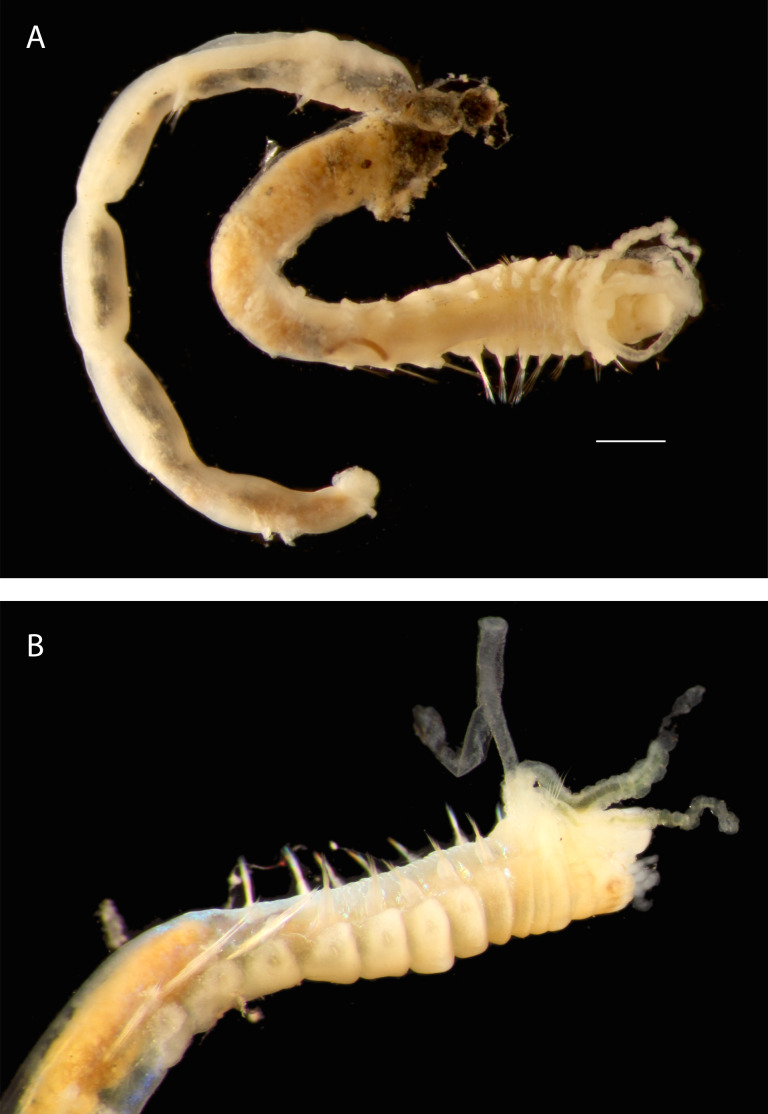
Ampharetidae sp. (NHM_1082). **A** Live specimen NHM_1082 in dorsal view. Scale bar: 1 mm; **B** Thorax and anterior end of specimen NHM_1082 in lateral view.

Damaged specimens (Fig. 11) consistent with placement within family Ampharetidae, based on morphology and DNA.

#### Ampharetidae sp. (NHM_163)

5D41FD6C-786B-542A-9C82-84D0EED7C26D

##### Materials

- **Type status:** Other material. **Occurrence:** catalogNumber: NHMUK ANEA 2023.324; recordNumber: NHM_1025B; recordedBy: Adrian Glover | Helena Wiklund | Thomas Dahlgren | Madeleine Brasier; individualCount: 1; preparations: specimen stored in 80% non-denatured ethanol aqueous solution | DNA voucher stored in buffer; otherCatalogNumbers: 0174126558; associatedSequences: OQ746608 (16S); occurrenceID: 21131AAA-75B7-5229-84A0-C3C8A62E3EEE; **Taxon:** taxonConceptID: Ampharetidae sp. (NHM_163); scientificName: Ampharetidae; kingdom: Animalia; phylum: Annelida; class: Polychaeta; order: Terebellida; family: Ampharetidae; taxonRank: family; scientificNameAuthorship: Malmgren, 1866; **Location:** waterBody: Pacific; stateProvince: Clarion Clipperton Zone; locality: Ocean Mineral Singapore exploration claim Stratum A; verbatimLocality: OMS Stratum A; maximumDepthInMeters: 4122; locationRemarks: Deployment EB04; at Station S1; from R/V Thomas G. Thompson Cruise no. TN319; verbatimLatitude: 12'08.02; verbatimLongitude: 117'17.52; decimalLatitude: 12.13367; decimalLongitude: -117.292; geodeticDatum: WGS84; **Identification:** identifiedBy: Helena Wiklund | Lenka Neal | Thomas Dahlgren | Adrian Glover | Madeleine Brasier | Regan Drennan | Eva Stewart; dateIdentified: 2021-04-20; identificationRemarks: identified by DNA and morphology; **Event:** eventID: OMS1_AB02_EB04; samplingProtocol: Brenke Epibenthic Sledge; eventDate: 2015-02-24; eventTime: 19:10; habitat: Abyssal plain; fieldNotes: Collected from epi net (on the epibenthic sledge); **Record Level:** language: en; institutionCode: NHMUK; collectionCode: ZOO; datasetName: ABYSSLINE; basisOfRecord: PreservedSpecimen
- **Type status:** Other material. **Occurrence:** catalogNumber: NHMUK ANEA 2023.322; recordNumber: NHM_0163; recordedBy: Adrian Glover | Helena Wiklund | Thomas Dahlgren | Magdalena Georgieva; individualCount: 1; preparations: specimen stored in 80% non-denatured ethanol aqueous solution | DNA voucher stored in buffer; otherCatalogNumbers: 0174127365; associatedSequences: OQ746480 (16S) | OQ746802 (18S) | OQ738502 (COI); occurrenceID: EF5227F0-E817-59D1-9FAD-D00A41C502EB; **Taxon:** taxonConceptID: Ampharetidae sp. (NHM_163); scientificName: Ampharetidae; kingdom: Animalia; phylum: Annelida; class: Polychaeta; order: Terebellida; family: Ampharetidae; taxonRank: family; scientificNameAuthorship: Malmgren, 1866; **Location:** waterBody: Pacific; stateProvince: Clarion Clipperton Zone; locality: UK Seabed Resources Ltd exploration area UK-1 Stratum A; verbatimLocality: UK1 Stratum A; maximumDepthInMeters: 4084; locationRemarks: Deployment BC06; at Station D; from R/V Melville Cruise no. MV1313; verbatimLatitude: 13°57.794; verbatimLongitude: 116°34.093; decimalLatitude: 13.96323; decimalLongitude: -116.56822; geodeticDatum: WGS84; **Identification:** identifiedBy: Helena Wiklund | Lenka Neal | Thomas Dahlgren | Adrian Glover | Madeleine Brasier | Regan Drennan | Eva Stewart; dateIdentified: 2021-04-20; identificationRemarks: identified by DNA and morphology; **Event:** eventID: UK1_AB01_BC06; samplingProtocol: USNEL Box Core; eventDate: 2013-10-12; eventTime: 23:01:00; habitat: Abyssal plain; fieldNotes: Collected from 0-2 cm layer of box core using a 300 micron sieve; **Record Level:** language: en; institutionCode: NHMUK; collectionCode: ZOO; datasetName: ABYSSLINE; basisOfRecord: PreservedSpecimen
- **Type status:** Other material. **Occurrence:** catalogNumber: NHMUK ANEA 2023.323; recordNumber: NHM_0334; recordedBy: Adrian Glover | Helena Wiklund | Thomas Dahlgren | Magdalena Georgieva; individualCount: 1; preparations: specimen stored in 80% non-denatured ethanol aqueous solution | DNA voucher stored in buffer; otherCatalogNumbers: 0174127373; associatedSequences: OQ746498 (16S) | OQ746816 (18S); occurrenceID: 3D9F7E8B-9CBD-504B-ADA7-3A5FAFC1523C; **Taxon:** taxonConceptID: Ampharetidae sp. (NHM_163); scientificName: Ampharetidae; kingdom: Animalia; phylum: Annelida; class: Polychaeta; order: Terebellida; family: Ampharetidae; taxonRank: family; scientificNameAuthorship: Malmgren, 1866; **Location:** waterBody: Pacific; stateProvince: Clarion Clipperton Zone; locality: UK Seabed Resources Ltd exploration area UK-1 Stratum A; verbatimLocality: UK1 Stratum A; maximumDepthInMeters: 4075; locationRemarks: Deployment RV05; at Station G; from R/V Melville Cruise no. MV1313; decimalLatitude: 13.76085; decimalLongitude: -116.4653; geodeticDatum: WGS84; **Identification:** identifiedBy: Helena Wiklund | Lenka Neal | Thomas Dahlgren | Adrian Glover | Madeleine Brasier | Regan Drennan | Eva Stewart; dateIdentified: 2021-04-20; identificationRemarks: identified by DNA and morphology; **Event:** eventID: UK1_AB01_RV05; samplingProtocol: Remotely Operated Vehicle; eventDate: 2013-10-17; eventTime: 19:06; habitat: Abyssal plain; **Record Level:** language: en; institutionCode: NHMUK; collectionCode: ZOO; datasetName: ABYSSLINE; basisOfRecord: PreservedSpecimen

##### Distribution

Eastern Clarion-Clipperton Zone, central Pacific Ocean.

##### Diagnosis

**Figure 12.**
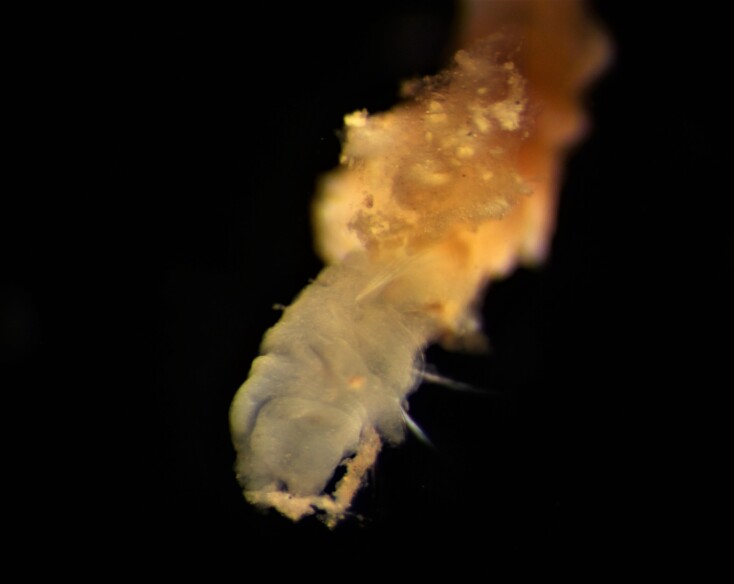
Ampharetidae sp. (NHM_163), anterior fragment of live specimen NHM_1025B, in dorsal view.

Damaged specimens (Fig. 12) consistent with placement within family Ampharetidae, based on morphology and DNA.

#### Ampharetidae sp. (NHM_1599)

81F5F9ED-3659-5118-BD30-0C46F2841FB7

##### Materials

- **Type status:** Other material. **Occurrence:** catalogNumber: NHMUK ANEA 2023.321; recordNumber: NHM_3052; recordedBy: Adrian Glover | Helena Wiklund | Thomas Dahlgren | Madeleine Brasier; individualCount: 1; preparations: specimen stored in 80% non-denatured ethanol aqueous solution | DNA voucher stored in buffer; otherCatalogNumbers: 0174123645; associatedSequences: OQ746787 (16S) | OQ738620 (COI); occurrenceID: BC7C7D83-CC6F-556D-9852-569AECCFDD26; **Taxon:** taxonConceptID: Ampharetidae sp. (NHM_1599); scientificName: Ampharetidae; kingdom: Animalia; phylum: Annelida; class: Polychaeta; order: Terebellida; family: Ampharetidae; taxonRank: family; scientificNameAuthorship: Malmgren, 1866; **Location:** waterBody: Pacific; stateProvince: Clarion Clipperton Zone; locality: Ocean Mineral Singapore exploration claim Stratum A; verbatimLocality: OMS Stratum A; maximumDepthInMeters: 4094; locationRemarks: Deployment EB11; at Station S10; from R/V Thomas G. Thompson Cruise no. TN319; verbatimLatitude: 12°02.49’; verbatimLongitude: 117°13.03’; decimalLatitude: 12.0415; decimalLongitude: -117.21717; geodeticDatum: WGS84; **Identification:** identifiedBy: Helena Wiklund | Lenka Neal | Thomas Dahlgren | Adrian Glover | Madeleine Brasier | Regan Drennan | Eva Stewart; dateIdentified: 2021-04-20; identificationRemarks: identified by DNA and morphology; **Event:** eventID: OMS1_AB02_EB11; samplingProtocol: Brenke Epibenthic Sledge; eventDate: 2015-03-13; habitat: Abyssal plain; fieldNotes: Collected from supra net (on the epibenthic sledge); **Record Level:** language: en; institutionCode: NHMUK; collectionCode: ZOO; datasetName: ABYSSLINE; basisOfRecord: PreservedSpecimen
- **Type status:** Other material. **Occurrence:** catalogNumber: NHMUK ANEA 2023.320; recordNumber: NHM_1599; recordedBy: Adrian Glover | Helena Wiklund | Thomas Dahlgren | Madeleine Brasier; individualCount: 1; preparations: specimen stored in 80% non-denatured ethanol aqueous solution | DNA voucher stored in buffer; otherCatalogNumbers: 0174126145; associatedSequences: OQ746697 (16S) | OQ738594 (COI); occurrenceID: 8CA1389D-CD21-5A98-8548-02A0E366113B; **Taxon:** taxonConceptID: Ampharetidae sp. (NHM_1599); scientificName: Ampharetidae; kingdom: Animalia; phylum: Annelida; class: Polychaeta; order: Terebellida; family: Ampharetidae; taxonRank: family; scientificNameAuthorship: Malmgren, 1866; **Location:** waterBody: Pacific; stateProvince: Clarion Clipperton Zone; locality: UK Seabed Resources Ltd exploration area UK-1 Stratum B; verbatimLocality: UK1 Stratum B; maximumDepthInMeters: 4237; locationRemarks: Deployment BC19; at Station U12; from R/V Thomas G. Thompson Cruise no. TN319; verbatimLatitude: 12'31.273; verbatimLongitude: 116'41.889; decimalLatitude: 12.52122; decimalLongitude: -116.69815; geodeticDatum: WGS84; **Identification:** identifiedBy: Helena Wiklund | Lenka Neal | Thomas Dahlgren | Adrian Glover | Madeleine Brasier | Regan Drennan | Eva Stewart; dateIdentified: 2021-04-20; identificationRemarks: identified by DNA and morphology; **Event:** eventID: UK1_AB02_BC19; samplingProtocol: USNEL Box Core; eventDate: 2015-03-08; eventTime: 01:04; habitat: Abyssal plain; fieldNotes: Collected from 0-2 cm layer of box core using a 300 micron sieve; **Record Level:** language: en; institutionCode: NHMUK; collectionCode: ZOO; datasetName: ABYSSLINE; basisOfRecord: PreservedSpecimen

##### Distribution

Eastern Clarion-Clipperton Zone, central Pacific Ocean.

##### Diagnosis

**Figure 13.**
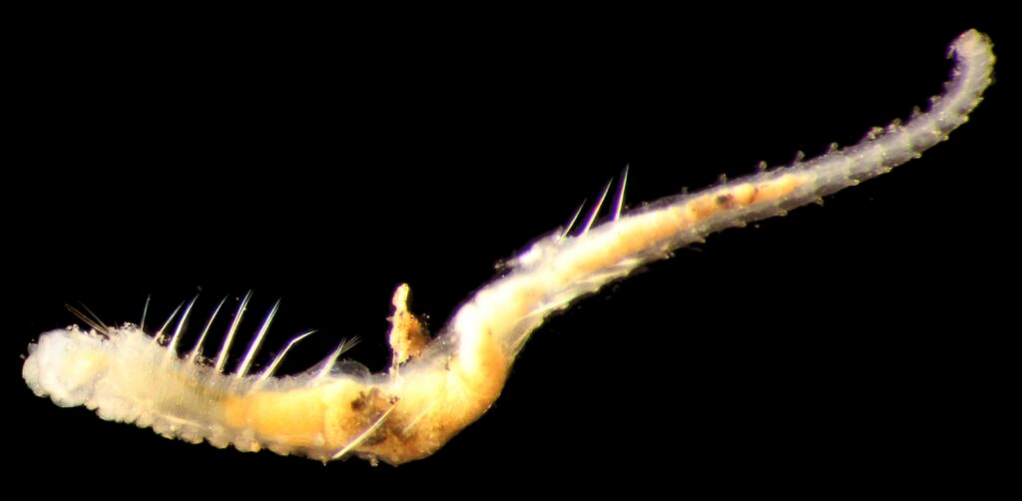
Ampharetidae sp. (NHM_1599), live specimen NHM_1599 in dorsal view.

Damaged specimen (Fig. 13) consistent with placement within family Ampharetidae, based on morphology and DNA.

#### Ampharetidae sp. (NHM_1578)

E63E5087-D4BB-56A3-81C5-1945CA28D3DB

##### Materials

- **Type status:** Other material. **Occurrence:** recordNumber: NHM_1679; recordedBy: Adrian Glover | Helena Wiklund | Thomas Dahlgren | Madeleine Brasier; individualCount: 1; preparations: Tissue voucher stored in 80% non-denatured ethanol aqueous solution | DNA voucher stored in buffer; otherCatalogNumbers: 0109405403 | 0174126146; associatedSequences: OQ746705 (16S) | OQ738596 (COI); occurrenceID: 7AB8D3F6-877D-58CC-B47B-ED903976172F; **Taxon:** taxonConceptID: Ampharetidae sp. (NHM_1578); scientificName: Ampharetidae; kingdom: Animalia; phylum: Annelida; class: Polychaeta; order: Terebellida; family: Ampharetidae; taxonRank: family; scientificNameAuthorship: Malmgren, 1866; **Location:** waterBody: Pacific; stateProvince: Clarion Clipperton Zone; locality: UK Seabed Resources Ltd exploration area UK-1 Stratum B; verbatimLocality: UK1 Stratum B; maximumDepthInMeters: 4233; locationRemarks: Deployment EB09; at Station U1; from R/V Thomas G. Thompson Cruise no. TN319; verbatimLatitude: 12'21.81; verbatimLongitude: 116'40.86; decimalLatitude: 12.3635; decimalLongitude: -116.681; geodeticDatum: WGS84; **Identification:** identifiedBy: Helena Wiklund | Lenka Neal | Thomas Dahlgren | Adrian Glover | Madeleine Brasier | Regan Drennan | Eva Stewart; dateIdentified: 2021-04-20; identificationRemarks: identified by DNA and morphology; **Event:** eventID: UK1_AB02_EB09; samplingProtocol: Brenke Epibenthic Sledge; eventDate: 2015-03-10; eventTime: 10:46; habitat: Abyssal plain; fieldNotes: Collected from epi net (on the epibenthic sledge); **Record Level:** language: en; institutionCode: NHMUK; collectionCode: ZOO; datasetName: ABYSSLINE; basisOfRecord: PreservedSpecimen
- **Type status:** Other material. **Occurrence:** recordNumber: NHM_1578; recordedBy: Adrian Glover | Helena Wiklund | Thomas Dahlgren | Madeleine Brasier; individualCount: 1; preparations: Tissue voucher stored in 80% non-denatured ethanol aqueous solution | DNA voucher stored in buffer; otherCatalogNumbers: 0109405427 | 0174126169; associatedSequences: OQ746695 (16S); occurrenceID: AE0B551A-4B52-5749-83EB-BFEC9AF4DDB2; **Taxon:** taxonConceptID: Ampharetidae sp. (NHM_1578); scientificName: Ampharetidae; kingdom: Animalia; phylum: Annelida; class: Polychaeta; order: Terebellida; family: Ampharetidae; taxonRank: family; scientificNameAuthorship: Malmgren, 1866; **Location:** waterBody: Pacific; stateProvince: Clarion Clipperton Zone; locality: UK Seabed Resources Ltd exploration area UK-1 Stratum B; verbatimLocality: UK1 Stratum B; maximumDepthInMeters: 4136; locationRemarks: Deployment BC18; at Station U12; from R/V Thomas G. Thompson Cruise no. TN319; verbatimLatitude: 12'25.195; verbatimLongitude: 116'37.477; decimalLatitude: 12.41992; decimalLongitude: -116.62462; geodeticDatum: WGS84; **Identification:** identifiedBy: Helena Wiklund | Lenka Neal | Thomas Dahlgren | Adrian Glover | Madeleine Brasier | Regan Drennan | Eva Stewart; dateIdentified: 2021-04-20; identificationRemarks: identified by DNA and morphology; **Event:** eventID: UK1_AB02_BC18; samplingProtocol: USNEL Box Core; eventDate: 2015-03-07; habitat: Abyssal plain; fieldNotes: Collected from nodule in box core sample; **Record Level:** language: en; institutionCode: NHMUK; collectionCode: ZOO; datasetName: ABYSSLINE; basisOfRecord: PreservedSpecimen

##### Distribution

Eastern Clarion-Clipperton Zone, central Pacific Ocean.

##### Diagnosis

**Figure 14.**
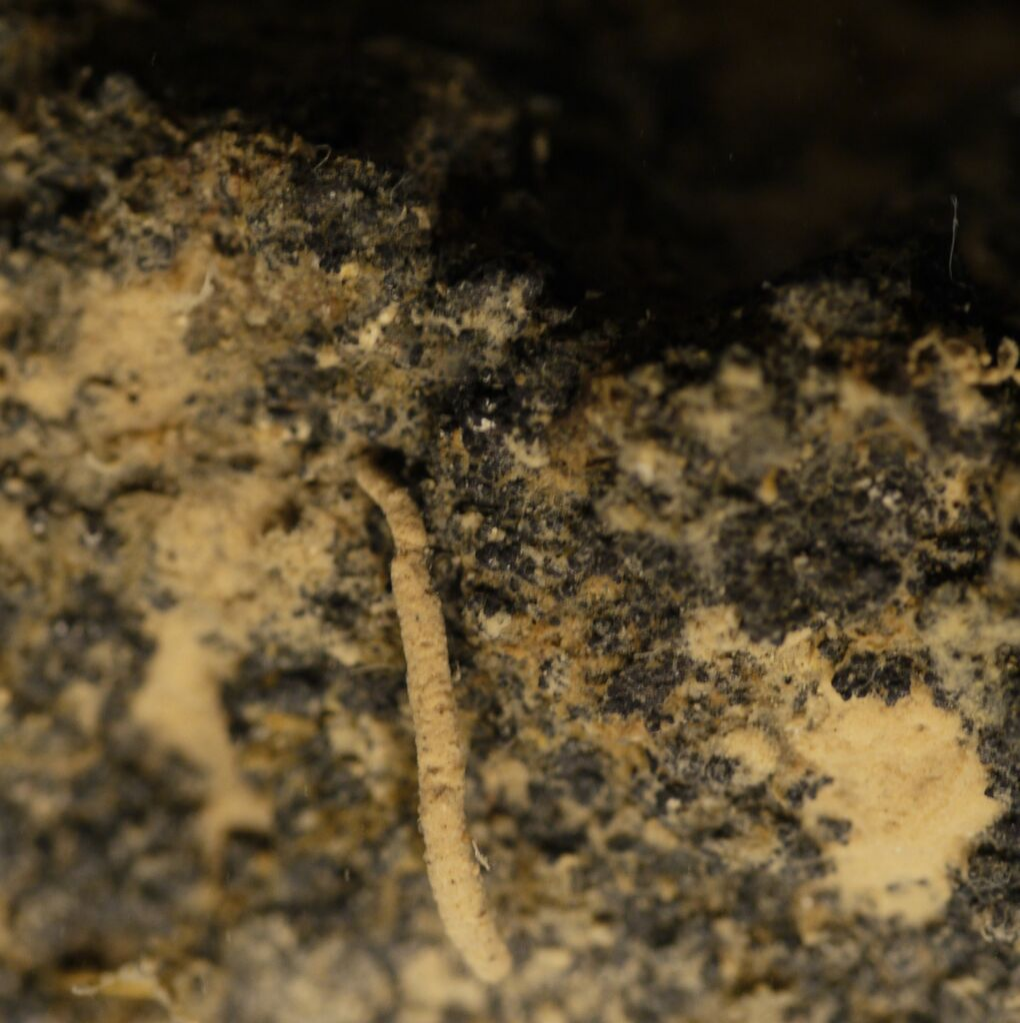
Ampharetidae sp. (NHM_1578), live specimen NHM_1578 inside the muddy tube attached to the nodule.

Damaged specimens consistent with placement within family Ampharetidae, based on morphology and DNA. Live specimens inside the tube found attached to the nodules (Fig. 14).

### Chaetopteridae Audouin and Milne Edwards, 1833

#### Chaetopteridae sp. (NHM_331)

38E31F63-D7D0-520C-B97C-AA6B09E367FE

##### Materials

- **Type status:** Other material. **Occurrence:** catalogNumber: NHMUK ANEA 2023.336; recordNumber: NHM_1163B; recordedBy: Adrian Glover | Helena Wiklund | Thomas Dahlgren | Madeleine Brasier; individualCount: 1; preparations: specimen stored in 80% non-denatured ethanol aqueous solution | DNA voucher stored in buffer; otherCatalogNumbers: 0174126615; associatedSequences: OQ738564 (COI); occurrenceID: 33907DBF-3CBE-5938-AFF9-D89E722F9420; **Taxon:** taxonConceptID: Chaetopteridae sp. (NHM_331); scientificName: Chaetopteridae; kingdom: Animalia; phylum: Annelida; class: Polychaeta; family: Chaetopteridae; taxonRank: family; scientificNameAuthorship: Audouin & Milne Edwards, 1833; **Location:** waterBody: Pacific; stateProvince: Clarion Clipperton Zone; locality: Ocean Mineral Singapore exploration claim Stratum A; verbatimLocality: OMS Stratum A; maximumDepthInMeters: 4100; locationRemarks: Deployment EB05; at Station S2; from R/V Thomas G. Thompson Cruise no. TN319; verbatimLatitude: 12'06.93; verbatimLongitude: 117'09.87; decimalLatitude: 12.1155; decimalLongitude: -117.1645; geodeticDatum: WGS84; **Identification:** identifiedBy: Helena Wiklund | Lenka Neal | Thomas Dahlgren | Adrian Glover | Madeleine Brasier | Regan Drennan | Eva Stewart; dateIdentified: 2021-04-20; identificationRemarks: identified by DNA and morphology; **Event:** eventID: OMS1_AB02_EB05; samplingProtocol: Brenke Epibenthic Sledge; eventDate: 2015-02-26; eventTime: 21:29; habitat: Abyssal plain; fieldNotes: Collected from epi net (on the epibenthic sledge); **Record Level:** language: en; institutionCode: NHMUK; collectionCode: ZOO; datasetName: ABYSSLINE; basisOfRecord: PreservedSpecimen
- **Type status:** Other material. **Occurrence:** catalogNumber: NHMUK ANEA 2023.335; recordNumber: NHM_0461; recordedBy: Adrian Glover | Helena Wiklund | Thomas Dahlgren | Magdalena Georgieva; individualCount: 1; preparations: specimen stored in 80% non-denatured ethanol aqueous solution | DNA voucher stored in buffer; otherCatalogNumbers: 0174127341; associatedSequences: OQ746523 (16S) | OQ746839 (18S) | OQ738523 (COI); occurrenceID: 3E51E30E-453C-5372-A61F-04D09B8071BD; **Taxon:** taxonConceptID: Chaetopteridae sp. (NHM_331); scientificName: Chaetopteridae; kingdom: Animalia; phylum: Annelida; class: Polychaeta; family: Chaetopteridae; taxonRank: family; scientificNameAuthorship: Audouin & Milne Edwards, 1833; **Location:** waterBody: Pacific; stateProvince: Clarion Clipperton Zone; locality: UK Seabed Resources Ltd exploration area UK-1 Stratum A; verbatimLocality: UK1 Stratum A; maximumDepthInMeters: 4160; locationRemarks: Deployment BC14; at Station L; from R/V Melville Cruise no. MV1313; verbatimLatitude: 13°43.597; verbatimLongitude: 116°40.200; decimalLatitude: 13.72662; decimalLongitude: -116.67; geodeticDatum: WGS84; **Identification:** identifiedBy: Helena Wiklund | Lenka Neal | Thomas Dahlgren | Adrian Glover | Madeleine Brasier | Regan Drennan | Eva Stewart; dateIdentified: 2021-04-20; identificationRemarks: identified by DNA and morphology; **Event:** eventID: UK1_AB01_BC14; samplingProtocol: USNEL Box Core; eventDate: 2013-10-22; eventTime: 07:25; habitat: Abyssal plain; fieldNotes: Collected from 0-2 cm layer of box core using a 300 micron sieve; **Record Level:** language: en; institutionCode: NHMUK; collectionCode: ZOO; datasetName: ABYSSLINE; basisOfRecord: PreservedSpecimen
- **Type status:** Other material. **Occurrence:** catalogNumber: NHMUK ANEA 2023.334; recordNumber: NHM_0331; recordedBy: Adrian Glover | Helena Wiklund | Thomas Dahlgren | Magdalena Georgieva; individualCount: 1; preparations: specimen stored in 80% non-denatured ethanol aqueous solution | DNA voucher stored in buffer; otherCatalogNumbers: 0174127338; associatedSequences: OQ746496 (16S) | OQ746815 (18S); occurrenceID: D3FE16FA-F760-5239-8DB1-BA9216776507; **Taxon:** taxonConceptID: Chaetopteridae sp. (NHM_331); scientificName: Chaetopteridae; kingdom: Animalia; phylum: Annelida; class: Polychaeta; family: Chaetopteridae; taxonRank: family; scientificNameAuthorship: Audouin & Milne Edwards, 1833; **Location:** waterBody: Pacific; stateProvince: Clarion Clipperton Zone; locality: UK Seabed Resources Ltd exploration area UK-1 Stratum A; verbatimLocality: UK1 Stratum A; maximumDepthInMeters: 4075; locationRemarks: Deployment RV05; at Station G; from R/V Melville Cruise no. MV1313; decimalLatitude: 13.76085; decimalLongitude: -116.4653; geodeticDatum: WGS84; **Identification:** identifiedBy: Helena Wiklund | Lenka Neal | Thomas Dahlgren | Adrian Glover | Madeleine Brasier | Regan Drennan | Eva Stewart; dateIdentified: 2021-04-20; identificationRemarks: identified by DNA and morphology; **Event:** eventID: UK1_AB01_RV05; samplingProtocol: Remotely Operated Vehicle; eventDate: 2013-10-17; eventTime: 19:06; habitat: Abyssal plain; **Record Level:** language: en; institutionCode: NHMUK; collectionCode: ZOO; datasetName: ABYSSLINE; basisOfRecord: PreservedSpecimen

##### Distribution

Eastern Clarion-Clipperton Zone, central Pacific Ocean.

##### Diagnosis

**Figure 15.**
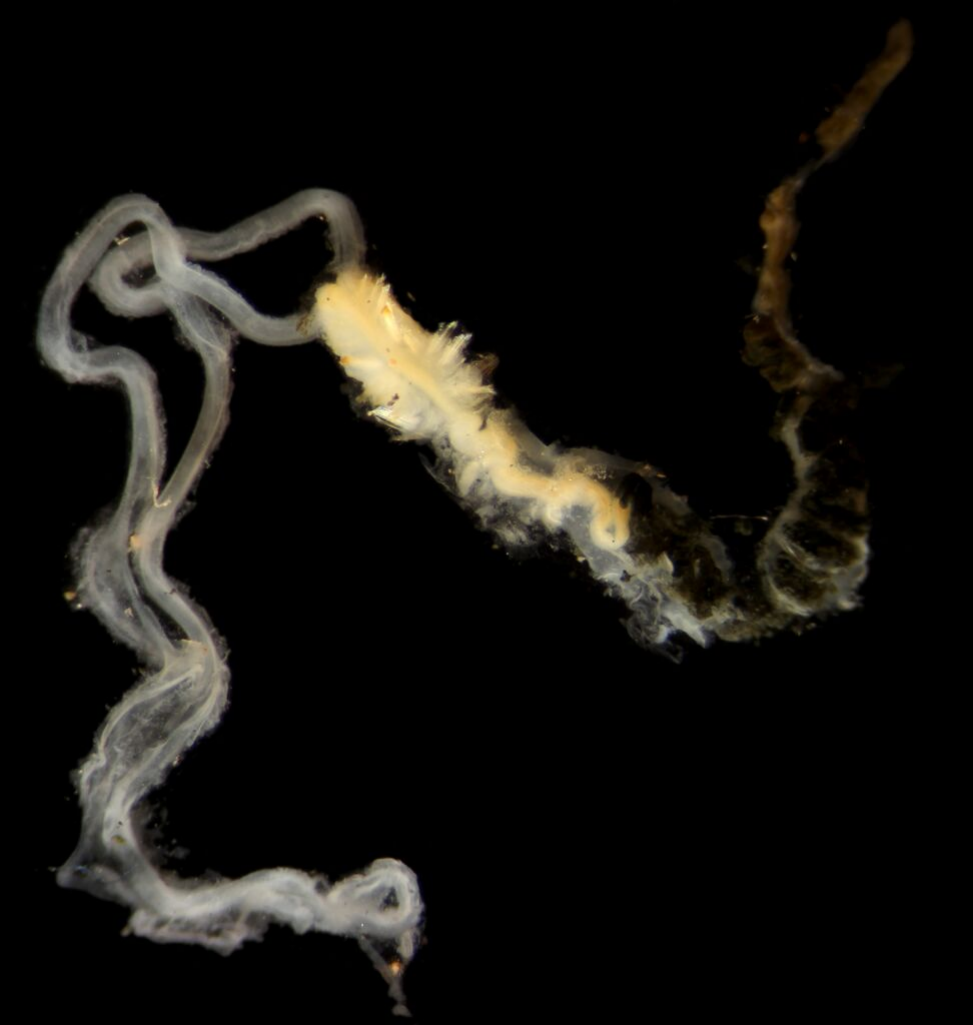
Chaetopteridae sp. (NHM_331), live specimen NHM_461 in dorsal view.

Damaged specimens (Fig. 15) consistent with placement within family Chaetopteridae, based on morphology and DNA.

### Chrysopetalidae Ehlers, 1864

#### Chrysopetalidae sp. (NHM_2026)

5C7DCF09-B5BB-500B-A07D-FF5655A2ECFD

##### Materials

- **Type status:** Other material. **Occurrence:** recordNumber: NHM_2026; recordedBy: Adrian Glover | Helena Wiklund | Thomas Dahlgren | Madeleine Brasier; individualCount: 2; preparations: Tissue voucher stored in 80% non-denatured ethanol aqueous solution | DNA voucher stored in buffer; otherCatalogNumbers: 0174127391 | 0174123646; associatedSequences: OQ746747 (16S); occurrenceID: 6CB2964D-1B80-5F26-ABCD-332F57EACAD9; **Taxon:** taxonConceptID: Chrysopetalidae sp. (NHM_2026); scientificName: Chrysopetalidae; kingdom: Animalia; phylum: Annelida; class: Polychaeta; order: Phyllodocida; family: Chrysopetalidae; taxonRank: family; scientificNameAuthorship: Ehlers, 1864; **Location:** waterBody: Pacific; stateProvince: Clarion Clipperton Zone; locality: Ocean Mineral Singapore exploration claim Stratum A; verbatimLocality: OMS Stratum A; maximumDepthInMeters: 4235; locationRemarks: Deployment EB12; at Station S11; from R/V Thomas G. Thompson Cruise no. TN319; verbatimLatitude: 12'03.03; verbatimLongitude: 117'24.28; decimalLatitude: 12.0505; decimalLongitude: -117.40467; geodeticDatum: WGS84; **Identification:** identifiedBy: Helena Wiklund | Lenka Neal | Thomas Dahlgren | Adrian Glover | Madeleine Brasier | Regan Drennan | Eva Stewart; dateIdentified: 2021-04-20; identificationRemarks: identified by DNA and morphology; **Event:** eventID: OMS1_AB02_EB12; samplingProtocol: Brenke Epibenthic Sledge; eventDate: 2015-03-16; eventTime: 05:30; habitat: Abyssal plain; fieldNotes: Collected from epi net (on the epibenthic sledge); **Record Level:** language: en; institutionCode: NHMUK; collectionCode: ZOO; datasetName: ABYSSLINE; basisOfRecord: PreservedSpecimen

##### Distribution

Eastern Clarion-Clipperton Zone, central Pacific Ocean.

##### Diagnosis

Damaged specimen consistent with placement within family Chrysopetalidae Ehlers, 1864 based on morphology and DNA.

#### Chrysopetalidae sp. (NHM_410)

122D27C7-44FB-5394-AF11-B25CA4C46958

##### Materials

- **Type status:** Other material. **Occurrence:** recordNumber: NHM_0410; recordedBy: Adrian Glover | Helena Wiklund | Thomas Dahlgren | Magdalena Georgieva; individualCount: 1; preparations: Tissue voucher stored in 80% non-denatured ethanol aqueous solution | DNA voucher stored in buffer; otherCatalogNumbers: 0109405360 | 0174127297; associatedSequences: OQ746510 (16S) | OQ738518 (COI); occurrenceID: A1F1FF52-ABD9-58B5-B30D-AEDC4CF8C359; **Taxon:** taxonConceptID: Chrysopetalidae sp. (NHM_410); scientificName: Chrysopetalidae; kingdom: Animalia; phylum: Annelida; class: Polychaeta; order: Phyllodocida; family: Chrysopetalidae; taxonRank: family; scientificNameAuthorship: Ehlers, 1864; **Location:** waterBody: Pacific; stateProvince: Clarion Clipperton Zone; locality: UK Seabed Resources Ltd exploration area UK-1 Stratum A; verbatimLocality: UK1 Stratum A; maximumDepthInMeters: 4500; locationRemarks: Deployment BC12; at Station K; from R/V Melville Cruise no. MV1313; decimalLatitude: 13.86328; decimalLongitude: -116.54885; geodeticDatum: WGS84; **Identification:** identifiedBy: Helena Wiklund | Lenka Neal | Thomas Dahlgren | Adrian Glover | Madeleine Brasier | Regan Drennan | Eva Stewart; dateIdentified: 2021-04-20; identificationRemarks: identified by DNA and morphology; **Event:** eventID: UK1_AB01_BC12; samplingProtocol: USNEL Box Core; eventDate: 2013-10-20; eventTime: 03:39; habitat: Abyssal plain; fieldNotes: Collected from 0-2 cm layer of box core using a 300 micron sieve; **Record Level:** language: en; institutionCode: NHMUK; collectionCode: ZOO; datasetName: ABYSSLINE; basisOfRecord: PreservedSpecimen

##### Distribution

Eastern Clarion-Clipperton Zone, central Pacific Ocean.

##### Diagnosis

**Figure 16.**
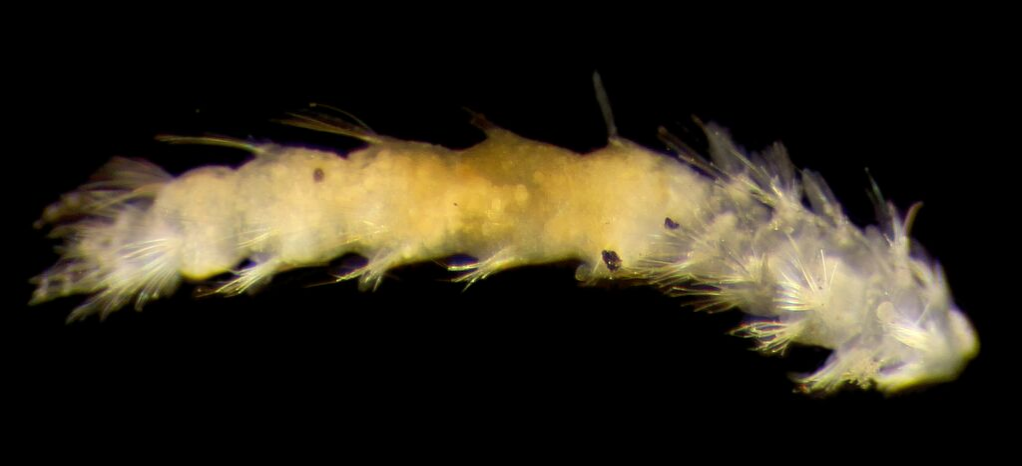
Chrysopetalidae sp. (NHM_410), anterior end of live specimen NHM_410 in dorsal view.

Damaged specimen (Fig. 16) consistent with placement within family Chrysopetalidae Ehlers, 1864, based on morphology and DNA.

#### Chrysopetalidae sp. (NHM_1550)

5277B5EC-46F4-5AF5-A29C-E453E4E29BDA

##### Materials

- **Type status:** Other material. **Occurrence:** recordNumber: NHM_1550; recordedBy: Adrian Glover | Helena Wiklund | Thomas Dahlgren | Madeleine Brasier; individualCount: 1; preparations: Tissue voucher stored in 80% non-denatured ethanol aqueous solution | DNA voucher stored in buffer; otherCatalogNumbers: 0109405058 | 0174126190; associatedSequences: OQ746694 (16S); occurrenceID: 6F7DBD3E-1EEF-597F-B530-C7C2771A9BB0; **Taxon:** taxonConceptID: Chrysopetalidae sp. (NHM_1550); scientificName: Chrysopetalidae; kingdom: Animalia; phylum: Annelida; class: Polychaeta; order: Phyllodocida; family: Chrysopetalidae; taxonRank: family; scientificNameAuthorship: Ehlers, 1864; **Location:** waterBody: Pacific; stateProvince: Clarion Clipperton Zone; locality: UK Seabed Resources Ltd exploration area UK-1 Stratum B; verbatimLocality: UK1 Stratum B; maximumDepthInMeters: 4226; locationRemarks: Deployment MC15; at Station U10; from R/V Thomas G. Thompson Cruise no. TN319; verbatimLatitude: 12'34.188; verbatimLongitude: 116'32.331; decimalLatitude: 12.5698; decimalLongitude: -116.53885; geodeticDatum: WGS84; **Identification:** identifiedBy: Helena Wiklund | Lenka Neal | Thomas Dahlgren | Adrian Glover | Madeleine Brasier | Regan Drennan | Eva Stewart; dateIdentified: 2021-04-20; identificationRemarks: identified by DNA and morphology; **Event:** eventID: UK1_AB02_MC15; samplingProtocol: Multi Corer; eventDate: 2015-03-06; eventTime: 06:40; habitat: Abyssal plain; **Record Level:** language: en; institutionCode: NHMUK; collectionCode: ZOO; datasetName: ABYSSLINE; basisOfRecord: PreservedSpecimen

##### Distribution

Eastern Clarion-Clipperton Zone, central Pacific Ocean.

##### Diagnosis

**Figure 17.**
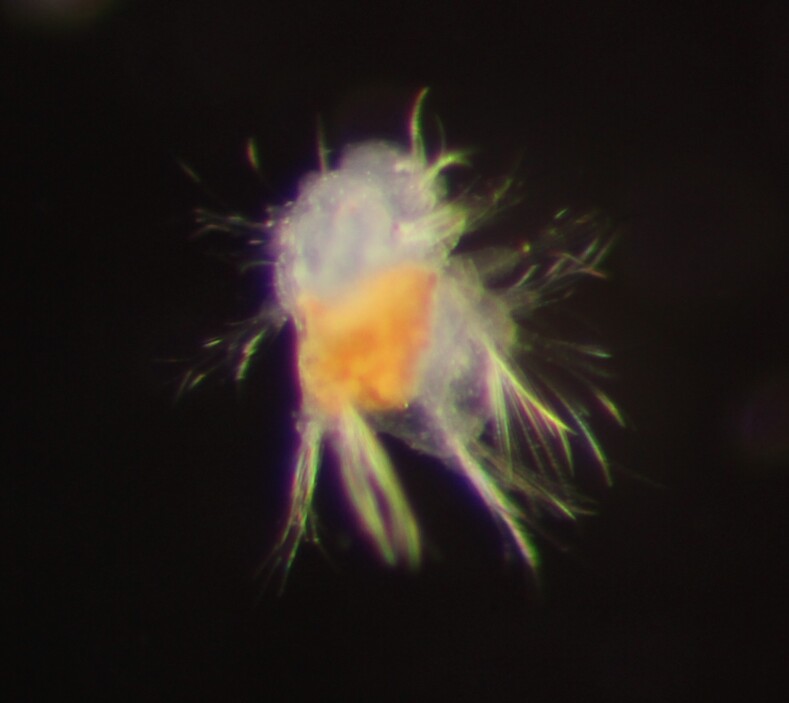
Chrysopetalidae sp. (NHM_1550), body fragment of live specimen NHM_1550.

Damaged specimen (Fig. 17) consistent with placement within family Chrysopetalidae, based on morphology and DNA.

#### Chrysopetalidae sp. NHM (1303)

A4B39A76-19F1-5407-9A28-8329D882C2FF

##### Materials

- **Type status:** Other material. **Occurrence:** catalogNumber: NHMUK ANEA 2023.338; recordNumber: NHM_1303; recordedBy: Adrian Glover | Helena Wiklund | Thomas Dahlgren | Madeleine Brasier; individualCount: 1; preparations: specimen stored in 80% non-denatured ethanol aqueous solution | DNA voucher stored in buffer; otherCatalogNumbers: 0174126610; associatedSequences: OQ746654 (16S); occurrenceID: 27C6DC8E-377E-551A-8913-9B45466E4A56; **Taxon:** taxonConceptID: Chrysopetalidae NHM (1303); scientificName: Chrysopetalidae; kingdom: Animalia; phylum: Annelida; class: Polychaeta; order: Phyllodocida; family: Chrysopetalidae; taxonRank: family; scientificNameAuthorship: Ehlers, 1864; **Location:** waterBody: Pacific; stateProvince: Clarion Clipperton Zone; locality: Ocean Mineral Singapore exploration claim Stratum A; verbatimLocality: OMS Stratum A; maximumDepthInMeters: 4302; locationRemarks: Deployment EB06; at Station S5; from R/V Thomas G. Thompson Cruise no. TN319; verbatimLatitude: 12'15.44; verbatimLongitude: 117'18.13; decimalLatitude: 12.25733; decimalLongitude: -117.30217; geodeticDatum: WGS84; **Identification:** identifiedBy: Helena Wiklund | Lenka Neal | Thomas Dahlgren | Adrian Glover | Madeleine Brasier | Regan Drennan | Eva Stewart; dateIdentified: 2021-04-20; identificationRemarks: identified by DNA and morphology; **Event:** eventID: OMS1_AB02_EB06; samplingProtocol: Brenke Epibenthic Sledge; eventDate: 2015-03-01; eventTime: 04:02; habitat: Abyssal plain; fieldNotes: Collected from epi net (on the epibenthic sledge); **Record Level:** language: en; institutionCode: NHMUK; collectionCode: ZOO; datasetName: ABYSSLINE; basisOfRecord: PreservedSpecimen
- **Type status:** Other material. **Occurrence:** recordNumber: NHM_1349; recordedBy: Adrian Glover | Helena Wiklund | Thomas Dahlgren | Madeleine Brasier; individualCount: 1; preparations: Tissue voucher stored in 80% non-denatured ethanol aqueous solution | DNA voucher stored in buffer; otherCatalogNumbers: 0109405357 | 0174126564; associatedSequences: OQ746671 (16S); occurrenceID: F2F25CEF-4B63-50BF-A2D1-D4AC898A0151; **Taxon:** taxonConceptID: Chrysopetalidae NHM (1303); scientificName: Chrysopetalidae; kingdom: Animalia; phylum: Annelida; class: Polychaeta; order: Phyllodocida; family: Chrysopetalidae; taxonRank: family; scientificNameAuthorship: Ehlers, 1864; **Location:** waterBody: Pacific; stateProvince: Clarion Clipperton Zone; locality: Ocean Mineral Singapore exploration claim Stratum A; verbatimLocality: OMS Stratum A; maximumDepthInMeters: 4302; locationRemarks: Deployment EB06; at Station S5; from R/V Thomas G. Thompson Cruise no. TN319; verbatimLatitude: 12'15.44; verbatimLongitude: 117'18.13; decimalLatitude: 12.25733; decimalLongitude: -117.30217; geodeticDatum: WGS84; **Identification:** identifiedBy: Helena Wiklund | Lenka Neal | Thomas Dahlgren | Adrian Glover | Madeleine Brasier | Regan Drennan | Eva Stewart; dateIdentified: 2021-04-20; identificationRemarks: identified by DNA and morphology; **Event:** eventID: OMS1_AB02_EB06; samplingProtocol: Brenke Epibenthic Sledge; eventDate: 2015-03-01; eventTime: 04:02; habitat: Abyssal plain; fieldNotes: Collected from epi net (on the epibenthic sledge); **Record Level:** language: en; institutionCode: NHMUK; collectionCode: ZOO; datasetName: ABYSSLINE; basisOfRecord: PreservedSpecimen

##### Distribution

Eastern Clarion-Clipperton Zone, central Pacific Ocean.

##### Diagnosis

Damaged specimens (Fig. 18) consistent with placement within family Chrysopetalidae, based on morphology and DNA.

#### Chrysopetalidae sp. (NHM_748A)

45FF7F13-0501-5422-9686-9984DF008CA4

##### Materials

- **Type status:** Other material. **Occurrence:** catalogNumber: NHMUK ANEA 2023.337; recordNumber: NHM_0748A; recordedBy: Adrian Glover | Helena Wiklund | Thomas Dahlgren | Madeleine Brasier; individualCount: 1; preparations: specimen stored in 80% non-denatured ethanol aqueous solution | DNA voucher stored in buffer; otherCatalogNumbers: 0174126549; associatedSequences: OQ746550 (16S) | OQ746856 (18S); occurrenceID: 51A6FF98-3DC7-5009-94F0-DFF69B9F859E; **Taxon:** taxonConceptID: Chrysopetalidae sp. (NHM_748A); scientificName: Chrysopetalidae; kingdom: Animalia; phylum: Annelida; class: Polychaeta; order: Phyllodocida; family: Chrysopetalidae; taxonRank: family; scientificNameAuthorship: Ehlers, 1864; **Location:** waterBody: Pacific; stateProvince: Clarion Clipperton Zone; locality: UK Seabed Resources Ltd exploration area UK-1 Stratum B; verbatimLocality: UK1 Stratum B; maximumDepthInMeters: 4425; locationRemarks: Deployment EB02; at Station U5; from R/V Thomas G. Thompson Cruise no. TN319; verbatimLatitude: 12'32.23; verbatimLongitude: 116'36.25; decimalLatitude: 12.53717; decimalLongitude: -116.60417; geodeticDatum: WGS84; **Identification:** identifiedBy: Helena Wiklund | Lenka Neal | Thomas Dahlgren | Adrian Glover | Madeleine Brasier | Regan Drennan | Eva Stewart; dateIdentified: 2021-04-20; identificationRemarks: identified by DNA and morphology; **Event:** eventID: UK1_AB02_EB02; samplingProtocol: Brenke Epibenthic Sledge; eventDate: 2015-02-20; eventTime: 06:24; habitat: Abyssal plain; fieldNotes: Collected from epi net (on the epibenthic sledge); **Record Level:** language: en; institutionCode: NHMUK; collectionCode: ZOO; datasetName: ABYSSLINE; basisOfRecord: PreservedSpecimen

##### Distribution

Eastern Clarion-Clipperton Zone, central Pacific Ocean.

##### Diagnosis

**Figure 19.**
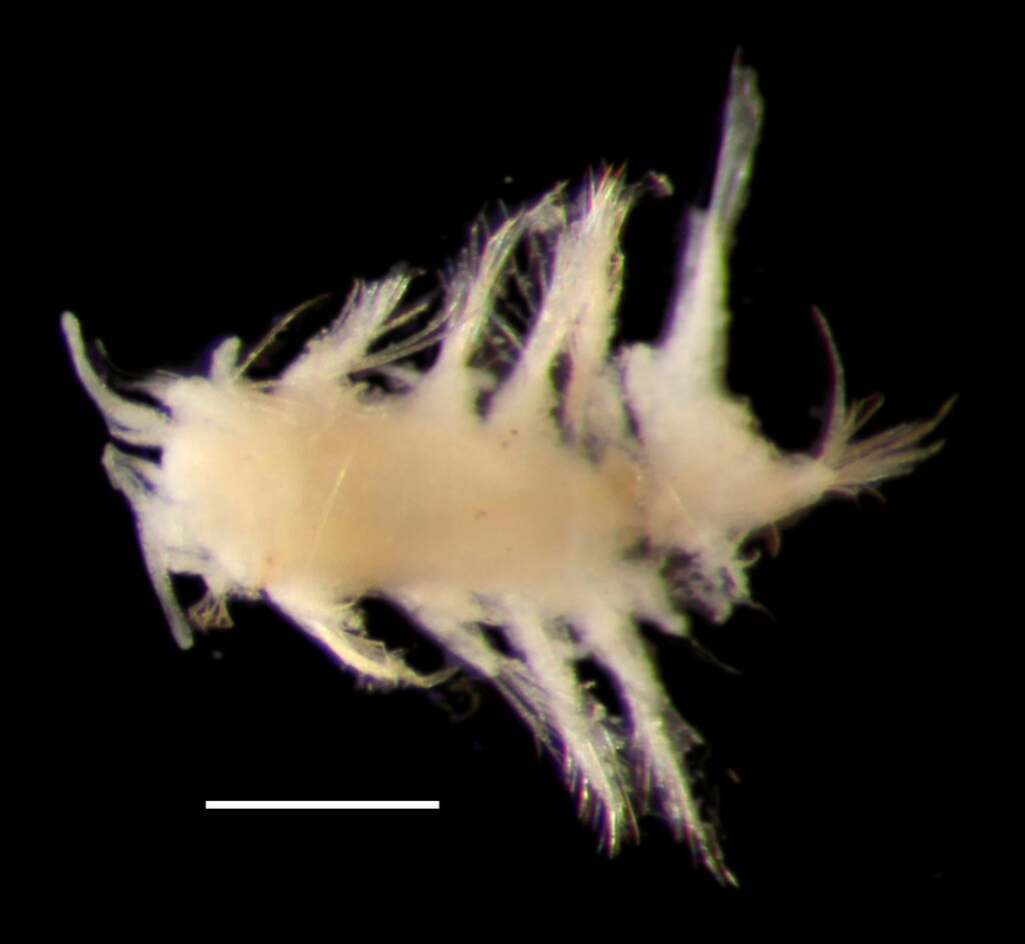
Chrysopetalidae sp. (NHM_748A), anterior fragment of live specimen NHM_748A. Scale bar: 500 µm.

Damaged specimen (Fig. 19) consistent with placement within family Chrysopetalidae, based on morphology and DNA.

### Cirratulidae Ryckholt, 1851

#### Cirratulidae sp. (NHM_530)

F58F89B6-C2A4-57C3-BF66-47DD389C1228

##### Materials

- **Type status:** Other material. **Occurrence:** catalogNumber: NHMUK ANEA 2023.362; recordNumber: NHM_1268; recordedBy: Adrian Glover | Helena Wiklund | Thomas Dahlgren | Madeleine Brasier; individualCount: 1; preparations: specimen stored in 80% non-denatured ethanol aqueous solution | DNA voucher stored in buffer; otherCatalogNumbers: 0174126537; associatedSequences: OQ746648 (16S) | OQ738575 (COI); occurrenceID: 62CED7EC-7D44-53E5-856F-BDC4B84AA420; **Taxon:** taxonConceptID: Cirratulidae sp. (NHM_530); scientificName: Cirratulidae; kingdom: Animalia; phylum: Annelida; class: Polychaeta; order: Terebellida; family: Cirratulidae; taxonRank: family; scientificNameAuthorship: Ryckholt, 1851; **Location:** waterBody: Pacific; stateProvince: Clarion Clipperton Zone; locality: Ocean Mineral Singapore exploration claim Stratum A; verbatimLocality: OMS Stratum A; maximumDepthInMeters: 4302; locationRemarks: Deployment EB06; at Station S5; from R/V Thomas G. Thompson Cruise no. TN319; verbatimLatitude: 12'15.44; verbatimLongitude: 117'18.13; decimalLatitude: 12.25733; decimalLongitude: -117.30217; geodeticDatum: WGS84; **Identification:** identifiedBy: Helena Wiklund | Lenka Neal | Thomas Dahlgren | Adrian Glover | Madeleine Brasier | Regan Drennan | Eva Stewart; dateIdentified: 2021-04-20; identificationRemarks: identified by DNA and morphology; **Event:** eventID: OMS1_AB02_EB06; samplingProtocol: Brenke Epibenthic Sledge; eventDate: 2015-03-01; eventTime: 04:02; habitat: Abyssal plain; fieldNotes: Collected from epi net (on the epibenthic sledge); **Record Level:** language: en; institutionCode: NHMUK; collectionCode: ZOO; datasetName: ABYSSLINE; basisOfRecord: PreservedSpecimen
- **Type status:** Other material. **Occurrence:** recordNumber: NHM_1348H; recordedBy: Adrian Glover | Helena Wiklund | Thomas Dahlgren | Madeleine Brasier; individualCount: 1; preparations: Tissue voucher stored in 80% non-denatured ethanol aqueous solution | DNA voucher stored in buffer; otherCatalogNumbers: 0109405378 | 0174126588; associatedSequences: OQ746669 (16S) | OQ738579 (COI); occurrenceID: 643009D7-D068-5941-8848-13A7BB7FC6FD; **Taxon:** taxonConceptID: Cirratulidae sp. (NHM_530); scientificName: Cirratulidae; kingdom: Animalia; phylum: Annelida; class: Polychaeta; order: Terebellida; family: Cirratulidae; taxonRank: family; scientificNameAuthorship: Ryckholt, 1851; **Location:** waterBody: Pacific; stateProvince: Clarion Clipperton Zone; locality: Ocean Mineral Singapore exploration claim Stratum A; verbatimLocality: OMS Stratum A; maximumDepthInMeters: 4302; locationRemarks: Deployment EB06; at Station S5; from R/V Thomas G. Thompson Cruise no. TN319; verbatimLatitude: 12'15.44; verbatimLongitude: 117'18.13; decimalLatitude: 12.25733; decimalLongitude: -117.30217; geodeticDatum: WGS84; **Identification:** identifiedBy: Helena Wiklund | Lenka Neal | Thomas Dahlgren | Adrian Glover | Madeleine Brasier | Regan Drennan | Eva Stewart; dateIdentified: 2021-04-20; identificationRemarks: identified by DNA and morphology; **Event:** eventID: OMS1_AB02_EB06; samplingProtocol: Brenke Epibenthic Sledge; eventDate: 2015-03-01; eventTime: 04:02; habitat: Abyssal plain; fieldNotes: Collected from epi net (on the epibenthic sledge); **Record Level:** language: en; institutionCode: NHMUK; collectionCode: ZOO; datasetName: ABYSSLINE; basisOfRecord: PreservedSpecimen
- **Type status:** Other material. **Occurrence:** catalogNumber: NHMUK ANEA 2023.361; recordNumber: NHM_0530; recordedBy: Adrian Glover | Helena Wiklund | Thomas Dahlgren | Madeleine Brasier; individualCount: 1; preparations: specimen stored in 80% non-denatured ethanol aqueous solution | DNA voucher stored in buffer; otherCatalogNumbers: 0174126731; associatedSequences: OQ746525 (16S) | OQ746842 (18S) | OQ738524 (COI); occurrenceID: D779D49E-D225-5B2F-8BC6-E72B98AE12A1; **Taxon:** taxonConceptID: Cirratulidae sp. (NHM_530); scientificName: Cirratulidae; kingdom: Animalia; phylum: Annelida; class: Polychaeta; order: Terebellida; family: Cirratulidae; taxonRank: family; scientificNameAuthorship: Ryckholt, 1851; **Location:** waterBody: Pacific; stateProvince: Clarion Clipperton Zone; locality: UK Seabed Resources Ltd exploration area UK-1 Stratum B; verbatimLocality: UK1 Stratum B; maximumDepthInMeters: 4158; locationRemarks: Deployment BC02; at Station U2; from R/V Thomas G. Thompson Cruise no. TN319; verbatimLatitude: 12'22.020; verbatimLongitude: 116'31.017; decimalLatitude: 12.367; decimalLongitude: -116.51695; geodeticDatum: WGS84; **Identification:** identifiedBy: Helena Wiklund | Lenka Neal | Thomas Dahlgren | Adrian Glover | Madeleine Brasier | Regan Drennan | Eva Stewart; dateIdentified: 2021-04-20; identificationRemarks: identified by DNA and morphology; **Event:** eventID: UK1_AB02_BC02; samplingProtocol: USNEL Box Core; eventDate: 2015-02-17; eventTime: 12:12; habitat: Abyssal plain; fieldNotes: Collected from 0-2 cm layer of box core using a 300 micron sieve; **Record Level:** language: en; institutionCode: NHMUK; collectionCode: ZOO; datasetName: ABYSSLINE; basisOfRecord: PreservedSpecimen

##### Distribution

Eastern Clarion-Clipperton Zone, central Pacific Ocean.

##### Diagnosis

**Figure 20.**
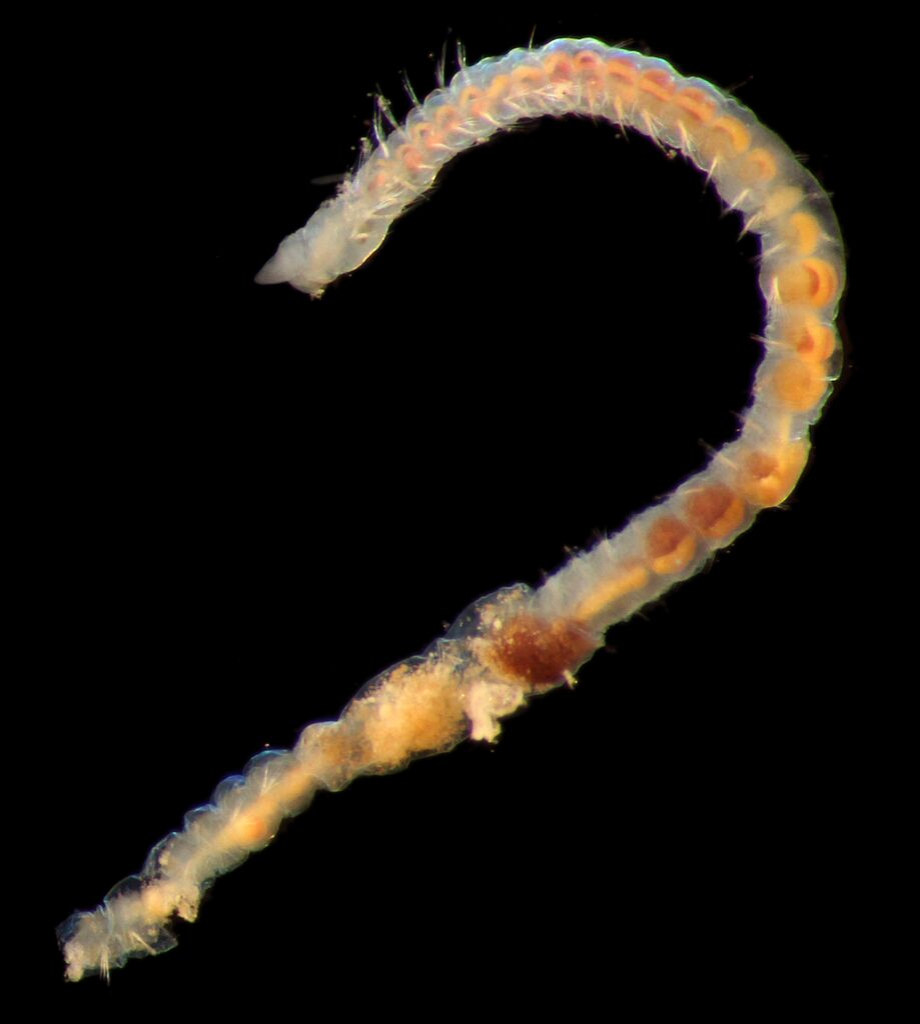
Cirratulidae sp. (NHM_530), live specimen NHM_1268 in dorsolateral view.

Damaged specimens (Fig. 20) consistent with placement within family Cirratulidae, based on morphology and DNA.

#### Cirratulidae sp. (NHM_734)

B173DCCE-B8E0-500F-9ACE-CA48BDA3385A

##### Materials

- **Type status:** Other material. **Occurrence:** recordNumber: NHM_0734; recordedBy: Adrian Glover | Helena Wiklund | Thomas Dahlgren | Madeleine Brasier; individualCount: 1; preparations: Tissue voucher stored in 80% non-denatured ethanol aqueous solution | DNA voucher stored in buffer; otherCatalogNumbers: 0109405383 | 0174126621; associatedSequences: OQ746545 (16S) | OQ746853 (18S) | OQ738534 (COI); occurrenceID: B9BD1477-E343-5B43-8335-1A7EE6EBF14B; **Taxon:** taxonConceptID: Cirratulidae sp. (NHM_734); scientificName: Cirratulidae; kingdom: Animalia; phylum: Annelida; class: Polychaeta; order: Terebellida; family: Cirratulidae; taxonRank: family; scientificNameAuthorship: Ryckholt, 1851; **Location:** waterBody: Pacific; stateProvince: Clarion Clipperton Zone; locality: UK Seabed Resources Ltd exploration area UK-1 Stratum B; verbatimLocality: UK1 Stratum B; maximumDepthInMeters: 4425; locationRemarks: Deployment EB02; at Station U5; from R/V Thomas G. Thompson Cruise no. TN319; verbatimLatitude: 12'32.23; verbatimLongitude: 116'36.25; decimalLatitude: 12.53717; decimalLongitude: -116.60417; geodeticDatum: WGS84; **Identification:** identifiedBy: Helena Wiklund | Lenka Neal | Thomas Dahlgren | Adrian Glover | Madeleine Brasier | Regan Drennan | Eva Stewart; dateIdentified: 2021-04-20; identificationRemarks: identified by DNA and morphology; **Event:** eventID: UK1_AB02_EB02; samplingProtocol: Brenke Epibenthic Sledge; eventDate: 2015-02-20; eventTime: 06:24; habitat: Abyssal plain; fieldNotes: Collected from epi net (on the epibenthic sledge); **Record Level:** language: en; institutionCode: NHMUK; collectionCode: ZOO; datasetName: ABYSSLINE; basisOfRecord: PreservedSpecimen

##### Distribution

Eastern Clarion-Clipperton Zone, central Pacific Ocean.

##### Diagnosis

**Figure 21.**
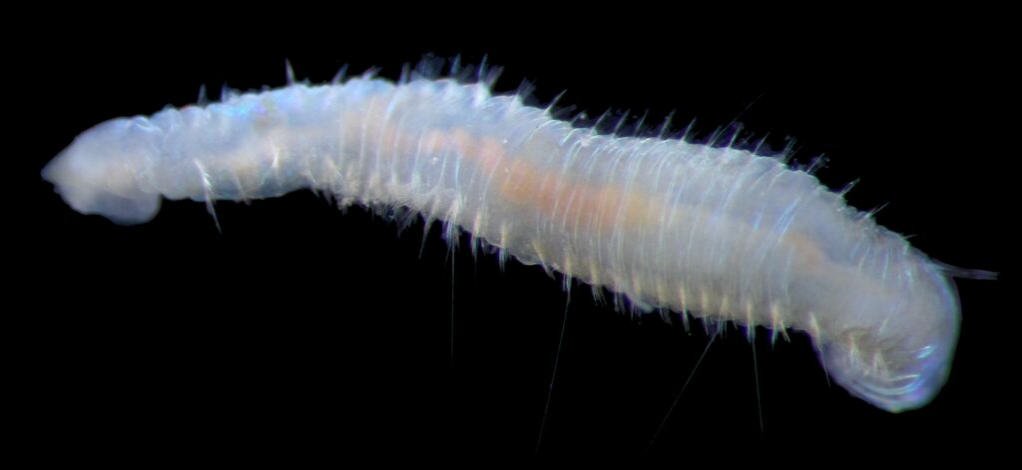
Cirratulidae sp. (NHM_734), anterior fragment of the live specimen NHM_734.

Damaged specimen (Fig. 21) consistent with placement within family Cirratulidae, based on morphology and DNA.

#### Cirratulidae sp. (NHM_904)

4597568E-DC80-57F1-A352-EBCE76577FA8

##### Materials

- **Type status:** Other material. **Occurrence:** recordNumber: NHM_1348I; recordedBy: Adrian Glover | Helena Wiklund | Thomas Dahlgren | Madeleine Brasier; individualCount: 1; preparations: Tissue voucher stored in 80% non-denatured ethanol aqueous solution | DNA voucher stored in buffer; otherCatalogNumbers: 0109405357 | 0174126587; associatedSequences: OQ746670 (16S) | OQ738580 (COI); occurrenceID: 2A094F01-8289-5FB6-826D-5AEA170FC622; **Taxon:** taxonConceptID: Cirratulidae sp. (NHM_904); scientificName: Cirratulidae; kingdom: Animalia; phylum: Annelida; class: Polychaeta; order: Terebellida; family: Cirratulidae; taxonRank: family; scientificNameAuthorship: Ryckholt, 1851; **Location:** waterBody: Pacific; stateProvince: Clarion Clipperton Zone; locality: Ocean Mineral Singapore exploration claim Stratum A; verbatimLocality: OMS Stratum A; maximumDepthInMeters: 4302; locationRemarks: Deployment EB06; at Station S5; from R/V Thomas G. Thompson Cruise no. TN319; verbatimLatitude: 12'15.44; verbatimLongitude: 117'18.13; decimalLatitude: 12.25733; decimalLongitude: -117.30217; geodeticDatum: WGS84; **Identification:** identifiedBy: Helena Wiklund | Lenka Neal | Thomas Dahlgren | Adrian Glover | Madeleine Brasier | Regan Drennan | Eva Stewart; dateIdentified: 2021-04-20; identificationRemarks: identified by DNA and morphology; **Event:** eventID: OMS1_AB02_EB06; samplingProtocol: Brenke Epibenthic Sledge; eventDate: 2015-03-01; eventTime: 04:02; habitat: Abyssal plain; fieldNotes: Collected from epi net (on the epibenthic sledge); **Record Level:** language: en; institutionCode: NHMUK; collectionCode: ZOO; datasetName: ABYSSLINE; basisOfRecord: PreservedSpecimen
- **Type status:** Other material. **Occurrence:** catalogNumber: NHMUK ANEA 2023.363; recordNumber: NHM_0904; recordedBy: Adrian Glover | Helena Wiklund | Thomas Dahlgren | Madeleine Brasier; individualCount: 1; preparations: specimen stored in 80% non-denatured ethanol aqueous solution | DNA voucher stored in buffer; otherCatalogNumbers: 0174126812; associatedSequences: OQ746574 (16S) | OQ746865 (18S) | OQ738542 (COI); occurrenceID: 5A98AB6B-6421-531E-8C96-8DC8A332DDC0; **Taxon:** taxonConceptID: Cirratulidae sp. (NHM_904); scientificName: Cirratulidae; kingdom: Animalia; phylum: Annelida; class: Polychaeta; order: Terebellida; family: Cirratulidae; taxonRank: family; scientificNameAuthorship: Ryckholt, 1851; **Location:** waterBody: Pacific; stateProvince: Clarion Clipperton Zone; locality: UK Seabed Resources Ltd exploration area UK-1 Stratum B; verbatimLocality: UK1 Stratum B; maximumDepthInMeters: 4198; locationRemarks: Deployment EB03; at Station U4; from R/V Thomas G. Thompson Cruise no. TN319; verbatimLatitude: 12'34.28; verbatimLongitude: 116'36.63; decimalLatitude: 12.57133; decimalLongitude: -116.6105; geodeticDatum: WGS84; **Identification:** identifiedBy: Helena Wiklund | Lenka Neal | Thomas Dahlgren | Adrian Glover | Madeleine Brasier | Regan Drennan | Eva Stewart; dateIdentified: 2021-04-20; identificationRemarks: identified by DNA and morphology; **Event:** eventID: UK1_AB02_EB03; samplingProtocol: Brenke Epibenthic Sledge; eventDate: 2015-02-23; eventTime: 05:39; habitat: Abyssal plain; fieldNotes: Collected from epi net (on the epibenthic sledge); **Record Level:** language: en; institutionCode: NHMUK; collectionCode: ZOO; datasetName: ABYSSLINE; basisOfRecord: PreservedSpecimen

##### Distribution

Eastern Clarion-Clipperton Zone, central Pacific Ocean.

##### Diagnosis

**Figure 22.**
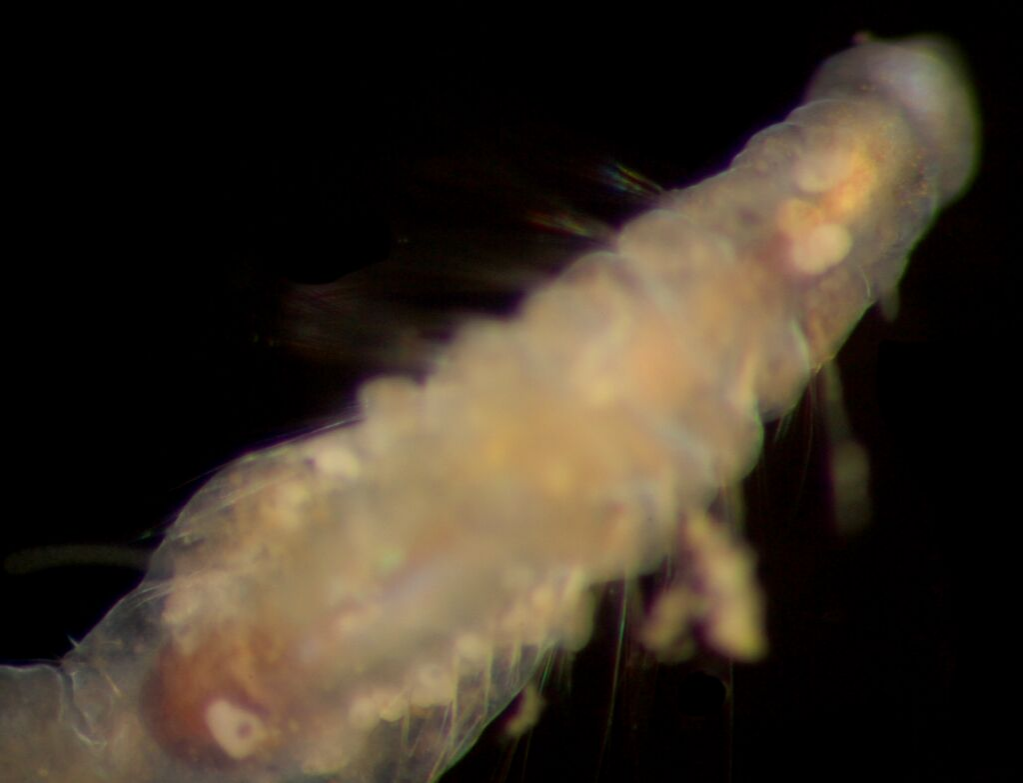
Cirratulidae sp. (NHM_904), anterior end of live specimen NHM_904.

Damaged specimens (Fig. 22) consistent with placement within family Cirratulidae, based on morphology and DNA.

#### Cirratulidae sp. (NHM_915G)

961D762D-6D1E-591E-8A1C-A9694FBAF50F

##### Materials

- **Type status:** Other material. **Occurrence:** catalogNumber: NHMUK ANEA 2023.365; recordNumber: NHM_1271; recordedBy: Adrian Glover | Helena Wiklund | Thomas Dahlgren | Madeleine Brasier; individualCount: 1; preparations: specimen stored in 80% non-denatured ethanol aqueous solution | DNA voucher stored in buffer; otherCatalogNumbers: 0174126613; associatedSequences: OQ746650 (16S); occurrenceID: 3DECDE61-60B5-5027-AA89-5E9EC344DF92; **Taxon:** taxonConceptID: Cirratulidae sp. (NHM_915G); scientificName: Cirratulidae; kingdom: Animalia; phylum: Annelida; class: Polychaeta; order: Terebellida; family: Cirratulidae; taxonRank: family; scientificNameAuthorship: Ryckholt, 1851; **Location:** waterBody: Pacific; stateProvince: Clarion Clipperton Zone; locality: Ocean Mineral Singapore exploration claim Stratum A; verbatimLocality: OMS Stratum A; maximumDepthInMeters: 4302; locationRemarks: Deployment EB06; at Station S5; from R/V Thomas G. Thompson Cruise no. TN319; verbatimLatitude: 12'15.44; verbatimLongitude: 117'18.13; decimalLatitude: 12.25733; decimalLongitude: -117.30217; geodeticDatum: WGS84; **Identification:** identifiedBy: Helena Wiklund | Lenka Neal | Thomas Dahlgren | Adrian Glover | Madeleine Brasier | Regan Drennan | Eva Stewart; dateIdentified: 2021-04-20; identificationRemarks: identified by DNA and morphology; **Event:** eventID: OMS1_AB02_EB06; samplingProtocol: Brenke Epibenthic Sledge; eventDate: 2015-03-01; eventTime: 04:02; habitat: Abyssal plain; fieldNotes: Collected from epi net (on the epibenthic sledge); **Record Level:** language: en; institutionCode: NHMUK; collectionCode: ZOO; datasetName: ABYSSLINE; basisOfRecord: PreservedSpecimen
- **Type status:** Other material. **Occurrence:** catalogNumber: NHMUK ANEA 2023.367; recordNumber: NHM_1948B; recordedBy: Adrian Glover | Helena Wiklund | Thomas Dahlgren | Madeleine Brasier; individualCount: 1; preparations: specimen stored in 80% non-denatured ethanol aqueous solution | DNA voucher stored in buffer; otherCatalogNumbers: 0174126149; associatedSequences: OQ746740 (16S); occurrenceID: 71C5D278-2C87-538C-9950-51E9499B8BEE; **Taxon:** taxonConceptID: Cirratulidae sp. (NHM_915G); scientificName: Cirratulidae; kingdom: Animalia; phylum: Annelida; class: Polychaeta; order: Terebellida; family: Cirratulidae; taxonRank: family; scientificNameAuthorship: Ryckholt, 1851; **Location:** waterBody: Pacific; stateProvince: Clarion Clipperton Zone; locality: Ocean Mineral Singapore exploration claim Stratum A; verbatimLocality: OMS Stratum A; maximumDepthInMeters: 4094; locationRemarks: Deployment EB11; at Station S10; from R/V Thomas G. Thompson Cruise no. TN319; verbatimLatitude: 12°02.49’; verbatimLongitude: 117°13.03’; decimalLatitude: 12.0415; decimalLongitude: -117.21717; geodeticDatum: WGS84; **Identification:** identifiedBy: Helena Wiklund | Lenka Neal | Thomas Dahlgren | Adrian Glover | Madeleine Brasier | Regan Drennan | Eva Stewart; dateIdentified: 2021-04-20; identificationRemarks: identified by DNA and morphology; **Event:** eventID: OMS1_AB02_EB11; samplingProtocol: Brenke Epibenthic Sledge; eventDate: 2015-03-13; habitat: Abyssal plain; fieldNotes: Collected from epi net (on the epibenthic sledge); **Record Level:** language: en; institutionCode: NHMUK; collectionCode: ZOO; datasetName: ABYSSLINE; basisOfRecord: PreservedSpecimen
- **Type status:** Other material. **Occurrence:** catalogNumber: NHMUK ANEA 2023.366; recordNumber: NHM_1784; recordedBy: Adrian Glover | Helena Wiklund | Thomas Dahlgren | Madeleine Brasier; individualCount: 1; preparations: specimen stored in 80% non-denatured ethanol aqueous solution | DNA voucher stored in buffer; otherCatalogNumbers: 0174126148; associatedSequences: OQ746720 (16S); occurrenceID: C26DC393-E47F-551B-9E90-1E968E3D8419; **Taxon:** taxonConceptID: Cirratulidae sp. (NHM_915G); scientificName: Cirratulidae; kingdom: Animalia; phylum: Annelida; class: Polychaeta; order: Terebellida; family: Cirratulidae; taxonRank: family; scientificNameAuthorship: Ryckholt, 1851; **Location:** waterBody: Pacific; stateProvince: Clarion Clipperton Zone; locality: Ocean Mineral Singapore exploration claim Stratum A; verbatimLocality: OMS Stratum A; maximumDepthInMeters: 4045; locationRemarks: Deployment EB10; at Station S7; from R/V Thomas G. Thompson Cruise no. TN319; verbatimLatitude: 12'10.43; verbatimLongitude: 117'11.57; decimalLatitude: 12.17383; decimalLongitude: -117.19283; geodeticDatum: WGS84; **Identification:** identifiedBy: Helena Wiklund | Lenka Neal | Thomas Dahlgren | Adrian Glover | Madeleine Brasier | Regan Drennan | Eva Stewart; dateIdentified: 2021-04-20; identificationRemarks: identified by DNA and morphology; **Event:** eventID: OMS1_AB02_EB10; samplingProtocol: Brenke Epibenthic Sledge; eventDate: 2015-03-11; eventTime: 22:49; habitat: Abyssal plain; fieldNotes: Collected from epi net (on the epibenthic sledge); **Record Level:** language: en; institutionCode: NHMUK; collectionCode: ZOO; datasetName: ABYSSLINE; basisOfRecord: PreservedSpecimen
- **Type status:** Other material. **Occurrence:** catalogNumber: NHMUK ANEA 2023.364; recordNumber: NHM_0915G; recordedBy: Adrian Glover | Helena Wiklund | Thomas Dahlgren | Madeleine Brasier; individualCount: 1; preparations: specimen stored in 80% non-denatured ethanol aqueous solution | DNA voucher stored in buffer; otherCatalogNumbers: 0174126547; associatedSequences: OQ746582 (16S) | OQ746866 (18S) | OQ738545 (COI); occurrenceID: BA345B22-C418-55F2-9AA5-DE601137D123; **Taxon:** taxonConceptID: Cirratulidae sp. (NHM_915G); scientificName: Cirratulidae; kingdom: Animalia; phylum: Annelida; class: Polychaeta; order: Terebellida; family: Cirratulidae; taxonRank: family; scientificNameAuthorship: Ryckholt, 1851; **Location:** waterBody: Pacific; stateProvince: Clarion Clipperton Zone; locality: UK Seabed Resources Ltd exploration area UK-1 Stratum B; verbatimLocality: UK1 Stratum B; maximumDepthInMeters: 4198; locationRemarks: Deployment EB03; at Station U4; from R/V Thomas G. Thompson Cruise no. TN319; verbatimLatitude: 12'34.28; verbatimLongitude: 116'36.63; decimalLatitude: 12.57133; decimalLongitude: -116.6105; geodeticDatum: WGS84; **Identification:** identifiedBy: Helena Wiklund | Lenka Neal | Thomas Dahlgren | Adrian Glover | Madeleine Brasier | Regan Drennan | Eva Stewart; dateIdentified: 2021-04-20; identificationRemarks: identified by DNA and morphology; **Event:** eventID: UK1_AB02_EB03; samplingProtocol: Brenke Epibenthic Sledge; eventDate: 2015-02-23; eventTime: 05:39; habitat: Abyssal plain; fieldNotes: Collected from epi net (on the epibenthic sledge); **Record Level:** language: en; institutionCode: NHMUK; collectionCode: ZOO; datasetName: ABYSSLINE; basisOfRecord: PreservedSpecimen

##### Distribution

Eastern Clarion-Clipperton Zone, central Pacific Ocean.

##### Diagnosis

**Figure 23.**
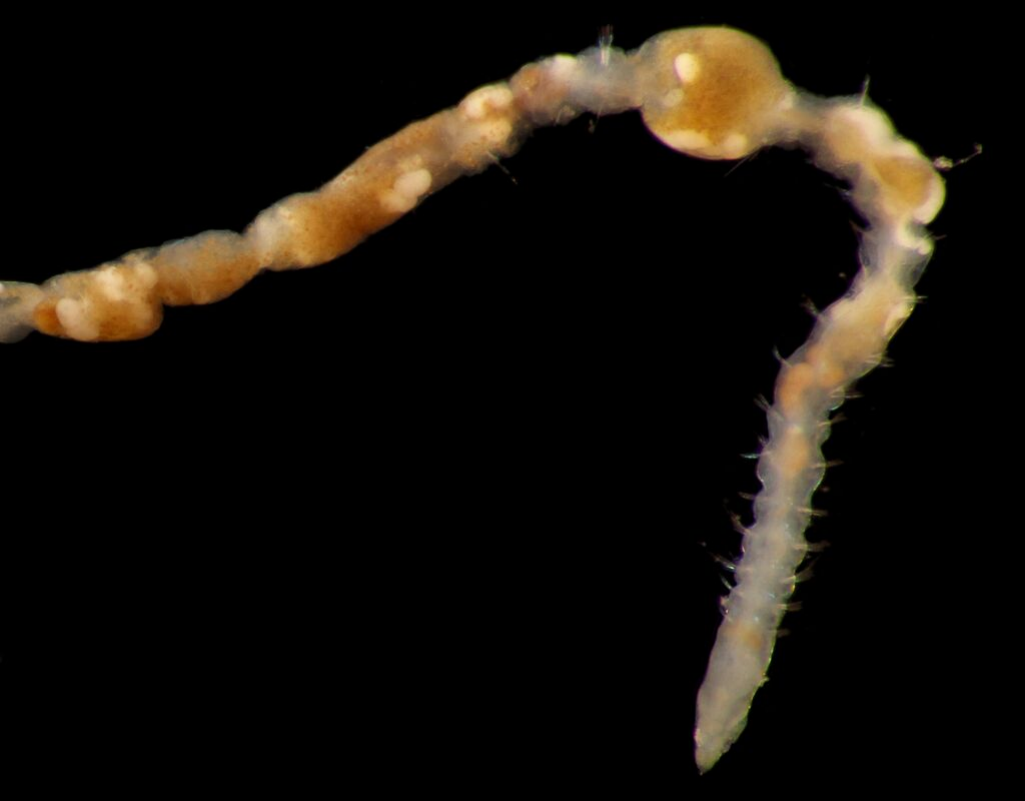
Cirratulidae sp. (NHM_915G), anterior of fragment of live specimen NHM_1271.

Damaged specimens (Fig. 23) consistent with placement within family Cirratulidae, based on morphology and DNA.

#### Cirratulidae sp. (NHM_945C)

A082FFFE-6569-50D2-BE03-5FEB0B73A128

##### Materials

- **Type status:** Other material. **Occurrence:** catalogNumber: NHMUK ANEA 2023.368; recordNumber: NHM_0945C; recordedBy: Adrian Glover | Helena Wiklund | Thomas Dahlgren | Madeleine Brasier; individualCount: 1; preparations: specimen stored in 80% non-denatured ethanol aqueous solution | DNA voucher stored in buffer; otherCatalogNumbers: 0174126546; associatedSequences: OQ746594 (16S) | OQ746867 (18S) | OQ738551 (COI); occurrenceID: C3206274-F5C3-52DD-9CDB-DAE9C198C20B; **Taxon:** taxonConceptID: Cirratulidae sp. (NHM_945C); scientificName: Cirratulidae; kingdom: Animalia; phylum: Annelida; class: Polychaeta; order: Terebellida; family: Cirratulidae; taxonRank: family; scientificNameAuthorship: Ryckholt, 1851; **Location:** waterBody: Pacific; stateProvince: Clarion Clipperton Zone; locality: UK Seabed Resources Ltd exploration area UK-1 Stratum B; verbatimLocality: UK1 Stratum B; maximumDepthInMeters: 4198; locationRemarks: Deployment EB03; at Station U4; from R/V Thomas G. Thompson Cruise no. TN319; verbatimLatitude: 12'34.28; verbatimLongitude: 116'36.63; decimalLatitude: 12.57133; decimalLongitude: -116.6105; geodeticDatum: WGS84; **Identification:** identifiedBy: Helena Wiklund | Lenka Neal | Thomas Dahlgren | Adrian Glover | Madeleine Brasier | Regan Drennan | Eva Stewart; dateIdentified: 2021-04-20; identificationRemarks: identified by DNA and morphology; **Event:** eventID: UK1_AB02_EB03; samplingProtocol: Brenke Epibenthic Sledge; eventDate: 2015-02-23; eventTime: 05:39; habitat: Abyssal plain; fieldNotes: Collected from epi net (on the epibenthic sledge); **Record Level:** language: en; institutionCode: NHMUK; collectionCode: ZOO; datasetName: ABYSSLINE; basisOfRecord: PreservedSpecimen

##### Distribution

Eastern Clarion-Clipperton Zone, central Pacific Ocean.

##### Diagnosis

**Figure 24.**
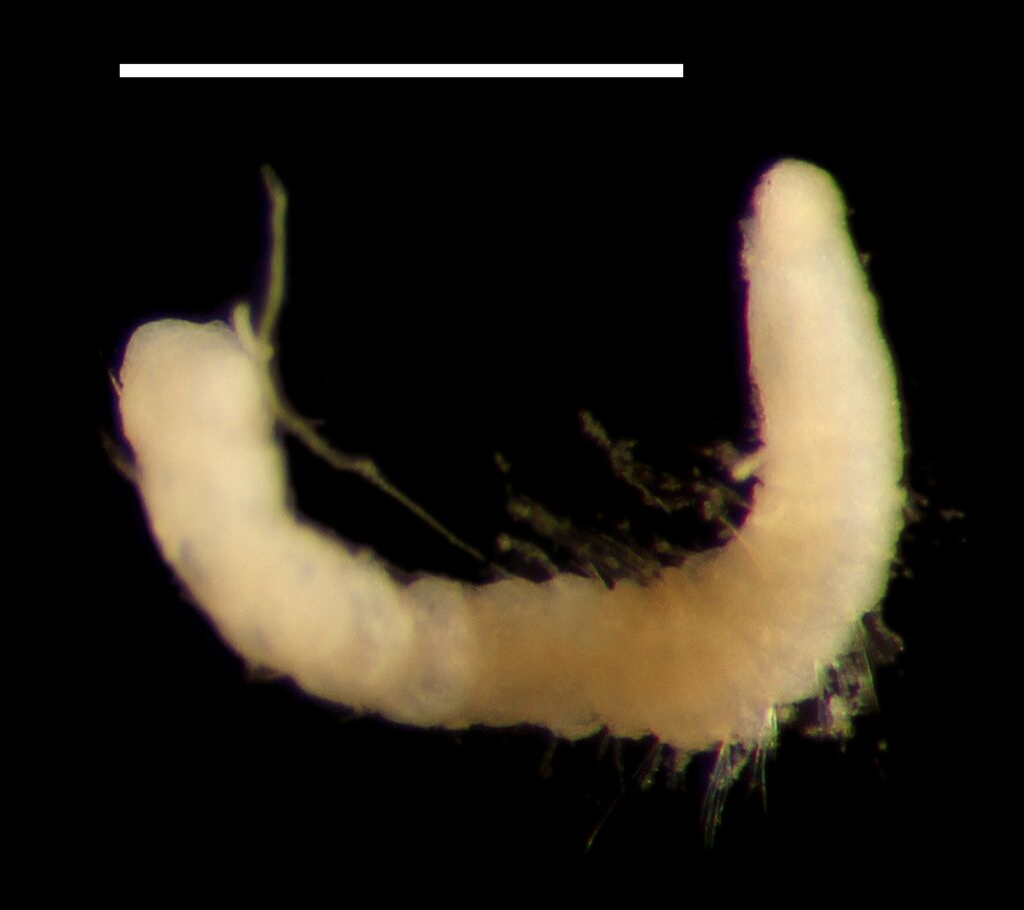
Cirratulidae sp. (NHM_945C), anterior end of live specimen NHM_945C in lateral view. Scale bar: 1 mm.

Damaged specimen (Fig. 24) consistent with placement within family Cirratulidae, based on morphology and DNA.

#### Cirratulidae sp. (NHM_1001)

04C66540-B7A0-59D0-A340-05E21FC79F3B

##### Materials

- **Type status:** Other material. **Occurrence:** catalogNumber: NHMUK ANEA 2023.343; recordNumber: NHM_1001; recordedBy: Adrian Glover | Helena Wiklund | Thomas Dahlgren | Madeleine Brasier; individualCount: 1; preparations: specimen stored in 80% non-denatured ethanol aqueous solution | DNA voucher stored in buffer; otherCatalogNumbers: 0174126582; associatedSequences: OQ746602 (16S) | OQ746869 (18S) | OQ738554 (COI); occurrenceID: 38F92F98-0ADD-5143-B6D9-9429F17B9BFE; **Taxon:** taxonConceptID: Cirratulidae sp. (NHM_1001); scientificName: Cirratulidae; kingdom: Animalia; phylum: Annelida; class: Polychaeta; order: Terebellida; family: Cirratulidae; taxonRank: family; scientificNameAuthorship: Ryckholt, 1851; **Location:** waterBody: Pacific; stateProvince: Clarion Clipperton Zone; locality: Ocean Mineral Singapore exploration claim Stratum A; verbatimLocality: OMS Stratum A; maximumDepthInMeters: 4122; locationRemarks: Deployment EB04; at Station S1; from R/V Thomas G. Thompson Cruise no. TN319; verbatimLatitude: 12'08.02; verbatimLongitude: 117'17.52; decimalLatitude: 12.13367; decimalLongitude: -117.292; geodeticDatum: WGS84; **Identification:** identifiedBy: Helena Wiklund | Lenka Neal | Thomas Dahlgren | Adrian Glover | Madeleine Brasier | Regan Drennan | Eva Stewart; dateIdentified: 2021-04-20; identificationRemarks: identified by DNA and morphology; **Event:** eventID: OMS1_AB02_EB04; samplingProtocol: Brenke Epibenthic Sledge; eventDate: 2015-02-24; eventTime: 19:10; habitat: Abyssal plain; fieldNotes: Collected from epi net (on the epibenthic sledge); **Record Level:** language: en; institutionCode: NHMUK; collectionCode: ZOO; datasetName: ABYSSLINE; basisOfRecord: PreservedSpecimen

##### Distribution

Eastern Clarion-Clipperton Zone, central Pacific Ocean.

##### Diagnosis

**Figure 25.**
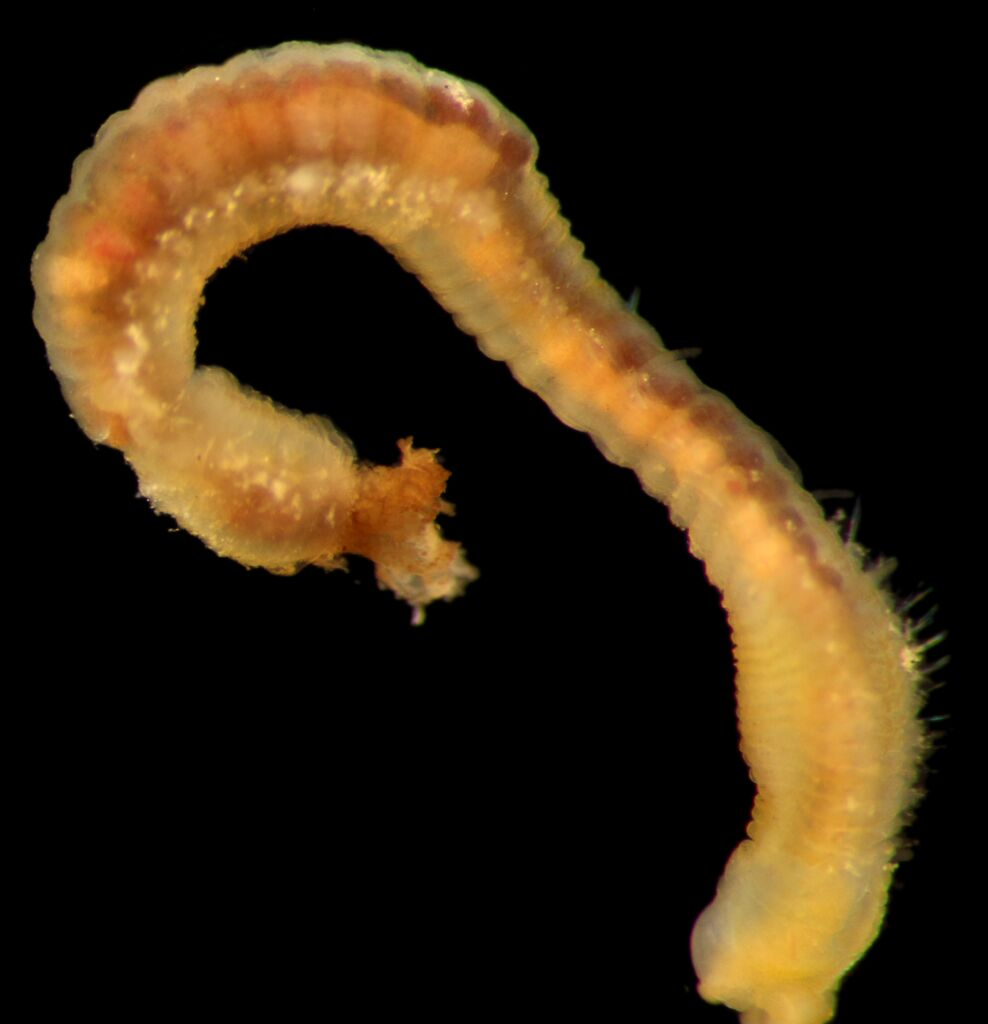
Cirratulidae sp. (NHM_1001), anterior fragment of live specimen NHM_1001 in lateral view.

Damaged specimen (Fig. 25) consistent with placement within family Cirratulidae, based on morphology and DNA.

#### Cirratulidae sp. (NHM_1235)

FECD648F-3313-53B6-9C6D-7E8AE57A8F0E

##### Materials

- **Type status:** Other material. **Occurrence:** catalogNumber: NHMUK ANEA 2023.344; recordNumber: NHM_1235; recordedBy: Adrian Glover | Helena Wiklund | Thomas Dahlgren | Madeleine Brasier; individualCount: 1; preparations: specimen stored in 80% non-denatured ethanol aqueous solution | DNA voucher stored in buffer; otherCatalogNumbers: 0174126761; associatedSequences: OQ746642 (16S) | OQ746882 (18S) | OQ738572 (COI); occurrenceID: 9E7B92EE-AFA2-56E7-A102-AF9FF59E2832; **Taxon:** taxonConceptID: Cirratulidae sp. (NHM_1235); scientificName: Cirratulidae; kingdom: Animalia; phylum: Annelida; class: Polychaeta; order: Terebellida; family: Cirratulidae; taxonRank: family; scientificNameAuthorship: Ryckholt, 1851; **Location:** waterBody: Pacific; stateProvince: Clarion Clipperton Zone; locality: Ocean Mineral Singapore exploration claim Stratum A; verbatimLocality: OMS Stratum A; maximumDepthInMeters: 4090; locationRemarks: Deployment BC11; at Station S5; from R/V Thomas G. Thompson Cruise no. TN319; verbatimLatitude: 12'13.0425; verbatimLongitude: 117'19.5229; decimalLatitude: 12.21738; decimalLongitude: -117.32538; geodeticDatum: WGS84; **Identification:** identifiedBy: Helena Wiklund | Lenka Neal | Thomas Dahlgren | Adrian Glover | Madeleine Brasier | Regan Drennan | Eva Stewart; dateIdentified: 2021-04-20; identificationRemarks: identified by DNA and morphology; **Event:** eventID: OMS1_AB02_BC11; samplingProtocol: USNEL Box Core; eventDate: 2015-02-28; eventTime: 12:09; habitat: Abyssal plain; fieldNotes: Collected from 0-2 cm layer of box core using a 300 micron sieve; **Record Level:** language: en; institutionCode: NHMUK; collectionCode: ZOO; datasetName: ABYSSLINE; basisOfRecord: PreservedSpecimen
- **Type status:** Other material. **Occurrence:** recordNumber: NHM_1236; recordedBy: Adrian Glover | Helena Wiklund | Thomas Dahlgren | Madeleine Brasier; individualCount: 1; preparations: Tissue voucher stored in 80% non-denatured ethanol aqueous solution | DNA voucher stored in buffer; otherCatalogNumbers: 0109405405 | 0174126561; associatedSequences: OQ746643 (16S) | OQ738573 (COI); occurrenceID: 68CF5773-79FC-5354-800D-E3FB33F56BDA; **Taxon:** taxonConceptID: Cirratulidae sp. (NHM_1235); scientificName: Cirratulidae; kingdom: Animalia; phylum: Annelida; class: Polychaeta; order: Terebellida; family: Cirratulidae; taxonRank: family; scientificNameAuthorship: Ryckholt, 1851; **Location:** waterBody: Pacific; stateProvince: Clarion Clipperton Zone; locality: Ocean Mineral Singapore exploration claim Stratum A; verbatimLocality: OMS Stratum A; maximumDepthInMeters: 4090; locationRemarks: Deployment BC11; at Station S5; from R/V Thomas G. Thompson Cruise no. TN319; verbatimLatitude: 12'13.0425; verbatimLongitude: 117'19.5229; decimalLatitude: 12.21738; decimalLongitude: -117.32538; geodeticDatum: WGS84; **Identification:** identifiedBy: Helena Wiklund | Lenka Neal | Thomas Dahlgren | Adrian Glover | Madeleine Brasier | Regan Drennan | Eva Stewart; dateIdentified: 2021-04-20; identificationRemarks: identified by DNA and morphology; **Event:** eventID: OMS1_AB02_BC11; samplingProtocol: USNEL Box Core; eventDate: 2015-02-28; eventTime: 12:09; habitat: Abyssal plain; fieldNotes: Collected from 0-2 cm layer of box core using a 300 micron sieve; **Record Level:** language: en; institutionCode: NHMUK; collectionCode: ZOO; datasetName: ABYSSLINE; basisOfRecord: PreservedSpecimen
- **Type status:** Other material. **Occurrence:** catalogNumber: NHMUK ANEA 2023.345; recordNumber: NHM_2267; recordedBy: Adrian Glover | Helena Wiklund | Thomas Dahlgren | Madeleine Brasier; individualCount: 1; preparations: specimen stored in 80% non-denatured ethanol aqueous solution | DNA voucher stored in buffer; otherCatalogNumbers: 0174126160; associatedSequences: OQ746768 (16S) | OQ738610 (COI); occurrenceID: 575F84CD-A6B7-5A27-B43C-1C505F4DA337; **Taxon:** taxonConceptID: Cirratulidae sp. (NHM_1235); scientificName: Cirratulidae; kingdom: Animalia; phylum: Annelida; class: Polychaeta; order: Terebellida; family: Cirratulidae; taxonRank: family; scientificNameAuthorship: Ryckholt, 1851; **Location:** waterBody: Pacific; stateProvince: Clarion Clipperton Zone; locality: Ocean Mineral Singapore exploration claim Stratum A; verbatimLocality: OMS Stratum A; maximumDepthInMeters: 4302; locationRemarks: Deployment EB06; at Station S5; from R/V Thomas G. Thompson Cruise no. TN319; verbatimLatitude: 12'15.44; verbatimLongitude: 117'18.13; decimalLatitude: 12.25733; decimalLongitude: -117.30217; geodeticDatum: WGS84; **Identification:** identifiedBy: Helena Wiklund | Lenka Neal | Thomas Dahlgren | Adrian Glover | Madeleine Brasier | Regan Drennan | Eva Stewart; dateIdentified: 2021-04-20; identificationRemarks: identified by DNA and morphology; **Event:** eventID: OMS1_AB02_EB06; samplingProtocol: Brenke Epibenthic Sledge; eventDate: 2015-03-01; eventTime: 04:02; habitat: Abyssal plain; fieldNotes: Collected from supra net (on the epibenthic sledge); **Record Level:** language: en; institutionCode: NHMUK; collectionCode: ZOO; datasetName: ABYSSLINE; basisOfRecord: PreservedSpecimen

##### Distribution

Eastern Clarion-Clipperton Zone, central Pacific Ocean.

##### Diagnosis

**Figure 26.**
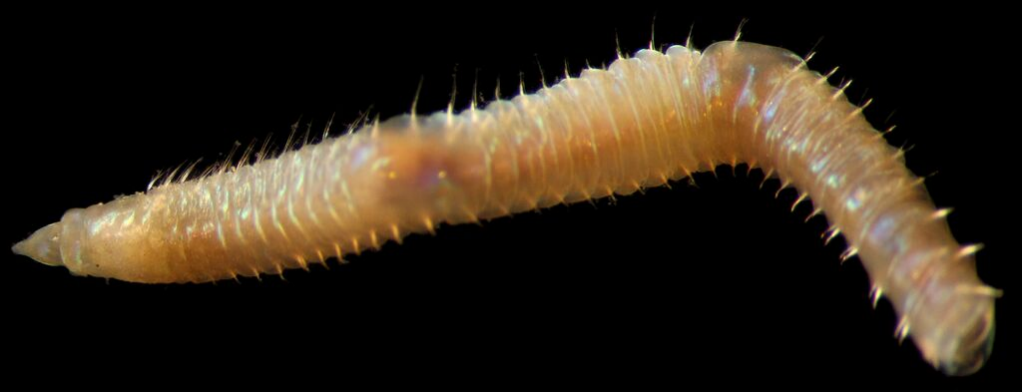
Cirratulidae sp. (NHM_1235), anterior end of live specimen NHM_1235.

Damaged specimen (Fig. 26) consistent with placement within family Cirratulidae, based on morphology and DNA.

#### Cirratulidae sp. (NHM_1429)

B32194E0-39F8-586C-B430-D369BDB20194

##### Materials

- **Type status:** Other material. **Occurrence:** catalogNumber: NHMUK ANEA 2023.346; recordNumber: NHM_1429; recordedBy: Adrian Glover | Helena Wiklund | Thomas Dahlgren | Madeleine Brasier; individualCount: 1; preparations: specimen stored in 80% non-denatured ethanol aqueous solution | DNA voucher stored in buffer; otherCatalogNumbers: 0174126795; associatedSequences: OQ746679 (16S) | OQ738584 (COI); occurrenceID: A47A987B-669A-5489-B17B-0D9A6B1D5575; **Taxon:** taxonConceptID: Cirratulidae sp. (NHM_1429); scientificName: Cirratulidae; kingdom: Animalia; phylum: Annelida; class: Polychaeta; order: Terebellida; family: Cirratulidae; taxonRank: family; scientificNameAuthorship: Ryckholt, 1851; **Location:** waterBody: Pacific; stateProvince: Clarion Clipperton Zone; locality: UK Seabed Resources Ltd exploration area UK-1 Stratum B; verbatimLocality: UK1 Stratum B; maximumDepthInMeters: 4137; locationRemarks: Deployment EB07; at Station U7; from R/V Thomas G. Thompson Cruise no. TN319; verbatimLatitude: 12'27.26; verbatimLongitude: 116'36.77; decimalLatitude: 12.45433; decimalLongitude: -116.61283; geodeticDatum: WGS84; **Identification:** identifiedBy: Helena Wiklund | Lenka Neal | Thomas Dahlgren | Adrian Glover | Madeleine Brasier | Regan Drennan | Eva Stewart; dateIdentified: 2021-04-20; identificationRemarks: identified by DNA and morphology; **Event:** eventID: UK1_AB02_EB07; samplingProtocol: Brenke Epibenthic Sledge; eventDate: 2015-03-03; eventTime: 20:40; habitat: Abyssal plain; fieldNotes: Collected from epi net (on the epibenthic sledge); **Record Level:** language: en; institutionCode: NHMUK; collectionCode: ZOO; datasetName: ABYSSLINE; basisOfRecord: PreservedSpecimen

##### Distribution

Eastern Clarion-Clipperton Zone, central Pacific Ocean.

##### Diagnosis

**Figure 27.**
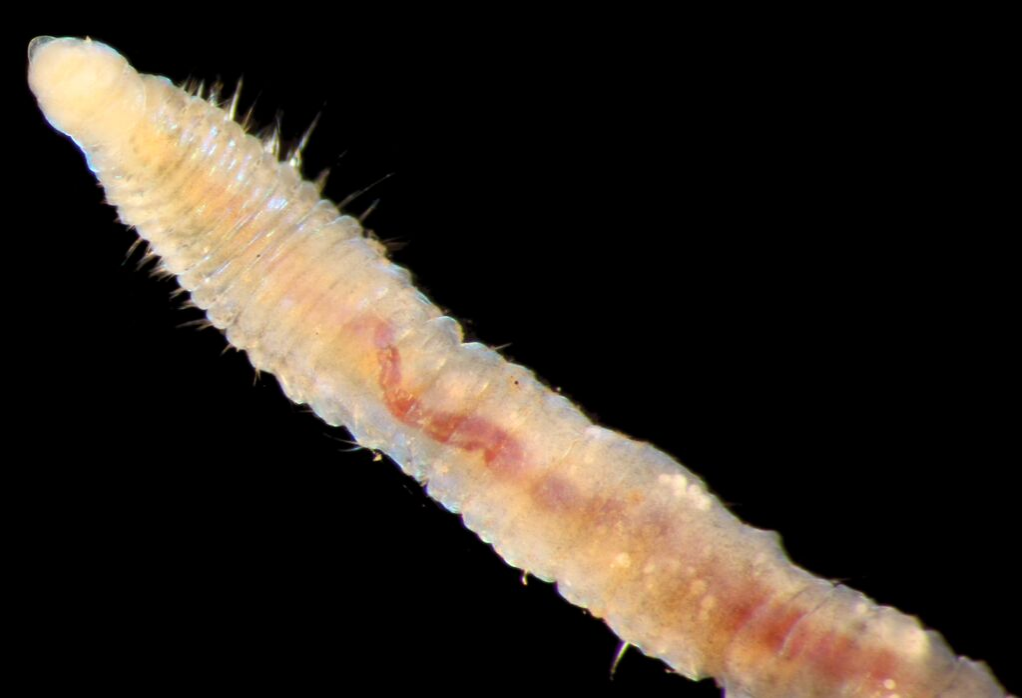
Cirratulidae sp. (NHM_1429), anterior end of live specimen NHM_1429.

Damaged specimen (Fig. 27) consistent with placement within family Cirratulidae, based on morphology and DNA.

#### Cirratulidae sp. (NHM_1518)

D0D035A8-A33F-5A69-9947-D0D80091B2E5

##### Materials

- **Type status:** Other material. **Occurrence:** catalogNumber: NHMUK ANEA 2023.347; recordNumber: NHM_1518; recordedBy: Adrian Glover | Helena Wiklund | Thomas Dahlgren | Madeleine Brasier; individualCount: 1; preparations: specimen stored in 80% non-denatured ethanol aqueous solution | DNA voucher stored in buffer; otherCatalogNumbers: 0174126217; associatedSequences: OQ746690 (16S) | OQ738592 (COI); occurrenceID: 3F24B635-16EE-501C-B5D6-1489D94A1E25; **Taxon:** taxonConceptID: Cirratulidae sp. (NHM_1518); scientificName: Cirratulidae; kingdom: Animalia; phylum: Annelida; class: Polychaeta; order: Terebellida; family: Cirratulidae; taxonRank: family; scientificNameAuthorship: Ryckholt, 1851; **Location:** waterBody: Pacific; stateProvince: Clarion Clipperton Zone; locality: UK Seabed Resources Ltd exploration area UK-1 Stratum B; verbatimLocality: UK1 Stratum B; maximumDepthInMeters: 4252; locationRemarks: Deployment EB08; at Station U11; from R/V Thomas G. Thompson Cruise no. TN319; verbatimLatitude: 12'30.79; verbatimLongitude: 116'29.48; decimalLatitude: 12.51317; decimalLongitude: -116.49133; geodeticDatum: WGS84; **Identification:** identifiedBy: Helena Wiklund | Lenka Neal | Thomas Dahlgren | Adrian Glover | Madeleine Brasier | Regan Drennan | Eva Stewart; dateIdentified: 2021-04-20; identificationRemarks: identified by DNA and morphology; **Event:** eventID: UK1_AB02_EB08; samplingProtocol: Brenke Epibenthic Sledge; eventDate: 2015-03-05; eventTime: 18:53; habitat: Abyssal plain; fieldNotes: Collected from epi net (on the epibenthic sledge); **Record Level:** language: en; institutionCode: NHMUK; collectionCode: ZOO; datasetName: ABYSSLINE; basisOfRecord: PreservedSpecimen

##### Distribution

Eastern Clarion-Clipperton Zone, central Pacific Ocean.

##### Diagnosis

**Figure 28.**
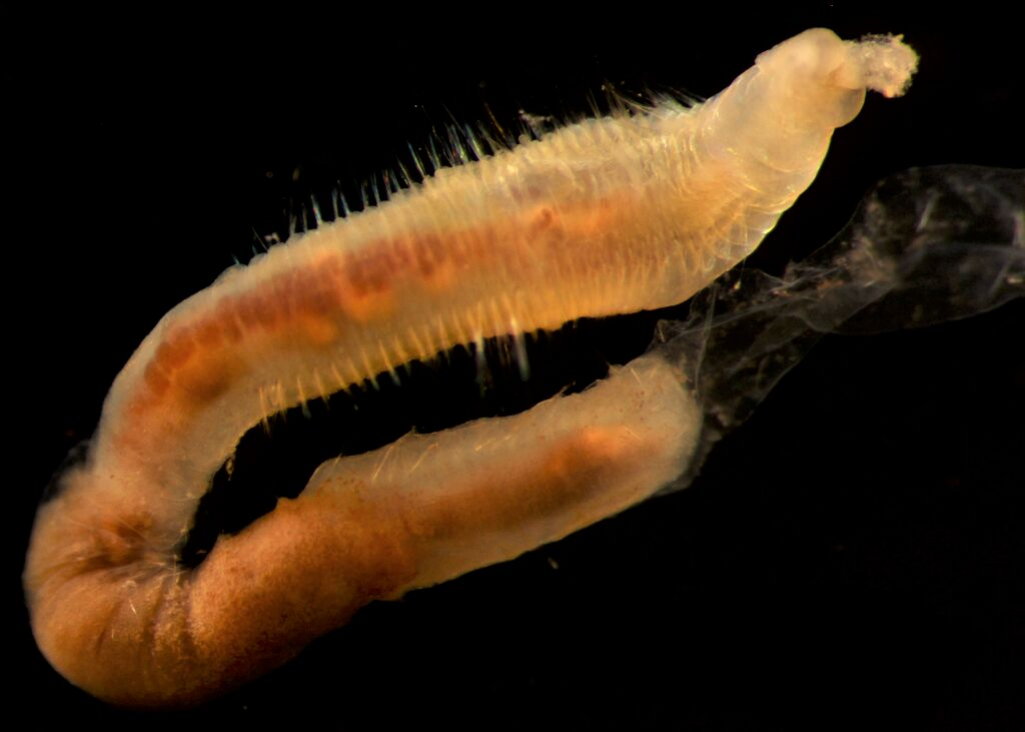
Cirratulidae sp. (NHM_1518), anterior end of live specimen NHM_1518 in dorsal view.

Damaged specimen (Fig. 28) consistent with placement within family Cirratulidae, based on morphology and DNA.

#### Cirratulidae sp. (NHM_2093)

88C6F3F7-8B70-5ABF-A7A2-A05FA122782A

##### Materials

- **Type status:** Other material. **Occurrence:** catalogNumber: NHMUK ANEA 2023.349; recordNumber: NHM_2093; recordedBy: Adrian Glover | Helena Wiklund | Thomas Dahlgren | Madeleine Brasier; individualCount: 1; preparations: specimen stored in 80% non-denatured ethanol aqueous solution | DNA voucher stored in buffer; otherCatalogNumbers: 0174126762; associatedSequences: OQ746749 (16S) | OQ746903 (18S) | OQ738604 (COI); occurrenceID: C28A4742-08BA-53DB-8CEB-CBBE8834F4E9; **Taxon:** taxonConceptID: Cirratulidae sp. (NHM_2093); scientificName: Cirratulidae; kingdom: Animalia; phylum: Annelida; class: Polychaeta; order: Terebellida; family: Cirratulidae; taxonRank: family; scientificNameAuthorship: Ryckholt, 1851; **Location:** waterBody: Pacific; stateProvince: Clarion Clipperton Zone; locality: Area of Particular Interest APEI-6; verbatimLocality: APEI-6; maximumDepthInMeters: 4026; locationRemarks: Deployment EB13; at Station APEI; from R/V Thomas G. Thompson Cruise no. TN319; verbatimLatitude: 19 27.874; verbatimLongitude: 120 01.525; decimalLatitude: 19.46457; decimalLongitude: -120.02542; geodeticDatum: WGS84; **Identification:** identifiedBy: Helena Wiklund | Lenka Neal | Thomas Dahlgren | Adrian Glover | Madeleine Brasier | Regan Drennan | Eva Stewart; dateIdentified: 2021-04-20; identificationRemarks: identified by DNA and morphology; **Event:** eventID: APEI6_AB02_EB13; samplingProtocol: Brenke Epibenthic Sledge; eventDate: 2015-03-20; eventTime: 16:12; habitat: Abyssal plain; fieldNotes: Collected from epi net (on the epibenthic sledge); **Record Level:** language: en; institutionCode: NHMUK; collectionCode: ZOO; datasetName: ABYSSLINE; basisOfRecord: PreservedSpecimen

##### Distribution

Eastern Clarion-Clipperton Zone, central Pacific Ocean.

##### Diagnosis

**Figure 29.**
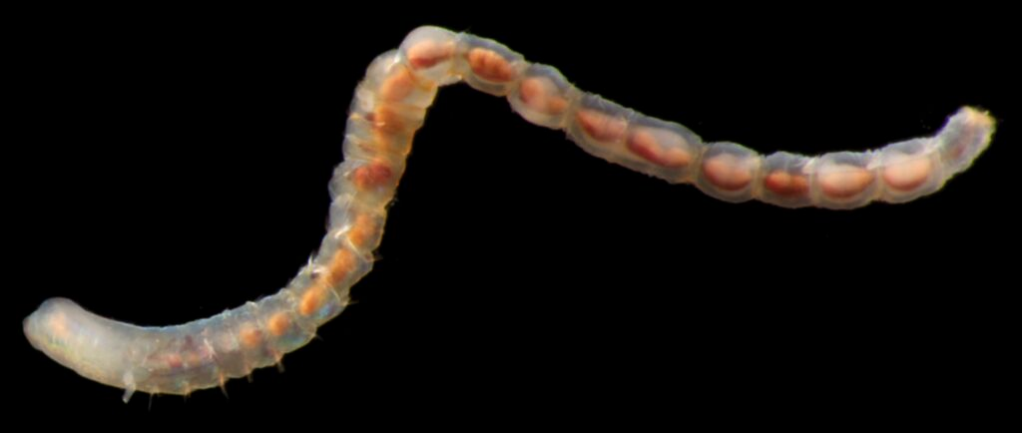
Cirratulidae sp. (NHM_2093), anterior end of live specimen NHM_2093 in lateral view.

Damaged specimen (Fig. 29) consistent with placement within family Cirratulidae, based on morphology and DNA.

#### Cirratulidae sp. (NHM_2163)

AC295316-93DE-523E-A5AF-D6B18A6EB126

##### Materials

- **Type status:** Other material. **Occurrence:** catalogNumber: NHMUK ANEA 2023.353; recordNumber: NHM_2163; recordedBy: Adrian Glover | Helena Wiklund | Thomas Dahlgren | Madeleine Brasier; individualCount: 1; preparations: specimen stored in 80% non-denatured ethanol aqueous solution | DNA voucher stored in buffer; otherCatalogNumbers: 0174126737; associatedSequences: OQ746759 (16S) | OQ746911 (18S); occurrenceID: 89A2C19A-FE0A-5EA4-86BE-63223A93E843; **Taxon:** taxonConceptID: Cirratulidae sp. (NHM_2163); scientificName: Cirratulidae; kingdom: Animalia; phylum: Annelida; class: Polychaeta; order: Terebellida; family: Cirratulidae; taxonRank: family; scientificNameAuthorship: Ryckholt, 1851; **Location:** waterBody: Pacific; stateProvince: Clarion Clipperton Zone; locality: Area of Particular Interest APEI-6; verbatimLocality: APEI-6; maximumDepthInMeters: 4026; locationRemarks: Deployment EB13; at Station APEI; from R/V Thomas G. Thompson Cruise no. TN319; verbatimLatitude: 19 27.874; verbatimLongitude: 120 01.525; decimalLatitude: 19.46457; decimalLongitude: -120.02542; geodeticDatum: WGS84; **Identification:** identifiedBy: Helena Wiklund | Lenka Neal | Thomas Dahlgren | Adrian Glover | Madeleine Brasier | Regan Drennan | Eva Stewart; dateIdentified: 2021-04-20; identificationRemarks: identified by DNA and morphology; **Event:** eventID: APEI6_AB02_EB13; samplingProtocol: Brenke Epibenthic Sledge; eventDate: 2015-03-20; eventTime: 16:12; habitat: Abyssal plain; fieldNotes: Collected from epi net (on the epibenthic sledge); **Record Level:** language: en; institutionCode: NHMUK; collectionCode: ZOO; datasetName: ABYSSLINE; basisOfRecord: PreservedSpecimen
- **Type status:** Other material. **Occurrence:** catalogNumber: NHMUK ANEA 2023.352; recordNumber: NHM_1877; recordedBy: Adrian Glover | Helena Wiklund | Thomas Dahlgren | Madeleine Brasier; individualCount: 1; preparations: specimen stored in 80% non-denatured ethanol aqueous solution | DNA voucher stored in buffer; otherCatalogNumbers: 0174126220; associatedSequences: OQ746727 (16S); occurrenceID: 11DC1AF6-B86B-5EF8-A220-94D3C9A0FD89; **Taxon:** taxonConceptID: Cirratulidae sp. (NHM_2163); scientificName: Cirratulidae; kingdom: Animalia; phylum: Annelida; class: Polychaeta; order: Terebellida; family: Cirratulidae; taxonRank: family; scientificNameAuthorship: Ryckholt, 1851; **Location:** waterBody: Pacific; stateProvince: Clarion Clipperton Zone; locality: Ocean Mineral Singapore exploration claim Stratum A; verbatimLocality: OMS Stratum A; maximumDepthInMeters: 4094; locationRemarks: Deployment EB11; at Station S10; from R/V Thomas G. Thompson Cruise no. TN319; verbatimLatitude: 12°02.49’; verbatimLongitude: 117°13.03’; decimalLatitude: 12.0415; decimalLongitude: -117.21717; geodeticDatum: WGS84; **Identification:** identifiedBy: Helena Wiklund | Lenka Neal | Thomas Dahlgren | Adrian Glover | Madeleine Brasier | Regan Drennan | Eva Stewart; dateIdentified: 2021-04-20; identificationRemarks: identified by DNA and morphology; **Event:** eventID: OMS1_AB02_EB11; samplingProtocol: Brenke Epibenthic Sledge; eventDate: 2015-03-13; habitat: Abyssal plain; fieldNotes: Collected from epi net (on the epibenthic sledge); **Record Level:** language: en; institutionCode: NHMUK; collectionCode: ZOO; datasetName: ABYSSLINE; basisOfRecord: PreservedSpecimen
- **Type status:** Other material. **Occurrence:** catalogNumber: NHMUK ANEA 2023.351; recordNumber: NHM_1782; recordedBy: Adrian Glover | Helena Wiklund | Thomas Dahlgren | Madeleine Brasier; individualCount: 1; preparations: specimen stored in 80% non-denatured ethanol aqueous solution | DNA voucher stored in buffer; otherCatalogNumbers: 0174126163; associatedSequences: OQ746719 (16S); occurrenceID: C60BBE56-41FC-5183-8C71-76F697D875F3; **Taxon:** taxonConceptID: Cirratulidae sp. (NHM_2163); scientificName: Cirratulidae; kingdom: Animalia; phylum: Annelida; class: Polychaeta; order: Terebellida; family: Cirratulidae; taxonRank: family; scientificNameAuthorship: Ryckholt, 1851; **Location:** waterBody: Pacific; stateProvince: Clarion Clipperton Zone; locality: Ocean Mineral Singapore exploration claim Stratum A; verbatimLocality: OMS Stratum A; maximumDepthInMeters: 4045; locationRemarks: Deployment EB10; at Station S7; from R/V Thomas G. Thompson Cruise no. TN319; verbatimLatitude: 12'10.43; verbatimLongitude: 117'11.57; decimalLatitude: 12.17383; decimalLongitude: -117.19283; geodeticDatum: WGS84; **Identification:** identifiedBy: Helena Wiklund | Lenka Neal | Thomas Dahlgren | Adrian Glover | Madeleine Brasier | Regan Drennan | Eva Stewart; dateIdentified: 2021-04-20; identificationRemarks: identified by DNA and morphology; **Event:** eventID: OMS1_AB02_EB10; samplingProtocol: Brenke Epibenthic Sledge; eventDate: 2015-03-11; eventTime: 22:49; habitat: Abyssal plain; fieldNotes: Collected from epi net (on the epibenthic sledge); **Record Level:** language: en; institutionCode: NHMUK; collectionCode: ZOO; datasetName: ABYSSLINE; basisOfRecord: PreservedSpecimen
- **Type status:** Other material. **Occurrence:** catalogNumber: NHMUK ANEA 2023.350; recordNumber: NHM_1096; recordedBy: Adrian Glover | Helena Wiklund | Thomas Dahlgren | Madeleine Brasier; individualCount: 1; preparations: specimen stored in 80% non-denatured ethanol aqueous solution | DNA voucher stored in buffer; otherCatalogNumbers: 0174126568; associatedSequences: OQ746617 (16S); occurrenceID: 3BA57B19-53ED-51F9-9CC8-46117685CA8C; **Taxon:** taxonConceptID: Cirratulidae sp. (NHM_2163); scientificName: Cirratulidae; kingdom: Animalia; phylum: Annelida; class: Polychaeta; order: Terebellida; family: Cirratulidae; taxonRank: family; scientificNameAuthorship: Ryckholt, 1851; **Location:** waterBody: Pacific; stateProvince: Clarion Clipperton Zone; locality: Ocean Mineral Singapore exploration claim Stratum A; verbatimLocality: OMS Stratum A; maximumDepthInMeters: 4100; locationRemarks: Deployment EB05; at Station S2; from R/V Thomas G. Thompson Cruise no. TN319; verbatimLatitude: 12'06.93; verbatimLongitude: 117'09.87; decimalLatitude: 12.1155; decimalLongitude: -117.1645; geodeticDatum: WGS84; **Identification:** identifiedBy: Helena Wiklund | Lenka Neal | Thomas Dahlgren | Adrian Glover | Madeleine Brasier | Regan Drennan | Eva Stewart; dateIdentified: 2021-04-20; identificationRemarks: identified by DNA and morphology; **Event:** eventID: OMS1_AB02_EB05; samplingProtocol: Brenke Epibenthic Sledge; eventDate: 2015-02-26; eventTime: 21:29; habitat: Abyssal plain; fieldNotes: Collected from epi net (on the epibenthic sledge); **Record Level:** language: en; institutionCode: NHMUK; collectionCode: ZOO; datasetName: ABYSSLINE; basisOfRecord: PreservedSpecimen

##### Distribution

Eastern Clarion-Clipperton Zone, central Pacific Ocean.

##### Diagnosis

**Figure 30.**
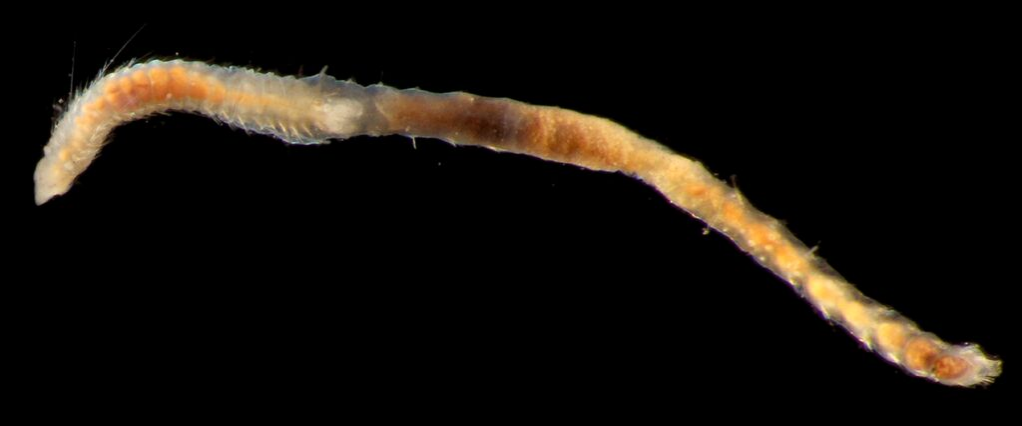
Cirratulidae sp. (NHM_2163), anterior end of live specimen NHM_1096 in dorsal view.

Damaged specimens (Fig. 30) consistent with placement within family Cirratulidae, based on morphology and DNA.

#### Cirratulidae sp. (NHM_172)

56FF271F-D48E-5D5E-B17A-C787D4DAEDC5

##### Materials

- **Type status:** Other material. **Occurrence:** catalogNumber: NHMUK ANEA 2023.342; recordNumber: NHM_2309; recordedBy: Adrian Glover | Helena Wiklund | Thomas Dahlgren | Madeleine Brasier; individualCount: 1; preparations: specimen stored in 80% non-denatured ethanol aqueous solution | DNA voucher stored in buffer; associatedSequences: OQ746771 (16S) | OQ738611 (COI); occurrenceID: BE172C52-935C-5BA3-BAE1-F0711A206E15; **Taxon:** taxonConceptID: Cirratulidae (NHM_172); scientificName: Cirratulidae; kingdom: Animalia; phylum: Annelida; class: Polychaeta; order: Terebellida; family: Cirratulidae; taxonRank: family; scientificNameAuthorship: Ryckholt, 1851; **Location:** waterBody: Pacific; stateProvince: Clarion Clipperton Zone; locality: Ocean Mineral Singapore exploration claim Stratum A; verbatimLocality: OMS Stratum A; maximumDepthInMeters: 4302; locationRemarks: Deployment EB06; at Station S5; from R/V Thomas G. Thompson Cruise no. TN319; verbatimLatitude: 12'15.44; verbatimLongitude: 117'18.13; decimalLatitude: 12.25733; decimalLongitude: -117.30217; geodeticDatum: WGS84; **Identification:** identifiedBy: Helena Wiklund | Lenka Neal | Thomas Dahlgren | Adrian Glover | Madeleine Brasier | Regan Drennan | Eva Stewart; dateIdentified: 2021-04-20; identificationRemarks: identified by DNA and morphology; **Event:** eventID: OMS1_AB02_EB06; samplingProtocol: Brenke Epibenthic Sledge; eventDate: 2015-03-01; eventTime: 04:02; habitat: Abyssal plain; fieldNotes: Collected from supra net (on the epibenthic sledge); **Record Level:** language: en; institutionCode: NHMUK; collectionCode: ZOO; datasetName: ABYSSLINE; basisOfRecord: PreservedSpecimen
- **Type status:** Other material. **Occurrence:** catalogNumber: NHMUK ANEA 2023.341; recordNumber: NHM_1772; recordedBy: Adrian Glover | Helena Wiklund | Thomas Dahlgren | Madeleine Brasier; individualCount: 1; preparations: specimen stored in 80% non-denatured ethanol aqueous solution | DNA voucher stored in buffer; otherCatalogNumbers: 0174126212; associatedSequences: OQ746716 (16S); occurrenceID: DF4DBD4A-8905-5F96-958D-48DD2A9AD350; **Taxon:** taxonConceptID: Cirratulidae (NHM_172); scientificName: Cirratulidae; kingdom: Animalia; phylum: Annelida; class: Polychaeta; order: Terebellida; family: Cirratulidae; taxonRank: family; scientificNameAuthorship: Ryckholt, 1851; **Location:** waterBody: Pacific; stateProvince: Clarion Clipperton Zone; locality: Ocean Mineral Singapore exploration claim Stratum A; verbatimLocality: OMS Stratum A; maximumDepthInMeters: 4045; locationRemarks: Deployment EB10; at Station S7; from R/V Thomas G. Thompson Cruise no. TN319; verbatimLatitude: 12'10.43; verbatimLongitude: 117'11.57; decimalLatitude: 12.17383; decimalLongitude: -117.19283; geodeticDatum: WGS84; **Identification:** identifiedBy: Helena Wiklund | Lenka Neal | Thomas Dahlgren | Adrian Glover | Madeleine Brasier | Regan Drennan | Eva Stewart; dateIdentified: 2021-04-20; identificationRemarks: identified by DNA and morphology; **Event:** eventID: OMS1_AB02_EB10; samplingProtocol: Brenke Epibenthic Sledge; eventDate: 2015-03-11; eventTime: 22:49; habitat: Abyssal plain; fieldNotes: Collected from epi net (on the epibenthic sledge); **Record Level:** language: en; institutionCode: NHMUK; collectionCode: ZOO; datasetName: ABYSSLINE; basisOfRecord: PreservedSpecimen
- **Type status:** Other material. **Occurrence:** catalogNumber: NHMUK ANEA 2023.340; recordNumber: NHM_0915E; recordedBy: Adrian Glover | Helena Wiklund | Thomas Dahlgren | Madeleine Brasier; individualCount: 1; preparations: specimen stored in 80% non-denatured ethanol aqueous solution | DNA voucher stored in buffer; otherCatalogNumbers: 0174126571; associatedSequences: OQ746580 (16S); occurrenceID: 5ACA0F65-7843-5AC6-B52F-4C8349B3CCB9; **Taxon:** taxonConceptID: Cirratulidae (NHM_172); scientificName: Cirratulidae; kingdom: Animalia; phylum: Annelida; class: Polychaeta; order: Terebellida; family: Cirratulidae; taxonRank: family; scientificNameAuthorship: Ryckholt, 1851; **Location:** waterBody: Pacific; stateProvince: Clarion Clipperton Zone; locality: UK Seabed Resources Ltd exploration area UK-1 Stratum B; verbatimLocality: UK1 Stratum B; maximumDepthInMeters: 4198; locationRemarks: Deployment EB03; at Station U4; from R/V Thomas G. Thompson Cruise no. TN319; verbatimLatitude: 12'34.28; verbatimLongitude: 116'36.63; decimalLatitude: 12.57133; decimalLongitude: -116.6105; geodeticDatum: WGS84; **Identification:** identifiedBy: Helena Wiklund | Lenka Neal | Thomas Dahlgren | Adrian Glover | Madeleine Brasier | Regan Drennan | Eva Stewart; dateIdentified: 2021-04-20; identificationRemarks: identified by DNA and morphology; **Event:** eventID: UK1_AB02_EB03; samplingProtocol: Brenke Epibenthic Sledge; eventDate: 2015-02-23; eventTime: 05:39; habitat: Abyssal plain; fieldNotes: Collected from epi net (on the epibenthic sledge); **Record Level:** language: en; institutionCode: NHMUK; collectionCode: ZOO; datasetName: ABYSSLINE; basisOfRecord: PreservedSpecimen
- **Type status:** Other material. **Occurrence:** catalogNumber: NHMUK ANEA 2023.339; recordNumber: NHM_0172; recordedBy: Adrian Glover | Helena Wiklund | Thomas Dahlgren | Magdalena Georgieva; individualCount: 1; preparations: specimen stored in 80% non-denatured ethanol aqueous solution | DNA voucher stored in buffer; otherCatalogNumbers: 0174127326; associatedSequences: OQ746483 (16S) | OQ746804 (18S) | OQ738504 (COI); occurrenceID: 957BB35B-29D6-59FC-92E1-741291447C68; **Taxon:** taxonConceptID: Cirratulidae (NHM_172); scientificName: Cirratulidae; kingdom: Animalia; phylum: Annelida; class: Polychaeta; order: Terebellida; family: Cirratulidae; taxonRank: family; scientificNameAuthorship: Ryckholt, 1851; **Location:** waterBody: Pacific; stateProvince: Clarion Clipperton Zone; locality: UK Seabed Resources Ltd exploration area UK-1 Stratum A; verbatimLocality: UK1 Stratum A; maximumDepthInMeters: 4082; locationRemarks: Deployment EB03; at Station D; from R/V Melville Cruise no. MV1313; verbatimLatitude: 13°56.089; verbatimLongitude: 116°33.011; decimalLatitude: 13.93482; decimalLongitude: -116.55018; geodeticDatum: WGS84; **Identification:** identifiedBy: Helena Wiklund | Lenka Neal | Thomas Dahlgren | Adrian Glover | Madeleine Brasier | Regan Drennan | Eva Stewart; dateIdentified: 2021-04-20; identificationRemarks: identified by DNA and morphology; **Event:** eventID: UK1_AB01_EB03; samplingProtocol: Brenke Epibenthic Sledge; eventDate: 2013-10-13; eventTime: 12:40; habitat: Abyssal plain; fieldNotes: Collected from epi net (on the epibenthic sledge); **Record Level:** language: en; institutionCode: NHMUK; collectionCode: ZOO; datasetName: ABYSSLINE; basisOfRecord: PreservedSpecimen

##### Distribution

Eastern Clarion-Clipperton Zone, central Pacific Ocean.

##### Diagnosis

**Figure 31.**
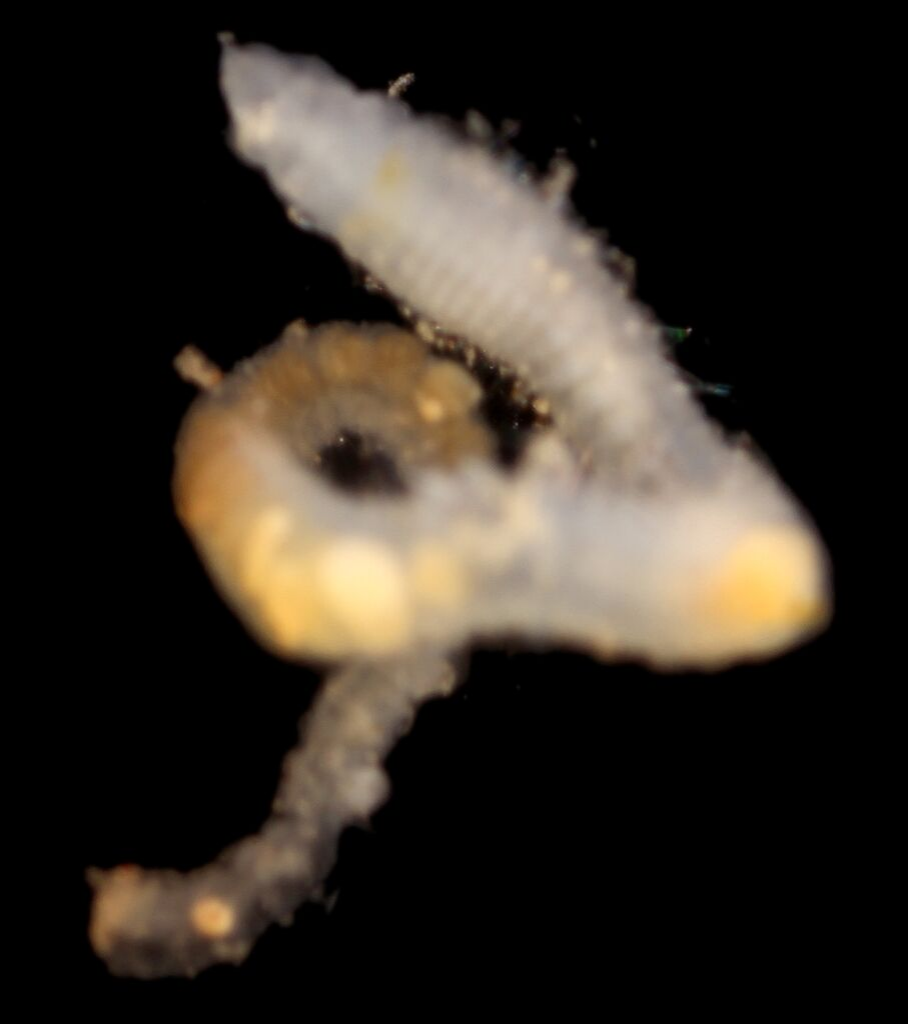
Cirratulidae sp. (NHM_172), anterior end of live specimen NHM_172.

Damaged specimens (Fig. 31) consistent with placement within family Cirratulidae, based on morphology and DNA.

#### Cirratulidae sp. (NHM_165)

3EB8B928-0CD7-5C6C-8C81-9DE8248EAACE

##### Materials

- **Type status:** Other material. **Occurrence:** catalogNumber: NHMUK ANEA 2023.348; recordNumber: NHM_0165; recordedBy: Adrian Glover | Helena Wiklund | Thomas Dahlgren | Magdalena Georgieva; individualCount: 1; preparations: specimen stored in 80% non-denatured ethanol aqueous solution | DNA voucher stored in buffer; otherCatalogNumbers: 0174127361; associatedSequences: OQ746481 (16S) | OQ746803 (18S) | OQ738503 (COI); occurrenceID: F675AF2E-B016-54ED-B577-64CB2B52489B; **Taxon:** taxonConceptID: Cirratulidae sp. (NHM_165); scientificName: Cirratulidae; kingdom: Animalia; phylum: Annelida; class: Polychaeta; order: Terebellida; family: Cirratulidae; taxonRank: family; scientificNameAuthorship: Ryckholt, 1851; **Location:** waterBody: Pacific; stateProvince: Clarion Clipperton Zone; locality: UK Seabed Resources Ltd exploration area UK-1 Stratum A; verbatimLocality: UK1 Stratum A; maximumDepthInMeters: 4084; locationRemarks: Deployment BC06; at Station D; from R/V Melville Cruise no. MV1313; verbatimLatitude: 13°57.794; verbatimLongitude: 116°34.093; decimalLatitude: 13.96323; decimalLongitude: -116.56822; geodeticDatum: WGS84; **Identification:** identifiedBy: Helena Wiklund | Lenka Neal | Thomas Dahlgren | Adrian Glover | Madeleine Brasier | Regan Drennan | Eva Stewart; dateIdentified: 2021-04-20; identificationRemarks: identified by DNA and morphology; **Event:** eventID: UK1_AB01_BC06; samplingProtocol: USNEL Box Core; eventDate: 2013-10-12; eventTime: 23:01:00; habitat: Abyssal plain; fieldNotes: Collected from 0-2 cm layer of box core using a 300 micron sieve; **Record Level:** language: en; institutionCode: NHMUK; collectionCode: ZOO; datasetName: ABYSSLINE; basisOfRecord: PreservedSpecimen

##### Distribution

Eastern Clarion-Clipperton Zone, central Pacific Ocean.

##### Diagnosis

**Figure 32.**
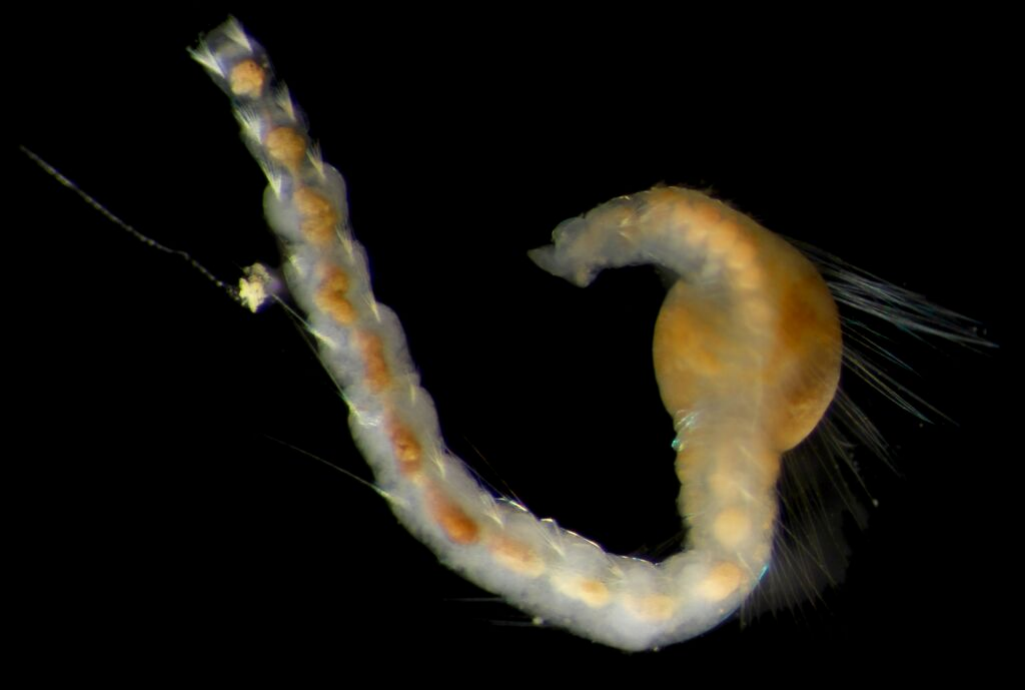
Cirratulidae sp. (NHM_165), anterior end of live specimen NHM_165.

Damaged specimen (Fig. 32) consistent with placement within family Cirratulidae, based on morphology and DNA.

#### Cirratulidae sp. (NHM_340)

FE12A152-0A28-5D95-B4BE-08C4F89D9B81

##### Materials

- **Type status:** Other material. **Occurrence:** catalogNumber: NHMUK ANEA 2023.360; recordNumber: NHM_0911; recordedBy: Adrian Glover | Helena Wiklund | Thomas Dahlgren | Madeleine Brasier; individualCount: 1; preparations: specimen stored in 80% non-denatured ethanol aqueous solution | DNA voucher stored in buffer; otherCatalogNumbers: 0174126788; associatedSequences: OQ746576 (16S); occurrenceID: 3FC55FCC-FB4F-5192-8F37-E6785857B365; **Taxon:** taxonConceptID: Cirratulidae sp. (NHM_340); scientificName: Cirratulidae; kingdom: Animalia; phylum: Annelida; class: Polychaeta; order: Terebellida; family: Cirratulidae; taxonRank: family; scientificNameAuthorship: Ryckholt, 1851; **Location:** waterBody: Pacific; stateProvince: Clarion Clipperton Zone; locality: UK Seabed Resources Ltd exploration area UK-1 Stratum B; verbatimLocality: UK1 Stratum B; maximumDepthInMeters: 4198; locationRemarks: Deployment EB03; at Station U4; from R/V Thomas G. Thompson Cruise no. TN319; verbatimLatitude: 12'34.28; verbatimLongitude: 116'36.63; decimalLatitude: 12.57133; decimalLongitude: -116.6105; geodeticDatum: WGS84; **Identification:** identifiedBy: Helena Wiklund | Lenka Neal | Thomas Dahlgren | Adrian Glover | Madeleine Brasier | Regan Drennan | Eva Stewart; dateIdentified: 2021-04-20; identificationRemarks: identified by DNA and morphology; **Event:** eventID: UK1_AB02_EB03; samplingProtocol: Brenke Epibenthic Sledge; eventDate: 2015-02-23; eventTime: 05:39; habitat: Abyssal plain; fieldNotes: Collected from epi net (on the epibenthic sledge); **Record Level:** language: en; institutionCode: NHMUK; collectionCode: ZOO; datasetName: ABYSSLINE; basisOfRecord: PreservedSpecimen
- **Type status:** Other material. **Occurrence:** catalogNumber: NHMUK ANEA 2023.357; recordNumber: NHM_0184; recordedBy: Adrian Glover | Helena Wiklund | Thomas Dahlgren | Magdalena Georgieva; individualCount: 1; preparations: specimen stored in 80% non-denatured ethanol aqueous solution | DNA voucher stored in buffer; otherCatalogNumbers: 0174127302; associatedSequences: OQ746806 (18S); occurrenceID: 3FBCA88D-028A-5A31-9CE3-7CB2BDBCA756; **Taxon:** taxonConceptID: Cirratulidae sp. (NHM_340); scientificName: Cirratulidae; kingdom: Animalia; phylum: Annelida; class: Polychaeta; order: Terebellida; family: Cirratulidae; taxonRank: family; scientificNameAuthorship: Ryckholt, 1851; **Location:** waterBody: Pacific; stateProvince: Clarion Clipperton Zone; locality: UK Seabed Resources Ltd exploration area UK-1 Stratum A; verbatimLocality: UK1 Stratum A; maximumDepthInMeters: 4082; locationRemarks: Deployment EB03; at Station D; from R/V Melville Cruise no. MV1313; verbatimLatitude: 13°56.089; verbatimLongitude: 116°33.011; decimalLatitude: 13.93482; decimalLongitude: -116.55018; geodeticDatum: WGS84; **Identification:** identifiedBy: Helena Wiklund | Lenka Neal | Thomas Dahlgren | Adrian Glover | Madeleine Brasier | Regan Drennan | Eva Stewart; dateIdentified: 2021-04-20; identificationRemarks: identified by DNA and morphology; **Event:** eventID: UK1_AB01_EB03; samplingProtocol: Brenke Epibenthic Sledge; eventDate: 2013-10-13; eventTime: 12:40; habitat: Abyssal plain; fieldNotes: Collected from epi net (on the epibenthic sledge); **Record Level:** language: en; institutionCode: NHMUK; collectionCode: ZOO; datasetName: ABYSSLINE; basisOfRecord: PreservedSpecimen
- **Type status:** Other material. **Occurrence:** recordNumber: NHM_0403; recordedBy: Adrian Glover | Helena Wiklund | Thomas Dahlgren | Magdalena Georgieva; individualCount: 1; preparations: DNA voucher stored in buffer; otherCatalogNumbers: 0174127303; associatedSequences: OQ746508 (16S) | OQ746825 (18S); occurrenceID: 9FF5C1EE-9345-50F1-9F2B-D00BCCF5D7CB; **Taxon:** taxonConceptID: Cirratulidae sp. (NHM_340); scientificName: Cirratulidae; kingdom: Animalia; phylum: Annelida; class: Polychaeta; order: Terebellida; family: Cirratulidae; taxonRank: family; scientificNameAuthorship: Ryckholt, 1851; **Location:** waterBody: Pacific; stateProvince: Clarion Clipperton Zone; locality: UK Seabed Resources Ltd exploration area UK-1 Stratum A; verbatimLocality: UK1 Stratum A; maximumDepthInMeters: 4500; locationRemarks: Deployment BC12; at Station K; from R/V Melville Cruise no. MV1313; decimalLatitude: 13.86328; decimalLongitude: -116.54885; geodeticDatum: WGS84; **Identification:** identifiedBy: Helena Wiklund | Lenka Neal | Thomas Dahlgren | Adrian Glover | Madeleine Brasier | Regan Drennan | Eva Stewart; dateIdentified: 2021-04-20; identificationRemarks: identified by DNA and morphology; **Event:** eventID: UK1_AB01_BC12; samplingProtocol: USNEL Box Core; eventDate: 2013-10-20; eventTime: 03:39; habitat: Abyssal plain; fieldNotes: Collected from 0-2 cm layer of box core using a 300 micron sieve; **Record Level:** language: en; institutionCode: NHMUK; collectionCode: ZOO; datasetName: ABYSSLINE; basisOfRecord: PreservedSpecimen
- **Type status:** Other material. **Occurrence:** catalogNumber: NHMUK ANEA 2023.359; recordNumber: NHM_0432; recordedBy: Adrian Glover | Helena Wiklund | Thomas Dahlgren | Magdalena Georgieva; individualCount: 1; preparations: specimen stored in 80% non-denatured ethanol aqueous solution | DNA voucher stored in buffer; otherCatalogNumbers: 0174127301; associatedSequences: OQ746517 (16S) | OQ746833 (18S); occurrenceID: 151A87F8-AA16-585C-B2D9-7E17E8C45C39; **Taxon:** taxonConceptID: Cirratulidae sp. (NHM_340); scientificName: Cirratulidae; kingdom: Animalia; phylum: Annelida; class: Polychaeta; order: Terebellida; family: Cirratulidae; taxonRank: family; scientificNameAuthorship: Ryckholt, 1851; **Location:** waterBody: Pacific; stateProvince: Clarion Clipperton Zone; locality: UK Seabed Resources Ltd exploration area UK-1 Stratum A; verbatimLocality: UK1 Stratum A; maximumDepthInMeters: 4011; locationRemarks: Deployment RV06; at Station K; from R/V Melville Cruise no. MV1313; decimalLatitude: 13.86367; decimalLongitude: -116.54432; geodeticDatum: WGS84; **Identification:** identifiedBy: Helena Wiklund | Lenka Neal | Thomas Dahlgren | Adrian Glover | Madeleine Brasier | Regan Drennan | Eva Stewart; dateIdentified: 2021-04-20; identificationRemarks: identified by DNA and morphology; **Event:** eventID: UK1_AB01_RV06; samplingProtocol: Remotely Operated Vehicle; eventDate: 2013-10-20; eventTime: 10:32; habitat: Abyssal plain; **Record Level:** language: en; institutionCode: NHMUK; collectionCode: ZOO; datasetName: ABYSSLINE; basisOfRecord: PreservedSpecimen
- **Type status:** Other material. **Occurrence:** catalogNumber: NHMUK ANEA 2023.358; recordNumber: NHM_0340; recordedBy: Adrian Glover | Helena Wiklund | Thomas Dahlgren | Magdalena Georgieva; individualCount: 1; preparations: specimen stored in 80% non-denatured ethanol aqueous solution | DNA voucher stored in buffer; otherCatalogNumbers: 0174127360; associatedSequences: OQ746499 (16S) | OQ746817 (18S); occurrenceID: AB0DE221-4514-5D34-979F-D3ABE4D8D7D8; **Taxon:** taxonConceptID: Cirratulidae sp. (NHM_340); scientificName: Cirratulidae; kingdom: Animalia; phylum: Annelida; class: Polychaeta; order: Terebellida; family: Cirratulidae; taxonRank: family; scientificNameAuthorship: Ryckholt, 1851; **Location:** waterBody: Pacific; stateProvince: Clarion Clipperton Zone; locality: UK Seabed Resources Ltd exploration area UK-1 Stratum A; verbatimLocality: UK1 Stratum A; maximumDepthInMeters: 4111; locationRemarks: Deployment MC08; at Station I; from R/V Melville Cruise no. MV1313; verbatimLatitude: 13°45.700; verbatimLongitude: 116°27.620; decimalLatitude: 13.76167; decimalLongitude: -116.46033; geodeticDatum: WGS84; **Identification:** identifiedBy: Helena Wiklund | Lenka Neal | Thomas Dahlgren | Adrian Glover | Madeleine Brasier | Regan Drennan | Eva Stewart; dateIdentified: 2021-04-20; identificationRemarks: identified by DNA and morphology; **Event:** eventID: UK1_AB01_MC08; samplingProtocol: Multi Corer; eventDate: 2013-10-18; eventTime: 15:54; habitat: Abyssal plain; **Record Level:** language: en; institutionCode: NHMUK; collectionCode: ZOO; datasetName: ABYSSLINE; basisOfRecord: PreservedSpecimen

##### Distribution

Eastern Clarion-Clipperton Zone, central Pacific Ocean.

##### Diagnosis

**Figure 33.**
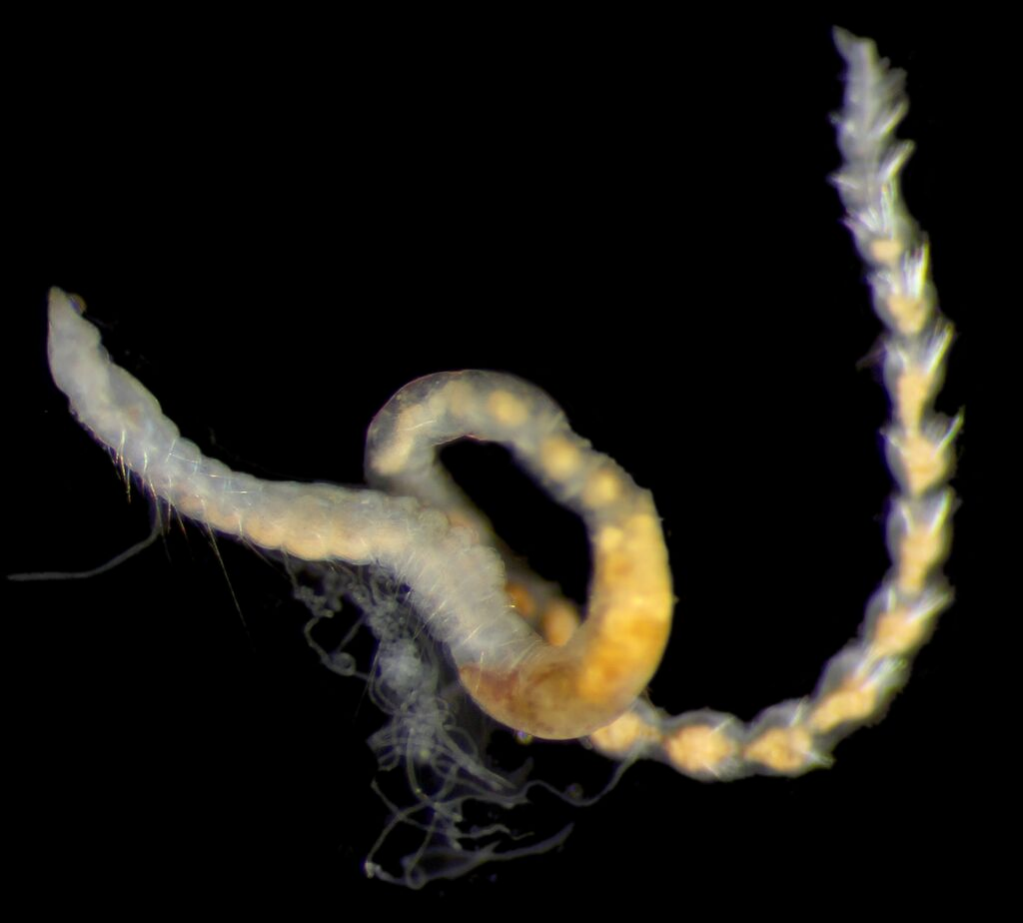
Cirratulidae sp. (NHM_340), live specimen NHM_340 in lateral view.

Complete (Fig. 33) and damaged specimens consistent with placement within family Cirratulidae, based on morphology and DNA.

#### Cirratulidae sp. (NHM_269)

56616B1D-4457-5179-945C-B15EA2EA3EB5

##### Materials

- **Type status:** Other material. **Occurrence:** catalogNumber: NHMUK ANEA 2023.356; recordNumber: NHM_1652; recordedBy: Adrian Glover | Helena Wiklund | Thomas Dahlgren | Madeleine Brasier; individualCount: 1; preparations: specimen stored in 80% non-denatured ethanol aqueous solution | DNA voucher stored in buffer; otherCatalogNumbers: 0174126218; associatedSequences: OQ746700 (16S) | OQ738595 (COI); occurrenceID: C511CA51-DCD7-5E5A-8E66-27A63B6CB7C9; **Taxon:** taxonConceptID: Cirratulidae sp. (NHM_269); scientificName: Cirratulidae; kingdom: Animalia; phylum: Annelida; class: Polychaeta; order: Terebellida; family: Cirratulidae; taxonRank: family; scientificNameAuthorship: Ryckholt, 1851; **Location:** waterBody: Pacific; stateProvince: Clarion Clipperton Zone; locality: UK Seabed Resources Ltd exploration area UK-1 Stratum B; verbatimLocality: UK1 Stratum B; maximumDepthInMeters: 4233; locationRemarks: Deployment EB09; at Station U1; from R/V Thomas G. Thompson Cruise no. TN319; verbatimLatitude: 12'21.81; verbatimLongitude: 116'40.86; decimalLatitude: 12.3635; decimalLongitude: -116.681; geodeticDatum: WGS84; **Identification:** identifiedBy: Helena Wiklund | Lenka Neal | Thomas Dahlgren | Adrian Glover | Madeleine Brasier | Regan Drennan | Eva Stewart; dateIdentified: 2021-04-20; identificationRemarks: identified by DNA and morphology; **Event:** eventID: UK1_AB02_EB09; samplingProtocol: Brenke Epibenthic Sledge; eventDate: 2015-03-10; eventTime: 10:46; habitat: Abyssal plain; fieldNotes: Collected from epi net (on the epibenthic sledge); **Record Level:** language: en; institutionCode: NHMUK; collectionCode: ZOO; datasetName: ABYSSLINE; basisOfRecord: PreservedSpecimen
- **Type status:** Other material. **Occurrence:** catalogNumber: NHMUK ANEA 2023.355; recordNumber: NHM_0460; recordedBy: Adrian Glover | Helena Wiklund | Thomas Dahlgren | Magdalena Georgieva; individualCount: 1; preparations: specimen stored in 80% non-denatured ethanol aqueous solution | DNA voucher stored in buffer; otherCatalogNumbers: 0174127366; associatedSequences: OQ746522 (16S) | OQ746838 (18S) | OQ738522 (COI); occurrenceID: 79C78F68-E7ED-5E7C-9C95-ADF37F299F8D; **Taxon:** taxonConceptID: Cirratulidae sp. (NHM_269); scientificName: Cirratulidae; kingdom: Animalia; phylum: Annelida; class: Polychaeta; order: Terebellida; family: Cirratulidae; taxonRank: family; scientificNameAuthorship: Ryckholt, 1851; **Location:** waterBody: Pacific; stateProvince: Clarion Clipperton Zone; locality: UK Seabed Resources Ltd exploration area UK-1 Stratum A; verbatimLocality: UK1 Stratum A; maximumDepthInMeters: 4163; locationRemarks: Deployment BC13; at Station J; from R/V Melville Cruise no. MV1313; verbatimLatitude: 13°54.099; verbatimLongitude: 116°35.400; decimalLatitude: 13.90165; decimalLongitude: -116.59; geodeticDatum: WGS84; **Identification:** identifiedBy: Helena Wiklund | Lenka Neal | Thomas Dahlgren | Adrian Glover | Madeleine Brasier | Regan Drennan | Eva Stewart; dateIdentified: 2021-04-20; identificationRemarks: identified by DNA and morphology; **Event:** eventID: UK1_AB01_BC13; samplingProtocol: USNEL Box Core; eventDate: 2013-10-21; eventTime: 13:27; habitat: Abyssal plain; fieldNotes: Collected from 0-2 cm layer of box core using a 300 micron sieve; **Record Level:** language: en; institutionCode: NHMUK; collectionCode: ZOO; datasetName: ABYSSLINE; basisOfRecord: PreservedSpecimen
- **Type status:** Other material. **Occurrence:** catalogNumber: NHMUK ANEA 2023.354; recordNumber: NHM_0269; recordedBy: Adrian Glover | Helena Wiklund | Thomas Dahlgren | Magdalena Georgieva; individualCount: 1; preparations: specimen stored in 80% non-denatured ethanol aqueous solution | DNA voucher stored in buffer; otherCatalogNumbers: 0174127320; associatedSequences: OQ746491 (16S) | OQ746810 (18S) | OQ738509 (COI); occurrenceID: CBB19F81-1000-562E-B34C-2B7D3FBEF4F9; **Taxon:** taxonConceptID: Cirratulidae sp. (NHM_269); scientificName: Cirratulidae; kingdom: Animalia; phylum: Annelida; class: Polychaeta; order: Terebellida; family: Cirratulidae; taxonRank: family; scientificNameAuthorship: Ryckholt, 1851; **Location:** waterBody: Pacific; stateProvince: Clarion Clipperton Zone; locality: UK Seabed Resources Ltd exploration area UK-1 Stratum A; verbatimLocality: UK1 Stratum A; maximumDepthInMeters: 4128; locationRemarks: Deployment EB04; at Station G-I; from R/V Melville Cruise no. MV1313; verbatimLatitude: 13°45.21N; verbatimLongitude: 116°29.12W; decimalLatitude: 13.75583; decimalLongitude: -116.48667; geodeticDatum: WGS84; **Identification:** identifiedBy: Helena Wiklund | Lenka Neal | Thomas Dahlgren | Adrian Glover | Madeleine Brasier | Regan Drennan | Eva Stewart; dateIdentified: 2021-04-20; identificationRemarks: identified by DNA and morphology; **Event:** eventID: UK1_AB01_EB04; samplingProtocol: Brenke Epibenthic Sledge; eventDate: 2013-10-17; eventTime: 01:50; habitat: Abyssal plain; fieldNotes: Collected from epi net (on the epibenthic sledge); **Record Level:** language: en; institutionCode: NHMUK; collectionCode: ZOO; datasetName: ABYSSLINE; basisOfRecord: PreservedSpecimen
- **Type status:** Other material. **Occurrence:** recordNumber: NHM_0305; recordedBy: Adrian Glover | Helena Wiklund | Thomas Dahlgren | Magdalena Georgieva; individualCount: 1; preparations: DNA voucher stored in buffer; otherCatalogNumbers: 0174127386; associatedSequences: OQ746495 (16S) | OQ746814 (18S) | OQ738513 (COI); occurrenceID: 97124115-6305-55D9-902D-141A70D7D67C; **Taxon:** taxonConceptID: Cirratulidae sp. (NHM_269); scientificName: Cirratulidae; kingdom: Animalia; phylum: Annelida; class: Polychaeta; order: Terebellida; family: Cirratulidae; taxonRank: family; scientificNameAuthorship: Ryckholt, 1851; **Location:** waterBody: Pacific; stateProvince: Clarion Clipperton Zone; locality: UK Seabed Resources Ltd exploration area UK-1 Stratum A; verbatimLocality: UK1 Stratum A; maximumDepthInMeters: 4110; locationRemarks: Deployment BC09; at Station G; from R/V Melville Cruise no. MV1313; verbatimLatitude: 13°45.726; verbatimLongitude: 116°27.825; decimalLatitude: 13.7621; decimalLongitude: -116.46375; geodeticDatum: WGS84; **Identification:** identifiedBy: Helena Wiklund | Lenka Neal | Thomas Dahlgren | Adrian Glover | Madeleine Brasier | Regan Drennan | Eva Stewart; dateIdentified: 2021-04-20; identificationRemarks: identified by DNA and morphology; **Event:** eventID: UK1_AB01_BC09; samplingProtocol: USNEL Box Core; eventDate: 2013-10-17; eventTime: 13:40; habitat: Abyssal plain; fieldNotes: Collected from 0-2 cm layer of box core using a 300 micron sieve; **Record Level:** language: en; institutionCode: NHMUK; collectionCode: ZOO; datasetName: ABYSSLINE; basisOfRecord: PreservedSpecimen

##### Distribution

Eastern Clarion-Clipperton Zone, central Pacific Ocean.

##### Diagnosis

**Figure 34.**
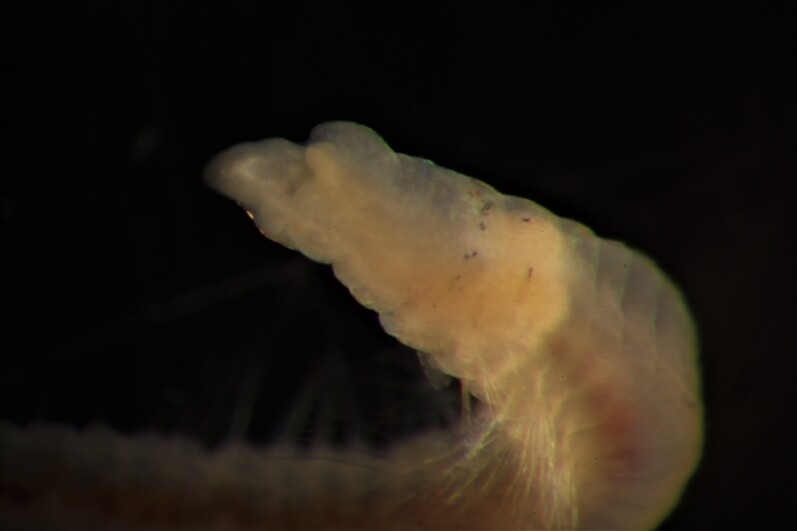
Cirratulidae sp. (NHM_269), anterior end of live specimen NHM_1652 in ventrolateral view.

Damaged specimens (Fig. 34) consistent with placement within family Cirratulidae, based on morphology and DNA.

### Flabelligeridae de Saint-Joseph, 1894

#### Flabelligeridae sp. (NHM_555)

33A5CEBF-7377-594D-9B38-57784660D163

##### Materials

- **Type status:** Other material. **Occurrence:** catalogNumber: NHMUK ANEA 2023.378; recordNumber: NHM_0555; recordedBy: Adrian Glover | Helena Wiklund | Thomas Dahlgren | Madeleine Brasier; individualCount: 1; preparations: specimen stored in 80% non-denatured ethanol aqueous solution | DNA voucher stored in buffer; otherCatalogNumbers: 0174126722; associatedSequences: OQ746527 (16S) | OQ738526 (COI); occurrenceID: A46BF556-5580-514D-BB28-98664A740171; **Taxon:** taxonConceptID: Flabelligeridae sp. (NHM_555); scientificName: Flabelligeridae; kingdom: Animalia; phylum: Annelida; class: Polychaeta; order: Terebellida; family: Flabelligeridae; taxonRank: family; scientificNameAuthorship: de Saint-Joseph, 1894; **Location:** waterBody: Pacific; stateProvince: Clarion Clipperton Zone; locality: UK Seabed Resources Ltd exploration area UK-1 Stratum B; verbatimLocality: UK1 Stratum B; maximumDepthInMeters: 4202; locationRemarks: Deployment EB01; at Station U2; from R/V Thomas G. Thompson Cruise no. TN319; verbatimLatitude: 12'23.17456; verbatimLongitude: 116'32.92021; decimalLatitude: 12.38624; decimalLongitude: -116.54867; geodeticDatum: WGS84; **Identification:** identifiedBy: Helena Wiklund | Lenka Neal | Thomas Dahlgren | Adrian Glover | Madeleine Brasier | Regan Drennan | Eva Stewart; dateIdentified: 2021-04-20; identificationRemarks: identified by DNA and morphology; **Event:** eventID: UK1_AB02_EB01; samplingProtocol: Brenke Epibenthic Sledge; eventDate: 2015-02-17; eventTime: 05:15; habitat: Abyssal plain; fieldNotes: Collected from epi net (on the epibenthic sledge); **Record Level:** language: en; institutionCode: NHMUK; collectionCode: ZOO; datasetName: ABYSSLINE; basisOfRecord: PreservedSpecimen

##### Distribution

Eastern Clarion-Clipperton Zone, central Pacific Ocean.

##### Diagnosis

**Figure 35.**
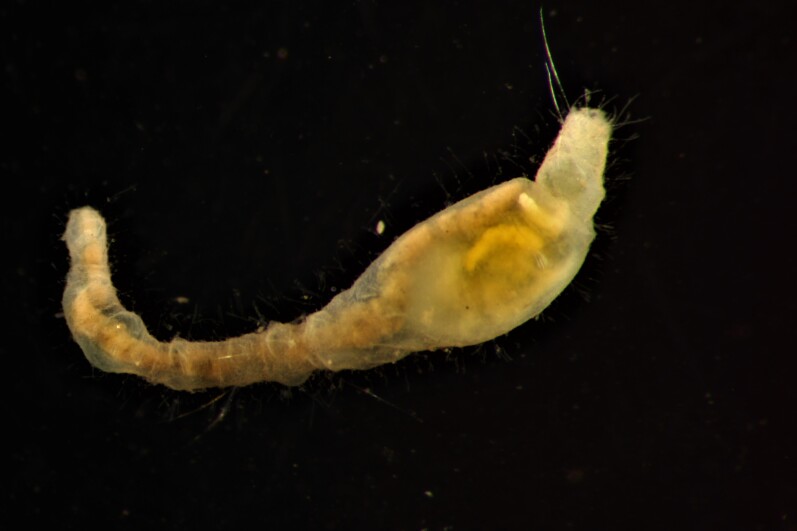
Flabelligeridae sp. (NHM_555), live specimen NHM_555.

Damaged specimen (Fig. 35) consistent with placement within family Flabelligeridae, based on morphology and DNA.

#### Flabelligeridae sp. (NHM_630A)

BEDBBC88-DA93-521F-A1E3-DCCB50320598

##### Materials

- **Type status:** Other material. **Occurrence:** recordNumber: NHM_0630A; recordedBy: Adrian Glover | Helena Wiklund | Thomas Dahlgren | Madeleine Brasier; individualCount: 1; preparations: Tissue voucher stored in 80% non-denatured ethanol aqueous solution | DNA voucher stored in buffer; otherCatalogNumbers: 0109405359 | 0174126598; associatedSequences: OQ746536 (16S) | OQ746848 (18S) | OQ738530 (COI); occurrenceID: A038CEEE-DD5D-5CB5-9E2B-BBE04CA01D22; **Taxon:** taxonConceptID: Flabelligeridae sp. (NHM_630A); scientificName: Flabelligeridae; kingdom: Animalia; phylum: Annelida; class: Polychaeta; order: Terebellida; family: Flabelligeridae; taxonRank: family; scientificNameAuthorship: de Saint-Joseph, 1894; **Location:** waterBody: Pacific; stateProvince: Clarion Clipperton Zone; locality: UK Seabed Resources Ltd exploration area UK-1 Stratum B; verbatimLocality: UK1 Stratum B; maximumDepthInMeters: 4202; locationRemarks: Deployment EB01; at Station U2; from R/V Thomas G. Thompson Cruise no. TN319; verbatimLatitude: 12'23.17456; verbatimLongitude: 116'32.92021; decimalLatitude: 12.38624; decimalLongitude: -116.54867; geodeticDatum: WGS84; **Identification:** identifiedBy: Helena Wiklund | Lenka Neal | Thomas Dahlgren | Adrian Glover | Madeleine Brasier | Regan Drennan | Eva Stewart; dateIdentified: 2021-04-20; identificationRemarks: identified by DNA and morphology; **Event:** eventID: UK1_AB02_EB01; samplingProtocol: Brenke Epibenthic Sledge; eventDate: 2015-02-17; eventTime: 05:15; habitat: Abyssal plain; fieldNotes: Collected from epi net (on the epibenthic sledge); **Record Level:** language: en; institutionCode: NHMUK; collectionCode: ZOO; datasetName: ABYSSLINE; basisOfRecord: PreservedSpecimen

##### Distribution

Eastern Clarion-Clipperton Zone, central Pacific Ocean.

##### Diagnosis

**Figure 36.**
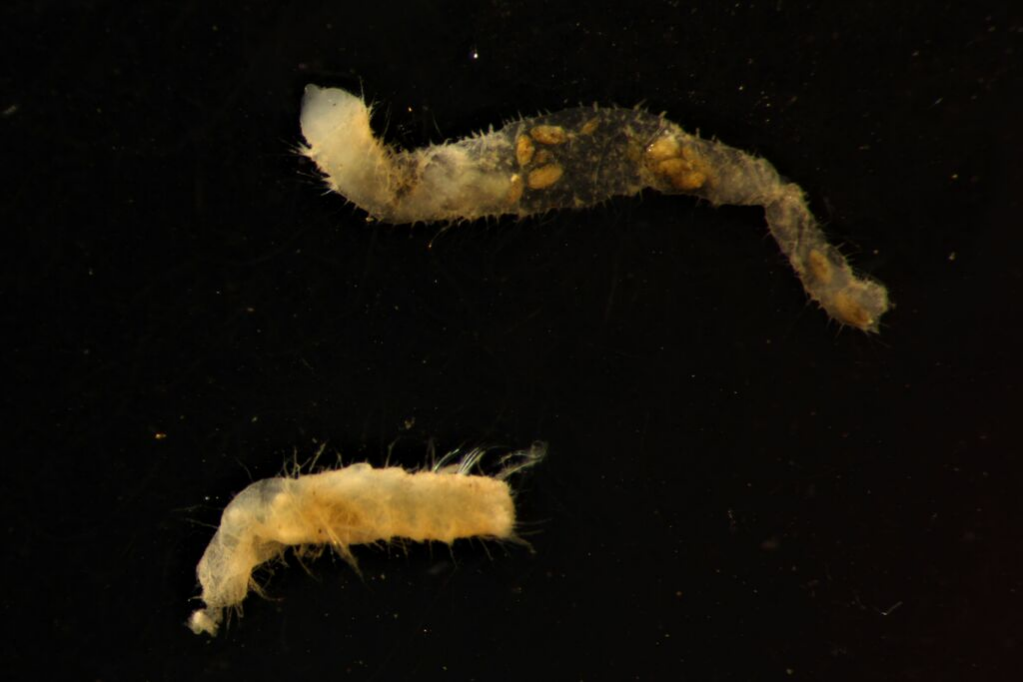
Flabelligeridae sp. (NHM_630A), fragmented live specimen NHM_630A.

Fragmented specimen (Fig. 36) consistent with placement within family Flabelligeridae, based on morphology and DNA.

#### Flabelligeridae sp. (NHM_738)

60669F98-A522-5E81-AFFC-84B8EB95DC6B

##### Materials

- **Type status:** Other material. **Occurrence:** catalogNumber: NHMUK ANEA 2023.380; recordNumber: NHM_1157; recordedBy: Adrian Glover | Helena Wiklund | Thomas Dahlgren | Madeleine Brasier; individualCount: 1; preparations: specimen stored in 80% non-denatured ethanol aqueous solution | DNA voucher stored in buffer; otherCatalogNumbers: 0174126783; associatedSequences: OQ746626 (16S); occurrenceID: F8E5E6FB-486C-55ED-AA3F-1EE13027E262; **Taxon:** taxonConceptID: Flabelligeridae sp. (NHM_738); scientificName: Flabelligeridae; kingdom: Animalia; phylum: Annelida; class: Polychaeta; order: Terebellida; family: Flabelligeridae; taxonRank: family; scientificNameAuthorship: de Saint-Joseph, 1894; **Location:** waterBody: Pacific; stateProvince: Clarion Clipperton Zone; locality: Ocean Mineral Singapore exploration claim Stratum A; verbatimLocality: OMS Stratum A; maximumDepthInMeters: 4100; locationRemarks: Deployment EB05; at Station S2; from R/V Thomas G. Thompson Cruise no. TN319; verbatimLatitude: 12'06.93; verbatimLongitude: 117'09.87; decimalLatitude: 12.1155; decimalLongitude: -117.1645; geodeticDatum: WGS84; **Identification:** identifiedBy: Helena Wiklund | Lenka Neal | Thomas Dahlgren | Adrian Glover | Madeleine Brasier | Regan Drennan | Eva Stewart; dateIdentified: 2021-04-20; identificationRemarks: identified by DNA and morphology; **Event:** eventID: OMS1_AB02_EB05; samplingProtocol: Brenke Epibenthic Sledge; eventDate: 2015-02-26; eventTime: 21:29; habitat: Abyssal plain; fieldNotes: Collected from epi net (on the epibenthic sledge); **Record Level:** language: en; institutionCode: NHMUK; collectionCode: ZOO; datasetName: ABYSSLINE; basisOfRecord: PreservedSpecimen
- **Type status:** Other material. **Occurrence:** catalogNumber: NHMUK ANEA 2023.381; recordNumber: NHM_1158; recordedBy: Adrian Glover | Helena Wiklund | Thomas Dahlgren | Madeleine Brasier; individualCount: 1; preparations: specimen stored in 80% non-denatured ethanol aqueous solution | DNA voucher stored in buffer; otherCatalogNumbers: 0174126776; associatedSequences: OQ746627 (16S) | OQ746876 (18S) | OQ738562 (COI); occurrenceID: 43E0796C-723E-5CC4-8FBB-15FE98659B69; **Taxon:** taxonConceptID: Flabelligeridae sp. (NHM_738); scientificName: Flabelligeridae; kingdom: Animalia; phylum: Annelida; class: Polychaeta; order: Terebellida; family: Flabelligeridae; taxonRank: family; scientificNameAuthorship: de Saint-Joseph, 1894; **Location:** waterBody: Pacific; stateProvince: Clarion Clipperton Zone; locality: Ocean Mineral Singapore exploration claim Stratum A; verbatimLocality: OMS Stratum A; maximumDepthInMeters: 4100; locationRemarks: Deployment EB05; at Station S2; from R/V Thomas G. Thompson Cruise no. TN319; verbatimLatitude: 12'06.93; verbatimLongitude: 117'09.87; decimalLatitude: 12.1155; decimalLongitude: -117.1645; geodeticDatum: WGS84; **Identification:** identifiedBy: Helena Wiklund | Lenka Neal | Thomas Dahlgren | Adrian Glover | Madeleine Brasier | Regan Drennan | Eva Stewart; dateIdentified: 2021-04-20; identificationRemarks: identified by DNA and morphology; **Event:** eventID: OMS1_AB02_EB05; samplingProtocol: Brenke Epibenthic Sledge; eventDate: 2015-02-26; eventTime: 21:29; habitat: Abyssal plain; fieldNotes: Collected from epi net (on the epibenthic sledge); **Record Level:** language: en; institutionCode: NHMUK; collectionCode: ZOO; datasetName: ABYSSLINE; basisOfRecord: PreservedSpecimen
- **Type status:** Other material. **Occurrence:** catalogNumber: NHMUK ANEA 2023.379; recordNumber: NHM_0738; recordedBy: Adrian Glover | Helena Wiklund | Thomas Dahlgren | Madeleine Brasier; individualCount: 1; preparations: specimen stored in 80% non-denatured ethanol aqueous solution | DNA voucher stored in buffer; otherCatalogNumbers: 0174126732; associatedSequences: OQ746546 (16S); occurrenceID: 6CB261ED-082E-5DF0-8815-4213709237FB; **Taxon:** taxonConceptID: Flabelligeridae sp. (NHM_738); scientificName: Flabelligeridae; kingdom: Animalia; phylum: Annelida; class: Polychaeta; order: Terebellida; family: Flabelligeridae; taxonRank: family; scientificNameAuthorship: de Saint-Joseph, 1894; **Location:** waterBody: Pacific; stateProvince: Clarion Clipperton Zone; locality: UK Seabed Resources Ltd exploration area UK-1 Stratum B; verbatimLocality: UK1 Stratum B; maximumDepthInMeters: 4425; locationRemarks: Deployment EB02; at Station U5; from R/V Thomas G. Thompson Cruise no. TN319; verbatimLatitude: 12'32.23; verbatimLongitude: 116'36.25; decimalLatitude: 12.53717; decimalLongitude: -116.60417; geodeticDatum: WGS84; **Identification:** identifiedBy: Helena Wiklund | Lenka Neal | Thomas Dahlgren | Adrian Glover | Madeleine Brasier | Regan Drennan | Eva Stewart; dateIdentified: 2021-04-20; identificationRemarks: identified by DNA and morphology; **Event:** eventID: UK1_AB02_EB02; samplingProtocol: Brenke Epibenthic Sledge; eventDate: 2015-02-20; eventTime: 06:24; habitat: Abyssal plain; fieldNotes: Collected from epi net (on the epibenthic sledge); **Record Level:** language: en; institutionCode: NHMUK; collectionCode: ZOO; datasetName: ABYSSLINE; basisOfRecord: PreservedSpecimen

##### Distribution

Eastern Clarion-Clipperton Zone, central Pacific Ocean.

##### Diagnosis

**Figure 37.**
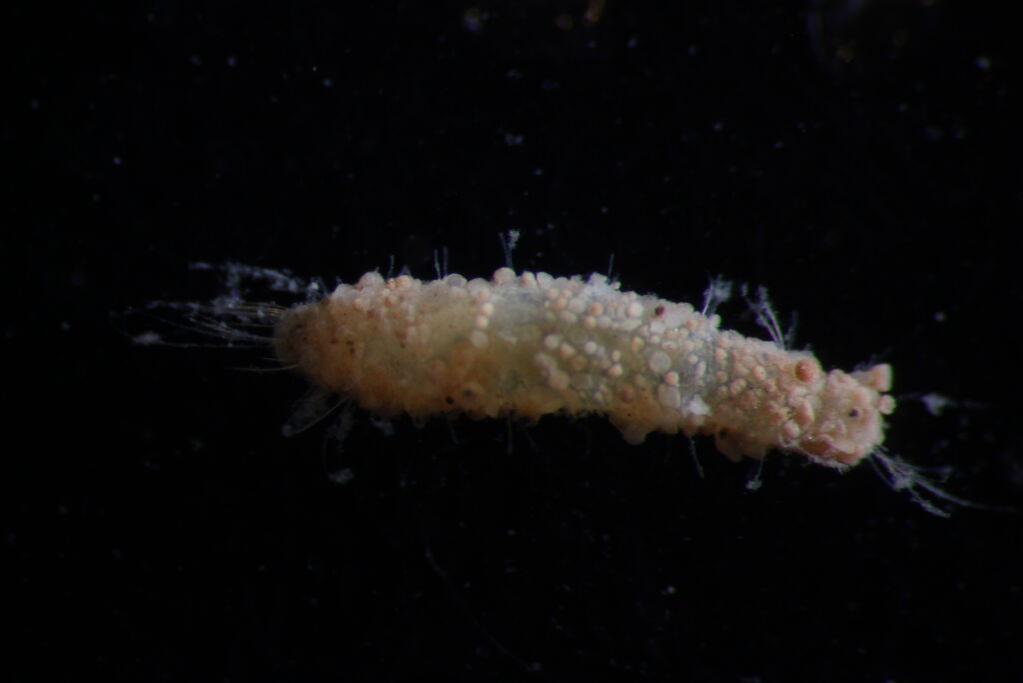
Flabelligeridae sp. (NHM_738), anterior fragment of live specimen NHM_738.

Damaged specimens (Fig. 37) consistent with placement within family Flabelligeridae, based on morphology and DNA.

#### Flabelligeridae sp. (NHM_955)

EC2F01F3-635C-52E0-AE6E-70146F1F4557

##### Materials

- **Type status:** Other material. **Occurrence:** catalogNumber: NHMUK ANEA 2023.384; recordNumber: NHM_1300; recordedBy: Adrian Glover | Helena Wiklund | Thomas Dahlgren | Madeleine Brasier; individualCount: 1; preparations: specimen stored in 80% non-denatured ethanol aqueous solution | DNA voucher stored in buffer; otherCatalogNumbers: 0174126802; associatedSequences: OQ746653 (16S); occurrenceID: 6E5706B1-5A36-52CE-ADC2-B69545A5A27A; **Taxon:** taxonConceptID: Flabelligeridae sp. (NHM_955); scientificName: Flabelligeridae; kingdom: Animalia; phylum: Annelida; class: Polychaeta; order: Terebellida; family: Flabelligeridae; taxonRank: family; scientificNameAuthorship: de Saint-Joseph, 1894; **Location:** waterBody: Pacific; stateProvince: Clarion Clipperton Zone; locality: Ocean Mineral Singapore exploration claim Stratum A; verbatimLocality: OMS Stratum A; maximumDepthInMeters: 4302; locationRemarks: Deployment EB06; at Station S5; from R/V Thomas G. Thompson Cruise no. TN319; verbatimLatitude: 12'15.44; verbatimLongitude: 117'18.13; decimalLatitude: 12.25733; decimalLongitude: -117.30217; geodeticDatum: WGS84; **Identification:** identifiedBy: Helena Wiklund | Lenka Neal | Thomas Dahlgren | Adrian Glover | Madeleine Brasier | Regan Drennan | Eva Stewart; dateIdentified: 2021-04-20; identificationRemarks: identified by DNA and morphology; **Event:** eventID: OMS1_AB02_EB06; samplingProtocol: Brenke Epibenthic Sledge; eventDate: 2015-03-01; eventTime: 04:02; habitat: Abyssal plain; fieldNotes: Collected from epi net (on the epibenthic sledge); **Record Level:** language: en; institutionCode: NHMUK; collectionCode: ZOO; datasetName: ABYSSLINE; basisOfRecord: PreservedSpecimen
- **Type status:** Other material. **Occurrence:** catalogNumber: NHMUK ANEA 2023.385; recordNumber: NHM_1314; recordedBy: Adrian Glover | Helena Wiklund | Thomas Dahlgren | Madeleine Brasier; individualCount: 1; preparations: specimen stored in 80% non-denatured ethanol aqueous solution | DNA voucher stored in buffer; otherCatalogNumbers: 0174126753; associatedSequences: OQ746657 (16S) | OQ746888 (18S); occurrenceID: EA029561-EE52-513B-8E99-8F0C30FABC0C; **Taxon:** taxonConceptID: Flabelligeridae sp. (NHM_955); scientificName: Flabelligeridae; kingdom: Animalia; phylum: Annelida; class: Polychaeta; order: Terebellida; family: Flabelligeridae; taxonRank: family; scientificNameAuthorship: de Saint-Joseph, 1894; **Location:** waterBody: Pacific; stateProvince: Clarion Clipperton Zone; locality: Ocean Mineral Singapore exploration claim Stratum A; verbatimLocality: OMS Stratum A; maximumDepthInMeters: 4302; locationRemarks: Deployment EB06; at Station S5; from R/V Thomas G. Thompson Cruise no. TN319; verbatimLatitude: 12'15.44; verbatimLongitude: 117'18.13; decimalLatitude: 12.25733; decimalLongitude: -117.30217; geodeticDatum: WGS84; **Identification:** identifiedBy: Helena Wiklund | Lenka Neal | Thomas Dahlgren | Adrian Glover | Madeleine Brasier | Regan Drennan | Eva Stewart; dateIdentified: 2021-04-20; identificationRemarks: identified by DNA and morphology; **Event:** eventID: OMS1_AB02_EB06; samplingProtocol: Brenke Epibenthic Sledge; eventDate: 2015-03-01; eventTime: 04:02; habitat: Abyssal plain; fieldNotes: Collected from epi net (on the epibenthic sledge); **Record Level:** language: en; institutionCode: NHMUK; collectionCode: ZOO; datasetName: ABYSSLINE; basisOfRecord: PreservedSpecimen
- **Type status:** Other material. **Occurrence:** catalogNumber: NHMUK ANEA 2023.386; recordNumber: NHM_1347E; recordedBy: Adrian Glover | Helena Wiklund | Thomas Dahlgren | Madeleine Brasier; individualCount: 1; preparations: specimen stored in 80% non-denatured ethanol aqueous solution | DNA voucher stored in buffer; otherCatalogNumbers: 0174126288; associatedSequences: OQ746665 (16S); occurrenceID: 9E399991-6BF4-55A8-A427-52299B077484; **Taxon:** taxonConceptID: Flabelligeridae sp. (NHM_955); scientificName: Flabelligeridae; kingdom: Animalia; phylum: Annelida; class: Polychaeta; order: Terebellida; family: Flabelligeridae; taxonRank: family; scientificNameAuthorship: de Saint-Joseph, 1894; **Location:** waterBody: Pacific; stateProvince: Clarion Clipperton Zone; locality: Ocean Mineral Singapore exploration claim Stratum A; verbatimLocality: OMS Stratum A; maximumDepthInMeters: 4302; locationRemarks: Deployment EB06; at Station S5; from R/V Thomas G. Thompson Cruise no. TN319; verbatimLatitude: 12'15.44; verbatimLongitude: 117'18.13; decimalLatitude: 12.25733; decimalLongitude: -117.30217; geodeticDatum: WGS84; **Identification:** identifiedBy: Helena Wiklund | Lenka Neal | Thomas Dahlgren | Adrian Glover | Madeleine Brasier | Regan Drennan | Eva Stewart; dateIdentified: 2021-04-20; identificationRemarks: identified by DNA and morphology; **Event:** eventID: OMS1_AB02_EB06; samplingProtocol: Brenke Epibenthic Sledge; eventDate: 2015-03-01; eventTime: 04:02; habitat: Abyssal plain; fieldNotes: Collected from epi net (on the epibenthic sledge); **Record Level:** language: en; institutionCode: NHMUK; collectionCode: ZOO; datasetName: ABYSSLINE; basisOfRecord: PreservedSpecimen
- **Type status:** Other material. **Occurrence:** catalogNumber: NHMUK ANEA 2023.388; recordNumber: NHM_1761A; recordedBy: Adrian Glover | Helena Wiklund | Thomas Dahlgren | Madeleine Brasier; individualCount: 1; preparations: specimen stored in 80% non-denatured ethanol aqueous solution | DNA voucher stored in buffer; otherCatalogNumbers: 0174126754; associatedSequences: OQ746711 (16S); occurrenceID: 222C7412-0FF9-53D4-8659-5715ADD19A11; **Taxon:** taxonConceptID: Flabelligeridae sp. (NHM_955); scientificName: Flabelligeridae; kingdom: Animalia; phylum: Annelida; class: Polychaeta; order: Terebellida; family: Flabelligeridae; taxonRank: family; scientificNameAuthorship: de Saint-Joseph, 1894; **Location:** waterBody: Pacific; stateProvince: Clarion Clipperton Zone; locality: Ocean Mineral Singapore exploration claim Stratum A; verbatimLocality: OMS Stratum A; maximumDepthInMeters: 4045; locationRemarks: Deployment EB10; at Station S7; from R/V Thomas G. Thompson Cruise no. TN319; verbatimLatitude: 12'10.43; verbatimLongitude: 117'11.57; decimalLatitude: 12.17383; decimalLongitude: -117.19283; geodeticDatum: WGS84; **Identification:** identifiedBy: Helena Wiklund | Lenka Neal | Thomas Dahlgren | Adrian Glover | Madeleine Brasier | Regan Drennan | Eva Stewart; dateIdentified: 2021-04-20; identificationRemarks: identified by DNA and morphology; **Event:** eventID: OMS1_AB02_EB10; samplingProtocol: Brenke Epibenthic Sledge; eventDate: 2015-03-11; eventTime: 22:49; habitat: Abyssal plain; fieldNotes: Collected from epi net (on the epibenthic sledge); **Record Level:** language: en; institutionCode: NHMUK; collectionCode: ZOO; datasetName: ABYSSLINE; basisOfRecord: PreservedSpecimen
- **Type status:** Other material. **Occurrence:** catalogNumber: NHMUK ANEA 2023.387; recordNumber: NHM_1761B; recordedBy: Adrian Glover | Helena Wiklund | Thomas Dahlgren | Madeleine Brasier; individualCount: 1; preparations: specimen stored in 80% non-denatured ethanol aqueous solution | DNA voucher stored in buffer; associatedSequences: OQ746712 (16S); occurrenceID: BDADCC75-028E-54FC-805E-0CFC01048EDD; **Taxon:** taxonConceptID: Flabelligeridae sp. (NHM_955); scientificName: Flabelligeridae; kingdom: Animalia; phylum: Annelida; class: Polychaeta; order: Terebellida; family: Flabelligeridae; taxonRank: family; scientificNameAuthorship: de Saint-Joseph, 1894; **Location:** waterBody: Pacific; stateProvince: Clarion Clipperton Zone; locality: Ocean Mineral Singapore exploration claim Stratum A; verbatimLocality: OMS Stratum A; maximumDepthInMeters: 4045; locationRemarks: Deployment EB10; at Station S7; from R/V Thomas G. Thompson Cruise no. TN319; verbatimLatitude: 12'10.43; verbatimLongitude: 117'11.57; decimalLatitude: 12.17383; decimalLongitude: -117.19283; geodeticDatum: WGS84; **Identification:** identifiedBy: Helena Wiklund | Lenka Neal | Thomas Dahlgren | Adrian Glover | Madeleine Brasier | Regan Drennan | Eva Stewart; dateIdentified: 2021-04-20; identificationRemarks: identified by DNA and morphology; **Event:** eventID: OMS1_AB02_EB10; samplingProtocol: Brenke Epibenthic Sledge; eventDate: 2015-03-11; eventTime: 22:49; habitat: Abyssal plain; fieldNotes: Collected from epi net (on the epibenthic sledge); **Record Level:** language: en; institutionCode: NHMUK; collectionCode: ZOO; datasetName: ABYSSLINE; basisOfRecord: PreservedSpecimen
- **Type status:** Other material. **Occurrence:** catalogNumber: NHMUK ANEA 2023.383; recordNumber: NHM_1164A; recordedBy: Adrian Glover | Helena Wiklund | Thomas Dahlgren | Madeleine Brasier; individualCount: 1; preparations: specimen stored in 80% non-denatured ethanol aqueous solution | DNA voucher stored in buffer; otherCatalogNumbers: 0174126608; associatedSequences: OQ746631 (16S); occurrenceID: B5AE6B54-D5BB-51AF-AE0A-7D16BC46E15B; **Taxon:** taxonConceptID: Flabelligeridae sp. (NHM_955); scientificName: Flabelligeridae; kingdom: Animalia; phylum: Annelida; class: Polychaeta; order: Terebellida; family: Flabelligeridae; taxonRank: family; scientificNameAuthorship: de Saint-Joseph, 1894; **Location:** waterBody: Pacific; stateProvince: Clarion Clipperton Zone; locality: Ocean Mineral Singapore exploration claim Stratum A; verbatimLocality: OMS Stratum A; maximumDepthInMeters: 4100; locationRemarks: Deployment EB05; at Station S2; from R/V Thomas G. Thompson Cruise no. TN319; verbatimLatitude: 12'06.93; verbatimLongitude: 117'09.87; decimalLatitude: 12.1155; decimalLongitude: -117.1645; geodeticDatum: WGS84; **Identification:** identifiedBy: Helena Wiklund | Lenka Neal | Thomas Dahlgren | Adrian Glover | Madeleine Brasier | Regan Drennan | Eva Stewart; dateIdentified: 2021-04-20; identificationRemarks: identified by DNA and morphology; **Event:** eventID: OMS1_AB02_EB05; samplingProtocol: Brenke Epibenthic Sledge; eventDate: 2015-02-26; eventTime: 21:29; habitat: Abyssal plain; fieldNotes: Collected from epi net (on the epibenthic sledge); **Record Level:** language: en; institutionCode: NHMUK; collectionCode: ZOO; datasetName: ABYSSLINE; basisOfRecord: PreservedSpecimen
- **Type status:** Other material. **Occurrence:** catalogNumber: NHMUK ANEA 2023.382; recordNumber: NHM_0955; recordedBy: Adrian Glover | Helena Wiklund | Thomas Dahlgren | Madeleine Brasier; individualCount: 1; preparations: specimen stored in 80% non-denatured ethanol aqueous solution | DNA voucher stored in buffer; otherCatalogNumbers: 0174126805; associatedSequences: OQ746596 (16S); occurrenceID: 8E9DD864-1311-5A0E-BC52-F7AB9D53EAEC; **Taxon:** taxonConceptID: Flabelligeridae sp. (NHM_955); scientificName: Flabelligeridae; kingdom: Animalia; phylum: Annelida; class: Polychaeta; order: Terebellida; family: Flabelligeridae; taxonRank: family; scientificNameAuthorship: de Saint-Joseph, 1894; **Location:** waterBody: Pacific; stateProvince: Clarion Clipperton Zone; locality: UK Seabed Resources Ltd exploration area UK-1 Stratum B; verbatimLocality: UK1 Stratum B; maximumDepthInMeters: 4198; locationRemarks: Deployment EB03; at Station U4; from R/V Thomas G. Thompson Cruise no. TN319; verbatimLatitude: 12'34.28; verbatimLongitude: 116'36.63; decimalLatitude: 12.57133; decimalLongitude: -116.6105; geodeticDatum: WGS84; **Identification:** identifiedBy: Helena Wiklund | Lenka Neal | Thomas Dahlgren | Adrian Glover | Madeleine Brasier | Regan Drennan | Eva Stewart; dateIdentified: 2021-04-20; identificationRemarks: identified by DNA and morphology; **Event:** eventID: UK1_AB02_EB03; samplingProtocol: Brenke Epibenthic Sledge; eventDate: 2015-02-23; eventTime: 05:39; habitat: Abyssal plain; fieldNotes: Collected from epi net (on the epibenthic sledge); **Record Level:** language: en; institutionCode: NHMUK; collectionCode: ZOO; datasetName: ABYSSLINE; basisOfRecord: PreservedSpecimen

##### Distribution

Eastern Clarion-Clipperton Zone, central Pacific Ocean.

##### Diagnosis

**Figure 38.**
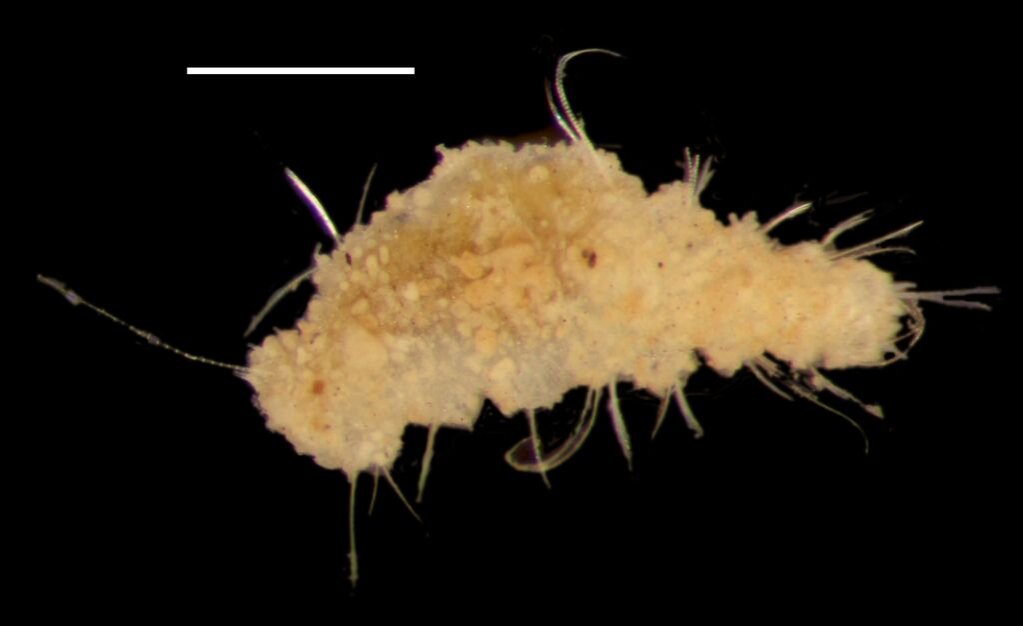
Flabelligeridae sp. (NHM_955), fragment of live specimen NHM_955. Scale bar 1 mm.

Damaged specimens (Fig. 38) consistent with placement within family Flabelligeridae, based on morphology and DNA.

#### Flabelligeridae sp. (NHM_1274)

9C82236B-8554-5E2A-947F-C197A770F844

##### Materials

- **Type status:** Other material. **Occurrence:** catalogNumber: NHMUK ANEA 2023.371; recordNumber: NHM_1274; recordedBy: Adrian Glover | Helena Wiklund | Thomas Dahlgren | Madeleine Brasier; individualCount: 1; preparations: specimen stored in 80% non-denatured ethanol aqueous solution | DNA voucher stored in buffer; otherCatalogNumbers: 0174126759; associatedSequences: OQ746651 (16S); occurrenceID: 58A621C8-6E87-5A1A-B662-1AC206417E3B; **Taxon:** taxonConceptID: Flabelligeridae sp. (NHM_1274); scientificName: Flabelligeridae; kingdom: Animalia; phylum: Annelida; class: Polychaeta; order: Terebellida; family: Flabelligeridae; taxonRank: family; scientificNameAuthorship: de Saint-Joseph, 1894; **Location:** waterBody: Pacific; stateProvince: Clarion Clipperton Zone; locality: Ocean Mineral Singapore exploration claim Stratum A; verbatimLocality: OMS Stratum A; maximumDepthInMeters: 4302; locationRemarks: Deployment EB06; at Station S5; from R/V Thomas G. Thompson Cruise no. TN319; verbatimLatitude: 12'15.44; verbatimLongitude: 117'18.13; decimalLatitude: 12.25733; decimalLongitude: -117.30217; geodeticDatum: WGS84; **Identification:** identifiedBy: Helena Wiklund | Lenka Neal | Thomas Dahlgren | Adrian Glover | Madeleine Brasier | Regan Drennan | Eva Stewart; dateIdentified: 2021-04-20; identificationRemarks: identified by DNA and morphology; **Event:** eventID: OMS1_AB02_EB06; samplingProtocol: Brenke Epibenthic Sledge; eventDate: 2015-03-01; eventTime: 04:02; habitat: Abyssal plain; fieldNotes: Collected from epi net (on the epibenthic sledge); **Record Level:** language: en; institutionCode: NHMUK; collectionCode: ZOO; datasetName: ABYSSLINE; basisOfRecord: PreservedSpecimen
- **Type status:** Other material. **Occurrence:** catalogNumber: NHMUK ANEA 2023.372; recordNumber: NHM_1453; recordedBy: Adrian Glover | Helena Wiklund | Thomas Dahlgren | Madeleine Brasier; individualCount: 1; preparations: specimen stored in 80% non-denatured ethanol aqueous solution | DNA voucher stored in buffer; otherCatalogNumbers: 0174126778; associatedSequences: OQ746682 (16S); occurrenceID: F9719B4E-3067-5C78-B007-22F3A824400C; **Taxon:** taxonConceptID: Flabelligeridae sp. (NHM_1274); scientificName: Flabelligeridae; kingdom: Animalia; phylum: Annelida; class: Polychaeta; order: Terebellida; family: Flabelligeridae; taxonRank: family; scientificNameAuthorship: de Saint-Joseph, 1894; **Location:** waterBody: Pacific; stateProvince: Clarion Clipperton Zone; locality: UK Seabed Resources Ltd exploration area UK-1 Stratum B; verbatimLocality: UK1 Stratum B; maximumDepthInMeters: 4137; locationRemarks: Deployment EB07; at Station U7; from R/V Thomas G. Thompson Cruise no. TN319; verbatimLatitude: 12'27.26; verbatimLongitude: 116'36.77; decimalLatitude: 12.45433; decimalLongitude: -116.61283; geodeticDatum: WGS84; **Identification:** identifiedBy: Helena Wiklund | Lenka Neal | Thomas Dahlgren | Adrian Glover | Madeleine Brasier | Regan Drennan | Eva Stewart; dateIdentified: 2021-04-20; identificationRemarks: identified by DNA and morphology; **Event:** eventID: UK1_AB02_EB07; samplingProtocol: Brenke Epibenthic Sledge; eventDate: 2015-03-03; eventTime: 20:40; habitat: Abyssal plain; fieldNotes: Collected from epi net (on the epibenthic sledge); **Record Level:** language: en; institutionCode: NHMUK; collectionCode: ZOO; datasetName: ABYSSLINE; basisOfRecord: PreservedSpecimen

##### Distribution

Eastern Clarion-Clipperton Zone, central Pacific Ocean.

##### Diagnosis

**Figure 39.**
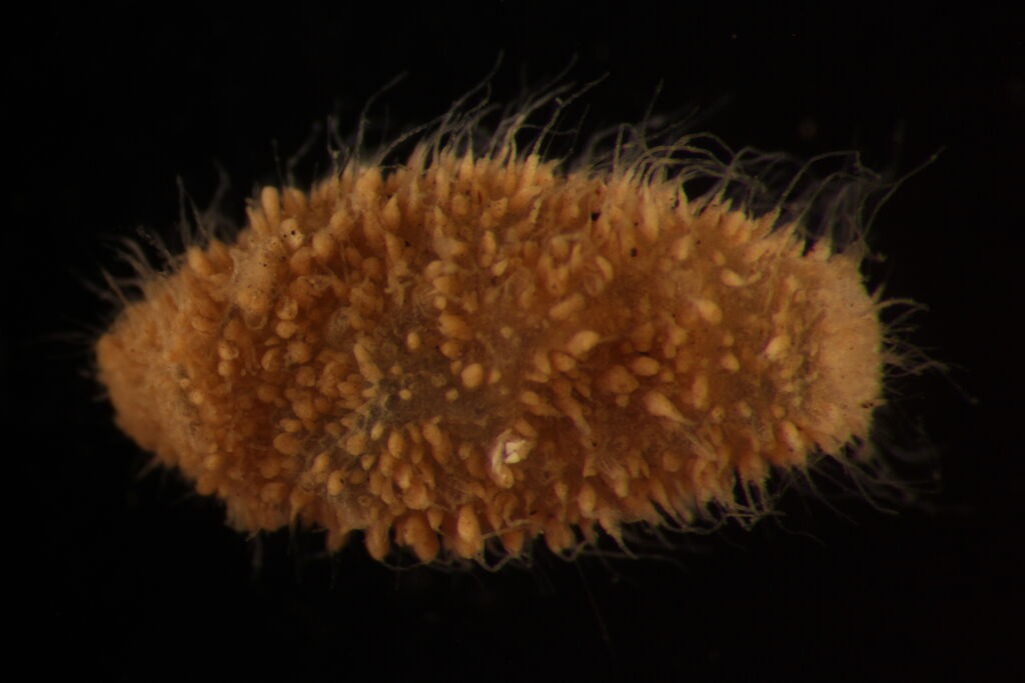
Flabelligeridae sp. (NHM_1274), fragment of a live specimen NHM_1274.

Damaged specimens (Fig. 39) consistent with placement within family Flabelligeridae, based on morphology and DNA.

#### Flabelligeridae sp. (NHM_1313)

7E4EC248-8E6B-5CD0-9B06-BB6A3E2335A3

##### Materials

- **Type status:** Other material. **Occurrence:** catalogNumber: NHMUK ANEA 2023.373; recordNumber: NHM_1313; recordedBy: Adrian Glover | Helena Wiklund | Thomas Dahlgren | Madeleine Brasier; individualCount: 1; preparations: specimen stored in 80% non-denatured ethanol aqueous solution | DNA voucher stored in buffer; otherCatalogNumbers: 0174126752; associatedSequences: OQ746656 (16S) | OQ746887 (18S); occurrenceID: 682122FA-68C2-5C9C-A37F-258BB9EAB154; **Taxon:** taxonConceptID: Flabelligeridae sp. (NHM_1313); scientificName: Flabelligeridae; kingdom: Animalia; phylum: Annelida; class: Polychaeta; order: Terebellida; family: Flabelligeridae; taxonRank: family; scientificNameAuthorship: de Saint-Joseph, 1894; **Location:** waterBody: Pacific; stateProvince: Clarion Clipperton Zone; locality: Ocean Mineral Singapore exploration claim Stratum A; verbatimLocality: OMS Stratum A; maximumDepthInMeters: 4302; locationRemarks: Deployment EB06; at Station S5; from R/V Thomas G. Thompson Cruise no. TN319; verbatimLatitude: 12'15.44; verbatimLongitude: 117'18.13; decimalLatitude: 12.25733; decimalLongitude: -117.30217; geodeticDatum: WGS84; **Identification:** identifiedBy: Helena Wiklund | Lenka Neal | Thomas Dahlgren | Adrian Glover | Madeleine Brasier | Regan Drennan | Eva Stewart; dateIdentified: 2021-04-20; identificationRemarks: identified by DNA and morphology; **Event:** eventID: OMS1_AB02_EB06; samplingProtocol: Brenke Epibenthic Sledge; eventDate: 2015-03-01; eventTime: 04:02; habitat: Abyssal plain; fieldNotes: Collected from epi net (on the epibenthic sledge); **Record Level:** language: en; institutionCode: NHMUK; collectionCode: ZOO; datasetName: ABYSSLINE; basisOfRecord: PreservedSpecimen
- **Type status:** Other material. **Occurrence:** catalogNumber: NHMUK ANEA 2023.374; recordNumber: NHM_1742; recordedBy: Adrian Glover | Helena Wiklund | Thomas Dahlgren | Madeleine Brasier; individualCount: 1; preparations: specimen stored in 80% non-denatured ethanol aqueous solution | DNA voucher stored in buffer; otherCatalogNumbers: 0174126757; associatedSequences: OQ746710 (16S); occurrenceID: 2BB43794-C386-57F0-BB72-097FA4DCB598; **Taxon:** taxonConceptID: Flabelligeridae sp. (NHM_1313); scientificName: Flabelligeridae; kingdom: Animalia; phylum: Annelida; class: Polychaeta; order: Terebellida; family: Flabelligeridae; taxonRank: family; scientificNameAuthorship: de Saint-Joseph, 1894; **Location:** waterBody: Pacific; stateProvince: Clarion Clipperton Zone; locality: Ocean Mineral Singapore exploration claim Stratum A; verbatimLocality: OMS Stratum A; maximumDepthInMeters: 4045; locationRemarks: Deployment EB10; at Station S7; from R/V Thomas G. Thompson Cruise no. TN319; verbatimLatitude: 12'10.43; verbatimLongitude: 117'11.57; decimalLatitude: 12.17383; decimalLongitude: -117.19283; geodeticDatum: WGS84; **Identification:** identifiedBy: Helena Wiklund | Lenka Neal | Thomas Dahlgren | Adrian Glover | Madeleine Brasier | Regan Drennan | Eva Stewart; dateIdentified: 2021-04-20; identificationRemarks: identified by DNA and morphology; **Event:** eventID: OMS1_AB02_EB10; samplingProtocol: Brenke Epibenthic Sledge; eventDate: 2015-03-11; eventTime: 22:49; habitat: Abyssal plain; fieldNotes: Collected from epi net (on the epibenthic sledge); **Record Level:** language: en; institutionCode: NHMUK; collectionCode: ZOO; datasetName: ABYSSLINE; basisOfRecord: PreservedSpecimen
- **Type status:** Other material. **Occurrence:** catalogNumber: NHMUK ANEA 2023.375; recordNumber: NHM_1761; recordedBy: Adrian Glover | Helena Wiklund | Thomas Dahlgren | Madeleine Brasier; individualCount: 1; preparations: specimen stored in 80% non-denatured ethanol aqueous solution | DNA voucher stored in buffer; otherCatalogNumbers: 0174126733; associatedSequences: OQ746713 (16S); occurrenceID: 158AE3DC-BC9A-5F16-8D16-7C584946EFDE; **Taxon:** taxonConceptID: Flabelligeridae sp. (NHM_1313); scientificName: Flabelligeridae; kingdom: Animalia; phylum: Annelida; class: Polychaeta; order: Terebellida; family: Flabelligeridae; taxonRank: family; scientificNameAuthorship: de Saint-Joseph, 1894; **Location:** waterBody: Pacific; stateProvince: Clarion Clipperton Zone; locality: Ocean Mineral Singapore exploration claim Stratum A; verbatimLocality: OMS Stratum A; maximumDepthInMeters: 4045; locationRemarks: Deployment EB10; at Station S7; from R/V Thomas G. Thompson Cruise no. TN319; verbatimLatitude: 12'10.43; verbatimLongitude: 117'11.57; decimalLatitude: 12.17383; decimalLongitude: -117.19283; geodeticDatum: WGS84; **Identification:** identifiedBy: Helena Wiklund | Lenka Neal | Thomas Dahlgren | Adrian Glover | Madeleine Brasier | Regan Drennan | Eva Stewart; dateIdentified: 2021-04-20; identificationRemarks: identified by DNA and morphology; **Event:** eventID: OMS1_AB02_EB10; samplingProtocol: Brenke Epibenthic Sledge; eventDate: 2015-03-11; eventTime: 22:49; habitat: Abyssal plain; fieldNotes: Collected from epi net (on the epibenthic sledge); **Record Level:** language: en; institutionCode: NHMUK; collectionCode: ZOO; datasetName: ABYSSLINE; basisOfRecord: PreservedSpecimen

##### Distribution

Eastern Clarion-Clipperton Zone, central Pacific Ocean.

##### Diagnosis

**Figure 40.**
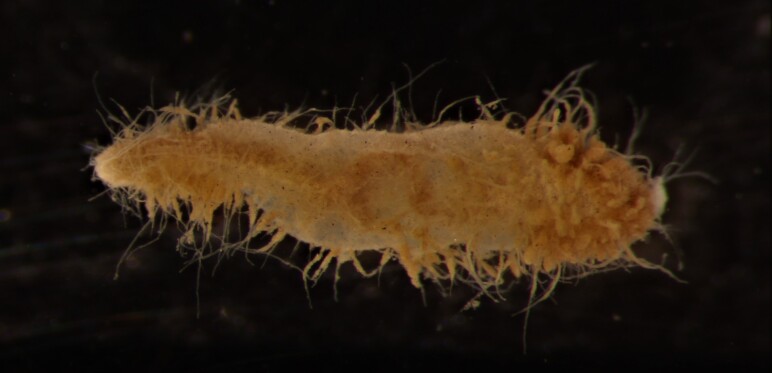
Flabelligeridae sp. (NHM_1313), fragment of live specimen NHM_1742.

Damaged specimens (Fig. 40) consistent with placement within family Flabelligeridae, based on morphology and DNA.

#### Flabelligeridae sp. (NHM_1638)

3A2BB00A-740A-5181-BA47-44A28CBFB86C

##### Materials

- **Type status:** Other material. **Occurrence:** catalogNumber: NHMUK ANEA 2023.376; recordNumber: NHM_1175; recordedBy: Adrian Glover | Helena Wiklund | Thomas Dahlgren | Madeleine Brasier; individualCount: 1; preparations: specimen stored in 80% non-denatured ethanol aqueous solution | DNA voucher stored in buffer; otherCatalogNumbers: 0174126609; associatedSequences: OQ746638 (16S) | OQ738568 (COI); occurrenceID: 4FC4A494-6B5F-5BC4-AFE4-F459ED1141D6; **Taxon:** taxonConceptID: Flabelligeridae sp. (NHM_1638); scientificName: Flabelligeridae; kingdom: Animalia; phylum: Annelida; class: Polychaeta; order: Terebellida; family: Flabelligeridae; taxonRank: family; scientificNameAuthorship: de Saint-Joseph, 1894; **Location:** waterBody: Pacific; stateProvince: Clarion Clipperton Zone; locality: Ocean Mineral Singapore exploration claim Stratum A; verbatimLocality: OMS Stratum A; maximumDepthInMeters: 4100; locationRemarks: Deployment EB05; at Station S2; from R/V Thomas G. Thompson Cruise no. TN319; verbatimLatitude: 12'06.93; verbatimLongitude: 117'09.87; decimalLatitude: 12.1155; decimalLongitude: -117.1645; geodeticDatum: WGS84; **Identification:** identifiedBy: Helena Wiklund | Lenka Neal | Thomas Dahlgren | Adrian Glover | Madeleine Brasier | Regan Drennan | Eva Stewart; dateIdentified: 2021-04-20; identificationRemarks: identified by DNA and morphology; **Event:** eventID: OMS1_AB02_EB05; samplingProtocol: Brenke Epibenthic Sledge; eventDate: 2015-02-26; eventTime: 21:29; habitat: Abyssal plain; fieldNotes: Collected from epi net (on the epibenthic sledge); **Record Level:** language: en; institutionCode: NHMUK; collectionCode: ZOO; datasetName: ABYSSLINE; basisOfRecord: PreservedSpecimen
- **Type status:** Other material. **Occurrence:** recordNumber: NHM_1638; recordedBy: Adrian Glover | Helena Wiklund | Thomas Dahlgren | Madeleine Brasier; individualCount: 1; preparations: DNA voucher stored in buffer; otherCatalogNumbers: 0174126743; associatedSequences: OQ746699 (16S) | OQ746895 (18S); occurrenceID: C86053A5-3549-500C-8D83-0E316CD9503C; **Taxon:** taxonConceptID: Flabelligeridae sp. (NHM_1638); scientificName: Flabelligeridae; kingdom: Animalia; phylum: Annelida; class: Polychaeta; order: Terebellida; family: Flabelligeridae; taxonRank: family; scientificNameAuthorship: de Saint-Joseph, 1894; **Location:** waterBody: Pacific; stateProvince: Clarion Clipperton Zone; locality: UK Seabed Resources Ltd exploration area UK-1 Stratum B; verbatimLocality: UK1 Stratum B; maximumDepthInMeters: 4233; locationRemarks: Deployment EB09; at Station U1; from R/V Thomas G. Thompson Cruise no. TN319; verbatimLatitude: 12'21.81; verbatimLongitude: 116'40.86; decimalLatitude: 12.3635; decimalLongitude: -116.681; geodeticDatum: WGS84; **Identification:** identifiedBy: Helena Wiklund | Lenka Neal | Thomas Dahlgren | Adrian Glover | Madeleine Brasier | Regan Drennan | Eva Stewart; dateIdentified: 2021-04-20; identificationRemarks: identified by DNA and morphology; **Event:** eventID: UK1_AB02_EB09; samplingProtocol: Brenke Epibenthic Sledge; eventDate: 2015-03-10; eventTime: 10:46; habitat: Abyssal plain; fieldNotes: Collected from supra net (on the epibenthic sledge); **Record Level:** language: en; institutionCode: NHMUK; collectionCode: ZOO; datasetName: ABYSSLINE; basisOfRecord: PreservedSpecimen

##### Distribution

Eastern Clarion-Clipperton Zone, central Pacific Ocean.

##### Diagnosis

**Figure 41.**
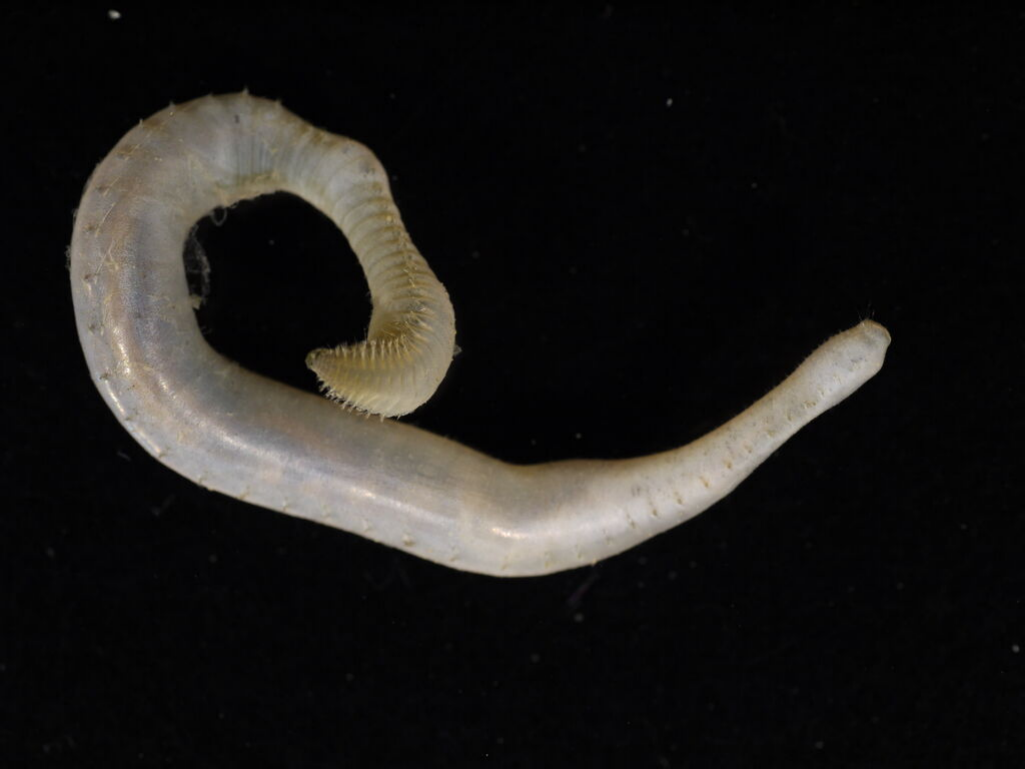
Flabelligeridae sp. (NHM_1638), live specimen NHM_1638 in lateral view.

Complete (Fig. 41) specimens consistent with placement within family Flabelligeridae, based on morphology and DNA.

#### Flabelligeridae sp. (NHM_2124)

A6F9BB00-2E4C-5AF2-AAF0-DD7A5591880A

##### Materials

- **Type status:** Other material. **Occurrence:** catalogNumber: NHMUK ANEA 2023.377; recordNumber: NHM_2124; recordedBy: Adrian Glover | Helena Wiklund | Thomas Dahlgren | Madeleine Brasier; individualCount: 1; preparations: specimen stored in 80% non-denatured ethanol aqueous solution | DNA voucher stored in buffer; otherCatalogNumbers: 0174126785; associatedSequences: OQ746758 (16S) | OQ746910 (18S); occurrenceID: 65471A5A-BF8F-531E-8EAF-EBF17725864A; **Taxon:** taxonConceptID: Flabelligeridae sp. (NHM_2124); scientificName: Flabelligeridae; kingdom: Animalia; phylum: Annelida; class: Polychaeta; order: Terebellida; family: Flabelligeridae; taxonRank: family; scientificNameAuthorship: de Saint-Joseph, 1894; **Location:** waterBody: Pacific; stateProvince: Clarion Clipperton Zone; locality: Area of Particular Interest APEI-6; verbatimLocality: APEI-6; maximumDepthInMeters: 4026; locationRemarks: Deployment EB13; at Station APEI; from R/V Thomas G. Thompson Cruise no. TN319; verbatimLatitude: 19 27.874; verbatimLongitude: 120 01.525; decimalLatitude: 19.46457; decimalLongitude: -120.02542; geodeticDatum: WGS84; **Identification:** identifiedBy: Helena Wiklund | Lenka Neal | Thomas Dahlgren | Adrian Glover | Madeleine Brasier | Regan Drennan | Eva Stewart; dateIdentified: 2021-04-20; identificationRemarks: identified by DNA and morphology; **Event:** eventID: APEI6_AB02_EB13; samplingProtocol: Brenke Epibenthic Sledge; eventDate: 2015-03-20; eventTime: 16:12; habitat: Abyssal plain; fieldNotes: Collected from epi net (on the epibenthic sledge); **Record Level:** language: en; institutionCode: NHMUK; collectionCode: ZOO; datasetName: ABYSSLINE; basisOfRecord: PreservedSpecimen

##### Distribution

Eastern Clarion-Clipperton Zone, central Pacific Ocean.

##### Diagnosis

**Figure 42.**
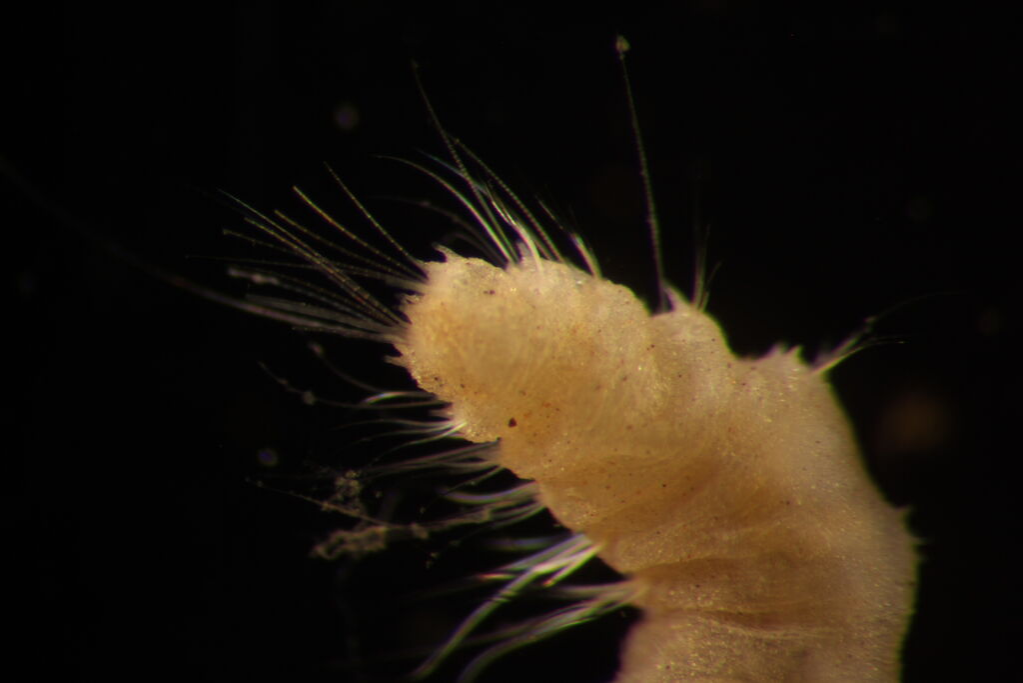
Flabelligeridae sp. (NHM_2124), anterior end of live specimen NHM_2124.

Damaged specimen (Fig. 42) consistent with placement within family Flabelligeridae, based on morphology and DNA.

#### Flabelligeridae sp. (NHM_045)

2CB97810-B469-5876-BA5F-5CCFD7393D28

##### Materials

- **Type status:** Other material. **Occurrence:** catalogNumber: NHMUK ANEA 2023.370; recordNumber: NHM_1159; recordedBy: Adrian Glover | Helena Wiklund | Thomas Dahlgren | Madeleine Brasier; individualCount: 1; preparations: specimen stored in 80% non-denatured ethanol aqueous solution | DNA voucher stored in buffer; otherCatalogNumbers: 0174126729; associatedSequences: OQ746628 (16S) | OQ746877 (18S); occurrenceID: 471CC6E7-2909-54B5-81D6-62696C67F3AC; **Taxon:** taxonConceptID: Flabelligeridae sp. (NHM_045); scientificName: Flabelligeridae; kingdom: Animalia; phylum: Annelida; class: Polychaeta; order: Terebellida; family: Flabelligeridae; taxonRank: family; scientificNameAuthorship: de Saint-Joseph, 1894; **Location:** waterBody: Pacific; stateProvince: Clarion Clipperton Zone; locality: Ocean Mineral Singapore exploration claim Stratum A; verbatimLocality: OMS Stratum A; maximumDepthInMeters: 4100; locationRemarks: Deployment EB05; at Station S2; from R/V Thomas G. Thompson Cruise no. TN319; verbatimLatitude: 12'06.93; verbatimLongitude: 117'09.87; decimalLatitude: 12.1155; decimalLongitude: -117.1645; geodeticDatum: WGS84; **Identification:** identifiedBy: Helena Wiklund | Lenka Neal | Thomas Dahlgren | Adrian Glover | Madeleine Brasier | Regan Drennan | Eva Stewart; dateIdentified: 2021-04-20; identificationRemarks: identified by DNA and morphology; **Event:** eventID: OMS1_AB02_EB05; samplingProtocol: Brenke Epibenthic Sledge; eventDate: 2015-02-26; eventTime: 21:29; habitat: Abyssal plain; fieldNotes: Collected from epi net (on the epibenthic sledge); **Record Level:** language: en; institutionCode: NHMUK; collectionCode: ZOO; datasetName: ABYSSLINE; basisOfRecord: PreservedSpecimen
- **Type status:** Other material. **Occurrence:** catalogNumber: NHMUK ANEA 2023.369; recordNumber: NHM_0945B; recordedBy: Adrian Glover | Helena Wiklund | Thomas Dahlgren | Madeleine Brasier; individualCount: 1; preparations: specimen stored in 80% non-denatured ethanol aqueous solution | DNA voucher stored in buffer; otherCatalogNumbers: 0174126557; associatedSequences: OQ746593 (16S); occurrenceID: 22DA3FC9-7E47-587E-9B9A-62AF954556FD; **Taxon:** taxonConceptID: Flabelligeridae sp. (NHM_045); scientificName: Flabelligeridae; kingdom: Animalia; phylum: Annelida; class: Polychaeta; order: Terebellida; family: Flabelligeridae; taxonRank: family; scientificNameAuthorship: de Saint-Joseph, 1894; **Location:** waterBody: Pacific; stateProvince: Clarion Clipperton Zone; locality: UK Seabed Resources Ltd exploration area UK-1 Stratum B; verbatimLocality: UK1 Stratum B; maximumDepthInMeters: 4198; locationRemarks: Deployment EB03; at Station U4; from R/V Thomas G. Thompson Cruise no. TN319; verbatimLatitude: 12'34.28; verbatimLongitude: 116'36.63; decimalLatitude: 12.57133; decimalLongitude: -116.6105; geodeticDatum: WGS84; **Identification:** identifiedBy: Helena Wiklund | Lenka Neal | Thomas Dahlgren | Adrian Glover | Madeleine Brasier | Regan Drennan | Eva Stewart; dateIdentified: 2021-04-20; identificationRemarks: identified by DNA and morphology; **Event:** eventID: UK1_AB02_EB03; samplingProtocol: Brenke Epibenthic Sledge; eventDate: 2015-02-23; eventTime: 05:39; habitat: Abyssal plain; fieldNotes: Collected from epi net (on the epibenthic sledge); **Record Level:** language: en; institutionCode: NHMUK; collectionCode: ZOO; datasetName: ABYSSLINE; basisOfRecord: PreservedSpecimen
- **Type status:** Other material. **Occurrence:** recordNumber: NHM_0045; recordedBy: Adrian Glover | Helena Wiklund | Thomas Dahlgren | Magdalena Georgieva; individualCount: 1; preparations: Tissue voucher stored in 80% non-denatured ethanol aqueous solution | DNA voucher stored in buffer; otherCatalogNumbers: 0109405385 | 0174127317; associatedSequences: OQ746471 (16S) | OQ746794 (18S) | OQ738497 (COI); occurrenceID: E3E86B41-80A1-58ED-A8E8-F52BC0E24356; **Taxon:** taxonConceptID: Flabelligeridae sp. (NHM_045); scientificName: Flabelligeridae; kingdom: Animalia; phylum: Annelida; class: Polychaeta; order: Terebellida; family: Flabelligeridae; taxonRank: family; scientificNameAuthorship: de Saint-Joseph, 1894; **Location:** waterBody: Pacific; stateProvince: Clarion Clipperton Zone; locality: UK Seabed Resources Ltd exploration area UK-1 Stratum A; verbatimLocality: UK1 Stratum A; maximumDepthInMeters: 4336; locationRemarks: Deployment EB01; at Station B-K-E; from R/V Melville Cruise no. MV1313; verbatimLatitude: 13°50.232; verbatimLongitude: 116°33.506; decimalLatitude: 13.8372; decimalLongitude: -116.55843; geodeticDatum: WGS84; **Identification:** identifiedBy: Helena Wiklund | Lenka Neal | Thomas Dahlgren | Adrian Glover | Madeleine Brasier | Regan Drennan | Eva Stewart; dateIdentified: 2021-04-20; identificationRemarks: identified by DNA and morphology; **Event:** eventID: UK1_AB01_EB01; samplingProtocol: Brenke Epibenthic Sledge; eventDate: 2013-10-09; eventTime: 10:26; habitat: Abyssal plain; fieldNotes: Collected from epi net (on the epibenthic sledge); **Record Level:** language: en; institutionCode: NHMUK; collectionCode: ZOO; datasetName: ABYSSLINE; basisOfRecord: PreservedSpecimen

##### Distribution

Eastern Clarion-Clipperton Zone, central Pacific Ocean.

##### Diagnosis

Damaged specimens (Fig. 43) consistent with placement within family Flabelligeridae, based on morphology and DNA.

### Glyceridae Grube, 1850

#### Glyceridae sp. (NHM_207)

C4031937-0241-5907-8D2F-A6A5CE79E51E

##### Materials

- **Type status:** Other material. **Occurrence:** catalogNumber: NHMUK ANEA 2023.402; recordNumber: NHM_1304; recordedBy: Adrian Glover | Helena Wiklund | Thomas Dahlgren | Madeleine Brasier; individualCount: 1; preparations: specimen stored in 80% non-denatured ethanol aqueous solution | DNA voucher stored in buffer; otherCatalogNumbers: 0174126589; associatedSequences: OQ746655 (16S); occurrenceID: 4DF24331-BABA-5C73-BE1C-1E5EDDA696FA; **Taxon:** taxonConceptID: Glyceridae sp. (NHM_207); scientificName: Glyceridae; kingdom: Animalia; phylum: Annelida; class: Polychaeta; order: Phyllodocida; family: Glyceridae; taxonRank: family; scientificNameAuthorship: Grube, 1850; **Location:** waterBody: Pacific; stateProvince: Clarion Clipperton Zone; locality: Ocean Mineral Singapore exploration claim Stratum A; verbatimLocality: OMS Stratum A; maximumDepthInMeters: 4302; locationRemarks: Deployment EB06; at Station S5; from R/V Thomas G. Thompson Cruise no. TN319; verbatimLatitude: 12'15.44; verbatimLongitude: 117'18.13; decimalLatitude: 12.25733; decimalLongitude: -117.30217; geodeticDatum: WGS84; **Identification:** identifiedBy: Helena Wiklund | Lenka Neal | Thomas Dahlgren | Adrian Glover | Madeleine Brasier | Regan Drennan | Eva Stewart; dateIdentified: 2021-04-20; identificationRemarks: identified by DNA and morphology; **Event:** eventID: OMS1_AB02_EB06; samplingProtocol: Brenke Epibenthic Sledge; eventDate: 2015-03-01; eventTime: 04:02; habitat: Abyssal plain; fieldNotes: Collected from epi net (on the epibenthic sledge); **Record Level:** language: en; institutionCode: NHMUK; collectionCode: ZOO; datasetName: ABYSSLINE; basisOfRecord: PreservedSpecimen
- **Type status:** Other material. **Occurrence:** catalogNumber: NHMUK ANEA 2023.400; recordNumber: NHM_1046; recordedBy: Adrian Glover | Helena Wiklund | Thomas Dahlgren | Madeleine Brasier; individualCount: 1; preparations: specimen stored in 80% non-denatured ethanol aqueous solution | DNA voucher stored in buffer; otherCatalogNumbers: 0174126545; associatedSequences: OQ746609 (16S); occurrenceID: C704599C-75E7-52BE-B4F5-29E15EE2FE38; **Taxon:** taxonConceptID: Glyceridae sp. (NHM_207); scientificName: Glyceridae; kingdom: Animalia; phylum: Annelida; class: Polychaeta; order: Phyllodocida; family: Glyceridae; taxonRank: family; scientificNameAuthorship: Grube, 1850; **Location:** waterBody: Pacific; stateProvince: Clarion Clipperton Zone; locality: Ocean Mineral Singapore exploration claim Stratum A; verbatimLocality: OMS Stratum A; maximumDepthInMeters: 4114; locationRemarks: Deployment BC08; at Station S3; from R/V Thomas G. Thompson Cruise no. TN319; verbatimLatitude: 12'10.868; verbatimLongitude: 117'15.659; decimalLatitude: 12.18113; decimalLongitude: -117.26098; geodeticDatum: WGS84; **Identification:** identifiedBy: Helena Wiklund | Lenka Neal | Thomas Dahlgren | Adrian Glover | Madeleine Brasier | Regan Drennan | Eva Stewart; dateIdentified: 2021-04-20; identificationRemarks: identified by DNA and morphology; **Event:** eventID: OMS1_AB02_BC08; samplingProtocol: USNEL Box Core; eventDate: 2015-02-25; eventTime: 05:58; habitat: Abyssal plain; fieldNotes: Collected from 0-2 cm layer of box core using a 300 micron sieve; **Record Level:** language: en; institutionCode: NHMUK; collectionCode: ZOO; datasetName: ABYSSLINE; basisOfRecord: PreservedSpecimen
- **Type status:** Other material. **Occurrence:** catalogNumber: NHMUK ANEA 2023.401; recordNumber: NHM_1070; recordedBy: Adrian Glover | Helena Wiklund | Thomas Dahlgren | Madeleine Brasier; individualCount: 1; preparations: specimen stored in 80% non-denatured ethanol aqueous solution | DNA voucher stored in buffer; otherCatalogNumbers: 0174126616; associatedSequences: OQ746611 (16S); occurrenceID: CD4F9EDE-2338-5BFE-95EA-2F3FF00612BD; **Taxon:** taxonConceptID: Glyceridae sp. (NHM_207); scientificName: Glyceridae; kingdom: Animalia; phylum: Annelida; class: Polychaeta; order: Phyllodocida; family: Glyceridae; taxonRank: family; scientificNameAuthorship: Grube, 1850; **Location:** waterBody: Pacific; stateProvince: Clarion Clipperton Zone; locality: Ocean Mineral Singapore exploration claim Stratum A; verbatimLocality: OMS Stratum A; maximumDepthInMeters: 4100; locationRemarks: Deployment EB05; at Station S2; from R/V Thomas G. Thompson Cruise no. TN319; verbatimLatitude: 12'06.93; verbatimLongitude: 117'09.87; decimalLatitude: 12.1155; decimalLongitude: -117.1645; geodeticDatum: WGS84; **Identification:** identifiedBy: Helena Wiklund | Lenka Neal | Thomas Dahlgren | Adrian Glover | Madeleine Brasier | Regan Drennan | Eva Stewart; dateIdentified: 2021-04-20; identificationRemarks: identified by DNA and morphology; **Event:** eventID: OMS1_AB02_EB05; samplingProtocol: Brenke Epibenthic Sledge; eventDate: 2015-02-26; eventTime: 21:29; habitat: Abyssal plain; fieldNotes: Collected from epi net (on the epibenthic sledge); **Record Level:** language: en; institutionCode: NHMUK; collectionCode: ZOO; datasetName: ABYSSLINE; basisOfRecord: PreservedSpecimen
- **Type status:** Other material. **Occurrence:** catalogNumber: NHMUK ANEA 2023.397; recordNumber: NHM_0844; recordedBy: Adrian Glover | Helena Wiklund | Thomas Dahlgren | Madeleine Brasier; individualCount: 1; preparations: specimen stored in 80% non-denatured ethanol aqueous solution | DNA voucher stored in buffer; otherCatalogNumbers: 0174126548; associatedSequences: OQ746566 (16S); occurrenceID: 14402E39-7C23-5737-BFE5-EDEB1D99F6DD; **Taxon:** taxonConceptID: Glyceridae sp. (NHM_207); scientificName: Glyceridae; kingdom: Animalia; phylum: Annelida; class: Polychaeta; order: Phyllodocida; family: Glyceridae; taxonRank: family; scientificNameAuthorship: Grube, 1850; **Location:** waterBody: Pacific; stateProvince: Clarion Clipperton Zone; locality: UK Seabed Resources Ltd exploration area UK-1 Stratum B; verbatimLocality: UK1 Stratum B; maximumDepthInMeters: 4218; locationRemarks: Deployment BC05; at Station U4; from R/V Thomas G. Thompson Cruise no. TN319; verbatimLatitude: 12'34.742; verbatimLongitude: 116'43.427; decimalLatitude: 12.57903; decimalLongitude: -116.72378; geodeticDatum: WGS84; **Identification:** identifiedBy: Helena Wiklund | Lenka Neal | Thomas Dahlgren | Adrian Glover | Madeleine Brasier | Regan Drennan | Eva Stewart; dateIdentified: 2021-04-20; identificationRemarks: identified by DNA and morphology; **Event:** eventID: UK1_AB02_BC05; samplingProtocol: USNEL Box Core; eventDate: 2015-02-21; eventTime: 16:23; habitat: Abyssal plain; fieldNotes: Collected from 0-2 cm layer of box core using a 300 micron sieve; **Record Level:** language: en; institutionCode: NHMUK; collectionCode: ZOO; datasetName: ABYSSLINE; basisOfRecord: PreservedSpecimen
- **Type status:** Other material. **Occurrence:** catalogNumber: NHMUK ANEA 2023.398; recordNumber: NHM_0845; recordedBy: Adrian Glover | Helena Wiklund | Thomas Dahlgren | Madeleine Brasier; individualCount: 1; preparations: specimen stored in 80% non-denatured ethanol aqueous solution | DNA voucher stored in buffer; otherCatalogNumbers: 0174126531; associatedSequences: OQ746567 (16S); occurrenceID: 29FA9BCC-0517-5E66-B9F7-F85BB431B7A6; **Taxon:** taxonConceptID: Glyceridae sp. (NHM_207); scientificName: Glyceridae; kingdom: Animalia; phylum: Annelida; class: Polychaeta; order: Phyllodocida; family: Glyceridae; taxonRank: family; scientificNameAuthorship: Grube, 1850; **Location:** waterBody: Pacific; stateProvince: Clarion Clipperton Zone; locality: UK Seabed Resources Ltd exploration area UK-1 Stratum B; verbatimLocality: UK1 Stratum B; maximumDepthInMeters: 4218; locationRemarks: Deployment BC05; at Station U4; from R/V Thomas G. Thompson Cruise no. TN319; verbatimLatitude: 12'34.742; verbatimLongitude: 116'43.427; decimalLatitude: 12.57903; decimalLongitude: -116.72378; geodeticDatum: WGS84; **Identification:** identifiedBy: Helena Wiklund | Lenka Neal | Thomas Dahlgren | Adrian Glover | Madeleine Brasier | Regan Drennan | Eva Stewart; dateIdentified: 2021-04-20; identificationRemarks: identified by DNA and morphology; **Event:** eventID: UK1_AB02_BC05; samplingProtocol: USNEL Box Core; eventDate: 2015-02-21; eventTime: 16:23; habitat: Abyssal plain; fieldNotes: Collected from 0-2 cm layer of box core using a 300 micron sieve; **Record Level:** language: en; institutionCode: NHMUK; collectionCode: ZOO; datasetName: ABYSSLINE; basisOfRecord: PreservedSpecimen
- **Type status:** Other material. **Occurrence:** catalogNumber: NHMUK ANEA 2023.393; recordNumber: NHM_0359; recordedBy: Adrian Glover | Helena Wiklund | Thomas Dahlgren | Magdalena Georgieva; individualCount: 1; preparations: specimen stored in 80% non-denatured ethanol aqueous solution | DNA voucher stored in buffer; otherCatalogNumbers: 0174127371; associatedSequences: OQ746504 (16S) | OQ746821 (18S); occurrenceID: 15B194EF-91DB-5DFA-AEFE-4425B8C759E5; **Taxon:** taxonConceptID: Glyceridae sp. (NHM_207); scientificName: Glyceridae; kingdom: Animalia; phylum: Annelida; class: Polychaeta; order: Phyllodocida; family: Glyceridae; taxonRank: family; scientificNameAuthorship: Grube, 1850; **Location:** waterBody: Pacific; stateProvince: Clarion Clipperton Zone; locality: UK Seabed Resources Ltd exploration area UK-1 Stratum A; verbatimLocality: UK1 Stratum A; maximumDepthInMeters: 4182; locationRemarks: Deployment EB05; at Station H-J; from R/V Melville Cruise no. MV1313; verbatimLatitude: 13°55.984; verbatimLongitude: 116°42.977; decimalLatitude: 13.93307; decimalLongitude: -116.72378; geodeticDatum: WGS84; **Identification:** identifiedBy: Helena Wiklund | Lenka Neal | Thomas Dahlgren | Adrian Glover | Madeleine Brasier | Regan Drennan | Eva Stewart; dateIdentified: 2021-04-20; identificationRemarks: identified by DNA and morphology; **Event:** eventID: UK1_AB01_EB05; samplingProtocol: Brenke Epibenthic Sledge; eventDate: 2013-10-19; eventTime: 12:16; habitat: Abyssal plain; fieldNotes: Collected from epi net (on the epibenthic sledge); **Record Level:** language: en; institutionCode: NHMUK; collectionCode: ZOO; datasetName: ABYSSLINE; basisOfRecord: PreservedSpecimen
- **Type status:** Other material. **Occurrence:** catalogNumber: NHMUK ANEA 2023.392; recordNumber: NHM_0349; recordedBy: Adrian Glover | Helena Wiklund | Thomas Dahlgren | Magdalena Georgieva; individualCount: 1; preparations: specimen stored in 80% non-denatured ethanol aqueous solution | DNA voucher stored in buffer; otherCatalogNumbers: 0174127388; associatedSequences: OQ746502 (16S) | OQ746820 (18S); occurrenceID: 0E0FD453-EC04-57E1-84AD-56A265ED93D7; **Taxon:** taxonConceptID: Glyceridae sp. (NHM_207); scientificName: Glyceridae; kingdom: Animalia; phylum: Annelida; class: Polychaeta; order: Phyllodocida; family: Glyceridae; taxonRank: family; scientificNameAuthorship: Grube, 1850; **Location:** waterBody: Pacific; stateProvince: Clarion Clipperton Zone; locality: UK Seabed Resources Ltd exploration area UK-1 Stratum A; verbatimLocality: UK1 Stratum A; maximumDepthInMeters: 4150; locationRemarks: Deployment BC11; at Station H; from R/V Melville Cruise no. MV1313; verbatimLatitude: 13°53.300; verbatimLongitude: 116°41.399; decimalLatitude: 13.88833; decimalLongitude: -116.68998; geodeticDatum: WGS84; **Identification:** identifiedBy: Helena Wiklund | Lenka Neal | Thomas Dahlgren | Adrian Glover | Madeleine Brasier | Regan Drennan | Eva Stewart; dateIdentified: 2021-04-20; identificationRemarks: identified by DNA and morphology; **Event:** eventID: UK1_AB01_BC11; samplingProtocol: USNEL Box Core; eventDate: 2013-10-19; eventTime: 02:25; habitat: Abyssal plain; fieldNotes: Collected from 0-2 cm layer of box core using a 300 micron sieve; **Record Level:** language: en; institutionCode: NHMUK; collectionCode: ZOO; datasetName: ABYSSLINE; basisOfRecord: PreservedSpecimen
- **Type status:** Other material. **Occurrence:** catalogNumber: NHMUK ANEA 2023.403; recordNumber: NHM_1658; recordedBy: Adrian Glover | Helena Wiklund | Thomas Dahlgren | Madeleine Brasier; individualCount: 1; preparations: specimen stored in 80% non-denatured ethanol aqueous solution | DNA voucher stored in buffer; otherCatalogNumbers: 0174126213; associatedSequences: OQ746703 (16S); occurrenceID: 30E12C58-483A-50F8-8139-B4F2EC43041F; **Taxon:** taxonConceptID: Glyceridae sp. (NHM_207); scientificName: Glyceridae; kingdom: Animalia; phylum: Annelida; class: Polychaeta; order: Phyllodocida; family: Glyceridae; taxonRank: family; scientificNameAuthorship: Grube, 1850; **Location:** waterBody: Pacific; stateProvince: Clarion Clipperton Zone; locality: UK Seabed Resources Ltd exploration area UK-1 Stratum B; verbatimLocality: UK1 Stratum B; maximumDepthInMeters: 4233; locationRemarks: Deployment EB09; at Station U1; from R/V Thomas G. Thompson Cruise no. TN319; verbatimLatitude: 12'21.81; verbatimLongitude: 116'40.86; decimalLatitude: 12.3635; decimalLongitude: -116.681; geodeticDatum: WGS84; **Identification:** identifiedBy: Helena Wiklund | Lenka Neal | Thomas Dahlgren | Adrian Glover | Madeleine Brasier | Regan Drennan | Eva Stewart; dateIdentified: 2021-04-20; identificationRemarks: identified by DNA and morphology; **Event:** eventID: UK1_AB02_EB09; samplingProtocol: Brenke Epibenthic Sledge; eventDate: 2015-03-10; eventTime: 10:46; habitat: Abyssal plain; fieldNotes: Collected from epi net (on the epibenthic sledge); **Record Level:** language: en; institutionCode: NHMUK; collectionCode: ZOO; datasetName: ABYSSLINE; basisOfRecord: PreservedSpecimen
- **Type status:** Other material. **Occurrence:** catalogNumber: NHMUK ANEA 2023.399; recordNumber: NHM_0906; recordedBy: Adrian Glover | Helena Wiklund | Thomas Dahlgren | Madeleine Brasier; individualCount: 1; preparations: specimen stored in 80% non-denatured ethanol aqueous solution | DNA voucher stored in buffer; otherCatalogNumbers: 0174126604; associatedSequences: OQ746575 (16S); occurrenceID: 02F11B3B-5F65-5DE5-BBF7-92A41DAAF159; **Taxon:** taxonConceptID: Glyceridae sp. (NHM_207); scientificName: Glyceridae; kingdom: Animalia; phylum: Annelida; class: Polychaeta; order: Phyllodocida; family: Glyceridae; taxonRank: family; scientificNameAuthorship: Grube, 1850; **Location:** waterBody: Pacific; stateProvince: Clarion Clipperton Zone; locality: UK Seabed Resources Ltd exploration area UK-1 Stratum B; verbatimLocality: UK1 Stratum B; maximumDepthInMeters: 4198; locationRemarks: Deployment EB03; at Station U4; from R/V Thomas G. Thompson Cruise no. TN319; verbatimLatitude: 12'34.28; verbatimLongitude: 116'36.63; decimalLatitude: 12.57133; decimalLongitude: -116.6105; geodeticDatum: WGS84; **Identification:** identifiedBy: Helena Wiklund | Lenka Neal | Thomas Dahlgren | Adrian Glover | Madeleine Brasier | Regan Drennan | Eva Stewart; dateIdentified: 2021-04-20; identificationRemarks: identified by DNA and morphology; **Event:** eventID: UK1_AB02_EB03; samplingProtocol: Brenke Epibenthic Sledge; eventDate: 2015-02-23; eventTime: 05:39; habitat: Abyssal plain; fieldNotes: Collected from epi net (on the epibenthic sledge); **Record Level:** language: en; institutionCode: NHMUK; collectionCode: ZOO; datasetName: ABYSSLINE; basisOfRecord: PreservedSpecimen
- **Type status:** Other material. **Occurrence:** catalogNumber: NHMUK ANEA 2023.395; recordNumber: NHM_0704; recordedBy: Adrian Glover | Helena Wiklund | Thomas Dahlgren | Madeleine Brasier; individualCount: 1; preparations: specimen stored in 80% non-denatured ethanol aqueous solution | DNA voucher stored in buffer; otherCatalogNumbers: 0174126529; associatedSequences: OQ746542 (16S); occurrenceID: CA9FCD73-B060-5D6A-A1A9-C9D5BA4EC691; **Taxon:** taxonConceptID: Glyceridae sp. (NHM_207); scientificName: Glyceridae; kingdom: Animalia; phylum: Annelida; class: Polychaeta; order: Phyllodocida; family: Glyceridae; taxonRank: family; scientificNameAuthorship: Grube, 1850; **Location:** waterBody: Pacific; stateProvince: Clarion Clipperton Zone; locality: UK Seabed Resources Ltd exploration area UK-1 Stratum B; verbatimLocality: UK1 Stratum B; maximumDepthInMeters: 4425; locationRemarks: Deployment EB02; at Station U5; from R/V Thomas G. Thompson Cruise no. TN319; verbatimLatitude: 12'32.23; verbatimLongitude: 116'36.25; decimalLatitude: 12.53717; decimalLongitude: -116.60417; geodeticDatum: WGS84; **Identification:** identifiedBy: Helena Wiklund | Lenka Neal | Thomas Dahlgren | Adrian Glover | Madeleine Brasier | Regan Drennan | Eva Stewart; dateIdentified: 2021-04-20; identificationRemarks: identified by DNA and morphology; **Event:** eventID: UK1_AB02_EB02; samplingProtocol: Brenke Epibenthic Sledge; eventDate: 2015-02-20; eventTime: 06:24; habitat: Abyssal plain; fieldNotes: Collected from epi net (on the epibenthic sledge); **Record Level:** language: en; institutionCode: NHMUK; collectionCode: ZOO; datasetName: ABYSSLINE; basisOfRecord: PreservedSpecimen
- **Type status:** Other material. **Occurrence:** catalogNumber: NHMUK ANEA 2023.396; recordNumber: NHM_0752; recordedBy: Adrian Glover | Helena Wiklund | Thomas Dahlgren | Madeleine Brasier; individualCount: 1; preparations: specimen stored in 80% non-denatured ethanol aqueous solution | DNA voucher stored in buffer; otherCatalogNumbers: 0174126573; associatedSequences: OQ746552 (16S); occurrenceID: 341CF4EF-9BE7-5996-8CB6-FD57F39E4531; **Taxon:** taxonConceptID: Glyceridae sp. (NHM_207); scientificName: Glyceridae; kingdom: Animalia; phylum: Annelida; class: Polychaeta; order: Phyllodocida; family: Glyceridae; taxonRank: family; scientificNameAuthorship: Grube, 1850; **Location:** waterBody: Pacific; stateProvince: Clarion Clipperton Zone; locality: UK Seabed Resources Ltd exploration area UK-1 Stratum B; verbatimLocality: UK1 Stratum B; maximumDepthInMeters: 4425; locationRemarks: Deployment EB02; at Station U5; from R/V Thomas G. Thompson Cruise no. TN319; verbatimLatitude: 12'32.23; verbatimLongitude: 116'36.25; decimalLatitude: 12.53717; decimalLongitude: -116.60417; geodeticDatum: WGS84; **Identification:** identifiedBy: Helena Wiklund | Lenka Neal | Thomas Dahlgren | Adrian Glover | Madeleine Brasier | Regan Drennan | Eva Stewart; dateIdentified: 2021-04-20; identificationRemarks: identified by DNA and morphology; **Event:** eventID: UK1_AB02_EB02; samplingProtocol: Brenke Epibenthic Sledge; eventDate: 2015-02-20; eventTime: 06:24; habitat: Abyssal plain; fieldNotes: Collected from epi net (on the epibenthic sledge); **Record Level:** language: en; institutionCode: NHMUK; collectionCode: ZOO; datasetName: ABYSSLINE; basisOfRecord: PreservedSpecimen
- **Type status:** Other material. **Occurrence:** catalogNumber: NHMUK ANEA 2023.394; recordNumber: NHM_0445; recordedBy: Adrian Glover | Helena Wiklund | Thomas Dahlgren | Magdalena Georgieva; individualCount: 1; preparations: specimen stored in 80% non-denatured ethanol aqueous solution | DNA voucher stored in buffer; otherCatalogNumbers: 0174127364; associatedSequences: OQ746520 (16S) | OQ746836 (18S); occurrenceID: 96B6D0BA-0B0A-511D-837B-9681A6CB94A8; **Taxon:** taxonConceptID: Glyceridae sp. (NHM_207); scientificName: Glyceridae; kingdom: Animalia; phylum: Annelida; class: Polychaeta; order: Phyllodocida; family: Glyceridae; taxonRank: family; scientificNameAuthorship: Grube, 1850; **Location:** waterBody: Pacific; stateProvince: Clarion Clipperton Zone; locality: UK Seabed Resources Ltd exploration area UK-1 Stratum A; verbatimLocality: UK1 Stratum A; maximumDepthInMeters: 4163; locationRemarks: Deployment BC13; at Station J; from R/V Melville Cruise no. MV1313; verbatimLatitude: 13°54.099; verbatimLongitude: 116°35.400; decimalLatitude: 13.90165; decimalLongitude: -116.59; geodeticDatum: WGS84; **Identification:** identifiedBy: Helena Wiklund | Lenka Neal | Thomas Dahlgren | Adrian Glover | Madeleine Brasier | Regan Drennan | Eva Stewart; dateIdentified: 2021-04-20; identificationRemarks: identified by DNA and morphology; **Event:** eventID: UK1_AB01_BC13; samplingProtocol: USNEL Box Core; eventDate: 2013-10-21; eventTime: 13:27; habitat: Abyssal plain; fieldNotes: Collected from 0-2 cm layer of box core using a 300 micron sieve; **Record Level:** language: en; institutionCode: NHMUK; collectionCode: ZOO; datasetName: ABYSSLINE; basisOfRecord: PreservedSpecimen
- **Type status:** Other material. **Occurrence:** catalogNumber: NHMUK ANEA 2023.391; recordNumber: NHM_0207; recordedBy: Adrian Glover | Helena Wiklund | Thomas Dahlgren | Magdalena Georgieva; individualCount: 1; preparations: specimen stored in 80% non-denatured ethanol aqueous solution | DNA voucher stored in buffer; otherCatalogNumbers: 0174127298; associatedSequences: OQ746486 (16S) | OQ746807 (18S); occurrenceID: 03F29E22-E422-506E-8A95-1C6E4966D1F3; **Taxon:** taxonConceptID: Glyceridae sp. (NHM_207); scientificName: Glyceridae; kingdom: Animalia; phylum: Annelida; class: Polychaeta; order: Phyllodocida; family: Glyceridae; taxonRank: family; scientificNameAuthorship: Grube, 1850; **Location:** waterBody: Pacific; stateProvince: Clarion Clipperton Zone; locality: UK Seabed Resources Ltd exploration area UK-1 Stratum A; verbatimLocality: UK1 Stratum A; maximumDepthInMeters: 4054; locationRemarks: Deployment BC07; at Station E; from R/V Melville Cruise no. MV1313; verbatimLatitude: 13°49.447; verbatimLongitude: 116°32.055; decimalLatitude: 13.82412; decimalLongitude: -116.53425; geodeticDatum: WGS84; **Identification:** identifiedBy: Helena Wiklund | Lenka Neal | Thomas Dahlgren | Adrian Glover | Madeleine Brasier | Regan Drennan | Eva Stewart; dateIdentified: 2021-04-20; identificationRemarks: identified by DNA and morphology; **Event:** eventID: UK1_AB01_BC07; samplingProtocol: USNEL Box Core; eventDate: 2013-10-14; eventTime: 21:37; habitat: Abyssal plain; fieldNotes: Collected from 0-2 cm layer of box core using a 300 micron sieve; **Record Level:** language: en; institutionCode: NHMUK; collectionCode: ZOO; datasetName: ABYSSLINE; basisOfRecord: PreservedSpecimen

##### Distribution

Eastern Clarion-Clipperton Zone, central Pacific Ocean.

##### Diagnosis

**Figure 44.**
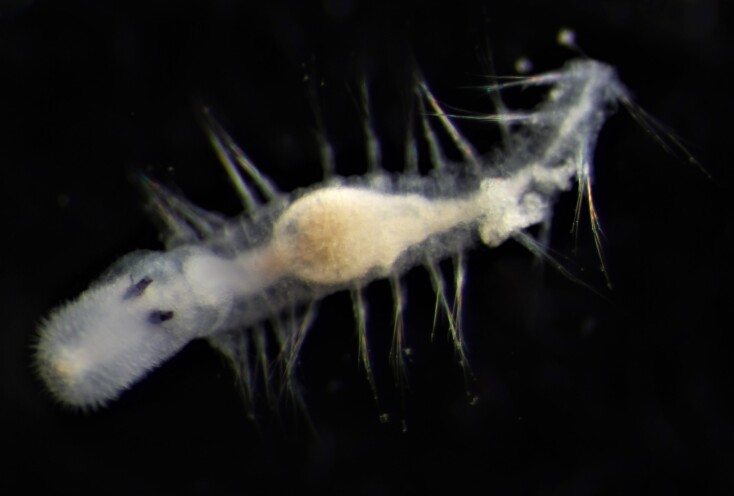
Glyceridae sp. (NHM_207), anterior end of live specimen NHM_359 with extended proboscis.

Damaged specimen (Fig. 44) consistent with placement within family Glyceridae, based on morphology and DNA.

#### Glyceridae sp. (NHM_1242)

39887A9D-AA3F-5B0D-BAF8-F51E53A5BBDF

##### Materials

- **Type status:** Other material. **Occurrence:** catalogNumber: NHMUK ANEA 2023.389; recordNumber: NHM_1242; recordedBy: Adrian Glover | Helena Wiklund | Thomas Dahlgren | Madeleine Brasier; individualCount: 1; preparations: specimen stored in 80% non-denatured ethanol aqueous solution | DNA voucher stored in buffer; otherCatalogNumbers: 0174126782; associatedSequences: OQ746644 (16S) | OQ746883 (18S); occurrenceID: CCBEDF76-C1E5-5A1C-9CAF-233DE8F83D2C; **Taxon:** taxonConceptID: Glyceridae sp. (NHM_1242); scientificName: Glyceridae; kingdom: Animalia; phylum: Annelida; class: Polychaeta; order: Phyllodocida; family: Glyceridae; taxonRank: family; scientificNameAuthorship: Grube, 1850; **Location:** waterBody: Pacific; stateProvince: Clarion Clipperton Zone; locality: Ocean Mineral Singapore exploration claim Stratum A; verbatimLocality: OMS Stratum A; maximumDepthInMeters: 4302; locationRemarks: Deployment EB06; at Station S5; from R/V Thomas G. Thompson Cruise no. TN319; verbatimLatitude: 12'15.44; verbatimLongitude: 117'18.13; decimalLatitude: 12.25733; decimalLongitude: -117.30217; geodeticDatum: WGS84; **Identification:** identifiedBy: Helena Wiklund | Lenka Neal | Thomas Dahlgren | Adrian Glover | Madeleine Brasier | Regan Drennan | Eva Stewart; dateIdentified: 2021-04-20; identificationRemarks: identified by DNA and morphology; **Event:** eventID: OMS1_AB02_EB06; samplingProtocol: Brenke Epibenthic Sledge; eventDate: 2015-03-01; eventTime: 04:02; habitat: Abyssal plain; fieldNotes: Collected from epi net (on the epibenthic sledge); **Record Level:** language: en; institutionCode: NHMUK; collectionCode: ZOO; datasetName: ABYSSLINE; basisOfRecord: PreservedSpecimen
- **Type status:** Other material. **Occurrence:** recordNumber: NHM_1348D; recordedBy: Adrian Glover | Helena Wiklund | Thomas Dahlgren | Madeleine Brasier; individualCount: 1; preparations: Tissue voucher stored in 80% non-denatured ethanol aqueous solution | DNA voucher stored in buffer; otherCatalogNumbers: 0109405381 | 0174126612; associatedSequences: OQ746667 (16S); occurrenceID: 0D6FBE6A-270D-58F9-9082-A16B5D730676; **Taxon:** taxonConceptID: Glyceridae sp. (NHM_1242); scientificName: Glyceridae; kingdom: Animalia; phylum: Annelida; class: Polychaeta; order: Phyllodocida; family: Glyceridae; taxonRank: family; scientificNameAuthorship: Grube, 1850; **Location:** waterBody: Pacific; stateProvince: Clarion Clipperton Zone; locality: Ocean Mineral Singapore exploration claim Stratum A; verbatimLocality: OMS Stratum A; maximumDepthInMeters: 4302; locationRemarks: Deployment EB06; at Station S5; from R/V Thomas G. Thompson Cruise no. TN319; verbatimLatitude: 12'15.44; verbatimLongitude: 117'18.13; decimalLatitude: 12.25733; decimalLongitude: -117.30217; geodeticDatum: WGS84; **Identification:** identifiedBy: Helena Wiklund | Lenka Neal | Thomas Dahlgren | Adrian Glover | Madeleine Brasier | Regan Drennan | Eva Stewart; dateIdentified: 2021-04-20; identificationRemarks: identified by DNA and morphology; **Event:** eventID: OMS1_AB02_EB06; samplingProtocol: Brenke Epibenthic Sledge; eventDate: 2015-03-01; eventTime: 04:02; habitat: Abyssal plain; fieldNotes: Collected from epi net (on the epibenthic sledge); **Record Level:** language: en; institutionCode: NHMUK; collectionCode: ZOO; datasetName: ABYSSLINE; basisOfRecord: PreservedSpecimen
- **Type status:** Other material. **Occurrence:** catalogNumber: NHMUK ANEA 2023.390; recordNumber: NHM_1579; recordedBy: Adrian Glover | Helena Wiklund | Thomas Dahlgren | Madeleine Brasier; individualCount: 1; preparations: specimen stored in 80% non-denatured ethanol aqueous solution | DNA voucher stored in buffer; otherCatalogNumbers: 0174126777; associatedSequences: OQ746696 (16S); occurrenceID: CBF3AD4C-376E-508C-934D-FE55368605E3; **Taxon:** taxonConceptID: Glyceridae sp. (NHM_1242); scientificName: Glyceridae; kingdom: Animalia; phylum: Annelida; class: Polychaeta; order: Phyllodocida; family: Glyceridae; taxonRank: family; scientificNameAuthorship: Grube, 1850; **Location:** waterBody: Pacific; stateProvince: Clarion Clipperton Zone; locality: UK Seabed Resources Ltd exploration area UK-1 Stratum B; verbatimLocality: UK1 Stratum B; maximumDepthInMeters: 4136; locationRemarks: Deployment BC18; at Station U12; from R/V Thomas G. Thompson Cruise no. TN319; verbatimLatitude: 12'25.195; verbatimLongitude: 116'37.477; decimalLatitude: 12.41992; decimalLongitude: -116.62462; geodeticDatum: WGS84; **Identification:** identifiedBy: Helena Wiklund | Lenka Neal | Thomas Dahlgren | Adrian Glover | Madeleine Brasier | Regan Drennan | Eva Stewart; dateIdentified: 2021-04-20; identificationRemarks: identified by DNA and morphology; **Event:** eventID: UK1_AB02_BC18; samplingProtocol: USNEL Box Core; eventDate: 2015-03-07; habitat: Abyssal plain; fieldNotes: Collected from 0-2 cm layer of box core using a 300 micron sieve; **Record Level:** language: en; institutionCode: NHMUK; collectionCode: ZOO; datasetName: ABYSSLINE; basisOfRecord: PreservedSpecimen

##### Distribution

Eastern Clarion-Clipperton Zone, central Pacific Ocean.

##### Diagnosis

**Figure 45.**
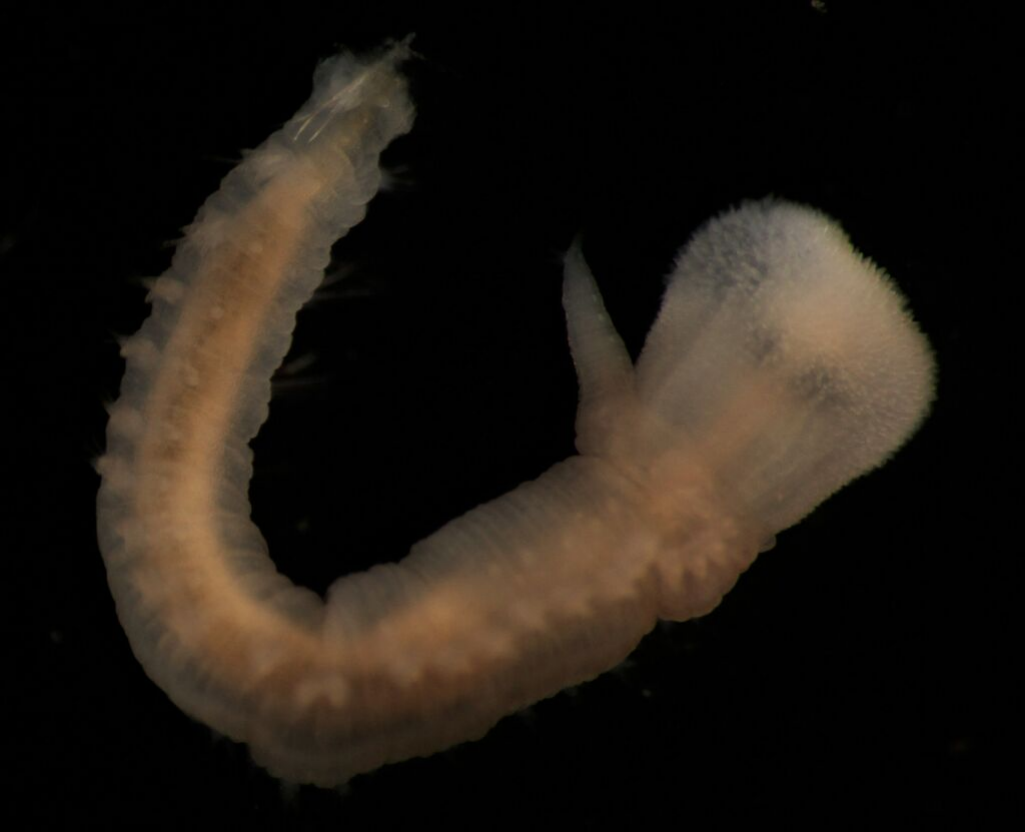
Glyceridae sp. (NHM_1242), anterior fragment of live specimen NHM_1242 in lateral view with extended proboscis.

Damaged specimens (Fig. 45) consistent with placement within family Glyceridae, based on morphology and DNA.

#### Glyceridae sp. (NHM_2089)

82F2BFD2-CE95-5120-A7AF-748952AAAD72

##### Materials

- **Type status:** Other material. **Occurrence:** catalogNumber: NHMUK ANEA 2023.404; recordNumber: NHM_2089; recordedBy: Adrian Glover | Helena Wiklund | Thomas Dahlgren | Madeleine Brasier; individualCount: 1; preparations: specimen stored in 80% non-denatured ethanol aqueous solution | DNA voucher stored in buffer; otherCatalogNumbers: 0174126773; associatedSequences: OQ746748 (16S); occurrenceID: 09E6B5BB-7E7B-57D4-B765-B41C2DF7D37B; **Taxon:** taxonConceptID: Glyceridae sp. (NHM_2089); scientificName: Glyceridae; kingdom: Animalia; phylum: Annelida; class: Polychaeta; order: Phyllodocida; family: Glyceridae; taxonRank: family; scientificNameAuthorship: Grube, 1850; **Location:** waterBody: Pacific; stateProvince: Clarion Clipperton Zone; locality: Area of Particular Interest APEI-6; verbatimLocality: APEI-6; maximumDepthInMeters: 4026; locationRemarks: Deployment EB13; at Station APEI; from R/V Thomas G. Thompson Cruise no. TN319; verbatimLatitude: 19 27.874; verbatimLongitude: 120 01.525; decimalLatitude: 19.46457; decimalLongitude: -120.02542; geodeticDatum: WGS84; **Identification:** identifiedBy: Helena Wiklund | Lenka Neal | Thomas Dahlgren | Adrian Glover | Madeleine Brasier | Regan Drennan | Eva Stewart; dateIdentified: 2021-04-20; identificationRemarks: identified by DNA and morphology; **Event:** eventID: APEI6_AB02_EB13; samplingProtocol: Brenke Epibenthic Sledge; eventDate: 2015-03-20; eventTime: 16:12; habitat: Abyssal plain; fieldNotes: Collected from epi net (on the epibenthic sledge); **Record Level:** language: en; institutionCode: NHMUK; collectionCode: ZOO; datasetName: ABYSSLINE; basisOfRecord: PreservedSpecimen
- **Type status:** Other material. **Occurrence:** catalogNumber: NHMUK ANEA 2023.405; recordNumber: NHM_2096; recordedBy: Adrian Glover | Helena Wiklund | Thomas Dahlgren | Madeleine Brasier; individualCount: 1; preparations: specimen stored in 80% non-denatured ethanol aqueous solution | DNA voucher stored in buffer; otherCatalogNumbers: 0174126738; associatedSequences: OQ746751 (16S) | OQ746904 (18S) | OQ738606 (COI); occurrenceID: 42C31FE6-550C-52F2-900D-972C8EEB50F7; **Taxon:** taxonConceptID: Glyceridae sp. (NHM_2089); scientificName: Glyceridae; kingdom: Animalia; phylum: Annelida; class: Polychaeta; order: Phyllodocida; family: Glyceridae; taxonRank: family; scientificNameAuthorship: Grube, 1850; **Location:** waterBody: Pacific; stateProvince: Clarion Clipperton Zone; locality: Area of Particular Interest APEI-6; verbatimLocality: APEI-6; maximumDepthInMeters: 4026; locationRemarks: Deployment EB13; at Station APEI; from R/V Thomas G. Thompson Cruise no. TN319; verbatimLatitude: 19 27.874; verbatimLongitude: 120 01.525; decimalLatitude: 19.46457; decimalLongitude: -120.02542; geodeticDatum: WGS84; **Identification:** identifiedBy: Helena Wiklund | Lenka Neal | Thomas Dahlgren | Adrian Glover | Madeleine Brasier | Regan Drennan | Eva Stewart; dateIdentified: 2021-04-20; identificationRemarks: identified by DNA and morphology; **Event:** eventID: APEI6_AB02_EB13; samplingProtocol: Brenke Epibenthic Sledge; eventDate: 2015-03-20; eventTime: 16:12; habitat: Abyssal plain; fieldNotes: Collected from epi net (on the epibenthic sledge); **Record Level:** language: en; institutionCode: NHMUK; collectionCode: ZOO; datasetName: ABYSSLINE; basisOfRecord: PreservedSpecimen

##### Distribution

Eastern Clarion-Clipperton Zone, central Pacific Ocean.

##### Diagnosis

**Figure 46.**
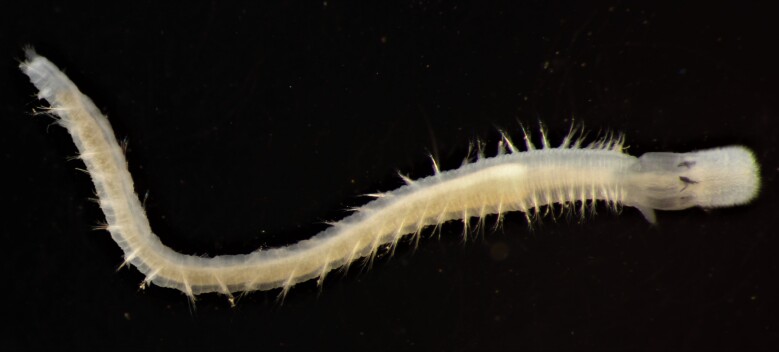
Glyceridae sp. (NHM_2089), anterior fragment of live specimen NHM_2089 in ventral view, with extended proboscis.

Damaged specimens (Fig. 46) consistent with placement within family Glyceridae, based on morphology and DNA.

### Goniadidae Kinberg, 1866

#### Goniadidae sp. (NHM_1512)

50FF9281-626A-5417-B5AC-203AF3FE0DFD

##### Materials

- **Type status:** Other material. **Occurrence:** catalogNumber: NHMUK ANEA 2023.407; recordNumber: NHM_1882; recordedBy: Adrian Glover | Helena Wiklund | Thomas Dahlgren | Madeleine Brasier; individualCount: 1; preparations: specimen stored in 80% non-denatured ethanol aqueous solution | DNA voucher stored in buffer; otherCatalogNumbers: 0174127334; associatedSequences: OQ746902 (18S) | OQ738601 (COI); occurrenceID: 0A082912-7D35-57AC-8AE6-B197F08A41D7; **Taxon:** taxonConceptID: Goniadidae sp. (NHM_1512); scientificName: Goniadidae; kingdom: Animalia; phylum: Annelida; class: Polychaeta; order: Phyllodocida; family: Goniadidae; taxonRank: family; scientificNameAuthorship: Kinberg, 1866; **Location:** waterBody: Pacific; stateProvince: Clarion Clipperton Zone; locality: Ocean Mineral Singapore exploration claim Stratum A; verbatimLocality: OMS Stratum A; maximumDepthInMeters: 4094; locationRemarks: Deployment EB11; at Station S10; from R/V Thomas G. Thompson Cruise no. TN319; verbatimLatitude: 12°02.49’; verbatimLongitude: 117°13.03’; decimalLatitude: 12.0415; decimalLongitude: -117.21717; geodeticDatum: WGS84; **Identification:** identifiedBy: Helena Wiklund | Lenka Neal | Thomas Dahlgren | Adrian Glover | Madeleine Brasier | Regan Drennan | Eva Stewart; dateIdentified: 2021-04-20; identificationRemarks: identified by DNA and morphology; **Event:** eventID: OMS1_AB02_EB11; samplingProtocol: Brenke Epibenthic Sledge; eventDate: 2015-03-13; habitat: Abyssal plain; fieldNotes: Collected from epi net (on the epibenthic sledge); **Record Level:** language: en; institutionCode: NHMUK; collectionCode: ZOO; datasetName: ABYSSLINE; basisOfRecord: PreservedSpecimen
- **Type status:** Other material. **Occurrence:** catalogNumber: NHMUK ANEA 2023.406; recordNumber: NHM_1512; recordedBy: Adrian Glover | Helena Wiklund | Thomas Dahlgren | Madeleine Brasier; individualCount: 1; preparations: specimen stored in 80% non-denatured ethanol aqueous solution | DNA voucher stored in buffer; otherCatalogNumbers: 0174127358; associatedSequences: OQ746893 (18S) | OQ738591 (COI); occurrenceID: E4084B0C-27F0-594B-A27B-AAA5A8DB2187; **Taxon:** taxonConceptID: Goniadidae sp. (NHM_1512); scientificName: Goniadidae; kingdom: Animalia; phylum: Annelida; class: Polychaeta; order: Phyllodocida; family: Goniadidae; taxonRank: family; scientificNameAuthorship: Kinberg, 1866; **Location:** waterBody: Pacific; stateProvince: Clarion Clipperton Zone; locality: UK Seabed Resources Ltd exploration area UK-1 Stratum B; verbatimLocality: UK1 Stratum B; maximumDepthInMeters: 4252; locationRemarks: Deployment EB08; at Station U11; from R/V Thomas G. Thompson Cruise no. TN319; verbatimLatitude: 12'30.79; verbatimLongitude: 116'29.48; decimalLatitude: 12.51317; decimalLongitude: -116.49133; geodeticDatum: WGS84; **Identification:** identifiedBy: Helena Wiklund | Lenka Neal | Thomas Dahlgren | Adrian Glover | Madeleine Brasier | Regan Drennan | Eva Stewart; dateIdentified: 2021-04-20; identificationRemarks: identified by DNA and morphology; **Event:** eventID: UK1_AB02_EB08; samplingProtocol: Brenke Epibenthic Sledge; eventDate: 2015-03-05; eventTime: 18:53; habitat: Abyssal plain; fieldNotes: Collected from epi net (on the epibenthic sledge); **Record Level:** language: en; institutionCode: NHMUK; collectionCode: ZOO; datasetName: ABYSSLINE; basisOfRecord: PreservedSpecimen

##### Distribution

Eastern Clarion-Clipperton Zone, central Pacific Ocean.

##### Diagnosis

**Figure 47.**
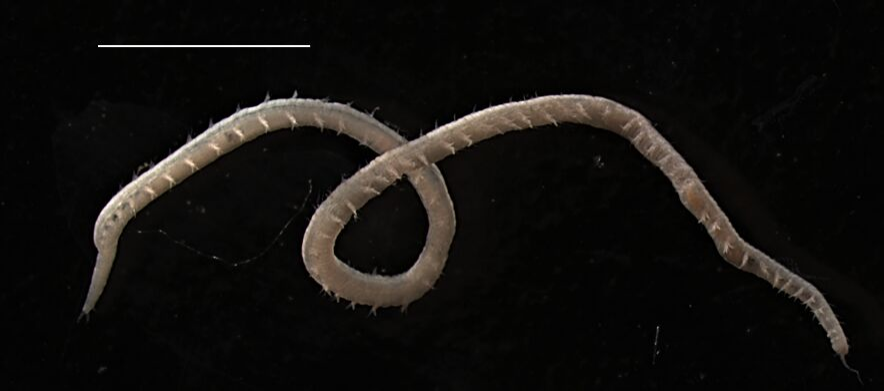
Goniadidae sp. (NHM_1512), complete live specimen NHM_1512 in lateral view. Scale bar 2 mm.

Specimens (Fig. 47) consistent with placement within family Goniadidae, based on morphology and DNA.

### Lacydoniidae Bergström, 1914

#### Lacydoniidae sp. (NHM_898)

7A56BE43-9CC0-5C04-8BB1-E8235B03B4C9

##### Materials

- **Type status:** Other material. **Occurrence:** catalogNumber: NHMUK ANEA 2023.410; recordNumber: NHM_0898; recordedBy: Adrian Glover | Helena Wiklund | Thomas Dahlgren | Madeleine Brasier; individualCount: 1; preparations: specimen stored in 80% non-denatured ethanol aqueous solution | DNA voucher stored in buffer; otherCatalogNumbers: 0174126809; associatedSequences: OQ746571 (16S) | OQ746864 (18S); occurrenceID: 458871D8-966E-5929-9BFE-57FC6870457B; **Taxon:** taxonConceptID: Lacydoniidae sp. (NHM_898); scientificName: Lacydoniidae; kingdom: Animalia; phylum: Annelida; class: Polychaeta; order: Phyllodocida; family: Lacydoniidae; taxonRank: family; scientificNameAuthorship: Bergström, 1914; **Location:** waterBody: Pacific; stateProvince: Clarion Clipperton Zone; locality: UK Seabed Resources Ltd exploration area UK-1 Stratum B; verbatimLocality: UK1 Stratum B; maximumDepthInMeters: 4198; locationRemarks: Deployment EB03; at Station U4; from R/V Thomas G. Thompson Cruise no. TN319; verbatimLatitude: 12'34.28; verbatimLongitude: 116'36.63; decimalLatitude: 12.57133; decimalLongitude: -116.6105; geodeticDatum: WGS84; **Identification:** identifiedBy: Helena Wiklund | Lenka Neal | Thomas Dahlgren | Adrian Glover | Madeleine Brasier | Regan Drennan | Eva Stewart; dateIdentified: 2021-04-20; identificationRemarks: identified by DNA and morphology; **Event:** eventID: UK1_AB02_EB03; samplingProtocol: Brenke Epibenthic Sledge; eventDate: 2015-02-23; eventTime: 05:39; habitat: Abyssal plain; fieldNotes: Collected from epi net (on the epibenthic sledge); **Record Level:** language: en; institutionCode: NHMUK; collectionCode: ZOO; datasetName: ABYSSLINE; basisOfRecord: PreservedSpecimen

##### Distribution

Eastern Clarion-Clipperton Zone, central Pacific Ocean.

##### Diagnosis

**Figure 48.**
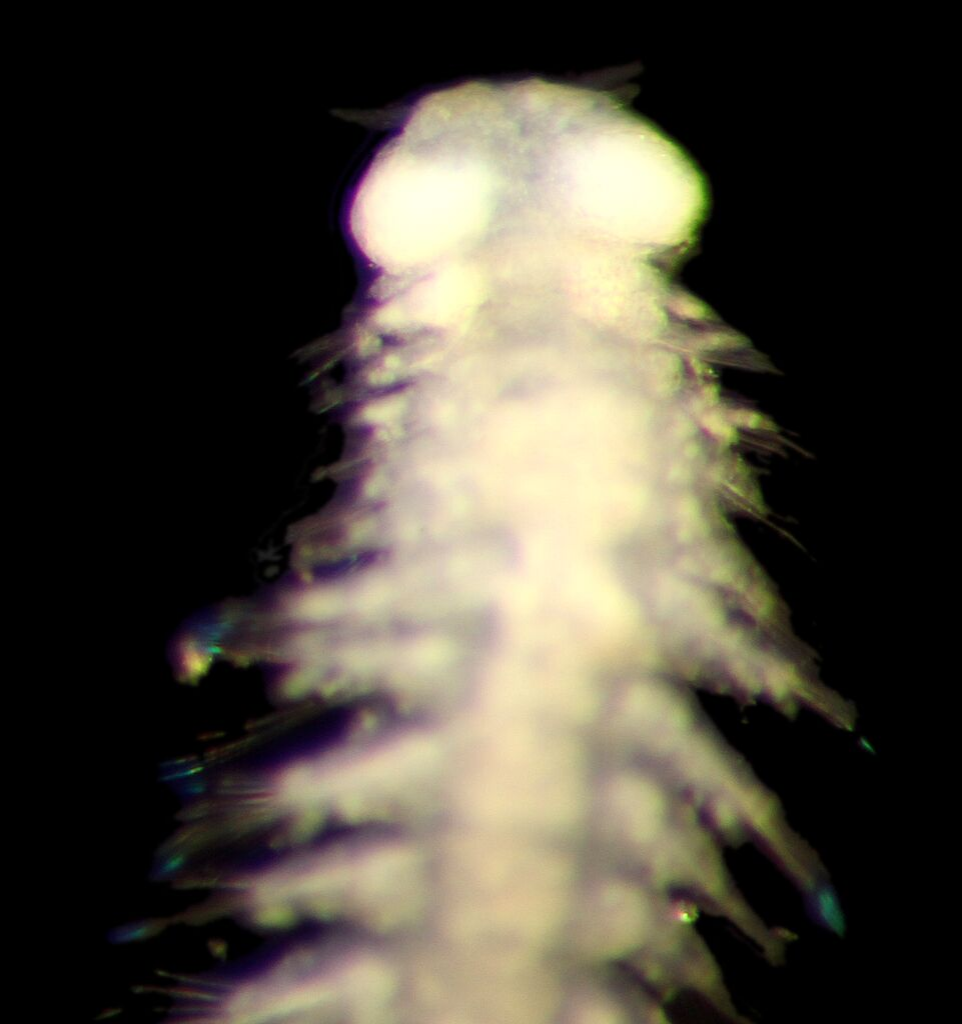
Lacydoniidae sp. (NHM_898), anterior fragment of live specimen NHM_898 in dorsal view.

Damaged specimen (Fig. 48) consistent with placement within family Lacydoniidae, based on morphology and DNA.

#### Lacydoniidae sp. (NHM_1355C)

A75F4256-18BC-52C9-ABBA-3E1186641236

##### Materials

- **Type status:** Other material. **Occurrence:** catalogNumber: NHMUK ANEA 2023.408; recordNumber: NHM_1355C; recordedBy: Adrian Glover | Helena Wiklund | Thomas Dahlgren | Madeleine Brasier; individualCount: 1; preparations: specimen stored in 80% non-denatured ethanol aqueous solution | DNA voucher stored in buffer; otherCatalogNumbers: 0174126216; associatedSequences: OQ746676 (16S); occurrenceID: 2DC2F7A6-9898-538F-BCCB-B3D1FF4595EF; **Taxon:** taxonConceptID: Lacydoniidae sp. (NHM_1355C); scientificName: Lacydoniidae; kingdom: Animalia; phylum: Annelida; class: Polychaeta; order: Phyllodocida; family: Lacydoniidae; taxonRank: family; scientificNameAuthorship: Bergström, 1914; **Location:** waterBody: Pacific; stateProvince: Clarion Clipperton Zone; locality: Ocean Mineral Singapore exploration claim Stratum A; verbatimLocality: OMS Stratum A; maximumDepthInMeters: 4302; locationRemarks: Deployment EB06; at Station S5; from R/V Thomas G. Thompson Cruise no. TN319; verbatimLatitude: 12'15.44; verbatimLongitude: 117'18.13; decimalLatitude: 12.25733; decimalLongitude: -117.30217; geodeticDatum: WGS84; **Identification:** identifiedBy: Helena Wiklund | Lenka Neal | Thomas Dahlgren | Adrian Glover | Madeleine Brasier | Regan Drennan | Eva Stewart; dateIdentified: 2021-04-20; identificationRemarks: identified by DNA and morphology; **Event:** eventID: OMS1_AB02_EB06; samplingProtocol: Brenke Epibenthic Sledge; eventDate: 2015-03-01; eventTime: 04:02; habitat: Abyssal plain; fieldNotes: Collected from epi net (on the epibenthic sledge); **Record Level:** language: en; institutionCode: NHMUK; collectionCode: ZOO; datasetName: ABYSSLINE; basisOfRecord: PreservedSpecimen

##### Distribution

Eastern Clarion-Clipperton Zone, central Pacific Ocean.

##### Diagnosis

**Figure 49.**
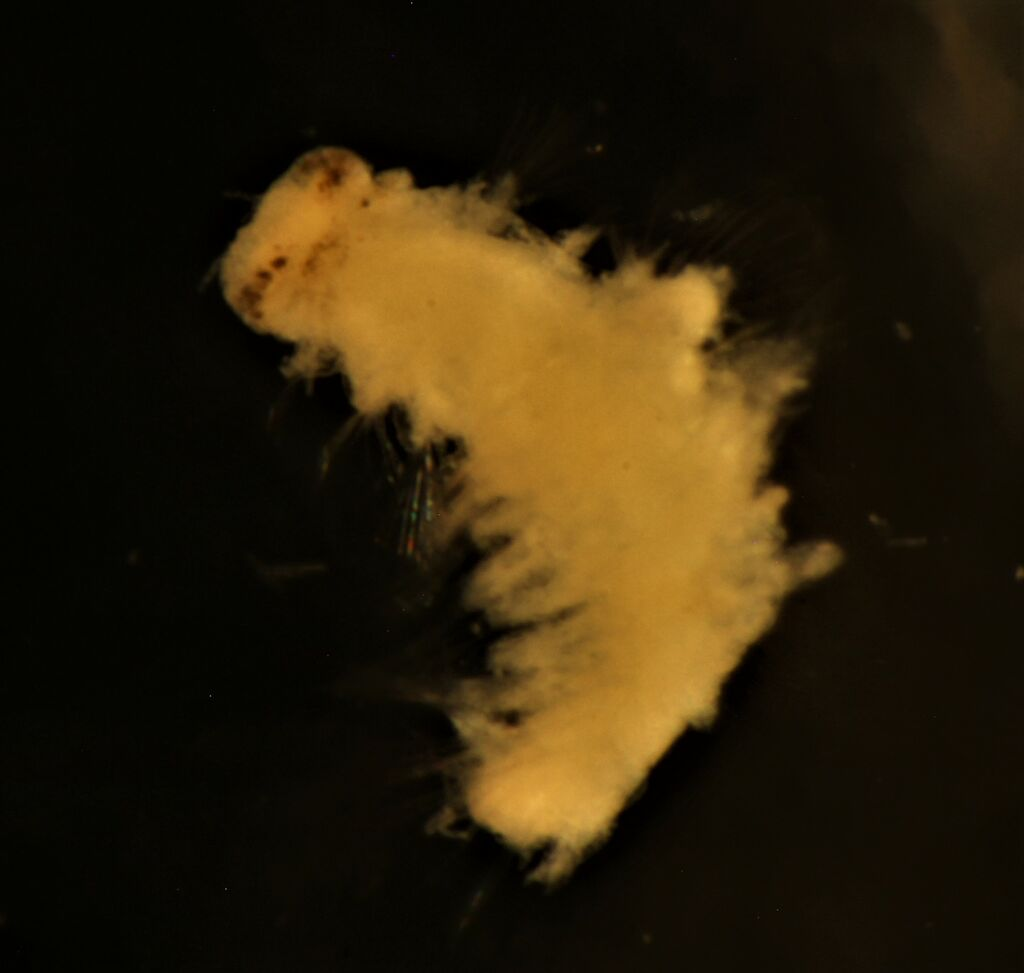
Lacydoniidae sp. (NHM_1355C), anterior fragment of live specimen NHM_1355C in dorsal view.

Damaged specimen (Fig. 49) consistent with placement within family Lacydoniidae, based on morphology and DNA.

#### Lacydoniidae sp. (NHM_1797D)

757B15C3-0330-53CE-87C3-021C78D6E7A2

##### Materials

- **Type status:** Other material. **Occurrence:** catalogNumber: NHMUK ANEA 2023.409; recordNumber: NHM_1797D; recordedBy: Adrian Glover | Helena Wiklund | Thomas Dahlgren | Madeleine Brasier; individualCount: 1; preparations: specimen stored in 80% non-denatured ethanol aqueous solution | DNA voucher stored in buffer; otherCatalogNumbers: 0174126234; associatedSequences: OQ746722 (16S); occurrenceID: 7E3F1A6A-CF53-58BD-B82D-D06F8BD94E0B; **Taxon:** taxonConceptID: Lacydoniidae sp. (NHM_1797D); scientificName: Lacydoniidae; kingdom: Animalia; phylum: Annelida; class: Polychaeta; order: Phyllodocida; family: Lacydoniidae; taxonRank: family; scientificNameAuthorship: Bergström, 1914; **Location:** waterBody: Pacific; stateProvince: Clarion Clipperton Zone; locality: Ocean Mineral Singapore exploration claim Stratum A; verbatimLocality: OMS Stratum A; maximumDepthInMeters: 4045; locationRemarks: Deployment EB10; at Station S7; from R/V Thomas G. Thompson Cruise no. TN319; verbatimLatitude: 12'10.43; verbatimLongitude: 117'11.57; decimalLatitude: 12.17383; decimalLongitude: -117.19283; geodeticDatum: WGS84; **Identification:** identifiedBy: Helena Wiklund | Lenka Neal | Thomas Dahlgren | Adrian Glover | Madeleine Brasier | Regan Drennan | Eva Stewart; dateIdentified: 2021-04-20; identificationRemarks: identified by DNA and morphology; **Event:** eventID: OMS1_AB02_EB10; samplingProtocol: Brenke Epibenthic Sledge; eventDate: 2015-03-11; eventTime: 22:49; habitat: Abyssal plain; fieldNotes: Collected from epi net (on the epibenthic sledge); **Record Level:** language: en; institutionCode: NHMUK; collectionCode: ZOO; datasetName: ABYSSLINE; basisOfRecord: PreservedSpecimen

##### Distribution

Eastern Clarion-Clipperton Zone, central Pacific Ocean.

##### Diagnosis

**Figure 50.**
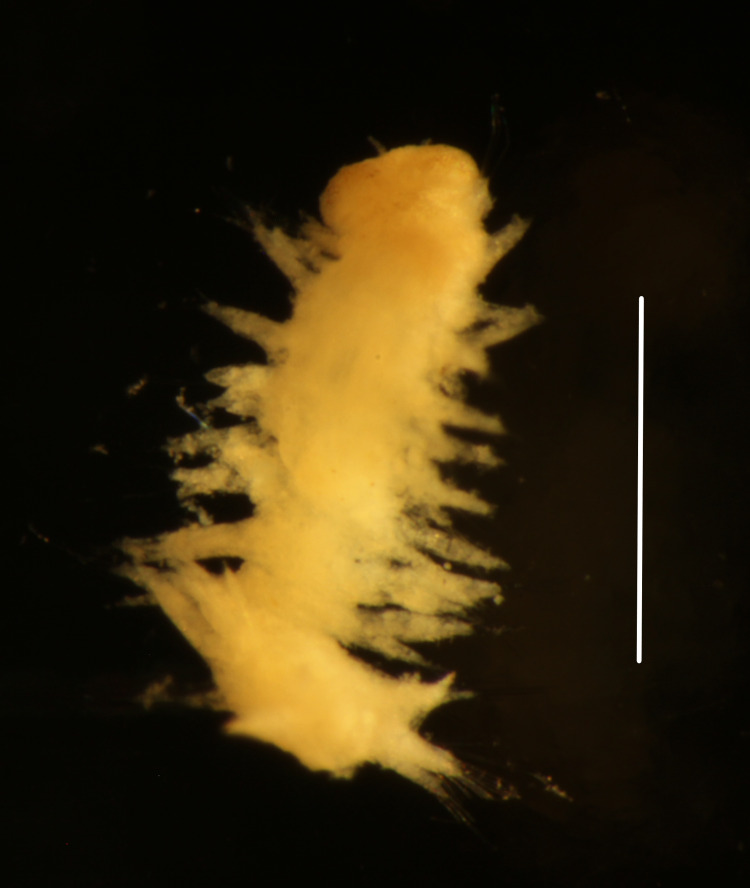
Lacydoniidae sp. (NHM_1797D), anterior fragment of live specimen NHM_1797D in dorsal view. Scale bar 1 mm.

Damaged specimen (Fig. 50) consistent with placement within family Lacydoniidae, based on morphology and DNA.

### Magelonidae Cunningham & Ramage, 1888

#### Magelonidae sp. (NHM_1340)

6A070C81-C170-5A82-BA70-7E1A607E224A

##### Materials

- **Type status:** Other material. **Occurrence:** catalogNumber: NHMUK ANEA 2023.411; recordNumber: NHM_1340; recordedBy: Adrian Glover | Helena Wiklund | Thomas Dahlgren | Madeleine Brasier; individualCount: 1; preparations: specimen stored in 80% non-denatured ethanol aqueous solution | DNA voucher stored in buffer; otherCatalogNumbers: 0174126312; associatedSequences: OQ746663 (16S) | OQ746891 (18S); occurrenceID: D7AB891A-A74A-5C3B-BD2B-6784F223AFC3; **Taxon:** taxonConceptID: Magelonidae sp. (NHM_1340); scientificName: Magelonidae; kingdom: Animalia; phylum: Annelida; class: Polychaeta; family: Magelonidae; taxonRank: family; scientificNameAuthorship: Cunningham & Ramage, 1888; **Location:** waterBody: Pacific; stateProvince: Clarion Clipperton Zone; locality: Ocean Mineral Singapore exploration claim Stratum A; verbatimLocality: OMS Stratum A; maximumDepthInMeters: 4302; locationRemarks: Deployment EB06; at Station S5; from R/V Thomas G. Thompson Cruise no. TN319; verbatimLatitude: 12'15.44; verbatimLongitude: 117'18.13; decimalLatitude: 12.25733; decimalLongitude: -117.30217; geodeticDatum: WGS84; **Identification:** identifiedBy: Helena Wiklund | Lenka Neal | Thomas Dahlgren | Adrian Glover | Madeleine Brasier | Regan Drennan | Eva Stewart; dateIdentified: 2021-04-20; identificationRemarks: identified by DNA and morphology; **Event:** eventID: OMS1_AB02_EB06; samplingProtocol: Brenke Epibenthic Sledge; eventDate: 2015-03-01; eventTime: 04:02; habitat: Abyssal plain; fieldNotes: Collected from epi net (on the epibenthic sledge); **Record Level:** language: en; institutionCode: NHMUK; collectionCode: ZOO; datasetName: ABYSSLINE; basisOfRecord: PreservedSpecimen
- **Type status:** Other material. **Occurrence:** catalogNumber: NHMUK ANEA 2023.412; recordNumber: NHM_2412; recordedBy: Adrian Glover | Helena Wiklund | Thomas Dahlgren | Madeleine Brasier; individualCount: 1; preparations: specimen stored in 80% non-denatured ethanol aqueous solution | DNA voucher stored in buffer; otherCatalogNumbers: 0174126151; associatedSequences: OQ746773 (16S) | OQ738612 (COI); occurrenceID: 31C68758-3181-5CD6-AC2C-557FEA2B1C3C; **Taxon:** taxonConceptID: Magelonidae sp. (NHM_1340); scientificName: Magelonidae; kingdom: Animalia; phylum: Annelida; class: Polychaeta; family: Magelonidae; taxonRank: family; scientificNameAuthorship: Cunningham & Ramage, 1888; **Location:** waterBody: Pacific; stateProvince: Clarion Clipperton Zone; locality: UK Seabed Resources Ltd exploration area UK-1 Stratum B; verbatimLocality: UK1 Stratum B; maximumDepthInMeters: 4425; locationRemarks: Deployment EB02; at Station U5; from R/V Thomas G. Thompson Cruise no. TN319; verbatimLatitude: 12'32.23; verbatimLongitude: 116'36.25; decimalLatitude: 12.53717; decimalLongitude: -116.60417; geodeticDatum: WGS84; **Identification:** identifiedBy: Helena Wiklund | Lenka Neal | Thomas Dahlgren | Adrian Glover | Madeleine Brasier | Regan Drennan | Eva Stewart; dateIdentified: 2021-04-20; identificationRemarks: identified by DNA and morphology; **Event:** eventID: UK1_AB02_EB02; samplingProtocol: Brenke Epibenthic Sledge; eventDate: 2015-02-20; eventTime: 06:24; habitat: Abyssal plain; fieldNotes: Collected from supra net (on the epibenthic sledge); **Record Level:** language: en; institutionCode: NHMUK; collectionCode: ZOO; datasetName: ABYSSLINE; basisOfRecord: PreservedSpecimen

##### Distribution

Eastern Clarion-Clipperton Zone, central Pacific Ocean.

##### Diagnosis

**Figure 51.**
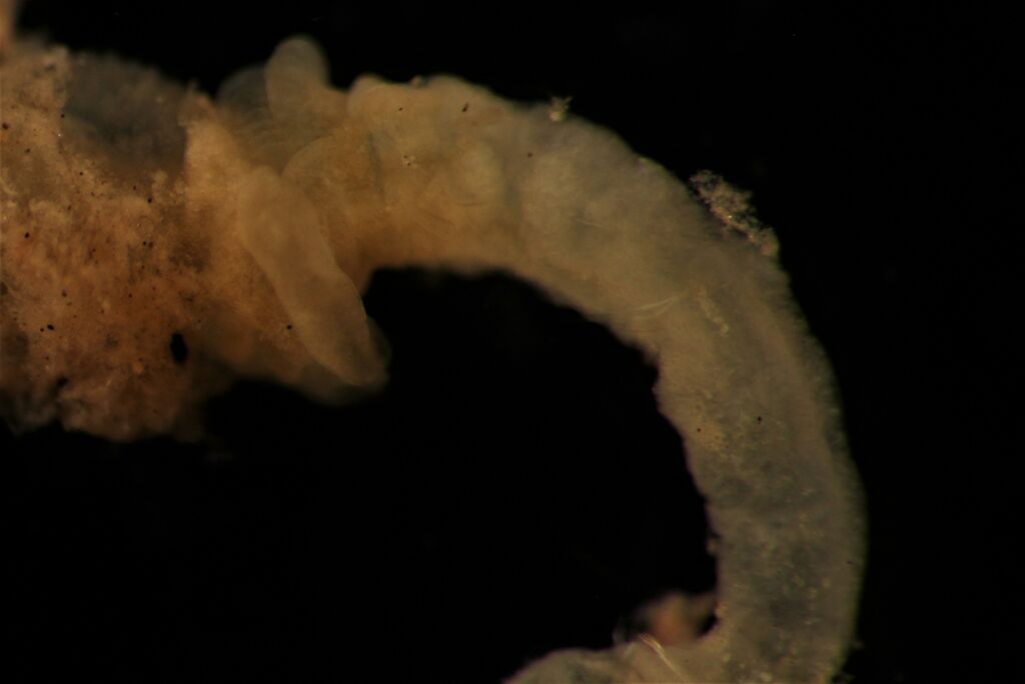
Magelonidae sp. (NHM_1340), fragment of live specimen NHM_1340.

Damaged specimens (Fig. 51) consistent with placement within family Magelonidae, based on morphology and DNA.

### Maldanidae Malmgren, 1867

#### Maldanidae sp. (NHM_026)

D036A4CE-B6EA-5FF8-8E66-C79B425AAE79

##### Materials

- **Type status:** Other material. **Occurrence:** recordNumber: NHM_2016; recordedBy: Adrian Glover | Helena Wiklund | Thomas Dahlgren | Madeleine Brasier; individualCount: 1; preparations: DNA voucher stored in buffer; otherCatalogNumbers: 0174126765; associatedSequences: OQ746744 (16S); occurrenceID: 9F9B05E6-6020-5732-9198-93600C04AEEB; **Taxon:** taxonConceptID: Maldanidae sp. (NHM_026); scientificName: Maldanidae; kingdom: Animalia; phylum: Annelida; class: Polychaeta; family: Maldanidae; taxonRank: family; scientificNameAuthorship: Malmgren, 1867; **Location:** waterBody: Pacific; stateProvince: Clarion Clipperton Zone; locality: Ocean Mineral Singapore exploration claim Stratum A; verbatimLocality: OMS Stratum A; maximumDepthInMeters: 4235; locationRemarks: Deployment EB12; at Station S11; from R/V Thomas G. Thompson Cruise no. TN319; verbatimLatitude: 12'03.03; verbatimLongitude: 117'24.28; decimalLatitude: 12.0505; decimalLongitude: -117.40467; geodeticDatum: WGS84; **Identification:** identifiedBy: Helena Wiklund | Lenka Neal | Thomas Dahlgren | Adrian Glover | Madeleine Brasier | Regan Drennan | Eva Stewart; dateIdentified: 2021-04-20; identificationRemarks: identified by DNA and morphology; **Event:** eventID: OMS1_AB02_EB12; samplingProtocol: Brenke Epibenthic Sledge; eventDate: 2015-03-16; eventTime: 05:30; habitat: Abyssal plain; fieldNotes: Collected from epi net (on the epibenthic sledge); **Record Level:** language: en; institutionCode: NHMUK; collectionCode: ZOO; datasetName: ABYSSLINE; basisOfRecord: PreservedSpecimen
- **Type status:** Other material. **Occurrence:** catalogNumber: NHMUK ANEA 2023.416; recordNumber: NHM_2021; recordedBy: Adrian Glover | Helena Wiklund | Thomas Dahlgren | Madeleine Brasier; individualCount: 1; preparations: specimen stored in 80% non-denatured ethanol aqueous solution | DNA voucher stored in buffer; otherCatalogNumbers: 0174126746; associatedSequences: OQ746745 (16S); occurrenceID: E3B5A6BA-8A77-554A-9BBB-C83AFCE4F3AA; **Taxon:** taxonConceptID: Maldanidae sp. (NHM_026); scientificName: Maldanidae; kingdom: Animalia; phylum: Annelida; class: Polychaeta; family: Maldanidae; taxonRank: family; scientificNameAuthorship: Malmgren, 1867; **Location:** waterBody: Pacific; stateProvince: Clarion Clipperton Zone; locality: Ocean Mineral Singapore exploration claim Stratum A; verbatimLocality: OMS Stratum A; maximumDepthInMeters: 4235; locationRemarks: Deployment EB12; at Station S11; from R/V Thomas G. Thompson Cruise no. TN319; verbatimLatitude: 12'03.03; verbatimLongitude: 117'24.28; decimalLatitude: 12.0505; decimalLongitude: -117.40467; geodeticDatum: WGS84; **Identification:** identifiedBy: Helena Wiklund | Lenka Neal | Thomas Dahlgren | Adrian Glover | Madeleine Brasier | Regan Drennan | Eva Stewart; dateIdentified: 2021-04-20; identificationRemarks: identified by DNA and morphology; **Event:** eventID: OMS1_AB02_EB12; samplingProtocol: Brenke Epibenthic Sledge; eventDate: 2015-03-16; eventTime: 05:30; habitat: Abyssal plain; fieldNotes: Collected from epi net (on the epibenthic sledge); **Record Level:** language: en; institutionCode: NHMUK; collectionCode: ZOO; datasetName: ABYSSLINE; basisOfRecord: PreservedSpecimen
- **Type status:** Other material. **Occurrence:** catalogNumber: NHMUK ANEA 2023.417; recordNumber: NHM_2022; recordedBy: Adrian Glover | Helena Wiklund | Thomas Dahlgren | Madeleine Brasier; individualCount: 1; preparations: specimen stored in 80% non-denatured ethanol aqueous solution | DNA voucher stored in buffer; otherCatalogNumbers: 0174126741; associatedSequences: OQ746746 (16S); occurrenceID: A4B1F9F7-40FE-59A4-8821-872786879FFC; **Taxon:** taxonConceptID: Maldanidae sp. (NHM_026); scientificName: Maldanidae; kingdom: Animalia; phylum: Annelida; class: Polychaeta; family: Maldanidae; taxonRank: family; scientificNameAuthorship: Malmgren, 1867; **Location:** waterBody: Pacific; stateProvince: Clarion Clipperton Zone; locality: Ocean Mineral Singapore exploration claim Stratum A; verbatimLocality: OMS Stratum A; maximumDepthInMeters: 4235; locationRemarks: Deployment EB12; at Station S11; from R/V Thomas G. Thompson Cruise no. TN319; verbatimLatitude: 12'03.03; verbatimLongitude: 117'24.28; decimalLatitude: 12.0505; decimalLongitude: -117.40467; geodeticDatum: WGS84; **Identification:** identifiedBy: Helena Wiklund | Lenka Neal | Thomas Dahlgren | Adrian Glover | Madeleine Brasier | Regan Drennan | Eva Stewart; dateIdentified: 2021-04-20; identificationRemarks: identified by DNA and morphology; **Event:** eventID: OMS1_AB02_EB12; samplingProtocol: Brenke Epibenthic Sledge; eventDate: 2015-03-16; eventTime: 05:30; habitat: Abyssal plain; fieldNotes: Collected from epi net (on the epibenthic sledge); **Record Level:** language: en; institutionCode: NHMUK; collectionCode: ZOO; datasetName: ABYSSLINE; basisOfRecord: PreservedSpecimen
- **Type status:** Other material. **Occurrence:** catalogNumber: NHMUK ANEA 2023.414; recordNumber: NHM_1350H; recordedBy: Adrian Glover | Helena Wiklund | Thomas Dahlgren | Madeleine Brasier; individualCount: 1; preparations: specimen stored in 80% non-denatured ethanol aqueous solution | DNA voucher stored in buffer; otherCatalogNumbers: 0174126563; associatedSequences: OQ746672 (16S); occurrenceID: 41B2F126-ECDC-55F1-8775-6447FB277222; **Taxon:** taxonConceptID: Maldanidae sp. (NHM_026); scientificName: Maldanidae; kingdom: Animalia; phylum: Annelida; class: Polychaeta; family: Maldanidae; taxonRank: family; scientificNameAuthorship: Malmgren, 1867; **Location:** waterBody: Pacific; stateProvince: Clarion Clipperton Zone; locality: Ocean Mineral Singapore exploration claim Stratum A; verbatimLocality: OMS Stratum A; maximumDepthInMeters: 4302; locationRemarks: Deployment EB06; at Station S5; from R/V Thomas G. Thompson Cruise no. TN319; verbatimLatitude: 12'15.44; verbatimLongitude: 117'18.13; decimalLatitude: 12.25733; decimalLongitude: -117.30217; geodeticDatum: WGS84; **Identification:** identifiedBy: Helena Wiklund | Lenka Neal | Thomas Dahlgren | Adrian Glover | Madeleine Brasier | Regan Drennan | Eva Stewart; dateIdentified: 2021-04-20; identificationRemarks: identified by DNA and morphology; **Event:** eventID: OMS1_AB02_EB06; samplingProtocol: Brenke Epibenthic Sledge; eventDate: 2015-03-01; eventTime: 04:02; habitat: Abyssal plain; fieldNotes: Collected from epi net (on the epibenthic sledge); **Record Level:** language: en; institutionCode: NHMUK; collectionCode: ZOO; datasetName: ABYSSLINE; basisOfRecord: PreservedSpecimen
- **Type status:** Other material. **Occurrence:** catalogNumber: NHMUK ANEA 2023.413; recordNumber: NHM_0026; recordedBy: Adrian Glover | Helena Wiklund | Thomas Dahlgren | Magdalena Georgieva; individualCount: 1; preparations: specimen stored in 80% non-denatured ethanol aqueous solution | DNA voucher stored in buffer; otherCatalogNumbers: 0174127347; associatedSequences: OQ746468 (16S) | OQ746791 (18S) | OQ738495 (COI); occurrenceID: C6676585-38E9-51D3-9DDE-EA630D350CA0; **Taxon:** taxonConceptID: Maldanidae sp. (NHM_026); scientificName: Maldanidae; kingdom: Animalia; phylum: Annelida; class: Polychaeta; family: Maldanidae; taxonRank: family; scientificNameAuthorship: Malmgren, 1867; **Location:** waterBody: Pacific; stateProvince: Clarion Clipperton Zone; locality: UK Seabed Resources Ltd exploration area UK-1 Stratum A; verbatimLocality: UK1 Stratum A; maximumDepthInMeters: 4336; locationRemarks: Deployment EB01; at Station B-K-E; from R/V Melville Cruise no. MV1313; verbatimLatitude: 13°50.232; verbatimLongitude: 116°33.506; decimalLatitude: 13.8372; decimalLongitude: -116.55843; geodeticDatum: WGS84; **Identification:** identifiedBy: Helena Wiklund | Lenka Neal | Thomas Dahlgren | Adrian Glover | Madeleine Brasier | Regan Drennan | Eva Stewart; dateIdentified: 2021-04-20; identificationRemarks: identified by DNA and morphology; **Event:** eventID: UK1_AB01_EB01; samplingProtocol: Brenke Epibenthic Sledge; eventDate: 2013-10-09; eventTime: 10:26; habitat: Abyssal plain; fieldNotes: Collected from epi net (on the epibenthic sledge); **Record Level:** language: en; institutionCode: NHMUK; collectionCode: ZOO; datasetName: ABYSSLINE; basisOfRecord: PreservedSpecimen

##### Distribution

Eastern Clarion-Clipperton Zone, central Pacific Ocean.

##### Diagnosis

**Figure 52.**
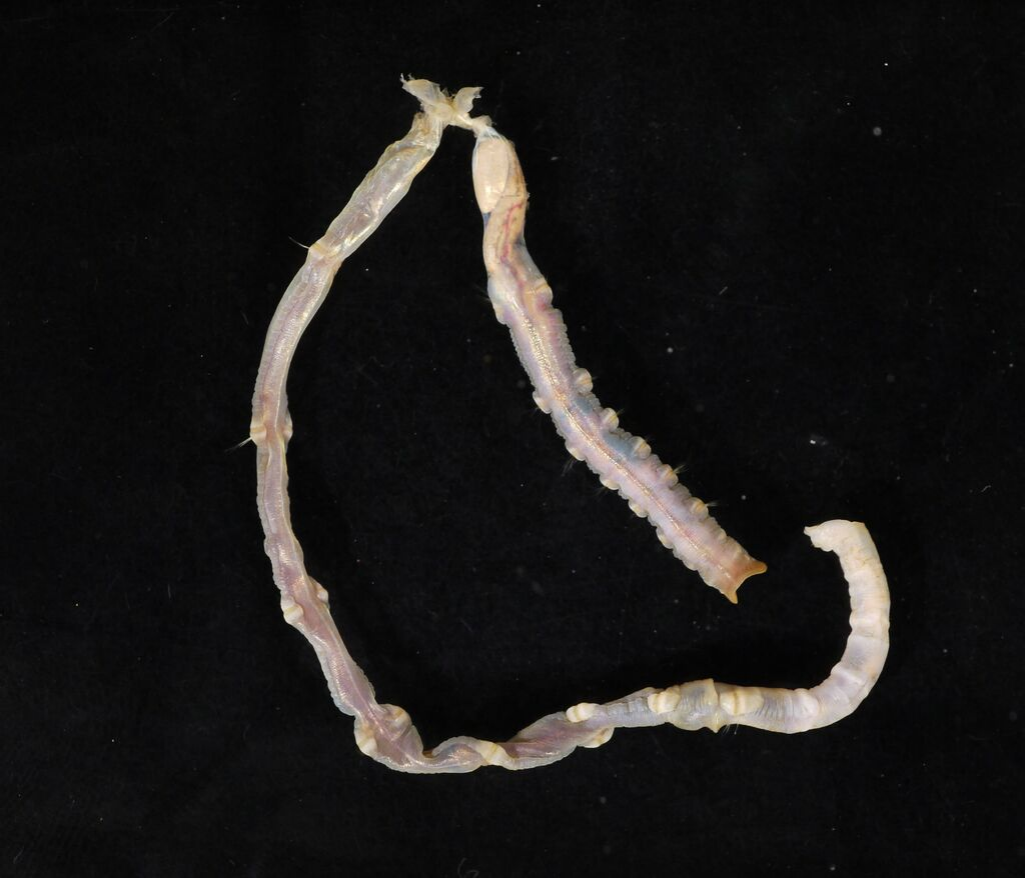
Maldanidae sp. (NHM_026), complete live specimen NHM_026 in lateral view.

Specimens (Fig. 52) consistent with placement within family Maldanidae, based on morphology and DNA.

#### Maldanidae sp. (NHM_836)

DFB7498E-0395-5112-B8EB-CDE2053D891C

##### Materials

- **Type status:** Other material. **Occurrence:** catalogNumber: NHMUK ANEA 2023.422; recordNumber: NHM_1947J; recordedBy: Adrian Glover | Helena Wiklund | Thomas Dahlgren | Madeleine Brasier; individualCount: 1; preparations: specimen stored in 80% non-denatured ethanol aqueous solution | DNA voucher stored in buffer; otherCatalogNumbers: 0174126162; associatedSequences: OQ746739 (16S); occurrenceID: B7A179AE-572B-57F0-B3DB-AFEB5ED341AC; **Taxon:** taxonConceptID: Maldanidae sp. (NHM_836); scientificName: Maldanidae; kingdom: Animalia; phylum: Annelida; class: Polychaeta; family: Maldanidae; taxonRank: family; scientificNameAuthorship: Malmgren, 1867; **Location:** waterBody: Pacific; stateProvince: Clarion Clipperton Zone; locality: Ocean Mineral Singapore exploration claim Stratum A; verbatimLocality: OMS Stratum A; maximumDepthInMeters: 4094; locationRemarks: Deployment EB11; at Station S10; from R/V Thomas G. Thompson Cruise no. TN319; verbatimLatitude: 12°02.49’; verbatimLongitude: 117°13.03’; decimalLatitude: 12.0415; decimalLongitude: -117.21717; geodeticDatum: WGS84; **Identification:** identifiedBy: Helena Wiklund | Lenka Neal | Thomas Dahlgren | Adrian Glover | Madeleine Brasier | Regan Drennan | Eva Stewart; dateIdentified: 2021-04-20; identificationRemarks: identified by DNA and morphology; **Event:** eventID: OMS1_AB02_EB11; samplingProtocol: Brenke Epibenthic Sledge; eventDate: 2015-03-13; habitat: Abyssal plain; fieldNotes: Collected from epi net (on the epibenthic sledge); **Record Level:** language: en; institutionCode: NHMUK; collectionCode: ZOO; datasetName: ABYSSLINE; basisOfRecord: PreservedSpecimen
- **Type status:** Other material. **Occurrence:** catalogNumber: NHMUK ANEA 2023.421; recordNumber: NHM_1770; recordedBy: Adrian Glover | Helena Wiklund | Thomas Dahlgren | Madeleine Brasier; individualCount: 1; preparations: specimen stored in 80% non-denatured ethanol aqueous solution | DNA voucher stored in buffer; otherCatalogNumbers: 0174126219; associatedSequences: OQ746715 (16S) | OQ738599 (COI); occurrenceID: 00385ACB-1719-5465-B61C-A67C1C98895D; **Taxon:** taxonConceptID: Maldanidae sp. (NHM_836); scientificName: Maldanidae; kingdom: Animalia; phylum: Annelida; class: Polychaeta; family: Maldanidae; taxonRank: family; scientificNameAuthorship: Malmgren, 1867; **Location:** waterBody: Pacific; stateProvince: Clarion Clipperton Zone; locality: Ocean Mineral Singapore exploration claim Stratum A; verbatimLocality: OMS Stratum A; maximumDepthInMeters: 4045; locationRemarks: Deployment EB10; at Station S7; from R/V Thomas G. Thompson Cruise no. TN319; verbatimLatitude: 12'10.43; verbatimLongitude: 117'11.57; decimalLatitude: 12.17383; decimalLongitude: -117.19283; geodeticDatum: WGS84; **Identification:** identifiedBy: Helena Wiklund | Lenka Neal | Thomas Dahlgren | Adrian Glover | Madeleine Brasier | Regan Drennan | Eva Stewart; dateIdentified: 2021-04-20; identificationRemarks: identified by DNA and morphology; **Event:** eventID: OMS1_AB02_EB10; samplingProtocol: Brenke Epibenthic Sledge; eventDate: 2015-03-11; eventTime: 22:49; habitat: Abyssal plain; fieldNotes: Collected from epi net (on the epibenthic sledge); **Record Level:** language: en; institutionCode: NHMUK; collectionCode: ZOO; datasetName: ABYSSLINE; basisOfRecord: PreservedSpecimen
- **Type status:** Other material. **Occurrence:** recordNumber: NHM_1163A; recordedBy: Adrian Glover | Helena Wiklund | Thomas Dahlgren | Madeleine Brasier; individualCount: 1; preparations: DNA voucher stored in buffer; otherCatalogNumbers: 0174126535; associatedSequences: OQ746630 (16S) | OQ738563 (COI); occurrenceID: DA7EC0BA-AE42-591A-91B5-8938AF9CED9E; **Taxon:** taxonConceptID: Maldanidae sp. (NHM_836); scientificName: Maldanidae; kingdom: Animalia; phylum: Annelida; class: Polychaeta; family: Maldanidae; taxonRank: family; scientificNameAuthorship: Malmgren, 1867; **Location:** waterBody: Pacific; stateProvince: Clarion Clipperton Zone; locality: Ocean Mineral Singapore exploration claim Stratum A; verbatimLocality: OMS Stratum A; maximumDepthInMeters: 4100; locationRemarks: Deployment EB05; at Station S2; from R/V Thomas G. Thompson Cruise no. TN319; verbatimLatitude: 12'06.93; verbatimLongitude: 117'09.87; decimalLatitude: 12.1155; decimalLongitude: -117.1645; geodeticDatum: WGS84; **Identification:** identifiedBy: Helena Wiklund | Lenka Neal | Thomas Dahlgren | Adrian Glover | Madeleine Brasier | Regan Drennan | Eva Stewart; dateIdentified: 2021-04-20; identificationRemarks: identified by DNA and morphology; **Event:** eventID: OMS1_AB02_EB05; samplingProtocol: Brenke Epibenthic Sledge; eventDate: 2015-02-26; eventTime: 21:29; habitat: Abyssal plain; fieldNotes: Collected from epi net (on the epibenthic sledge); **Record Level:** language: en; institutionCode: NHMUK; collectionCode: ZOO; datasetName: ABYSSLINE; basisOfRecord: PreservedSpecimen
- **Type status:** Other material. **Occurrence:** catalogNumber: NHMUK ANEA 2023.419; recordNumber: NHM_0836; recordedBy: Adrian Glover | Helena Wiklund | Thomas Dahlgren | Madeleine Brasier; individualCount: 1; preparations: specimen stored in 80% non-denatured ethanol aqueous solution | DNA voucher stored in buffer; otherCatalogNumbers: 0174126790; associatedSequences: OQ746565 (16S) | OQ746862 (18S) | OQ738538 (COI); occurrenceID: E7B350FA-94C1-5AA9-B1A9-D7F6C5209C13; **Taxon:** taxonConceptID: Maldanidae sp. (NHM_836); scientificName: Maldanidae; kingdom: Animalia; phylum: Annelida; class: Polychaeta; family: Maldanidae; taxonRank: family; scientificNameAuthorship: Malmgren, 1867; **Location:** waterBody: Pacific; stateProvince: Clarion Clipperton Zone; locality: UK Seabed Resources Ltd exploration area UK-1 Stratum B; verbatimLocality: UK1 Stratum B; maximumDepthInMeters: 4160; locationRemarks: Deployment BC04; at Station U5; from R/V Thomas G. Thompson Cruise no. TN319; verbatimLatitude: 12'22.259; verbatimLongitude: 116'36.819; decimalLatitude: 12.37098; decimalLongitude: -116.61365; geodeticDatum: WGS84; **Identification:** identifiedBy: Helena Wiklund | Lenka Neal | Thomas Dahlgren | Adrian Glover | Madeleine Brasier | Regan Drennan | Eva Stewart; dateIdentified: 2021-04-20; identificationRemarks: identified by DNA and morphology; **Event:** eventID: UK1_AB02_BC04; samplingProtocol: USNEL Box Core; eventDate: 2015-02-21; eventTime: 03:38; habitat: Abyssal plain; fieldNotes: Collected from 0-2 cm layer of box core using a 300 micron sieve; **Record Level:** language: en; institutionCode: NHMUK; collectionCode: ZOO; datasetName: ABYSSLINE; basisOfRecord: PreservedSpecimen
- **Type status:** Other material. **Occurrence:** recordNumber: NHM_0682; recordedBy: Adrian Glover | Helena Wiklund | Thomas Dahlgren | Madeleine Brasier; individualCount: 1; preparations: Tissue voucher stored in 80% non-denatured ethanol aqueous solution | DNA voucher stored in buffer; otherCatalogNumbers: 0109405400 | 0174126553; associatedSequences: OQ746539 (16S) | OQ738531 (COI); occurrenceID: CF8157F0-BCB8-520A-9678-23E9240EC80A; **Taxon:** taxonConceptID: Maldanidae sp. (NHM_836); scientificName: Maldanidae; kingdom: Animalia; phylum: Annelida; class: Polychaeta; family: Maldanidae; taxonRank: family; scientificNameAuthorship: Malmgren, 1867; **Location:** waterBody: Pacific; stateProvince: Clarion Clipperton Zone; locality: UK Seabed Resources Ltd exploration area UK-1 Stratum B; verbatimLocality: UK1 Stratum B; maximumDepthInMeters: 4425; locationRemarks: Deployment EB02; at Station U5; from R/V Thomas G. Thompson Cruise no. TN319; verbatimLatitude: 12'32.23; verbatimLongitude: 116'36.25; decimalLatitude: 12.53717; decimalLongitude: -116.60417; geodeticDatum: WGS84; **Identification:** identifiedBy: Helena Wiklund | Lenka Neal | Thomas Dahlgren | Adrian Glover | Madeleine Brasier | Regan Drennan | Eva Stewart; dateIdentified: 2021-04-20; identificationRemarks: identified by DNA and morphology; **Event:** eventID: UK1_AB02_EB02; samplingProtocol: Brenke Epibenthic Sledge; eventDate: 2015-02-20; eventTime: 06:24; habitat: Abyssal plain; fieldNotes: Collected from epi net (on the epibenthic sledge); **Record Level:** language: en; institutionCode: NHMUK; collectionCode: ZOO; datasetName: ABYSSLINE; basisOfRecord: PreservedSpecimen

##### Distribution

Eastern Clarion-Clipperton Zone, central Pacific Ocean.

##### Diagnosis

**Figure 53.**
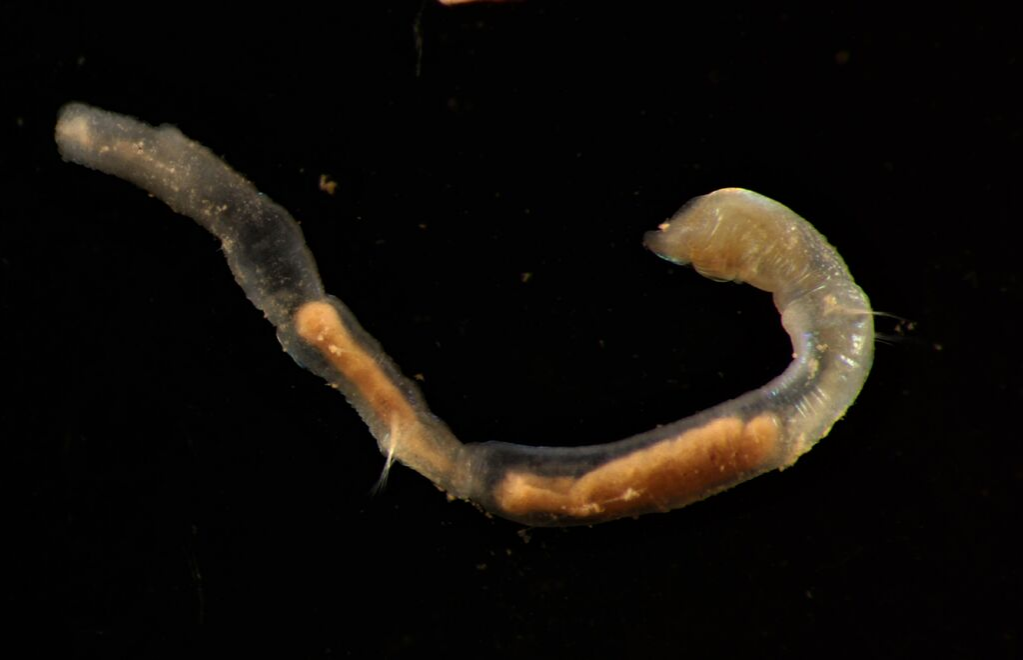
Maldanidae sp. (NHM_836), anterior fragment of live specimen NHM_682 in lateral view.

Damaged specimens (Fig. 53) consistent with placement within family Maldanidae, based on morphology and DNA.

#### Maldanidae sp. (NHM_900)

CAB5F6D3-C1B9-5062-9604-44612B515C3D

##### Materials

- **Type status:** Other material. **Occurrence:** recordNumber: NHM_0900; recordedBy: Adrian Glover | Helena Wiklund | Thomas Dahlgren | Madeleine Brasier; individualCount: 1; preparations: Tissue voucher stored in 80% non-denatured ethanol aqueous solution | DNA voucher stored in buffer; otherCatalogNumbers: 0109405352 | 0174126769; associatedSequences: OQ746572 (16S); occurrenceID: 00409B34-D306-5E80-B0D9-8CB2877BB22F; **Taxon:** taxonConceptID: Maldanidae sp. (NHM_900); scientificName: Maldanidae; kingdom: Animalia; phylum: Annelida; class: Polychaeta; family: Maldanidae; taxonRank: family; scientificNameAuthorship: Malmgren, 1867; **Location:** waterBody: Pacific; stateProvince: Clarion Clipperton Zone; locality: UK Seabed Resources Ltd exploration area UK-1 Stratum B; verbatimLocality: UK1 Stratum B; maximumDepthInMeters: 4198; locationRemarks: Deployment EB03; at Station U4; from R/V Thomas G. Thompson Cruise no. TN319; verbatimLatitude: 12'34.28; verbatimLongitude: 116'36.63; decimalLatitude: 12.57133; decimalLongitude: -116.6105; geodeticDatum: WGS84; **Identification:** identifiedBy: Helena Wiklund | Lenka Neal | Thomas Dahlgren | Adrian Glover | Madeleine Brasier | Regan Drennan | Eva Stewart; dateIdentified: 2021-04-20; identificationRemarks: identified by DNA and morphology; **Event:** eventID: UK1_AB02_EB03; samplingProtocol: Brenke Epibenthic Sledge; eventDate: 2015-02-23; eventTime: 05:39; habitat: Abyssal plain; fieldNotes: Collected from epi net (on the epibenthic sledge); **Record Level:** language: en; institutionCode: NHMUK; collectionCode: ZOO; datasetName: ABYSSLINE; basisOfRecord: PreservedSpecimen

##### Distribution

Eastern Clarion-Clipperton Zone, central Pacific Ocean.

##### Diagnosis

**Figure 54.**
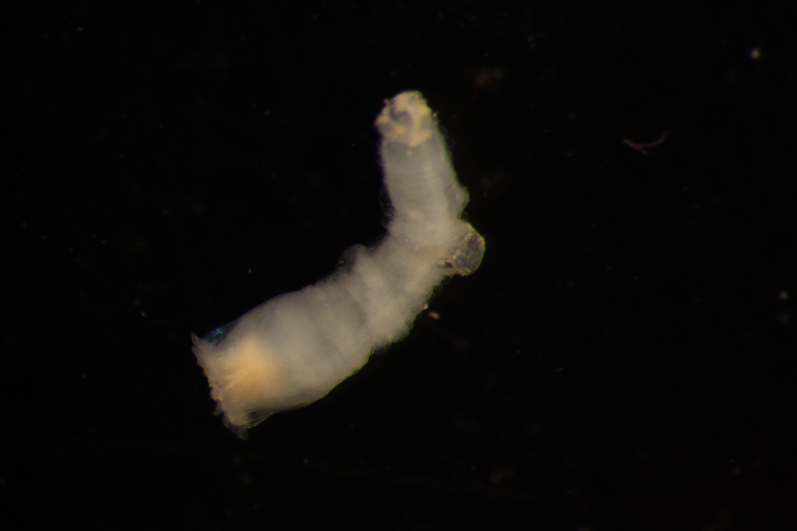
Maldanidae sp. (NHM_900), posterior fragment of specimen NHM_900.

Damaged specimen (Fig. 54) consistent with placement within family Maldanidae, based on morphology and DNA.

#### Maldanidae sp. (NHM_1170)

3C9D8C17-25F8-5DD5-927A-8ABD507310B9

##### Materials

- **Type status:** Other material. **Occurrence:** catalogNumber: NHMUK ANEA 2023.420; recordNumber: NHM_1170; recordedBy: Adrian Glover | Helena Wiklund | Thomas Dahlgren | Madeleine Brasier; individualCount: 1; preparations: specimen stored in 80% non-denatured ethanol aqueous solution | DNA voucher stored in buffer; otherCatalogNumbers: 0174126766; associatedSequences: OQ746636 (16S) | OQ746879 (18S) | OQ738566 (COI); occurrenceID: 0ED743C7-B5FD-5691-8CBF-552062DAB313; **Taxon:** taxonConceptID: Maldanidae sp. (NHM_1170); scientificName: Maldanidae; kingdom: Animalia; phylum: Annelida; class: Polychaeta; family: Maldanidae; taxonRank: family; scientificNameAuthorship: Malmgren, 1867; **Location:** waterBody: Pacific; stateProvince: Clarion Clipperton Zone; locality: Ocean Mineral Singapore exploration claim Stratum A; verbatimLocality: OMS Stratum A; maximumDepthInMeters: 4100; locationRemarks: Deployment EB05; at Station S2; from R/V Thomas G. Thompson Cruise no. TN319; verbatimLatitude: 12'06.93; verbatimLongitude: 117'09.87; decimalLatitude: 12.1155; decimalLongitude: -117.1645; geodeticDatum: WGS84; **Identification:** identifiedBy: Helena Wiklund | Lenka Neal | Thomas Dahlgren | Adrian Glover | Madeleine Brasier | Regan Drennan | Eva Stewart; dateIdentified: 2021-04-20; identificationRemarks: identified by DNA and morphology; **Event:** eventID: OMS1_AB02_EB05; samplingProtocol: Brenke Epibenthic Sledge; eventDate: 2015-02-26; eventTime: 21:29; habitat: Abyssal plain; fieldNotes: Collected from epi net (on the epibenthic sledge); **Record Level:** language: en; institutionCode: NHMUK; collectionCode: ZOO; datasetName: ABYSSLINE; basisOfRecord: PreservedSpecimen

##### Distribution

Eastern Clarion-Clipperton Zone, central Pacific Ocean.

##### Diagnosis

**Figure 55.**
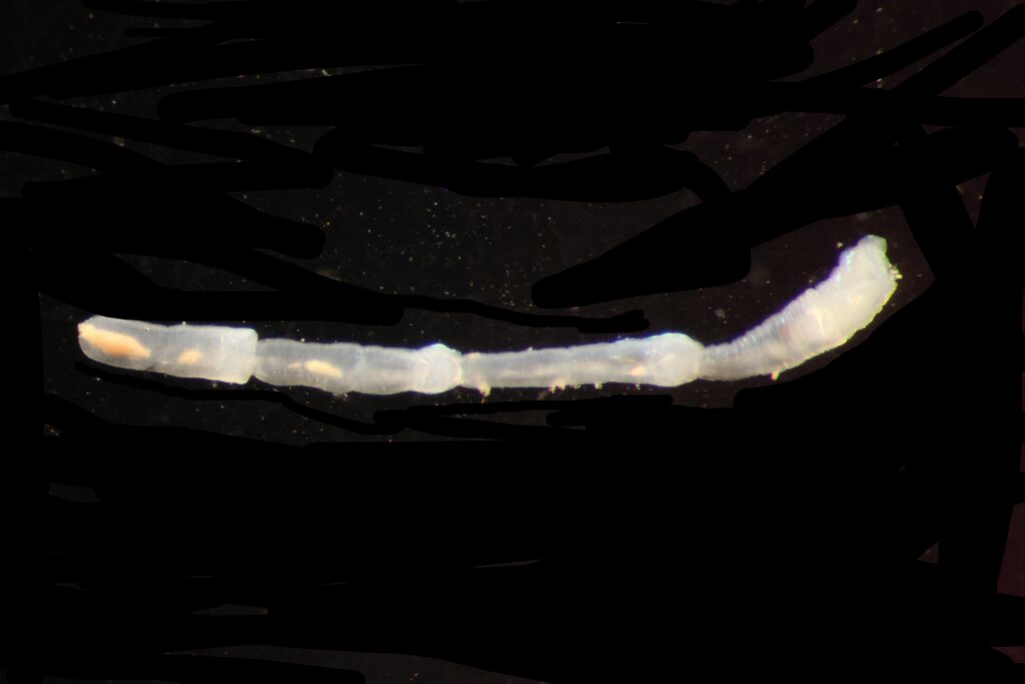
Maldanidae sp. (NHM_1170), anterior end in live specimen NHM_1170 in lateral view.

Damaged specimen (Fig. 55) consistent with placement within family Maldanidae, based on morphology and DNA.

#### Maldanidae sp. (NHM_1178)

24951446-7DDF-5877-8AD6-5AE81FFEEEFF

##### Materials

- **Type status:** Other material. **Occurrence:** catalogNumber: NHMUK ANEA 2023.418; recordNumber: NHM_1890; recordedBy: Adrian Glover | Helena Wiklund | Thomas Dahlgren | Madeleine Brasier; individualCount: 1; preparations: specimen stored in 80% non-denatured ethanol aqueous solution | DNA voucher stored in buffer; otherCatalogNumbers: 0174126770; associatedSequences: OQ746729 (16S) | OQ738602 (COI); occurrenceID: F919E9E3-6E48-541F-AFEE-C999BD3CE16C; **Taxon:** taxonConceptID: Maldanidae sp. (NHM_1178); scientificName: Maldanidae; kingdom: Animalia; phylum: Annelida; class: Polychaeta; family: Maldanidae; taxonRank: family; scientificNameAuthorship: Malmgren, 1867; **Location:** waterBody: Pacific; stateProvince: Clarion Clipperton Zone; locality: Ocean Mineral Singapore exploration claim Stratum A; verbatimLocality: OMS Stratum A; maximumDepthInMeters: 4094; locationRemarks: Deployment EB11; at Station S10; from R/V Thomas G. Thompson Cruise no. TN319; verbatimLatitude: 12°02.49’; verbatimLongitude: 117°13.03’; decimalLatitude: 12.0415; decimalLongitude: -117.21717; geodeticDatum: WGS84; **Identification:** identifiedBy: Helena Wiklund | Lenka Neal | Thomas Dahlgren | Adrian Glover | Madeleine Brasier | Regan Drennan | Eva Stewart; dateIdentified: 2021-04-20; identificationRemarks: identified by DNA and morphology; **Event:** eventID: OMS1_AB02_EB11; samplingProtocol: Brenke Epibenthic Sledge; eventDate: 2015-03-13; habitat: Abyssal plain; fieldNotes: Collected from nodule (on the epibenthic sledge); **Record Level:** language: en; institutionCode: NHMUK; collectionCode: ZOO; datasetName: ABYSSLINE; basisOfRecord: PreservedSpecimen
- **Type status:** Other material. **Occurrence:** recordNumber: NHM_1178; recordedBy: Adrian Glover | Helena Wiklund | Thomas Dahlgren | Madeleine Brasier; individualCount: 1; preparations: Tissue voucher stored in 80% non-denatured ethanol aqueous solution | DNA voucher stored in buffer; otherCatalogNumbers: 0109405429 | 0174126745; associatedSequences: OQ746640 (16S) | OQ746880 (18S) | OQ738570 (COI); occurrenceID: 0EC57A8E-1261-542D-9589-AE18638276A2; **Taxon:** taxonConceptID: Maldanidae sp. (NHM_1178); scientificName: Maldanidae; kingdom: Animalia; phylum: Annelida; class: Polychaeta; family: Maldanidae; taxonRank: family; scientificNameAuthorship: Malmgren, 1867; **Location:** waterBody: Pacific; stateProvince: Clarion Clipperton Zone; locality: Ocean Mineral Singapore exploration claim Stratum A; verbatimLocality: OMS Stratum A; maximumDepthInMeters: 4100; locationRemarks: Deployment EB05; at Station S2; from R/V Thomas G. Thompson Cruise no. TN319; verbatimLatitude: 12'06.93; verbatimLongitude: 117'09.87; decimalLatitude: 12.1155; decimalLongitude: -117.1645; geodeticDatum: WGS84; **Identification:** identifiedBy: Helena Wiklund | Lenka Neal | Thomas Dahlgren | Adrian Glover | Madeleine Brasier | Regan Drennan | Eva Stewart; dateIdentified: 2021-04-20; identificationRemarks: identified by DNA and morphology; **Event:** eventID: OMS1_AB02_EB05; samplingProtocol: Brenke Epibenthic Sledge; eventDate: 2015-02-26; eventTime: 21:29; habitat: Abyssal plain; fieldNotes: Collected from epi net (on the epibenthic sledge); **Record Level:** language: en; institutionCode: NHMUK; collectionCode: ZOO; datasetName: ABYSSLINE; basisOfRecord: PreservedSpecimen

##### Distribution

Eastern Clarion-Clipperton Zone, central Pacific Ocean.

##### Diagnosis

**Figure 56.**
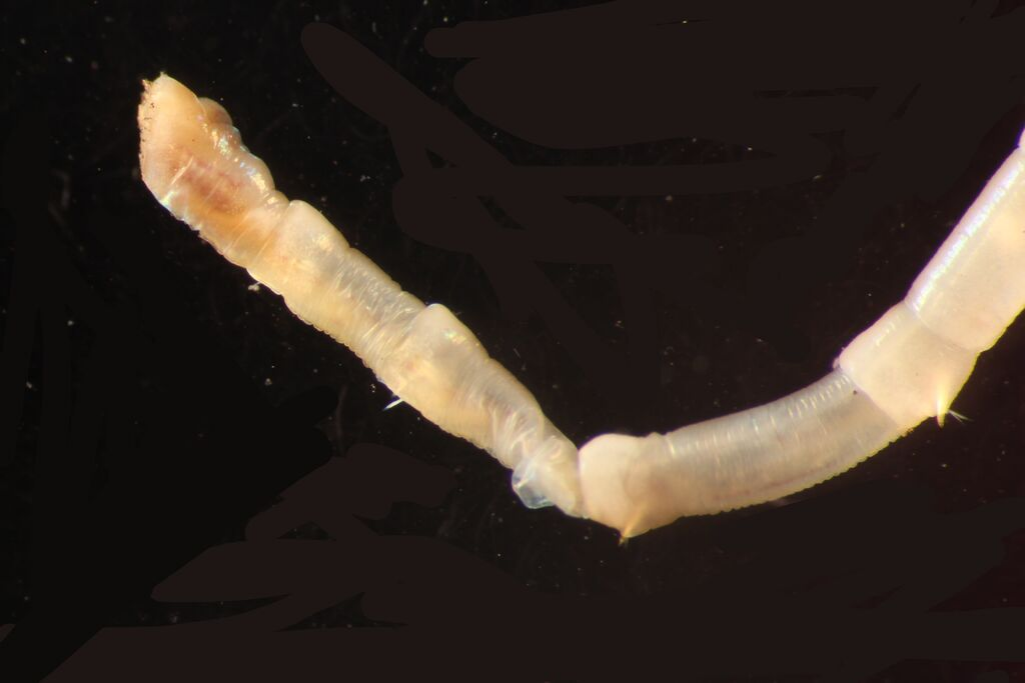
Maldanidae sp. (NHM_1178), anterior fragment of specimen NHM_1890 in lateral view.

Damaged specimens (Fig. 56) consistent with placement within family Maldanidae, based on morphology and DNA.

### Onuphidae Kinberg, 1865

#### Onuphidae sp. (NHM_1010)

34653B27-00DE-5B7E-9BFD-3BCAC90B822C

##### Materials

- **Type status:** Other material. **Occurrence:** catalogNumber: NHMUK ANEA 2023.423; recordNumber: NHM_1010; recordedBy: Adrian Glover | Helena Wiklund | Thomas Dahlgren | Madeleine Brasier; individualCount: 1; preparations: specimen stored in 80% non-denatured ethanol aqueous solution | DNA voucher stored in buffer; otherCatalogNumbers: 0174126311; associatedSequences: OQ746605 (16S) | OQ746872 (18S) | OQ738555 (COI); occurrenceID: A5737552-DFB9-5DF4-B445-FB6CF77AB07C; **Taxon:** taxonConceptID: Onuphidae sp. (NHM_1010); scientificName: Onuphidae; kingdom: Animalia; phylum: Annelida; class: Polychaeta; order: Eunicida; family: Onuphidae; taxonRank: family; scientificNameAuthorship: Kinberg, 1865; **Location:** waterBody: Pacific; stateProvince: Clarion Clipperton Zone; locality: Ocean Mineral Singapore exploration claim Stratum A; verbatimLocality: OMS Stratum A; maximumDepthInMeters: 4122; locationRemarks: Deployment EB04; at Station S1; from R/V Thomas G. Thompson Cruise no. TN319; verbatimLatitude: 12'08.02; verbatimLongitude: 117'17.52; decimalLatitude: 12.13367; decimalLongitude: -117.292; geodeticDatum: WGS84; **Identification:** identifiedBy: Helena Wiklund | Lenka Neal | Thomas Dahlgren | Adrian Glover | Madeleine Brasier | Regan Drennan | Eva Stewart; dateIdentified: 2021-04-20; identificationRemarks: identified by DNA and morphology; **Event:** eventID: OMS1_AB02_EB04; samplingProtocol: Brenke Epibenthic Sledge; eventDate: 2015-02-24; eventTime: 19:10; habitat: Abyssal plain; fieldNotes: Collected from epi net (on the epibenthic sledge); **Record Level:** language: en; institutionCode: NHMUK; collectionCode: ZOO; datasetName: ABYSSLINE; basisOfRecord: PreservedSpecimen
- **Type status:** Other material. **Occurrence:** catalogNumber: NHMUK ANEA 2023.424; recordNumber: NHM_2629; recordedBy: Adrian Glover | Helena Wiklund | Thomas Dahlgren | Madeleine Brasier; individualCount: 1; preparations: specimen stored in 80% non-denatured ethanol aqueous solution | DNA voucher stored in buffer; otherCatalogNumbers: 0174126230; associatedSequences: OQ746781 (16S) | OQ738617 (COI); occurrenceID: 0C9C0FC1-5335-5889-9C92-39C22528822B; **Taxon:** taxonConceptID: Onuphidae sp. (NHM_1010); scientificName: Onuphidae; kingdom: Animalia; phylum: Annelida; class: Polychaeta; order: Eunicida; family: Onuphidae; taxonRank: family; scientificNameAuthorship: Kinberg, 1865; **Location:** waterBody: Pacific; stateProvince: Clarion Clipperton Zone; locality: Ocean Mineral Singapore exploration claim Stratum A; verbatimLocality: OMS Stratum A; maximumDepthInMeters: 4122; locationRemarks: Deployment EB04; at Station S1; from R/V Thomas G. Thompson Cruise no. TN319; verbatimLatitude: 12'08.02; verbatimLongitude: 117'17.52; decimalLatitude: 12.13367; decimalLongitude: -117.292; geodeticDatum: WGS84; **Identification:** identifiedBy: Helena Wiklund | Lenka Neal | Thomas Dahlgren | Adrian Glover | Madeleine Brasier | Regan Drennan | Eva Stewart; dateIdentified: 2021-04-20; identificationRemarks: identified by DNA and morphology; **Event:** eventID: OMS1_AB02_EB04; samplingProtocol: Brenke Epibenthic Sledge; eventDate: 2015-02-24; eventTime: 19:10; habitat: Abyssal plain; fieldNotes: Collected from supra net (on the epibenthic sledge); **Record Level:** language: en; institutionCode: NHMUK; collectionCode: ZOO; datasetName: ABYSSLINE; basisOfRecord: PreservedSpecimen

##### Distribution

Eastern Clarion-Clipperton Zone, central Pacific Ocean.

##### Diagnosis

**Figure 57.**
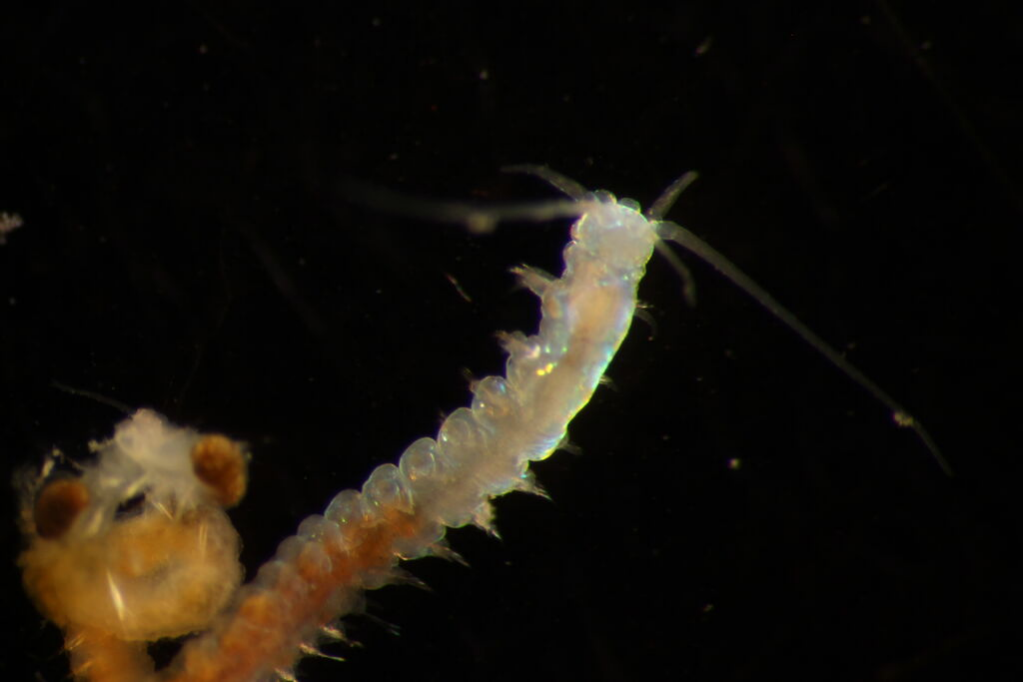
Onuphidae sp. (NHM_1010), anterior fragment of live specimen NHM_1010 in dorsal view.

Damaged specimens (Fig. 57) consistent with placement within family Onuphidae, based on morphology and DNA.

#### Onuphidae sp. (NHM_2430)

F72EF9FD-2F0D-5EB9-88E2-8194EC51B6E7

##### Materials

- **Type status:** Other material. **Occurrence:** catalogNumber: NHMUK ANEA 2023.425; recordNumber: NHM_2430; recordedBy: Adrian Glover | Helena Wiklund | Thomas Dahlgren | Madeleine Brasier; individualCount: 1; preparations: specimen stored in 80% non-denatured ethanol aqueous solution | DNA voucher stored in buffer; otherCatalogNumbers: 0174126231; associatedSequences: OQ746774 (16S) | OQ738613 (COI); occurrenceID: FDF0BF13-0B82-5B23-8DB9-9191367A1AE7; **Taxon:** taxonConceptID: Onuphidae sp. (NHM_2430); scientificName: Onuphidae; kingdom: Animalia; phylum: Annelida; class: Polychaeta; order: Eunicida; family: Onuphidae; taxonRank: family; scientificNameAuthorship: Kinberg, 1865; **Location:** waterBody: Pacific; stateProvince: Clarion Clipperton Zone; locality: UK Seabed Resources Ltd exploration area UK-1 Stratum B; verbatimLocality: UK1 Stratum B; maximumDepthInMeters: 4202; locationRemarks: Deployment EB01; at Station U2; from R/V Thomas G. Thompson Cruise no. TN319; verbatimLatitude: 12'23.17456; verbatimLongitude: 116'32.92021; decimalLatitude: 12.38624; decimalLongitude: -116.54867; geodeticDatum: WGS84; **Identification:** identifiedBy: Helena Wiklund | Lenka Neal | Thomas Dahlgren | Adrian Glover | Madeleine Brasier | Regan Drennan | Eva Stewart; dateIdentified: 2021-04-20; identificationRemarks: identified by DNA and morphology; **Event:** eventID: UK1_AB02_EB01; samplingProtocol: Brenke Epibenthic Sledge; eventDate: 2015-02-17; eventTime: 05:15; habitat: Abyssal plain; fieldNotes: Collected from supra net (on the epibenthic sledge); **Record Level:** language: en; institutionCode: NHMUK; collectionCode: ZOO; datasetName: ABYSSLINE; basisOfRecord: PreservedSpecimen

##### Distribution

Eastern Clarion-Clipperton Zone, central Pacific Ocean.

##### Diagnosis

Damaged specimen (Fig. 58) consistent with placement within family Onuphidae, based on morphology and DNA.

### Orbiniidae Hartman, 1942

#### Orbiniidae sp. (NHM_102)

85D33A7A-F0CB-50A9-9115-DACC925953D3

##### Materials

- **Type status:** Other material. **Occurrence:** recordNumber: NHM_0102; recordedBy: Adrian Glover | Helena Wiklund | Thomas Dahlgren | Magdalena Georgieva; individualCount: 1; preparations: DNA voucher stored in buffer; otherCatalogNumbers: 0174127362; associatedSequences: OQ746475 (16S); occurrenceID: 3EB890E9-AE32-5794-A0B7-D2DEBC81E463; **Taxon:** taxonConceptID: Orbiniidae sp. (NHM_102); scientificName: Orbiniidae; kingdom: Animalia; phylum: Annelida; class: Polychaeta; family: Orbiniidae; taxonRank: family; scientificNameAuthorship: Hartman, 1942; **Location:** waterBody: Pacific; stateProvince: Clarion Clipperton Zone; locality: UK Seabed Resources Ltd exploration area UK-1 Stratum A; verbatimLocality: UK1 Stratum A; maximumDepthInMeters: 4081; locationRemarks: Deployment BC05; at Station C; from R/V Melville Cruise no. MV1313; verbatimLatitude: 13°47.601; verbatimLongitude: 116°42.185; decimalLatitude: 13.79335; decimalLongitude: -116.70308; geodeticDatum: WGS84; **Identification:** identifiedBy: Helena Wiklund | Lenka Neal | Thomas Dahlgren | Adrian Glover | Madeleine Brasier | Regan Drennan | Eva Stewart; dateIdentified: 2021-04-20; identificationRemarks: identified by DNA and morphology; **Event:** eventID: UK1_AB01_BC05; samplingProtocol: USNEL Box Core; eventDate: 2013-10-11; eventTime: 12:30; habitat: Abyssal plain; fieldNotes: Collected from 0-2 cm layer of box core using a 300 micron sieve; **Record Level:** language: en; institutionCode: NHMUK; collectionCode: ZOO; datasetName: ABYSSLINE; basisOfRecord: PreservedSpecimen
- **Type status:** Other material. **Occurrence:** catalogNumber: NHMUK ANEA 2023.429; recordNumber: NHM_0110; recordedBy: Adrian Glover | Helena Wiklund | Thomas Dahlgren | Magdalena Georgieva; individualCount: 1; preparations: specimen stored in 80% non-denatured ethanol aqueous solution | DNA voucher stored in buffer; otherCatalogNumbers: 0174127337; associatedSequences: OQ746476 (16S) | OQ746797 (18S) | OQ738500 (COI); occurrenceID: D8FF54C2-FA5F-58B5-92FC-C4E9657FF80B; **Taxon:** taxonConceptID: Orbiniidae sp. (NHM_102); scientificName: Orbiniidae; kingdom: Animalia; phylum: Annelida; class: Polychaeta; family: Orbiniidae; taxonRank: family; scientificNameAuthorship: Hartman, 1942; **Location:** waterBody: Pacific; stateProvince: Clarion Clipperton Zone; locality: UK Seabed Resources Ltd exploration area UK-1 Stratum A; verbatimLocality: UK1 Stratum A; maximumDepthInMeters: 4081; locationRemarks: Deployment BC05; at Station C; from R/V Melville Cruise no. MV1313; verbatimLatitude: 13°47.601; verbatimLongitude: 116°42.185; decimalLatitude: 13.79335; decimalLongitude: -116.70308; geodeticDatum: WGS84; **Identification:** identifiedBy: Helena Wiklund | Lenka Neal | Thomas Dahlgren | Adrian Glover | Madeleine Brasier | Regan Drennan | Eva Stewart; dateIdentified: 2021-04-20; identificationRemarks: identified by DNA and morphology; **Event:** eventID: UK1_AB01_BC05; samplingProtocol: USNEL Box Core; eventDate: 2013-10-11; eventTime: 12:30; habitat: Abyssal plain; fieldNotes: Collected from 0-2 cm layer of box core using a 300 micron sieve; **Record Level:** language: en; institutionCode: NHMUK; collectionCode: ZOO; datasetName: ABYSSLINE; basisOfRecord: PreservedSpecimen
- **Type status:** Other material. **Occurrence:** recordNumber: NHM_0111; recordedBy: Adrian Glover | Helena Wiklund | Thomas Dahlgren | Magdalena Georgieva; individualCount: 1; preparations: Tissue voucher stored in 80% non-denatured ethanol aqueous solution | DNA voucher stored in buffer; otherCatalogNumbers: 0109405361 | 0174127343; associatedSequences: OQ746477 (16S) | OQ746798 (18S) | OQ738501 (COI); occurrenceID: 4E709F63-2922-5600-8305-2F31B6A2F89C; **Taxon:** taxonConceptID: Orbiniidae sp. (NHM_102); scientificName: Orbiniidae; kingdom: Animalia; phylum: Annelida; class: Polychaeta; family: Orbiniidae; taxonRank: family; scientificNameAuthorship: Hartman, 1942; **Location:** waterBody: Pacific; stateProvince: Clarion Clipperton Zone; locality: UK Seabed Resources Ltd exploration area UK-1 Stratum A; verbatimLocality: UK1 Stratum A; maximumDepthInMeters: 4081; locationRemarks: Deployment BC05; at Station C; from R/V Melville Cruise no. MV1313; verbatimLatitude: 13°47.601; verbatimLongitude: 116°42.185; decimalLatitude: 13.79335; decimalLongitude: -116.70308; geodeticDatum: WGS84; **Identification:** identifiedBy: Helena Wiklund | Lenka Neal | Thomas Dahlgren | Adrian Glover | Madeleine Brasier | Regan Drennan | Eva Stewart; dateIdentified: 2021-04-20; identificationRemarks: identified by DNA and morphology; **Event:** eventID: UK1_AB01_BC05; samplingProtocol: USNEL Box Core; eventDate: 2013-10-11; eventTime: 12:30; habitat: Abyssal plain; fieldNotes: Collected from 0-2 cm layer of box core using a 300 micron sieve; **Record Level:** language: en; institutionCode: NHMUK; collectionCode: ZOO; datasetName: ABYSSLINE; basisOfRecord: PreservedSpecimen
- **Type status:** Other material. **Occurrence:** recordNumber: NHM_0343; recordedBy: Adrian Glover | Helena Wiklund | Thomas Dahlgren | Magdalena Georgieva; individualCount: 1; preparations: Tissue voucher stored in 80% non-denatured ethanol aqueous solution | DNA voucher stored in buffer; otherCatalogNumbers: 0109405408 | 0174127296; associatedSequences: OQ746501 (16S) | OQ746819 (18S) | OQ738515 (COI); occurrenceID: 88B99713-6816-5E70-B73F-3C6114739C00; **Taxon:** taxonConceptID: Orbiniidae sp. (NHM_102); scientificName: Orbiniidae; kingdom: Animalia; phylum: Annelida; class: Polychaeta; family: Orbiniidae; taxonRank: family; scientificNameAuthorship: Hartman, 1942; **Location:** waterBody: Pacific; stateProvince: Clarion Clipperton Zone; locality: UK Seabed Resources Ltd exploration area UK-1 Stratum A; verbatimLocality: UK1 Stratum A; maximumDepthInMeters: 4128; locationRemarks: Deployment EB04; at Station G-I; from R/V Melville Cruise no. MV1313; verbatimLatitude: 13°45.21N; verbatimLongitude: 116°29.12W; decimalLatitude: 13.75583; decimalLongitude: -116.48667; geodeticDatum: WGS84; **Identification:** identifiedBy: Helena Wiklund | Lenka Neal | Thomas Dahlgren | Adrian Glover | Madeleine Brasier | Regan Drennan | Eva Stewart; dateIdentified: 2021-04-20; identificationRemarks: identified by DNA and morphology; **Event:** eventID: UK1_AB01_EB04; samplingProtocol: Brenke Epibenthic Sledge; eventDate: 2013-10-17; eventTime: 01:50; habitat: Abyssal plain; fieldNotes: Collected from epi net (on the epibenthic sledge); **Record Level:** language: en; institutionCode: NHMUK; collectionCode: ZOO; datasetName: ABYSSLINE; basisOfRecord: PreservedSpecimen

##### Distribution

Eastern Clarion-Clipperton Zone, central Pacific Ocean.

##### Diagnosis

**Figure 59.**
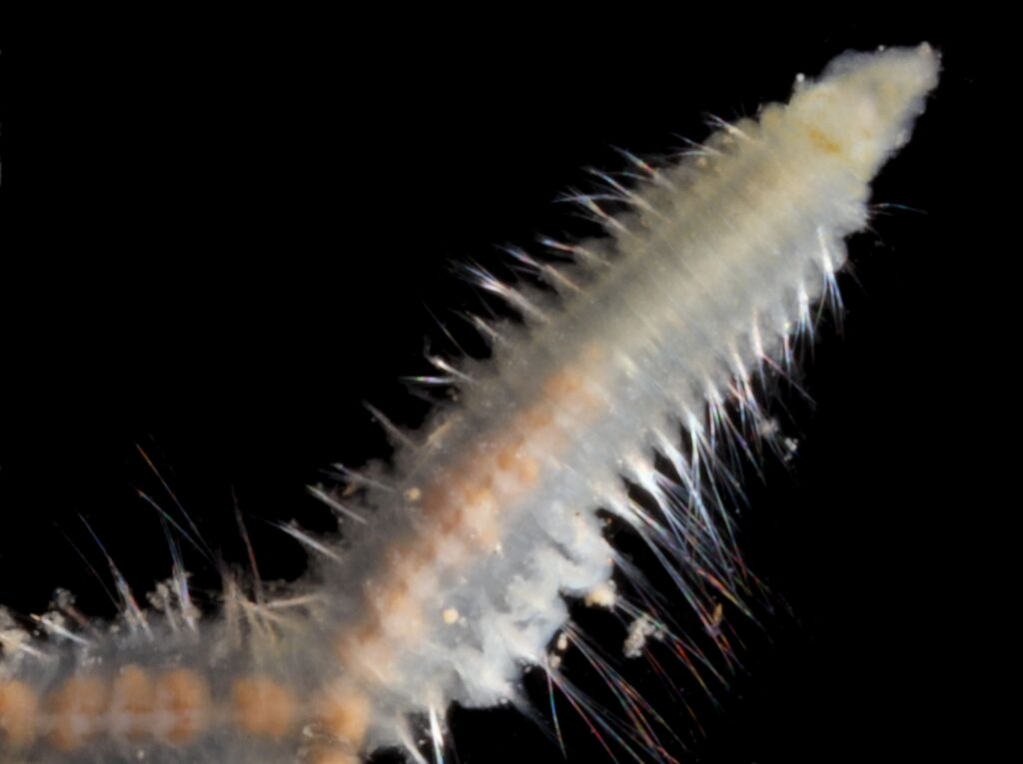
Orbiniidae sp. (NHM_102), anterior end of live specimen NHM_110.

Damaged specimens (Fig. 59) consistent with placement within family Orbiniidae, based on morphology and DNA.

#### Orbiniidae sp. (NHM_264)

F3D55652-0A8D-552D-BDFE-F9DA50923C58

##### Materials

- **Type status:** Other material. **Occurrence:** catalogNumber: NHMUK ANEA 2023.431; recordNumber: NHM_0264; recordedBy: Adrian Glover | Helena Wiklund | Thomas Dahlgren | Magdalena Georgieva; individualCount: 1; preparations: specimen stored in 80% non-denatured ethanol aqueous solution | DNA voucher stored in buffer; otherCatalogNumbers: 0174127384; associatedSequences: OQ746489 (16S); occurrenceID: 8F09DB5F-2E99-5767-ACB6-30CDEE90BD40; **Taxon:** taxonConceptID: Orbiniidae sp. (NHM_264); scientificName: Orbiniidae; kingdom: Animalia; phylum: Annelida; class: Polychaeta; family: Orbiniidae; taxonRank: family; scientificNameAuthorship: Hartman, 1942; **Location:** waterBody: Pacific; stateProvince: Clarion Clipperton Zone; locality: UK Seabed Resources Ltd exploration area UK-1 Stratum A; verbatimLocality: UK1 Stratum A; maximumDepthInMeters: 4128; locationRemarks: Deployment EB04; at Station G-I; from R/V Melville Cruise no. MV1313; verbatimLatitude: 13°45.21N; verbatimLongitude: 116°29.12W; decimalLatitude: 13.75583; decimalLongitude: -116.48667; geodeticDatum: WGS84; **Identification:** identifiedBy: Helena Wiklund | Lenka Neal | Thomas Dahlgren | Adrian Glover | Madeleine Brasier | Regan Drennan | Eva Stewart; dateIdentified: 2021-04-20; identificationRemarks: identified by DNA and morphology; **Event:** eventID: UK1_AB01_EB04; samplingProtocol: Brenke Epibenthic Sledge; eventDate: 2013-10-17; eventTime: 01:50; habitat: Abyssal plain; fieldNotes: Collected from epi net (on the epibenthic sledge); **Record Level:** language: en; institutionCode: NHMUK; collectionCode: ZOO; datasetName: ABYSSLINE; basisOfRecord: PreservedSpecimen
- **Type status:** Other material. **Occurrence:** recordNumber: NHM_0270; recordedBy: Adrian Glover | Helena Wiklund | Thomas Dahlgren | Magdalena Georgieva; individualCount: 1; preparations: Tissue voucher stored in 80% non-denatured ethanol aqueous solution | DNA voucher stored in buffer; otherCatalogNumbers: 0109405432 | 0174127319; associatedSequences: OQ746492 (16S) | OQ746811 (18S) | OQ738510 (COI); occurrenceID: A759970F-644A-5CD2-946C-6BB1542BE1E8; **Taxon:** taxonConceptID: Orbiniidae sp. (NHM_264); scientificName: Orbiniidae; kingdom: Animalia; phylum: Annelida; class: Polychaeta; family: Orbiniidae; taxonRank: family; scientificNameAuthorship: Hartman, 1942; **Location:** waterBody: Pacific; stateProvince: Clarion Clipperton Zone; locality: UK Seabed Resources Ltd exploration area UK-1 Stratum A; verbatimLocality: UK1 Stratum A; maximumDepthInMeters: 4128; locationRemarks: Deployment EB04; at Station G-I; from R/V Melville Cruise no. MV1313; verbatimLatitude: 13°45.21N; verbatimLongitude: 116°29.12W; decimalLatitude: 13.75583; decimalLongitude: -116.48667; geodeticDatum: WGS84; **Identification:** identifiedBy: Helena Wiklund | Lenka Neal | Thomas Dahlgren | Adrian Glover | Madeleine Brasier | Regan Drennan | Eva Stewart; dateIdentified: 2021-04-20; identificationRemarks: identified by DNA and morphology; **Event:** eventID: UK1_AB01_EB04; samplingProtocol: Brenke Epibenthic Sledge; eventDate: 2013-10-17; eventTime: 01:50; habitat: Abyssal plain; fieldNotes: Collected from epi net (on the epibenthic sledge); **Record Level:** language: en; institutionCode: NHMUK; collectionCode: ZOO; datasetName: ABYSSLINE; basisOfRecord: PreservedSpecimen

##### Distribution

Eastern Clarion-Clipperton Zone, central Pacific Ocean.

##### Diagnosis

**Figure 60.**
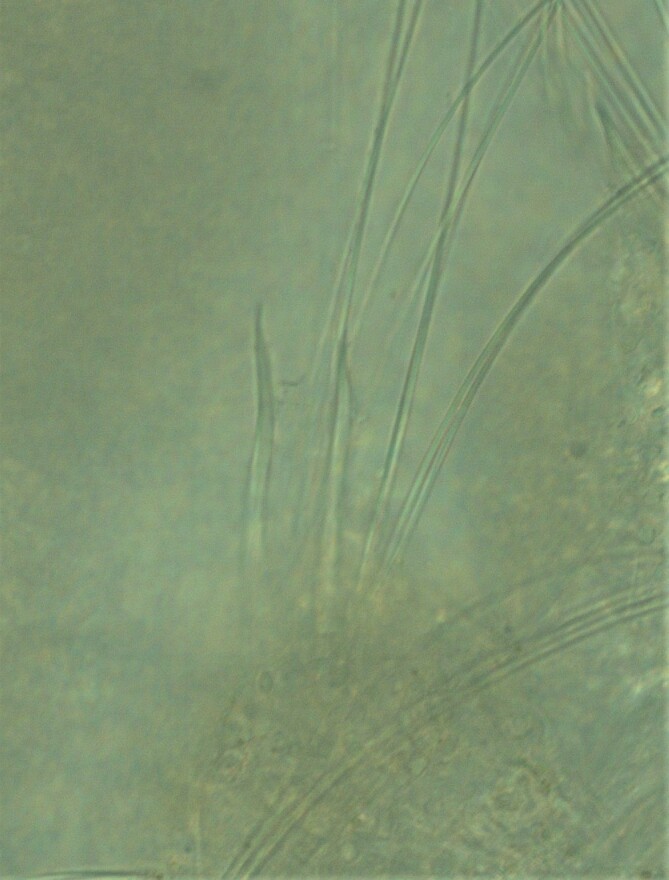
Orbiniidae sp. (NHM_264), neuropodial spines from specimen NHM_264.

Damaged specimens (Fig. 60) consistent with placement within family Orbiniidae, based on morphology and DNA.

#### Orbiniidae sp. (NHM_458)

AE6FBB35-2527-5918-A2A7-79F2186B7215

##### Materials

- **Type status:** Other material. **Occurrence:** catalogNumber: NHMUK ANEA 2023.432; recordNumber: NHM_0458; recordedBy: Adrian Glover | Helena Wiklund | Thomas Dahlgren | Magdalena Georgieva; individualCount: 1; preparations: specimen stored in 80% non-denatured ethanol aqueous solution | DNA voucher stored in buffer; otherCatalogNumbers: 0174127328; associatedSequences: OQ746521 (16S) | OQ746837 (18S); occurrenceID: A48B0CD4-44DA-5317-9899-AF4280AF029D; **Taxon:** taxonConceptID: Orbiniidae sp. (NHM_458); scientificName: Orbiniidae; kingdom: Animalia; phylum: Annelida; class: Polychaeta; family: Orbiniidae; taxonRank: family; scientificNameAuthorship: Hartman, 1942; **Location:** waterBody: Pacific; stateProvince: Clarion Clipperton Zone; locality: UK Seabed Resources Ltd exploration area UK-1 Stratum A; verbatimLocality: UK1 Stratum A; maximumDepthInMeters: 4163; locationRemarks: Deployment BC13; at Station J; from R/V Melville Cruise no. MV1313; verbatimLatitude: 13°54.099; verbatimLongitude: 116°35.400; decimalLatitude: 13.90165; decimalLongitude: -116.59; geodeticDatum: WGS84; **Identification:** identifiedBy: Helena Wiklund | Lenka Neal | Thomas Dahlgren | Adrian Glover | Madeleine Brasier | Regan Drennan | Eva Stewart; dateIdentified: 2021-04-20; identificationRemarks: identified by DNA and morphology; **Event:** eventID: UK1_AB01_BC13; samplingProtocol: USNEL Box Core; eventDate: 2013-10-21; eventTime: 13:27; habitat: Abyssal plain; fieldNotes: Collected from 0-2 cm layer of box core using a 300 micron sieve; **Record Level:** language: en; institutionCode: NHMUK; collectionCode: ZOO; datasetName: ABYSSLINE; basisOfRecord: PreservedSpecimen
- **Type status:** Other material. **Occurrence:** catalogNumber: NHMUK ANEA 2023.433; recordNumber: NHM_0567; recordedBy: Adrian Glover | Helena Wiklund | Thomas Dahlgren | Madeleine Brasier; individualCount: 1; preparations: specimen stored in 80% non-denatured ethanol aqueous solution | DNA voucher stored in buffer; otherCatalogNumbers: 0174126623; associatedSequences: OQ746528 (16S); occurrenceID: 065B1B78-084E-5A12-B60F-BFB054EC2557; **Taxon:** taxonConceptID: Orbiniidae sp. (NHM_458); scientificName: Orbiniidae; kingdom: Animalia; phylum: Annelida; class: Polychaeta; family: Orbiniidae; taxonRank: family; scientificNameAuthorship: Hartman, 1942; **Location:** waterBody: Pacific; stateProvince: Clarion Clipperton Zone; locality: UK Seabed Resources Ltd exploration area UK-1 Stratum B; verbatimLocality: UK1 Stratum B; maximumDepthInMeters: 4202; locationRemarks: Deployment EB01; at Station U2; from R/V Thomas G. Thompson Cruise no. TN319; verbatimLatitude: 12'23.17456; verbatimLongitude: 116'32.92021; decimalLatitude: 12.38624; decimalLongitude: -116.54867; geodeticDatum: WGS84; **Identification:** identifiedBy: Helena Wiklund | Lenka Neal | Thomas Dahlgren | Adrian Glover | Madeleine Brasier | Regan Drennan | Eva Stewart; dateIdentified: 2021-04-20; identificationRemarks: identified by DNA and morphology; **Event:** eventID: UK1_AB02_EB01; samplingProtocol: Brenke Epibenthic Sledge; eventDate: 2015-02-17; eventTime: 05:15; habitat: Abyssal plain; fieldNotes: Collected from epi net (on the epibenthic sledge); **Record Level:** language: en; institutionCode: NHMUK; collectionCode: ZOO; datasetName: ABYSSLINE; basisOfRecord: PreservedSpecimen

##### Distribution

Eastern Clarion-Clipperton Zone, central Pacific Ocean.

##### Diagnosis

**Figure 61.**
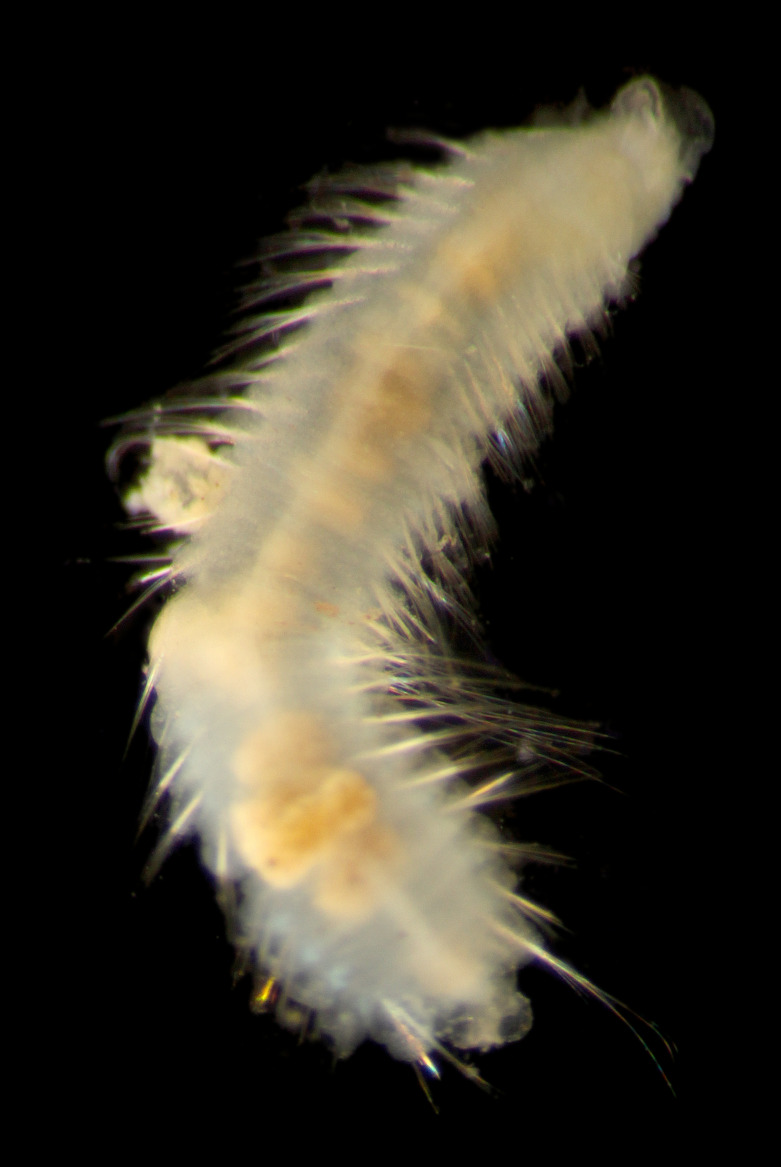
Orbiniidae sp. (NHM_458), anterior fragment of live specimen NHM_567.

Damaged specimens (Fig. 61) consistent with placement within family Orbiniidae, based on morphology and DNA.

#### Orbiniidae sp. (NHM_754)

5FC92107-446D-562F-8C71-A2F084AFB019

##### Materials

- **Type status:** Other material. **Occurrence:** catalogNumber: NHMUK ANEA 2023.435; recordNumber: NHM_1246; recordedBy: Adrian Glover | Helena Wiklund | Thomas Dahlgren | Madeleine Brasier; individualCount: 1; preparations: specimen stored in 80% non-denatured ethanol aqueous solution | DNA voucher stored in buffer; otherCatalogNumbers: 0174127332; associatedSequences: OQ746884 (18S) | OQ738574 (COI); occurrenceID: 5C6AB06F-75A0-528E-AD57-1A57D0A20389; **Taxon:** taxonConceptID: Orbiniidae sp. (NHM_754); scientificName: Orbiniidae; kingdom: Animalia; phylum: Annelida; class: Polychaeta; family: Orbiniidae; taxonRank: family; scientificNameAuthorship: Hartman, 1942; **Location:** waterBody: Pacific; stateProvince: Clarion Clipperton Zone; locality: Ocean Mineral Singapore exploration claim Stratum A; verbatimLocality: OMS Stratum A; maximumDepthInMeters: 4302; locationRemarks: Deployment EB06; at Station S5; from R/V Thomas G. Thompson Cruise no. TN319; verbatimLatitude: 12'15.44; verbatimLongitude: 117'18.13; decimalLatitude: 12.25733; decimalLongitude: -117.30217; geodeticDatum: WGS84; **Identification:** identifiedBy: Helena Wiklund | Lenka Neal | Thomas Dahlgren | Adrian Glover | Madeleine Brasier | Regan Drennan | Eva Stewart; dateIdentified: 2021-04-20; identificationRemarks: identified by DNA and morphology; **Event:** eventID: OMS1_AB02_EB06; samplingProtocol: Brenke Epibenthic Sledge; eventDate: 2015-03-01; eventTime: 04:02; habitat: Abyssal plain; fieldNotes: Collected from epi net (on the epibenthic sledge); **Record Level:** language: en; institutionCode: NHMUK; collectionCode: ZOO; datasetName: ABYSSLINE; basisOfRecord: PreservedSpecimen
- **Type status:** Other material. **Occurrence:** catalogNumber: NHMUK ANEA 2023.434; recordNumber: NHM_1071; recordedBy: Adrian Glover | Helena Wiklund | Thomas Dahlgren | Madeleine Brasier; individualCount: 1; preparations: specimen stored in 80% non-denatured ethanol aqueous solution | DNA voucher stored in buffer; otherCatalogNumbers: 0174126607; associatedSequences: OQ746612 (16S); occurrenceID: 8E004E54-B495-5138-B18C-CDD69AD5BD32; **Taxon:** taxonConceptID: Orbiniidae sp. (NHM_754); scientificName: Orbiniidae; kingdom: Animalia; phylum: Annelida; class: Polychaeta; family: Orbiniidae; taxonRank: family; scientificNameAuthorship: Hartman, 1942; **Location:** waterBody: Pacific; stateProvince: Clarion Clipperton Zone; locality: Ocean Mineral Singapore exploration claim Stratum A; verbatimLocality: OMS Stratum A; maximumDepthInMeters: 4100; locationRemarks: Deployment EB05; at Station S2; from R/V Thomas G. Thompson Cruise no. TN319; verbatimLatitude: 12'06.93; verbatimLongitude: 117'09.87; decimalLatitude: 12.1155; decimalLongitude: -117.1645; geodeticDatum: WGS84; **Identification:** identifiedBy: Helena Wiklund | Lenka Neal | Thomas Dahlgren | Adrian Glover | Madeleine Brasier | Regan Drennan | Eva Stewart; dateIdentified: 2021-04-20; identificationRemarks: identified by DNA and morphology; **Event:** eventID: OMS1_AB02_EB05; samplingProtocol: Brenke Epibenthic Sledge; eventDate: 2015-02-26; eventTime: 21:29; habitat: Abyssal plain; fieldNotes: Collected from epi net (on the epibenthic sledge); **Record Level:** language: en; institutionCode: NHMUK; collectionCode: ZOO; datasetName: ABYSSLINE; basisOfRecord: PreservedSpecimen
- **Type status:** Other material. **Occurrence:** recordNumber: NHM_1174B; recordedBy: Adrian Glover | Helena Wiklund | Thomas Dahlgren | Madeleine Brasier; individualCount: 1; preparations: Tissue voucher stored in 80% non-denatured ethanol aqueous solution | DNA voucher stored in buffer; otherCatalogNumbers: 0109405059 | 0174126614; associatedSequences: OQ746637 (16S) | OQ738567 (COI); occurrenceID: 13E031A8-2D48-570D-B770-6A697FA3DD16; **Taxon:** taxonConceptID: Orbiniidae sp. (NHM_754); scientificName: Orbiniidae; kingdom: Animalia; phylum: Annelida; class: Polychaeta; family: Orbiniidae; taxonRank: family; scientificNameAuthorship: Hartman, 1942; **Location:** waterBody: Pacific; stateProvince: Clarion Clipperton Zone; locality: Ocean Mineral Singapore exploration claim Stratum A; verbatimLocality: OMS Stratum A; maximumDepthInMeters: 4100; locationRemarks: Deployment EB05; at Station S2; from R/V Thomas G. Thompson Cruise no. TN319; verbatimLatitude: 12'06.93; verbatimLongitude: 117'09.87; decimalLatitude: 12.1155; decimalLongitude: -117.1645; geodeticDatum: WGS84; **Identification:** identifiedBy: Helena Wiklund | Lenka Neal | Thomas Dahlgren | Adrian Glover | Madeleine Brasier | Regan Drennan | Eva Stewart; dateIdentified: 2021-04-20; identificationRemarks: identified by DNA and morphology; **Event:** eventID: OMS1_AB02_EB05; samplingProtocol: Brenke Epibenthic Sledge; eventDate: 2015-02-26; eventTime: 21:29; habitat: Abyssal plain; fieldNotes: Collected from epi net (on the epibenthic sledge); **Record Level:** language: en; institutionCode: NHMUK; collectionCode: ZOO; datasetName: ABYSSLINE; basisOfRecord: PreservedSpecimen
- **Type status:** Other material. **Occurrence:** recordNumber: NHM_0754; recordedBy: Adrian Glover | Helena Wiklund | Thomas Dahlgren | Madeleine Brasier; individualCount: 1; preparations: Tissue voucher stored in 80% non-denatured ethanol aqueous solution | DNA voucher stored in buffer; otherCatalogNumbers: 0109405376 | 0174126554; associatedSequences: OQ746553 (16S); occurrenceID: 03BB386A-3AF0-50BB-B8C9-6BC22D444D8F; **Taxon:** taxonConceptID: Orbiniidae sp. (NHM_754); scientificName: Orbiniidae; kingdom: Animalia; phylum: Annelida; class: Polychaeta; family: Orbiniidae; taxonRank: family; scientificNameAuthorship: Hartman, 1942; **Location:** waterBody: Pacific; stateProvince: Clarion Clipperton Zone; locality: UK Seabed Resources Ltd exploration area UK-1 Stratum B; verbatimLocality: UK1 Stratum B; maximumDepthInMeters: 4425; locationRemarks: Deployment EB02; at Station U5; from R/V Thomas G. Thompson Cruise no. TN319; verbatimLatitude: 12'32.23; verbatimLongitude: 116'36.25; decimalLatitude: 12.53717; decimalLongitude: -116.60417; geodeticDatum: WGS84; **Identification:** identifiedBy: Helena Wiklund | Lenka Neal | Thomas Dahlgren | Adrian Glover | Madeleine Brasier | Regan Drennan | Eva Stewart; dateIdentified: 2021-04-20; identificationRemarks: identified by DNA and morphology; **Event:** eventID: UK1_AB02_EB02; samplingProtocol: Brenke Epibenthic Sledge; eventDate: 2015-02-20; eventTime: 06:24; habitat: Abyssal plain; fieldNotes: Collected from epi net (on the epibenthic sledge); **Record Level:** language: en; institutionCode: NHMUK; collectionCode: ZOO; datasetName: ABYSSLINE; basisOfRecord: PreservedSpecimen
- **Type status:** Other material. **Occurrence:** catalogNumber: NHMUK ANEA 2023.436; recordNumber: NHM_1505; recordedBy: Adrian Glover | Helena Wiklund | Thomas Dahlgren | Madeleine Brasier; individualCount: 1; preparations: specimen stored in 80% non-denatured ethanol aqueous solution | DNA voucher stored in buffer; otherCatalogNumbers: 0174126806; associatedSequences: OQ746687 (16S) | OQ746892 (18S); occurrenceID: 7CFFDB24-F9EA-5AD1-B248-1F3AD3CF9726; **Taxon:** taxonConceptID: Orbiniidae sp. (NHM_754); scientificName: Orbiniidae; kingdom: Animalia; phylum: Annelida; class: Polychaeta; family: Orbiniidae; taxonRank: family; scientificNameAuthorship: Hartman, 1942; **Location:** waterBody: Pacific; stateProvince: Clarion Clipperton Zone; locality: UK Seabed Resources Ltd exploration area UK-1 Stratum B; verbatimLocality: UK1 Stratum B; maximumDepthInMeters: 4196; locationRemarks: Deployment BC15; at Station U9; from R/V Thomas G. Thompson Cruise no. TN319; verbatimLatitude: 12'27.107; verbatimLongitude: 116'30.736; decimalLatitude: 12.45178; decimalLongitude: -116.51227; geodeticDatum: WGS84; **Identification:** identifiedBy: Helena Wiklund | Lenka Neal | Thomas Dahlgren | Adrian Glover | Madeleine Brasier | Regan Drennan | Eva Stewart; dateIdentified: 2021-04-20; identificationRemarks: identified by DNA and morphology; **Event:** eventID: UK1_AB02_BC15; samplingProtocol: USNEL Box Core; eventDate: 2015-03-04; eventTime: 23:23; habitat: Abyssal plain; fieldNotes: Collected from 0-2 cm layer of box core using a 300 micron sieve; **Record Level:** language: en; institutionCode: NHMUK; collectionCode: ZOO; datasetName: ABYSSLINE; basisOfRecord: PreservedSpecimen
- **Type status:** Other material. **Occurrence:** recordNumber: NHM_1508A; recordedBy: Adrian Glover | Helena Wiklund | Thomas Dahlgren | Madeleine Brasier; individualCount: 1; preparations: Tissue voucher stored in 80% non-denatured ethanol aqueous solution | DNA voucher stored in buffer; otherCatalogNumbers: 0109405354 | 0174126144; associatedSequences: OQ746688 (16S) | OQ738589 (COI); occurrenceID: 0C59A876-60CD-5087-87AA-0D7308C6535D; **Taxon:** taxonConceptID: Orbiniidae sp. (NHM_754); scientificName: Orbiniidae; kingdom: Animalia; phylum: Annelida; class: Polychaeta; family: Orbiniidae; taxonRank: family; scientificNameAuthorship: Hartman, 1942; **Location:** waterBody: Pacific; stateProvince: Clarion Clipperton Zone; locality: UK Seabed Resources Ltd exploration area UK-1 Stratum B; verbatimLocality: UK1 Stratum B; maximumDepthInMeters: 4196; locationRemarks: Deployment BC15; at Station U9; from R/V Thomas G. Thompson Cruise no. TN319; verbatimLatitude: 12'27.107; verbatimLongitude: 116'30.736; decimalLatitude: 12.45178; decimalLongitude: -116.51227; geodeticDatum: WGS84; **Identification:** identifiedBy: Helena Wiklund | Lenka Neal | Thomas Dahlgren | Adrian Glover | Madeleine Brasier | Regan Drennan | Eva Stewart; dateIdentified: 2021-04-20; identificationRemarks: identified by DNA and morphology; **Event:** eventID: UK1_AB02_BC15; samplingProtocol: USNEL Box Core; eventDate: 2015-03-04; eventTime: 23:23; habitat: Abyssal plain; fieldNotes: Collected from 0-2 cm layer of box core using a 300 micron sieve; **Record Level:** language: en; institutionCode: NHMUK; collectionCode: ZOO; datasetName: ABYSSLINE; basisOfRecord: PreservedSpecimen

##### Distribution

Eastern Clarion-Clipperton Zone, central Pacific Ocean.

##### Diagnosis

Damaged specimens (Fig. 62) consistent with placement within family Orbiniidae, based on morphology and DNA.

#### Orbiniidae sp. (NHM_791)

4F65A794-C5D4-5879-891B-C2D1C3BD13FD

##### Materials

- **Type status:** Other material. **Occurrence:** catalogNumber: NHMUK ANEA 2023.438; recordNumber: NHM_1024; recordedBy: Adrian Glover | Helena Wiklund | Thomas Dahlgren | Madeleine Brasier; individualCount: 1; preparations: specimen stored in 80% non-denatured ethanol aqueous solution | DNA voucher stored in buffer; otherCatalogNumbers: 0174126726; associatedSequences: OQ746607 (16S); occurrenceID: 66D690A1-CBD8-5490-B45E-1E9D1BF9D466; **Taxon:** taxonConceptID: Orbiniidae sp. (NHM_791); scientificName: Orbiniidae; kingdom: Animalia; phylum: Annelida; class: Polychaeta; family: Orbiniidae; taxonRank: family; scientificNameAuthorship: Hartman, 1942; **Location:** waterBody: Pacific; stateProvince: Clarion Clipperton Zone; locality: Ocean Mineral Singapore exploration claim Stratum A; verbatimLocality: OMS Stratum A; maximumDepthInMeters: 4122; locationRemarks: Deployment EB04; at Station S1; from R/V Thomas G. Thompson Cruise no. TN319; verbatimLatitude: 12'08.02; verbatimLongitude: 117'17.52; decimalLatitude: 12.13367; decimalLongitude: -117.292; geodeticDatum: WGS84; **Identification:** identifiedBy: Helena Wiklund | Lenka Neal | Thomas Dahlgren | Adrian Glover | Madeleine Brasier | Regan Drennan | Eva Stewart; dateIdentified: 2021-04-20; identificationRemarks: identified by DNA and morphology; **Event:** eventID: OMS1_AB02_EB04; samplingProtocol: Brenke Epibenthic Sledge; eventDate: 2015-02-24; eventTime: 19:10; habitat: Abyssal plain; fieldNotes: Collected from epi net (on the epibenthic sledge); **Record Level:** language: en; institutionCode: NHMUK; collectionCode: ZOO; datasetName: ABYSSLINE; basisOfRecord: PreservedSpecimen
- **Type status:** Other material. **Occurrence:** catalogNumber: NHMUK ANEA 2023.437; recordNumber: NHM_0791; recordedBy: Adrian Glover | Helena Wiklund | Thomas Dahlgren | Madeleine Brasier; individualCount: 1; preparations: specimen stored in 80% non-denatured ethanol aqueous solution | DNA voucher stored in buffer; otherCatalogNumbers: 0174126579; associatedSequences: OQ746562 (16S) | OQ746861 (18S); occurrenceID: 463FA1B8-BBED-55FC-81E8-FAE1F49E61EF; **Taxon:** taxonConceptID: Orbiniidae sp. (NHM_791); scientificName: Orbiniidae; kingdom: Animalia; phylum: Annelida; class: Polychaeta; family: Orbiniidae; taxonRank: family; scientificNameAuthorship: Hartman, 1942; **Location:** waterBody: Pacific; stateProvince: Clarion Clipperton Zone; locality: UK Seabed Resources Ltd exploration area UK-1 Stratum B; verbatimLocality: UK1 Stratum B; maximumDepthInMeters: 4425; locationRemarks: Deployment EB02; at Station U5; from R/V Thomas G. Thompson Cruise no. TN319; verbatimLatitude: 12'32.23; verbatimLongitude: 116'36.25; decimalLatitude: 12.53717; decimalLongitude: -116.60417; geodeticDatum: WGS84; **Identification:** identifiedBy: Helena Wiklund | Lenka Neal | Thomas Dahlgren | Adrian Glover | Madeleine Brasier | Regan Drennan | Eva Stewart; dateIdentified: 2021-04-20; identificationRemarks: identified by DNA and morphology; **Event:** eventID: UK1_AB02_EB02; samplingProtocol: Brenke Epibenthic Sledge; eventDate: 2015-02-20; eventTime: 06:24; habitat: Abyssal plain; fieldNotes: Collected from epi net (on the epibenthic sledge); **Record Level:** language: en; institutionCode: NHMUK; collectionCode: ZOO; datasetName: ABYSSLINE; basisOfRecord: PreservedSpecimen

##### Distribution

Eastern Clarion-Clipperton Zone, central Pacific Ocean.

##### Diagnosis

**Figure 63.**
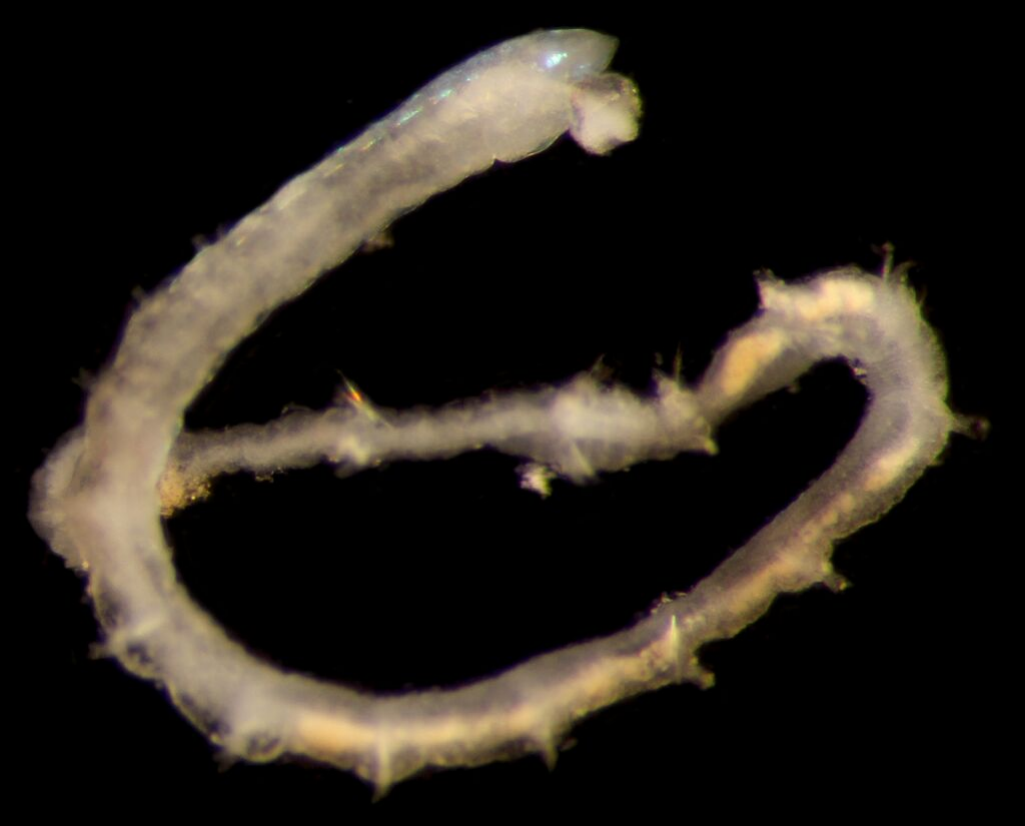
Orbiniidae sp. (NHM_791), anterior fragment of live specimen NHM_1024 in lateral view.

Damaged specimens (Fig. 63) consistent with placement within family Orbiniidae, based on morphology and DNA.

#### Orbiniidae sp. (NHM_824)

C0682E1D-9635-5569-AACC-47FA8DB69774

##### Materials

- **Type status:** Other material. **Occurrence:** catalogNumber: NHMUK ANEA 2023.443; recordNumber: NHM_1232; recordedBy: Adrian Glover | Helena Wiklund | Thomas Dahlgren | Madeleine Brasier; individualCount: 1; preparations: specimen stored in 80% non-denatured ethanol aqueous solution | DNA voucher stored in buffer; otherCatalogNumbers: 0174126774; associatedSequences: OQ746641 (16S) | OQ746881 (18S) | OQ738571 (COI); occurrenceID: 5FC70405-D9B2-5C65-90E8-D048C0E86E42; **Taxon:** taxonConceptID: Orbiniidae sp. (NHM_824); scientificName: Orbiniidae; kingdom: Animalia; phylum: Annelida; class: Polychaeta; family: Orbiniidae; taxonRank: family; scientificNameAuthorship: Hartman, 1942; **Location:** waterBody: Pacific; stateProvince: Clarion Clipperton Zone; locality: Ocean Mineral Singapore exploration claim Stratum A; verbatimLocality: OMS Stratum A; maximumDepthInMeters: 4090; locationRemarks: Deployment BC11; at Station S5; from R/V Thomas G. Thompson Cruise no. TN319; verbatimLatitude: 12'13.0425; verbatimLongitude: 117'19.5229; decimalLatitude: 12.21738; decimalLongitude: -117.32538; geodeticDatum: WGS84; **Identification:** identifiedBy: Helena Wiklund | Lenka Neal | Thomas Dahlgren | Adrian Glover | Madeleine Brasier | Regan Drennan | Eva Stewart; dateIdentified: 2021-04-20; identificationRemarks: identified by DNA and morphology; **Event:** eventID: OMS1_AB02_BC11; samplingProtocol: USNEL Box Core; eventDate: 2015-02-28; eventTime: 12:09; habitat: Abyssal plain; fieldNotes: Collected from 0-2 cm layer of box core using a 300 micron sieve; **Record Level:** language: en; institutionCode: NHMUK; collectionCode: ZOO; datasetName: ABYSSLINE; basisOfRecord: PreservedSpecimen
- **Type status:** Other material. **Occurrence:** catalogNumber: NHMUK ANEA 2023.445; recordNumber: NHM_1892; recordedBy: Adrian Glover | Helena Wiklund | Thomas Dahlgren | Madeleine Brasier; individualCount: 1; preparations: specimen stored in 80% non-denatured ethanol aqueous solution | DNA voucher stored in buffer; otherCatalogNumbers: 0174126755; associatedSequences: OQ746730 (16S) | OQ738603 (COI); occurrenceID: 661EA9D9-318E-5266-8FE7-BFE38F4BB380; **Taxon:** taxonConceptID: Orbiniidae sp. (NHM_824); scientificName: Orbiniidae; kingdom: Animalia; phylum: Annelida; class: Polychaeta; family: Orbiniidae; taxonRank: family; scientificNameAuthorship: Hartman, 1942; **Location:** waterBody: Pacific; stateProvince: Clarion Clipperton Zone; locality: Ocean Mineral Singapore exploration claim Stratum A; verbatimLocality: OMS Stratum A; maximumDepthInMeters: 4094; locationRemarks: Deployment EB11; at Station S10; from R/V Thomas G. Thompson Cruise no. TN319; verbatimLatitude: 12°02.49’; verbatimLongitude: 117°13.03’; decimalLatitude: 12.0415; decimalLongitude: -117.21717; geodeticDatum: WGS84; **Identification:** identifiedBy: Helena Wiklund | Lenka Neal | Thomas Dahlgren | Adrian Glover | Madeleine Brasier | Regan Drennan | Eva Stewart; dateIdentified: 2021-04-20; identificationRemarks: identified by DNA and morphology; **Event:** eventID: OMS1_AB02_EB11; samplingProtocol: Brenke Epibenthic Sledge; eventDate: 2015-03-13; habitat: Abyssal plain; fieldNotes: Collected from epi net (on the epibenthic sledge); **Record Level:** language: en; institutionCode: NHMUK; collectionCode: ZOO; datasetName: ABYSSLINE; basisOfRecord: PreservedSpecimen
- **Type status:** Other material. **Occurrence:** catalogNumber: NHMUK ANEA 2023.444; recordNumber: NHM_1736; recordedBy: Adrian Glover | Helena Wiklund | Thomas Dahlgren | Madeleine Brasier; individualCount: 1; preparations: specimen stored in 80% non-denatured ethanol aqueous solution | DNA voucher stored in buffer; otherCatalogNumbers: 0174123618; associatedSequences: OQ746708 (16S) | OQ738598 (COI); occurrenceID: 421D1604-424D-5232-98CD-D08515DBC54D; **Taxon:** taxonConceptID: Orbiniidae sp. (NHM_824); scientificName: Orbiniidae; kingdom: Animalia; phylum: Annelida; class: Polychaeta; family: Orbiniidae; taxonRank: family; scientificNameAuthorship: Hartman, 1942; **Location:** waterBody: Pacific; stateProvince: Clarion Clipperton Zone; locality: Ocean Mineral Singapore exploration claim Stratum A; verbatimLocality: OMS Stratum A; maximumDepthInMeters: 4045; locationRemarks: Deployment EB10; at Station S7; from R/V Thomas G. Thompson Cruise no. TN319; verbatimLatitude: 12'10.43; verbatimLongitude: 117'11.57; decimalLatitude: 12.17383; decimalLongitude: -117.19283; geodeticDatum: WGS84; **Identification:** identifiedBy: Helena Wiklund | Lenka Neal | Thomas Dahlgren | Adrian Glover | Madeleine Brasier | Regan Drennan | Eva Stewart; dateIdentified: 2021-04-20; identificationRemarks: identified by DNA and morphology; **Event:** eventID: OMS1_AB02_EB10; samplingProtocol: Brenke Epibenthic Sledge; eventDate: 2015-03-11; eventTime: 22:49; habitat: Abyssal plain; fieldNotes: Collected from epi net (on the epibenthic sledge); **Record Level:** language: en; institutionCode: NHMUK; collectionCode: ZOO; datasetName: ABYSSLINE; basisOfRecord: PreservedSpecimen
- **Type status:** Other material. **Occurrence:** catalogNumber: NHMUK ANEA 2023.440; recordNumber: NHM_1095; recordedBy: Adrian Glover | Helena Wiklund | Thomas Dahlgren | Madeleine Brasier; individualCount: 1; preparations: specimen stored in 80% non-denatured ethanol aqueous solution | DNA voucher stored in buffer; otherCatalogNumbers: 0174126780; associatedSequences: OQ746616 (16S) | OQ738556 (COI); occurrenceID: 282ADD5E-B662-5BC2-8527-0DD16BE58822; **Taxon:** taxonConceptID: Orbiniidae sp. (NHM_824); scientificName: Orbiniidae; kingdom: Animalia; phylum: Annelida; class: Polychaeta; family: Orbiniidae; taxonRank: family; scientificNameAuthorship: Hartman, 1942; **Location:** waterBody: Pacific; stateProvince: Clarion Clipperton Zone; locality: Ocean Mineral Singapore exploration claim Stratum A; verbatimLocality: OMS Stratum A; maximumDepthInMeters: 4100; locationRemarks: Deployment EB05; at Station S2; from R/V Thomas G. Thompson Cruise no. TN319; verbatimLatitude: 12'06.93; verbatimLongitude: 117'09.87; decimalLatitude: 12.1155; decimalLongitude: -117.1645; geodeticDatum: WGS84; **Identification:** identifiedBy: Helena Wiklund | Lenka Neal | Thomas Dahlgren | Adrian Glover | Madeleine Brasier | Regan Drennan | Eva Stewart; dateIdentified: 2021-04-20; identificationRemarks: identified by DNA and morphology; **Event:** eventID: OMS1_AB02_EB05; samplingProtocol: Brenke Epibenthic Sledge; eventDate: 2015-02-26; eventTime: 21:29; habitat: Abyssal plain; fieldNotes: Collected from epi net (on the epibenthic sledge); **Record Level:** language: en; institutionCode: NHMUK; collectionCode: ZOO; datasetName: ABYSSLINE; basisOfRecord: PreservedSpecimen
- **Type status:** Other material. **Occurrence:** catalogNumber: NHMUK ANEA 2023.441; recordNumber: NHM_1140; recordedBy: Adrian Glover | Helena Wiklund | Thomas Dahlgren | Madeleine Brasier; individualCount: 1; preparations: specimen stored in 80% non-denatured ethanol aqueous solution | DNA voucher stored in buffer; otherCatalogNumbers: 0174126779; associatedSequences: OQ746623 (16S) | OQ738560 (COI); occurrenceID: 8BBD02DA-B771-589A-93A5-B395EA0A11EF; **Taxon:** taxonConceptID: Orbiniidae sp. (NHM_824); scientificName: Orbiniidae; kingdom: Animalia; phylum: Annelida; class: Polychaeta; family: Orbiniidae; taxonRank: family; scientificNameAuthorship: Hartman, 1942; **Location:** waterBody: Pacific; stateProvince: Clarion Clipperton Zone; locality: Ocean Mineral Singapore exploration claim Stratum A; verbatimLocality: OMS Stratum A; maximumDepthInMeters: 4100; locationRemarks: Deployment EB05; at Station S2; from R/V Thomas G. Thompson Cruise no. TN319; verbatimLatitude: 12'06.93; verbatimLongitude: 117'09.87; decimalLatitude: 12.1155; decimalLongitude: -117.1645; geodeticDatum: WGS84; **Identification:** identifiedBy: Helena Wiklund | Lenka Neal | Thomas Dahlgren | Adrian Glover | Madeleine Brasier | Regan Drennan | Eva Stewart; dateIdentified: 2021-04-20; identificationRemarks: identified by DNA and morphology; **Event:** eventID: OMS1_AB02_EB05; samplingProtocol: Brenke Epibenthic Sledge; eventDate: 2015-02-26; eventTime: 21:29; habitat: Abyssal plain; fieldNotes: Collected from epi net (on the epibenthic sledge); **Record Level:** language: en; institutionCode: NHMUK; collectionCode: ZOO; datasetName: ABYSSLINE; basisOfRecord: PreservedSpecimen
- **Type status:** Other material. **Occurrence:** catalogNumber: NHMUK ANEA 2023.442; recordNumber: NHM_1151; recordedBy: Adrian Glover | Helena Wiklund | Thomas Dahlgren | Madeleine Brasier; individualCount: 1; preparations: specimen stored in 80% non-denatured ethanol aqueous solution | DNA voucher stored in buffer; otherCatalogNumbers: 0174126781; associatedSequences: OQ746625 (16S); occurrenceID: AFD6925B-577A-56AB-A407-D410EFD39D53; **Taxon:** taxonConceptID: Orbiniidae sp. (NHM_824); scientificName: Orbiniidae; kingdom: Animalia; phylum: Annelida; class: Polychaeta; family: Orbiniidae; taxonRank: family; scientificNameAuthorship: Hartman, 1942; **Location:** waterBody: Pacific; stateProvince: Clarion Clipperton Zone; locality: Ocean Mineral Singapore exploration claim Stratum A; verbatimLocality: OMS Stratum A; maximumDepthInMeters: 4100; locationRemarks: Deployment EB05; at Station S2; from R/V Thomas G. Thompson Cruise no. TN319; verbatimLatitude: 12'06.93; verbatimLongitude: 117'09.87; decimalLatitude: 12.1155; decimalLongitude: -117.1645; geodeticDatum: WGS84; **Identification:** identifiedBy: Helena Wiklund | Lenka Neal | Thomas Dahlgren | Adrian Glover | Madeleine Brasier | Regan Drennan | Eva Stewart; dateIdentified: 2021-04-20; identificationRemarks: identified by DNA and morphology; **Event:** eventID: OMS1_AB02_EB05; samplingProtocol: Brenke Epibenthic Sledge; eventDate: 2015-02-26; eventTime: 21:29; habitat: Abyssal plain; fieldNotes: Collected from epi net (on the epibenthic sledge); **Record Level:** language: en; institutionCode: NHMUK; collectionCode: ZOO; datasetName: ABYSSLINE; basisOfRecord: PreservedSpecimen
- **Type status:** Other material. **Occurrence:** catalogNumber: NHMUK ANEA 2023.439; recordNumber: NHM_0824; recordedBy: Adrian Glover | Helena Wiklund | Thomas Dahlgren | Madeleine Brasier; individualCount: 1; preparations: specimen stored in 80% non-denatured ethanol aqueous solution | DNA voucher stored in buffer; otherCatalogNumbers: 0174126728; associatedSequences: OQ746564 (16S); occurrenceID: 10D0108F-C623-5D49-BAAC-4737042D7FD0; **Taxon:** taxonConceptID: Orbiniidae sp. (NHM_824); scientificName: Orbiniidae; kingdom: Animalia; phylum: Annelida; class: Polychaeta; family: Orbiniidae; taxonRank: family; scientificNameAuthorship: Hartman, 1942; **Location:** waterBody: Pacific; stateProvince: Clarion Clipperton Zone; locality: UK Seabed Resources Ltd exploration area UK-1 Stratum B; verbatimLocality: UK1 Stratum B; maximumDepthInMeters: 4425; locationRemarks: Deployment EB02; at Station U5; from R/V Thomas G. Thompson Cruise no. TN319; verbatimLatitude: 12'32.23; verbatimLongitude: 116'36.25; decimalLatitude: 12.53717; decimalLongitude: -116.60417; geodeticDatum: WGS84; **Identification:** identifiedBy: Helena Wiklund | Lenka Neal | Thomas Dahlgren | Adrian Glover | Madeleine Brasier | Regan Drennan | Eva Stewart; dateIdentified: 2021-04-20; identificationRemarks: identified by DNA and morphology; **Event:** eventID: UK1_AB02_EB02; samplingProtocol: Brenke Epibenthic Sledge; eventDate: 2015-02-20; eventTime: 06:24; habitat: Abyssal plain; fieldNotes: Collected from epi net (on the epibenthic sledge); **Record Level:** language: en; institutionCode: NHMUK; collectionCode: ZOO; datasetName: ABYSSLINE; basisOfRecord: PreservedSpecimen

##### Distribution

Eastern Clarion-Clipperton Zone, central Pacific Ocean.

##### Diagnosis

**Figure 64.**
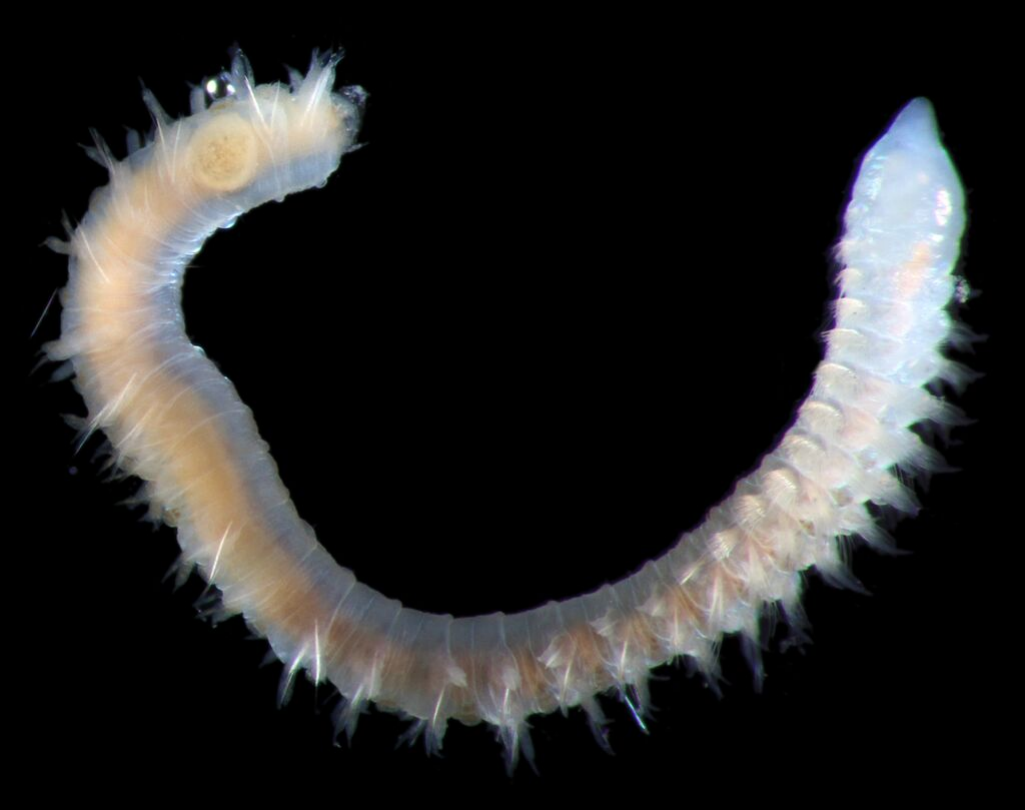
Orbiniidae sp. (NHM_824), anterior end of live specimen NHM_824 in lateral view.

Damaged specimens (Fig. 64) consistent with placement within family Orbiniidae, based on morphology and DNA.

#### Orbiniidae sp. (NHM_1947G)

E70D7F75-1BD9-5F05-9CFE-62582576837A

##### Materials

- **Type status:** Other material. **Occurrence:** catalogNumber: NHMUK ANEA 2023.430; recordNumber: NHM_1947G; recordedBy: Adrian Glover | Helena Wiklund | Thomas Dahlgren | Madeleine Brasier; individualCount: 1; preparations: specimen stored in 80% non-denatured ethanol aqueous solution | DNA voucher stored in buffer; associatedSequences: OQ746736 (16S); occurrenceID: E8050000-8529-5BE3-ACAE-BC0302C43FE8; **Taxon:** taxonConceptID: Orbiniidae sp. (NHM_1947G); scientificName: Orbiniidae; kingdom: Animalia; phylum: Annelida; class: Polychaeta; family: Orbiniidae; taxonRank: family; scientificNameAuthorship: Hartman, 1942; **Location:** waterBody: Pacific; stateProvince: Clarion Clipperton Zone; locality: Ocean Mineral Singapore exploration claim Stratum A; verbatimLocality: OMS Stratum A; maximumDepthInMeters: 4094; locationRemarks: Deployment EB11; at Station S10; from R/V Thomas G. Thompson Cruise no. TN319; verbatimLatitude: 12°02.49’; verbatimLongitude: 117°13.03’; decimalLatitude: 12.0415; decimalLongitude: -117.21717; geodeticDatum: WGS84; **Identification:** identifiedBy: Helena Wiklund | Lenka Neal | Thomas Dahlgren | Adrian Glover | Madeleine Brasier | Regan Drennan | Eva Stewart; dateIdentified: 2021-04-20; identificationRemarks: identified by DNA and morphology; **Event:** eventID: OMS1_AB02_EB11; samplingProtocol: Brenke Epibenthic Sledge; eventDate: 2015-03-13; habitat: Abyssal plain; fieldNotes: Collected from epi net (on the epibenthic sledge); **Record Level:** language: en; institutionCode: NHMUK; collectionCode: ZOO; datasetName: ABYSSLINE; basisOfRecord: PreservedSpecimen

##### Distribution

Eastern Clarion-Clipperton Zone, central Pacific Ocean.

##### Diagnosis

**Figure 65.**
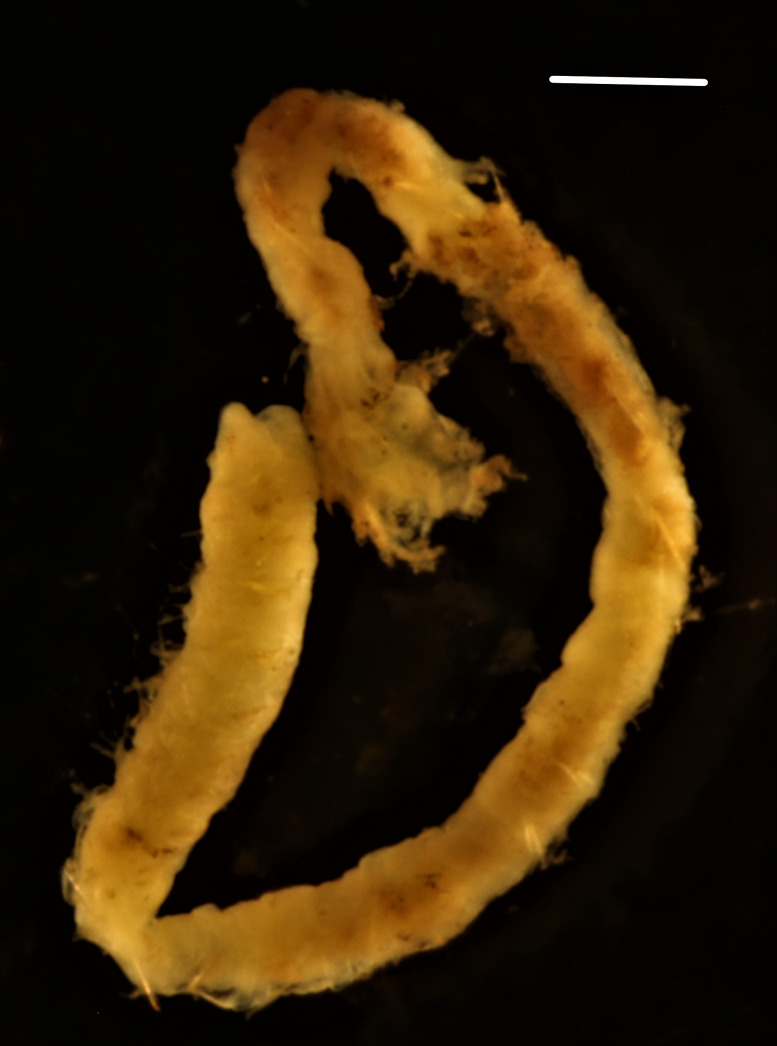
Orbiniidae sp. (NHM_1947), anterior fragment of preserved specimen NHM_1947G in lateral view.

Damaged specimen (Fig. 65) consistent with placement within family Orbiniidae, based on morphology and DNA.

#### Orbiniidae sp. (NHM_050)

574B7BB6-D582-5B25-99FA-6E15D3196E0F

##### Materials

- **Type status:** Other material. **Occurrence:** catalogNumber: NHMUK ANEA 2023.428; recordNumber: NHM_1348G; recordedBy: Adrian Glover | Helena Wiklund | Thomas Dahlgren | Madeleine Brasier; individualCount: 1; preparations: specimen stored in 80% non-denatured ethanol aqueous solution | DNA voucher stored in buffer; otherCatalogNumbers: 0174126611; associatedSequences: OQ746668 (16S) | OQ738578 (COI); occurrenceID: AE698CF0-20D8-56E2-BEB5-5784FB40597D; **Taxon:** taxonConceptID: Orbiniidae sp. (NHM_050); scientificName: Orbiniidae; kingdom: Animalia; phylum: Annelida; class: Polychaeta; family: Orbiniidae; taxonRank: family; scientificNameAuthorship: Hartman, 1942; **Location:** waterBody: Pacific; stateProvince: Clarion Clipperton Zone; locality: Ocean Mineral Singapore exploration claim Stratum A; verbatimLocality: OMS Stratum A; maximumDepthInMeters: 4302; locationRemarks: Deployment EB06; at Station S5; from R/V Thomas G. Thompson Cruise no. TN319; verbatimLatitude: 12'15.44; verbatimLongitude: 117'18.13; decimalLatitude: 12.25733; decimalLongitude: -117.30217; geodeticDatum: WGS84; **Identification:** identifiedBy: Helena Wiklund | Lenka Neal | Thomas Dahlgren | Adrian Glover | Madeleine Brasier | Regan Drennan | Eva Stewart; dateIdentified: 2021-04-20; identificationRemarks: identified by DNA and morphology; **Event:** eventID: OMS1_AB02_EB06; samplingProtocol: Brenke Epibenthic Sledge; eventDate: 2015-03-01; eventTime: 04:02; habitat: Abyssal plain; fieldNotes: Collected from epi net (on the epibenthic sledge); **Record Level:** language: en; institutionCode: NHMUK; collectionCode: ZOO; datasetName: ABYSSLINE; basisOfRecord: PreservedSpecimen
- **Type status:** Other material. **Occurrence:** catalogNumber: NHMUK ANEA 2023.427; recordNumber: NHM_0917E; recordedBy: Adrian Glover | Helena Wiklund | Thomas Dahlgren | Madeleine Brasier; individualCount: 1; preparations: specimen stored in 80% non-denatured ethanol aqueous solution | DNA voucher stored in buffer; otherCatalogNumbers: 0174126581; associatedSequences: OQ746587 (16S); occurrenceID: 31DABB85-0C10-5E3D-AC29-56438552CCE8; **Taxon:** taxonConceptID: Orbiniidae sp. (NHM_050); scientificName: Orbiniidae; kingdom: Animalia; phylum: Annelida; class: Polychaeta; family: Orbiniidae; taxonRank: family; scientificNameAuthorship: Hartman, 1942; **Location:** waterBody: Pacific; stateProvince: Clarion Clipperton Zone; locality: UK Seabed Resources Ltd exploration area UK-1 Stratum B; verbatimLocality: UK1 Stratum B; maximumDepthInMeters: 4198; locationRemarks: Deployment EB03; at Station U4; from R/V Thomas G. Thompson Cruise no. TN319; verbatimLatitude: 12'34.28; verbatimLongitude: 116'36.63; decimalLatitude: 12.57133; decimalLongitude: -116.6105; geodeticDatum: WGS84; **Identification:** identifiedBy: Helena Wiklund | Lenka Neal | Thomas Dahlgren | Adrian Glover | Madeleine Brasier | Regan Drennan | Eva Stewart; dateIdentified: 2021-04-20; identificationRemarks: identified by DNA and morphology; **Event:** eventID: UK1_AB02_EB03; samplingProtocol: Brenke Epibenthic Sledge; eventDate: 2015-02-23; eventTime: 05:39; habitat: Abyssal plain; fieldNotes: Collected from epi net (on the epibenthic sledge); **Record Level:** language: en; institutionCode: NHMUK; collectionCode: ZOO; datasetName: ABYSSLINE; basisOfRecord: PreservedSpecimen
- **Type status:** Other material. **Occurrence:** recordNumber: NHM_0050; recordedBy: Adrian Glover | Helena Wiklund | Thomas Dahlgren | Magdalena Georgieva; individualCount: 1; preparations: Tissue voucher stored in 80% non-denatured ethanol aqueous solution | DNA voucher stored in buffer; otherCatalogNumbers: 0109405374 | 0174127344; associatedSequences: OQ746472 (16S) | OQ746795 (18S) | OQ738498 (COI); occurrenceID: 167D7CD8-A41C-5B9B-8D25-F2C84F2EF1F3; **Taxon:** taxonConceptID: Orbiniidae sp. (NHM_050); scientificName: Orbiniidae; kingdom: Animalia; phylum: Annelida; class: Polychaeta; family: Orbiniidae; taxonRank: family; scientificNameAuthorship: Hartman, 1942; **Location:** waterBody: Pacific; stateProvince: Clarion Clipperton Zone; locality: UK Seabed Resources Ltd exploration area UK-1 Stratum A; verbatimLocality: UK1 Stratum A; maximumDepthInMeters: 4336; locationRemarks: Deployment EB01; at Station B-K-E; from R/V Melville Cruise no. MV1313; verbatimLatitude: 13°50.232; verbatimLongitude: 116°33.506; decimalLatitude: 13.8372; decimalLongitude: -116.55843; geodeticDatum: WGS84; **Identification:** identifiedBy: Helena Wiklund | Lenka Neal | Thomas Dahlgren | Adrian Glover | Madeleine Brasier | Regan Drennan | Eva Stewart; dateIdentified: 2021-04-20; identificationRemarks: identified by DNA and morphology; **Event:** eventID: UK1_AB01_EB01; samplingProtocol: Brenke Epibenthic Sledge; eventDate: 2013-10-09; eventTime: 10:26; habitat: Abyssal plain; fieldNotes: Collected from epi net (on the epibenthic sledge); **Record Level:** language: en; institutionCode: NHMUK; collectionCode: ZOO; datasetName: ABYSSLINE; basisOfRecord: PreservedSpecimen
- **Type status:** Other material. **Occurrence:** catalogNumber: NHMUK ANEA 2023.426; recordNumber: NHM_0489; recordedBy: Adrian Glover | Helena Wiklund | Thomas Dahlgren | Magdalena Georgieva; individualCount: 1; preparations: specimen stored in 80% non-denatured ethanol aqueous solution | DNA voucher stored in buffer; otherCatalogNumbers: 0174127311; associatedSequences: OQ746524 (16S) | OQ746841 (18S); occurrenceID: 6A60CF1B-BC6E-5F77-8F09-F77BA8CEF159; **Taxon:** taxonConceptID: Orbiniidae sp. (NHM_050); scientificName: Orbiniidae; kingdom: Animalia; phylum: Annelida; class: Polychaeta; family: Orbiniidae; taxonRank: family; scientificNameAuthorship: Hartman, 1942; **Location:** waterBody: Pacific; stateProvince: Clarion Clipperton Zone; locality: UK Seabed Resources Ltd exploration area UK-1 Stratum A; verbatimLocality: UK1 Stratum A; maximumDepthInMeters: 4128; locationRemarks: Deployment EB04; at Station G-I; from R/V Melville Cruise no. MV1313; verbatimLatitude: 13°45.21N; verbatimLongitude: 116°29.12W; decimalLatitude: 13.75583; decimalLongitude: -116.48667; geodeticDatum: WGS84; **Identification:** identifiedBy: Helena Wiklund | Lenka Neal | Thomas Dahlgren | Adrian Glover | Madeleine Brasier | Regan Drennan | Eva Stewart; dateIdentified: 2021-04-20; identificationRemarks: identified by DNA and morphology; **Event:** eventID: UK1_AB01_EB04; samplingProtocol: Brenke Epibenthic Sledge; eventDate: 2013-10-17; eventTime: 01:50; habitat: Abyssal plain; fieldNotes: Collected from epi net (on the epibenthic sledge); **Record Level:** language: en; institutionCode: NHMUK; collectionCode: ZOO; datasetName: ABYSSLINE; basisOfRecord: PreservedSpecimen

##### Distribution

Eastern Clarion-Clipperton Zone, central Pacific Ocean.

##### Diagnosis

**Figure 66.**
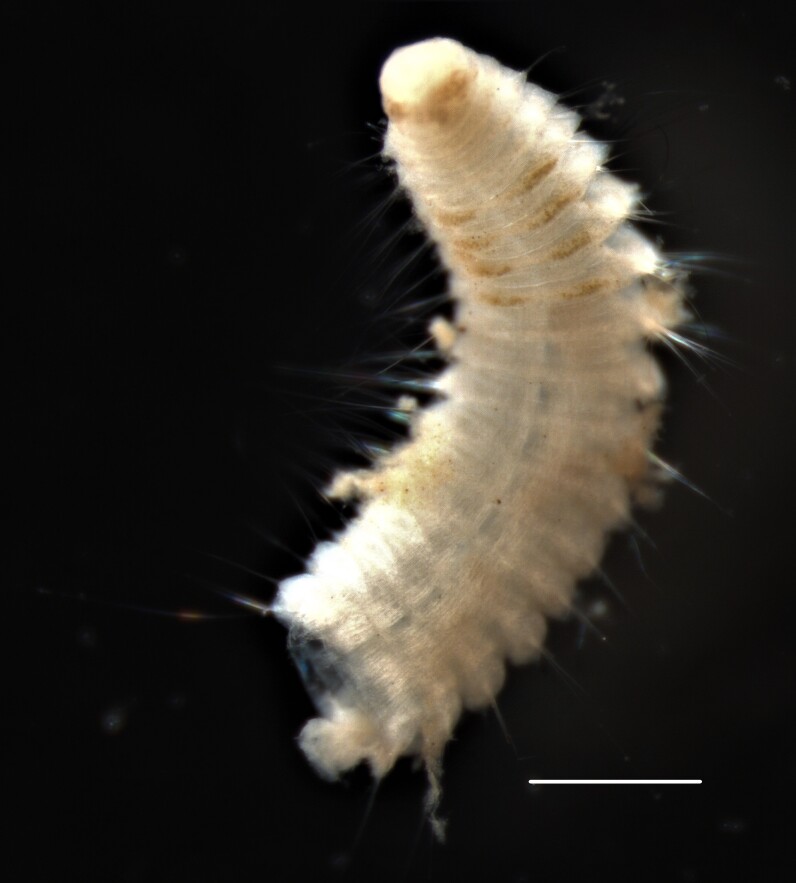
Orbiniidae sp. (NHM_050), anterior fragment of preserved specimen NHM_050 in lateral view. Scale bar 500 μm.

Damaged specimens (Fig. 66) consistent with placement within family Orbiniidae, based on morphology and DNA.

### Paraonidae Cerruti, 1909

#### Paraonidae sp. (NHM_059)

A5ECC138-DD5C-5050-B7BE-67650D8B352F

##### Materials

- **Type status:** Other material. **Occurrence:** catalogNumber: NHMUK ANEA 2023.451; recordNumber: NHM_1956; recordedBy: Adrian Glover | Helena Wiklund | Thomas Dahlgren | Madeleine Brasier; individualCount: 1; preparations: specimen stored in 80% non-denatured ethanol aqueous solution | DNA voucher stored in buffer; otherCatalogNumbers: 0174126209; associatedSequences: OQ746742 (16S); occurrenceID: 090DECDA-2272-58FD-9268-D2BACC243A45; **Taxon:** taxonConceptID: Paraonidae sp. (NHM_059); scientificName: Paraonidae; kingdom: Animalia; phylum: Annelida; class: Polychaeta; family: Paraonidae; taxonRank: family; scientificNameAuthorship: Cerruti, 1909; **Location:** waterBody: Pacific; stateProvince: Clarion Clipperton Zone; locality: Ocean Mineral Singapore exploration claim Stratum A; verbatimLocality: OMS Stratum A; maximumDepthInMeters: 4182; locationRemarks: Deployment BC24; at Station S8; from R/V Thomas G. Thompson Cruise no. TN319; verbatimLatitude: 12'11.406; verbatimLongitude: 117'22.282; decimalLatitude: 12.1901; decimalLongitude: -117.37137; geodeticDatum: WGS84; **Identification:** identifiedBy: Helena Wiklund | Lenka Neal | Thomas Dahlgren | Adrian Glover | Madeleine Brasier | Regan Drennan | Eva Stewart; dateIdentified: 2021-04-20; identificationRemarks: identified by DNA and morphology; **Event:** eventID: OMS1_AB02_BC24; samplingProtocol: USNEL Box Core; eventDate: 2015-03-14; eventTime: 05:16; habitat: Abyssal plain; fieldNotes: Collected from 0-2 cm layer of box core using a 300 micron sieve; **Record Level:** language: en; institutionCode: NHMUK; collectionCode: ZOO; datasetName: ABYSSLINE; basisOfRecord: PreservedSpecimen
- **Type status:** Other material. **Occurrence:** catalogNumber: NHMUK ANEA 2023.447; recordNumber: NHM_0244; recordedBy: Adrian Glover | Helena Wiklund | Thomas Dahlgren | Magdalena Georgieva; individualCount: 1; preparations: specimen stored in 80% non-denatured ethanol aqueous solution | DNA voucher stored in buffer; otherCatalogNumbers: 0174127315; associatedSequences: OQ746488 (16S) | OQ746808 (18S); occurrenceID: 7765656F-65DD-5808-A228-AED612F5282E; **Taxon:** taxonConceptID: Paraonidae sp. (NHM_059); scientificName: Paraonidae; kingdom: Animalia; phylum: Annelida; class: Polychaeta; family: Paraonidae; taxonRank: family; scientificNameAuthorship: Cerruti, 1909; **Location:** waterBody: Pacific; stateProvince: Clarion Clipperton Zone; locality: UK Seabed Resources Ltd exploration area UK-1 Stratum A; verbatimLocality: UK1 Stratum A; maximumDepthInMeters: 4076; locationRemarks: Deployment BC08; at Station F; from R/V Melville Cruise no. MV1313; verbatimLatitude: 13°48.700; verbatimLongitude: 116°42.600; decimalLatitude: 13.81167; decimalLongitude: -116.71; geodeticDatum: WGS84; **Identification:** identifiedBy: Helena Wiklund | Lenka Neal | Thomas Dahlgren | Adrian Glover | Madeleine Brasier | Regan Drennan | Eva Stewart; dateIdentified: 2021-04-20; identificationRemarks: identified by DNA and morphology; **Event:** eventID: UK1_AB01_BC08; samplingProtocol: USNEL Box Core; eventDate: 2013-10-16; eventTime: 14:33; habitat: Abyssal plain; fieldNotes: Collected from 0-2 cm layer of box core using a 300 micron sieve; **Record Level:** language: en; institutionCode: NHMUK; collectionCode: ZOO; datasetName: ABYSSLINE; basisOfRecord: PreservedSpecimen
- **Type status:** Other material. **Occurrence:** catalogNumber: NHMUK ANEA 2023.446; recordNumber: NHM_0059; recordedBy: Adrian Glover | Helena Wiklund | Thomas Dahlgren | Magdalena Georgieva; individualCount: 1; preparations: specimen stored in 80% non-denatured ethanol aqueous solution | DNA voucher stored in buffer; otherCatalogNumbers: 0174127350; associatedSequences: OQ746473 (16S) | OQ746796 (18S); occurrenceID: 1E143A86-996E-5894-8511-D1070D6391AB; **Taxon:** taxonConceptID: Paraonidae sp. (NHM_059); scientificName: Paraonidae; kingdom: Animalia; phylum: Annelida; class: Polychaeta; family: Paraonidae; taxonRank: family; scientificNameAuthorship: Cerruti, 1909; **Location:** waterBody: Pacific; stateProvince: Clarion Clipperton Zone; locality: UK Seabed Resources Ltd exploration area UK-1 Stratum A; verbatimLocality: UK1 Stratum A; maximumDepthInMeters: 4108; locationRemarks: Deployment BC04; at Station B; from R/V Melville Cruise no. MV1313; verbatimLatitude: 13°50.993; verbatimLongitude: 116°38.697; decimalLatitude: 13.84988; decimalLongitude: -116.64495; geodeticDatum: WGS84; **Identification:** identifiedBy: Helena Wiklund | Lenka Neal | Thomas Dahlgren | Adrian Glover | Madeleine Brasier | Regan Drennan | Eva Stewart; dateIdentified: 2021-04-20; identificationRemarks: identified by DNA and morphology; **Event:** eventID: UK1_AB01_BC04; samplingProtocol: USNEL Box Core; eventDate: 2013-10-09; eventTime: 17:34; habitat: Abyssal plain; fieldNotes: Collected from 0-2 cm layer of box core using a 300 micron sieve; **Record Level:** language: en; institutionCode: NHMUK; collectionCode: ZOO; datasetName: ABYSSLINE; basisOfRecord: PreservedSpecimen
- **Type status:** Other material. **Occurrence:** catalogNumber: NHMUK ANEA 2023.450; recordNumber: NHM_0915H; recordedBy: Adrian Glover | Helena Wiklund | Thomas Dahlgren | Madeleine Brasier; individualCount: 1; preparations: specimen stored in 80% non-denatured ethanol aqueous solution | DNA voucher stored in buffer; otherCatalogNumbers: 0174126532; associatedSequences: OQ746583 (16S); occurrenceID: 924C02AF-8192-5471-9C78-2194DC5551E7; **Taxon:** taxonConceptID: Paraonidae sp. (NHM_059); scientificName: Paraonidae; kingdom: Animalia; phylum: Annelida; class: Polychaeta; family: Paraonidae; taxonRank: family; scientificNameAuthorship: Cerruti, 1909; **Location:** waterBody: Pacific; stateProvince: Clarion Clipperton Zone; locality: UK Seabed Resources Ltd exploration area UK-1 Stratum B; verbatimLocality: UK1 Stratum B; maximumDepthInMeters: 4198; locationRemarks: Deployment EB03; at Station U4; from R/V Thomas G. Thompson Cruise no. TN319; verbatimLatitude: 12'34.28; verbatimLongitude: 116'36.63; decimalLatitude: 12.57133; decimalLongitude: -116.6105; geodeticDatum: WGS84; **Identification:** identifiedBy: Helena Wiklund | Lenka Neal | Thomas Dahlgren | Adrian Glover | Madeleine Brasier | Regan Drennan | Eva Stewart; dateIdentified: 2021-04-20; identificationRemarks: identified by DNA and morphology; **Event:** eventID: UK1_AB02_EB03; samplingProtocol: Brenke Epibenthic Sledge; eventDate: 2015-02-23; eventTime: 05:39; habitat: Abyssal plain; fieldNotes: Collected from epi net (on the epibenthic sledge); **Record Level:** language: en; institutionCode: NHMUK; collectionCode: ZOO; datasetName: ABYSSLINE; basisOfRecord: PreservedSpecimen
- **Type status:** Other material. **Occurrence:** catalogNumber: NHMUK ANEA 2023.448; recordNumber: NHM_0424; recordedBy: Adrian Glover | Helena Wiklund | Thomas Dahlgren | Magdalena Georgieva; individualCount: 1; preparations: specimen stored in 80% non-denatured ethanol aqueous solution | DNA voucher stored in buffer; otherCatalogNumbers: 0174127352; associatedSequences: OQ746515 (16S) | OQ746831 (18S); occurrenceID: 538D98FA-C165-5114-A5EA-154DEF3E4475; **Taxon:** taxonConceptID: Paraonidae sp. (NHM_059); scientificName: Paraonidae; kingdom: Animalia; phylum: Annelida; class: Polychaeta; family: Paraonidae; taxonRank: family; scientificNameAuthorship: Cerruti, 1909; **Location:** waterBody: Pacific; stateProvince: Clarion Clipperton Zone; locality: UK Seabed Resources Ltd exploration area UK-1 Stratum A; verbatimLocality: UK1 Stratum A; maximumDepthInMeters: 4011; locationRemarks: Deployment RV06; at Station K; from R/V Melville Cruise no. MV1313; decimalLatitude: 13.86367; decimalLongitude: -116.54432; geodeticDatum: WGS84; **Identification:** identifiedBy: Helena Wiklund | Lenka Neal | Thomas Dahlgren | Adrian Glover | Madeleine Brasier | Regan Drennan | Eva Stewart; dateIdentified: 2021-04-20; identificationRemarks: identified by DNA and morphology; **Event:** eventID: UK1_AB01_RV06; samplingProtocol: Remotely Operated Vehicle; eventDate: 2013-10-20; eventTime: 10:32; habitat: Abyssal plain; **Record Level:** language: en; institutionCode: NHMUK; collectionCode: ZOO; datasetName: ABYSSLINE; basisOfRecord: PreservedSpecimen
- **Type status:** Other material. **Occurrence:** catalogNumber: NHMUK ANEA 2023.449; recordNumber: NHM_0429; recordedBy: Adrian Glover | Helena Wiklund | Thomas Dahlgren | Magdalena Georgieva; individualCount: 1; preparations: specimen stored in 80% non-denatured ethanol aqueous solution | DNA voucher stored in buffer; otherCatalogNumbers: 0174127335; associatedSequences: OQ746516 (16S) | OQ746832 (18S); occurrenceID: 2F23F3EC-468A-5A19-99B7-799C0EBA30A4; **Taxon:** taxonConceptID: Paraonidae sp. (NHM_059); scientificName: Paraonidae; kingdom: Animalia; phylum: Annelida; class: Polychaeta; family: Paraonidae; taxonRank: family; scientificNameAuthorship: Cerruti, 1909; **Location:** waterBody: Pacific; stateProvince: Clarion Clipperton Zone; locality: UK Seabed Resources Ltd exploration area UK-1 Stratum A; verbatimLocality: UK1 Stratum A; maximumDepthInMeters: 4011; locationRemarks: Deployment RV06; at Station K; from R/V Melville Cruise no. MV1313; decimalLatitude: 13.86367; decimalLongitude: -116.54432; geodeticDatum: WGS84; **Identification:** identifiedBy: Helena Wiklund | Lenka Neal | Thomas Dahlgren | Adrian Glover | Madeleine Brasier | Regan Drennan | Eva Stewart; dateIdentified: 2021-04-20; identificationRemarks: identified by DNA and morphology; **Event:** eventID: UK1_AB01_RV06; samplingProtocol: Remotely Operated Vehicle; eventDate: 2013-10-20; eventTime: 10:32; habitat: Abyssal plain; **Record Level:** language: en; institutionCode: NHMUK; collectionCode: ZOO; datasetName: ABYSSLINE; basisOfRecord: PreservedSpecimen
- **Type status:** Other material. **Occurrence:** recordNumber: NHM_0436; recordedBy: Adrian Glover | Helena Wiklund | Thomas Dahlgren | Magdalena Georgieva; individualCount: 1; preparations: Tissue voucher stored in 80% non-denatured ethanol aqueous solution | DNA voucher stored in buffer; otherCatalogNumbers: 0109405351 | 0174127385; associatedSequences: OQ746519 (16S) | OQ746835 (18S); occurrenceID: 34B673E7-B7E3-5DAF-B7DA-C34F67288BC6; **Taxon:** taxonConceptID: Paraonidae sp. (NHM_059); scientificName: Paraonidae; kingdom: Animalia; phylum: Annelida; class: Polychaeta; family: Paraonidae; taxonRank: family; scientificNameAuthorship: Cerruti, 1909; **Location:** waterBody: Pacific; stateProvince: Clarion Clipperton Zone; locality: UK Seabed Resources Ltd exploration area UK-1 Stratum A; verbatimLocality: UK1 Stratum A; maximumDepthInMeters: 4011; locationRemarks: Deployment RV06; at Station K; from R/V Melville Cruise no. MV1313; decimalLatitude: 13.86367; decimalLongitude: -116.54432; geodeticDatum: WGS84; **Identification:** identifiedBy: Helena Wiklund | Lenka Neal | Thomas Dahlgren | Adrian Glover | Madeleine Brasier | Regan Drennan | Eva Stewart; dateIdentified: 2021-04-20; identificationRemarks: identified by DNA and morphology; **Event:** eventID: UK1_AB01_RV06; samplingProtocol: Remotely Operated Vehicle; eventDate: 2013-10-20; eventTime: 10:32; habitat: Abyssal plain; **Record Level:** language: en; institutionCode: NHMUK; collectionCode: ZOO; datasetName: ABYSSLINE; basisOfRecord: PreservedSpecimen

##### Distribution

Eastern Clarion-Clipperton Zone, central Pacific Ocean.

##### Diagnosis

**Figure 67.**
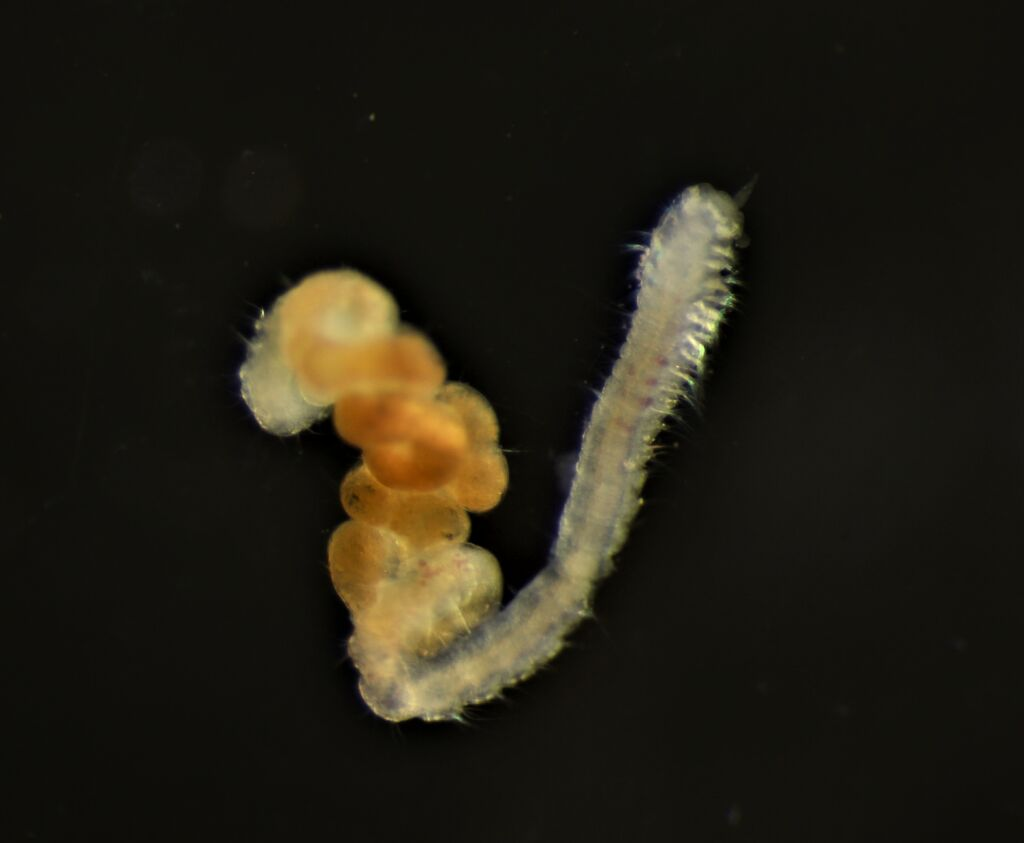
Paraonidae sp. (NHM_059), live specimen NHM_059 in ventral view.

Damaged specimens (Fig. 67) consistent with placement within family Paraonidae, based on morphology and DNA.

#### Paraonidae sp. (NHM_177)

9966DA7E-4AEE-54E9-A83B-92FD2AB8C984

##### Materials

- **Type status:** Other material. **Occurrence:** catalogNumber: NHMUK ANEA 2023.453; recordNumber: NHM_0177; recordedBy: Adrian Glover | Helena Wiklund | Thomas Dahlgren | Magdalena Georgieva; individualCount: 1; preparations: specimen stored in 80% non-denatured ethanol aqueous solution | DNA voucher stored in buffer; otherCatalogNumbers: 0174127313; associatedSequences: OQ746484 (16S) | OQ746805 (18S) | OQ738505 (COI); occurrenceID: 87913991-001C-5234-BBB8-1343FA5451BF; **Taxon:** taxonConceptID: Paraonidae sp. (NHM_177); scientificName: Paraonidae; kingdom: Animalia; phylum: Annelida; class: Polychaeta; family: Paraonidae; taxonRank: family; scientificNameAuthorship: Cerruti, 1909; **Location:** waterBody: Pacific; stateProvince: Clarion Clipperton Zone; locality: UK Seabed Resources Ltd exploration area UK-1 Stratum A; verbatimLocality: UK1 Stratum A; maximumDepthInMeters: 4082; locationRemarks: Deployment EB03; at Station D; from R/V Melville Cruise no. MV1313; verbatimLatitude: 13°56.089; verbatimLongitude: 116°33.011; decimalLatitude: 13.93482; decimalLongitude: -116.55018; geodeticDatum: WGS84; **Identification:** identifiedBy: Helena Wiklund | Lenka Neal | Thomas Dahlgren | Adrian Glover | Madeleine Brasier | Regan Drennan | Eva Stewart; dateIdentified: 2021-04-20; identificationRemarks: identified by DNA and morphology; **Event:** eventID: UK1_AB01_EB03; samplingProtocol: Brenke Epibenthic Sledge; eventDate: 2013-10-13; eventTime: 12:40; habitat: Abyssal plain; fieldNotes: Collected from epi net (on the epibenthic sledge); **Record Level:** language: en; institutionCode: NHMUK; collectionCode: ZOO; datasetName: ABYSSLINE; basisOfRecord: PreservedSpecimen
- **Type status:** Other material. **Occurrence:** recordNumber: NHM_0304; recordedBy: Adrian Glover | Helena Wiklund | Thomas Dahlgren | Magdalena Georgieva; individualCount: 1; preparations: Tissue voucher stored in 80% non-denatured ethanol aqueous solution | DNA voucher stored in buffer; otherCatalogNumbers: 0109405423 | 0174127300; associatedSequences: OQ746494 (16S) | OQ746813 (18S) | OQ738512 (COI); occurrenceID: 507DE5DB-6967-571D-B901-C14E3C74DE39; **Taxon:** taxonConceptID: Paraonidae sp. (NHM_177); scientificName: Paraonidae; kingdom: Animalia; phylum: Annelida; class: Polychaeta; family: Paraonidae; taxonRank: family; scientificNameAuthorship: Cerruti, 1909; **Location:** waterBody: Pacific; stateProvince: Clarion Clipperton Zone; locality: UK Seabed Resources Ltd exploration area UK-1 Stratum A; verbatimLocality: UK1 Stratum A; maximumDepthInMeters: 4110; locationRemarks: Deployment BC09; at Station G; from R/V Melville Cruise no. MV1313; verbatimLatitude: 13°45.726; verbatimLongitude: 116°27.825; decimalLatitude: 13.7621; decimalLongitude: -116.46375; geodeticDatum: WGS84; **Identification:** identifiedBy: Helena Wiklund | Lenka Neal | Thomas Dahlgren | Adrian Glover | Madeleine Brasier | Regan Drennan | Eva Stewart; dateIdentified: 2021-04-20; identificationRemarks: identified by DNA and morphology; **Event:** eventID: UK1_AB01_BC09; samplingProtocol: USNEL Box Core; eventDate: 2013-10-17; eventTime: 13:40; habitat: Abyssal plain; fieldNotes: Collected from 0-2 cm layer of box core using a 300 micron sieve; **Record Level:** language: en; institutionCode: NHMUK; collectionCode: ZOO; datasetName: ABYSSLINE; basisOfRecord: PreservedSpecimen

##### Distribution

Eastern Clarion-Clipperton Zone, central Pacific Ocean.

##### Diagnosis

**Figure 68.**
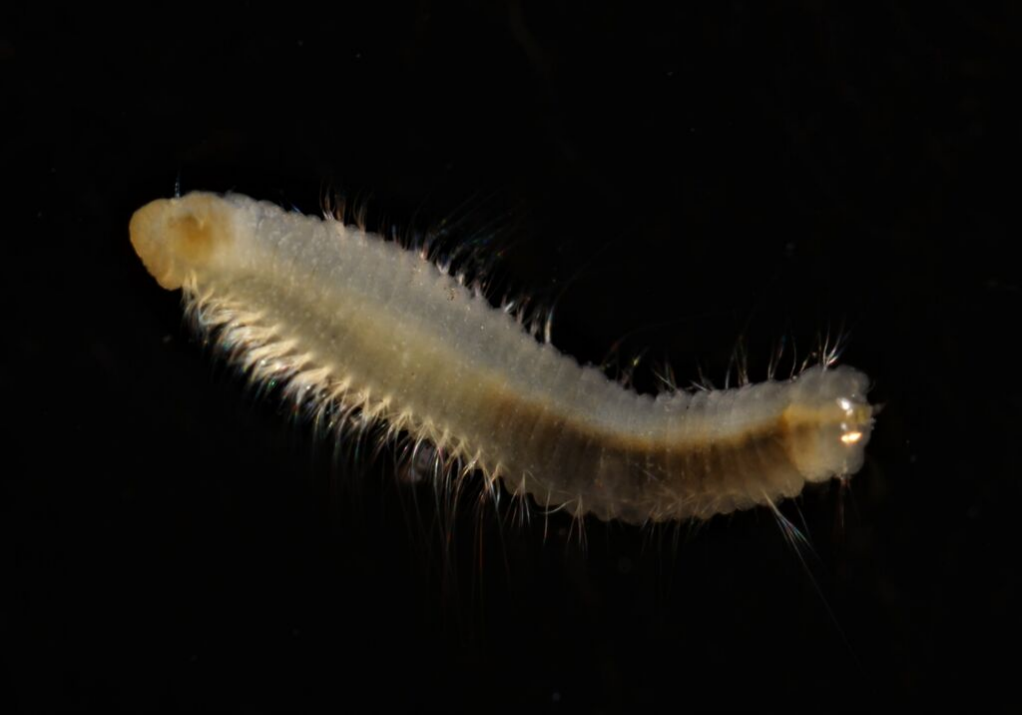
Paraonidae sp. (NHM_177), anterior fragment of live specimen NHM_177 in ventral view.

Damaged specimens (Fig. 68) consistent with placement within family Paraonidae, based on morphology and DNA.

#### Paraonidae sp. (NHM_332)

E96E0F2B-73D9-587F-AE0F-E2019A0BAE3E

##### Materials

- **Type status:** Other material. **Occurrence:** catalogNumber: NHMUK ANEA 2023.459; recordNumber: NHM_1320; recordedBy: Adrian Glover | Helena Wiklund | Thomas Dahlgren | Madeleine Brasier; individualCount: 1; preparations: specimen stored in 80% non-denatured ethanol aqueous solution | DNA voucher stored in buffer; otherCatalogNumbers: 0174126565; associatedSequences: OQ746659 (16S); occurrenceID: 15C39F1F-43A7-5AD6-A263-7491CFABADA7; **Taxon:** taxonConceptID: Paraonidae sp. (NHM_332); scientificName: Paraonidae; kingdom: Animalia; phylum: Annelida; class: Polychaeta; family: Paraonidae; taxonRank: family; scientificNameAuthorship: Cerruti, 1909; **Location:** waterBody: Pacific; stateProvince: Clarion Clipperton Zone; locality: Ocean Mineral Singapore exploration claim Stratum A; verbatimLocality: OMS Stratum A; maximumDepthInMeters: 4302; locationRemarks: Deployment EB06; at Station S5; from R/V Thomas G. Thompson Cruise no. TN319; verbatimLatitude: 12'15.44; verbatimLongitude: 117'18.13; decimalLatitude: 12.25733; decimalLongitude: -117.30217; geodeticDatum: WGS84; **Identification:** identifiedBy: Helena Wiklund | Lenka Neal | Thomas Dahlgren | Adrian Glover | Madeleine Brasier | Regan Drennan | Eva Stewart; dateIdentified: 2021-04-20; identificationRemarks: identified by DNA and morphology; **Event:** eventID: OMS1_AB02_EB06; samplingProtocol: Brenke Epibenthic Sledge; eventDate: 2015-03-01; eventTime: 04:02; habitat: Abyssal plain; fieldNotes: Collected from epi net (on the epibenthic sledge); **Record Level:** language: en; institutionCode: NHMUK; collectionCode: ZOO; datasetName: ABYSSLINE; basisOfRecord: PreservedSpecimen
- **Type status:** Other material. **Occurrence:** catalogNumber: NHMUK ANEA 2023.458; recordNumber: NHM_1005; recordedBy: Adrian Glover | Helena Wiklund | Thomas Dahlgren | Madeleine Brasier; individualCount: 1; preparations: specimen stored in 80% non-denatured ethanol aqueous solution | DNA voucher stored in buffer; otherCatalogNumbers: 0174126569; associatedSequences: OQ746603 (16S); occurrenceID: C7870CCB-8E8E-55BE-A6B4-55AC2E7D2654; **Taxon:** taxonConceptID: Paraonidae sp. (NHM_332); scientificName: Paraonidae; kingdom: Animalia; phylum: Annelida; class: Polychaeta; family: Paraonidae; taxonRank: family; scientificNameAuthorship: Cerruti, 1909; **Location:** waterBody: Pacific; stateProvince: Clarion Clipperton Zone; locality: Ocean Mineral Singapore exploration claim Stratum A; verbatimLocality: OMS Stratum A; maximumDepthInMeters: 4122; locationRemarks: Deployment EB04; at Station S1; from R/V Thomas G. Thompson Cruise no. TN319; verbatimLatitude: 12'08.02; verbatimLongitude: 117'17.52; decimalLatitude: 12.13367; decimalLongitude: -117.292; geodeticDatum: WGS84; **Identification:** identifiedBy: Helena Wiklund | Lenka Neal | Thomas Dahlgren | Adrian Glover | Madeleine Brasier | Regan Drennan | Eva Stewart; dateIdentified: 2021-04-20; identificationRemarks: identified by DNA and morphology; **Event:** eventID: OMS1_AB02_EB04; samplingProtocol: Brenke Epibenthic Sledge; eventDate: 2015-02-24; eventTime: 19:10; habitat: Abyssal plain; fieldNotes: Collected from epi net (on the epibenthic sledge); **Record Level:** language: en; institutionCode: NHMUK; collectionCode: ZOO; datasetName: ABYSSLINE; basisOfRecord: PreservedSpecimen
- **Type status:** Other material. **Occurrence:** catalogNumber: NHMUK ANEA 2023.460; recordNumber: NHM_1797K; recordedBy: Adrian Glover | Helena Wiklund | Thomas Dahlgren | Madeleine Brasier; individualCount: 1; preparations: specimen stored in 80% non-denatured ethanol aqueous solution | DNA voucher stored in buffer; otherCatalogNumbers: 0174126210; associatedSequences: OQ746723 (16S); occurrenceID: 28700313-3481-505B-AF55-054C718839DF; **Taxon:** taxonConceptID: Paraonidae sp. (NHM_332); scientificName: Paraonidae; kingdom: Animalia; phylum: Annelida; class: Polychaeta; family: Paraonidae; taxonRank: family; scientificNameAuthorship: Cerruti, 1909; **Location:** waterBody: Pacific; stateProvince: Clarion Clipperton Zone; locality: Ocean Mineral Singapore exploration claim Stratum A; verbatimLocality: OMS Stratum A; maximumDepthInMeters: 4045; locationRemarks: Deployment EB10; at Station S7; from R/V Thomas G. Thompson Cruise no. TN319; verbatimLatitude: 12'10.43; verbatimLongitude: 117'11.57; decimalLatitude: 12.17383; decimalLongitude: -117.19283; geodeticDatum: WGS84; **Identification:** identifiedBy: Helena Wiklund | Lenka Neal | Thomas Dahlgren | Adrian Glover | Madeleine Brasier | Regan Drennan | Eva Stewart; dateIdentified: 2021-04-20; identificationRemarks: identified by DNA and morphology; **Event:** eventID: OMS1_AB02_EB10; samplingProtocol: Brenke Epibenthic Sledge; eventDate: 2015-03-11; eventTime: 22:49; habitat: Abyssal plain; fieldNotes: Collected from epi net (on the epibenthic sledge); **Record Level:** language: en; institutionCode: NHMUK; collectionCode: ZOO; datasetName: ABYSSLINE; basisOfRecord: PreservedSpecimen
- **Type status:** Other material. **Occurrence:** catalogNumber: NHMUK ANEA 2023.456; recordNumber: NHM_0419; recordedBy: Adrian Glover | Helena Wiklund | Thomas Dahlgren | Magdalena Georgieva; individualCount: 1; preparations: specimen stored in 80% non-denatured ethanol aqueous solution | DNA voucher stored in buffer; otherCatalogNumbers: 0174127359; associatedSequences: OQ746513 (16S) | OQ746829 (18S); occurrenceID: CF1F0581-60D4-5F04-AFD3-65644885680E; **Taxon:** taxonConceptID: Paraonidae sp. (NHM_332); scientificName: Paraonidae; kingdom: Animalia; phylum: Annelida; class: Polychaeta; family: Paraonidae; taxonRank: family; scientificNameAuthorship: Cerruti, 1909; **Location:** waterBody: Pacific; stateProvince: Clarion Clipperton Zone; locality: UK Seabed Resources Ltd exploration area UK-1 Stratum A; verbatimLocality: UK1 Stratum A; maximumDepthInMeters: 4011; locationRemarks: Deployment RV06; at Station K; from R/V Melville Cruise no. MV1313; decimalLatitude: 13.86367; decimalLongitude: -116.54432; geodeticDatum: WGS84; **Identification:** identifiedBy: Helena Wiklund | Lenka Neal | Thomas Dahlgren | Adrian Glover | Madeleine Brasier | Regan Drennan | Eva Stewart; dateIdentified: 2021-04-20; identificationRemarks: identified by DNA and morphology; **Event:** eventID: UK1_AB01_RV06; samplingProtocol: Remotely Operated Vehicle; eventDate: 2013-10-20; eventTime: 10:32; habitat: Abyssal plain; **Record Level:** language: en; institutionCode: NHMUK; collectionCode: ZOO; datasetName: ABYSSLINE; basisOfRecord: PreservedSpecimen
- **Type status:** Other material. **Occurrence:** catalogNumber: NHMUK ANEA 2023.457; recordNumber: NHM_0423; recordedBy: Adrian Glover | Helena Wiklund | Thomas Dahlgren | Magdalena Georgieva; individualCount: 1; preparations: specimen stored in 80% non-denatured ethanol aqueous solution | DNA voucher stored in buffer; otherCatalogNumbers: 0174127314; associatedSequences: OQ746514 (16S) | OQ746830 (18S) | OQ738521 (COI); occurrenceID: 30F3C55C-55D5-5998-A547-D2BFF518E842; **Taxon:** taxonConceptID: Paraonidae sp. (NHM_332); scientificName: Paraonidae; kingdom: Animalia; phylum: Annelida; class: Polychaeta; family: Paraonidae; taxonRank: family; scientificNameAuthorship: Cerruti, 1909; **Location:** waterBody: Pacific; stateProvince: Clarion Clipperton Zone; locality: UK Seabed Resources Ltd exploration area UK-1 Stratum A; verbatimLocality: UK1 Stratum A; maximumDepthInMeters: 4011; locationRemarks: Deployment RV06; at Station K; from R/V Melville Cruise no. MV1313; decimalLatitude: 13.86367; decimalLongitude: -116.54432; geodeticDatum: WGS84; **Identification:** identifiedBy: Helena Wiklund | Lenka Neal | Thomas Dahlgren | Adrian Glover | Madeleine Brasier | Regan Drennan | Eva Stewart; dateIdentified: 2021-04-20; identificationRemarks: identified by DNA and morphology; **Event:** eventID: UK1_AB01_RV06; samplingProtocol: Remotely Operated Vehicle; eventDate: 2013-10-20; eventTime: 10:32; habitat: Abyssal plain; **Record Level:** language: en; institutionCode: NHMUK; collectionCode: ZOO; datasetName: ABYSSLINE; basisOfRecord: PreservedSpecimen
- **Type status:** Other material. **Occurrence:** catalogNumber: NHMUK ANEA 2023.455; recordNumber: NHM_0332; recordedBy: Adrian Glover | Helena Wiklund | Thomas Dahlgren | Magdalena Georgieva; individualCount: 1; preparations: specimen stored in 80% non-denatured ethanol aqueous solution | DNA voucher stored in buffer; otherCatalogNumbers: 0174127375; associatedSequences: OQ746497 (16S); occurrenceID: 73716274-AB6E-5E26-B67A-09B52BF746D0; **Taxon:** taxonConceptID: Paraonidae sp. (NHM_332); scientificName: Paraonidae; kingdom: Animalia; phylum: Annelida; class: Polychaeta; family: Paraonidae; taxonRank: family; scientificNameAuthorship: Cerruti, 1909; **Location:** waterBody: Pacific; stateProvince: Clarion Clipperton Zone; locality: UK Seabed Resources Ltd exploration area UK-1 Stratum A; verbatimLocality: UK1 Stratum A; maximumDepthInMeters: 4075; locationRemarks: Deployment RV05; at Station G; from R/V Melville Cruise no. MV1313; decimalLatitude: 13.76085; decimalLongitude: -116.4653; geodeticDatum: WGS84; **Identification:** identifiedBy: Helena Wiklund | Lenka Neal | Thomas Dahlgren | Adrian Glover | Madeleine Brasier | Regan Drennan | Eva Stewart; dateIdentified: 2021-04-20; identificationRemarks: identified by DNA and morphology; **Event:** eventID: UK1_AB01_RV05; samplingProtocol: Remotely Operated Vehicle; eventDate: 2013-10-17; eventTime: 19:06; habitat: Abyssal plain; **Record Level:** language: en; institutionCode: NHMUK; collectionCode: ZOO; datasetName: ABYSSLINE; basisOfRecord: PreservedSpecimen

##### Distribution

Eastern Clarion-Clipperton Zone, central Pacific Ocean.

##### Diagnosis

**Figure 69.**
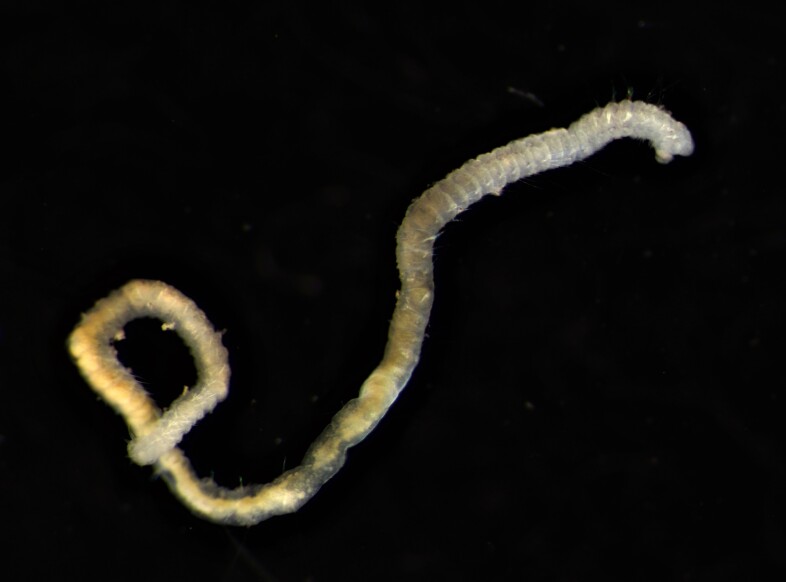
Paraonidae sp. (NHM_332), complete live specimen NHM_419 in dorsolateral view.

Complete (Fig. 69) and damaged specimens consistent with placement within family Paraonidae, based on morphology and DNA.

#### Paraonidae sp. (NHM_412)

4EB6AA8D-7CF7-562E-9A9F-1C13BCF0F928

##### Materials

- **Type status:** Other material. **Occurrence:** catalogNumber: NHMUK ANEA 2023.465; recordNumber: NHM_0412; recordedBy: Adrian Glover | Helena Wiklund | Thomas Dahlgren | Magdalena Georgieva; individualCount: 1; preparations: specimen stored in 80% non-denatured ethanol aqueous solution | DNA voucher stored in buffer; otherCatalogNumbers: 0174127383; associatedSequences: OQ746511 (16S) | OQ746827 (18S) | OQ738519 (COI); occurrenceID: 4ADADB89-2540-50D3-BDB7-6B4F97E335C4; **Taxon:** taxonConceptID: Paraonidae sp. (NHM_412); scientificName: Paraonidae; kingdom: Animalia; phylum: Annelida; class: Polychaeta; family: Paraonidae; taxonRank: family; scientificNameAuthorship: Cerruti, 1909; **Location:** waterBody: Pacific; stateProvince: Clarion Clipperton Zone; locality: UK Seabed Resources Ltd exploration area UK-1 Stratum A; verbatimLocality: UK1 Stratum A; maximumDepthInMeters: 4150; locationRemarks: Deployment MC09; at Station H; from R/V Melville Cruise no. MV1313; verbatimLatitude: 13°53.300; verbatimLongitude: 116°41.399; decimalLatitude: 13.88833; decimalLongitude: -116.68998; geodeticDatum: WGS84; **Identification:** identifiedBy: Helena Wiklund | Lenka Neal | Thomas Dahlgren | Adrian Glover | Madeleine Brasier | Regan Drennan | Eva Stewart; dateIdentified: 2021-04-20; identificationRemarks: identified by DNA and morphology; **Event:** eventID: UK1_AB01_MC09; samplingProtocol: Multi Corer; eventDate: 2013-10-19; eventTime: 07:14; habitat: Abyssal plain; **Record Level:** language: en; institutionCode: NHMUK; collectionCode: ZOO; datasetName: ABYSSLINE; basisOfRecord: PreservedSpecimen
- **Type status:** Other material. **Occurrence:** catalogNumber: NHMUK ANEA 2023.467; recordNumber: NHM_0916; recordedBy: Adrian Glover | Helena Wiklund | Thomas Dahlgren | Madeleine Brasier; individualCount: 1; preparations: specimen stored in 80% non-denatured ethanol aqueous solution | DNA voucher stored in buffer; otherCatalogNumbers: 0174126618; associatedSequences: OQ746584 (16S); occurrenceID: 628C02F5-783E-59B3-9E45-3CE1BD31910F; **Taxon:** taxonConceptID: Paraonidae sp. (NHM_412); scientificName: Paraonidae; kingdom: Animalia; phylum: Annelida; class: Polychaeta; family: Paraonidae; taxonRank: family; scientificNameAuthorship: Cerruti, 1909; **Location:** waterBody: Pacific; stateProvince: Clarion Clipperton Zone; locality: UK Seabed Resources Ltd exploration area UK-1 Stratum B; verbatimLocality: UK1 Stratum B; maximumDepthInMeters: 4198; locationRemarks: Deployment EB03; at Station U4; from R/V Thomas G. Thompson Cruise no. TN319; verbatimLatitude: 12'34.28; verbatimLongitude: 116'36.63; decimalLatitude: 12.57133; decimalLongitude: -116.6105; geodeticDatum: WGS84; **Identification:** identifiedBy: Helena Wiklund | Lenka Neal | Thomas Dahlgren | Adrian Glover | Madeleine Brasier | Regan Drennan | Eva Stewart; dateIdentified: 2021-04-20; identificationRemarks: identified by DNA and morphology; **Event:** eventID: UK1_AB02_EB03; samplingProtocol: Brenke Epibenthic Sledge; eventDate: 2015-02-23; eventTime: 05:39; habitat: Abyssal plain; fieldNotes: Collected from epi net (on the epibenthic sledge); **Record Level:** language: en; institutionCode: NHMUK; collectionCode: ZOO; datasetName: ABYSSLINE; basisOfRecord: PreservedSpecimen
- **Type status:** Other material. **Occurrence:** catalogNumber: NHMUK ANEA 2023.466; recordNumber: NHM_0418; recordedBy: Adrian Glover | Helena Wiklund | Thomas Dahlgren | Magdalena Georgieva; individualCount: 1; preparations: specimen stored in 80% non-denatured ethanol aqueous solution | DNA voucher stored in buffer; otherCatalogNumbers: 0174127376; associatedSequences: OQ746512 (16S) | OQ746828 (18S) | OQ738520 (COI); occurrenceID: CAFE01E6-36CA-57FF-B8BB-4BA2D4E55FF5; **Taxon:** taxonConceptID: Paraonidae sp. (NHM_412); scientificName: Paraonidae; kingdom: Animalia; phylum: Annelida; class: Polychaeta; family: Paraonidae; taxonRank: family; scientificNameAuthorship: Cerruti, 1909; **Location:** waterBody: Pacific; stateProvince: Clarion Clipperton Zone; locality: UK Seabed Resources Ltd exploration area UK-1 Stratum A; verbatimLocality: UK1 Stratum A; maximumDepthInMeters: 4011; locationRemarks: Deployment RV06; at Station K; from R/V Melville Cruise no. MV1313; decimalLatitude: 13.86367; decimalLongitude: -116.54432; geodeticDatum: WGS84; **Identification:** identifiedBy: Helena Wiklund | Lenka Neal | Thomas Dahlgren | Adrian Glover | Madeleine Brasier | Regan Drennan | Eva Stewart; dateIdentified: 2021-04-20; identificationRemarks: identified by DNA and morphology; **Event:** eventID: UK1_AB01_RV06; samplingProtocol: Remotely Operated Vehicle; eventDate: 2013-10-20; eventTime: 10:32; habitat: Abyssal plain; **Record Level:** language: en; institutionCode: NHMUK; collectionCode: ZOO; datasetName: ABYSSLINE; basisOfRecord: PreservedSpecimen

##### Distribution

Eastern Clarion-Clipperton Zone, central Pacific Ocean.

##### Diagnosis

**Figure 70.**
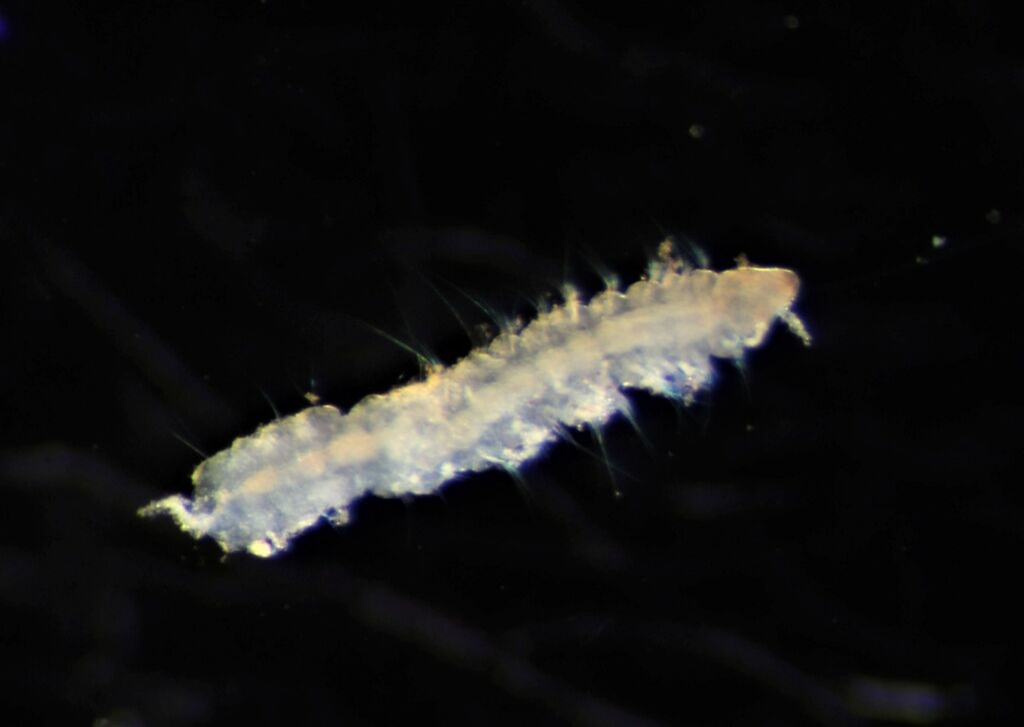
Paraonidae sp. (NHM_418), anterior fragment of live specimen NHM_418 in ventral view.

Complete (Fig. 70) and damaged specimens consistent with placement within family Paraonidae, based on morphology and DNA.

#### Paraonidae sp. (NHM_434)

9F6DF6D9-58D0-5DBE-BF17-9E13B4B4CBA7

##### Materials

- **Type status:** Other material. **Occurrence:** catalogNumber: NHMUK ANEA 2023.469; recordNumber: NHM_0996; recordedBy: Adrian Glover | Helena Wiklund | Thomas Dahlgren | Madeleine Brasier; individualCount: 1; preparations: specimen stored in 80% non-denatured ethanol aqueous solution | DNA voucher stored in buffer; otherCatalogNumbers: 0174126813; associatedSequences: OQ746600 (16S) | OQ738552 (COI); occurrenceID: 74139444-3A6F-5637-A2EA-4DB021FE6946; **Taxon:** taxonConceptID: Paraonidae sp. (NHM_434); scientificName: Paraonidae; kingdom: Animalia; phylum: Annelida; class: Polychaeta; family: Paraonidae; taxonRank: family; scientificNameAuthorship: Cerruti, 1909; **Location:** waterBody: Pacific; stateProvince: Clarion Clipperton Zone; locality: Ocean Mineral Singapore exploration claim Stratum A; verbatimLocality: OMS Stratum A; maximumDepthInMeters: 4122; locationRemarks: Deployment EB04; at Station S1; from R/V Thomas G. Thompson Cruise no. TN319; verbatimLatitude: 12'08.02; verbatimLongitude: 117'17.52; decimalLatitude: 12.13367; decimalLongitude: -117.292; geodeticDatum: WGS84; **Identification:** identifiedBy: Helena Wiklund | Lenka Neal | Thomas Dahlgren | Adrian Glover | Madeleine Brasier | Regan Drennan | Eva Stewart; dateIdentified: 2021-04-20; identificationRemarks: identified by DNA and morphology; **Event:** eventID: OMS1_AB02_EB04; samplingProtocol: Brenke Epibenthic Sledge; eventDate: 2015-02-24; eventTime: 19:10; habitat: Abyssal plain; fieldNotes: Collected from epi net (on the epibenthic sledge); **Record Level:** language: en; institutionCode: NHMUK; collectionCode: ZOO; datasetName: ABYSSLINE; basisOfRecord: PreservedSpecimen
- **Type status:** Other material. **Occurrence:** recordNumber: NHM_1948E; recordedBy: Adrian Glover | Helena Wiklund | Thomas Dahlgren | Madeleine Brasier; individualCount: 1; preparations: Tissue voucher stored in 80% non-denatured ethanol aqueous solution | DNA voucher stored in buffer; otherCatalogNumbers: 0109405427 | 0174126233; associatedSequences: OQ746741 (16S); occurrenceID: C0B79091-1010-59E8-AB8E-690834D55927; **Taxon:** taxonConceptID: Paraonidae sp. (NHM_434); scientificName: Paraonidae; kingdom: Animalia; phylum: Annelida; class: Polychaeta; family: Paraonidae; taxonRank: family; scientificNameAuthorship: Cerruti, 1909; **Location:** waterBody: Pacific; stateProvince: Clarion Clipperton Zone; locality: Ocean Mineral Singapore exploration claim Stratum A; verbatimLocality: OMS Stratum A; maximumDepthInMeters: 4094; locationRemarks: Deployment EB11; at Station S10; from R/V Thomas G. Thompson Cruise no. TN319; verbatimLatitude: 12°02.49’; verbatimLongitude: 117°13.03’; decimalLatitude: 12.0415; decimalLongitude: -117.21717; geodeticDatum: WGS84; **Identification:** identifiedBy: Helena Wiklund | Lenka Neal | Thomas Dahlgren | Adrian Glover | Madeleine Brasier | Regan Drennan | Eva Stewart; dateIdentified: 2021-04-20; identificationRemarks: identified by DNA and morphology; **Event:** eventID: OMS1_AB02_EB11; samplingProtocol: Brenke Epibenthic Sledge; eventDate: 2015-03-13; habitat: Abyssal plain; fieldNotes: Collected from epi net (on the epibenthic sledge); **Record Level:** language: en; institutionCode: NHMUK; collectionCode: ZOO; datasetName: ABYSSLINE; basisOfRecord: PreservedSpecimen
- **Type status:** Other material. **Occurrence:** catalogNumber: NHMUK ANEA 2023.468; recordNumber: NHM_0434; recordedBy: Adrian Glover | Helena Wiklund | Thomas Dahlgren | Magdalena Georgieva; individualCount: 1; preparations: specimen stored in 80% non-denatured ethanol aqueous solution | DNA voucher stored in buffer; otherCatalogNumbers: 0174127340; associatedSequences: OQ746518 (16S) | OQ746834 (18S); occurrenceID: 7860C216-EE0B-5A00-92A8-2E40A3C9A714; **Taxon:** taxonConceptID: Paraonidae sp. (NHM_434); scientificName: Paraonidae; kingdom: Animalia; phylum: Annelida; class: Polychaeta; family: Paraonidae; taxonRank: family; scientificNameAuthorship: Cerruti, 1909; **Location:** waterBody: Pacific; stateProvince: Clarion Clipperton Zone; locality: UK Seabed Resources Ltd exploration area UK-1 Stratum A; verbatimLocality: UK1 Stratum A; maximumDepthInMeters: 4011; locationRemarks: Deployment RV06; at Station K; from R/V Melville Cruise no. MV1313; decimalLatitude: 13.86367; decimalLongitude: -116.54432; geodeticDatum: WGS84; **Identification:** identifiedBy: Helena Wiklund | Lenka Neal | Thomas Dahlgren | Adrian Glover | Madeleine Brasier | Regan Drennan | Eva Stewart; dateIdentified: 2021-04-20; identificationRemarks: identified by DNA and morphology; **Event:** eventID: UK1_AB01_RV06; samplingProtocol: Remotely Operated Vehicle; eventDate: 2013-10-20; eventTime: 10:32; habitat: Abyssal plain; **Record Level:** language: en; institutionCode: NHMUK; collectionCode: ZOO; datasetName: ABYSSLINE; basisOfRecord: PreservedSpecimen

##### Distribution

Eastern Clarion-Clipperton Zone, central Pacific Ocean.

##### Diagnosis

**Figure 71.**
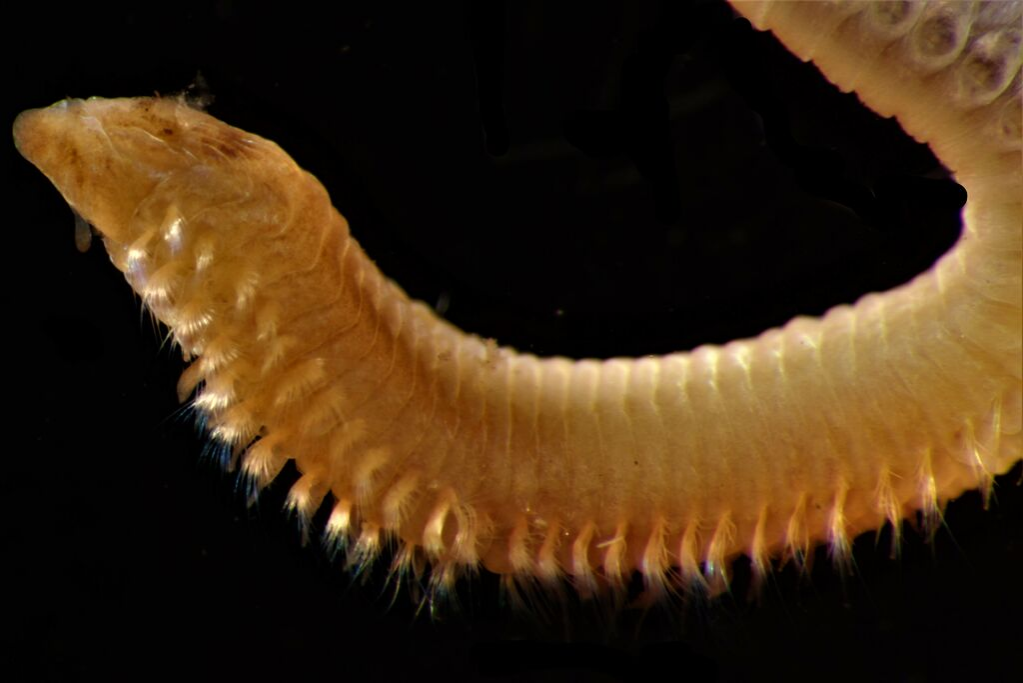
Paraonidae sp. (NHM_434), anterior fragment of live specimen NHM_996 in ventrolateral view.

Damaged specimens (Fig. 71) consistent with placement within family Paraonidae, based on morphology and DNA.

#### Paraonidae sp. (NHM_584)

CD254566-6A63-56D5-8167-93168B39DDF2

##### Materials

- **Type status:** Other material. **Occurrence:** catalogNumber: NHMUK ANEA 2023.470; recordNumber: NHM_1251; recordedBy: Adrian Glover | Helena Wiklund | Thomas Dahlgren | Madeleine Brasier; individualCount: 1; preparations: specimen stored in 80% non-denatured ethanol aqueous solution | DNA voucher stored in buffer; otherCatalogNumbers: 0174126542; associatedSequences: OQ746646 (16S); occurrenceID: CB175DF4-8DF2-5D5A-BA9B-CAB1D70A98A7; **Taxon:** taxonConceptID: Paraonidae sp. (NHM_584); scientificName: Paraonidae; kingdom: Animalia; phylum: Annelida; class: Polychaeta; family: Paraonidae; taxonRank: family; scientificNameAuthorship: Cerruti, 1909; **Location:** waterBody: Pacific; stateProvince: Clarion Clipperton Zone; locality: Ocean Mineral Singapore exploration claim Stratum A; verbatimLocality: OMS Stratum A; maximumDepthInMeters: 4302; locationRemarks: Deployment EB06; at Station S5; from R/V Thomas G. Thompson Cruise no. TN319; verbatimLatitude: 12'15.44; verbatimLongitude: 117'18.13; decimalLatitude: 12.25733; decimalLongitude: -117.30217; geodeticDatum: WGS84; **Identification:** identifiedBy: Helena Wiklund | Lenka Neal | Thomas Dahlgren | Adrian Glover | Madeleine Brasier | Regan Drennan | Eva Stewart; dateIdentified: 2021-04-20; identificationRemarks: identified by DNA and morphology; **Event:** eventID: OMS1_AB02_EB06; samplingProtocol: Brenke Epibenthic Sledge; eventDate: 2015-03-01; eventTime: 04:02; habitat: Abyssal plain; fieldNotes: Collected from epi net (on the epibenthic sledge); **Record Level:** language: en; institutionCode: NHMUK; collectionCode: ZOO; datasetName: ABYSSLINE; basisOfRecord: PreservedSpecimen
- **Type status:** Other material. **Occurrence:** recordNumber: NHM_0584; recordedBy: Adrian Glover | Helena Wiklund | Thomas Dahlgren | Madeleine Brasier; individualCount: 1; preparations: Tissue voucher stored in 80% non-denatured ethanol aqueous solution | DNA voucher stored in buffer; otherCatalogNumbers: 0109405407 | 0174126599; associatedSequences: OQ746530 (16S) | OQ746844 (18S); occurrenceID: 9F43A08C-6447-5E02-9671-2B818E44F083; **Taxon:** taxonConceptID: Paraonidae sp. (NHM_584); scientificName: Paraonidae; kingdom: Animalia; phylum: Annelida; class: Polychaeta; family: Paraonidae; taxonRank: family; scientificNameAuthorship: Cerruti, 1909; **Location:** waterBody: Pacific; stateProvince: Clarion Clipperton Zone; locality: UK Seabed Resources Ltd exploration area UK-1 Stratum B; verbatimLocality: UK1 Stratum B; maximumDepthInMeters: 4202; locationRemarks: Deployment EB01; at Station U2; from R/V Thomas G. Thompson Cruise no. TN319; verbatimLatitude: 12'23.17456; verbatimLongitude: 116'32.92021; decimalLatitude: 12.38624; decimalLongitude: -116.54867; geodeticDatum: WGS84; **Identification:** identifiedBy: Helena Wiklund | Lenka Neal | Thomas Dahlgren | Adrian Glover | Madeleine Brasier | Regan Drennan | Eva Stewart; dateIdentified: 2021-04-20; identificationRemarks: identified by DNA and morphology; **Event:** eventID: UK1_AB02_EB01; samplingProtocol: Brenke Epibenthic Sledge; eventDate: 2015-02-17; eventTime: 05:15; habitat: Abyssal plain; fieldNotes: Collected from epi net (on the epibenthic sledge); **Record Level:** language: en; institutionCode: NHMUK; collectionCode: ZOO; datasetName: ABYSSLINE; basisOfRecord: PreservedSpecimen

##### Distribution

Eastern Clarion-Clipperton Zone, central Pacific Ocean.

##### Diagnosis

**Figure 72.**
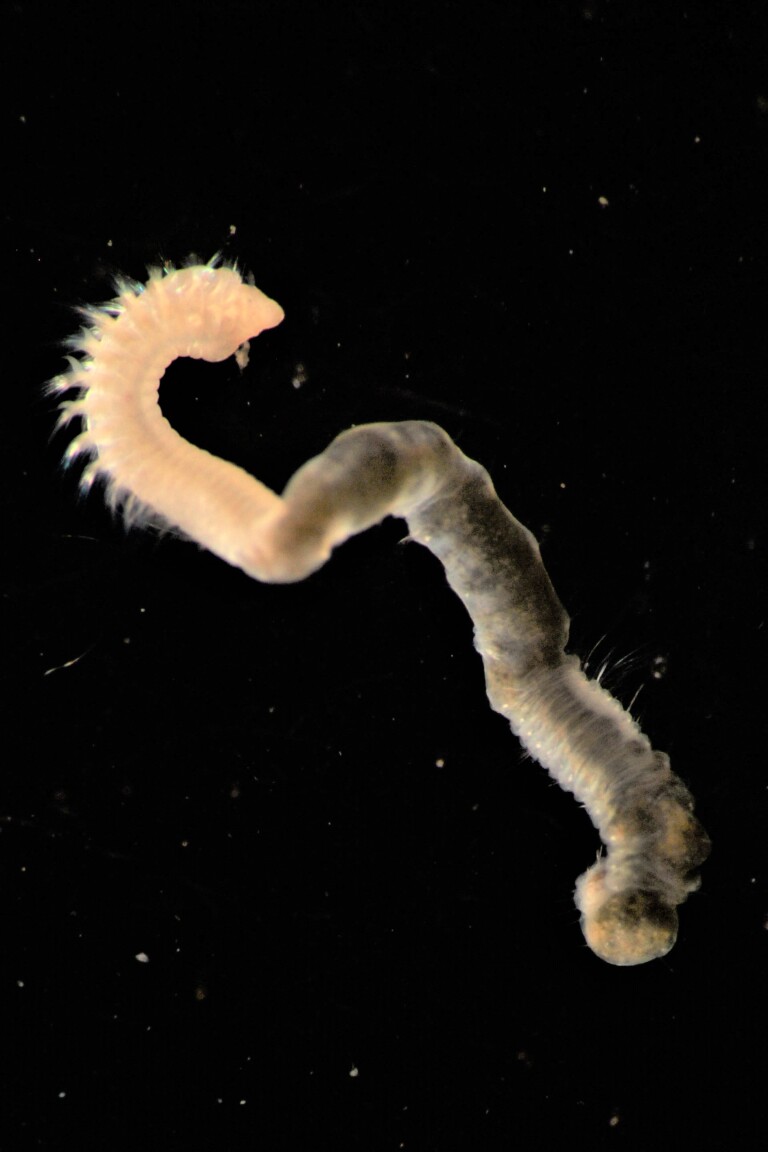
Paraonidae sp. (NHM_584), anterior fragment of a live specimen NHM_1251.

Fragmented specimens (Fig. 72) consistent with placement within family Paraonidae, based on morphology and DNA.

#### Paraonidae sp. (NHM_902)

878B1D43-37E4-5AA8-A05D-EAB0FD975EA7

##### Materials

- **Type status:** Other material. **Occurrence:** catalogNumber: NHMUK ANEA 2023.474; recordNumber: NHM_1841; recordedBy: Adrian Glover | Helena Wiklund | Thomas Dahlgren | Madeleine Brasier; individualCount: 1; preparations: specimen stored in 80% non-denatured ethanol aqueous solution | DNA voucher stored in buffer; otherCatalogNumbers: 0174126188; associatedSequences: OQ746724 (16S); occurrenceID: C318D5C9-31E2-5D88-B357-B63029398E7E; **Taxon:** taxonConceptID: Paraonidae sp. (NHM_902); scientificName: Paraonidae; kingdom: Animalia; phylum: Annelida; class: Polychaeta; family: Paraonidae; taxonRank: family; scientificNameAuthorship: Cerruti, 1909; **Location:** waterBody: Pacific; stateProvince: Clarion Clipperton Zone; locality: Ocean Mineral Singapore exploration claim Stratum A; verbatimLocality: OMS Stratum A; maximumDepthInMeters: 4051; locationRemarks: Deployment BC22; at Station S9; from R/V Thomas G. Thompson Cruise no. TN319; verbatimLatitude: 12'05.994; verbatimLongitude: 117'11.796; decimalLatitude: 12.0999; decimalLongitude: -117.1966; geodeticDatum: WGS84; **Identification:** identifiedBy: Helena Wiklund | Lenka Neal | Thomas Dahlgren | Adrian Glover | Madeleine Brasier | Regan Drennan | Eva Stewart; dateIdentified: 2021-04-20; identificationRemarks: identified by DNA and morphology; **Event:** eventID: OMS1_AB02_BC22; samplingProtocol: USNEL Box Core; eventDate: 2015-03-12; eventTime: 19:03; habitat: Abyssal plain; fieldNotes: Collected from 0-2 cm layer of box core using a 300 micron sieve; **Record Level:** language: en; institutionCode: NHMUK; collectionCode: ZOO; datasetName: ABYSSLINE; basisOfRecord: PreservedSpecimen
- **Type status:** Other material. **Occurrence:** catalogNumber: NHMUK ANEA 2023.473; recordNumber: NHM_1774; recordedBy: Adrian Glover | Helena Wiklund | Thomas Dahlgren | Madeleine Brasier; individualCount: 1; preparations: specimen stored in 80% non-denatured ethanol aqueous solution | DNA voucher stored in buffer; otherCatalogNumbers: 0174126195; associatedSequences: OQ746717 (16S); occurrenceID: 1B73CEE7-0CEB-58D1-B204-5997521AE97E; **Taxon:** taxonConceptID: Paraonidae sp. (NHM_902); scientificName: Paraonidae; kingdom: Animalia; phylum: Annelida; class: Polychaeta; family: Paraonidae; taxonRank: family; scientificNameAuthorship: Cerruti, 1909; **Location:** waterBody: Pacific; stateProvince: Clarion Clipperton Zone; locality: Ocean Mineral Singapore exploration claim Stratum A; verbatimLocality: OMS Stratum A; maximumDepthInMeters: 4045; locationRemarks: Deployment EB10; at Station S7; from R/V Thomas G. Thompson Cruise no. TN319; verbatimLatitude: 12'10.43; verbatimLongitude: 117'11.57; decimalLatitude: 12.17383; decimalLongitude: -117.19283; geodeticDatum: WGS84; **Identification:** identifiedBy: Helena Wiklund | Lenka Neal | Thomas Dahlgren | Adrian Glover | Madeleine Brasier | Regan Drennan | Eva Stewart; dateIdentified: 2021-04-20; identificationRemarks: identified by DNA and morphology; **Event:** eventID: OMS1_AB02_EB10; samplingProtocol: Brenke Epibenthic Sledge; eventDate: 2015-03-11; eventTime: 22:49; habitat: Abyssal plain; fieldNotes: Collected from epi net (on the epibenthic sledge); **Record Level:** language: en; institutionCode: NHMUK; collectionCode: ZOO; datasetName: ABYSSLINE; basisOfRecord: PreservedSpecimen
- **Type status:** Other material. **Occurrence:** catalogNumber: NHMUK ANEA 2023.472; recordNumber: NHM_1452; recordedBy: Adrian Glover | Helena Wiklund | Thomas Dahlgren | Madeleine Brasier; individualCount: 1; preparations: specimen stored in 80% non-denatured ethanol aqueous solution | DNA voucher stored in buffer; otherCatalogNumbers: 0174126771; associatedSequences: OQ746681 (16S) | OQ738585 (COI); occurrenceID: 809B6B20-59C8-5F3C-AA11-14CB2D37F674; **Taxon:** taxonConceptID: Paraonidae sp. (NHM_902); scientificName: Paraonidae; kingdom: Animalia; phylum: Annelida; class: Polychaeta; family: Paraonidae; taxonRank: family; scientificNameAuthorship: Cerruti, 1909; **Location:** waterBody: Pacific; stateProvince: Clarion Clipperton Zone; locality: UK Seabed Resources Ltd exploration area UK-1 Stratum B; verbatimLocality: UK1 Stratum B; maximumDepthInMeters: 4137; locationRemarks: Deployment EB07; at Station U7; from R/V Thomas G. Thompson Cruise no. TN319; verbatimLatitude: 12'27.26; verbatimLongitude: 116'36.77; decimalLatitude: 12.45433; decimalLongitude: -116.61283; geodeticDatum: WGS84; **Identification:** identifiedBy: Helena Wiklund | Lenka Neal | Thomas Dahlgren | Adrian Glover | Madeleine Brasier | Regan Drennan | Eva Stewart; dateIdentified: 2021-04-20; identificationRemarks: identified by DNA and morphology; **Event:** eventID: UK1_AB02_EB07; samplingProtocol: Brenke Epibenthic Sledge; eventDate: 2015-03-03; eventTime: 20:40; habitat: Abyssal plain; fieldNotes: Collected from epi net (on the epibenthic sledge); **Record Level:** language: en; institutionCode: NHMUK; collectionCode: ZOO; datasetName: ABYSSLINE; basisOfRecord: PreservedSpecimen
- **Type status:** Other material. **Occurrence:** catalogNumber: NHMUK ANEA 2023.471; recordNumber: NHM_0902; recordedBy: Adrian Glover | Helena Wiklund | Thomas Dahlgren | Madeleine Brasier; individualCount: 1; preparations: specimen stored in 80% non-denatured ethanol aqueous solution | DNA voucher stored in buffer; otherCatalogNumbers: 0174126798; associatedSequences: OQ746573 (16S); occurrenceID: 16DA3996-B03B-5743-AFFB-0676CA7CC718; **Taxon:** taxonConceptID: Paraonidae sp. (NHM_902); scientificName: Paraonidae; kingdom: Animalia; phylum: Annelida; class: Polychaeta; family: Paraonidae; taxonRank: family; scientificNameAuthorship: Cerruti, 1909; **Location:** waterBody: Pacific; stateProvince: Clarion Clipperton Zone; locality: UK Seabed Resources Ltd exploration area UK-1 Stratum B; verbatimLocality: UK1 Stratum B; maximumDepthInMeters: 4198; locationRemarks: Deployment EB03; at Station U4; from R/V Thomas G. Thompson Cruise no. TN319; verbatimLatitude: 12'34.28; verbatimLongitude: 116'36.63; decimalLatitude: 12.57133; decimalLongitude: -116.6105; geodeticDatum: WGS84; **Identification:** identifiedBy: Helena Wiklund | Lenka Neal | Thomas Dahlgren | Adrian Glover | Madeleine Brasier | Regan Drennan | Eva Stewart; dateIdentified: 2021-04-20; identificationRemarks: identified by DNA and morphology; **Event:** eventID: UK1_AB02_EB03; samplingProtocol: Brenke Epibenthic Sledge; eventDate: 2015-02-23; eventTime: 05:39; habitat: Abyssal plain; fieldNotes: Collected from epi net (on the epibenthic sledge); **Record Level:** language: en; institutionCode: NHMUK; collectionCode: ZOO; datasetName: ABYSSLINE; basisOfRecord: PreservedSpecimen

##### Distribution

Eastern Clarion-Clipperton Zone, central Pacific Ocean.

##### Diagnosis

Damaged specimens (Fig. 73) consistent with the placement within family Paraonidae, based on morphology and DNA.

#### Paraonidae sp. (NHM_1139)

3C965435-1C3E-5F8C-99B4-897B6174D6F2

##### Materials

- **Type status:** Other material. **Occurrence:** catalogNumber: NHMUK ANEA 2023.452; recordNumber: NHM_1139; recordedBy: Adrian Glover | Helena Wiklund | Thomas Dahlgren | Madeleine Brasier; individualCount: 1; preparations: specimen stored in 80% non-denatured ethanol aqueous solution | DNA voucher stored in buffer; otherCatalogNumbers: 0174126559; associatedSequences: OQ746622 (16S); occurrenceID: 7F2AB3D7-68B2-5064-9E93-98FAF8902755; **Taxon:** taxonConceptID: Paraonidae sp. (NHM_1139); scientificName: Paraonidae; kingdom: Animalia; phylum: Annelida; class: Polychaeta; family: Paraonidae; taxonRank: family; scientificNameAuthorship: Cerruti, 1909; **Location:** waterBody: Pacific; stateProvince: Clarion Clipperton Zone; locality: Ocean Mineral Singapore exploration claim Stratum A; verbatimLocality: OMS Stratum A; maximumDepthInMeters: 4100; locationRemarks: Deployment EB05; at Station S2; from R/V Thomas G. Thompson Cruise no. TN319; verbatimLatitude: 12'06.93; verbatimLongitude: 117'09.87; decimalLatitude: 12.1155; decimalLongitude: -117.1645; geodeticDatum: WGS84; **Identification:** identifiedBy: Helena Wiklund | Lenka Neal | Thomas Dahlgren | Adrian Glover | Madeleine Brasier | Regan Drennan | Eva Stewart; dateIdentified: 2021-04-20; identificationRemarks: identified by DNA and morphology; **Event:** eventID: OMS1_AB02_EB05; samplingProtocol: Brenke Epibenthic Sledge; eventDate: 2015-02-26; eventTime: 21:29; habitat: Abyssal plain; fieldNotes: Collected from epi net (on the epibenthic sledge); **Record Level:** language: en; institutionCode: NHMUK; collectionCode: ZOO; datasetName: ABYSSLINE; basisOfRecord: PreservedSpecimen

##### Distribution

Eastern Clarion-Clipperton Zone, central Pacific Ocean.

##### Diagnosis

**Figure 74.**
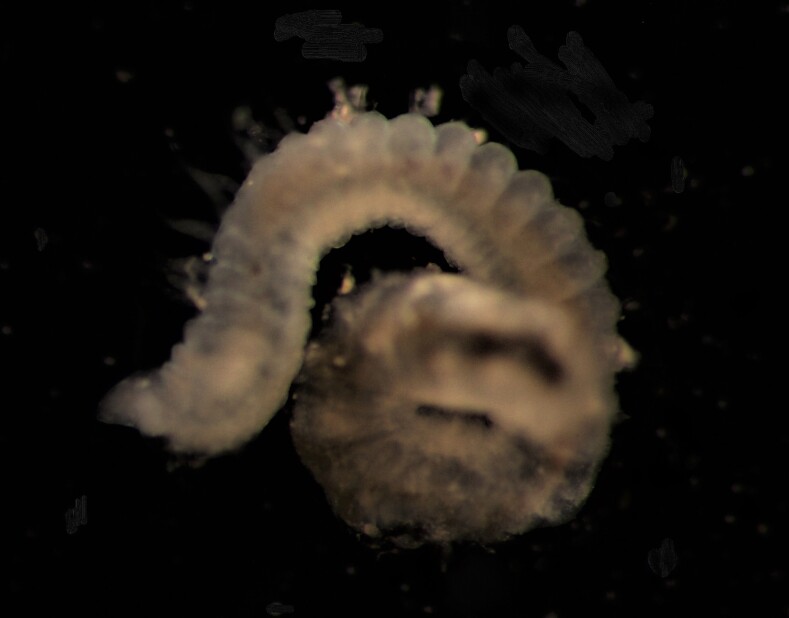
Paraonidae sp. (NHM_1139), anterior fragment of live specimen NHM_1139 in lateral view.

Damaged specimen (Fig. 74) consistent with placement within family Paraonidae, based on morphology and DNA.

#### Paraonidae sp. (NHM_2118)

793A34E9-C3A3-549C-B17A-3BB5EF62DCBF

##### Materials

- **Type status:** Other material. **Occurrence:** catalogNumber: NHMUK ANEA 2023.454; recordNumber: NHM_2118; recordedBy: Adrian Glover | Helena Wiklund | Thomas Dahlgren | Madeleine Brasier; individualCount: 1; preparations: specimen stored in 80% non-denatured ethanol aqueous solution | DNA voucher stored in buffer; otherCatalogNumbers: 0174126264; associatedSequences: OQ746756 (16S); occurrenceID: A0B504F1-745C-5067-9C5F-D9C2626DB57F; **Taxon:** taxonConceptID: Paraonidae sp. (NHM_2118); scientificName: Paraonidae; kingdom: Animalia; phylum: Annelida; class: Polychaeta; family: Paraonidae; taxonRank: family; scientificNameAuthorship: Cerruti, 1909; **Location:** waterBody: Pacific; stateProvince: Clarion Clipperton Zone; locality: Area of Particular Interest APEI-6; verbatimLocality: APEI-6; maximumDepthInMeters: 4026; locationRemarks: Deployment EB13; at Station APEI; from R/V Thomas G. Thompson Cruise no. TN319; verbatimLatitude: 19 27.874; verbatimLongitude: 120 01.525; decimalLatitude: 19.46457; decimalLongitude: -120.02542; geodeticDatum: WGS84; **Identification:** identifiedBy: Helena Wiklund | Lenka Neal | Thomas Dahlgren | Adrian Glover | Madeleine Brasier | Regan Drennan | Eva Stewart; dateIdentified: 2021-04-20; identificationRemarks: identified by DNA and morphology; **Event:** eventID: APEI6_AB02_EB13; samplingProtocol: Brenke Epibenthic Sledge; eventDate: 2015-03-20; eventTime: 16:12; habitat: Abyssal plain; fieldNotes: Collected from epi net (on the epibenthic sledge); **Record Level:** language: en; institutionCode: NHMUK; collectionCode: ZOO; datasetName: ABYSSLINE; basisOfRecord: PreservedSpecimen

##### Distribution

Eastern Clarion-Clipperton Zone, central Pacific Ocean.

##### Diagnosis

**Figure 75.**
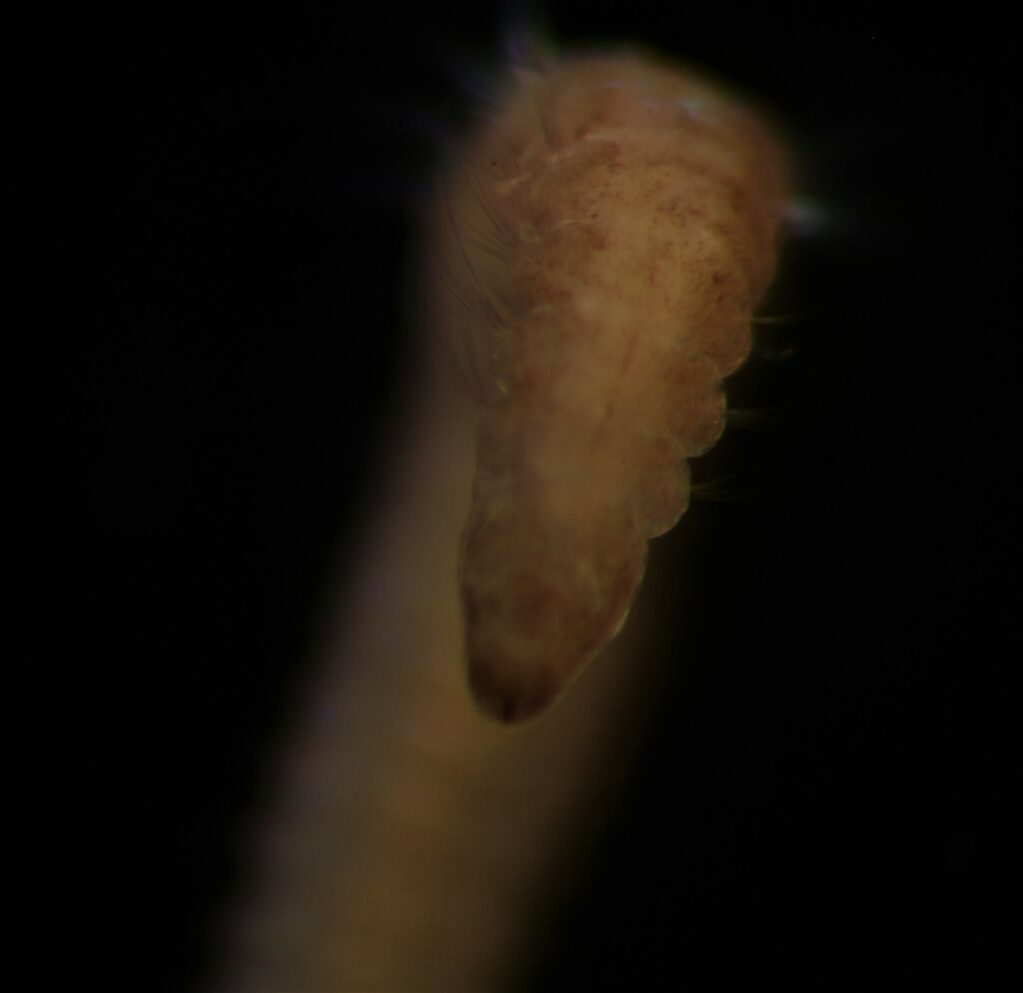
Paraonidae sp. (NHM_2118), anterior fragment of live specimen NHM_2118 in dorsal view.

Damaged specimen (Fig. 75) consistent with placement within family Paraonidae, based on morphology and DNA.

#### Paraonidae sp. (NHM_363)

E8C350E7-C35D-540F-AD7F-1C4F8479441B

##### Materials

- **Type status:** Other material. **Occurrence:** catalogNumber: NHMUK ANEA 2023.463; recordNumber: NHM_1334; recordedBy: Adrian Glover | Helena Wiklund | Thomas Dahlgren | Madeleine Brasier; individualCount: 1; preparations: specimen stored in 80% non-denatured ethanol aqueous solution | DNA voucher stored in buffer; otherCatalogNumbers: 0174126334; associatedSequences: OQ746660 (16S); occurrenceID: 71D7C1A7-5A76-5526-BD41-DDFBDFC31069; **Taxon:** taxonConceptID: Paraonidae sp. (NHM_363); scientificName: Paraonidae; kingdom: Animalia; phylum: Annelida; class: Polychaeta; family: Paraonidae; taxonRank: family; scientificNameAuthorship: Cerruti, 1909; **Location:** waterBody: Pacific; stateProvince: Clarion Clipperton Zone; locality: Ocean Mineral Singapore exploration claim Stratum A; verbatimLocality: OMS Stratum A; maximumDepthInMeters: 4302; locationRemarks: Deployment EB06; at Station S5; from R/V Thomas G. Thompson Cruise no. TN319; verbatimLatitude: 12'15.44; verbatimLongitude: 117'18.13; decimalLatitude: 12.25733; decimalLongitude: -117.30217; geodeticDatum: WGS84; **Identification:** identifiedBy: Helena Wiklund | Lenka Neal | Thomas Dahlgren | Adrian Glover | Madeleine Brasier | Regan Drennan | Eva Stewart; dateIdentified: 2021-04-20; identificationRemarks: identified by DNA and morphology; **Event:** eventID: OMS1_AB02_EB06; samplingProtocol: Brenke Epibenthic Sledge; eventDate: 2015-03-01; eventTime: 04:02; habitat: Abyssal plain; fieldNotes: Collected from epi net (on the epibenthic sledge); **Record Level:** language: en; institutionCode: NHMUK; collectionCode: ZOO; datasetName: ABYSSLINE; basisOfRecord: PreservedSpecimen
- **Type status:** Other material. **Occurrence:** catalogNumber: NHMUK ANEA 2023.462; recordNumber: NHM_1009; recordedBy: Adrian Glover | Helena Wiklund | Thomas Dahlgren | Madeleine Brasier; individualCount: 1; preparations: specimen stored in 80% non-denatured ethanol aqueous solution | DNA voucher stored in buffer; otherCatalogNumbers: 0174126263; associatedSequences: OQ746604 (16S) | OQ746871 (18S); occurrenceID: 5DF38A64-F1D2-5D86-A86C-46B20E1B6CD9; **Taxon:** taxonConceptID: Paraonidae sp. (NHM_363); scientificName: Paraonidae; kingdom: Animalia; phylum: Annelida; class: Polychaeta; family: Paraonidae; taxonRank: family; scientificNameAuthorship: Cerruti, 1909; **Location:** waterBody: Pacific; stateProvince: Clarion Clipperton Zone; locality: Ocean Mineral Singapore exploration claim Stratum A; verbatimLocality: OMS Stratum A; maximumDepthInMeters: 4122; locationRemarks: Deployment EB04; at Station S1; from R/V Thomas G. Thompson Cruise no. TN319; verbatimLatitude: 12'08.02; verbatimLongitude: 117'17.52; decimalLatitude: 12.13367; decimalLongitude: -117.292; geodeticDatum: WGS84; **Identification:** identifiedBy: Helena Wiklund | Lenka Neal | Thomas Dahlgren | Adrian Glover | Madeleine Brasier | Regan Drennan | Eva Stewart; dateIdentified: 2021-04-20; identificationRemarks: identified by DNA and morphology; **Event:** eventID: OMS1_AB02_EB04; samplingProtocol: Brenke Epibenthic Sledge; eventDate: 2015-02-24; eventTime: 19:10; habitat: Abyssal plain; fieldNotes: Collected from epi net (on the epibenthic sledge); **Record Level:** language: en; institutionCode: NHMUK; collectionCode: ZOO; datasetName: ABYSSLINE; basisOfRecord: PreservedSpecimen
- **Type status:** Other material. **Occurrence:** recordNumber: NHM_1020; recordedBy: Adrian Glover | Helena Wiklund | Thomas Dahlgren | Madeleine Brasier; individualCount: 1; preparations: DNA voucher stored in buffer; otherCatalogNumbers: 0174126240; associatedSequences: OQ746606 (16S); occurrenceID: 8FF33B88-8E5E-53E3-B9AC-73B78B433CB5; **Taxon:** taxonConceptID: Paraonidae sp. (NHM_363); scientificName: Paraonidae; kingdom: Animalia; phylum: Annelida; class: Polychaeta; family: Paraonidae; taxonRank: family; scientificNameAuthorship: Cerruti, 1909; **Location:** waterBody: Pacific; stateProvince: Clarion Clipperton Zone; locality: Ocean Mineral Singapore exploration claim Stratum A; verbatimLocality: OMS Stratum A; maximumDepthInMeters: 4122; locationRemarks: Deployment EB04; at Station S1; from R/V Thomas G. Thompson Cruise no. TN319; verbatimLatitude: 12'08.02; verbatimLongitude: 117'17.52; decimalLatitude: 12.13367; decimalLongitude: -117.292; geodeticDatum: WGS84; **Identification:** identifiedBy: Helena Wiklund | Lenka Neal | Thomas Dahlgren | Adrian Glover | Madeleine Brasier | Regan Drennan | Eva Stewart; dateIdentified: 2021-04-20; identificationRemarks: identified by DNA and morphology; **Event:** eventID: OMS1_AB02_EB04; samplingProtocol: Brenke Epibenthic Sledge; eventDate: 2015-02-24; eventTime: 19:10; habitat: Abyssal plain; fieldNotes: Collected from epi net (on the epibenthic sledge); **Record Level:** language: en; institutionCode: NHMUK; collectionCode: ZOO; datasetName: ABYSSLINE; basisOfRecord: PreservedSpecimen
- **Type status:** Other material. **Occurrence:** catalogNumber: NHMUK ANEA 2023.464; recordNumber: NHM_1932; recordedBy: Adrian Glover | Helena Wiklund | Thomas Dahlgren | Madeleine Brasier; individualCount: 1; preparations: specimen stored in 80% non-denatured ethanol aqueous solution | DNA voucher stored in buffer; otherCatalogNumbers: 0174126313; associatedSequences: OQ746733 (16S); occurrenceID: DB93644D-99E0-55C7-97E1-7959D3F0795F; **Taxon:** taxonConceptID: Paraonidae sp. (NHM_363); scientificName: Paraonidae; kingdom: Animalia; phylum: Annelida; class: Polychaeta; family: Paraonidae; taxonRank: family; scientificNameAuthorship: Cerruti, 1909; **Location:** waterBody: Pacific; stateProvince: Clarion Clipperton Zone; locality: Ocean Mineral Singapore exploration claim Stratum A; verbatimLocality: OMS Stratum A; maximumDepthInMeters: 4094; locationRemarks: Deployment EB11; at Station S10; from R/V Thomas G. Thompson Cruise no. TN319; verbatimLatitude: 12°02.49’; verbatimLongitude: 117°13.03’; decimalLatitude: 12.0415; decimalLongitude: -117.21717; geodeticDatum: WGS84; **Identification:** identifiedBy: Helena Wiklund | Lenka Neal | Thomas Dahlgren | Adrian Glover | Madeleine Brasier | Regan Drennan | Eva Stewart; dateIdentified: 2021-04-20; identificationRemarks: identified by DNA and morphology; **Event:** eventID: OMS1_AB02_EB11; samplingProtocol: Brenke Epibenthic Sledge; eventDate: 2015-03-13; habitat: Abyssal plain; fieldNotes: Collected from epi net (on the epibenthic sledge); **Record Level:** language: en; institutionCode: NHMUK; collectionCode: ZOO; datasetName: ABYSSLINE; basisOfRecord: PreservedSpecimen
- **Type status:** Other material. **Occurrence:** catalogNumber: NHMUK ANEA 2023.461; recordNumber: NHM_0363; recordedBy: Adrian Glover | Helena Wiklund | Thomas Dahlgren | Magdalena Georgieva; individualCount: 1; preparations: specimen stored in 80% non-denatured ethanol aqueous solution | DNA voucher stored in buffer; otherCatalogNumbers: 0174127336; associatedSequences: OQ746505 (16S) | OQ746822 (18S); occurrenceID: 4DF0A8F4-7BFC-52F8-8FAA-1EF3E4D90AD4; **Taxon:** taxonConceptID: Paraonidae sp. (NHM_363); scientificName: Paraonidae; kingdom: Animalia; phylum: Annelida; class: Polychaeta; family: Paraonidae; taxonRank: family; scientificNameAuthorship: Cerruti, 1909; **Location:** waterBody: Pacific; stateProvince: Clarion Clipperton Zone; locality: UK Seabed Resources Ltd exploration area UK-1 Stratum A; verbatimLocality: UK1 Stratum A; maximumDepthInMeters: 4182; locationRemarks: Deployment EB05; at Station H-J; from R/V Melville Cruise no. MV1313; verbatimLatitude: 13°55.984; verbatimLongitude: 116°42.977; decimalLatitude: 13.93307; decimalLongitude: -116.72378; geodeticDatum: WGS84; **Identification:** identifiedBy: Helena Wiklund | Lenka Neal | Thomas Dahlgren | Adrian Glover | Madeleine Brasier | Regan Drennan | Eva Stewart; dateIdentified: 2021-04-20; identificationRemarks: identified by DNA and morphology; **Event:** eventID: UK1_AB01_EB05; samplingProtocol: Brenke Epibenthic Sledge; eventDate: 2013-10-19; eventTime: 12:16; habitat: Abyssal plain; fieldNotes: Collected from epi net (on the epibenthic sledge); **Record Level:** language: en; institutionCode: NHMUK; collectionCode: ZOO; datasetName: ABYSSLINE; basisOfRecord: PreservedSpecimen

##### Distribution

Eastern Clarion-Clipperton Zone, central Pacific Ocean.

##### Diagnosis

**Figure 76.**
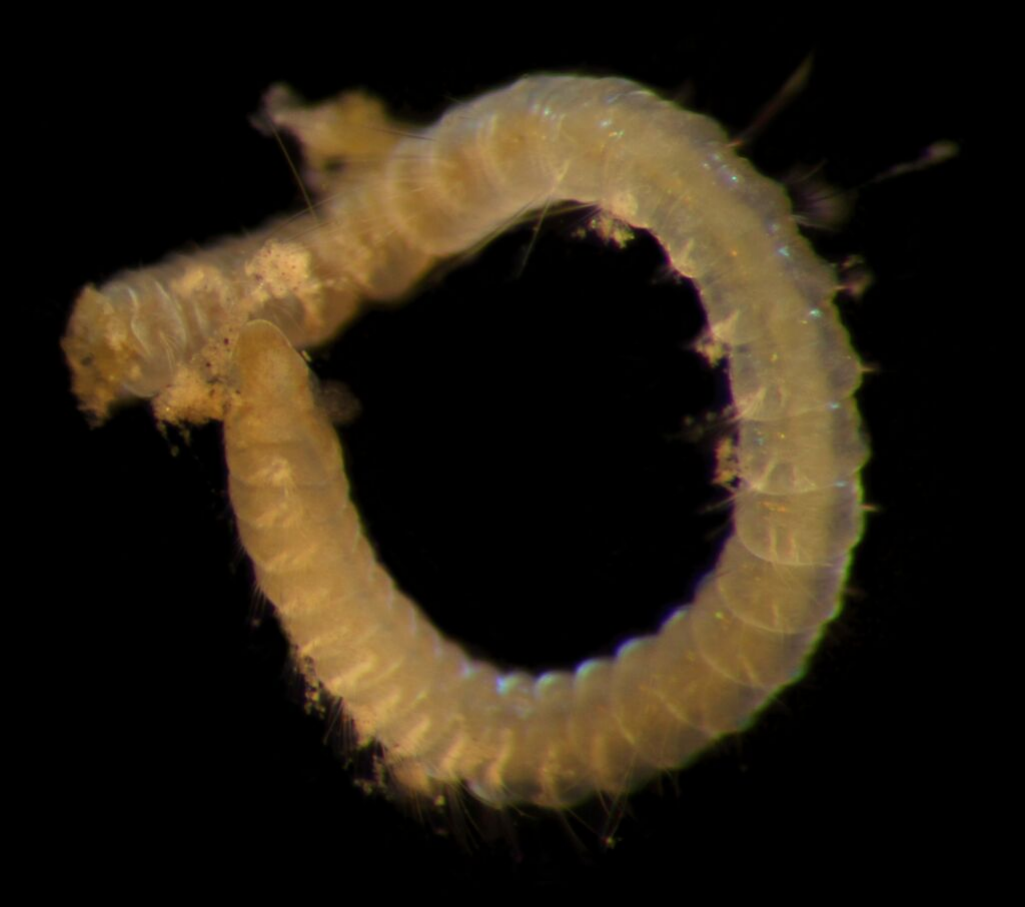
Paraonidae sp. (NHM_363) specimen NHM_1009, anterior end of live specimen in lateral view.

Damaged specimen (Fig. 76) consistent with placement within family Paraonidae, based on morphology and DNA.

### Pilargidae Saint-Joseph, 1899

#### Ancistrosyllis sp. (NHM_765)

8D4B071D-E950-5F80-9C31-BDDFEBC07116

##### Materials

- **Type status:** Other material. **Occurrence:** catalogNumber: NHMUK ANEA 2023.479; recordNumber: NHM_1385; recordedBy: Adrian Glover | Helena Wiklund | Thomas Dahlgren | Madeleine Brasier; individualCount: 1; preparations: specimen stored in 80% non-denatured ethanol aqueous solution | DNA voucher stored in buffer; otherCatalogNumbers: 0174126735; associatedSequences: OQ746677 (16S); occurrenceID: C76D3090-490F-50EC-BE13-91B6B3DE755E; **Taxon:** taxonConceptID: Ancistrosyllis sp. (NHM_765); scientificName: Ancistrosyllis; kingdom: Animalia; phylum: Annelida; class: Polychaeta; order: Phyllodocida; family: Pilargidae; genus: Ancistrosyllis; taxonRank: genus; scientificNameAuthorship: McIntosh, 1878; **Location:** waterBody: Pacific; stateProvince: Clarion Clipperton Zone; locality: Ocean Mineral Singapore exploration claim Stratum A; verbatimLocality: OMS Stratum A; maximumDepthInMeters: 4044; locationRemarks: Deployment BC12; at Station S6; from R/V Thomas G. Thompson Cruise no. TN319; verbatimLatitude: 12'08.695; verbatimLongitude: 117'19.526; decimalLatitude: 12.14492; decimalLongitude: -117.32543; geodeticDatum: WGS84; **Identification:** identifiedBy: Helena Wiklund | Lenka Neal | Thomas Dahlgren | Adrian Glover | Madeleine Brasier | Regan Drennan | Eva Stewart; dateIdentified: 2021-04-20; identificationRemarks: identified by DNA and morphology; **Event:** eventID: OMS1_AB02_BC12; samplingProtocol: USNEL Box Core; eventDate: 2015-03-02; eventTime: 02:20; habitat: Abyssal plain; fieldNotes: Collected from 0-2 cm layer of box core using a 300 micron sieve; **Record Level:** language: en; institutionCode: NHMUK; collectionCode: ZOO; datasetName: ABYSSLINE; basisOfRecord: PreservedSpecimen
- **Type status:** Other material. **Occurrence:** catalogNumber: NHMUK ANEA 2023.483; recordNumber: NHM_1775; recordedBy: Adrian Glover | Helena Wiklund | Thomas Dahlgren | Madeleine Brasier; individualCount: 1; preparations: specimen stored in 80% non-denatured ethanol aqueous solution | DNA voucher stored in buffer; otherCatalogNumbers: 0174126172; associatedSequences: OQ746718 (16S); occurrenceID: 3F596A09-AF50-51B0-B9C4-BC7A62CE9596; **Taxon:** taxonConceptID: Ancistrosyllis sp. (NHM_765); scientificName: Ancistrosyllis; kingdom: Animalia; phylum: Annelida; class: Polychaeta; order: Phyllodocida; family: Pilargidae; genus: Ancistrosyllis; taxonRank: genus; scientificNameAuthorship: McIntosh, 1878; **Location:** waterBody: Pacific; stateProvince: Clarion Clipperton Zone; locality: Ocean Mineral Singapore exploration claim Stratum A; verbatimLocality: OMS Stratum A; maximumDepthInMeters: 4045; locationRemarks: Deployment EB10; at Station S7; from R/V Thomas G. Thompson Cruise no. TN319; verbatimLatitude: 12'10.43; verbatimLongitude: 117'11.57; decimalLatitude: 12.17383; decimalLongitude: -117.19283; geodeticDatum: WGS84; **Identification:** identifiedBy: Helena Wiklund | Lenka Neal | Thomas Dahlgren | Adrian Glover | Madeleine Brasier | Regan Drennan | Eva Stewart; dateIdentified: 2021-04-20; identificationRemarks: identified by DNA and morphology; **Event:** eventID: OMS1_AB02_EB10; samplingProtocol: Brenke Epibenthic Sledge; eventDate: 2015-03-11; eventTime: 22:49; habitat: Abyssal plain; fieldNotes: Collected from epi net (on the epibenthic sledge); **Record Level:** language: en; institutionCode: NHMUK; collectionCode: ZOO; datasetName: ABYSSLINE; basisOfRecord: PreservedSpecimen
- **Type status:** Other material. **Occurrence:** catalogNumber: NHMUK ANEA 2023.478; recordNumber: NHM_1144; recordedBy: Adrian Glover | Helena Wiklund | Thomas Dahlgren | Madeleine Brasier; individualCount: 1; preparations: specimen stored in 80% non-denatured ethanol aqueous solution | DNA voucher stored in buffer; otherCatalogNumbers: 0174126740; associatedSequences: OQ746624 (16S) | OQ738561 (COI); occurrenceID: CBD086DA-2AB1-5B51-BE17-0D7835DE61B7; **Taxon:** taxonConceptID: Ancistrosyllis sp. (NHM_765); scientificName: Ancistrosyllis; kingdom: Animalia; phylum: Annelida; class: Polychaeta; order: Phyllodocida; family: Pilargidae; genus: Ancistrosyllis; taxonRank: genus; scientificNameAuthorship: McIntosh, 1878; **Location:** waterBody: Pacific; stateProvince: Clarion Clipperton Zone; locality: Ocean Mineral Singapore exploration claim Stratum A; verbatimLocality: OMS Stratum A; maximumDepthInMeters: 4100; locationRemarks: Deployment EB05; at Station S2; from R/V Thomas G. Thompson Cruise no. TN319; verbatimLatitude: 12'06.93; verbatimLongitude: 117'09.87; decimalLatitude: 12.1155; decimalLongitude: -117.1645; geodeticDatum: WGS84; **Identification:** identifiedBy: Helena Wiklund | Lenka Neal | Thomas Dahlgren | Adrian Glover | Madeleine Brasier | Regan Drennan | Eva Stewart; dateIdentified: 2021-04-20; identificationRemarks: identified by DNA and morphology; **Event:** eventID: OMS1_AB02_EB05; samplingProtocol: Brenke Epibenthic Sledge; eventDate: 2015-02-26; eventTime: 21:29; habitat: Abyssal plain; fieldNotes: Collected from epi net (on the epibenthic sledge); **Record Level:** language: en; institutionCode: NHMUK; collectionCode: ZOO; datasetName: ABYSSLINE; basisOfRecord: PreservedSpecimen
- **Type status:** Other material. **Occurrence:** catalogNumber: NHMUK ANEA 2023.476; recordNumber: NHM_0860; recordedBy: Adrian Glover | Helena Wiklund | Thomas Dahlgren | Madeleine Brasier; individualCount: 1; preparations: specimen stored in 80% non-denatured ethanol aqueous solution | DNA voucher stored in buffer; otherCatalogNumbers: 0174126758; associatedSequences: OQ746568 (16S) | OQ738539 (COI); occurrenceID: 8C0CFED9-2787-59DB-9957-D1737C52A253; **Taxon:** taxonConceptID: Ancistrosyllis sp. (NHM_765); scientificName: Ancistrosyllis; kingdom: Animalia; phylum: Annelida; class: Polychaeta; order: Phyllodocida; family: Pilargidae; genus: Ancistrosyllis; taxonRank: genus; scientificNameAuthorship: McIntosh, 1878; **Location:** waterBody: Pacific; stateProvince: Clarion Clipperton Zone; locality: UK Seabed Resources Ltd exploration area UK-1 Stratum B; verbatimLocality: UK1 Stratum B; maximumDepthInMeters: 4237; locationRemarks: Deployment BC06; at Station U6; from R/V Thomas G. Thompson Cruise no. TN319; verbatimLatitude: 12'34.742; verbatimLongitude: 116'41.218; decimalLatitude: 12.57903; decimalLongitude: -116.68697; geodeticDatum: WGS84; **Identification:** identifiedBy: Helena Wiklund | Lenka Neal | Thomas Dahlgren | Adrian Glover | Madeleine Brasier | Regan Drennan | Eva Stewart; dateIdentified: 2021-04-20; identificationRemarks: identified by DNA and morphology; **Event:** eventID: UK1_AB02_BC06; samplingProtocol: USNEL Box Core; eventDate: 2015-02-22; eventTime: 05:08; habitat: Abyssal plain; fieldNotes: Collected from 0-2 cm layer of box core using a 300 micron sieve; **Record Level:** language: en; institutionCode: NHMUK; collectionCode: ZOO; datasetName: ABYSSLINE; basisOfRecord: PreservedSpecimen
- **Type status:** Other material. **Occurrence:** catalogNumber: NHMUK ANEA 2023.480; recordNumber: NHM_1424; recordedBy: Adrian Glover | Helena Wiklund | Thomas Dahlgren | Madeleine Brasier; individualCount: 1; preparations: specimen stored in 80% non-denatured ethanol aqueous solution | DNA voucher stored in buffer; otherCatalogNumbers: 0174126734; associatedSequences: OQ746678 (16S) | OQ738583 (COI); occurrenceID: 1F587B14-6BB3-585C-9F8C-ED1BC7D96DB1; **Taxon:** taxonConceptID: Ancistrosyllis sp. (NHM_765); scientificName: Ancistrosyllis; kingdom: Animalia; phylum: Annelida; class: Polychaeta; order: Phyllodocida; family: Pilargidae; genus: Ancistrosyllis; taxonRank: genus; scientificNameAuthorship: McIntosh, 1878; **Location:** waterBody: Pacific; stateProvince: Clarion Clipperton Zone; locality: UK Seabed Resources Ltd exploration area UK-1 Stratum B; verbatimLocality: UK1 Stratum B; maximumDepthInMeters: 4137; locationRemarks: Deployment EB07; at Station U7; from R/V Thomas G. Thompson Cruise no. TN319; verbatimLatitude: 12'27.26; verbatimLongitude: 116'36.77; decimalLatitude: 12.45433; decimalLongitude: -116.61283; geodeticDatum: WGS84; **Identification:** identifiedBy: Helena Wiklund | Lenka Neal | Thomas Dahlgren | Adrian Glover | Madeleine Brasier | Regan Drennan | Eva Stewart; dateIdentified: 2021-04-20; identificationRemarks: identified by DNA and morphology; **Event:** eventID: UK1_AB02_EB07; samplingProtocol: Brenke Epibenthic Sledge; eventDate: 2015-03-03; eventTime: 20:40; habitat: Abyssal plain; fieldNotes: Collected from epi net (on the epibenthic sledge); **Record Level:** language: en; institutionCode: NHMUK; collectionCode: ZOO; datasetName: ABYSSLINE; basisOfRecord: PreservedSpecimen
- **Type status:** Other material. **Occurrence:** catalogNumber: NHMUK ANEA 2023.481; recordNumber: NHM_1480D; recordedBy: Adrian Glover | Helena Wiklund | Thomas Dahlgren | Madeleine Brasier; individualCount: 1; preparations: specimen stored in 80% non-denatured ethanol aqueous solution | DNA voucher stored in buffer; otherCatalogNumbers: 0174126168; associatedSequences: OQ746685 (16S) | OQ738588 (COI); occurrenceID: 0F3513BD-E36A-5F2D-A286-FE62755A9265; **Taxon:** taxonConceptID: Ancistrosyllis sp. (NHM_765); scientificName: Ancistrosyllis; kingdom: Animalia; phylum: Annelida; class: Polychaeta; order: Phyllodocida; family: Pilargidae; genus: Ancistrosyllis; taxonRank: genus; scientificNameAuthorship: McIntosh, 1878; **Location:** waterBody: Pacific; stateProvince: Clarion Clipperton Zone; locality: UK Seabed Resources Ltd exploration area UK-1 Stratum B; verbatimLocality: UK1 Stratum B; maximumDepthInMeters: 4137; locationRemarks: Deployment EB07; at Station U7; from R/V Thomas G. Thompson Cruise no. TN319; verbatimLatitude: 12'27.26; verbatimLongitude: 116'36.77; decimalLatitude: 12.45433; decimalLongitude: -116.61283; geodeticDatum: WGS84; **Identification:** identifiedBy: Helena Wiklund | Lenka Neal | Thomas Dahlgren | Adrian Glover | Madeleine Brasier | Regan Drennan | Eva Stewart; dateIdentified: 2021-04-20; identificationRemarks: identified by DNA and morphology; **Event:** eventID: UK1_AB02_EB07; samplingProtocol: Brenke Epibenthic Sledge; eventDate: 2015-03-03; eventTime: 20:40; habitat: Abyssal plain; fieldNotes: Collected from epi net (on the epibenthic sledge); **Record Level:** language: en; institutionCode: NHMUK; collectionCode: ZOO; datasetName: ABYSSLINE; basisOfRecord: PreservedSpecimen
- **Type status:** Other material. **Occurrence:** catalogNumber: NHMUK ANEA 2023.477; recordNumber: NHM_0951; recordedBy: Adrian Glover | Helena Wiklund | Thomas Dahlgren | Madeleine Brasier; individualCount: 1; preparations: specimen stored in 80% non-denatured ethanol aqueous solution | DNA voucher stored in buffer; otherCatalogNumbers: 0174127310; associatedSequences: OQ746595 (16S); occurrenceID: A86125FD-1A30-52D0-8A67-DEF5343B2F12; **Taxon:** taxonConceptID: Ancistrosyllis sp. (NHM_765); scientificName: Ancistrosyllis; kingdom: Animalia; phylum: Annelida; class: Polychaeta; order: Phyllodocida; family: Pilargidae; genus: Ancistrosyllis; taxonRank: genus; scientificNameAuthorship: McIntosh, 1878; **Location:** waterBody: Pacific; stateProvince: Clarion Clipperton Zone; locality: UK Seabed Resources Ltd exploration area UK-1 Stratum B; verbatimLocality: UK1 Stratum B; maximumDepthInMeters: 4198; locationRemarks: Deployment EB03; at Station U4; from R/V Thomas G. Thompson Cruise no. TN319; verbatimLatitude: 12'34.28; verbatimLongitude: 116'36.63; decimalLatitude: 12.57133; decimalLongitude: -116.6105; geodeticDatum: WGS84; **Identification:** identifiedBy: Helena Wiklund | Lenka Neal | Thomas Dahlgren | Adrian Glover | Madeleine Brasier | Regan Drennan | Eva Stewart; dateIdentified: 2021-04-20; identificationRemarks: identified by DNA and morphology; **Event:** eventID: UK1_AB02_EB03; samplingProtocol: Brenke Epibenthic Sledge; eventDate: 2015-02-23; eventTime: 05:39; habitat: Abyssal plain; fieldNotes: Collected from epi net (on the epibenthic sledge); **Record Level:** language: en; institutionCode: NHMUK; collectionCode: ZOO; datasetName: ABYSSLINE; basisOfRecord: PreservedSpecimen
- **Type status:** Other material. **Occurrence:** catalogNumber: NHMUK ANEA 2023.475; recordNumber: NHM_0765; recordedBy: Adrian Glover | Helena Wiklund | Thomas Dahlgren | Madeleine Brasier; individualCount: 1; preparations: specimen stored in 80% non-denatured ethanol aqueous solution | DNA voucher stored in buffer; otherCatalogNumbers: 0174127377; associatedSequences: OQ746555 (16S) | OQ746858 (18S) | OQ738535 (COI); occurrenceID: 7D64D041-E51D-57FF-92D9-03DDFEF76D33; **Taxon:** taxonConceptID: Ancistrosyllis sp. (NHM_765); scientificName: Ancistrosyllis; kingdom: Animalia; phylum: Annelida; class: Polychaeta; order: Phyllodocida; family: Pilargidae; genus: Ancistrosyllis; taxonRank: genus; scientificNameAuthorship: McIntosh, 1878; **Location:** waterBody: Pacific; stateProvince: Clarion Clipperton Zone; locality: UK Seabed Resources Ltd exploration area UK-1 Stratum B; verbatimLocality: UK1 Stratum B; maximumDepthInMeters: 4425; locationRemarks: Deployment EB02; at Station U5; from R/V Thomas G. Thompson Cruise no. TN319; verbatimLatitude: 12'32.23; verbatimLongitude: 116'36.25; decimalLatitude: 12.53717; decimalLongitude: -116.60417; geodeticDatum: WGS84; **Identification:** identifiedBy: Helena Wiklund | Lenka Neal | Thomas Dahlgren | Adrian Glover | Madeleine Brasier | Regan Drennan | Eva Stewart; dateIdentified: 2021-04-20; identificationRemarks: identified by DNA and morphology; **Event:** eventID: UK1_AB02_EB02; samplingProtocol: Brenke Epibenthic Sledge; eventDate: 2015-02-20; eventTime: 06:24; habitat: Abyssal plain; fieldNotes: Collected from epi net (on the epibenthic sledge); **Record Level:** language: en; institutionCode: NHMUK; collectionCode: ZOO; datasetName: ABYSSLINE; basisOfRecord: PreservedSpecimen
- **Type status:** Other material. **Occurrence:** catalogNumber: NHMUK ANEA 2023.482; recordNumber: NHM_1508B; recordedBy: Adrian Glover | Helena Wiklund | Thomas Dahlgren | Madeleine Brasier; individualCount: 1; preparations: specimen stored in 80% non-denatured ethanol aqueous solution | DNA voucher stored in buffer; otherCatalogNumbers: 0174126238; associatedSequences: OQ746689 (16S) | OQ738590 (COI); occurrenceID: 02AD9C23-0E55-5025-AD4B-22E7D11DED7A; **Taxon:** taxonConceptID: Ancistrosyllis sp. (NHM_765); scientificName: Ancistrosyllis; kingdom: Animalia; phylum: Annelida; class: Polychaeta; order: Phyllodocida; family: Pilargidae; genus: Ancistrosyllis; taxonRank: genus; scientificNameAuthorship: McIntosh, 1878; **Location:** waterBody: Pacific; stateProvince: Clarion Clipperton Zone; locality: UK Seabed Resources Ltd exploration area UK-1 Stratum B; verbatimLocality: UK1 Stratum B; maximumDepthInMeters: 4196; locationRemarks: Deployment BC15; at Station U9; from R/V Thomas G. Thompson Cruise no. TN319; verbatimLatitude: 12'27.107; verbatimLongitude: 116'30.736; decimalLatitude: 12.45178; decimalLongitude: -116.51227; geodeticDatum: WGS84; **Identification:** identifiedBy: Helena Wiklund | Lenka Neal | Thomas Dahlgren | Adrian Glover | Madeleine Brasier | Regan Drennan | Eva Stewart; dateIdentified: 2021-04-20; identificationRemarks: identified by DNA and morphology; **Event:** eventID: UK1_AB02_BC15; samplingProtocol: USNEL Box Core; eventDate: 2015-03-04; eventTime: 23:23; habitat: Abyssal plain; fieldNotes: Collected from 0-2 cm layer of box core using a 300 micron sieve; **Record Level:** language: en; institutionCode: NHMUK; collectionCode: ZOO; datasetName: ABYSSLINE; basisOfRecord: PreservedSpecimen

##### Distribution

Eastern Clarion-Clipperton Zone, central Pacific Ocean.

##### Diagnosis

Specimens (Fig. 77) consistent with placement within genus *Ancistrosyllis*, based on morphology and DNA. Similar morphologically to *Ancistrosyllisgroenlandica* McIntosh, 1878, but without comparative genetic data from *A.groenlandica* type locality.

### Polynoidae Kinberg, 1856

#### Bathyeliasona mariaae

Bonifácio & Menot, 2018

03B15436-2A8D-5087-BC4A-D65251470EC8

##### Materials

- **Type status:** Other material. **Occurrence:** catalogNumber: NHMUK ANEA 2023.547; recordNumber: NHM_2100; recordedBy: Adrian Glover | Helena Wiklund | Thomas Dahlgren | Madeleine Brasier; individualCount: 1; preparations: specimen stored in 80% non-denatured ethanol aqueous solution | DNA voucher stored in buffer; otherCatalogNumbers: 0174126814; associatedSequences: OQ746754 (16S) | OQ746907 (18S); occurrenceID: BD82225F-1C38-587C-8CE8-F084C2F6643D; **Taxon:** scientificName: Bathyeliasonamariaae; kingdom: Animalia; phylum: Annelida; class: Polychaeta; order: Phyllodocida; family: Polynoidae; genus: Bathyeliasona; specificEpithet: mariaae; taxonRank: species; scientificNameAuthorship: Bonifácio & Menot, 2018; **Location:** waterBody: Pacific; stateProvince: Clarion Clipperton Zone; locality: Area of Particular Interest APEI-6; verbatimLocality: APEI-6; maximumDepthInMeters: 4026; locationRemarks: Deployment EB13; at Station APEI; from R/V Thomas G. Thompson Cruise no. TN319; verbatimLatitude: 19 27.874; verbatimLongitude: 120 01.525; decimalLatitude: 19.46457; decimalLongitude: -120.02542; geodeticDatum: WGS84; **Identification:** identifiedBy: Helena Wiklund | Lenka Neal | Thomas Dahlgren | Adrian Glover | Madeleine Brasier | Regan Drennan | Eva Stewart; dateIdentified: 2021-04-20; identificationRemarks: identified by DNA and morphology; **Event:** eventID: APEI6_AB02_EB13; samplingProtocol: Brenke Epibenthic Sledge; eventDate: 2015-03-20; eventTime: 16:12; habitat: Abyssal plain; fieldNotes: Collected from epi net (on the epibenthic sledge); **Record Level:** language: en; institutionCode: NHMUK; collectionCode: ZOO; datasetName: ABYSSLINE; basisOfRecord: PreservedSpecimen
- **Type status:** Other material. **Occurrence:** catalogNumber: NHMUK ANEA 2023.550; recordNumber: NHM_2450; recordedBy: Adrian Glover | Helena Wiklund | Thomas Dahlgren | Madeleine Brasier; individualCount: 1; preparations: specimen stored in 80% non-denatured ethanol aqueous solution | DNA voucher stored in buffer; otherCatalogNumbers: 0174126204; associatedSequences: OQ746775 (16S); occurrenceID: CEE05701-ADD9-5E33-A264-70A0B4C2C91B; **Taxon:** scientificName: Bathyeliasonamariaae; kingdom: Animalia; phylum: Annelida; class: Polychaeta; order: Phyllodocida; family: Polynoidae; genus: Bathyeliasona; specificEpithet: mariaae; taxonRank: species; scientificNameAuthorship: Bonifácio & Menot, 2018; **Location:** waterBody: Pacific; stateProvince: Clarion Clipperton Zone; locality: UK Seabed Resources Ltd exploration area UK-1 Stratum B; verbatimLocality: UK1 Stratum B; maximumDepthInMeters: 4202; locationRemarks: Deployment EB01; at Station U2; from R/V Thomas G. Thompson Cruise no. TN319; verbatimLatitude: 12'23.17456; verbatimLongitude: 116'32.92021; decimalLatitude: 12.38624; decimalLongitude: -116.54867; geodeticDatum: WGS84; **Identification:** identifiedBy: Helena Wiklund | Lenka Neal | Thomas Dahlgren | Adrian Glover | Madeleine Brasier | Regan Drennan | Eva Stewart; dateIdentified: 2021-04-20; identificationRemarks: identified by DNA and morphology; **Event:** eventID: UK1_AB02_EB01; samplingProtocol: Brenke Epibenthic Sledge; eventDate: 2015-02-17; eventTime: 05:15; habitat: Abyssal plain; fieldNotes: Collected from supra net (on the epibenthic sledge); **Record Level:** language: en; institutionCode: NHMUK; collectionCode: ZOO; datasetName: ABYSSLINE; basisOfRecord: PreservedSpecimen

##### Distribution

Eastern Clarion-Clipperton Zone, central Pacific Ocean.

##### Diagnosis

**Figure 78.**
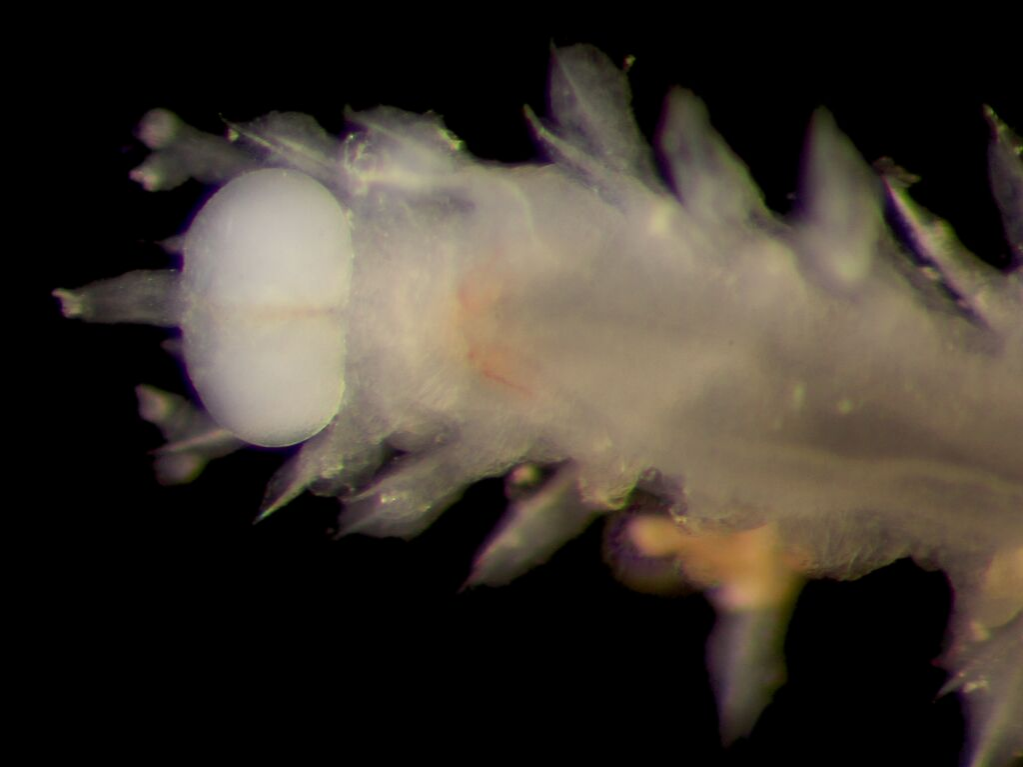
*Bathyeliasonamariaae*, anterior end of live specimen NHM_210 in dorsal view.

Specimens (Fig. 78) consistent with *Bathyeliasonamariaae*, based on morphology and DNA.

#### Bathyfauvelia glacigena

Bonifácio & Menot, 2018

82D8EF88-1340-5785-8990-1120EC5246E9

##### Materials

- **Type status:** Other material. **Occurrence:** catalogNumber: NHMUK ANEA 2023.545; recordNumber: NHM_1269; recordedBy: Adrian Glover | Helena Wiklund | Thomas Dahlgren | Madeleine Brasier; individualCount: 1; preparations: specimen stored in 80% non-denatured ethanol aqueous solution | DNA voucher stored in buffer; otherCatalogNumbers: 0174127381; associatedSequences: OQ746649 (16S) | OQ746885 (18S); occurrenceID: 025A850C-3796-565F-ACBF-F65EE9E09F48; **Taxon:** scientificName: Bathyfauveliaglacigena; kingdom: Animalia; phylum: Annelida; class: Polychaeta; order: Phyllodocida; family: Polynoidae; genus: Bathyfauvelia; specificEpithet: glacigena; taxonRank: species; scientificNameAuthorship: Bonifácio & Menot, 2018; **Location:** waterBody: Pacific; stateProvince: Clarion Clipperton Zone; locality: Ocean Mineral Singapore exploration claim Stratum A; verbatimLocality: OMS Stratum A; maximumDepthInMeters: 4302; locationRemarks: Deployment EB06; at Station S5; from R/V Thomas G. Thompson Cruise no. TN319; verbatimLatitude: 12'15.44; verbatimLongitude: 117'18.13; decimalLatitude: 12.25733; decimalLongitude: -117.30217; geodeticDatum: WGS84; **Identification:** identifiedBy: Helena Wiklund | Lenka Neal | Thomas Dahlgren | Adrian Glover | Madeleine Brasier | Regan Drennan | Eva Stewart; dateIdentified: 2021-04-20; identificationRemarks: identified by DNA and morphology; **Event:** eventID: OMS1_AB02_EB06; samplingProtocol: Brenke Epibenthic Sledge; eventDate: 2015-03-01; eventTime: 04:02; habitat: Abyssal plain; fieldNotes: Collected from epi net (on the epibenthic sledge); **Record Level:** language: en; institutionCode: NHMUK; collectionCode: ZOO; datasetName: ABYSSLINE; basisOfRecord: PreservedSpecimen
- **Type status:** Other material. **Occurrence:** catalogNumber: NHMUK ANEA 2023.549; recordNumber: NHM_2254; recordedBy: Adrian Glover | Helena Wiklund | Thomas Dahlgren | Madeleine Brasier; individualCount: 1; preparations: specimen stored in 80% non-denatured ethanol aqueous solution | DNA voucher stored in buffer; otherCatalogNumbers: 0174126202; associatedSequences: OQ746766 (16S); occurrenceID: 1782B711-4720-5104-B354-023538DDE095; **Taxon:** scientificName: Bathyfauveliaglacigena; kingdom: Animalia; phylum: Annelida; class: Polychaeta; order: Phyllodocida; family: Polynoidae; genus: Bathyfauvelia; specificEpithet: glacigena; taxonRank: species; scientificNameAuthorship: Bonifácio & Menot, 2018; **Location:** waterBody: Pacific; stateProvince: Clarion Clipperton Zone; locality: Ocean Mineral Singapore exploration claim Stratum A; verbatimLocality: OMS Stratum A; maximumDepthInMeters: 4302; locationRemarks: Deployment EB06; at Station S5; from R/V Thomas G. Thompson Cruise no. TN319; verbatimLatitude: 12'15.44; verbatimLongitude: 117'18.13; decimalLatitude: 12.25733; decimalLongitude: -117.30217; geodeticDatum: WGS84; **Identification:** identifiedBy: Helena Wiklund | Lenka Neal | Thomas Dahlgren | Adrian Glover | Madeleine Brasier | Regan Drennan | Eva Stewart; dateIdentified: 2021-04-20; identificationRemarks: identified by DNA and morphology; **Event:** eventID: OMS1_AB02_EB06; samplingProtocol: Brenke Epibenthic Sledge; eventDate: 2015-03-01; eventTime: 04:02; habitat: Abyssal plain; fieldNotes: Collected from supra net (on the epibenthic sledge); **Record Level:** language: en; institutionCode: NHMUK; collectionCode: ZOO; datasetName: ABYSSLINE; basisOfRecord: PreservedSpecimen
- **Type status:** Other material. **Occurrence:** catalogNumber: NHMUK ANEA 2023.553; recordNumber: NHM_3193; recordedBy: Adrian Glover | Helena Wiklund | Thomas Dahlgren | Madeleine Brasier; individualCount: 1; preparations: specimen stored in 80% non-denatured ethanol aqueous solution | DNA voucher stored in buffer; otherCatalogNumbers: 0174126206; associatedSequences: OQ746788 (16S); occurrenceID: 92730C6E-0178-57F2-A21B-DDC5DA266DD6; **Taxon:** scientificName: Bathyfauveliaglacigena; kingdom: Animalia; phylum: Annelida; class: Polychaeta; order: Phyllodocida; family: Polynoidae; genus: Bathyfauvelia; specificEpithet: glacigena; taxonRank: species; scientificNameAuthorship: Bonifácio & Menot, 2018; **Location:** waterBody: Pacific; stateProvince: Clarion Clipperton Zone; locality: Ocean Mineral Singapore exploration claim Stratum A; verbatimLocality: OMS Stratum A; maximumDepthInMeters: 4122; locationRemarks: Deployment EB04; at Station S1; from R/V Thomas G. Thompson Cruise no. TN319; verbatimLatitude: 12'08.02; verbatimLongitude: 117'17.52; decimalLatitude: 12.13367; decimalLongitude: -117.292; geodeticDatum: WGS84; **Identification:** identifiedBy: Helena Wiklund | Lenka Neal | Thomas Dahlgren | Adrian Glover | Madeleine Brasier | Regan Drennan | Eva Stewart; dateIdentified: 2021-04-20; identificationRemarks: identified by DNA and morphology; **Event:** eventID: OMS1_AB02_EB04; samplingProtocol: Brenke Epibenthic Sledge; eventDate: 2015-02-24; eventTime: 19:10; habitat: Abyssal plain; fieldNotes: Collected from supra net (on the epibenthic sledge); **Record Level:** language: en; institutionCode: NHMUK; collectionCode: ZOO; datasetName: ABYSSLINE; basisOfRecord: PreservedSpecimen
- **Type status:** Other material. **Occurrence:** catalogNumber: NHMUK ANEA 2023.544; recordNumber: NHM_1136; recordedBy: Adrian Glover | Helena Wiklund | Thomas Dahlgren | Madeleine Brasier; individualCount: 1; preparations: specimen stored in 80% non-denatured ethanol aqueous solution | DNA voucher stored in buffer; otherCatalogNumbers: 0174127355; associatedSequences: OQ746621 (16S) | OQ738559 (COI); occurrenceID: 5C635EDA-2862-5DC4-83BB-B9A7739F6459; **Taxon:** scientificName: Bathyfauveliaglacigena; kingdom: Animalia; phylum: Annelida; class: Polychaeta; order: Phyllodocida; family: Polynoidae; genus: Bathyfauvelia; specificEpithet: glacigena; taxonRank: species; scientificNameAuthorship: Bonifácio & Menot, 2018; **Location:** waterBody: Pacific; stateProvince: Clarion Clipperton Zone; locality: Ocean Mineral Singapore exploration claim Stratum A; verbatimLocality: OMS Stratum A; maximumDepthInMeters: 4100; locationRemarks: Deployment EB05; at Station S2; from R/V Thomas G. Thompson Cruise no. TN319; verbatimLatitude: 12'06.93; verbatimLongitude: 117'09.87; decimalLatitude: 12.1155; decimalLongitude: -117.1645; geodeticDatum: WGS84; **Identification:** identifiedBy: Helena Wiklund | Lenka Neal | Thomas Dahlgren | Adrian Glover | Madeleine Brasier | Regan Drennan | Eva Stewart; dateIdentified: 2021-04-20; identificationRemarks: identified by DNA and morphology; **Event:** eventID: OMS1_AB02_EB05; samplingProtocol: Brenke Epibenthic Sledge; eventDate: 2015-02-26; eventTime: 21:29; habitat: Abyssal plain; fieldNotes: Collected from epi net (on the epibenthic sledge); **Record Level:** language: en; institutionCode: NHMUK; collectionCode: ZOO; datasetName: ABYSSLINE; basisOfRecord: PreservedSpecimen
- **Type status:** Other material. **Occurrence:** catalogNumber: NHMUK ANEA 2023.548; recordNumber: NHM_2197; recordedBy: Adrian Glover | Helena Wiklund | Thomas Dahlgren | Madeleine Brasier; individualCount: 1; preparations: specimen stored in 80% non-denatured ethanol aqueous solution | DNA voucher stored in buffer; otherCatalogNumbers: 0174126727; associatedSequences: OQ746764 (16S); occurrenceID: 4833D11F-F7BB-5423-87C3-C97ACBC024C7; **Taxon:** scientificName: Bathyfauveliaglacigena; kingdom: Animalia; phylum: Annelida; class: Polychaeta; order: Phyllodocida; family: Polynoidae; genus: Bathyfauvelia; specificEpithet: glacigena; taxonRank: species; scientificNameAuthorship: Bonifácio & Menot, 2018; **Location:** waterBody: Pacific; stateProvince: Clarion Clipperton Zone; locality: Ocean Mineral Singapore exploration claim Stratum A; verbatimLocality: OMS Stratum A; maximumDepthInMeters: 4100; locationRemarks: Deployment EB05; at Station S2; from R/V Thomas G. Thompson Cruise no. TN319; verbatimLatitude: 12'06.93; verbatimLongitude: 117'09.87; decimalLatitude: 12.1155; decimalLongitude: -117.1645; geodeticDatum: WGS84; **Identification:** identifiedBy: Helena Wiklund | Lenka Neal | Thomas Dahlgren | Adrian Glover | Madeleine Brasier | Regan Drennan | Eva Stewart; dateIdentified: 2021-04-20; identificationRemarks: identified by DNA and morphology; **Event:** eventID: OMS1_AB02_EB05; samplingProtocol: Brenke Epibenthic Sledge; eventDate: 2015-02-26; eventTime: 21:29; habitat: Abyssal plain; fieldNotes: Collected from supra net (on the epibenthic sledge); **Record Level:** language: en; institutionCode: NHMUK; collectionCode: ZOO; datasetName: ABYSSLINE; basisOfRecord: PreservedSpecimen
- **Type status:** Other material. **Occurrence:** catalogNumber: NHMUK ANEA 2023.546; recordNumber: NHM_1654; recordedBy: Adrian Glover | Helena Wiklund | Thomas Dahlgren | Madeleine Brasier; individualCount: 1; preparations: specimen stored in 80% non-denatured ethanol aqueous solution | DNA voucher stored in buffer; otherCatalogNumbers: 0174126815; associatedSequences: OQ746701 (16S); occurrenceID: 9B825ACE-7889-5FCB-A8AA-EF77A51BDC6D; **Taxon:** scientificName: Bathyfauveliaglacigena; kingdom: Animalia; phylum: Annelida; class: Polychaeta; order: Phyllodocida; family: Polynoidae; genus: Bathyfauvelia; specificEpithet: glacigena; taxonRank: species; scientificNameAuthorship: Bonifácio & Menot, 2018; **Location:** waterBody: Pacific; stateProvince: Clarion Clipperton Zone; locality: UK Seabed Resources Ltd exploration area UK-1 Stratum B; verbatimLocality: UK1 Stratum B; maximumDepthInMeters: 4233; locationRemarks: Deployment EB09; at Station U1; from R/V Thomas G. Thompson Cruise no. TN319; verbatimLatitude: 12'21.81; verbatimLongitude: 116'40.86; decimalLatitude: 12.3635; decimalLongitude: -116.681; geodeticDatum: WGS84; **Identification:** identifiedBy: Helena Wiklund | Lenka Neal | Thomas Dahlgren | Adrian Glover | Madeleine Brasier | Regan Drennan | Eva Stewart; dateIdentified: 2021-04-20; identificationRemarks: identified by DNA and morphology; **Event:** eventID: UK1_AB02_EB09; samplingProtocol: Brenke Epibenthic Sledge; eventDate: 2015-03-10; eventTime: 10:46; habitat: Abyssal plain; fieldNotes: Collected from epi net (on the epibenthic sledge); **Record Level:** language: en; institutionCode: NHMUK; collectionCode: ZOO; datasetName: ABYSSLINE; basisOfRecord: PreservedSpecimen
- **Type status:** Other material. **Occurrence:** recordNumber: NHM_0749A; recordedBy: Adrian Glover | Helena Wiklund | Thomas Dahlgren | Madeleine Brasier; individualCount: 1; preparations: Tissue voucher stored in 80% non-denatured ethanol aqueous solution | DNA voucher stored in buffer; otherCatalogNumbers: 0109405352 | 0174126530; associatedSequences: OQ746551 (16S); occurrenceID: 3D84B39F-9519-5E5C-AFE7-D98D3413F2A3; **Taxon:** scientificName: Bathyfauveliaglacigena; kingdom: Animalia; phylum: Annelida; class: Polychaeta; order: Phyllodocida; family: Polynoidae; genus: Bathyfauvelia; specificEpithet: glacigena; taxonRank: species; scientificNameAuthorship: Bonifácio & Menot, 2018; **Location:** waterBody: Pacific; stateProvince: Clarion Clipperton Zone; locality: UK Seabed Resources Ltd exploration area UK-1 Stratum B; verbatimLocality: UK1 Stratum B; maximumDepthInMeters: 4425; locationRemarks: Deployment EB02; at Station U5; from R/V Thomas G. Thompson Cruise no. TN319; verbatimLatitude: 12'32.23; verbatimLongitude: 116'36.25; decimalLatitude: 12.53717; decimalLongitude: -116.60417; geodeticDatum: WGS84; **Identification:** identifiedBy: Helena Wiklund | Lenka Neal | Thomas Dahlgren | Adrian Glover | Madeleine Brasier | Regan Drennan | Eva Stewart; dateIdentified: 2021-04-20; identificationRemarks: identified by DNA and morphology; **Event:** eventID: UK1_AB02_EB02; samplingProtocol: Brenke Epibenthic Sledge; eventDate: 2015-02-20; eventTime: 06:24; habitat: Abyssal plain; fieldNotes: Collected from epi net (on the epibenthic sledge); **Record Level:** language: en; institutionCode: NHMUK; collectionCode: ZOO; datasetName: ABYSSLINE; basisOfRecord: PreservedSpecimen
- **Type status:** Other material. **Occurrence:** catalogNumber: NHMUK ANEA 2023.543; recordNumber: NHM_0773D; recordedBy: Adrian Glover | Helena Wiklund | Thomas Dahlgren | Madeleine Brasier; individualCount: 1; preparations: specimen stored in 80% non-denatured ethanol aqueous solution | DNA voucher stored in buffer; otherCatalogNumbers: 0174126601; associatedSequences: OQ746557 (16S); occurrenceID: 92CF56C8-960F-5EA2-857C-BDF2E8FACE09; **Taxon:** scientificName: Bathyfauveliaglacigena; kingdom: Animalia; phylum: Annelida; class: Polychaeta; order: Phyllodocida; family: Polynoidae; genus: Bathyfauvelia; specificEpithet: glacigena; taxonRank: species; scientificNameAuthorship: Bonifácio & Menot, 2018; **Location:** waterBody: Pacific; stateProvince: Clarion Clipperton Zone; locality: UK Seabed Resources Ltd exploration area UK-1 Stratum B; verbatimLocality: UK1 Stratum B; maximumDepthInMeters: 4425; locationRemarks: Deployment EB02; at Station U5; from R/V Thomas G. Thompson Cruise no. TN319; verbatimLatitude: 12'32.23; verbatimLongitude: 116'36.25; decimalLatitude: 12.53717; decimalLongitude: -116.60417; geodeticDatum: WGS84; **Identification:** identifiedBy: Helena Wiklund | Lenka Neal | Thomas Dahlgren | Adrian Glover | Madeleine Brasier | Regan Drennan | Eva Stewart; dateIdentified: 2021-04-20; identificationRemarks: identified by DNA and morphology; **Event:** eventID: UK1_AB02_EB02; samplingProtocol: Brenke Epibenthic Sledge; eventDate: 2015-02-20; eventTime: 06:24; habitat: Abyssal plain; fieldNotes: Collected from epi net (on the epibenthic sledge); **Record Level:** language: en; institutionCode: NHMUK; collectionCode: ZOO; datasetName: ABYSSLINE; basisOfRecord: PreservedSpecimen
- **Type status:** Other material. **Occurrence:** catalogNumber: NHMUK ANEA 2023.552; recordNumber: NHM_2937; recordedBy: Adrian Glover | Helena Wiklund | Thomas Dahlgren | Madeleine Brasier; individualCount: 1; preparations: specimen stored in 80% non-denatured ethanol aqueous solution | DNA voucher stored in buffer; otherCatalogNumbers: 0174126152; associatedSequences: OQ746786 (16S); occurrenceID: E015965C-3A43-58D7-8A82-CEE718CD8E62; **Taxon:** scientificName: Bathyfauveliaglacigena; kingdom: Animalia; phylum: Annelida; class: Polychaeta; order: Phyllodocida; family: Polynoidae; genus: Bathyfauvelia; specificEpithet: glacigena; taxonRank: species; scientificNameAuthorship: Bonifácio & Menot, 2018; **Location:** waterBody: Pacific; stateProvince: Clarion Clipperton Zone; locality: UK Seabed Resources Ltd exploration area UK-1 Stratum B; verbatimLocality: UK1 Stratum B; maximumDepthInMeters: 4425; locationRemarks: Deployment EB02; at Station U5; from R/V Thomas G. Thompson Cruise no. TN319; verbatimLatitude: 12'32.23; verbatimLongitude: 116'36.25; decimalLatitude: 12.53717; decimalLongitude: -116.60417; geodeticDatum: WGS84; **Identification:** identifiedBy: Helena Wiklund | Lenka Neal | Thomas Dahlgren | Adrian Glover | Madeleine Brasier | Regan Drennan | Eva Stewart; dateIdentified: 2021-04-20; identificationRemarks: identified by DNA and morphology; **Event:** eventID: UK1_AB02_EB02; samplingProtocol: Brenke Epibenthic Sledge; eventDate: 2015-02-20; eventTime: 06:24; habitat: Abyssal plain; fieldNotes: Collected from supra net (on the epibenthic sledge); **Record Level:** language: en; institutionCode: NHMUK; collectionCode: ZOO; datasetName: ABYSSLINE; basisOfRecord: PreservedSpecimen

##### Distribution

Eastern Clarion-Clipperton Zone, central Pacific Ocean.

##### Diagnosis

**Figure 79.**
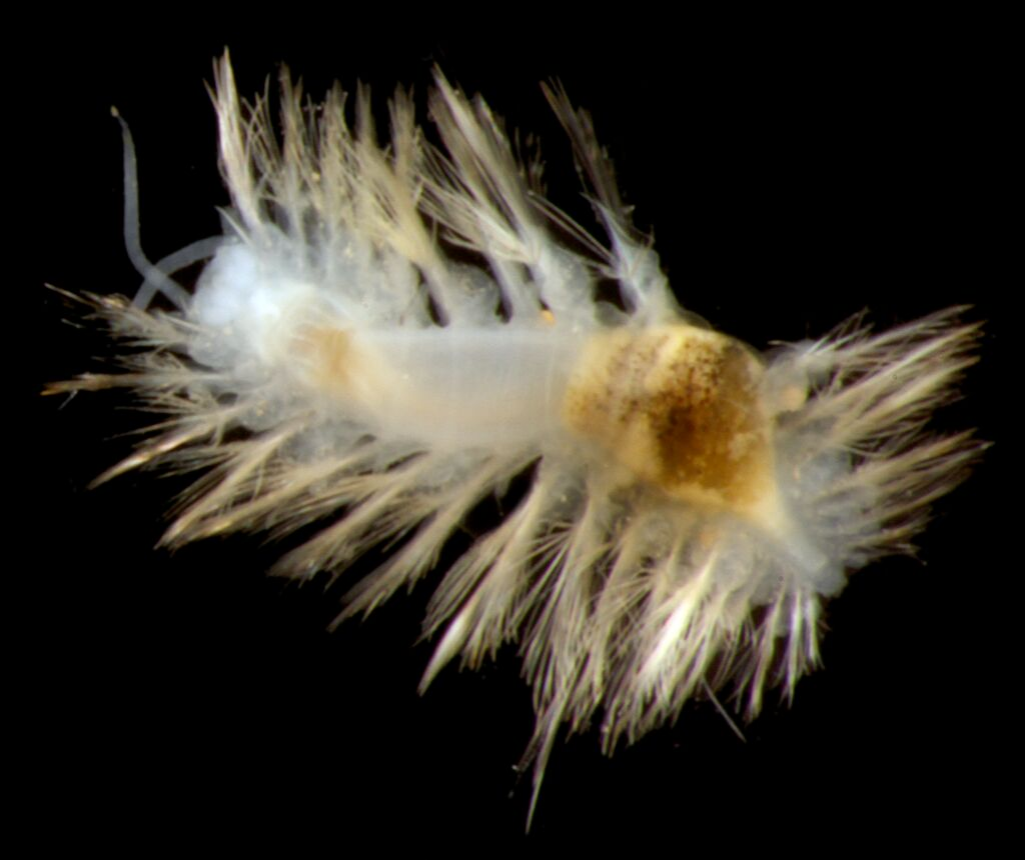
*Bathyfauveliaglacigena*, live complete specimen NHM_1654 in dorsal view.

Specimens (Fig. 79) consistent with *Bathyfauveliaglacigena*, based on morphology and DNA.

#### Bathyfauvelia ignigena

Bonifácio & Menot, 2018

39D9B307-5209-5713-BA0A-1D0CA3ECE459

##### Materials

- **Type status:** Other material. **Occurrence:** recordNumber: NHM_0234; recordedBy: Adrian Glover | Helena Wiklund | Thomas Dahlgren | Magdalena Georgieva; individualCount: 1; preparations: DNA voucher stored in buffer; otherCatalogNumbers: 0174127368; associatedSequences: OQ746487 (16S) | OQ738507 (COI); occurrenceID: 3934F1BB-193B-5AAF-8C10-405DEF8FDF19; **Taxon:** scientificName: Bathyfauveliaignigena; kingdom: Animalia; phylum: Annelida; class: Polychaeta; order: Phyllodocida; family: Polynoidae; genus: Bathyfauvelia; specificEpithet: ignigena; taxonRank: species; scientificNameAuthorship: Bonifácio & Menot, 2018; **Location:** waterBody: Pacific; stateProvince: Clarion Clipperton Zone; locality: UK Seabed Resources Ltd exploration area UK-1 Stratum A; verbatimLocality: UK1 Stratum A; maximumDepthInMeters: 4076; locationRemarks: Deployment BC08; at Station F; from R/V Melville Cruise no. MV1313; verbatimLatitude: 13°48.700; verbatimLongitude: 116°42.600; decimalLatitude: 13.81167; decimalLongitude: -116.71; geodeticDatum: WGS84; **Identification:** identifiedBy: Helena Wiklund | Lenka Neal | Thomas Dahlgren | Adrian Glover | Madeleine Brasier | Regan Drennan | Eva Stewart; dateIdentified: 2021-04-20; identificationRemarks: identified by DNA and morphology; **Event:** eventID: UK1_AB01_BC08; samplingProtocol: USNEL Box Core; eventDate: 2013-10-16; eventTime: 14:33; habitat: Abyssal plain; fieldNotes: Collected from 0-2 cm layer of box core using a 300 micron sieve; **Record Level:** language: en; institutionCode: NHMUK; collectionCode: ZOO; datasetName: ABYSSLINE; basisOfRecord: PreservedSpecimen

##### Distribution

Eastern Clarion-Clipperton Zone, central Pacific Ocean.

##### Diagnosis

Specimen (Fig. 80) consistent with *Bathyfauveliaignigena*, based on morphology and DNA.

#### Polynoidae sp. (NHM_1655)

6276C5E5-AF31-5D91-BC6F-402A222D0CA3

##### Materials

- **Type status:** Other material. **Occurrence:** catalogNumber: NHMUK ANEA 2023.500; recordNumber: NHM_1655; recordedBy: Adrian Glover | Helena Wiklund | Thomas Dahlgren | Madeleine Brasier; individualCount: 1; preparations: specimen stored in 80% non-denatured ethanol aqueous solution | DNA voucher stored in buffer; otherCatalogNumbers: 0174127353; associatedSequences: OQ746702 (16S) | OQ746896 (18S); occurrenceID: A55CDF38-DC9E-5DC4-BDCA-E91D1642675D; **Taxon:** taxonConceptID: Polynoidae sp. (NHM_1655); scientificName: Polynoidae; kingdom: Animalia; phylum: Annelida; class: Polychaeta; order: Phyllodocida; family: Polynoidae; taxonRank: family; scientificNameAuthorship: Kinberg, 1856; **Location:** waterBody: Pacific; stateProvince: Clarion Clipperton Zone; locality: UK Seabed Resources Ltd exploration area UK-1 Stratum B; verbatimLocality: UK1 Stratum B; maximumDepthInMeters: 4233; locationRemarks: Deployment EB09; at Station U1; from R/V Thomas G. Thompson Cruise no. TN319; verbatimLatitude: 12'21.81; verbatimLongitude: 116'40.86; decimalLatitude: 12.3635; decimalLongitude: -116.681; geodeticDatum: WGS84; **Identification:** identifiedBy: Helena Wiklund | Lenka Neal | Thomas Dahlgren | Adrian Glover | Madeleine Brasier | Regan Drennan | Eva Stewart; dateIdentified: 2021-04-20; identificationRemarks: identified by DNA and morphology; **Event:** eventID: UK1_AB02_EB09; samplingProtocol: Brenke Epibenthic Sledge; eventDate: 2015-03-10; eventTime: 10:46; habitat: Abyssal plain; fieldNotes: Collected from epi net (on the epibenthic sledge); **Record Level:** language: en; institutionCode: NHMUK; collectionCode: ZOO; datasetName: ABYSSLINE; basisOfRecord: PreservedSpecimen
- **Type status:** Other material. **Occurrence:** catalogNumber: NHMUK ANEA 2023.501; recordNumber: NHM_1677; recordedBy: Adrian Glover | Helena Wiklund | Thomas Dahlgren | Madeleine Brasier; individualCount: 1; preparations: specimen stored in 80% non-denatured ethanol aqueous solution | DNA voucher stored in buffer; otherCatalogNumbers: 0174127354; associatedSequences: OQ746704 (16S) | OQ746897 (18S); occurrenceID: 66FD4827-D075-58EB-9F15-A89FCF61075F; **Taxon:** taxonConceptID: Polynoidae sp. (NHM_1655); scientificName: Polynoidae; kingdom: Animalia; phylum: Annelida; class: Polychaeta; order: Phyllodocida; family: Polynoidae; taxonRank: family; scientificNameAuthorship: Kinberg, 1856; **Location:** waterBody: Pacific; stateProvince: Clarion Clipperton Zone; locality: UK Seabed Resources Ltd exploration area UK-1 Stratum B; verbatimLocality: UK1 Stratum B; maximumDepthInMeters: 4233; locationRemarks: Deployment EB09; at Station U1; from R/V Thomas G. Thompson Cruise no. TN319; verbatimLatitude: 12'21.81; verbatimLongitude: 116'40.86; decimalLatitude: 12.3635; decimalLongitude: -116.681; geodeticDatum: WGS84; **Identification:** identifiedBy: Helena Wiklund | Lenka Neal | Thomas Dahlgren | Adrian Glover | Madeleine Brasier | Regan Drennan | Eva Stewart; dateIdentified: 2021-04-20; identificationRemarks: identified by DNA and morphology; **Event:** eventID: UK1_AB02_EB09; samplingProtocol: Brenke Epibenthic Sledge; eventDate: 2015-03-10; eventTime: 10:46; habitat: Abyssal plain; fieldNotes: Collected from epi net (on the epibenthic sledge); **Record Level:** language: en; institutionCode: NHMUK; collectionCode: ZOO; datasetName: ABYSSLINE; basisOfRecord: PreservedSpecimen

##### Distribution

Eastern Clarion-Clipperton Zone, central Pacific Ocean.

##### Diagnosis

**Figure 81.**
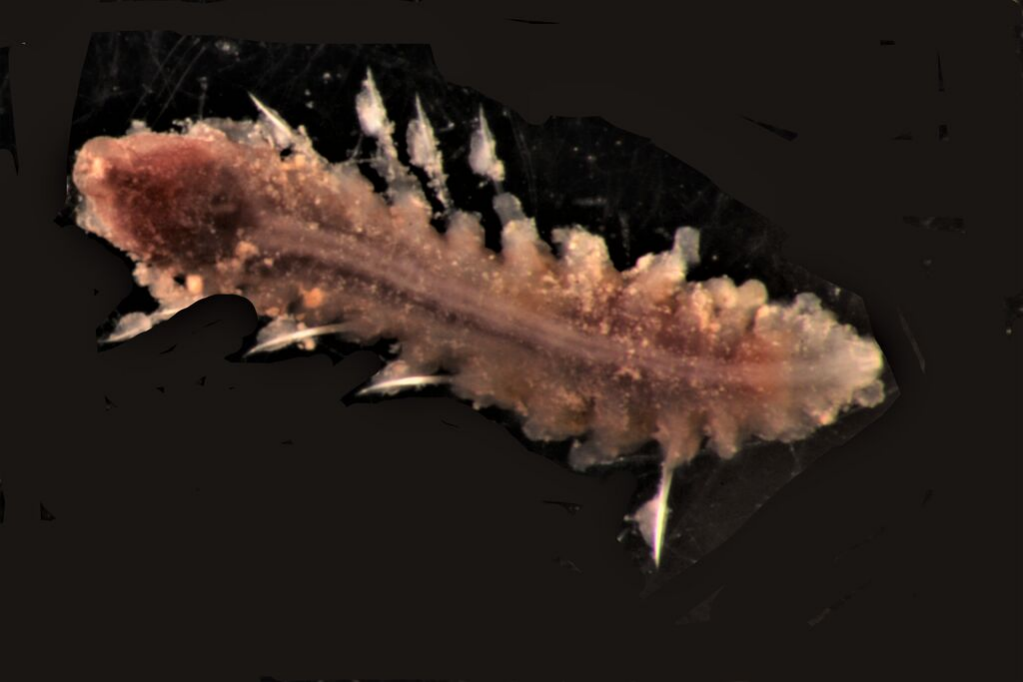
Polynoidae sp. (NHM_1655), complete specimen NHM_1655 in ventral view.

Damaged specimens (Fig. 81) consistent with the family Polynoidae.

#### Polaruschakov lamellae

Bonifácio & Menot, 2018

8B382B8A-1605-52B1-92EB-BC5F1DC75B43

##### Materials

- **Type status:** Other material. **Occurrence:** catalogNumber: NHMUK ANEA 2023.551; recordNumber: NHM_2819; recordedBy: Adrian Glover | Helena Wiklund | Thomas Dahlgren | Madeleine Brasier; individualCount: 1; preparations: specimen stored in 80% non-denatured ethanol aqueous solution | DNA voucher stored in buffer; otherCatalogNumbers: 0174126229; associatedSequences: OQ746785 (16S) | OQ738619 (COI); occurrenceID: DCEC7E85-F3D7-5777-A2FB-149A9FF47993; **Taxon:** scientificName: Polaruschakovlamellae; kingdom: Animalia; phylum: Annelida; class: Polychaeta; order: Phyllodocida; family: Polynoidae; genus: Polaruschakov; specificEpithet: lamellae; taxonRank: species; scientificNameAuthorship: Bonifácio & Menot, 2018; **Location:** waterBody: Pacific; stateProvince: Clarion Clipperton Zone; locality: Ocean Mineral Singapore exploration claim Stratum A; verbatimLocality: OMS Stratum A; maximumDepthInMeters: 4100; locationRemarks: Deployment EB05; at Station S2; from R/V Thomas G. Thompson Cruise no. TN319; verbatimLatitude: 12'06.93; verbatimLongitude: 117'09.87; decimalLatitude: 12.1155; decimalLongitude: -117.1645; geodeticDatum: WGS84; **Identification:** identifiedBy: Helena Wiklund | Lenka Neal | Thomas Dahlgren | Adrian Glover | Madeleine Brasier | Regan Drennan | Eva Stewart; dateIdentified: 2021-04-20; identificationRemarks: identified by DNA and morphology; **Event:** eventID: OMS1_AB02_EB05; samplingProtocol: Brenke Epibenthic Sledge; eventDate: 2015-02-26; eventTime: 21:29; habitat: Abyssal plain; fieldNotes: Collected from supra net (on the epibenthic sledge); **Record Level:** language: en; institutionCode: NHMUK; collectionCode: ZOO; datasetName: ABYSSLINE; basisOfRecord: PreservedSpecimen

##### Distribution

Eastern Clarion-Clipperton Zone, central Pacific Ocean.

##### Diagnosis

Damaged specimen consistent with *Polaruschakovlamellae*, based on DNA data.

#### Polynoidae sp. (NHM_034)

8F05DD6B-5338-55F6-94F9-A5FF1F8ADCFB

##### Materials

- **Type status:** Other material. **Occurrence:** catalogNumber: NHMUK ANEA 2023.530; recordNumber: NHM_0034; recordedBy: Adrian Glover | Helena Wiklund | Thomas Dahlgren | Magdalena Georgieva; individualCount: 1; preparations: specimen stored in 80% non-denatured ethanol aqueous solution | DNA voucher stored in buffer; otherCatalogNumbers: 0174127387; associatedSequences: OQ746469 (16S) | OQ746792 (18S) | OQ738496 (COI); occurrenceID: A2A285A1-4CC0-59AC-9E68-329747C29C96; **Taxon:** taxonConceptID: Polynoidae sp. (NHM_034); scientificName: Polynoidae; kingdom: Animalia; phylum: Annelida; class: Polychaeta; order: Phyllodocida; family: Polynoidae; taxonRank: family; scientificNameAuthorship: Kinberg, 1856; **Location:** waterBody: Pacific; stateProvince: Clarion Clipperton Zone; locality: UK Seabed Resources Ltd exploration area UK-1 Stratum A; verbatimLocality: UK1 Stratum A; maximumDepthInMeters: 4336; locationRemarks: Deployment EB01; at Station B-K-E; from R/V Melville Cruise no. MV1313; verbatimLatitude: 13°50.232; verbatimLongitude: 116°33.506; decimalLatitude: 13.8372; decimalLongitude: -116.55843; geodeticDatum: WGS84; **Identification:** identifiedBy: Helena Wiklund | Lenka Neal | Thomas Dahlgren | Adrian Glover | Madeleine Brasier | Regan Drennan | Eva Stewart; dateIdentified: 2021-04-20; identificationRemarks: identified by DNA and morphology; **Event:** eventID: UK1_AB01_EB01; samplingProtocol: Brenke Epibenthic Sledge; eventDate: 2013-10-09; eventTime: 10:26; habitat: Abyssal plain; fieldNotes: Collected from epi net (on the epibenthic sledge); **Record Level:** language: en; institutionCode: NHMUK; collectionCode: ZOO; datasetName: ABYSSLINE; basisOfRecord: PreservedSpecimen

##### Distribution

Eastern Clarion-Clipperton Zone, central Pacific Ocean.

##### Diagnosis

**Figure 82.**
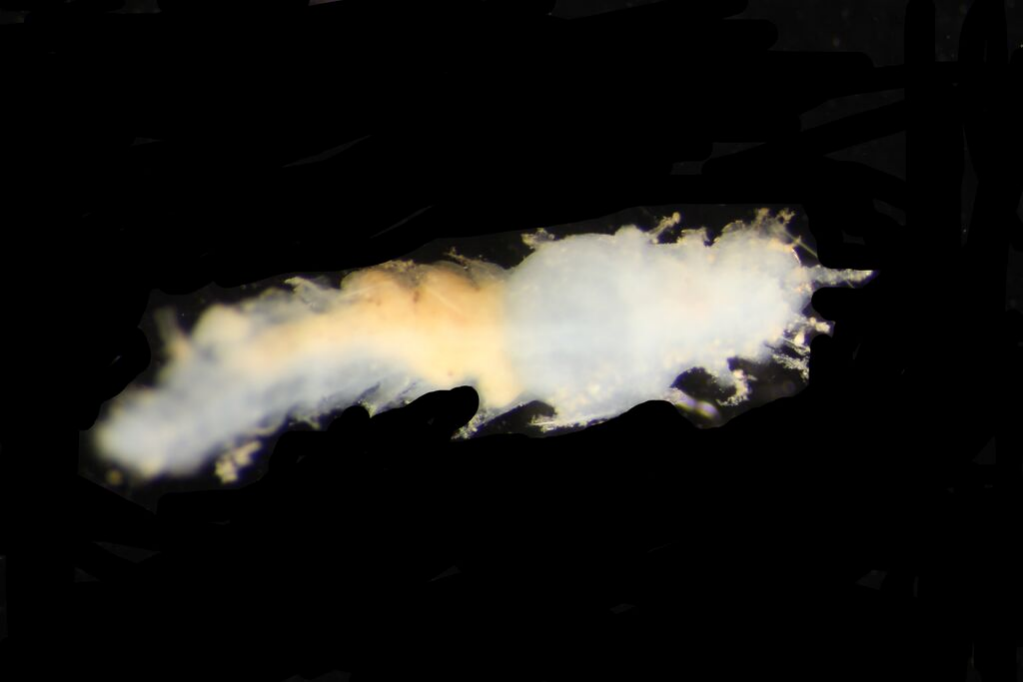
Polynoidae sp. (NHM_034), live specimen NHM_034 in dorsal view.

Damaged specimen (Fig. 82) consistent with placement within family Polynoidae, based on morphology and DNA. Genetically matches with *Polaruschakov* sp. 315 as identified in Bonifácio et al. (2021).

#### Polynoidae sp. (NHM_128)

FEB84DEF-D8A7-5F5F-A4DD-B1D208F575B6

##### Materials

- **Type status:** Other material. **Occurrence:** catalogNumber: NHMUK ANEA 2023.507; recordNumber: NHM_1351A; recordedBy: Adrian Glover | Helena Wiklund | Thomas Dahlgren | Madeleine Brasier; individualCount: 1; preparations: specimen stored in 80% non-denatured ethanol aqueous solution | DNA voucher stored in buffer; otherCatalogNumbers: 0174126540; associatedSequences: OQ746673 (16S); occurrenceID: D681B6F7-F411-5B4A-B4FF-D827DAD651FB; **Taxon:** taxonConceptID: Polynoidae sp. (NHM_128); scientificName: Polynoidae; kingdom: Animalia; phylum: Annelida; class: Polychaeta; order: Phyllodocida; family: Polynoidae; taxonRank: family; scientificNameAuthorship: Kinberg, 1856; **Location:** waterBody: Pacific; stateProvince: Clarion Clipperton Zone; locality: Ocean Mineral Singapore exploration claim Stratum A; verbatimLocality: OMS Stratum A; maximumDepthInMeters: 4302; locationRemarks: Deployment EB06; at Station S5; from R/V Thomas G. Thompson Cruise no. TN319; verbatimLatitude: 12'15.44; verbatimLongitude: 117'18.13; decimalLatitude: 12.25733; decimalLongitude: -117.30217; geodeticDatum: WGS84; **Identification:** identifiedBy: Helena Wiklund | Lenka Neal | Thomas Dahlgren | Adrian Glover | Madeleine Brasier | Regan Drennan | Eva Stewart; dateIdentified: 2021-04-20; identificationRemarks: identified by DNA and morphology; **Event:** eventID: OMS1_AB02_EB06; samplingProtocol: Brenke Epibenthic Sledge; eventDate: 2015-03-01; eventTime: 04:02; habitat: Abyssal plain; fieldNotes: Collected from epi net (on the epibenthic sledge); **Record Level:** language: en; institutionCode: NHMUK; collectionCode: ZOO; datasetName: ABYSSLINE; basisOfRecord: PreservedSpecimen
- **Type status:** Other material. **Occurrence:** catalogNumber: NHMUK ANEA 2023.510; recordNumber: NHM_1925; recordedBy: Adrian Glover | Helena Wiklund | Thomas Dahlgren | Madeleine Brasier; individualCount: 1; preparations: specimen stored in 80% non-denatured ethanol aqueous solution | DNA voucher stored in buffer; otherCatalogNumbers: 0174126767; associatedSequences: OQ746732 (16S); occurrenceID: B1708B48-41EC-5D4F-8793-E7CFCD7C2611; **Taxon:** taxonConceptID: Polynoidae sp. (NHM_128); scientificName: Polynoidae; kingdom: Animalia; phylum: Annelida; class: Polychaeta; order: Phyllodocida; family: Polynoidae; taxonRank: family; scientificNameAuthorship: Kinberg, 1856; **Location:** waterBody: Pacific; stateProvince: Clarion Clipperton Zone; locality: Ocean Mineral Singapore exploration claim Stratum A; verbatimLocality: OMS Stratum A; maximumDepthInMeters: 4094; locationRemarks: Deployment EB11; at Station S10; from R/V Thomas G. Thompson Cruise no. TN319; verbatimLatitude: 12°02.49’; verbatimLongitude: 117°13.03’; decimalLatitude: 12.0415; decimalLongitude: -117.21717; geodeticDatum: WGS84; **Identification:** identifiedBy: Helena Wiklund | Lenka Neal | Thomas Dahlgren | Adrian Glover | Madeleine Brasier | Regan Drennan | Eva Stewart; dateIdentified: 2021-04-20; identificationRemarks: identified by DNA and morphology; **Event:** eventID: OMS1_AB02_EB11; samplingProtocol: Brenke Epibenthic Sledge; eventDate: 2015-03-13; habitat: Abyssal plain; fieldNotes: Collected from epi net (on the epibenthic sledge); **Record Level:** language: en; institutionCode: NHMUK; collectionCode: ZOO; datasetName: ABYSSLINE; basisOfRecord: PreservedSpecimen
- **Type status:** Other material. **Occurrence:** catalogNumber: NHMUK ANEA 2023.509; recordNumber: NHM_1785; recordedBy: Adrian Glover | Helena Wiklund | Thomas Dahlgren | Madeleine Brasier; individualCount: 1; preparations: specimen stored in 80% non-denatured ethanol aqueous solution | DNA voucher stored in buffer; otherCatalogNumbers: 0174126768; associatedSequences: OQ746721 (16S) | OQ746901 (18S) | OQ738600 (COI); occurrenceID: 9A7F3286-CAF0-5EDF-B042-2C5714FC3863; **Taxon:** taxonConceptID: Polynoidae sp. (NHM_128); scientificName: Polynoidae; kingdom: Animalia; phylum: Annelida; class: Polychaeta; order: Phyllodocida; family: Polynoidae; taxonRank: family; scientificNameAuthorship: Kinberg, 1856; **Location:** waterBody: Pacific; stateProvince: Clarion Clipperton Zone; locality: Ocean Mineral Singapore exploration claim Stratum A; verbatimLocality: OMS Stratum A; maximumDepthInMeters: 4045; locationRemarks: Deployment EB10; at Station S7; from R/V Thomas G. Thompson Cruise no. TN319; verbatimLatitude: 12'10.43; verbatimLongitude: 117'11.57; decimalLatitude: 12.17383; decimalLongitude: -117.19283; geodeticDatum: WGS84; **Identification:** identifiedBy: Helena Wiklund | Lenka Neal | Thomas Dahlgren | Adrian Glover | Madeleine Brasier | Regan Drennan | Eva Stewart; dateIdentified: 2021-04-20; identificationRemarks: identified by DNA and morphology; **Event:** eventID: OMS1_AB02_EB10; samplingProtocol: Brenke Epibenthic Sledge; eventDate: 2015-03-11; eventTime: 22:49; habitat: Abyssal plain; fieldNotes: Collected from epi net (on the epibenthic sledge); **Record Level:** language: en; institutionCode: NHMUK; collectionCode: ZOO; datasetName: ABYSSLINE; basisOfRecord: PreservedSpecimen
- **Type status:** Other material. **Occurrence:** catalogNumber: NHMUK ANEA 2023.505; recordNumber: NHM_1164C; recordedBy: Adrian Glover | Helena Wiklund | Thomas Dahlgren | Madeleine Brasier; individualCount: 1; preparations: specimen stored in 80% non-denatured ethanol aqueous solution | DNA voucher stored in buffer; otherCatalogNumbers: 0174126591; associatedSequences: OQ746632 (16S); occurrenceID: 9B4A2CB8-DE47-5978-9555-AD9E28B1A430; **Taxon:** taxonConceptID: Polynoidae sp. (NHM_128); scientificName: Polynoidae; kingdom: Animalia; phylum: Annelida; class: Polychaeta; order: Phyllodocida; family: Polynoidae; taxonRank: family; scientificNameAuthorship: Kinberg, 1856; **Location:** waterBody: Pacific; stateProvince: Clarion Clipperton Zone; locality: Ocean Mineral Singapore exploration claim Stratum A; verbatimLocality: OMS Stratum A; maximumDepthInMeters: 4100; locationRemarks: Deployment EB05; at Station S2; from R/V Thomas G. Thompson Cruise no. TN319; verbatimLatitude: 12'06.93; verbatimLongitude: 117'09.87; decimalLatitude: 12.1155; decimalLongitude: -117.1645; geodeticDatum: WGS84; **Identification:** identifiedBy: Helena Wiklund | Lenka Neal | Thomas Dahlgren | Adrian Glover | Madeleine Brasier | Regan Drennan | Eva Stewart; dateIdentified: 2021-04-20; identificationRemarks: identified by DNA and morphology; **Event:** eventID: OMS1_AB02_EB05; samplingProtocol: Brenke Epibenthic Sledge; eventDate: 2015-02-26; eventTime: 21:29; habitat: Abyssal plain; fieldNotes: Collected from epi net (on the epibenthic sledge); **Record Level:** language: en; institutionCode: NHMUK; collectionCode: ZOO; datasetName: ABYSSLINE; basisOfRecord: PreservedSpecimen
- **Type status:** Other material. **Occurrence:** catalogNumber: NHMUK ANEA 2023.506; recordNumber: NHM_1165B; recordedBy: Adrian Glover | Helena Wiklund | Thomas Dahlgren | Madeleine Brasier; individualCount: 1; preparations: specimen stored in 80% non-denatured ethanol aqueous solution | DNA voucher stored in buffer; otherCatalogNumbers: 0174126567; associatedSequences: OQ746634 (16S); occurrenceID: 5E2D74AD-92E5-5D22-BC58-1D4710D3A711; **Taxon:** taxonConceptID: Polynoidae sp. (NHM_128); scientificName: Polynoidae; kingdom: Animalia; phylum: Annelida; class: Polychaeta; order: Phyllodocida; family: Polynoidae; taxonRank: family; scientificNameAuthorship: Kinberg, 1856; **Location:** waterBody: Pacific; stateProvince: Clarion Clipperton Zone; locality: Ocean Mineral Singapore exploration claim Stratum A; verbatimLocality: OMS Stratum A; maximumDepthInMeters: 4100; locationRemarks: Deployment EB05; at Station S2; from R/V Thomas G. Thompson Cruise no. TN319; verbatimLatitude: 12'06.93; verbatimLongitude: 117'09.87; decimalLatitude: 12.1155; decimalLongitude: -117.1645; geodeticDatum: WGS84; **Identification:** identifiedBy: Helena Wiklund | Lenka Neal | Thomas Dahlgren | Adrian Glover | Madeleine Brasier | Regan Drennan | Eva Stewart; dateIdentified: 2021-04-20; identificationRemarks: identified by DNA and morphology; **Event:** eventID: OMS1_AB02_EB05; samplingProtocol: Brenke Epibenthic Sledge; eventDate: 2015-02-26; eventTime: 21:29; habitat: Abyssal plain; fieldNotes: Collected from epi net (on the epibenthic sledge); **Record Level:** language: en; institutionCode: NHMUK; collectionCode: ZOO; datasetName: ABYSSLINE; basisOfRecord: PreservedSpecimen
- **Type status:** Other material. **Occurrence:** catalogNumber: NHMUK ANEA 2023.503; recordNumber: NHM_0128; recordedBy: Adrian Glover | Helena Wiklund | Thomas Dahlgren | Magdalena Georgieva; individualCount: 1; preparations: specimen stored in 80% non-denatured ethanol aqueous solution | DNA voucher stored in buffer; otherCatalogNumbers: 0174127323; associatedSequences: OQ746479 (16S) | OQ746800 (18S); occurrenceID: ABB33344-D920-598A-842E-0744CA94542D; **Taxon:** taxonConceptID: Polynoidae sp. (NHM_128); scientificName: Polynoidae; kingdom: Animalia; phylum: Annelida; class: Polychaeta; order: Phyllodocida; family: Polynoidae; taxonRank: family; scientificNameAuthorship: Kinberg, 1856; **Location:** waterBody: Pacific; stateProvince: Clarion Clipperton Zone; locality: UK Seabed Resources Ltd exploration area UK-1 Stratum A; verbatimLocality: UK1 Stratum A; maximumDepthInMeters: 4080; locationRemarks: Deployment EB02; at Station C; from R/V Melville Cruise no. MV1313; verbatimLatitude: 13°45.500; verbatimLongitude: 116°41.911; decimalLatitude: 13.75833; decimalLongitude: -116.69852; geodeticDatum: WGS84; **Identification:** identifiedBy: Helena Wiklund | Lenka Neal | Thomas Dahlgren | Adrian Glover | Madeleine Brasier | Regan Drennan | Eva Stewart; dateIdentified: 2021-04-20; identificationRemarks: identified by DNA and morphology; **Event:** eventID: UK1_AB01_EB02; samplingProtocol: Brenke Epibenthic Sledge; eventDate: 2013-10-11; eventTime: 10:32; habitat: Abyssal plain; fieldNotes: Collected from epi net (on the epibenthic sledge); **Record Level:** language: en; institutionCode: NHMUK; collectionCode: ZOO; datasetName: ABYSSLINE; basisOfRecord: PreservedSpecimen
- **Type status:** Other material. **Occurrence:** catalogNumber: NHMUK ANEA 2023.504; recordNumber: NHM_0957D; recordedBy: Adrian Glover | Helena Wiklund | Thomas Dahlgren | Madeleine Brasier; individualCount: 1; preparations: specimen stored in 80% non-denatured ethanol aqueous solution | DNA voucher stored in buffer; otherCatalogNumbers: 0174126593; associatedSequences: OQ746599 (16S); occurrenceID: 3E4A3E59-9E88-5E17-A77C-86EE7C00E029; **Taxon:** taxonConceptID: Polynoidae sp. (NHM_128); scientificName: Polynoidae; kingdom: Animalia; phylum: Annelida; class: Polychaeta; order: Phyllodocida; family: Polynoidae; taxonRank: family; scientificNameAuthorship: Kinberg, 1856; **Location:** waterBody: Pacific; stateProvince: Clarion Clipperton Zone; locality: UK Seabed Resources Ltd exploration area UK-1 Stratum B; verbatimLocality: UK1 Stratum B; maximumDepthInMeters: 4198; locationRemarks: Deployment EB03; at Station U4; from R/V Thomas G. Thompson Cruise no. TN319; verbatimLatitude: 12'34.28; verbatimLongitude: 116'36.63; decimalLatitude: 12.57133; decimalLongitude: -116.6105; geodeticDatum: WGS84; **Identification:** identifiedBy: Helena Wiklund | Lenka Neal | Thomas Dahlgren | Adrian Glover | Madeleine Brasier | Regan Drennan | Eva Stewart; dateIdentified: 2021-04-20; identificationRemarks: identified by DNA and morphology; **Event:** eventID: UK1_AB02_EB03; samplingProtocol: Brenke Epibenthic Sledge; eventDate: 2015-02-23; eventTime: 05:39; habitat: Abyssal plain; fieldNotes: Collected from epi net (on the epibenthic sledge); **Record Level:** language: en; institutionCode: NHMUK; collectionCode: ZOO; datasetName: ABYSSLINE; basisOfRecord: PreservedSpecimen
- **Type status:** Other material. **Occurrence:** catalogNumber: NHMUK ANEA 2023.508; recordNumber: NHM_1533A; recordedBy: Adrian Glover | Helena Wiklund | Thomas Dahlgren | Madeleine Brasier; individualCount: 1; preparations: specimen stored in 80% non-denatured ethanol aqueous solution | DNA voucher stored in buffer; otherCatalogNumbers: 0174126214; associatedSequences: OQ746692 (16S); occurrenceID: 48192FB1-B51E-5580-95FA-20F06B1B0407; **Taxon:** taxonConceptID: Polynoidae sp. (NHM_128); scientificName: Polynoidae; kingdom: Animalia; phylum: Annelida; class: Polychaeta; order: Phyllodocida; family: Polynoidae; taxonRank: family; scientificNameAuthorship: Kinberg, 1856; **Location:** waterBody: Pacific; stateProvince: Clarion Clipperton Zone; locality: UK Seabed Resources Ltd exploration area UK-1 Stratum B; verbatimLocality: UK1 Stratum B; maximumDepthInMeters: 4252; locationRemarks: Deployment EB08; at Station U11; from R/V Thomas G. Thompson Cruise no. TN319; verbatimLatitude: 12'30.79; verbatimLongitude: 116'29.48; decimalLatitude: 12.51317; decimalLongitude: -116.49133; geodeticDatum: WGS84; **Identification:** identifiedBy: Helena Wiklund | Lenka Neal | Thomas Dahlgren | Adrian Glover | Madeleine Brasier | Regan Drennan | Eva Stewart; dateIdentified: 2021-04-20; identificationRemarks: identified by DNA and morphology; **Event:** eventID: UK1_AB02_EB08; samplingProtocol: Brenke Epibenthic Sledge; eventDate: 2015-03-05; eventTime: 18:53; habitat: Abyssal plain; fieldNotes: Collected from epi net (on the epibenthic sledge); **Record Level:** language: en; institutionCode: NHMUK; collectionCode: ZOO; datasetName: ABYSSLINE; basisOfRecord: PreservedSpecimen

##### Distribution

Eastern Clarion-Clipperton Zone, central Pacific Ocean.

##### Diagnosis

**Figure 83.**
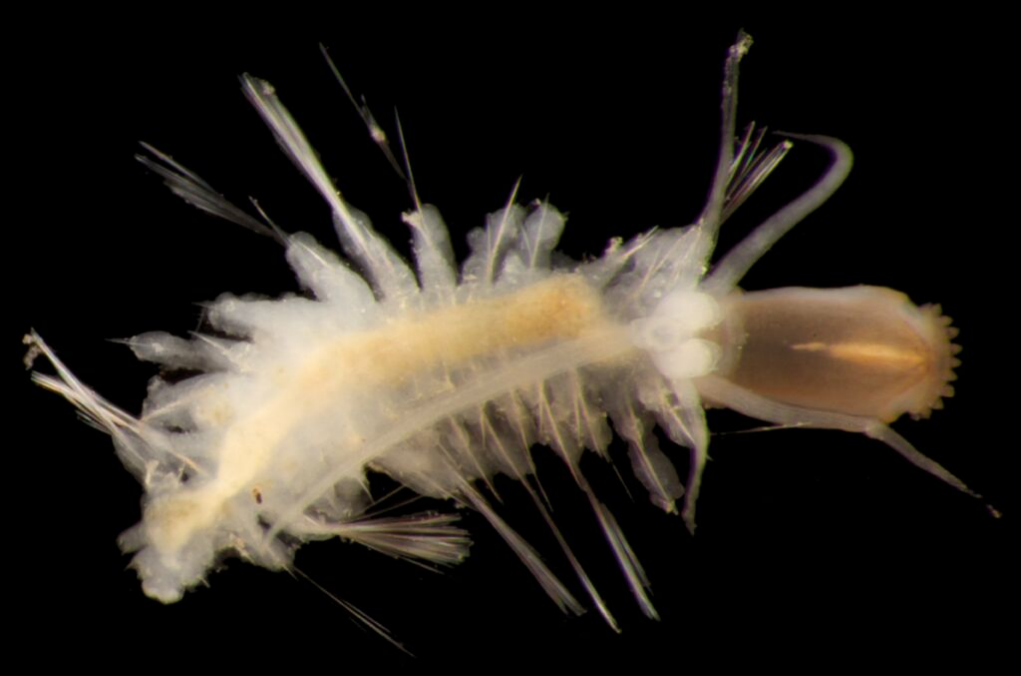
Polynoidae sp. (NHM_128), complete live specimen NHM_1925 in dorsal view.

Specimens (Fig. 83) consistent with placement within family Polynoidae, based on morphology and DNA. Genetically matches with *Macellicephala* sp. 308 as identified in Bonifácio et al. (2021).

#### Polynoidae sp. (NHM_583)

E720C7CB-99A0-568F-B1F0-41635586F656

##### Materials

- **Type status:** Other material. **Occurrence:** catalogNumber: NHMUK ANEA 2023.513; recordNumber: NHM_0733; recordedBy: Adrian Glover | Helena Wiklund | Thomas Dahlgren | Madeleine Brasier; individualCount: 1; preparations: specimen stored in 80% non-denatured ethanol aqueous solution | DNA voucher stored in buffer; otherCatalogNumbers: 0174127333; associatedSequences: OQ746544 (16S) | OQ746852 (18S); occurrenceID: FF061E25-F3E0-5EAF-AB22-19EF7145A6C3; **Taxon:** taxonConceptID: Polynoidae sp. (NHM_583); scientificName: Polynoidae; kingdom: Animalia; phylum: Annelida; class: Polychaeta; order: Phyllodocida; family: Polynoidae; taxonRank: family; scientificNameAuthorship: Kinberg, 1856; **Location:** waterBody: Pacific; stateProvince: Clarion Clipperton Zone; locality: UK Seabed Resources Ltd exploration area UK-1 Stratum B; verbatimLocality: UK1 Stratum B; maximumDepthInMeters: 4425; locationRemarks: Deployment EB02; at Station U5; from R/V Thomas G. Thompson Cruise no. TN319; verbatimLatitude: 12'32.23; verbatimLongitude: 116'36.25; decimalLatitude: 12.53717; decimalLongitude: -116.60417; geodeticDatum: WGS84; **Identification:** identifiedBy: Helena Wiklund | Lenka Neal | Thomas Dahlgren | Adrian Glover | Madeleine Brasier | Regan Drennan | Eva Stewart; dateIdentified: 2021-04-20; identificationRemarks: identified by DNA and morphology; **Event:** eventID: UK1_AB02_EB02; samplingProtocol: Brenke Epibenthic Sledge; eventDate: 2015-02-20; eventTime: 06:24; habitat: Abyssal plain; fieldNotes: Collected from epi net (on the epibenthic sledge); **Record Level:** language: en; institutionCode: NHMUK; collectionCode: ZOO; datasetName: ABYSSLINE; basisOfRecord: PreservedSpecimen
- **Type status:** Other material. **Occurrence:** catalogNumber: NHMUK ANEA 2023.512; recordNumber: NHM_0583; recordedBy: Adrian Glover | Helena Wiklund | Thomas Dahlgren | Madeleine Brasier; individualCount: 1; preparations: specimen stored in 80% non-denatured ethanol aqueous solution | DNA voucher stored in buffer; otherCatalogNumbers: 0174126600; associatedSequences: OQ746529 (16S) | OQ738527 (COI); occurrenceID: 6FE7C516-7CFD-514F-93B4-E499ABBC5C7A; **Taxon:** taxonConceptID: Polynoidae sp. (NHM_583); scientificName: Polynoidae; kingdom: Animalia; phylum: Annelida; class: Polychaeta; order: Phyllodocida; family: Polynoidae; taxonRank: family; scientificNameAuthorship: Kinberg, 1856; **Location:** waterBody: Pacific; stateProvince: Clarion Clipperton Zone; locality: UK Seabed Resources Ltd exploration area UK-1 Stratum B; verbatimLocality: UK1 Stratum B; maximumDepthInMeters: 4202; locationRemarks: Deployment EB01; at Station U2; from R/V Thomas G. Thompson Cruise no. TN319; verbatimLatitude: 12'23.17456; verbatimLongitude: 116'32.92021; decimalLatitude: 12.38624; decimalLongitude: -116.54867; geodeticDatum: WGS84; **Identification:** identifiedBy: Helena Wiklund | Lenka Neal | Thomas Dahlgren | Adrian Glover | Madeleine Brasier | Regan Drennan | Eva Stewart; dateIdentified: 2021-04-20; identificationRemarks: identified by DNA and morphology; **Event:** eventID: UK1_AB02_EB01; samplingProtocol: Brenke Epibenthic Sledge; eventDate: 2015-02-17; eventTime: 05:15; habitat: Abyssal plain; fieldNotes: Collected from epi net (on the epibenthic sledge); **Record Level:** language: en; institutionCode: NHMUK; collectionCode: ZOO; datasetName: ABYSSLINE; basisOfRecord: PreservedSpecimen

##### Distribution

Eastern Clarion-Clipperton Zone, central Pacific Ocean.

##### Diagnosis

**Figure 84.**
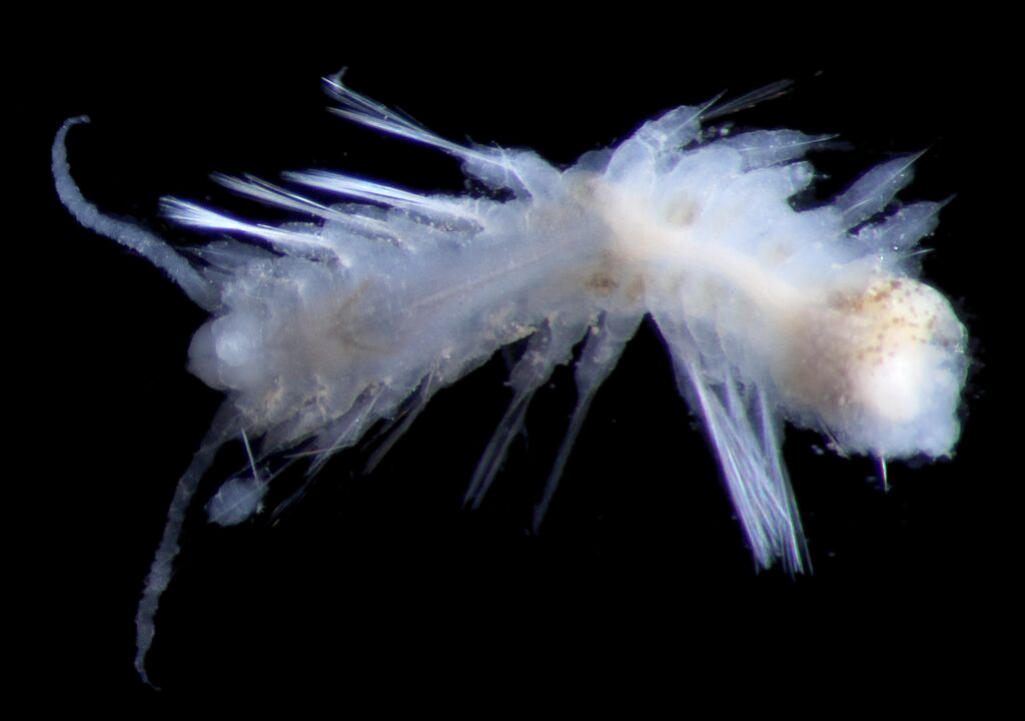
Polynoidae sp. (NHM_583), complete live specimen NHM_733 in ventral view.

Damaged specimens (Fig. 84) consistent with placement within family Polynoidae, based on morphology and DNA. Genetically matches with *Macellicephala* sp. 180 as identified in Bonifácio et al. (2021).

#### Polynoidae sp. (NHM_588)

348EF831-295F-500B-AD12-51399BF883BC

##### Materials

- **Type status:** Other material. **Occurrence:** catalogNumber: NHMUK ANEA 2023.538; recordNumber: NHM_0957B; recordedBy: Adrian Glover | Helena Wiklund | Thomas Dahlgren | Madeleine Brasier; individualCount: 1; preparations: specimen stored in 80% non-denatured ethanol aqueous solution | DNA voucher stored in buffer; otherCatalogNumbers: 0174126606; associatedSequences: OQ746598 (16S); occurrenceID: ACA236E6-D6A8-50B5-B473-E2B1A88B9003; **Taxon:** taxonConceptID: Polynoidae sp. (NHM_588); scientificName: Polynoidae; kingdom: Animalia; phylum: Annelida; class: Polychaeta; order: Phyllodocida; family: Polynoidae; taxonRank: family; scientificNameAuthorship: Kinberg, 1856; **Location:** waterBody: Pacific; stateProvince: Clarion Clipperton Zone; locality: UK Seabed Resources Ltd exploration area UK-1 Stratum B; verbatimLocality: UK1 Stratum B; maximumDepthInMeters: 4198; locationRemarks: Deployment EB03; at Station U4; from R/V Thomas G. Thompson Cruise no. TN319; verbatimLatitude: 12'34.28; verbatimLongitude: 116'36.63; decimalLatitude: 12.57133; decimalLongitude: -116.6105; geodeticDatum: WGS84; **Identification:** identifiedBy: Helena Wiklund | Lenka Neal | Thomas Dahlgren | Adrian Glover | Madeleine Brasier | Regan Drennan | Eva Stewart; dateIdentified: 2021-04-20; identificationRemarks: identified by DNA and morphology; **Event:** eventID: UK1_AB02_EB03; samplingProtocol: Brenke Epibenthic Sledge; eventDate: 2015-02-23; eventTime: 05:39; habitat: Abyssal plain; fieldNotes: Collected from epi net (on the epibenthic sledge); **Record Level:** language: en; institutionCode: NHMUK; collectionCode: ZOO; datasetName: ABYSSLINE; basisOfRecord: PreservedSpecimen
- **Type status:** Other material. **Occurrence:** catalogNumber: NHMUK ANEA 2023.528; recordNumber: NHM_0588; recordedBy: Adrian Glover | Helena Wiklund | Thomas Dahlgren | Madeleine Brasier; individualCount: 1; preparations: specimen stored in 80% non-denatured ethanol aqueous solution | DNA voucher stored in buffer; otherCatalogNumbers: 0174126576; associatedSequences: OQ746531 (16S) | OQ746845 (18S); occurrenceID: BBEB5812-F05A-542C-AA6E-9EAA55F84DA9; **Taxon:** taxonConceptID: Polynoidae sp. (NHM_588); scientificName: Polynoidae; kingdom: Animalia; phylum: Annelida; class: Polychaeta; order: Phyllodocida; family: Polynoidae; taxonRank: family; scientificNameAuthorship: Kinberg, 1856; **Location:** waterBody: Pacific; stateProvince: Clarion Clipperton Zone; locality: UK Seabed Resources Ltd exploration area UK-1 Stratum B; verbatimLocality: UK1 Stratum B; maximumDepthInMeters: 4202; locationRemarks: Deployment EB01; at Station U2; from R/V Thomas G. Thompson Cruise no. TN319; verbatimLatitude: 12'23.17456; verbatimLongitude: 116'32.92021; decimalLatitude: 12.38624; decimalLongitude: -116.54867; geodeticDatum: WGS84; **Identification:** identifiedBy: Helena Wiklund | Lenka Neal | Thomas Dahlgren | Adrian Glover | Madeleine Brasier | Regan Drennan | Eva Stewart; dateIdentified: 2021-04-20; identificationRemarks: identified by DNA and morphology; **Event:** eventID: UK1_AB02_EB01; samplingProtocol: Brenke Epibenthic Sledge; eventDate: 2015-02-17; eventTime: 05:15; habitat: Abyssal plain; fieldNotes: Collected from epi net (on the epibenthic sledge); **Record Level:** language: en; institutionCode: NHMUK; collectionCode: ZOO; datasetName: ABYSSLINE; basisOfRecord: PreservedSpecimen

##### Distribution

Eastern Clarion-Clipperton Zone, central Pacific Ocean.

##### Diagnosis

**Figure 85.**
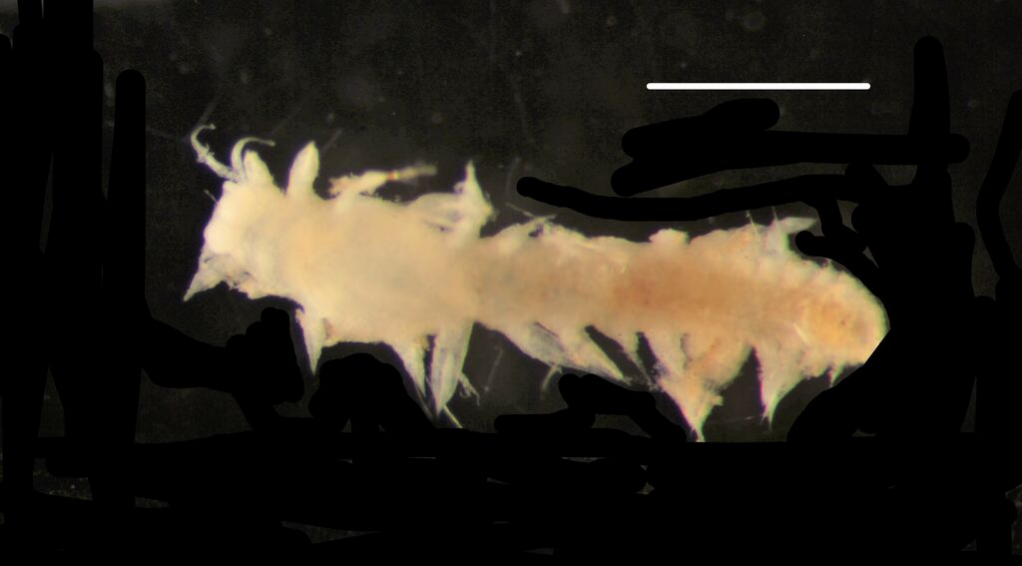
Polynoidae sp. (NHM_588), damaged live specimen NHM_957B in dorsal view. Scale bar 1 mm.

Damaged specimens (Fig. 85) consistent with placement within family Polynoidae, based on morphology and DNA. Genetically matches with *Polaruschakov* sp. 615 as identified in Bonifácio et al. (2021).

#### Polynoidae sp. (NHM_595)

BB25C47D-1882-5F9C-B0D6-77808C9DDEB2

##### Materials

- **Type status:** Other material. **Occurrence:** catalogNumber: NHMUK ANEA 2023.489; recordNumber: NHM_1337; recordedBy: Adrian Glover | Helena Wiklund | Thomas Dahlgren | Madeleine Brasier; individualCount: 1; preparations: specimen stored in 80% non-denatured ethanol aqueous solution | DNA voucher stored in buffer; otherCatalogNumbers: 0174127329; associatedSequences: OQ746661 (16S) | OQ746889 (18S); occurrenceID: 4C1BA9A6-B0FC-5A4D-BAAF-684DE1290A1D; **Taxon:** taxonConceptID: Polynoidae sp. (NHM_595); scientificName: Polynoidae; kingdom: Animalia; phylum: Annelida; class: Polychaeta; order: Phyllodocida; family: Polynoidae; taxonRank: family; scientificNameAuthorship: Kinberg, 1856; **Location:** waterBody: Pacific; stateProvince: Clarion Clipperton Zone; locality: Ocean Mineral Singapore exploration claim Stratum A; verbatimLocality: OMS Stratum A; maximumDepthInMeters: 4302; locationRemarks: Deployment EB06; at Station S5; from R/V Thomas G. Thompson Cruise no. TN319; verbatimLatitude: 12'15.44; verbatimLongitude: 117'18.13; decimalLatitude: 12.25733; decimalLongitude: -117.30217; geodeticDatum: WGS84; **Identification:** identifiedBy: Helena Wiklund | Lenka Neal | Thomas Dahlgren | Adrian Glover | Madeleine Brasier | Regan Drennan | Eva Stewart; dateIdentified: 2021-04-20; identificationRemarks: identified by DNA and morphology; **Event:** eventID: OMS1_AB02_EB06; samplingProtocol: Brenke Epibenthic Sledge; eventDate: 2015-03-01; eventTime: 04:02; habitat: Abyssal plain; fieldNotes: Collected from epi net (on the epibenthic sledge); **Record Level:** language: en; institutionCode: NHMUK; collectionCode: ZOO; datasetName: ABYSSLINE; basisOfRecord: PreservedSpecimen
- **Type status:** Other material. **Occurrence:** catalogNumber: NHMUK ANEA 2023.491; recordNumber: NHM_2284; recordedBy: Adrian Glover | Helena Wiklund | Thomas Dahlgren | Madeleine Brasier; individualCount: 1; preparations: specimen stored in 80% non-denatured ethanol aqueous solution | DNA voucher stored in buffer; otherCatalogNumbers: 0174126154; associatedSequences: OQ746769 (16S); occurrenceID: A4D7A14C-91C7-5E8C-BA38-44DA3BFAE371; **Taxon:** taxonConceptID: Polynoidae sp. (NHM_595); scientificName: Polynoidae; kingdom: Animalia; phylum: Annelida; class: Polychaeta; order: Phyllodocida; family: Polynoidae; taxonRank: family; scientificNameAuthorship: Kinberg, 1856; **Location:** waterBody: Pacific; stateProvince: Clarion Clipperton Zone; locality: Ocean Mineral Singapore exploration claim Stratum A; verbatimLocality: OMS Stratum A; maximumDepthInMeters: 4302; locationRemarks: Deployment EB06; at Station S5; from R/V Thomas G. Thompson Cruise no. TN319; verbatimLatitude: 12'15.44; verbatimLongitude: 117'18.13; decimalLatitude: 12.25733; decimalLongitude: -117.30217; geodeticDatum: WGS84; **Identification:** identifiedBy: Helena Wiklund | Lenka Neal | Thomas Dahlgren | Adrian Glover | Madeleine Brasier | Regan Drennan | Eva Stewart; dateIdentified: 2021-04-20; identificationRemarks: identified by DNA and morphology; **Event:** eventID: OMS1_AB02_EB06; samplingProtocol: Brenke Epibenthic Sledge; eventDate: 2015-03-01; eventTime: 04:02; habitat: Abyssal plain; fieldNotes: Collected from supra net (on the epibenthic sledge); **Record Level:** language: en; institutionCode: NHMUK; collectionCode: ZOO; datasetName: ABYSSLINE; basisOfRecord: PreservedSpecimen
- **Type status:** Other material. **Occurrence:** catalogNumber: NHMUK ANEA 2023.486; recordNumber: NHM_0915A; recordedBy: Adrian Glover | Helena Wiklund | Thomas Dahlgren | Madeleine Brasier; individualCount: 1; preparations: specimen stored in 80% non-denatured ethanol aqueous solution | DNA voucher stored in buffer; otherCatalogNumbers: 0174126595; associatedSequences: OQ746578 (16S); occurrenceID: 6197A875-08EC-5549-BB6E-BF9DDBB3AE27; **Taxon:** taxonConceptID: Polynoidae sp. (NHM_595); scientificName: Polynoidae; kingdom: Animalia; phylum: Annelida; class: Polychaeta; order: Phyllodocida; family: Polynoidae; taxonRank: family; scientificNameAuthorship: Kinberg, 1856; **Location:** waterBody: Pacific; stateProvince: Clarion Clipperton Zone; locality: UK Seabed Resources Ltd exploration area UK-1 Stratum B; verbatimLocality: UK1 Stratum B; maximumDepthInMeters: 4198; locationRemarks: Deployment EB03; at Station U4; from R/V Thomas G. Thompson Cruise no. TN319; verbatimLatitude: 12'34.28; verbatimLongitude: 116'36.63; decimalLatitude: 12.57133; decimalLongitude: -116.6105; geodeticDatum: WGS84; **Identification:** identifiedBy: Helena Wiklund | Lenka Neal | Thomas Dahlgren | Adrian Glover | Madeleine Brasier | Regan Drennan | Eva Stewart; dateIdentified: 2021-04-20; identificationRemarks: identified by DNA and morphology; **Event:** eventID: UK1_AB02_EB03; samplingProtocol: Brenke Epibenthic Sledge; eventDate: 2015-02-23; eventTime: 05:39; habitat: Abyssal plain; fieldNotes: Collected from epi net (on the epibenthic sledge); **Record Level:** language: en; institutionCode: NHMUK; collectionCode: ZOO; datasetName: ABYSSLINE; basisOfRecord: PreservedSpecimen
- **Type status:** Other material. **Occurrence:** catalogNumber: NHMUK ANEA 2023.485; recordNumber: NHM_0915F; recordedBy: Adrian Glover | Helena Wiklund | Thomas Dahlgren | Madeleine Brasier; individualCount: 1; preparations: specimen stored in 80% non-denatured ethanol aqueous solution | DNA voucher stored in buffer; otherCatalogNumbers: 0174126556; associatedSequences: OQ746581 (16S); occurrenceID: BE9D8E93-FD20-597E-9913-1B3A519D9966; **Taxon:** taxonConceptID: Polynoidae sp. (NHM_595); scientificName: Polynoidae; kingdom: Animalia; phylum: Annelida; class: Polychaeta; order: Phyllodocida; family: Polynoidae; taxonRank: family; scientificNameAuthorship: Kinberg, 1856; **Location:** waterBody: Pacific; stateProvince: Clarion Clipperton Zone; locality: UK Seabed Resources Ltd exploration area UK-1 Stratum B; verbatimLocality: UK1 Stratum B; maximumDepthInMeters: 4198; locationRemarks: Deployment EB03; at Station U4; from R/V Thomas G. Thompson Cruise no. TN319; verbatimLatitude: 12'34.28; verbatimLongitude: 116'36.63; decimalLatitude: 12.57133; decimalLongitude: -116.6105; geodeticDatum: WGS84; **Identification:** identifiedBy: Helena Wiklund | Lenka Neal | Thomas Dahlgren | Adrian Glover | Madeleine Brasier | Regan Drennan | Eva Stewart; dateIdentified: 2021-04-20; identificationRemarks: identified by DNA and morphology; **Event:** eventID: UK1_AB02_EB03; samplingProtocol: Brenke Epibenthic Sledge; eventDate: 2015-02-23; eventTime: 05:39; habitat: Abyssal plain; fieldNotes: Collected from epi net (on the epibenthic sledge); **Record Level:** language: en; institutionCode: NHMUK; collectionCode: ZOO; datasetName: ABYSSLINE; basisOfRecord: PreservedSpecimen
- **Type status:** Other material. **Occurrence:** catalogNumber: NHMUK ANEA 2023.488; recordNumber: NHM_0917A; recordedBy: Adrian Glover | Helena Wiklund | Thomas Dahlgren | Madeleine Brasier; individualCount: 1; preparations: specimen stored in 80% non-denatured ethanol aqueous solution | DNA voucher stored in buffer; otherCatalogNumbers: 0174126605; associatedSequences: OQ746585 (16S); occurrenceID: 6AF97FA5-847C-505B-9BB2-B9FA886DE098; **Taxon:** taxonConceptID: Polynoidae sp. (NHM_595); scientificName: Polynoidae; kingdom: Animalia; phylum: Annelida; class: Polychaeta; order: Phyllodocida; family: Polynoidae; taxonRank: family; scientificNameAuthorship: Kinberg, 1856; **Location:** waterBody: Pacific; stateProvince: Clarion Clipperton Zone; locality: UK Seabed Resources Ltd exploration area UK-1 Stratum B; verbatimLocality: UK1 Stratum B; maximumDepthInMeters: 4198; locationRemarks: Deployment EB03; at Station U4; from R/V Thomas G. Thompson Cruise no. TN319; verbatimLatitude: 12'34.28; verbatimLongitude: 116'36.63; decimalLatitude: 12.57133; decimalLongitude: -116.6105; geodeticDatum: WGS84; **Identification:** identifiedBy: Helena Wiklund | Lenka Neal | Thomas Dahlgren | Adrian Glover | Madeleine Brasier | Regan Drennan | Eva Stewart; dateIdentified: 2021-04-20; identificationRemarks: identified by DNA and morphology; **Event:** eventID: UK1_AB02_EB03; samplingProtocol: Brenke Epibenthic Sledge; eventDate: 2015-02-23; eventTime: 05:39; habitat: Abyssal plain; fieldNotes: Collected from epi net (on the epibenthic sledge); **Record Level:** language: en; institutionCode: NHMUK; collectionCode: ZOO; datasetName: ABYSSLINE; basisOfRecord: PreservedSpecimen
- **Type status:** Other material. **Occurrence:** catalogNumber: NHMUK ANEA 2023.487; recordNumber: NHM_0917C; recordedBy: Adrian Glover | Helena Wiklund | Thomas Dahlgren | Madeleine Brasier; individualCount: 1; preparations: specimen stored in 80% non-denatured ethanol aqueous solution | DNA voucher stored in buffer; otherCatalogNumbers: 0174126594; associatedSequences: OQ746586 (16S); occurrenceID: 9C37D81A-F741-5E8C-8341-510D7C6404D0; **Taxon:** taxonConceptID: Polynoidae sp. (NHM_595); scientificName: Polynoidae; kingdom: Animalia; phylum: Annelida; class: Polychaeta; order: Phyllodocida; family: Polynoidae; taxonRank: family; scientificNameAuthorship: Kinberg, 1856; **Location:** waterBody: Pacific; stateProvince: Clarion Clipperton Zone; locality: UK Seabed Resources Ltd exploration area UK-1 Stratum B; verbatimLocality: UK1 Stratum B; maximumDepthInMeters: 4198; locationRemarks: Deployment EB03; at Station U4; from R/V Thomas G. Thompson Cruise no. TN319; verbatimLatitude: 12'34.28; verbatimLongitude: 116'36.63; decimalLatitude: 12.57133; decimalLongitude: -116.6105; geodeticDatum: WGS84; **Identification:** identifiedBy: Helena Wiklund | Lenka Neal | Thomas Dahlgren | Adrian Glover | Madeleine Brasier | Regan Drennan | Eva Stewart; dateIdentified: 2021-04-20; identificationRemarks: identified by DNA and morphology; **Event:** eventID: UK1_AB02_EB03; samplingProtocol: Brenke Epibenthic Sledge; eventDate: 2015-02-23; eventTime: 05:39; habitat: Abyssal plain; fieldNotes: Collected from epi net (on the epibenthic sledge); **Record Level:** language: en; institutionCode: NHMUK; collectionCode: ZOO; datasetName: ABYSSLINE; basisOfRecord: PreservedSpecimen
- **Type status:** Other material. **Occurrence:** catalogNumber: NHMUK ANEA 2023.484; recordNumber: NHM_0595; recordedBy: Adrian Glover | Helena Wiklund | Thomas Dahlgren | Madeleine Brasier; individualCount: 1; preparations: specimen stored in 80% non-denatured ethanol aqueous solution | DNA voucher stored in buffer; otherCatalogNumbers: 0174126528; associatedSequences: OQ746534 (16S); occurrenceID: D58926FF-8AA9-5590-8012-5D6B8398FCC8; **Taxon:** taxonConceptID: Polynoidae sp. (NHM_595); scientificName: Polynoidae; kingdom: Animalia; phylum: Annelida; class: Polychaeta; order: Phyllodocida; family: Polynoidae; taxonRank: family; scientificNameAuthorship: Kinberg, 1856; **Location:** waterBody: Pacific; stateProvince: Clarion Clipperton Zone; locality: UK Seabed Resources Ltd exploration area UK-1 Stratum B; verbatimLocality: UK1 Stratum B; maximumDepthInMeters: 4202; locationRemarks: Deployment EB01; at Station U2; from R/V Thomas G. Thompson Cruise no. TN319; verbatimLatitude: 12'23.17456; verbatimLongitude: 116'32.92021; decimalLatitude: 12.38624; decimalLongitude: -116.54867; geodeticDatum: WGS84; **Identification:** identifiedBy: Helena Wiklund | Lenka Neal | Thomas Dahlgren | Adrian Glover | Madeleine Brasier | Regan Drennan | Eva Stewart; dateIdentified: 2021-04-20; identificationRemarks: identified by DNA and morphology; **Event:** eventID: UK1_AB02_EB01; samplingProtocol: Brenke Epibenthic Sledge; eventDate: 2015-02-17; eventTime: 05:15; habitat: Abyssal plain; fieldNotes: Collected from epi net (on the epibenthic sledge); **Record Level:** language: en; institutionCode: NHMUK; collectionCode: ZOO; datasetName: ABYSSLINE; basisOfRecord: PreservedSpecimen
- **Type status:** Other material. **Occurrence:** catalogNumber: NHMUK ANEA 2023.490; recordNumber: NHM_1519; recordedBy: Adrian Glover | Helena Wiklund | Thomas Dahlgren | Madeleine Brasier; individualCount: 1; preparations: specimen stored in 80% non-denatured ethanol aqueous solution | DNA voucher stored in buffer; otherCatalogNumbers: 0174126764; associatedSequences: OQ746691 (16S) | OQ738593 (COI); occurrenceID: 6588A7D3-6A66-539C-855B-AB2B55AABC0D; **Taxon:** taxonConceptID: Polynoidae sp. (NHM_595); scientificName: Polynoidae; kingdom: Animalia; phylum: Annelida; class: Polychaeta; order: Phyllodocida; family: Polynoidae; taxonRank: family; scientificNameAuthorship: Kinberg, 1856; **Location:** waterBody: Pacific; stateProvince: Clarion Clipperton Zone; locality: UK Seabed Resources Ltd exploration area UK-1 Stratum B; verbatimLocality: UK1 Stratum B; maximumDepthInMeters: 4252; locationRemarks: Deployment EB08; at Station U11; from R/V Thomas G. Thompson Cruise no. TN319; verbatimLatitude: 12'30.79; verbatimLongitude: 116'29.48; decimalLatitude: 12.51317; decimalLongitude: -116.49133; geodeticDatum: WGS84; **Identification:** identifiedBy: Helena Wiklund | Lenka Neal | Thomas Dahlgren | Adrian Glover | Madeleine Brasier | Regan Drennan | Eva Stewart; dateIdentified: 2021-04-20; identificationRemarks: identified by DNA and morphology; **Event:** eventID: UK1_AB02_EB08; samplingProtocol: Brenke Epibenthic Sledge; eventDate: 2015-03-05; eventTime: 18:53; habitat: Abyssal plain; fieldNotes: Collected from epi net (on the epibenthic sledge); **Record Level:** language: en; institutionCode: NHMUK; collectionCode: ZOO; datasetName: ABYSSLINE; basisOfRecord: PreservedSpecimen

##### Distribution

Eastern Clarion-Clipperton Zone, central Pacific Ocean.

##### Diagnosis

**Figure 86.**
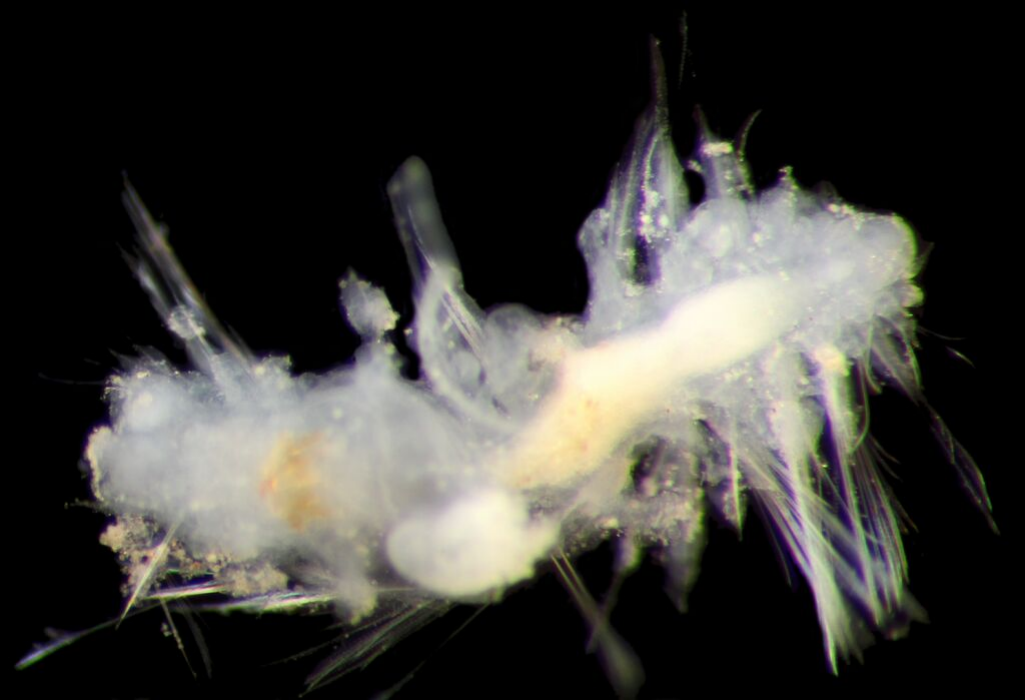
Polynoidae sp. (NHM_595), damaged live specimen NHM_595 on dorsal view.

Damaged specimens (Fig. 86) consistent with placement within family Polynoidae, based on morphology and DNA. Genetically matches with *Bathyfauvelia* sp. 225 as identified in Bonifácio et al. (2021).

#### Polynoidae sp. (NHM_679)

8BF34A73-E7C7-5CB8-B40A-4CB57433BADD

##### Materials

- **Type status:** Other material. **Occurrence:** catalogNumber: NHMUK ANEA 2023.495; recordNumber: NHM_1248; recordedBy: Adrian Glover | Helena Wiklund | Thomas Dahlgren | Madeleine Brasier; individualCount: 1; preparations: specimen stored in 80% non-denatured ethanol aqueous solution | DNA voucher stored in buffer; otherCatalogNumbers: 0174127305; associatedSequences: OQ746645 (16S); occurrenceID: 76D1553A-2216-5742-92CA-EBFDFA6033ED; **Taxon:** taxonConceptID: Polynoidae sp. (NHM_679); scientificName: Polynoidae; kingdom: Animalia; phylum: Annelida; class: Polychaeta; order: Phyllodocida; family: Polynoidae; taxonRank: family; scientificNameAuthorship: Kinberg, 1856; **Location:** waterBody: Pacific; stateProvince: Clarion Clipperton Zone; locality: Ocean Mineral Singapore exploration claim Stratum A; verbatimLocality: OMS Stratum A; maximumDepthInMeters: 4302; locationRemarks: Deployment EB06; at Station S5; from R/V Thomas G. Thompson Cruise no. TN319; verbatimLatitude: 12'15.44; verbatimLongitude: 117'18.13; decimalLatitude: 12.25733; decimalLongitude: -117.30217; geodeticDatum: WGS84; **Identification:** identifiedBy: Helena Wiklund | Lenka Neal | Thomas Dahlgren | Adrian Glover | Madeleine Brasier | Regan Drennan | Eva Stewart; dateIdentified: 2021-04-20; identificationRemarks: identified by DNA and morphology; **Event:** eventID: OMS1_AB02_EB06; samplingProtocol: Brenke Epibenthic Sledge; eventDate: 2015-03-01; eventTime: 04:02; habitat: Abyssal plain; fieldNotes: Collected from epi net (on the epibenthic sledge); **Record Level:** language: en; institutionCode: NHMUK; collectionCode: ZOO; datasetName: ABYSSLINE; basisOfRecord: PreservedSpecimen
- **Type status:** Other material. **Occurrence:** catalogNumber: NHMUK ANEA 2023.496; recordNumber: NHM_1346; recordedBy: Adrian Glover | Helena Wiklund | Thomas Dahlgren | Madeleine Brasier; individualCount: 1; preparations: specimen stored in 80% non-denatured ethanol aqueous solution | DNA voucher stored in buffer; otherCatalogNumbers: 0174127307; associatedSequences: OQ746664 (16S); occurrenceID: 1BF4C60A-BFA7-53E4-B0A2-A5F8A45454B2; **Taxon:** taxonConceptID: Polynoidae sp. (NHM_679); scientificName: Polynoidae; kingdom: Animalia; phylum: Annelida; class: Polychaeta; order: Phyllodocida; family: Polynoidae; taxonRank: family; scientificNameAuthorship: Kinberg, 1856; **Location:** waterBody: Pacific; stateProvince: Clarion Clipperton Zone; locality: Ocean Mineral Singapore exploration claim Stratum A; verbatimLocality: OMS Stratum A; maximumDepthInMeters: 4302; locationRemarks: Deployment EB06; at Station S5; from R/V Thomas G. Thompson Cruise no. TN319; verbatimLatitude: 12'15.44; verbatimLongitude: 117'18.13; decimalLatitude: 12.25733; decimalLongitude: -117.30217; geodeticDatum: WGS84; **Identification:** identifiedBy: Helena Wiklund | Lenka Neal | Thomas Dahlgren | Adrian Glover | Madeleine Brasier | Regan Drennan | Eva Stewart; dateIdentified: 2021-04-20; identificationRemarks: identified by DNA and morphology; **Event:** eventID: OMS1_AB02_EB06; samplingProtocol: Brenke Epibenthic Sledge; eventDate: 2015-03-01; eventTime: 04:02; habitat: Abyssal plain; fieldNotes: Collected from epi net (on the epibenthic sledge); **Record Level:** language: en; institutionCode: NHMUK; collectionCode: ZOO; datasetName: ABYSSLINE; basisOfRecord: PreservedSpecimen
- **Type status:** Other material. **Occurrence:** catalogNumber: NHMUK ANEA 2023.499; recordNumber: NHM_2562; recordedBy: Adrian Glover | Helena Wiklund | Thomas Dahlgren | Madeleine Brasier; individualCount: 1; preparations: specimen stored in 80% non-denatured ethanol aqueous solution | DNA voucher stored in buffer; otherCatalogNumbers: 0174126227; associatedSequences: OQ746780 (16S); occurrenceID: 252C34AB-EDA6-526D-80DB-6275E27109EB; **Taxon:** taxonConceptID: Polynoidae sp. (NHM_679); scientificName: Polynoidae; kingdom: Animalia; phylum: Annelida; class: Polychaeta; order: Phyllodocida; family: Polynoidae; taxonRank: family; scientificNameAuthorship: Kinberg, 1856; **Location:** waterBody: Pacific; stateProvince: Clarion Clipperton Zone; locality: Ocean Mineral Singapore exploration claim Stratum A; verbatimLocality: OMS Stratum A; maximumDepthInMeters: 4302; locationRemarks: Deployment EB06; at Station S5; from R/V Thomas G. Thompson Cruise no. TN319; verbatimLatitude: 12'15.44; verbatimLongitude: 117'18.13; decimalLatitude: 12.25733; decimalLongitude: -117.30217; geodeticDatum: WGS84; **Identification:** identifiedBy: Helena Wiklund | Lenka Neal | Thomas Dahlgren | Adrian Glover | Madeleine Brasier | Regan Drennan | Eva Stewart; dateIdentified: 2021-04-20; identificationRemarks: identified by DNA and morphology; **Event:** eventID: OMS1_AB02_EB06; samplingProtocol: Brenke Epibenthic Sledge; eventDate: 2015-03-01; eventTime: 04:02; habitat: Abyssal plain; fieldNotes: Collected from supra net (on the epibenthic sledge); **Record Level:** language: en; institutionCode: NHMUK; collectionCode: ZOO; datasetName: ABYSSLINE; basisOfRecord: PreservedSpecimen
- **Type status:** Other material. **Occurrence:** catalogNumber: NHMUK ANEA 2023.498; recordNumber: NHM_2344; recordedBy: Adrian Glover | Helena Wiklund | Thomas Dahlgren | Madeleine Brasier; individualCount: 1; preparations: specimen stored in 80% non-denatured ethanol aqueous solution | DNA voucher stored in buffer; otherCatalogNumbers: 0174126155; associatedSequences: OQ746772 (16S); occurrenceID: 629D9683-8A5D-5AD0-9ABF-08C8F99DC9F6; **Taxon:** taxonConceptID: Polynoidae sp. (NHM_679); scientificName: Polynoidae; kingdom: Animalia; phylum: Annelida; class: Polychaeta; order: Phyllodocida; family: Polynoidae; taxonRank: family; scientificNameAuthorship: Kinberg, 1856; **Location:** waterBody: Pacific; stateProvince: Clarion Clipperton Zone; locality: Ocean Mineral Singapore exploration claim Stratum A; verbatimLocality: OMS Stratum A; maximumDepthInMeters: 4100; locationRemarks: Deployment EB05; at Station S2; from R/V Thomas G. Thompson Cruise no. TN319; verbatimLatitude: 12'06.93; verbatimLongitude: 117'09.87; decimalLatitude: 12.1155; decimalLongitude: -117.1645; geodeticDatum: WGS84; **Identification:** identifiedBy: Helena Wiklund | Lenka Neal | Thomas Dahlgren | Adrian Glover | Madeleine Brasier | Regan Drennan | Eva Stewart; dateIdentified: 2021-04-20; identificationRemarks: identified by DNA and morphology; **Event:** eventID: OMS1_AB02_EB05; samplingProtocol: Brenke Epibenthic Sledge; eventDate: 2015-02-26; eventTime: 21:29; habitat: Abyssal plain; fieldNotes: Collected from supra net (on the epibenthic sledge); **Record Level:** language: en; institutionCode: NHMUK; collectionCode: ZOO; datasetName: ABYSSLINE; basisOfRecord: PreservedSpecimen
- **Type status:** Other material. **Occurrence:** catalogNumber: NHMUK ANEA 2023.493; recordNumber: NHM_0878; recordedBy: Adrian Glover | Helena Wiklund | Thomas Dahlgren | Madeleine Brasier; individualCount: 1; preparations: specimen stored in 80% non-denatured ethanol aqueous solution | DNA voucher stored in buffer; otherCatalogNumbers: 0174127306; associatedSequences: OQ746570 (16S) | OQ738540 (COI); occurrenceID: 9049D108-1814-5151-803D-65DF5604A6BD; **Taxon:** taxonConceptID: Polynoidae sp. (NHM_679); scientificName: Polynoidae; kingdom: Animalia; phylum: Annelida; class: Polychaeta; order: Phyllodocida; family: Polynoidae; taxonRank: family; scientificNameAuthorship: Kinberg, 1856; **Location:** waterBody: Pacific; stateProvince: Clarion Clipperton Zone; locality: UK Seabed Resources Ltd exploration area UK-1 Stratum B; verbatimLocality: UK1 Stratum B; maximumDepthInMeters: 4198; locationRemarks: Deployment EB03; at Station U4; from R/V Thomas G. Thompson Cruise no. TN319; verbatimLatitude: 12'34.28; verbatimLongitude: 116'36.63; decimalLatitude: 12.57133; decimalLongitude: -116.6105; geodeticDatum: WGS84; **Identification:** identifiedBy: Helena Wiklund | Lenka Neal | Thomas Dahlgren | Adrian Glover | Madeleine Brasier | Regan Drennan | Eva Stewart; dateIdentified: 2021-04-20; identificationRemarks: identified by DNA and morphology; **Event:** eventID: UK1_AB02_EB03; samplingProtocol: Brenke Epibenthic Sledge; eventDate: 2015-02-23; eventTime: 05:39; habitat: Abyssal plain; fieldNotes: Collected from epi net (on the epibenthic sledge); **Record Level:** language: en; institutionCode: NHMUK; collectionCode: ZOO; datasetName: ABYSSLINE; basisOfRecord: PreservedSpecimen
- **Type status:** Other material. **Occurrence:** catalogNumber: NHMUK ANEA 2023.494; recordNumber: NHM_0913; recordedBy: Adrian Glover | Helena Wiklund | Thomas Dahlgren | Madeleine Brasier; individualCount: 1; preparations: specimen stored in 80% non-denatured ethanol aqueous solution | DNA voucher stored in buffer; otherCatalogNumbers: 0174127379; associatedSequences: OQ746577 (16S) | OQ738544 (COI); occurrenceID: 79011DF3-1842-5782-B7FB-AF8C22BFD2F8; **Taxon:** taxonConceptID: Polynoidae sp. (NHM_679); scientificName: Polynoidae; kingdom: Animalia; phylum: Annelida; class: Polychaeta; order: Phyllodocida; family: Polynoidae; taxonRank: family; scientificNameAuthorship: Kinberg, 1856; **Location:** waterBody: Pacific; stateProvince: Clarion Clipperton Zone; locality: UK Seabed Resources Ltd exploration area UK-1 Stratum B; verbatimLocality: UK1 Stratum B; maximumDepthInMeters: 4198; locationRemarks: Deployment EB03; at Station U4; from R/V Thomas G. Thompson Cruise no. TN319; verbatimLatitude: 12'34.28; verbatimLongitude: 116'36.63; decimalLatitude: 12.57133; decimalLongitude: -116.6105; geodeticDatum: WGS84; **Identification:** identifiedBy: Helena Wiklund | Lenka Neal | Thomas Dahlgren | Adrian Glover | Madeleine Brasier | Regan Drennan | Eva Stewart; dateIdentified: 2021-04-20; identificationRemarks: identified by DNA and morphology; **Event:** eventID: UK1_AB02_EB03; samplingProtocol: Brenke Epibenthic Sledge; eventDate: 2015-02-23; eventTime: 05:39; habitat: Abyssal plain; fieldNotes: Collected from epi net (on the epibenthic sledge); **Record Level:** language: en; institutionCode: NHMUK; collectionCode: ZOO; datasetName: ABYSSLINE; basisOfRecord: PreservedSpecimen
- **Type status:** Other material. **Occurrence:** catalogNumber: NHMUK ANEA 2023.492; recordNumber: NHM_0679; recordedBy: Adrian Glover | Helena Wiklund | Thomas Dahlgren | Madeleine Brasier; individualCount: 1; preparations: specimen stored in 80% non-denatured ethanol aqueous solution | DNA voucher stored in buffer; otherCatalogNumbers: 0174126574; associatedSequences: OQ746538 (16S) | OQ746850 (18S); occurrenceID: F35020FE-CC5A-59B4-927D-FD389571244A; **Taxon:** taxonConceptID: Polynoidae sp. (NHM_679); scientificName: Polynoidae; kingdom: Animalia; phylum: Annelida; class: Polychaeta; order: Phyllodocida; family: Polynoidae; taxonRank: family; scientificNameAuthorship: Kinberg, 1856; **Location:** waterBody: Pacific; stateProvince: Clarion Clipperton Zone; locality: UK Seabed Resources Ltd exploration area UK-1 Stratum B; verbatimLocality: UK1 Stratum B; maximumDepthInMeters: 4425; locationRemarks: Deployment EB02; at Station U5; from R/V Thomas G. Thompson Cruise no. TN319; verbatimLatitude: 12'32.23; verbatimLongitude: 116'36.25; decimalLatitude: 12.53717; decimalLongitude: -116.60417; geodeticDatum: WGS84; **Identification:** identifiedBy: Helena Wiklund | Lenka Neal | Thomas Dahlgren | Adrian Glover | Madeleine Brasier | Regan Drennan | Eva Stewart; dateIdentified: 2021-04-20; identificationRemarks: identified by DNA and morphology; **Event:** eventID: UK1_AB02_EB02; samplingProtocol: Brenke Epibenthic Sledge; eventDate: 2015-02-20; eventTime: 06:24; habitat: Abyssal plain; fieldNotes: Collected from epi net (on the epibenthic sledge); **Record Level:** language: en; institutionCode: NHMUK; collectionCode: ZOO; datasetName: ABYSSLINE; basisOfRecord: PreservedSpecimen
- **Type status:** Other material. **Occurrence:** catalogNumber: NHMUK ANEA 2023.497; recordNumber: NHM_1533D; recordedBy: Adrian Glover | Helena Wiklund | Thomas Dahlgren | Madeleine Brasier; individualCount: 1; preparations: specimen stored in 80% non-denatured ethanol aqueous solution | DNA voucher stored in buffer; otherCatalogNumbers: 0174126193; associatedSequences: OQ746693 (16S); occurrenceID: 4167E49E-FFF6-5368-A7F2-E619D15B6D04; **Taxon:** taxonConceptID: Polynoidae sp. (NHM_679); scientificName: Polynoidae; kingdom: Animalia; phylum: Annelida; class: Polychaeta; order: Phyllodocida; family: Polynoidae; taxonRank: family; scientificNameAuthorship: Kinberg, 1856; **Location:** waterBody: Pacific; stateProvince: Clarion Clipperton Zone; locality: UK Seabed Resources Ltd exploration area UK-1 Stratum B; verbatimLocality: UK1 Stratum B; maximumDepthInMeters: 4252; locationRemarks: Deployment EB08; at Station U11; from R/V Thomas G. Thompson Cruise no. TN319; verbatimLatitude: 12'30.79; verbatimLongitude: 116'29.48; decimalLatitude: 12.51317; decimalLongitude: -116.49133; geodeticDatum: WGS84; **Identification:** identifiedBy: Helena Wiklund | Lenka Neal | Thomas Dahlgren | Adrian Glover | Madeleine Brasier | Regan Drennan | Eva Stewart; dateIdentified: 2021-04-20; identificationRemarks: identified by DNA and morphology; **Event:** eventID: UK1_AB02_EB08; samplingProtocol: Brenke Epibenthic Sledge; eventDate: 2015-03-05; eventTime: 18:53; habitat: Abyssal plain; fieldNotes: Collected from epi net (on the epibenthic sledge); **Record Level:** language: en; institutionCode: NHMUK; collectionCode: ZOO; datasetName: ABYSSLINE; basisOfRecord: PreservedSpecimen

##### Distribution

Eastern Clarion-Clipperton Zone, central Pacific Ocean.

##### Diagnosis

**Figure 87.**
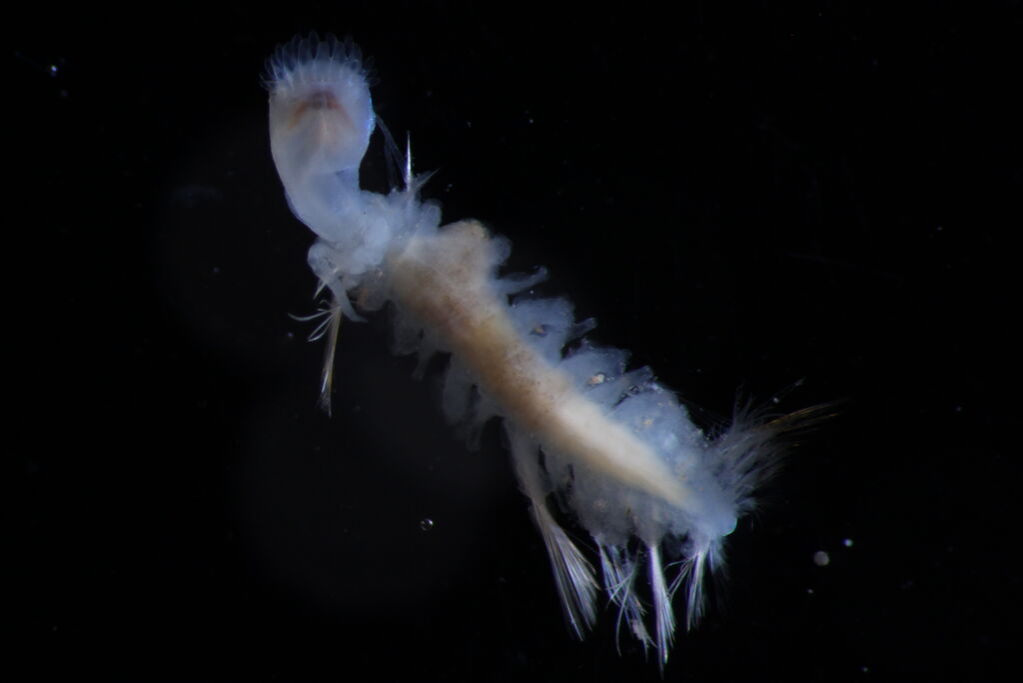
Polynoidae sp. (NHM_679), complete live specimen NHM_679 in dorsal view.

Specimens (Fig. 87) consistent with placement within family Polynoidae, based on morphology and DNA. Genetically matches with *Bathyfauvelia* sp. 224 as identified in Bonifácio et al. (2021).

#### Polynoidae sp. (NHM_747D)

7441644B-286B-591C-9F6F-3A54CFAC589E

##### Materials

- **Type status:** Other material. **Occurrence:** catalogNumber: NHMUK ANEA 2023.516; recordNumber: NHM_2266; recordedBy: Adrian Glover | Helena Wiklund | Thomas Dahlgren | Madeleine Brasier; individualCount: 1; preparations: specimen stored in 80% non-denatured ethanol aqueous solution | DNA voucher stored in buffer; otherCatalogNumbers: 0174126181; associatedSequences: OQ746767 (16S) | OQ738609 (COI); occurrenceID: A13E4020-D1E3-577C-B454-61C66A8E37BB; **Taxon:** taxonConceptID: Polynoidae sp. (NHM_747D); scientificName: Polynoidae; kingdom: Animalia; phylum: Annelida; class: Polychaeta; order: Phyllodocida; family: Polynoidae; taxonRank: family; scientificNameAuthorship: Kinberg, 1856; **Location:** waterBody: Pacific; stateProvince: Clarion Clipperton Zone; locality: Ocean Mineral Singapore exploration claim Stratum A; verbatimLocality: OMS Stratum A; maximumDepthInMeters: 4302; locationRemarks: Deployment EB06; at Station S5; from R/V Thomas G. Thompson Cruise no. TN319; verbatimLatitude: 12'15.44; verbatimLongitude: 117'18.13; decimalLatitude: 12.25733; decimalLongitude: -117.30217; geodeticDatum: WGS84; **Identification:** identifiedBy: Helena Wiklund | Lenka Neal | Thomas Dahlgren | Adrian Glover | Madeleine Brasier | Regan Drennan | Eva Stewart; dateIdentified: 2021-04-20; identificationRemarks: identified by DNA and morphology; **Event:** eventID: OMS1_AB02_EB06; samplingProtocol: Brenke Epibenthic Sledge; eventDate: 2015-03-01; eventTime: 04:02; habitat: Abyssal plain; fieldNotes: Collected from supra net (on the epibenthic sledge); **Record Level:** language: en; institutionCode: NHMUK; collectionCode: ZOO; datasetName: ABYSSLINE; basisOfRecord: PreservedSpecimen
- **Type status:** Other material. **Occurrence:** catalogNumber: NHMUK ANEA 2023.517; recordNumber: NHM_2633; recordedBy: Adrian Glover | Helena Wiklund | Thomas Dahlgren | Madeleine Brasier; individualCount: 1; preparations: specimen stored in 80% non-denatured ethanol aqueous solution | DNA voucher stored in buffer; otherCatalogNumbers: 0174126157; associatedSequences: OQ746782 (16S); occurrenceID: D0E4BAA0-ACD0-55D8-A295-755B7D1A559B; **Taxon:** taxonConceptID: Polynoidae sp. (NHM_747D); scientificName: Polynoidae; kingdom: Animalia; phylum: Annelida; class: Polychaeta; order: Phyllodocida; family: Polynoidae; taxonRank: family; scientificNameAuthorship: Kinberg, 1856; **Location:** waterBody: Pacific; stateProvince: Clarion Clipperton Zone; locality: Ocean Mineral Singapore exploration claim Stratum A; verbatimLocality: OMS Stratum A; maximumDepthInMeters: 4122; locationRemarks: Deployment EB04; at Station S1; from R/V Thomas G. Thompson Cruise no. TN319; verbatimLatitude: 12'08.02; verbatimLongitude: 117'17.52; decimalLatitude: 12.13367; decimalLongitude: -117.292; geodeticDatum: WGS84; **Identification:** identifiedBy: Helena Wiklund | Lenka Neal | Thomas Dahlgren | Adrian Glover | Madeleine Brasier | Regan Drennan | Eva Stewart; dateIdentified: 2021-04-20; identificationRemarks: identified by DNA and morphology; **Event:** eventID: OMS1_AB02_EB04; samplingProtocol: Brenke Epibenthic Sledge; eventDate: 2015-02-24; eventTime: 19:10; habitat: Abyssal plain; fieldNotes: Collected from supra net (on the epibenthic sledge); **Record Level:** language: en; institutionCode: NHMUK; collectionCode: ZOO; datasetName: ABYSSLINE; basisOfRecord: PreservedSpecimen
- **Type status:** Other material. **Occurrence:** catalogNumber: NHMUK ANEA 2023.518; recordNumber: NHM_2784; recordedBy: Adrian Glover | Helena Wiklund | Thomas Dahlgren | Madeleine Brasier; individualCount: 1; preparations: specimen stored in 80% non-denatured ethanol aqueous solution | DNA voucher stored in buffer; otherCatalogNumbers: 0174126177; associatedSequences: OQ746784 (16S) | OQ738618 (COI); occurrenceID: 13C35B93-24D6-5D88-AB61-2895F9346548; **Taxon:** taxonConceptID: Polynoidae sp. (NHM_747D); scientificName: Polynoidae; kingdom: Animalia; phylum: Annelida; class: Polychaeta; order: Phyllodocida; family: Polynoidae; taxonRank: family; scientificNameAuthorship: Kinberg, 1856; **Location:** waterBody: Pacific; stateProvince: Clarion Clipperton Zone; locality: Ocean Mineral Singapore exploration claim Stratum A; verbatimLocality: OMS Stratum A; maximumDepthInMeters: 4100; locationRemarks: Deployment EB05; at Station S2; from R/V Thomas G. Thompson Cruise no. TN319; verbatimLatitude: 12'06.93; verbatimLongitude: 117'09.87; decimalLatitude: 12.1155; decimalLongitude: -117.1645; geodeticDatum: WGS84; **Identification:** identifiedBy: Helena Wiklund | Lenka Neal | Thomas Dahlgren | Adrian Glover | Madeleine Brasier | Regan Drennan | Eva Stewart; dateIdentified: 2021-04-20; identificationRemarks: identified by DNA and morphology; **Event:** eventID: OMS1_AB02_EB05; samplingProtocol: Brenke Epibenthic Sledge; eventDate: 2015-02-26; eventTime: 21:29; habitat: Abyssal plain; fieldNotes: Collected from supra net (on the epibenthic sledge); **Record Level:** language: en; institutionCode: NHMUK; collectionCode: ZOO; datasetName: ABYSSLINE; basisOfRecord: PreservedSpecimen
- **Type status:** Other material. **Occurrence:** catalogNumber: NHMUK ANEA 2023.515; recordNumber: NHM_2205; recordedBy: Adrian Glover | Helena Wiklund | Thomas Dahlgren | Madeleine Brasier; individualCount: 1; preparations: specimen stored in 80% non-denatured ethanol aqueous solution | DNA voucher stored in buffer; otherCatalogNumbers: 0174126228; associatedSequences: OQ746765 (16S); occurrenceID: 81C3CB9A-CFE5-5FF2-8D57-F2D07240622E; **Taxon:** taxonConceptID: Polynoidae sp. (NHM_747D); scientificName: Polynoidae; kingdom: Animalia; phylum: Annelida; class: Polychaeta; order: Phyllodocida; family: Polynoidae; taxonRank: family; scientificNameAuthorship: Kinberg, 1856; **Location:** waterBody: Pacific; stateProvince: Clarion Clipperton Zone; locality: UK Seabed Resources Ltd exploration area UK-1 Stratum B; verbatimLocality: UK1 Stratum B; maximumDepthInMeters: 4233; locationRemarks: Deployment EB09; at Station U1; from R/V Thomas G. Thompson Cruise no. TN319; verbatimLatitude: 12'21.81; verbatimLongitude: 116'40.86; decimalLatitude: 12.3635; decimalLongitude: -116.681; geodeticDatum: WGS84; **Identification:** identifiedBy: Helena Wiklund | Lenka Neal | Thomas Dahlgren | Adrian Glover | Madeleine Brasier | Regan Drennan | Eva Stewart; dateIdentified: 2021-04-20; identificationRemarks: identified by DNA and morphology; **Event:** eventID: UK1_AB02_EB09; samplingProtocol: Brenke Epibenthic Sledge; eventDate: 2015-03-10; eventTime: 10:46; habitat: Abyssal plain; fieldNotes: Collected from supra net (on the epibenthic sledge); **Record Level:** language: en; institutionCode: NHMUK; collectionCode: ZOO; datasetName: ABYSSLINE; basisOfRecord: PreservedSpecimen
- **Type status:** Other material. **Occurrence:** catalogNumber: NHMUK ANEA 2023.514; recordNumber: NHM_0747D; recordedBy: Adrian Glover | Helena Wiklund | Thomas Dahlgren | Madeleine Brasier; individualCount: 1; preparations: specimen stored in 80% non-denatured ethanol aqueous solution | DNA voucher stored in buffer; otherCatalogNumbers: 0174126578; associatedSequences: OQ746549 (16S) | OQ746855 (18S); occurrenceID: 5B4E3E5B-BACA-59B6-A983-DDF94497010B; **Taxon:** taxonConceptID: Polynoidae sp. (NHM_747D); scientificName: Polynoidae; kingdom: Animalia; phylum: Annelida; class: Polychaeta; order: Phyllodocida; family: Polynoidae; taxonRank: family; scientificNameAuthorship: Kinberg, 1856; **Location:** waterBody: Pacific; stateProvince: Clarion Clipperton Zone; locality: UK Seabed Resources Ltd exploration area UK-1 Stratum B; verbatimLocality: UK1 Stratum B; maximumDepthInMeters: 4425; locationRemarks: Deployment EB02; at Station U5; from R/V Thomas G. Thompson Cruise no. TN319; verbatimLatitude: 12'32.23; verbatimLongitude: 116'36.25; decimalLatitude: 12.53717; decimalLongitude: -116.60417; geodeticDatum: WGS84; **Identification:** identifiedBy: Helena Wiklund | Lenka Neal | Thomas Dahlgren | Adrian Glover | Madeleine Brasier | Regan Drennan | Eva Stewart; dateIdentified: 2021-04-20; identificationRemarks: identified by DNA and morphology; **Event:** eventID: UK1_AB02_EB02; samplingProtocol: Brenke Epibenthic Sledge; eventDate: 2015-02-20; eventTime: 06:24; habitat: Abyssal plain; fieldNotes: Collected from epi net (on the epibenthic sledge); **Record Level:** language: en; institutionCode: NHMUK; collectionCode: ZOO; datasetName: ABYSSLINE; basisOfRecord: PreservedSpecimen

##### Distribution

Eastern Clarion-Clipperton Zone, central Pacific Ocean.

##### Diagnosis

**Figure 88.**
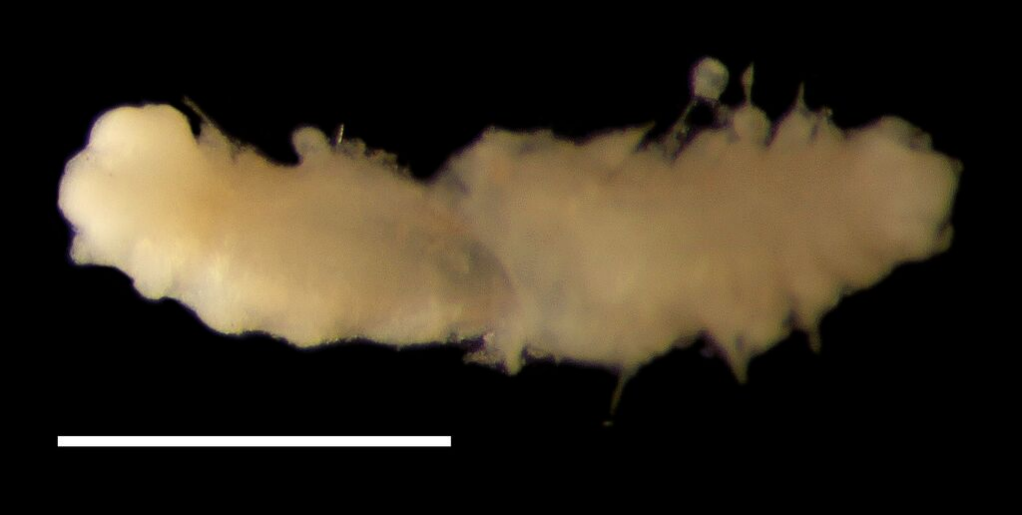
Polynoidae sp. (NHM_747D), damaged preserved specimen NHM_747D in dorsal view. Scale bar 1 mm.

Damaged specimens (Fig. 88) consistent with placement within family Polynoidae, based on morphology and DNA. Genetically matches with *Macellicephala* sp. 442 as identified in Bonifácio et al. (2021).

#### Polynoidae sp. (NHM_756)

66F70508-A96F-500C-B370-651802380F56

##### Materials

- **Type status:** Other material. **Occurrence:** catalogNumber: NHMUK ANEA 2023.520; recordNumber: NHM_1933; recordedBy: Adrian Glover | Helena Wiklund | Thomas Dahlgren | Madeleine Brasier; individualCount: 1; preparations: specimen stored in 80% non-denatured ethanol aqueous solution | DNA voucher stored in buffer; otherCatalogNumbers: 0174126744; associatedSequences: OQ746734 (16S); occurrenceID: DD41227F-6E65-5207-BE8F-FB126DAE7425; **Taxon:** taxonConceptID: Polynoidae sp. (NHM_756); scientificName: Polynoidae; kingdom: Animalia; phylum: Annelida; class: Polychaeta; order: Phyllodocida; family: Polynoidae; taxonRank: family; scientificNameAuthorship: Kinberg, 1856; **Location:** waterBody: Pacific; stateProvince: Clarion Clipperton Zone; locality: Ocean Mineral Singapore exploration claim Stratum A; verbatimLocality: OMS Stratum A; maximumDepthInMeters: 4094; locationRemarks: Deployment EB11; at Station S10; from R/V Thomas G. Thompson Cruise no. TN319; verbatimLatitude: 12°02.49’; verbatimLongitude: 117°13.03’; decimalLatitude: 12.0415; decimalLongitude: -117.21717; geodeticDatum: WGS84; **Identification:** identifiedBy: Helena Wiklund | Lenka Neal | Thomas Dahlgren | Adrian Glover | Madeleine Brasier | Regan Drennan | Eva Stewart; dateIdentified: 2021-04-20; identificationRemarks: identified by DNA and morphology; **Event:** eventID: OMS1_AB02_EB11; samplingProtocol: Brenke Epibenthic Sledge; eventDate: 2015-03-13; habitat: Abyssal plain; fieldNotes: Collected from epi net (on the epibenthic sledge); **Record Level:** language: en; institutionCode: NHMUK; collectionCode: ZOO; datasetName: ABYSSLINE; basisOfRecord: PreservedSpecimen
- **Type status:** Other material. **Occurrence:** catalogNumber: NHMUK ANEA 2023.519; recordNumber: NHM_0756; recordedBy: Adrian Glover | Helena Wiklund | Thomas Dahlgren | Madeleine Brasier; individualCount: 1; preparations: specimen stored in 80% non-denatured ethanol aqueous solution | DNA voucher stored in buffer; otherCatalogNumbers: 0174127330; associatedSequences: OQ746554 (16S) | OQ746857 (18S); occurrenceID: A25C7918-11C7-51C4-A01A-E936246B5BB3; **Taxon:** taxonConceptID: Polynoidae sp. (NHM_756); scientificName: Polynoidae; kingdom: Animalia; phylum: Annelida; class: Polychaeta; order: Phyllodocida; family: Polynoidae; taxonRank: family; scientificNameAuthorship: Kinberg, 1856; **Location:** waterBody: Pacific; stateProvince: Clarion Clipperton Zone; locality: UK Seabed Resources Ltd exploration area UK-1 Stratum B; verbatimLocality: UK1 Stratum B; maximumDepthInMeters: 4425; locationRemarks: Deployment EB02; at Station U5; from R/V Thomas G. Thompson Cruise no. TN319; verbatimLatitude: 12'32.23; verbatimLongitude: 116'36.25; decimalLatitude: 12.53717; decimalLongitude: -116.60417; geodeticDatum: WGS84; **Identification:** identifiedBy: Helena Wiklund | Lenka Neal | Thomas Dahlgren | Adrian Glover | Madeleine Brasier | Regan Drennan | Eva Stewart; dateIdentified: 2021-04-20; identificationRemarks: identified by DNA and morphology; **Event:** eventID: UK1_AB02_EB02; samplingProtocol: Brenke Epibenthic Sledge; eventDate: 2015-02-20; eventTime: 06:24; habitat: Abyssal plain; fieldNotes: Collected from epi net (on the epibenthic sledge); **Record Level:** language: en; institutionCode: NHMUK; collectionCode: ZOO; datasetName: ABYSSLINE; basisOfRecord: PreservedSpecimen

##### Distribution

Eastern Clarion-Clipperton Zone, central Pacific Ocean.

##### Diagnosis

**Figure 89.**
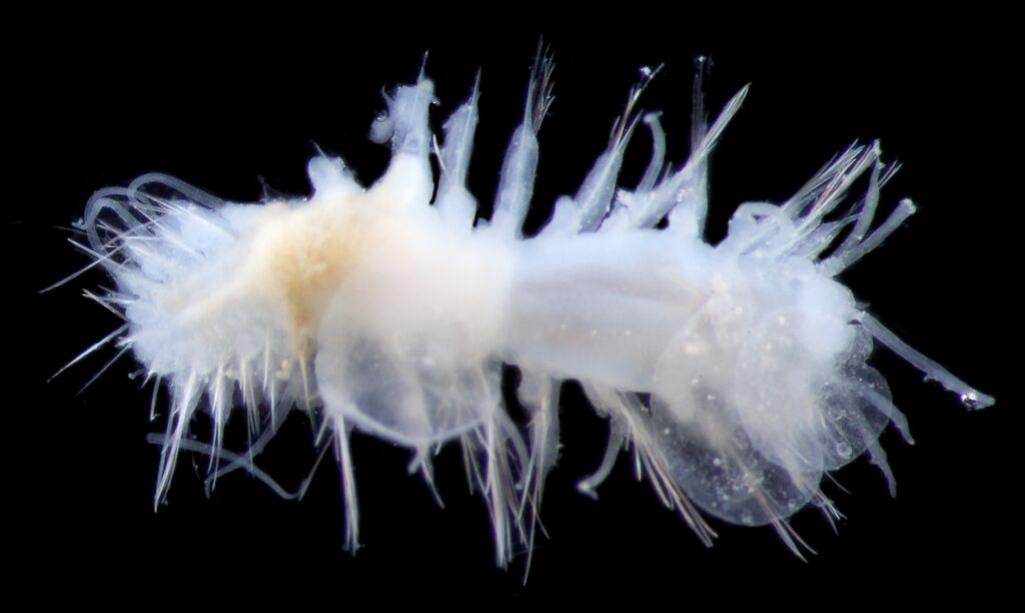
Polynoidae sp. (NHM_756), complete live specimen NHM_756 in dorsal view.

Damaged specimens (Fig. 89) consistent with placement within family Polynoidae, based on morphology and DNA. Genetically matches with *Macellicephala* sp. 437 as identified in Bonifácio et al. (2021).

#### Polynoidae sp. (NHM_773B)

A121697C-990A-55F2-B042-A6CBDFFC7B3E

##### Materials

- **Type status:** Other material. **Occurrence:** catalogNumber: NHMUK ANEA 2023.525; recordNumber: NHM_1355B; recordedBy: Adrian Glover | Helena Wiklund | Thomas Dahlgren | Madeleine Brasier; individualCount: 1; preparations: specimen stored in 80% non-denatured ethanol aqueous solution | DNA voucher stored in buffer; otherCatalogNumbers: 0174126239; associatedSequences: OQ746675 (16S) | OQ738581 (COI); occurrenceID: 3ED94846-4343-57F8-ACF4-8545B2B878D3; **Taxon:** taxonConceptID: Polynoidae sp. (NHM_773B); scientificName: Polynoidae; kingdom: Animalia; phylum: Annelida; class: Polychaeta; order: Phyllodocida; family: Polynoidae; taxonRank: family; scientificNameAuthorship: Kinberg, 1856; **Location:** waterBody: Pacific; stateProvince: Clarion Clipperton Zone; locality: Ocean Mineral Singapore exploration claim Stratum A; verbatimLocality: OMS Stratum A; maximumDepthInMeters: 4302; locationRemarks: Deployment EB06; at Station S5; from R/V Thomas G. Thompson Cruise no. TN319; verbatimLatitude: 12'15.44; verbatimLongitude: 117'18.13; decimalLatitude: 12.25733; decimalLongitude: -117.30217; geodeticDatum: WGS84; **Identification:** identifiedBy: Helena Wiklund | Lenka Neal | Thomas Dahlgren | Adrian Glover | Madeleine Brasier | Regan Drennan | Eva Stewart; dateIdentified: 2021-04-20; identificationRemarks: identified by DNA and morphology; **Event:** eventID: OMS1_AB02_EB06; samplingProtocol: Brenke Epibenthic Sledge; eventDate: 2015-03-01; eventTime: 04:02; habitat: Abyssal plain; fieldNotes: Collected from epi net (on the epibenthic sledge); **Record Level:** language: en; institutionCode: NHMUK; collectionCode: ZOO; datasetName: ABYSSLINE; basisOfRecord: PreservedSpecimen
- **Type status:** Other material. **Occurrence:** catalogNumber: NHMUK ANEA 2023.527; recordNumber: NHM_2291; recordedBy: Adrian Glover | Helena Wiklund | Thomas Dahlgren | Madeleine Brasier; individualCount: 1; preparations: specimen stored in 80% non-denatured ethanol aqueous solution | DNA voucher stored in buffer; otherCatalogNumbers: 0174126156; associatedSequences: OQ746770 (16S); occurrenceID: 252F6985-103B-5149-855F-9BA85ACF095E; **Taxon:** taxonConceptID: Polynoidae sp. (NHM_773B); scientificName: Polynoidae; kingdom: Animalia; phylum: Annelida; class: Polychaeta; order: Phyllodocida; family: Polynoidae; taxonRank: family; scientificNameAuthorship: Kinberg, 1856; **Location:** waterBody: Pacific; stateProvince: Clarion Clipperton Zone; locality: Ocean Mineral Singapore exploration claim Stratum A; verbatimLocality: OMS Stratum A; maximumDepthInMeters: 4302; locationRemarks: Deployment EB06; at Station S5; from R/V Thomas G. Thompson Cruise no. TN319; verbatimLatitude: 12'15.44; verbatimLongitude: 117'18.13; decimalLatitude: 12.25733; decimalLongitude: -117.30217; geodeticDatum: WGS84; **Identification:** identifiedBy: Helena Wiklund | Lenka Neal | Thomas Dahlgren | Adrian Glover | Madeleine Brasier | Regan Drennan | Eva Stewart; dateIdentified: 2021-04-20; identificationRemarks: identified by DNA and morphology; **Event:** eventID: OMS1_AB02_EB06; samplingProtocol: Brenke Epibenthic Sledge; eventDate: 2015-03-01; eventTime: 04:02; habitat: Abyssal plain; fieldNotes: Collected from supra net (on the epibenthic sledge); **Record Level:** language: en; institutionCode: NHMUK; collectionCode: ZOO; datasetName: ABYSSLINE; basisOfRecord: PreservedSpecimen
- **Type status:** Other material. **Occurrence:** catalogNumber: NHMUK ANEA 2023.526; recordNumber: NHM_1735; recordedBy: Adrian Glover | Helena Wiklund | Thomas Dahlgren | Madeleine Brasier; individualCount: 1; preparations: specimen stored in 80% non-denatured ethanol aqueous solution | DNA voucher stored in buffer; otherCatalogNumbers: 0174126792; associatedSequences: OQ746707 (16S) | OQ746898 (18S) | OQ738597 (COI); occurrenceID: E9A33C38-5BA1-599B-9F29-65A9A02765B0; **Taxon:** taxonConceptID: Polynoidae sp. (NHM_773B); scientificName: Polynoidae; kingdom: Animalia; phylum: Annelida; class: Polychaeta; order: Phyllodocida; family: Polynoidae; taxonRank: family; scientificNameAuthorship: Kinberg, 1856; **Location:** waterBody: Pacific; stateProvince: Clarion Clipperton Zone; locality: Ocean Mineral Singapore exploration claim Stratum A; verbatimLocality: OMS Stratum A; maximumDepthInMeters: 4045; locationRemarks: Deployment EB10; at Station S7; from R/V Thomas G. Thompson Cruise no. TN319; verbatimLatitude: 12'10.43; verbatimLongitude: 117'11.57; decimalLatitude: 12.17383; decimalLongitude: -117.19283; geodeticDatum: WGS84; **Identification:** identifiedBy: Helena Wiklund | Lenka Neal | Thomas Dahlgren | Adrian Glover | Madeleine Brasier | Regan Drennan | Eva Stewart; dateIdentified: 2021-04-20; identificationRemarks: identified by DNA and morphology; **Event:** eventID: OMS1_AB02_EB10; samplingProtocol: Brenke Epibenthic Sledge; eventDate: 2015-03-11; eventTime: 22:49; habitat: Abyssal plain; fieldNotes: Collected from epi net (on the epibenthic sledge); **Record Level:** language: en; institutionCode: NHMUK; collectionCode: ZOO; datasetName: ABYSSLINE; basisOfRecord: PreservedSpecimen
- **Type status:** Other material. **Occurrence:** catalogNumber: NHMUK ANEA 2023.524; recordNumber: NHM_0912; recordedBy: Adrian Glover | Helena Wiklund | Thomas Dahlgren | Madeleine Brasier; individualCount: 1; preparations: specimen stored in 80% non-denatured ethanol aqueous solution | DNA voucher stored in buffer; otherCatalogNumbers: 0174127380; associatedSequences: OQ738543 (COI); occurrenceID: E650AF5C-DA76-57C2-B8BF-BAD1BDE70459; **Taxon:** taxonConceptID: Polynoidae sp. (NHM_773B); scientificName: Polynoidae; kingdom: Animalia; phylum: Annelida; class: Polychaeta; order: Phyllodocida; family: Polynoidae; taxonRank: family; scientificNameAuthorship: Kinberg, 1856; **Location:** waterBody: Pacific; stateProvince: Clarion Clipperton Zone; locality: UK Seabed Resources Ltd exploration area UK-1 Stratum B; verbatimLocality: UK1 Stratum B; maximumDepthInMeters: 4198; locationRemarks: Deployment EB03; at Station U4; from R/V Thomas G. Thompson Cruise no. TN319; verbatimLatitude: 12'34.28; verbatimLongitude: 116'36.63; decimalLatitude: 12.57133; decimalLongitude: -116.6105; geodeticDatum: WGS84; **Identification:** identifiedBy: Helena Wiklund | Lenka Neal | Thomas Dahlgren | Adrian Glover | Madeleine Brasier | Regan Drennan | Eva Stewart; dateIdentified: 2021-04-20; identificationRemarks: identified by DNA and morphology; **Event:** eventID: UK1_AB02_EB03; samplingProtocol: Brenke Epibenthic Sledge; eventDate: 2015-02-23; eventTime: 05:39; habitat: Abyssal plain; fieldNotes: Collected from epi net (on the epibenthic sledge); **Record Level:** language: en; institutionCode: NHMUK; collectionCode: ZOO; datasetName: ABYSSLINE; basisOfRecord: PreservedSpecimen
- **Type status:** Other material. **Occurrence:** catalogNumber: NHMUK ANEA 2023.521; recordNumber: NHM_0773B; recordedBy: Adrian Glover | Helena Wiklund | Thomas Dahlgren | Madeleine Brasier; individualCount: 1; preparations: specimen stored in 80% non-denatured ethanol aqueous solution | DNA voucher stored in buffer; otherCatalogNumbers: 0174126551; associatedSequences: OQ746556 (16S); occurrenceID: CD692927-546D-5B66-9590-314044F5EEED; **Taxon:** taxonConceptID: Polynoidae sp. (NHM_773B); scientificName: Polynoidae; kingdom: Animalia; phylum: Annelida; class: Polychaeta; order: Phyllodocida; family: Polynoidae; taxonRank: family; scientificNameAuthorship: Kinberg, 1856; **Location:** waterBody: Pacific; stateProvince: Clarion Clipperton Zone; locality: UK Seabed Resources Ltd exploration area UK-1 Stratum B; verbatimLocality: UK1 Stratum B; maximumDepthInMeters: 4425; locationRemarks: Deployment EB02; at Station U5; from R/V Thomas G. Thompson Cruise no. TN319; verbatimLatitude: 12'32.23; verbatimLongitude: 116'36.25; decimalLatitude: 12.53717; decimalLongitude: -116.60417; geodeticDatum: WGS84; **Identification:** identifiedBy: Helena Wiklund | Lenka Neal | Thomas Dahlgren | Adrian Glover | Madeleine Brasier | Regan Drennan | Eva Stewart; dateIdentified: 2021-04-20; identificationRemarks: identified by DNA and morphology; **Event:** eventID: UK1_AB02_EB02; samplingProtocol: Brenke Epibenthic Sledge; eventDate: 2015-02-20; eventTime: 06:24; habitat: Abyssal plain; fieldNotes: Collected from epi net (on the epibenthic sledge); **Record Level:** language: en; institutionCode: NHMUK; collectionCode: ZOO; datasetName: ABYSSLINE; basisOfRecord: PreservedSpecimen
- **Type status:** Other material. **Occurrence:** catalogNumber: NHMUK ANEA 2023.522; recordNumber: NHM_0783B; recordedBy: Adrian Glover | Helena Wiklund | Thomas Dahlgren | Madeleine Brasier; individualCount: 1; preparations: specimen stored in 80% non-denatured ethanol aqueous solution | DNA voucher stored in buffer; otherCatalogNumbers: 0174126572; associatedSequences: OQ746559 (16S); occurrenceID: 1E7059D4-630F-57B1-91D4-3CE53F096D60; **Taxon:** taxonConceptID: Polynoidae sp. (NHM_773B); scientificName: Polynoidae; kingdom: Animalia; phylum: Annelida; class: Polychaeta; order: Phyllodocida; family: Polynoidae; taxonRank: family; scientificNameAuthorship: Kinberg, 1856; **Location:** waterBody: Pacific; stateProvince: Clarion Clipperton Zone; locality: UK Seabed Resources Ltd exploration area UK-1 Stratum B; verbatimLocality: UK1 Stratum B; maximumDepthInMeters: 4425; locationRemarks: Deployment EB02; at Station U5; from R/V Thomas G. Thompson Cruise no. TN319; verbatimLatitude: 12'32.23; verbatimLongitude: 116'36.25; decimalLatitude: 12.53717; decimalLongitude: -116.60417; geodeticDatum: WGS84; **Identification:** identifiedBy: Helena Wiklund | Lenka Neal | Thomas Dahlgren | Adrian Glover | Madeleine Brasier | Regan Drennan | Eva Stewart; dateIdentified: 2021-04-20; identificationRemarks: identified by DNA and morphology; **Event:** eventID: UK1_AB02_EB02; samplingProtocol: Brenke Epibenthic Sledge; eventDate: 2015-02-20; eventTime: 06:24; habitat: Abyssal plain; fieldNotes: Collected from epi net (on the epibenthic sledge); **Record Level:** language: en; institutionCode: NHMUK; collectionCode: ZOO; datasetName: ABYSSLINE; basisOfRecord: PreservedSpecimen
- **Type status:** Other material. **Occurrence:** catalogNumber: NHMUK ANEA 2023.523; recordNumber: NHM_0783D; recordedBy: Adrian Glover | Helena Wiklund | Thomas Dahlgren | Madeleine Brasier; individualCount: 1; preparations: specimen stored in 80% non-denatured ethanol aqueous solution | DNA voucher stored in buffer; otherCatalogNumbers: 0174126555; associatedSequences: OQ746560 (16S); occurrenceID: DBA078C4-E264-5CBE-8F04-C33CDC99DBC0; **Taxon:** taxonConceptID: Polynoidae sp. (NHM_773B); scientificName: Polynoidae; kingdom: Animalia; phylum: Annelida; class: Polychaeta; order: Phyllodocida; family: Polynoidae; taxonRank: family; scientificNameAuthorship: Kinberg, 1856; **Location:** waterBody: Pacific; stateProvince: Clarion Clipperton Zone; locality: UK Seabed Resources Ltd exploration area UK-1 Stratum B; verbatimLocality: UK1 Stratum B; maximumDepthInMeters: 4425; locationRemarks: Deployment EB02; at Station U5; from R/V Thomas G. Thompson Cruise no. TN319; verbatimLatitude: 12'32.23; verbatimLongitude: 116'36.25; decimalLatitude: 12.53717; decimalLongitude: -116.60417; geodeticDatum: WGS84; **Identification:** identifiedBy: Helena Wiklund | Lenka Neal | Thomas Dahlgren | Adrian Glover | Madeleine Brasier | Regan Drennan | Eva Stewart; dateIdentified: 2021-04-20; identificationRemarks: identified by DNA and morphology; **Event:** eventID: UK1_AB02_EB02; samplingProtocol: Brenke Epibenthic Sledge; eventDate: 2015-02-20; eventTime: 06:24; habitat: Abyssal plain; fieldNotes: Collected from epi net (on the epibenthic sledge); **Record Level:** language: en; institutionCode: NHMUK; collectionCode: ZOO; datasetName: ABYSSLINE; basisOfRecord: PreservedSpecimen

##### Distribution

Eastern Clarion-Clipperton Zone, central Pacific Ocean.

##### Diagnosis

**Figure 90.**
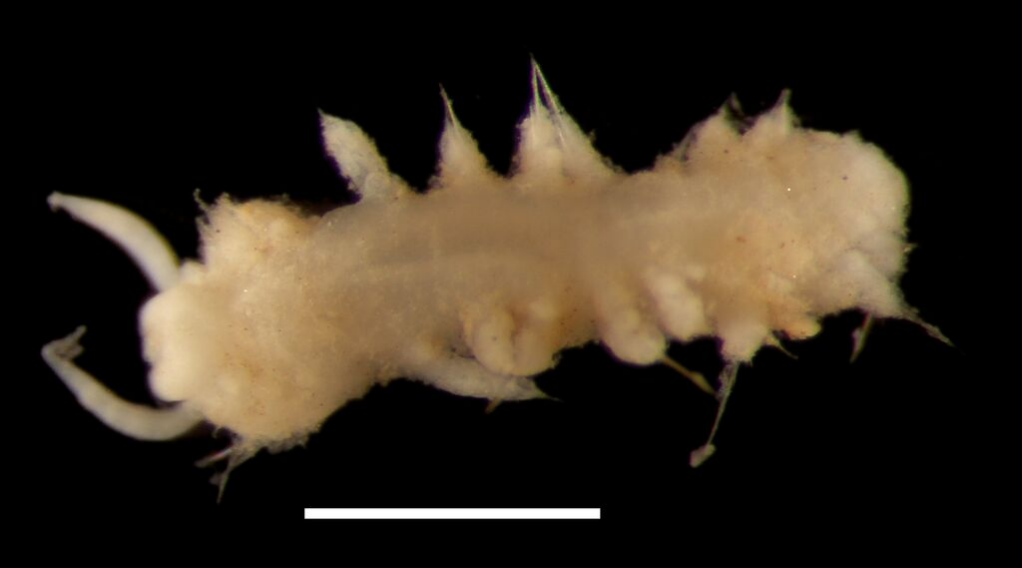
Polynoidae sp. (NHM_773B), preserved specimen NHM_ 783B in dorsal view. Scale bar 1 mm.

Damaged specimens (Fig. 90) consistent with placement within family Polynoidae, based on morphology and DNA. Genetically matches with *Macellicephala* sp. 35 as identified in Bonifácio et al. (2021).

#### Polynoidae sp. (NHM_834)

23DD7875-34BA-528A-B0AB-F75BD69E6E3F

##### Materials

- **Type status:** Other material. **Occurrence:** catalogNumber: NHMUK ANEA 2023.542; recordNumber: NHM_0834; recordedBy: Adrian Glover | Helena Wiklund | Thomas Dahlgren | Madeleine Brasier; individualCount: 1; preparations: specimen stored in 80% non-denatured ethanol aqueous solution | DNA voucher stored in buffer; otherCatalogNumbers: 0174127309; associatedSequences: OQ738537 (COI); occurrenceID: 545C885F-2527-5086-A3A5-03F5FBF57808; **Taxon:** taxonConceptID: Polynoidae sp. (NHM_834); scientificName: Polynoidae; kingdom: Animalia; phylum: Annelida; class: Polychaeta; order: Phyllodocida; family: Polynoidae; taxonRank: family; scientificNameAuthorship: Kinberg, 1856; **Location:** waterBody: Pacific; stateProvince: Clarion Clipperton Zone; locality: UK Seabed Resources Ltd exploration area UK-1 Stratum B; verbatimLocality: UK1 Stratum B; maximumDepthInMeters: 4160; locationRemarks: Deployment BC04; at Station U5; from R/V Thomas G. Thompson Cruise no. TN319; verbatimLatitude: 12'22.259; verbatimLongitude: 116'36.819; decimalLatitude: 12.37098; decimalLongitude: -116.61365; geodeticDatum: WGS84; **Identification:** identifiedBy: Helena Wiklund | Lenka Neal | Thomas Dahlgren | Adrian Glover | Madeleine Brasier | Regan Drennan | Eva Stewart; dateIdentified: 2021-04-20; identificationRemarks: identified by DNA and morphology; **Event:** eventID: UK1_AB02_BC04; samplingProtocol: USNEL Box Core; eventDate: 2015-02-21; eventTime: 03:38; habitat: Abyssal plain; fieldNotes: Collected from 0-2 cm layer of box core using a 300 micron sieve; **Record Level:** language: en; institutionCode: NHMUK; collectionCode: ZOO; datasetName: ABYSSLINE; basisOfRecord: PreservedSpecimen

##### Distribution

Eastern Clarion-Clipperton Zone, central Pacific Ocean.

##### Diagnosis

**Figure 91.**
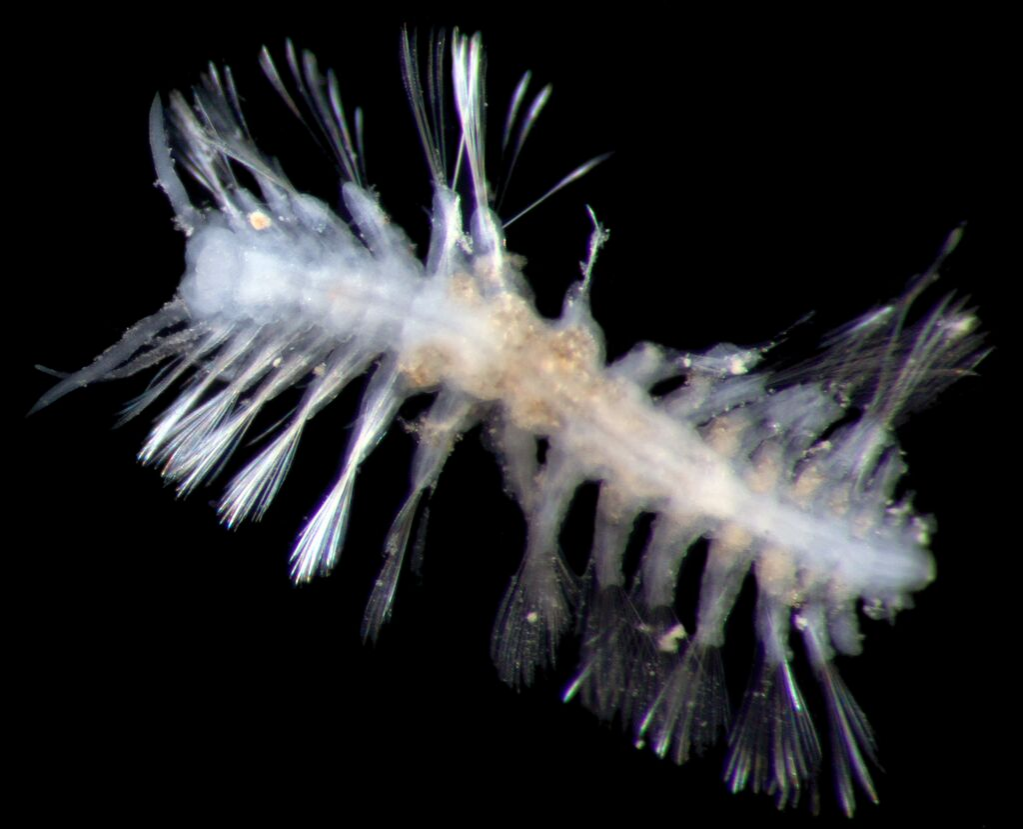
Polynoidae sp. (NHM_834), complete live specimen NHM_834 in dorsal view.

Specimen (Fig. 91) consistent with placement within family Polynoidae, based on morphology and DNA.

#### Polynoidae sp. (NHM_1000)

A320F9A5-89FA-5F6F-B2BD-D99E8A8450B5

##### Materials

- **Type status:** Other material. **Occurrence:** catalogNumber: NHMUK ANEA 2023.502; recordNumber: NHM_1000; recordedBy: Adrian Glover | Helena Wiklund | Thomas Dahlgren | Madeleine Brasier; individualCount: 1; preparations: specimen stored in 80% non-denatured ethanol aqueous solution | DNA voucher stored in buffer; otherCatalogNumbers: 0174126794; associatedSequences: OQ746601 (16S) | OQ746868 (18S) | OQ738553 (COI); occurrenceID: AFD22D55-7BC3-5B39-80AB-24003F5DBF56; **Taxon:** taxonConceptID: Polynoidae sp. (NHM_1000); scientificName: Polynoidae; kingdom: Animalia; phylum: Annelida; class: Polychaeta; order: Phyllodocida; family: Polynoidae; taxonRank: family; scientificNameAuthorship: Kinberg, 1856; **Location:** waterBody: Pacific; stateProvince: Clarion Clipperton Zone; locality: Ocean Mineral Singapore exploration claim Stratum A; verbatimLocality: OMS Stratum A; maximumDepthInMeters: 4122; locationRemarks: Deployment EB04; at Station S1; from R/V Thomas G. Thompson Cruise no. TN319; verbatimLatitude: 12'08.02; verbatimLongitude: 117'17.52; decimalLatitude: 12.13367; decimalLongitude: -117.292; geodeticDatum: WGS84; **Identification:** identifiedBy: Helena Wiklund | Lenka Neal | Thomas Dahlgren | Adrian Glover | Madeleine Brasier | Regan Drennan | Eva Stewart; dateIdentified: 2021-04-20; identificationRemarks: identified by DNA and morphology; **Event:** eventID: OMS1_AB02_EB04; samplingProtocol: Brenke Epibenthic Sledge; eventDate: 2015-02-24; eventTime: 19:10; habitat: Abyssal plain; fieldNotes: Collected from epi net (on the epibenthic sledge); **Record Level:** language: en; institutionCode: NHMUK; collectionCode: ZOO; datasetName: ABYSSLINE; basisOfRecord: PreservedSpecimen

##### Distribution

Eastern Clarion-Clipperton Zone, central Pacific Ocean.

##### Diagnosis

**Figure 92.**
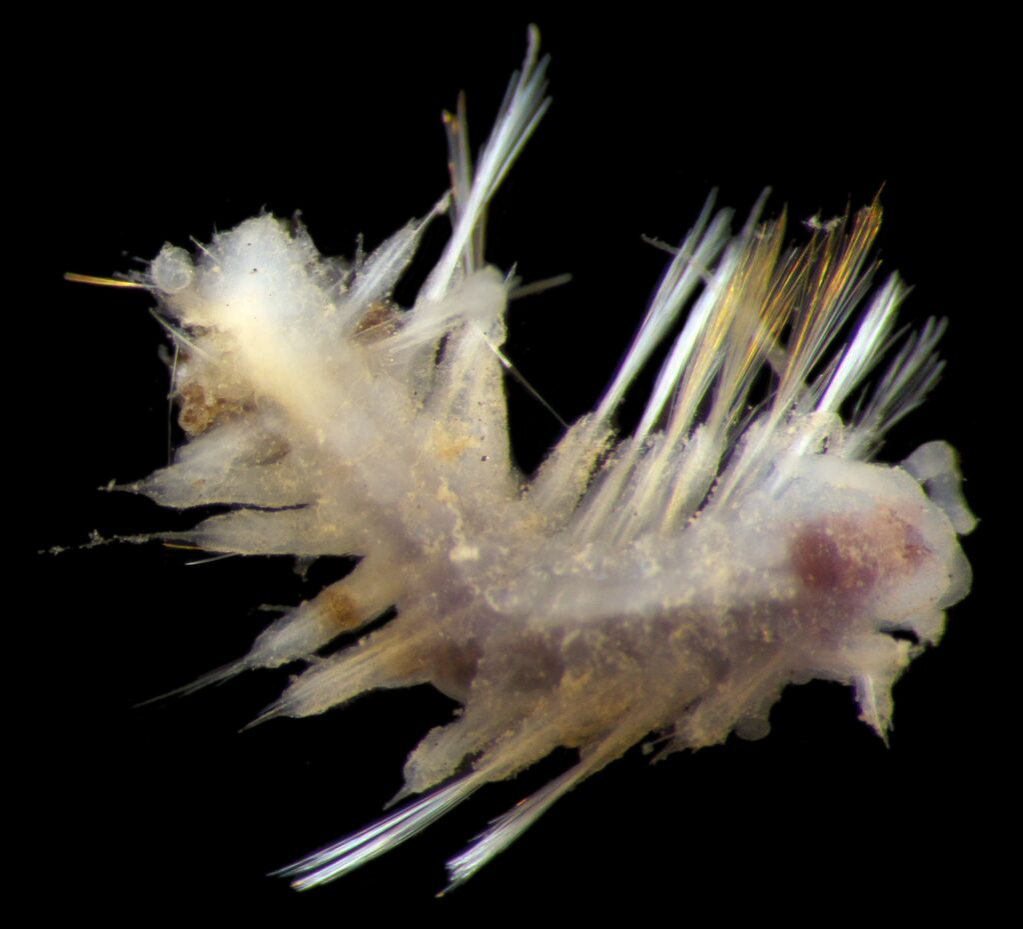
Polynoidae sp. (NHM_1000), live specimen NHM_1000 in dorsal view.

Specimen (Fig. 92) consistent with placement within family Polynoidae, based on morphology and DNA. Genetically matches with *Macellicephala* sp. 444 as identified in Bonifácio et al. (2021).

#### Polynoidae sp. (NHM_1763)

284B6987-1025-537D-9DEC-37BD8A38867C

##### Materials

- **Type status:** Other material. **Occurrence:** catalogNumber: NHMUK ANEA 2023.531; recordNumber: NHM_1763; recordedBy: Adrian Glover | Helena Wiklund | Thomas Dahlgren | Madeleine Brasier; individualCount: 1; preparations: specimen stored in 80% non-denatured ethanol aqueous solution | DNA voucher stored in buffer; otherCatalogNumbers: 0174126791; associatedSequences: OQ746714 (16S) | OQ746900 (18S); occurrenceID: C7EBF171-0C0F-5EB1-BA04-7DFC0C16AB10; **Taxon:** taxonConceptID: Polynoidae sp. (NHM_1763); scientificName: Polynoidae; kingdom: Animalia; phylum: Annelida; class: Polychaeta; order: Phyllodocida; family: Polynoidae; taxonRank: family; scientificNameAuthorship: Kinberg, 1856; **Location:** waterBody: Pacific; stateProvince: Clarion Clipperton Zone; locality: Ocean Mineral Singapore exploration claim Stratum A; verbatimLocality: OMS Stratum A; maximumDepthInMeters: 4045; locationRemarks: Deployment EB10; at Station S7; from R/V Thomas G. Thompson Cruise no. TN319; verbatimLatitude: 12'10.43; verbatimLongitude: 117'11.57; decimalLatitude: 12.17383; decimalLongitude: -117.19283; geodeticDatum: WGS84; **Identification:** identifiedBy: Helena Wiklund | Lenka Neal | Thomas Dahlgren | Adrian Glover | Madeleine Brasier | Regan Drennan | Eva Stewart; dateIdentified: 2021-04-20; identificationRemarks: identified by DNA and morphology; **Event:** eventID: OMS1_AB02_EB10; samplingProtocol: Brenke Epibenthic Sledge; eventDate: 2015-03-11; eventTime: 22:49; habitat: Abyssal plain; fieldNotes: Collected from epi net (on the epibenthic sledge); **Record Level:** language: en; institutionCode: NHMUK; collectionCode: ZOO; datasetName: ABYSSLINE; basisOfRecord: PreservedSpecimen

##### Distribution

Eastern Clarion-Clipperton Zone, central Pacific Ocean.

##### Diagnosis

**Figure 93.**
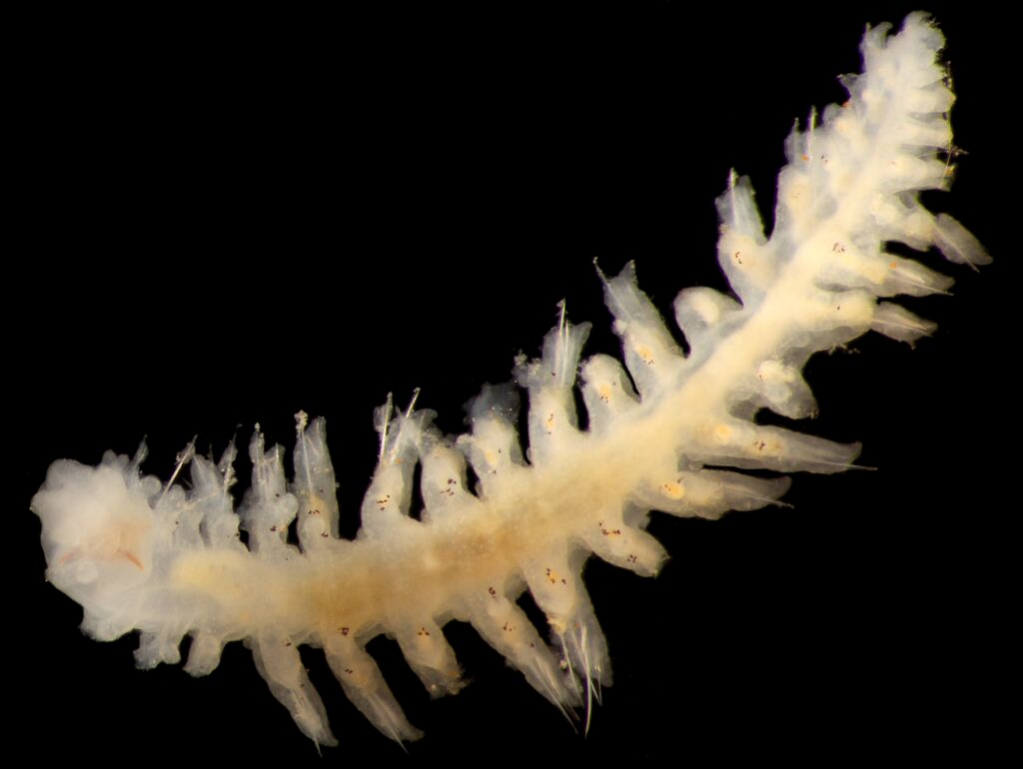
Polynoidae sp. (NHM_1763), live specimen NHM_1763 in dorsal view.

Damaged specimen (Fig. 93) consistent with placement within family Polynoidae, based on morphology and DNA.

#### Polynoidae sp. (NHM_2097)

D261ADBB-46AB-543F-8238-9F695074AFBB

##### Materials

- **Type status:** Other material. **Occurrence:** catalogNumber: NHMUK ANEA 2023.532; recordNumber: NHM_2097; recordedBy: Adrian Glover | Helena Wiklund | Thomas Dahlgren | Madeleine Brasier; individualCount: 1; preparations: specimen stored in 80% non-denatured ethanol aqueous solution | DNA voucher stored in buffer; otherCatalogNumbers: 0174126725; associatedSequences: OQ746752 (16S) | OQ746905 (18S) | OQ738607 (COI); occurrenceID: 6C93EF11-99FF-5325-95AE-E713C5476748; **Taxon:** taxonConceptID: Polynoidae sp. (NHM_2097); scientificName: Polynoidae; kingdom: Animalia; phylum: Annelida; class: Polychaeta; order: Phyllodocida; family: Polynoidae; taxonRank: family; scientificNameAuthorship: Kinberg, 1856; **Location:** waterBody: Pacific; stateProvince: Clarion Clipperton Zone; locality: Area of Particular Interest APEI-6; verbatimLocality: APEI-6; maximumDepthInMeters: 4026; locationRemarks: Deployment EB13; at Station APEI; from R/V Thomas G. Thompson Cruise no. TN319; verbatimLatitude: 19 27.874; verbatimLongitude: 120 01.525; decimalLatitude: 19.46457; decimalLongitude: -120.02542; geodeticDatum: WGS84; **Identification:** identifiedBy: Helena Wiklund | Lenka Neal | Thomas Dahlgren | Adrian Glover | Madeleine Brasier | Regan Drennan | Eva Stewart; dateIdentified: 2021-04-20; identificationRemarks: identified by DNA and morphology; **Event:** eventID: APEI6_AB02_EB13; samplingProtocol: Brenke Epibenthic Sledge; eventDate: 2015-03-20; eventTime: 16:12; habitat: Abyssal plain; fieldNotes: Collected from epi net (on the epibenthic sledge); **Record Level:** language: en; institutionCode: NHMUK; collectionCode: ZOO; datasetName: ABYSSLINE; basisOfRecord: PreservedSpecimen

##### Distribution

Eastern Clarion-Clipperton Zone, central Pacific Ocean.

##### Diagnosis

**Figure 94.**
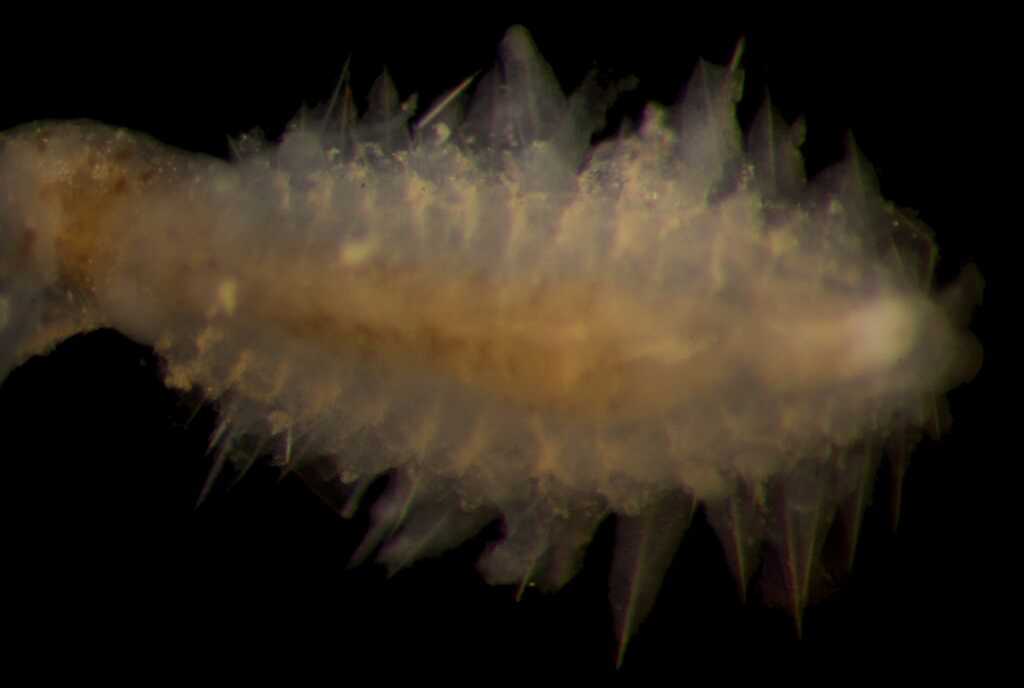
Polynoidae sp. (NHM_2097), live specimen NHM_2097 in dorsal view.

Damaged specimen (Fig. 94) consistent with placement within family Polynoidae, based on morphology and DNA.

#### Polynoidae sp. (NHM_2099)

49F8D2BA-9817-58E3-ADC6-34213D688B41

##### Materials

- **Type status:** Other material. **Occurrence:** catalogNumber: NHMUK ANEA 2023.533; recordNumber: NHM_2099; recordedBy: Adrian Glover | Helena Wiklund | Thomas Dahlgren | Madeleine Brasier; individualCount: 1; preparations: specimen stored in 80% non-denatured ethanol aqueous solution | DNA voucher stored in buffer; otherCatalogNumbers: 0174126720; associatedSequences: OQ746753 (16S) | OQ746906 (18S); occurrenceID: 10973B7C-E66E-5FEB-9F75-1C4E1CCB2B13; **Taxon:** taxonConceptID: Polynoidae sp. (NHM_2099); scientificName: Polynoidae; kingdom: Animalia; phylum: Annelida; class: Polychaeta; order: Phyllodocida; family: Polynoidae; taxonRank: family; scientificNameAuthorship: Kinberg, 1856; **Location:** waterBody: Pacific; stateProvince: Clarion Clipperton Zone; locality: Area of Particular Interest APEI-6; verbatimLocality: APEI-6; maximumDepthInMeters: 4026; locationRemarks: Deployment EB13; at Station APEI; from R/V Thomas G. Thompson Cruise no. TN319; verbatimLatitude: 19 27.874; verbatimLongitude: 120 01.525; decimalLatitude: 19.46457; decimalLongitude: -120.02542; geodeticDatum: WGS84; **Identification:** identifiedBy: Helena Wiklund | Lenka Neal | Thomas Dahlgren | Adrian Glover | Madeleine Brasier | Regan Drennan | Eva Stewart; dateIdentified: 2021-04-20; identificationRemarks: identified by DNA and morphology; **Event:** eventID: APEI6_AB02_EB13; samplingProtocol: Brenke Epibenthic Sledge; eventDate: 2015-03-20; eventTime: 16:12; habitat: Abyssal plain; fieldNotes: Collected from epi net (on the epibenthic sledge); **Record Level:** language: en; institutionCode: NHMUK; collectionCode: ZOO; datasetName: ABYSSLINE; basisOfRecord: PreservedSpecimen

##### Distribution

Eastern Clarion-Clipperton Zone, central Pacific Ocean.

##### Diagnosis

**Figure 95.**
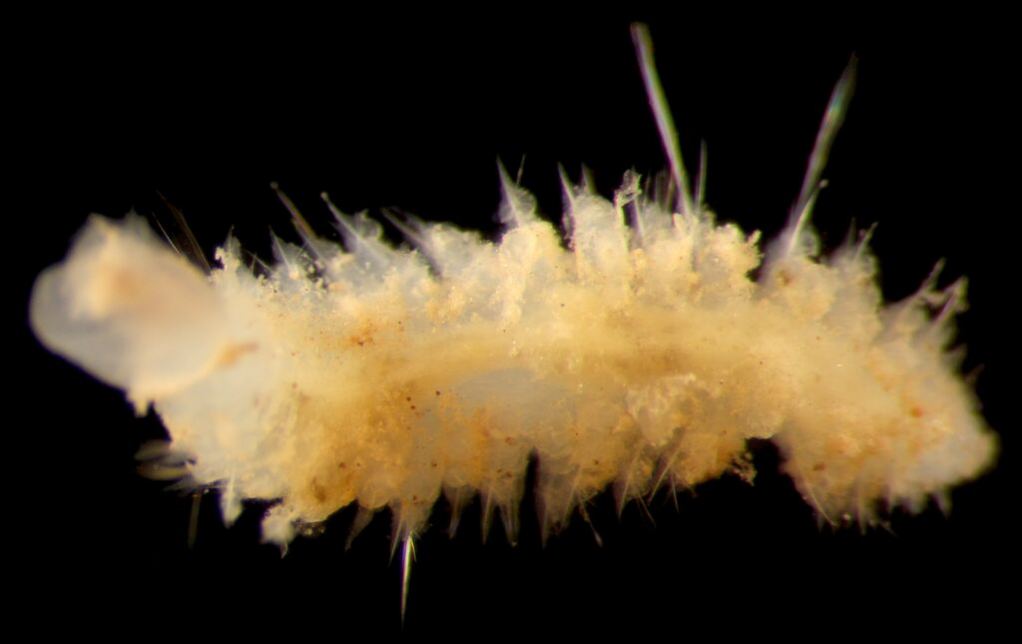
Polynoidae sp. (NHM_2099), live specimen NHM_2099 in dorsal view.

Damaged specimen (Fig. 95) consistent with placement within family Polynoidae, based on morphology and DNA.

#### Polynoidae sp. (NHM_2122)

647836AE-582F-575D-8E61-FBC978A02B4F

##### Materials

- **Type status:** Other material. **Occurrence:** catalogNumber: NHMUK ANEA 2023.511; recordNumber: NHM_2122; recordedBy: Adrian Glover | Helena Wiklund | Thomas Dahlgren | Madeleine Brasier; individualCount: 1; preparations: specimen stored in 80% non-denatured ethanol aqueous solution | DNA voucher stored in buffer; otherCatalogNumbers: 0174126793; associatedSequences: OQ746757 (16S) | OQ746909 (18S); occurrenceID: 00990127-1A40-5AF9-B16E-F4B2D059C0A8; **Taxon:** taxonConceptID: Polynoidae sp. (NHM_2122); scientificName: Polynoidae; kingdom: Animalia; phylum: Annelida; class: Polychaeta; order: Phyllodocida; family: Polynoidae; taxonRank: family; scientificNameAuthorship: Kinberg, 1856; **Location:** waterBody: Pacific; stateProvince: Clarion Clipperton Zone; locality: Area of Particular Interest APEI-6; verbatimLocality: APEI-6; maximumDepthInMeters: 4026; locationRemarks: Deployment EB13; at Station APEI; from R/V Thomas G. Thompson Cruise no. TN319; verbatimLatitude: 19 27.874; verbatimLongitude: 120 01.525; decimalLatitude: 19.46457; decimalLongitude: -120.02542; geodeticDatum: WGS84; **Identification:** identifiedBy: Helena Wiklund | Lenka Neal | Thomas Dahlgren | Adrian Glover | Madeleine Brasier | Regan Drennan | Eva Stewart; dateIdentified: 2021-04-20; identificationRemarks: identified by DNA and morphology; **Event:** eventID: APEI6_AB02_EB13; samplingProtocol: Brenke Epibenthic Sledge; eventDate: 2015-03-20; eventTime: 16:12; habitat: Abyssal plain; fieldNotes: Collected from epi net (on the epibenthic sledge); **Record Level:** language: en; institutionCode: NHMUK; collectionCode: ZOO; datasetName: ABYSSLINE; basisOfRecord: PreservedSpecimen

##### Distribution

Eastern Clarion-Clipperton Zone, central Pacific Ocean.

##### Diagnosis

**Figure 96.**
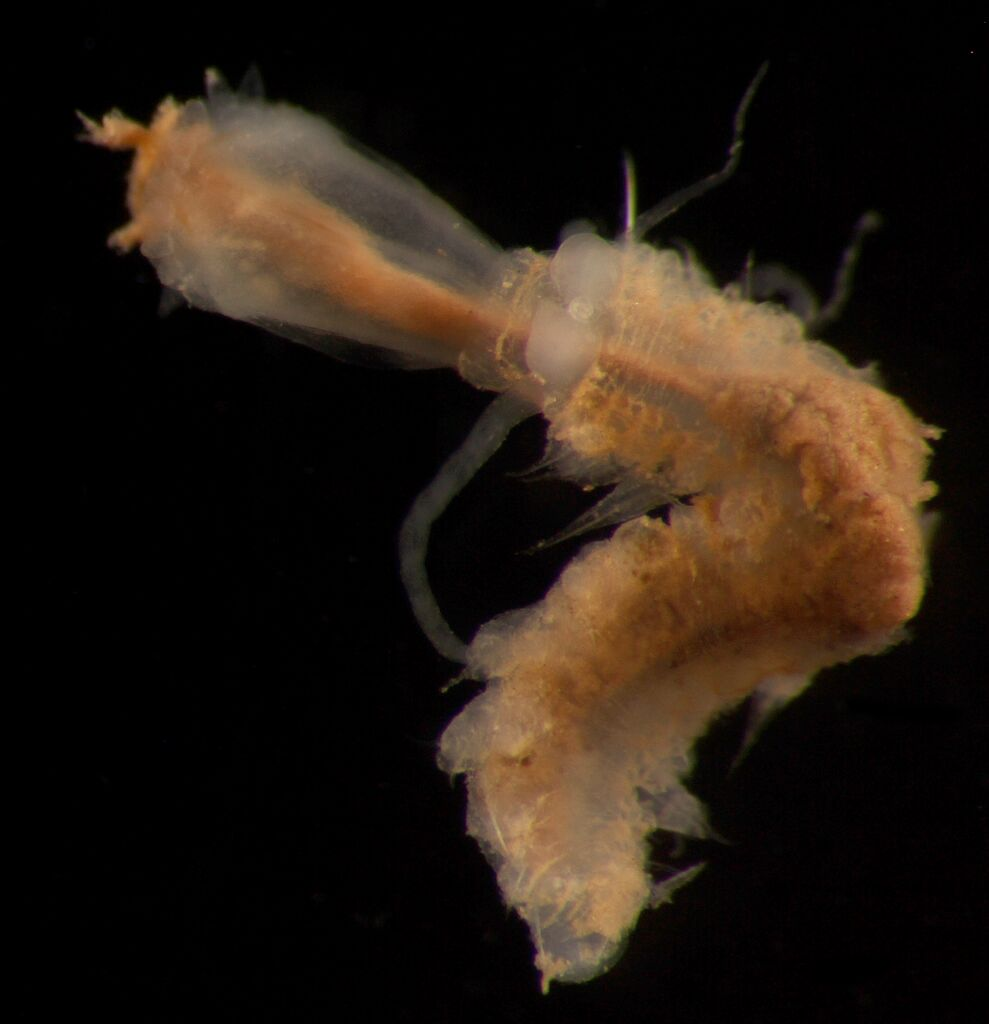
Polynoidae sp. (NHM_2122), live specimen NHM_2122 in dorsal view.

Specimen (Fig. 96) consistent with placement within family Polynoidae, based on morphology and DNA. Genetically matches with *Macellicephala* sp. 320 as identified in Bonifácio et al. (2021).

#### Polynoidae sp. (NHM_2101)

4B273D5B-90C2-55D6-AFE6-C98445B3C3EA

##### Materials

- **Type status:** Other material. **Occurrence:** catalogNumber: NHMUK ANEA 2023.535; recordNumber: NHM_2185; recordedBy: Adrian Glover | Helena Wiklund | Thomas Dahlgren | Madeleine Brasier; individualCount: 1; preparations: specimen stored in 80% non-denatured ethanol aqueous solution | DNA voucher stored in buffer; otherCatalogNumbers: 0174126223; associatedSequences: OQ746760 (16S); occurrenceID: 65397203-5DB9-52B3-8C16-7C18436D656B; **Taxon:** taxonConceptID: Polynoidae sp. (NHM_2101); scientificName: Polynoidae; kingdom: Animalia; phylum: Annelida; class: Polychaeta; order: Phyllodocida; family: Polynoidae; taxonRank: family; scientificNameAuthorship: Kinberg, 1856; **Location:** waterBody: Pacific; stateProvince: Clarion Clipperton Zone; locality: Area of Particular Interest APEI-6; verbatimLocality: APEI-6; maximumDepthInMeters: 4115; locationRemarks: Deployment BC29; at Station A02; from R/V Thomas G. Thompson Cruise no. TN319; verbatimLatitude: 19 28.342; verbatimLongitude: 120 11.495; decimalLatitude: 19.47237; decimalLongitude: -120.19158; geodeticDatum: WGS84; **Identification:** identifiedBy: Helena Wiklund | Lenka Neal | Thomas Dahlgren | Adrian Glover | Madeleine Brasier | Regan Drennan | Eva Stewart; dateIdentified: 2021-04-20; identificationRemarks: identified by DNA and morphology; **Event:** eventID: APEI6_AB02_BC29; samplingProtocol: USNEL Box Core; eventDate: 2015-03-22; eventTime: 02:19; habitat: Abyssal plain; fieldNotes: Collected from 0-2 cm layer of box core using a 300 micron sieve; **Record Level:** language: en; institutionCode: NHMUK; collectionCode: ZOO; datasetName: ABYSSLINE; basisOfRecord: PreservedSpecimen
- **Type status:** Other material. **Occurrence:** catalogNumber: NHMUK ANEA 2023.534; recordNumber: NHM_2101; recordedBy: Adrian Glover | Helena Wiklund | Thomas Dahlgren | Madeleine Brasier; individualCount: 1; preparations: specimen stored in 80% non-denatured ethanol aqueous solution | DNA voucher stored in buffer; otherCatalogNumbers: 0174126747; associatedSequences: OQ746755 (16S) | OQ746908 (18S) | OQ738608 (COI); occurrenceID: 9AA5CA8B-06CE-5248-8542-19F3CA5BCCFF; **Taxon:** taxonConceptID: Polynoidae sp. (NHM_2101); scientificName: Polynoidae; kingdom: Animalia; phylum: Annelida; class: Polychaeta; order: Phyllodocida; family: Polynoidae; taxonRank: family; scientificNameAuthorship: Kinberg, 1856; **Location:** waterBody: Pacific; stateProvince: Clarion Clipperton Zone; locality: Area of Particular Interest APEI-6; verbatimLocality: APEI-6; maximumDepthInMeters: 4026; locationRemarks: Deployment EB13; at Station APEI; from R/V Thomas G. Thompson Cruise no. TN319; verbatimLatitude: 19 27.874; verbatimLongitude: 120 01.525; decimalLatitude: 19.46457; decimalLongitude: -120.02542; geodeticDatum: WGS84; **Identification:** identifiedBy: Helena Wiklund | Lenka Neal | Thomas Dahlgren | Adrian Glover | Madeleine Brasier | Regan Drennan | Eva Stewart; dateIdentified: 2021-04-20; identificationRemarks: identified by DNA and morphology; **Event:** eventID: APEI6_AB02_EB13; samplingProtocol: Brenke Epibenthic Sledge; eventDate: 2015-03-20; eventTime: 16:12; habitat: Abyssal plain; fieldNotes: Collected from epi net (on the epibenthic sledge); **Record Level:** language: en; institutionCode: NHMUK; collectionCode: ZOO; datasetName: ABYSSLINE; basisOfRecord: PreservedSpecimen

##### Distribution

Eastern Clarion-Clipperton Zone, central Pacific Ocean.

##### Diagnosis

**Figure 97.**
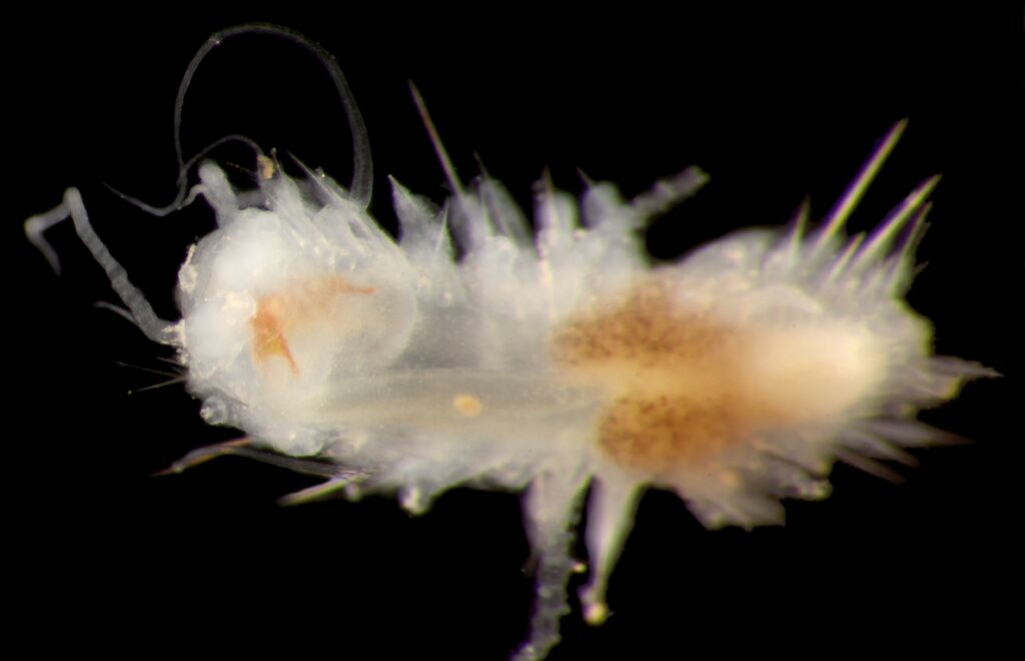
Polynoidae sp. (NHM_2101), live specimen NHM_2101 in dorsal view.

Damaged specimens (Fig. 97) consistent with placement within family Polynoidae, based on morphology and DNA.

#### Polynoidae sp. (NHM_589)

D1918DC0-1D42-5E3B-9B79-AD2A2F17D84A

##### Materials

- **Type status:** Other material. **Occurrence:** catalogNumber: NHMUK ANEA 2023.539; recordNumber: NHM_0589; recordedBy: Adrian Glover | Helena Wiklund | Thomas Dahlgren | Madeleine Brasier; individualCount: 1; preparations: specimen stored in 80% non-denatured ethanol aqueous solution | DNA voucher stored in buffer; otherCatalogNumbers: 0174126575; associatedSequences: OQ746532 (16S) | OQ746846 (18S); occurrenceID: F18909B1-77E9-57F9-A777-96DB6D918219; **Taxon:** taxonConceptID: Polynoidae sp. (NHM_589); scientificName: Polynoidae; kingdom: Animalia; phylum: Annelida; class: Polychaeta; order: Phyllodocida; family: Polynoidae; taxonRank: family; scientificNameAuthorship: Kinberg, 1856; **Location:** waterBody: Pacific; stateProvince: Clarion Clipperton Zone; locality: UK Seabed Resources Ltd exploration area UK-1 Stratum B; verbatimLocality: UK1 Stratum B; maximumDepthInMeters: 4202; locationRemarks: Deployment EB01; at Station U2; from R/V Thomas G. Thompson Cruise no. TN319; verbatimLatitude: 12'23.17456; verbatimLongitude: 116'32.92021; decimalLatitude: 12.38624; decimalLongitude: -116.54867; geodeticDatum: WGS84; **Identification:** identifiedBy: Helena Wiklund | Lenka Neal | Thomas Dahlgren | Adrian Glover | Madeleine Brasier | Regan Drennan | Eva Stewart; dateIdentified: 2021-04-20; identificationRemarks: identified by DNA and morphology; **Event:** eventID: UK1_AB02_EB01; samplingProtocol: Brenke Epibenthic Sledge; eventDate: 2015-02-17; eventTime: 05:15; habitat: Abyssal plain; fieldNotes: Collected from epi net (on the epibenthic sledge); **Record Level:** language: en; institutionCode: NHMUK; collectionCode: ZOO; datasetName: ABYSSLINE; basisOfRecord: PreservedSpecimen

##### Distribution

Eastern Clarion-Clipperton Zone, central Pacific Ocean.

##### Diagnosis

**Figure 98.**
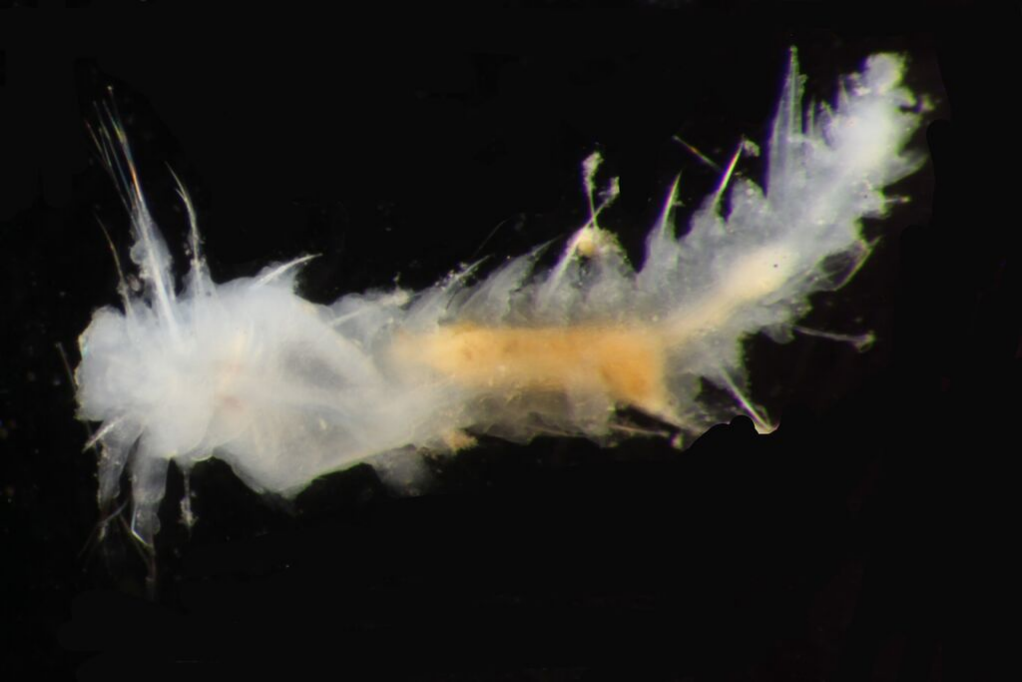
Polynoidae sp. (NHM_589), live specimen NHM_589 in dorsal view.

Damaged specimen (Fig. 98) consistent with placement within family Polynoidae, based on morphology and DNA.

#### Polynoidae sp. (NHM_690)

F978C05F-F6DD-5808-99F1-918BC07D0412

##### Materials

- **Type status:** Other material. **Occurrence:** catalogNumber: NHMUK ANEA 2023.540; recordNumber: NHM_0690; recordedBy: Adrian Glover | Helena Wiklund | Thomas Dahlgren | Madeleine Brasier; individualCount: 1; preparations: specimen stored in 80% non-denatured ethanol aqueous solution | DNA voucher stored in buffer; otherCatalogNumbers: 0174126550; associatedSequences: OQ746540 (16S) | OQ746851 (18S); occurrenceID: 885EB06E-AE2B-5A1E-B54D-61760A4ED81C; **Taxon:** taxonConceptID: Polynoidae sp. (NHM_690); scientificName: Polynoidae; kingdom: Animalia; phylum: Annelida; class: Polychaeta; order: Phyllodocida; family: Polynoidae; taxonRank: family; scientificNameAuthorship: Kinberg, 1856; **Location:** waterBody: Pacific; stateProvince: Clarion Clipperton Zone; locality: UK Seabed Resources Ltd exploration area UK-1 Stratum B; verbatimLocality: UK1 Stratum B; maximumDepthInMeters: 4425; locationRemarks: Deployment EB02; at Station U5; from R/V Thomas G. Thompson Cruise no. TN319; verbatimLatitude: 12'32.23; verbatimLongitude: 116'36.25; decimalLatitude: 12.53717; decimalLongitude: -116.60417; geodeticDatum: WGS84; **Identification:** identifiedBy: Helena Wiklund | Lenka Neal | Thomas Dahlgren | Adrian Glover | Madeleine Brasier | Regan Drennan | Eva Stewart; dateIdentified: 2021-04-20; identificationRemarks: identified by DNA and morphology; **Event:** eventID: UK1_AB02_EB02; samplingProtocol: Brenke Epibenthic Sledge; eventDate: 2015-02-20; eventTime: 06:24; habitat: Abyssal plain; fieldNotes: Collected from epi net (on the epibenthic sledge); **Record Level:** language: en; institutionCode: NHMUK; collectionCode: ZOO; datasetName: ABYSSLINE; basisOfRecord: PreservedSpecimen

##### Distribution

Eastern Clarion-Clipperton Zone, central Pacific Ocean.

##### Diagnosis

**Figure 99.**
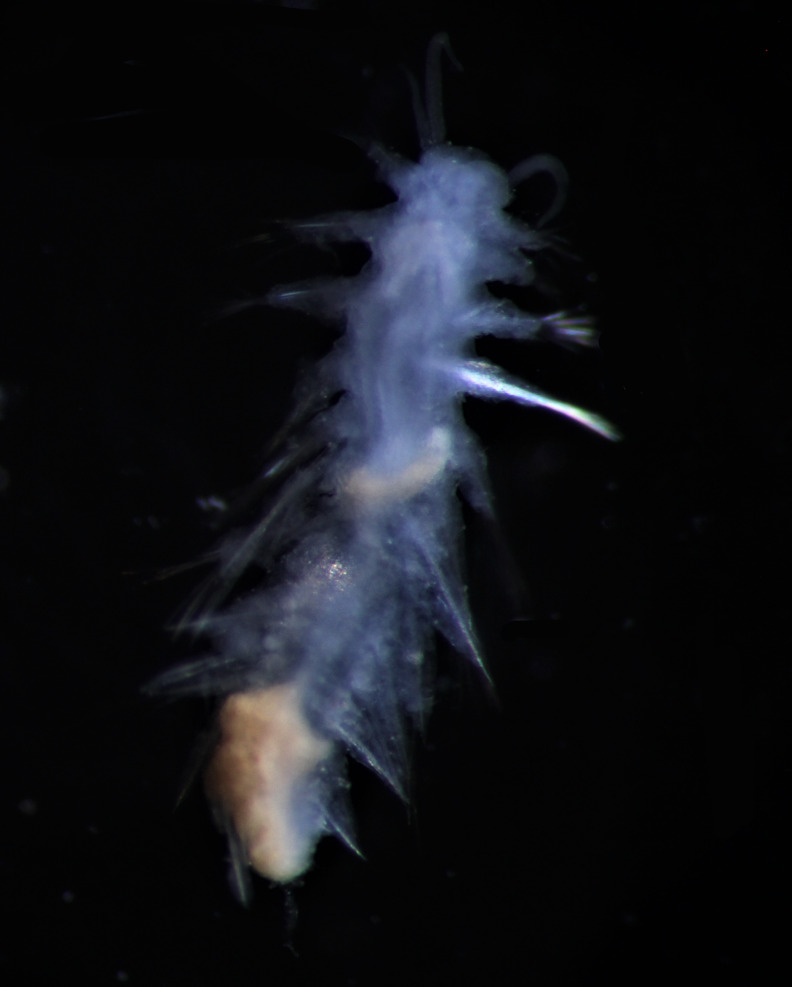
Polynoidae sp. (NHM_690), live specimen NHM_690 in dorsal view.

Damaged specimen (Fig. 99) consistent with placement within family Polynoidae, based on morphology and DNA.

#### Polynoidae sp. (NHM_1074)

8D6649EB-4FE5-5587-BF9A-A4D2D7620FA3

##### Materials

- **Type status:** Other material. **Occurrence:** catalogNumber: NHMUK ANEA 2023.529; recordNumber: NHM_1074; recordedBy: Adrian Glover | Helena Wiklund | Thomas Dahlgren | Madeleine Brasier; individualCount: 1; preparations: specimen stored in 80% non-denatured ethanol aqueous solution | DNA voucher stored in buffer; otherCatalogNumbers: 0174127356; associatedSequences: OQ746613 (16S) | OQ746873 (18S); occurrenceID: C57E7401-97C1-57A6-A742-3BF90E139F5C; **Taxon:** taxonConceptID: Polynoidae sp. (NHM_1074); scientificName: Polynoidae; kingdom: Animalia; phylum: Annelida; class: Polychaeta; order: Phyllodocida; family: Polynoidae; taxonRank: family; scientificNameAuthorship: Kinberg, 1856; **Location:** waterBody: Pacific; stateProvince: Clarion Clipperton Zone; locality: Ocean Mineral Singapore exploration claim Stratum A; verbatimLocality: OMS Stratum A; maximumDepthInMeters: 4100; locationRemarks: Deployment EB05; at Station S2; from R/V Thomas G. Thompson Cruise no. TN319; verbatimLatitude: 12'06.93; verbatimLongitude: 117'09.87; decimalLatitude: 12.1155; decimalLongitude: -117.1645; geodeticDatum: WGS84; **Identification:** identifiedBy: Helena Wiklund | Lenka Neal | Thomas Dahlgren | Adrian Glover | Madeleine Brasier | Regan Drennan | Eva Stewart; dateIdentified: 2021-04-20; identificationRemarks: identified by DNA and morphology; **Event:** eventID: OMS1_AB02_EB05; samplingProtocol: Brenke Epibenthic Sledge; eventDate: 2015-02-26; eventTime: 21:29; habitat: Abyssal plain; fieldNotes: Collected from epi net (on the epibenthic sledge); **Record Level:** language: en; institutionCode: NHMUK; collectionCode: ZOO; datasetName: ABYSSLINE; basisOfRecord: PreservedSpecimen

##### Distribution

Eastern Clarion-Clipperton Zone, central Pacific Ocean.

##### Diagnosis

**Figure 100.**
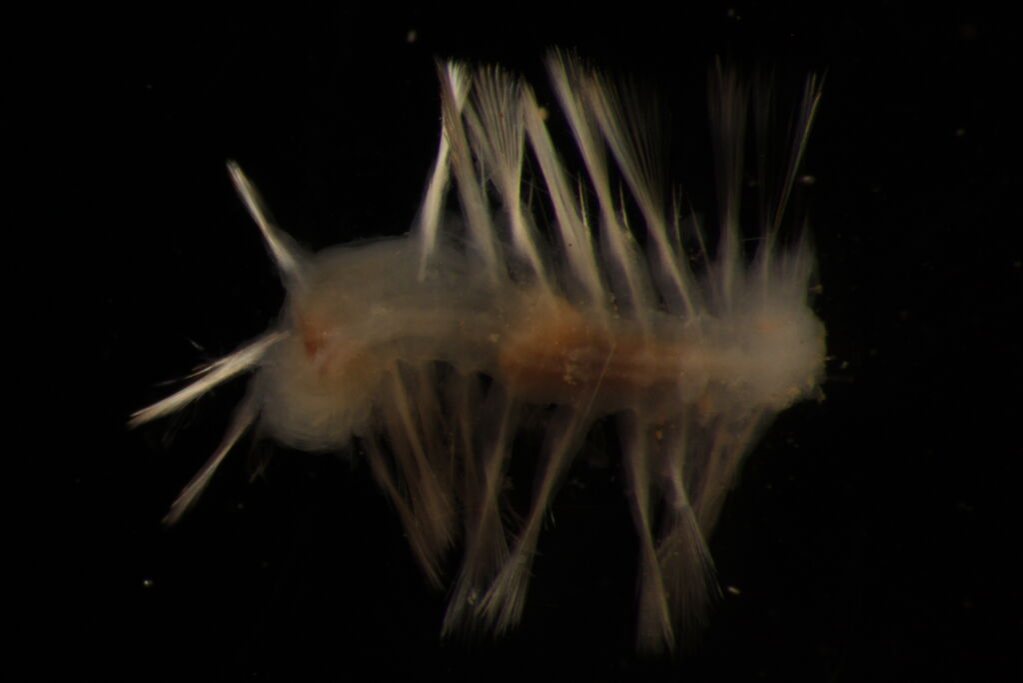
Polynoidae sp. (NHM_1074), live specimen NHM_1074 in ventral view.

Damaged specimen (Fig. 100) consistent with placement within family Polynoidae, based on morphology and DNA.

#### Polynoidae sp. (NHM_747A)

50393E88-422D-5E44-9DD3-FA1C214181BA

##### Materials

- **Type status:** Other material. **Occurrence:** catalogNumber: NHMUK ANEA 2023.541; recordNumber: NHM_0747A; recordedBy: Adrian Glover | Helena Wiklund | Thomas Dahlgren | Madeleine Brasier; individualCount: 1; preparations: specimen stored in 80% non-denatured ethanol aqueous solution | DNA voucher stored in buffer; otherCatalogNumbers: 0174126602; associatedSequences: OQ746548 (16S) | OQ746854 (18S); occurrenceID: D8437025-A5BA-5AC8-BD21-0C91ADCD3BCE; **Taxon:** taxonConceptID: Polynoidae sp. (NHM_747A); scientificName: Polynoidae; kingdom: Animalia; phylum: Annelida; class: Polychaeta; order: Phyllodocida; family: Polynoidae; taxonRank: family; scientificNameAuthorship: Kinberg, 1856; **Location:** waterBody: Pacific; stateProvince: Clarion Clipperton Zone; locality: UK Seabed Resources Ltd exploration area UK-1 Stratum B; verbatimLocality: UK1 Stratum B; maximumDepthInMeters: 4425; locationRemarks: Deployment EB02; at Station U5; from R/V Thomas G. Thompson Cruise no. TN319; verbatimLatitude: 12'32.23; verbatimLongitude: 116'36.25; decimalLatitude: 12.53717; decimalLongitude: -116.60417; geodeticDatum: WGS84; **Identification:** identifiedBy: Helena Wiklund | Lenka Neal | Thomas Dahlgren | Adrian Glover | Madeleine Brasier | Regan Drennan | Eva Stewart; dateIdentified: 2021-04-20; identificationRemarks: identified by DNA and morphology; **Event:** eventID: UK1_AB02_EB02; samplingProtocol: Brenke Epibenthic Sledge; eventDate: 2015-02-20; eventTime: 06:24; habitat: Abyssal plain; fieldNotes: Collected from epi net (on the epibenthic sledge); **Record Level:** language: en; institutionCode: NHMUK; collectionCode: ZOO; datasetName: ABYSSLINE; basisOfRecord: PreservedSpecimen

##### Distribution

Eastern Clarion-Clipperton Zone, central Pacific Ocean.

##### Diagnosis

Damaged specimen consistent with placement within family Polynoidae, based on DNA data.

#### Polynoidae sp. (NHM_2195)

D4B33426-6771-5CA0-BBE0-882D9597207B

##### Materials

- **Type status:** Other material. **Occurrence:** catalogNumber: NHMUK ANEA 2023.536; recordNumber: NHM_2195; recordedBy: Adrian Glover | Helena Wiklund | Thomas Dahlgren | Madeleine Brasier; individualCount: 1; preparations: specimen stored in 80% non-denatured ethanol aqueous solution | DNA voucher stored in buffer; otherCatalogNumbers: 0174126807; associatedSequences: OQ746763 (16S); occurrenceID: 00460F3F-74A5-5D99-A712-251DB68FBF0D; **Taxon:** taxonConceptID: Polynoidae sp. (NHM_2195); scientificName: Polynoidae; kingdom: Animalia; phylum: Annelida; class: Polychaeta; order: Phyllodocida; family: Polynoidae; taxonRank: family; scientificNameAuthorship: Kinberg, 1856; **Location:** waterBody: Pacific; stateProvince: Clarion Clipperton Zone; locality: Ocean Mineral Singapore exploration claim Stratum A; verbatimLocality: OMS Stratum A; maximumDepthInMeters: 4122; locationRemarks: Deployment EB04; at Station S1; from R/V Thomas G. Thompson Cruise no. TN319; verbatimLatitude: 12'08.02; verbatimLongitude: 117'17.52; decimalLatitude: 12.13367; decimalLongitude: -117.292; geodeticDatum: WGS84; **Identification:** identifiedBy: Helena Wiklund | Lenka Neal | Thomas Dahlgren | Adrian Glover | Madeleine Brasier | Regan Drennan | Eva Stewart; dateIdentified: 2021-04-20; identificationRemarks: identified by DNA and morphology; **Event:** eventID: OMS1_AB02_EB04; samplingProtocol: Brenke Epibenthic Sledge; eventDate: 2015-02-24; eventTime: 19:10; habitat: Abyssal plain; fieldNotes: Collected from supra net (on the epibenthic sledge); **Record Level:** language: en; institutionCode: NHMUK; collectionCode: ZOO; datasetName: ABYSSLINE; basisOfRecord: PreservedSpecimen
- **Type status:** Other material. **Occurrence:** catalogNumber: NHMUK ANEA 2023.537; recordNumber: NHM_2520; recordedBy: Adrian Glover | Helena Wiklund | Thomas Dahlgren | Madeleine Brasier; individualCount: 1; preparations: specimen stored in 80% non-denatured ethanol aqueous solution | DNA voucher stored in buffer; otherCatalogNumbers: 0174126335; associatedSequences: OQ746776 (16S); occurrenceID: B6F1D175-1839-5C0A-BAD3-98186415AF95; **Taxon:** taxonConceptID: Polynoidae sp. (NHM_2195); scientificName: Polynoidae; kingdom: Animalia; phylum: Annelida; class: Polychaeta; order: Phyllodocida; family: Polynoidae; taxonRank: family; scientificNameAuthorship: Kinberg, 1856; **Location:** waterBody: Pacific; stateProvince: Clarion Clipperton Zone; locality: UK Seabed Resources Ltd exploration area UK-1 Stratum B; verbatimLocality: UK1 Stratum B; maximumDepthInMeters: 4137; locationRemarks: Deployment EB07; at Station U7; from R/V Thomas G. Thompson Cruise no. TN319; verbatimLatitude: 12'27.26; verbatimLongitude: 116'36.77; decimalLatitude: 12.45433; decimalLongitude: -116.61283; geodeticDatum: WGS84; **Identification:** identifiedBy: Helena Wiklund | Lenka Neal | Thomas Dahlgren | Adrian Glover | Madeleine Brasier | Regan Drennan | Eva Stewart; dateIdentified: 2021-04-20; identificationRemarks: identified by DNA and morphology; **Event:** eventID: UK1_AB02_EB07; samplingProtocol: Brenke Epibenthic Sledge; eventDate: 2015-03-03; eventTime: 20:40; habitat: Abyssal plain; fieldNotes: Collected from supra net (on the epibenthic sledge); **Record Level:** language: en; institutionCode: NHMUK; collectionCode: ZOO; datasetName: ABYSSLINE; basisOfRecord: PreservedSpecimen

##### Distribution

Eastern Clarion-Clipperton Zone, central Pacific Ocean.

##### Diagnosis

Damaged specimens consistent with placement within family Polynoidae, based on DNA data.

### Sabellariidae Johnston, 1865

#### Sabellaridae sp. (NHM_1167B)

BEE552A3-9F4E-52B5-B2F0-060848721712

##### Materials

- **Type status:** Other material. **Occurrence:** catalogNumber: NHMUK ANEA 2023.556; recordNumber: NHM_1351B; recordedBy: Adrian Glover | Helena Wiklund | Thomas Dahlgren | Madeleine Brasier; individualCount: 1; preparations: specimen stored in 80% non-denatured ethanol aqueous solution | DNA voucher stored in buffer; otherCatalogNumbers: 0174126539; associatedSequences: OQ746674 (16S); occurrenceID: 7883076B-003B-5E56-B6BC-74A8AFEBA02B; **Taxon:** taxonConceptID: Sabellariidae sp. (NHM_1167B); scientificName: Sabellariidae; kingdom: Animalia; phylum: Annelida; class: Polychaeta; family: Sabellariidae; taxonRank: family; scientificNameAuthorship: Johnston, 1865; **Location:** waterBody: Pacific; stateProvince: Clarion Clipperton Zone; locality: Ocean Mineral Singapore exploration claim Stratum A; verbatimLocality: OMS Stratum A; maximumDepthInMeters: 4302; locationRemarks: Deployment EB06; at Station S5; from R/V Thomas G. Thompson Cruise no. TN319; verbatimLatitude: 12'15.44; verbatimLongitude: 117'18.13; decimalLatitude: 12.25733; decimalLongitude: -117.30217; geodeticDatum: WGS84; **Identification:** identifiedBy: Helena Wiklund | Lenka Neal | Thomas Dahlgren | Adrian Glover | Madeleine Brasier | Regan Drennan | Eva Stewart; dateIdentified: 2021-04-20; identificationRemarks: identified by DNA and morphology; **Event:** eventID: OMS1_AB02_EB06; samplingProtocol: Brenke Epibenthic Sledge; eventDate: 2015-03-01; eventTime: 04:02; habitat: Abyssal plain; fieldNotes: Collected from epi net (on the epibenthic sledge); **Record Level:** language: en; institutionCode: NHMUK; collectionCode: ZOO; datasetName: ABYSSLINE; basisOfRecord: PreservedSpecimen
- **Type status:** Other material. **Occurrence:** catalogNumber: NHMUK ANEA 2023.555; recordNumber: NHM_1167B; recordedBy: Adrian Glover | Helena Wiklund | Thomas Dahlgren | Madeleine Brasier; individualCount: 1; preparations: specimen stored in 80% non-denatured ethanol aqueous solution | DNA voucher stored in buffer; otherCatalogNumbers: 0174126543; associatedSequences: OQ746635 (16S) | OQ746878 (18S); occurrenceID: 7DE4C09D-818B-59A9-91CC-41670BAF8E7C; **Taxon:** taxonConceptID: Sabellariidae sp. (NHM_1167B); scientificName: Sabellariidae; kingdom: Animalia; phylum: Annelida; class: Polychaeta; family: Sabellariidae; taxonRank: family; scientificNameAuthorship: Johnston, 1865; **Location:** waterBody: Pacific; stateProvince: Clarion Clipperton Zone; locality: Ocean Mineral Singapore exploration claim Stratum A; verbatimLocality: OMS Stratum A; maximumDepthInMeters: 4100; locationRemarks: Deployment EB05; at Station S2; from R/V Thomas G. Thompson Cruise no. TN319; verbatimLatitude: 12'06.93; verbatimLongitude: 117'09.87; decimalLatitude: 12.1155; decimalLongitude: -117.1645; geodeticDatum: WGS84; **Identification:** identifiedBy: Helena Wiklund | Lenka Neal | Thomas Dahlgren | Adrian Glover | Madeleine Brasier | Regan Drennan | Eva Stewart; dateIdentified: 2021-04-20; identificationRemarks: identified by DNA and morphology; **Event:** eventID: OMS1_AB02_EB05; samplingProtocol: Brenke Epibenthic Sledge; eventDate: 2015-02-26; eventTime: 21:29; habitat: Abyssal plain; fieldNotes: Collected from epi net (on the epibenthic sledge); **Record Level:** language: en; institutionCode: NHMUK; collectionCode: ZOO; datasetName: ABYSSLINE; basisOfRecord: PreservedSpecimen
- **Type status:** Other material. **Occurrence:** catalogNumber: NHMUK ANEA 2023.557; recordNumber: NHM_2523; recordedBy: Adrian Glover | Helena Wiklund | Thomas Dahlgren | Madeleine Brasier; individualCount: 1; preparations: specimen stored in 80% non-denatured ethanol aqueous solution | DNA voucher stored in buffer; otherCatalogNumbers: 0174126180; associatedSequences: OQ746777 (16S); occurrenceID: 2512A3DA-5D1E-580D-9E57-177BE1C5D98C; **Taxon:** taxonConceptID: Sabellariidae sp. (NHM_1167B); scientificName: Sabellariidae; kingdom: Animalia; phylum: Annelida; class: Polychaeta; family: Sabellariidae; taxonRank: family; scientificNameAuthorship: Johnston, 1865; **Location:** waterBody: Pacific; stateProvince: Clarion Clipperton Zone; locality: UK Seabed Resources Ltd exploration area UK-1 Stratum B; verbatimLocality: UK1 Stratum B; maximumDepthInMeters: 4137; locationRemarks: Deployment EB07; at Station U7; from R/V Thomas G. Thompson Cruise no. TN319; verbatimLatitude: 12'27.26; verbatimLongitude: 116'36.77; decimalLatitude: 12.45433; decimalLongitude: -116.61283; geodeticDatum: WGS84; **Identification:** identifiedBy: Helena Wiklund | Lenka Neal | Thomas Dahlgren | Adrian Glover | Madeleine Brasier | Regan Drennan | Eva Stewart; dateIdentified: 2021-04-20; identificationRemarks: identified by DNA and morphology; **Event:** eventID: UK1_AB02_EB07; samplingProtocol: Brenke Epibenthic Sledge; eventDate: 2015-03-03; eventTime: 20:40; habitat: Abyssal plain; fieldNotes: Collected from supra net (on the epibenthic sledge); **Record Level:** language: en; institutionCode: NHMUK; collectionCode: ZOO; datasetName: ABYSSLINE; basisOfRecord: PreservedSpecimen
- **Type status:** Other material. **Occurrence:** catalogNumber: NHMUK ANEA 2023.554; recordNumber: NHM_0957A; recordedBy: Adrian Glover | Helena Wiklund | Thomas Dahlgren | Madeleine Brasier; individualCount: 1; preparations: specimen stored in 80% non-denatured ethanol aqueous solution | DNA voucher stored in buffer; otherCatalogNumbers: 0174126617; associatedSequences: OQ746597 (16S); occurrenceID: B8AF60B6-6138-5E13-AD57-170063C89E20; **Taxon:** taxonConceptID: Sabellariidae sp. (NHM_1167B); scientificName: Sabellariidae; kingdom: Animalia; phylum: Annelida; class: Polychaeta; family: Sabellariidae; taxonRank: family; scientificNameAuthorship: Johnston, 1865; **Location:** waterBody: Pacific; stateProvince: Clarion Clipperton Zone; locality: UK Seabed Resources Ltd exploration area UK-1 Stratum B; verbatimLocality: UK1 Stratum B; maximumDepthInMeters: 4198; locationRemarks: Deployment EB03; at Station U4; from R/V Thomas G. Thompson Cruise no. TN319; verbatimLatitude: 12'34.28; verbatimLongitude: 116'36.63; decimalLatitude: 12.57133; decimalLongitude: -116.6105; geodeticDatum: WGS84; **Identification:** identifiedBy: Helena Wiklund | Lenka Neal | Thomas Dahlgren | Adrian Glover | Madeleine Brasier | Regan Drennan | Eva Stewart; dateIdentified: 2021-04-20; identificationRemarks: identified by DNA and morphology; **Event:** eventID: UK1_AB02_EB03; samplingProtocol: Brenke Epibenthic Sledge; eventDate: 2015-02-23; eventTime: 05:39; habitat: Abyssal plain; fieldNotes: Collected from epi net (on the epibenthic sledge); **Record Level:** language: en; institutionCode: NHMUK; collectionCode: ZOO; datasetName: ABYSSLINE; basisOfRecord: PreservedSpecimen

##### Distribution

Eastern Clarion-Clipperton Zone, central Pacific Ocean.

##### Diagnosis

**Figure 101.**
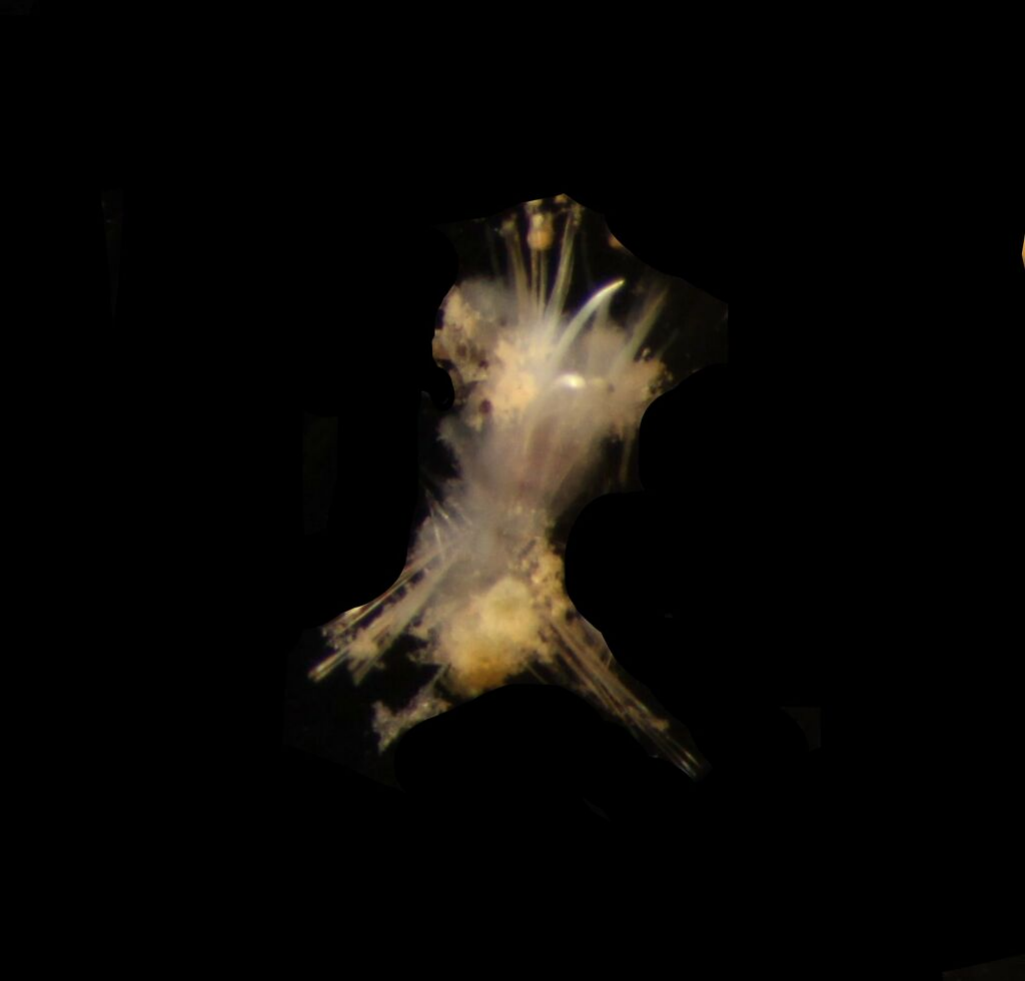
Sabellaridae sp. (NHM_1167B), anterior fragment of live specimen NHM_1167B.

Damaged specimens (Fig. 101) consistent with placement within family Sabellaridae, based on morphology and DNA.

### Sabellidae Latreille, 1825

#### Sabellidae sp. (NHM_189)

58391E56-573E-5607-83A1-68A3382F2700

##### Materials

- **Type status:** Other material. **Occurrence:** recordNumber: NHM_0189; recordedBy: Adrian Glover | Helena Wiklund | Thomas Dahlgren | Magdalena Georgieva; individualCount: 1; preparations: Tissue voucher stored in 80% non-denatured ethanol aqueous solution | DNA voucher stored in buffer; otherCatalogNumbers: 0109405350 | 0174127339; associatedSequences: OQ746485 (16S) | OQ738506 (COI); occurrenceID: EA7AB927-B73D-5FCA-9553-4C1DB3CDD29C; **Taxon:** taxonConceptID: Sabellidae sp. (NHM_189); scientificName: Sabellidae; kingdom: Animalia; phylum: Annelida; class: Polychaeta; order: Sabellida; family: Sabellidae; taxonRank: family; scientificNameAuthorship: Latreille, 1825; **Location:** waterBody: Pacific; stateProvince: Clarion Clipperton Zone; locality: UK Seabed Resources Ltd exploration area UK-1 Stratum A; verbatimLocality: UK1 Stratum A; maximumDepthInMeters: 4082; locationRemarks: Deployment EB03; at Station D; from R/V Melville Cruise no. MV1313; verbatimLatitude: 13°56.089; verbatimLongitude: 116°33.011; decimalLatitude: 13.93482; decimalLongitude: -116.55018; geodeticDatum: WGS84; **Identification:** identifiedBy: Helena Wiklund | Lenka Neal | Thomas Dahlgren | Adrian Glover | Madeleine Brasier | Regan Drennan | Eva Stewart; dateIdentified: 2021-04-20; identificationRemarks: identified by DNA and morphology; **Event:** eventID: UK1_AB01_EB03; samplingProtocol: Brenke Epibenthic Sledge; eventDate: 2013-10-13; eventTime: 12:40; habitat: Abyssal plain; fieldNotes: Collected from epi net (on the epibenthic sledge); **Record Level:** language: en; institutionCode: NHMUK; collectionCode: ZOO; datasetName: ABYSSLINE; basisOfRecord: PreservedSpecimen

##### Distribution

Eastern Clarion-Clipperton Zone, central Pacific Ocean.

##### Diagnosis

**Figure 102.**
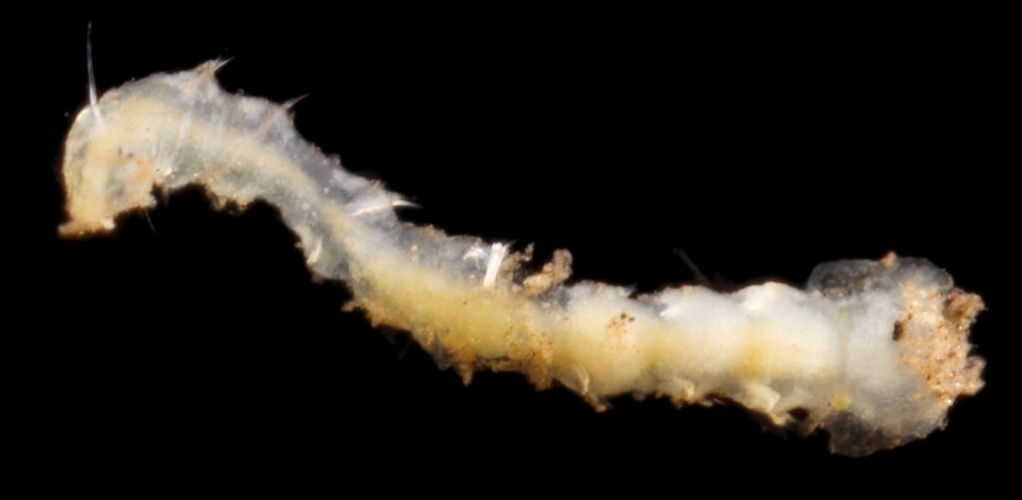
Sabellidae sp. (NHM_189), live specimen NHM_189, radiolar crown missing.

Damaged specimens (Fig. 102) consistent with placement within family Sabellidae, based on morphology and DNA.

#### Sabellidae sp. (NHM_351)

5BEB254D-32D5-5433-B34E-F55BCAEE1F3F

##### Materials

- **Type status:** Other material. **Occurrence:** catalogNumber: NHMUK ANEA 2023.562; recordNumber: NHM_0351; recordedBy: Adrian Glover | Helena Wiklund | Thomas Dahlgren | Magdalena Georgieva; individualCount: 1; preparations: specimen stored in 80% non-denatured ethanol aqueous solution | DNA voucher stored in buffer; otherCatalogNumbers: 0174127351; associatedSequences: OQ746503 (16S); occurrenceID: CD80AB7C-07FA-58E4-9BDB-B54B2F5A045C; **Taxon:** taxonConceptID: Sabellidae sp. (NHM_351); scientificName: Sabellidae; kingdom: Animalia; phylum: Annelida; class: Polychaeta; order: Sabellida; family: Sabellidae; taxonRank: family; scientificNameAuthorship: Latreille, 1825; **Location:** waterBody: Pacific; stateProvince: Clarion Clipperton Zone; locality: UK Seabed Resources Ltd exploration area UK-1 Stratum A; verbatimLocality: UK1 Stratum A; maximumDepthInMeters: 4150; locationRemarks: Deployment BC11; at Station H; from R/V Melville Cruise no. MV1313; verbatimLatitude: 13°53.300; verbatimLongitude: 116°41.399; decimalLatitude: 13.88833; decimalLongitude: -116.68998; geodeticDatum: WGS84; **Identification:** identifiedBy: Helena Wiklund | Lenka Neal | Thomas Dahlgren | Adrian Glover | Madeleine Brasier | Regan Drennan | Eva Stewart; dateIdentified: 2021-04-20; identificationRemarks: identified by DNA and morphology; **Event:** eventID: UK1_AB01_BC11; samplingProtocol: USNEL Box Core; eventDate: 2013-10-19; eventTime: 02:25; habitat: Abyssal plain; fieldNotes: Collected from 0-2 cm layer of box core using a 300 micron sieve; **Record Level:** language: en; institutionCode: NHMUK; collectionCode: ZOO; datasetName: ABYSSLINE; basisOfRecord: PreservedSpecimen

##### Distribution

Eastern Clarion-Clipperton Zone, central Pacific Ocean.

##### Diagnosis

**Figure 103.**
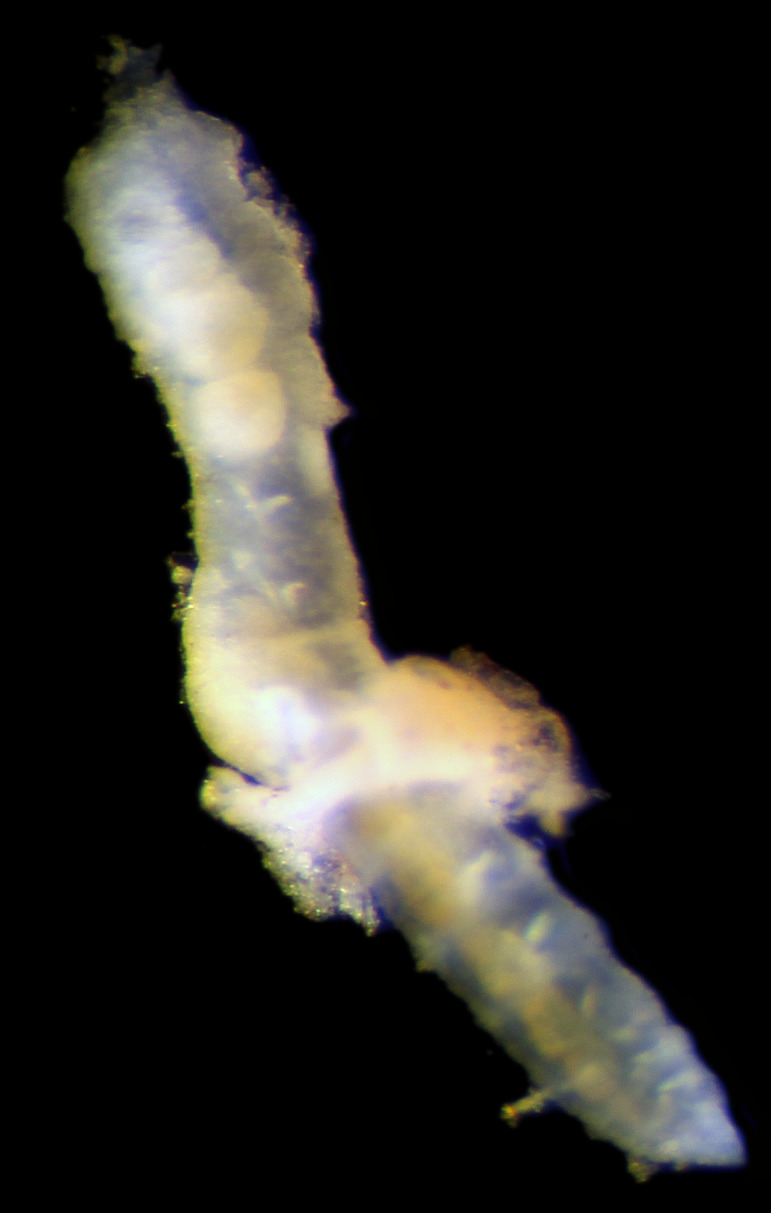
Sabellidae sp. (NHM_351), damaged specimen NHM_351 in dorsolateral view, radiolar crown missing.

Damaged specimen (Fig. 103) consistent with placement within family Sabellidae, based on morphology and DNA.

#### Sabellidae sp. (NHM_535)

1027ADA7-8402-562B-9F03-9C3296F5AE32

##### Materials

- **Type status:** Other material. **Occurrence:** catalogNumber: NHMUK ANEA 2023.563; recordNumber: NHM_0535; recordedBy: Adrian Glover | Helena Wiklund | Thomas Dahlgren | Madeleine Brasier; individualCount: 1; preparations: specimen stored in 80% non-denatured ethanol aqueous solution | DNA voucher stored in buffer; otherCatalogNumbers: 0174126801; associatedSequences: OQ746526 (16S) | OQ746843 (18S) | OQ738525 (COI); occurrenceID: A3F633A4-D509-5AAE-BC24-72EAE57EA38D; **Taxon:** taxonConceptID: Sabellidae sp. (NHM_535); scientificName: Sabellidae; kingdom: Animalia; phylum: Annelida; class: Polychaeta; order: Sabellida; family: Sabellidae; taxonRank: family; scientificNameAuthorship: Latreille, 1825; **Location:** waterBody: Pacific; stateProvince: Clarion Clipperton Zone; locality: UK Seabed Resources Ltd exploration area UK-1 Stratum B; verbatimLocality: UK1 Stratum B; maximumDepthInMeters: 4158; locationRemarks: Deployment BC02; at Station U2; from R/V Thomas G. Thompson Cruise no. TN319; verbatimLatitude: 12'22.020; verbatimLongitude: 116'31.017; decimalLatitude: 12.367; decimalLongitude: -116.51695; geodeticDatum: WGS84; **Identification:** identifiedBy: Helena Wiklund | Lenka Neal | Thomas Dahlgren | Adrian Glover | Madeleine Brasier | Regan Drennan | Eva Stewart; dateIdentified: 2021-04-20; identificationRemarks: identified by DNA and morphology; **Event:** eventID: UK1_AB02_BC02; samplingProtocol: USNEL Box Core; eventDate: 2015-02-17; eventTime: 12:12; habitat: Abyssal plain; fieldNotes: Collected from 0-2 cm layer of box core using a 300 micron sieve; **Record Level:** language: en; institutionCode: NHMUK; collectionCode: ZOO; datasetName: ABYSSLINE; basisOfRecord: PreservedSpecimen

##### Distribution

Eastern Clarion-Clipperton Zone, central Pacific Ocean.

##### Diagnosis

**Figure 104.**
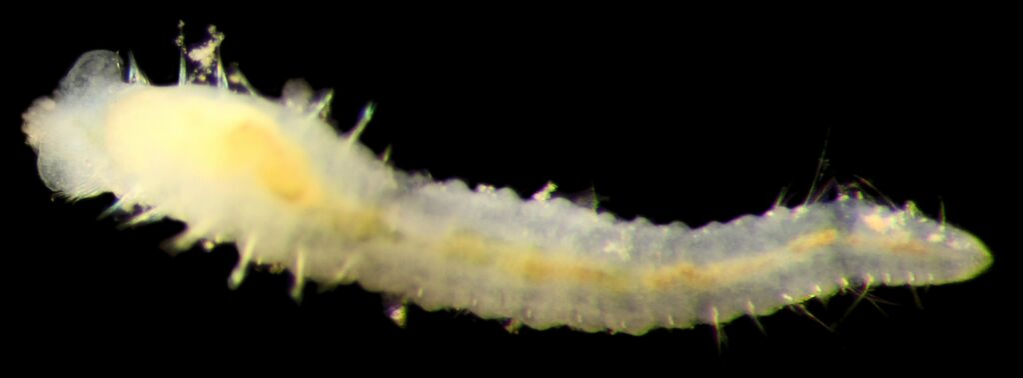
Sabellidae sp. (NHM_535), live specimen NHM_535 in dorsal view, radiolar crown missing.

Damaged specimen (Fig. 104) consistent with placement within family Sabellidae, based on morphology and DNA.

#### Sabellidae sp. (NHM_647)

0E2437EF-7CBC-5213-8A7F-97E1DB7BCDBE

##### Materials

- **Type status:** Other material. **Occurrence:** catalogNumber: NHMUK ANEA 2023.564; recordNumber: NHM_0647; recordedBy: Adrian Glover | Helena Wiklund | Thomas Dahlgren | Madeleine Brasier; individualCount: 1; preparations: specimen stored in 80% non-denatured ethanol aqueous solution | DNA voucher stored in buffer; otherCatalogNumbers: 0174126577; associatedSequences: OQ746537 (16S) | OQ746849 (18S); occurrenceID: D5A2B660-57E2-5A95-8F46-A6D11F59D6A0; **Taxon:** taxonConceptID: Sabellidae sp. (NHM_647); scientificName: Sabellidae; kingdom: Animalia; phylum: Annelida; class: Polychaeta; order: Sabellida; family: Sabellidae; taxonRank: family; scientificNameAuthorship: Latreille, 1825; **Location:** waterBody: Pacific; stateProvince: Clarion Clipperton Zone; locality: UK Seabed Resources Ltd exploration area UK-1 Stratum B; verbatimLocality: UK1 Stratum B; maximumDepthInMeters: 4202; locationRemarks: Deployment EB01; at Station U2; from R/V Thomas G. Thompson Cruise no. TN319; verbatimLatitude: 12'23.17456; verbatimLongitude: 116'32.92021; decimalLatitude: 12.38624; decimalLongitude: -116.54867; geodeticDatum: WGS84; **Identification:** identifiedBy: Helena Wiklund | Lenka Neal | Thomas Dahlgren | Adrian Glover | Madeleine Brasier | Regan Drennan | Eva Stewart; dateIdentified: 2021-04-20; identificationRemarks: identified by DNA and morphology; **Event:** eventID: UK1_AB02_EB01; samplingProtocol: Brenke Epibenthic Sledge; eventDate: 2015-02-17; eventTime: 05:15; habitat: Abyssal plain; fieldNotes: Collected from epi net (on the epibenthic sledge); **Record Level:** language: en; institutionCode: NHMUK; collectionCode: ZOO; datasetName: ABYSSLINE; basisOfRecord: PreservedSpecimen

##### Distribution

Eastern Clarion-Clipperton Zone, central Pacific Ocean.

##### Diagnosis

**Figure 105.**
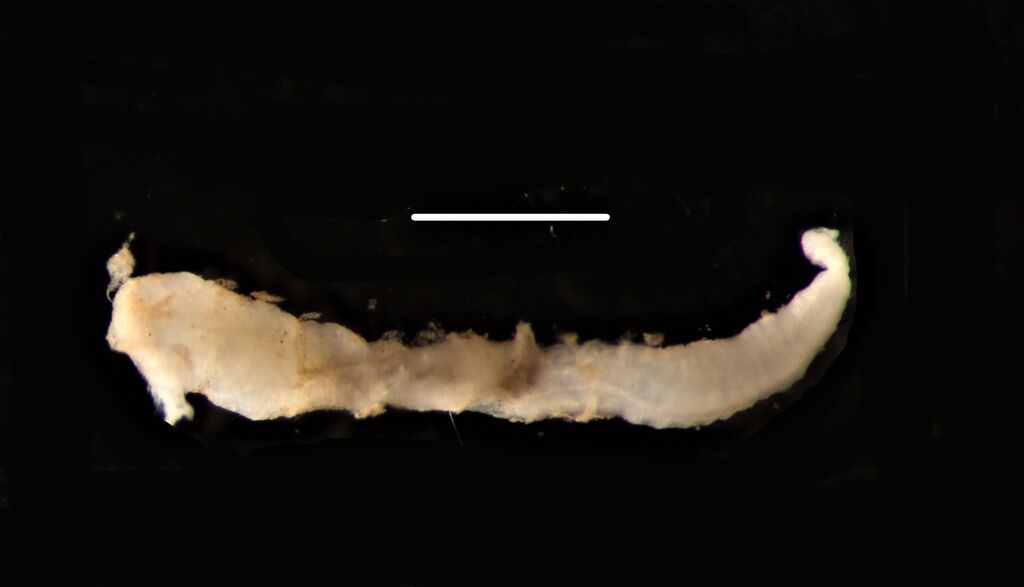
Sabellidae sp. (NHM_647), fragment of a preserved specimen NHM_647.

Damaged specimen (Fig. 105) consistent with placement within family Sabellidae, based on morphology and DNA.

#### Sabellidae sp. (NHM_915D)

7C55BC1C-C4C8-5199-82DB-430017F78E30

##### Materials

- **Type status:** Other material. **Occurrence:** recordNumber: NHM_1739; recordedBy: Adrian Glover | Helena Wiklund | Thomas Dahlgren | Madeleine Brasier; individualCount: 1; preparations: DNA voucher stored in buffer; otherCatalogNumbers: 0174126775; associatedSequences: OQ746709 (16S) | OQ746899 (18S); occurrenceID: 7D1506E6-690E-517D-A908-D3A5309C73EE; **Taxon:** taxonConceptID: Sabellidae sp. (NHM_915D); scientificName: Sabellidae; kingdom: Animalia; phylum: Annelida; class: Polychaeta; order: Sabellida; family: Sabellidae; taxonRank: family; scientificNameAuthorship: Latreille, 1825; **Location:** waterBody: Pacific; stateProvince: Clarion Clipperton Zone; locality: Ocean Mineral Singapore exploration claim Stratum A; verbatimLocality: OMS Stratum A; maximumDepthInMeters: 4045; locationRemarks: Deployment EB10; at Station S7; from R/V Thomas G. Thompson Cruise no. TN319; verbatimLatitude: 12'10.43; verbatimLongitude: 117'11.57; decimalLatitude: 12.17383; decimalLongitude: -117.19283; geodeticDatum: WGS84; **Identification:** identifiedBy: Helena Wiklund | Lenka Neal | Thomas Dahlgren | Adrian Glover | Madeleine Brasier | Regan Drennan | Eva Stewart; dateIdentified: 2021-04-20; identificationRemarks: identified by DNA and morphology; **Event:** eventID: OMS1_AB02_EB10; samplingProtocol: Brenke Epibenthic Sledge; eventDate: 2015-03-11; eventTime: 22:49; habitat: Abyssal plain; fieldNotes: Collected from epi net (on the epibenthic sledge); **Record Level:** language: en; institutionCode: NHMUK; collectionCode: ZOO; datasetName: ABYSSLINE; basisOfRecord: PreservedSpecimen
- **Type status:** Other material. **Occurrence:** recordNumber: NHM_0915D; recordedBy: Adrian Glover | Helena Wiklund | Thomas Dahlgren | Madeleine Brasier; individualCount: 1; preparations: Tissue voucher stored in 80% non-denatured ethanol aqueous solution | DNA voucher stored in buffer; otherCatalogNumbers: 0109405401 | 0174126580; associatedSequences: OQ746579 (16S); occurrenceID: CC066F6A-7E54-537E-8E19-5D5EE0C35B33; **Taxon:** taxonConceptID: Sabellidae sp. (NHM_915D); scientificName: Sabellidae; kingdom: Animalia; phylum: Annelida; class: Polychaeta; order: Sabellida; family: Sabellidae; taxonRank: family; scientificNameAuthorship: Latreille, 1825; **Location:** waterBody: Pacific; stateProvince: Clarion Clipperton Zone; locality: UK Seabed Resources Ltd exploration area UK-1 Stratum B; verbatimLocality: UK1 Stratum B; maximumDepthInMeters: 4198; locationRemarks: Deployment EB03; at Station U4; from R/V Thomas G. Thompson Cruise no. TN319; verbatimLatitude: 12'34.28; verbatimLongitude: 116'36.63; decimalLatitude: 12.57133; decimalLongitude: -116.6105; geodeticDatum: WGS84; **Identification:** identifiedBy: Helena Wiklund | Lenka Neal | Thomas Dahlgren | Adrian Glover | Madeleine Brasier | Regan Drennan | Eva Stewart; dateIdentified: 2021-04-20; identificationRemarks: identified by DNA and morphology; **Event:** eventID: UK1_AB02_EB03; samplingProtocol: Brenke Epibenthic Sledge; eventDate: 2015-02-23; eventTime: 05:39; habitat: Abyssal plain; fieldNotes: Collected from epi net (on the epibenthic sledge); **Record Level:** language: en; institutionCode: NHMUK; collectionCode: ZOO; datasetName: ABYSSLINE; basisOfRecord: PreservedSpecimen

##### Distribution

Eastern Clarion-Clipperton Zone, central Pacific Ocean.

##### Diagnosis

**Figure 106.**
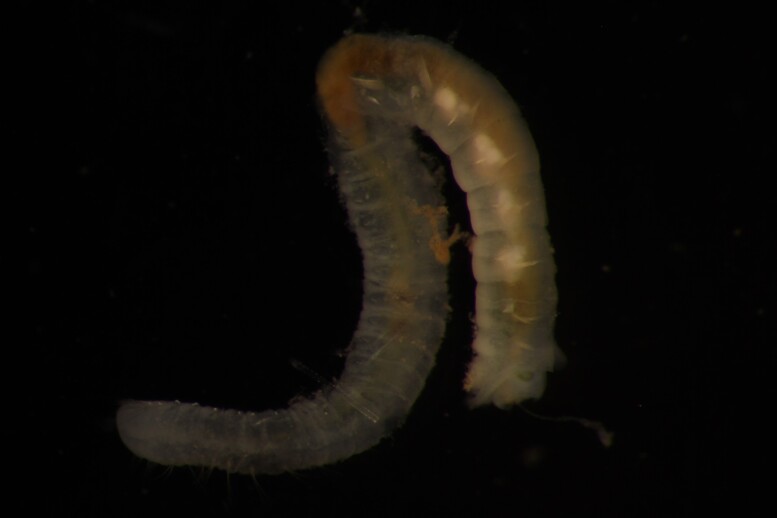
Sabellidae sp. (NHM_758), live specimen NHM_758 in lateral view.

Damaged specimens (Fig. 106) consistent with placement within family Sabellidae, based on morphology and DNA.

#### Sabellidae sp. (NHM_370)

0F5BDBAD-4E91-58D8-A4AC-54B0F383F417

##### Materials

- **Type status:** Other material. **Occurrence:** recordNumber: NHM_0370; recordedBy: Adrian Glover | Helena Wiklund | Thomas Dahlgren | Magdalena Georgieva; individualCount: 1; preparations: Tissue voucher stored in 80% non-denatured ethanol aqueous solution | DNA voucher stored in buffer; otherCatalogNumbers: 0109405375 | 0174127312; associatedSequences: OQ746507 (16S) | OQ746824 (18S); occurrenceID: CEB4D89A-37E7-5270-BA71-06B12938C9D4; **Taxon:** taxonConceptID: Sabellidae sp. (NHM_370); scientificName: Sabellidae; kingdom: Animalia; phylum: Annelida; class: Polychaeta; order: Sabellida; family: Sabellidae; taxonRank: family; scientificNameAuthorship: Latreille, 1825; **Location:** waterBody: Pacific; stateProvince: Clarion Clipperton Zone; locality: UK Seabed Resources Ltd exploration area UK-1 Stratum A; verbatimLocality: UK1 Stratum A; maximumDepthInMeters: 4182; locationRemarks: Deployment EB05; at Station H-J; from R/V Melville Cruise no. MV1313; verbatimLatitude: 13°55.984; verbatimLongitude: 116°42.977; decimalLatitude: 13.93307; decimalLongitude: -116.72378; geodeticDatum: WGS84; **Identification:** identifiedBy: Helena Wiklund | Lenka Neal | Thomas Dahlgren | Adrian Glover | Madeleine Brasier | Regan Drennan | Eva Stewart; dateIdentified: 2021-04-20; identificationRemarks: identified by DNA and morphology; **Event:** eventID: UK1_AB01_EB05; samplingProtocol: Brenke Epibenthic Sledge; eventDate: 2013-10-19; eventTime: 12:16; habitat: Abyssal plain; fieldNotes: Collected from epi net (on the epibenthic sledge); **Record Level:** language: en; institutionCode: NHMUK; collectionCode: ZOO; datasetName: ABYSSLINE; basisOfRecord: PreservedSpecimen

##### Distribution

Eastern Clarion-Clipperton Zone, central Pacific Ocean.

##### Diagnosis

**Figure 107.**
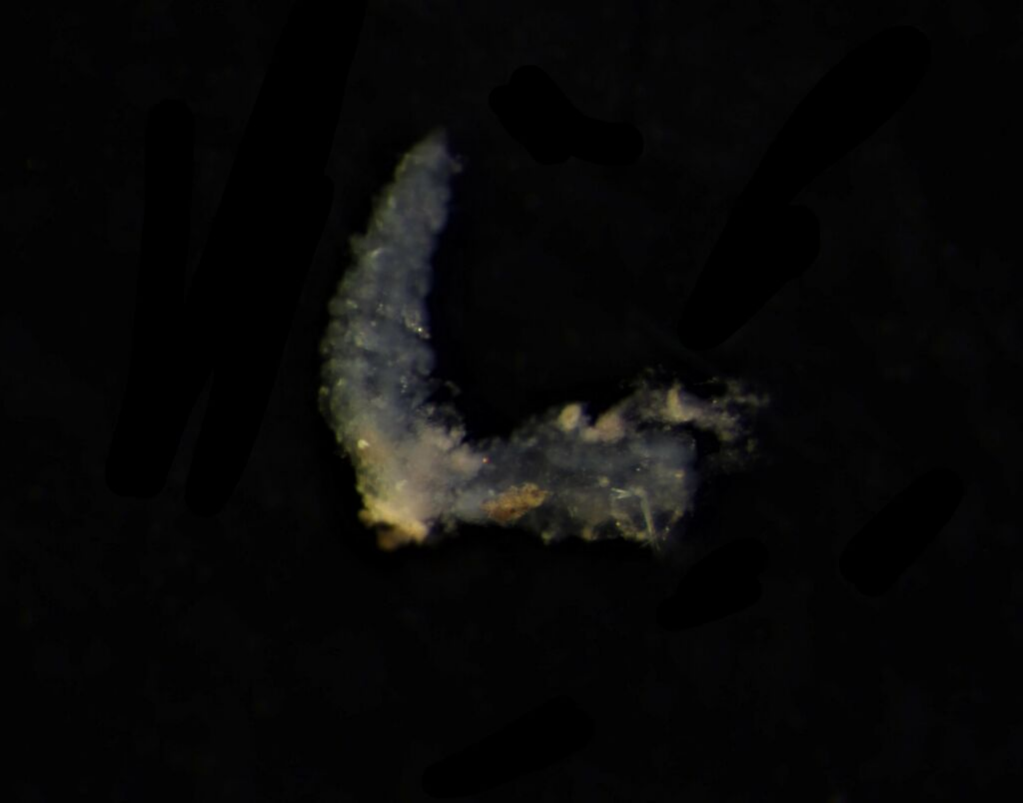
Sabellidae sp. (NHM_370), damaged live specimen NHM_370, radiolar crown missing.

Damaged specimen (Fig. 107) consistent with placement within family Sabellidae, based on morphology and DNA.

#### Sabellidae sp. (NHM_472)

ECB717F1-CF83-5C43-BE17-2DCE27A8F490

##### Materials

- **Type status:** Other material. **Occurrence:** recordNumber: NHM_0472; recordedBy: Adrian Glover | Helena Wiklund | Thomas Dahlgren | Magdalena Georgieva; individualCount: 1; preparations: Tissue voucher stored in 80% non-denatured ethanol aqueous solution | DNA voucher stored in buffer; otherCatalogNumbers: 0109405424 | 0174127304; associatedSequences: OQ746840 (18S); occurrenceID: 971F507E-8FFF-5B04-9A53-E5BB481D58EB; **Taxon:** taxonConceptID: Sabellidae sp. (NHM_472); scientificName: Sabellidae; kingdom: Animalia; phylum: Annelida; class: Polychaeta; order: Sabellida; family: Sabellidae; taxonRank: family; scientificNameAuthorship: Latreille, 1825; **Location:** waterBody: Pacific; stateProvince: Clarion Clipperton Zone; locality: UK Seabed Resources Ltd exploration area UK-1 Stratum A; verbatimLocality: UK1 Stratum A; maximumDepthInMeters: 4160; locationRemarks: Deployment BC14; at Station L; from R/V Melville Cruise no. MV1313; verbatimLatitude: 13°43.597; verbatimLongitude: 116°40.200; decimalLatitude: 13.72662; decimalLongitude: -116.67; geodeticDatum: WGS84; **Identification:** identifiedBy: Helena Wiklund | Lenka Neal | Thomas Dahlgren | Adrian Glover | Madeleine Brasier | Regan Drennan | Eva Stewart; dateIdentified: 2021-04-20; identificationRemarks: identified by DNA and morphology; **Event:** eventID: UK1_AB01_BC14; samplingProtocol: USNEL Box Core; eventDate: 2013-10-22; eventTime: 07:25; habitat: Abyssal plain; fieldNotes: Collected from 0-2 cm layer of box core using a 300 micron sieve; **Record Level:** language: en; institutionCode: NHMUK; collectionCode: ZOO; datasetName: ABYSSLINE; basisOfRecord: PreservedSpecimen

##### Distribution

Eastern Clarion-Clipperton Zone, central Pacific Ocean.

##### Diagnosis

**Figure 108.**
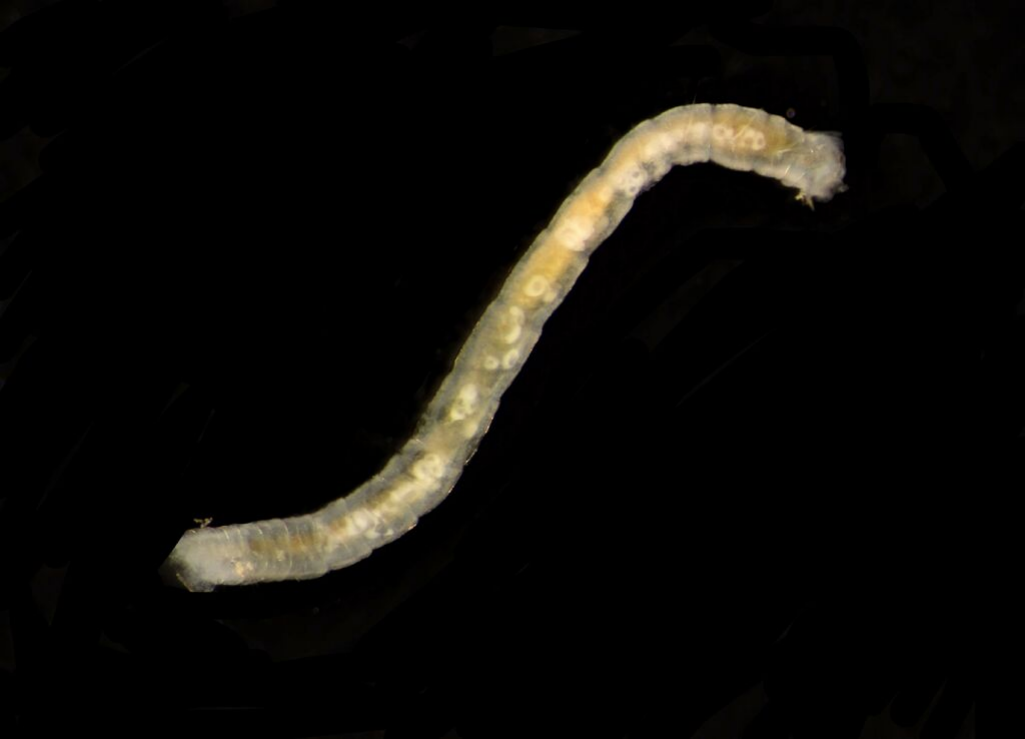
Sabellidae sp. (NHM_472), live specimen NHM_472 in lateral view, radiolar crown missing.

Damaged specimen (Fig. 108) consistent with placement within family Sabellidae, based on morphology and DNA.

#### Sabellidae sp. (NHM_1613)

9A2AB4B7-90E5-582E-913C-596F83B92EE5

##### Materials

- **Type status:** Other material. **Occurrence:** catalogNumber: NHMUK ANEA 2023.558; recordNumber: NHM_1613; recordedBy: Adrian Glover | Helena Wiklund | Thomas Dahlgren | Madeleine Brasier; individualCount: 1; preparations: specimen stored in 80% non-denatured ethanol aqueous solution | DNA voucher stored in buffer; otherCatalogNumbers: 0174126808; associatedSequences: OQ746894 (18S); occurrenceID: 316533AF-28AC-54EF-8A7B-86F7CF7DAD3E; **Taxon:** taxonConceptID: Sabellidae sp. (NHM_1613); scientificName: Sabellidae; kingdom: Animalia; phylum: Annelida; class: Polychaeta; order: Sabellida; family: Sabellidae; taxonRank: family; scientificNameAuthorship: Latreille, 1825; **Location:** waterBody: Pacific; stateProvince: Clarion Clipperton Zone; locality: UK Seabed Resources Ltd exploration area UK-1 Stratum B; verbatimLocality: UK1 Stratum B; maximumDepthInMeters: 4258; locationRemarks: Deployment BC20; at Station U13; from R/V Thomas G. Thompson Cruise no. TN319; verbatimLatitude: 12'35.813; verbatimLongitude: 116'29.614; decimalLatitude: 12.59688; decimalLongitude: -116.49357; geodeticDatum: WGS84; **Identification:** identifiedBy: Helena Wiklund | Lenka Neal | Thomas Dahlgren | Adrian Glover | Madeleine Brasier | Regan Drennan | Eva Stewart; dateIdentified: 2021-04-20; identificationRemarks: identified by DNA and morphology; **Event:** eventID: UK1_AB02_BC20; samplingProtocol: USNEL Box Core; eventDate: 2015-03-09; habitat: Abyssal plain; fieldNotes: Collected from nodule in box core sample; **Record Level:** language: en; institutionCode: NHMUK; collectionCode: ZOO; datasetName: ABYSSLINE; basisOfRecord: PreservedSpecimen

##### Distribution

Eastern Clarion-Clipperton Zone, central Pacific Ocean.

##### Diagnosis

**Figure 109.**
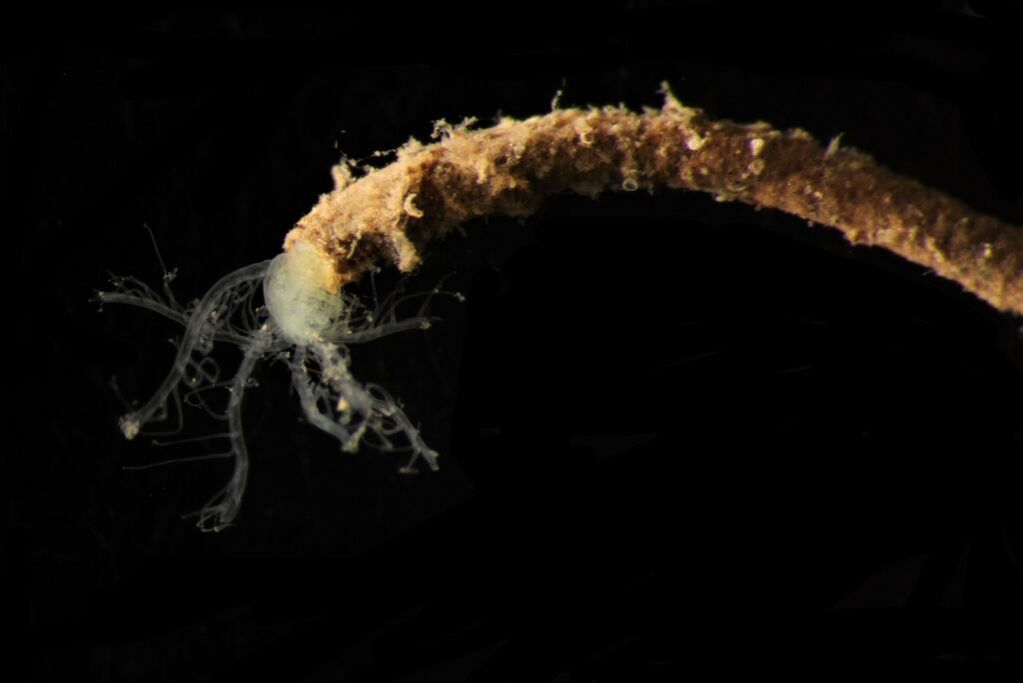
Sabellidae sp. (NHM_1613), live specimen NHM_1613 inside the tube, with radiolar crown exposed.

Specimen (Fig. 109) consistent with placement within family Sabellidae, based on morphology and DNA.

#### Sabellidae sp. (NHM_1859A)

9CB0078E-051F-5AFE-AC29-D0F4DFBFE13A

##### Materials

- **Type status:** Other material. **Occurrence:** catalogNumber: NHMUK ANEA 2023.559; recordNumber: NHM_1859A; recordedBy: Adrian Glover | Helena Wiklund | Thomas Dahlgren | Madeleine Brasier; individualCount: 1; preparations: specimen stored in 80% non-denatured ethanol aqueous solution | DNA voucher stored in buffer; otherCatalogNumbers: 0174126171; associatedSequences: OQ746725 (16S); occurrenceID: D41F517C-3909-5F06-83AE-FFB0BC6A812A; **Taxon:** taxonConceptID: Sabellidae sp. (NHM_1859A); scientificName: Sabellidae; kingdom: Animalia; phylum: Annelida; class: Polychaeta; order: Sabellida; family: Sabellidae; taxonRank: family; scientificNameAuthorship: Latreille, 1825; **Location:** waterBody: Pacific; stateProvince: Clarion Clipperton Zone; locality: Ocean Mineral Singapore exploration claim Stratum A; verbatimLocality: OMS Stratum A; maximumDepthInMeters: 4095; locationRemarks: Deployment BC23; at Station S10; from R/V Thomas G. Thompson Cruise no. TN319; verbatimLatitude: 12'03.278; verbatimLongitude: 117'15.103; decimalLatitude: 12.05463; decimalLongitude: -117.25172; geodeticDatum: WGS84; **Identification:** identifiedBy: Helena Wiklund | Lenka Neal | Thomas Dahlgren | Adrian Glover | Madeleine Brasier | Regan Drennan | Eva Stewart; dateIdentified: 2021-04-20; identificationRemarks: identified by DNA and morphology; **Event:** eventID: OMS1_AB02_BC23; samplingProtocol: USNEL Box Core; eventDate: 2015-03-13; eventTime: 09:10; habitat: Abyssal plain; fieldNotes: Collected from 0-2 cm layer of box core using a 300 micron sieve; **Record Level:** language: en; institutionCode: NHMUK; collectionCode: ZOO; datasetName: ABYSSLINE; basisOfRecord: PreservedSpecimen

##### Distribution

Eastern Clarion-Clipperton Zone, central Pacific Ocean.

##### Diagnosis

**Figure 110.**
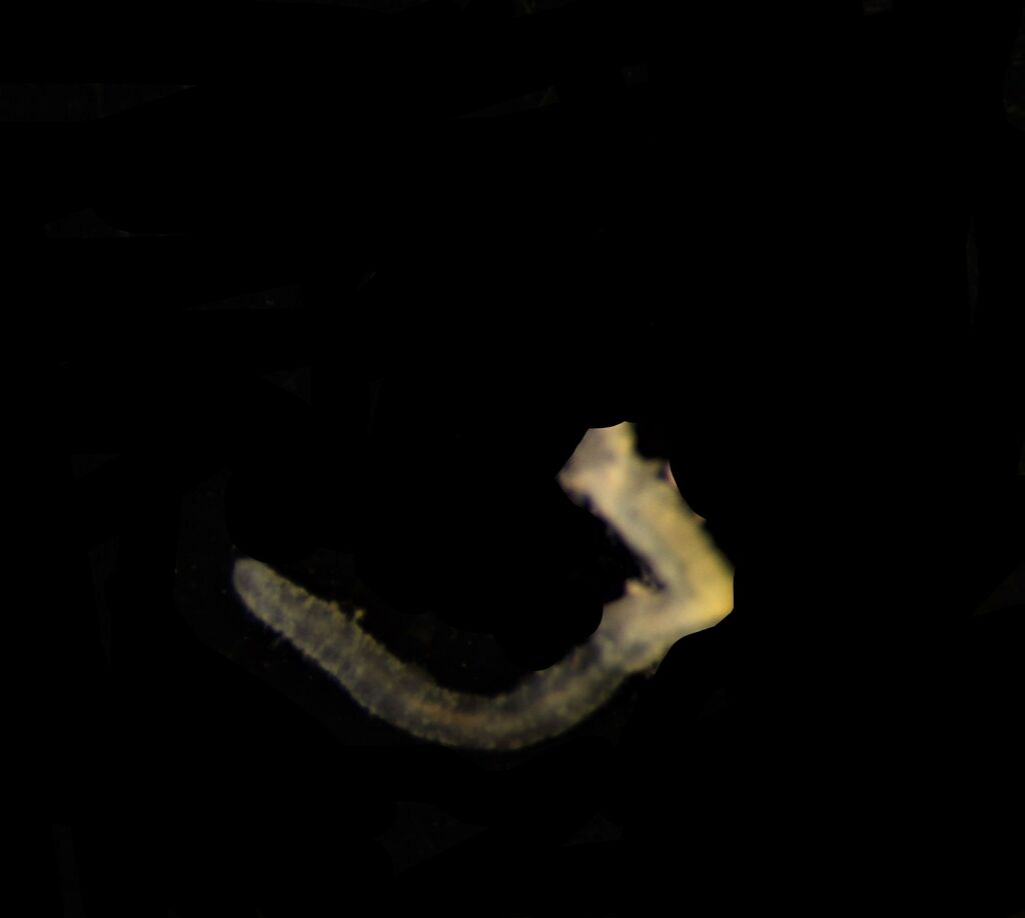
Sabellidae sp. (NHM_1859A) damaged, live specimen NHM_1859A, radiolar crown missing.

Damaged specimen (Fig. 110) consistent with placement within family Sabellidae, based on morphology and DNA.

#### Sabellidae sp. (NHM_1879)

DE33192B-448F-5BA6-86F4-6637796EA0E3

##### Materials

- **Type status:** Other material. **Occurrence:** catalogNumber: NHMUK ANEA 2023.560; recordNumber: NHM_1879; recordedBy: Adrian Glover | Helena Wiklund | Thomas Dahlgren | Madeleine Brasier; individualCount: 1; preparations: specimen stored in 80% non-denatured ethanol aqueous solution | DNA voucher stored in buffer; otherCatalogNumbers: 0174126211; associatedSequences: OQ746728 (16S); occurrenceID: 03338B89-4B00-5590-B6CD-03B8EA403062; **Taxon:** taxonConceptID: Sabellidae sp. (NHM_1879); scientificName: Sabellidae; kingdom: Animalia; phylum: Annelida; class: Polychaeta; order: Sabellida; family: Sabellidae; taxonRank: family; scientificNameAuthorship: Latreille, 1825; **Location:** waterBody: Pacific; stateProvince: Clarion Clipperton Zone; locality: Ocean Mineral Singapore exploration claim Stratum A; verbatimLocality: OMS Stratum A; maximumDepthInMeters: 4094; locationRemarks: Deployment EB11; at Station S10; from R/V Thomas G. Thompson Cruise no. TN319; verbatimLatitude: 12°02.49’; verbatimLongitude: 117°13.03’; decimalLatitude: 12.0415; decimalLongitude: -117.21717; geodeticDatum: WGS84; **Identification:** identifiedBy: Helena Wiklund | Lenka Neal | Thomas Dahlgren | Adrian Glover | Madeleine Brasier | Regan Drennan | Eva Stewart; dateIdentified: 2021-04-20; identificationRemarks: identified by DNA and morphology; **Event:** eventID: OMS1_AB02_EB11; samplingProtocol: Brenke Epibenthic Sledge; eventDate: 2015-03-13; habitat: Abyssal plain; fieldNotes: Collected from epi net (on the epibenthic sledge); **Record Level:** language: en; institutionCode: NHMUK; collectionCode: ZOO; datasetName: ABYSSLINE; basisOfRecord: PreservedSpecimen

##### Distribution

Eastern Clarion-Clipperton Zone, central Pacific Ocean.

##### Diagnosis

**Figure 111.**
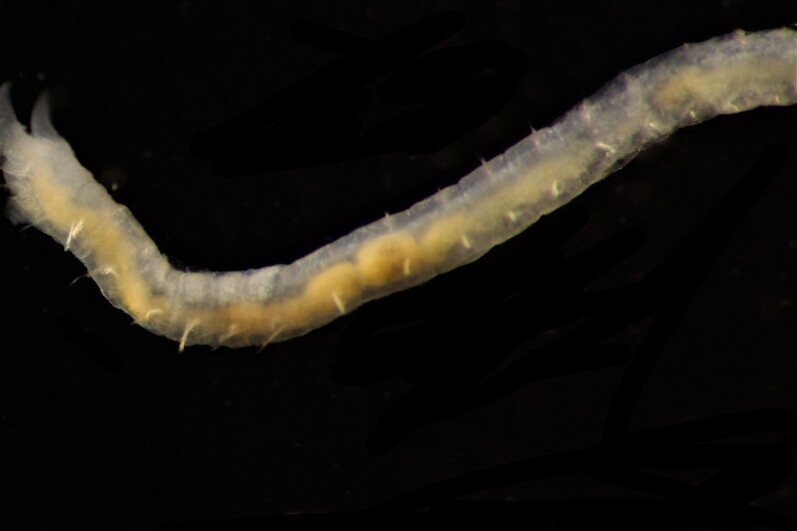
Sabellidae sp. (NHM_1879), live specimen NHM_1879 in lateral view, radiolar crown missing.

Damaged specimen (Fig. 111) consistent with placement within family Sabellidae, based on morphology and DNA.

#### Sabellidae sp. (NHM_1991)

3518B52D-482D-55D3-89A1-CDF26E6BC97E

##### Materials

- **Type status:** Other material. **Occurrence:** catalogNumber: NHMUK ANEA 2023.561; recordNumber: NHM_1991; recordedBy: Adrian Glover | Helena Wiklund | Thomas Dahlgren | Madeleine Brasier; individualCount: 1; preparations: specimen stored in 80% non-denatured ethanol aqueous solution | DNA voucher stored in buffer; otherCatalogNumbers: 0174123621; associatedSequences: OQ746743 (16S); occurrenceID: 8091763D-26CA-5528-9FFD-734ABBCDB377; **Taxon:** taxonConceptID: Sabellidae sp. (NHM_1991); scientificName: Sabellidae; kingdom: Animalia; phylum: Annelida; class: Polychaeta; order: Sabellida; family: Sabellidae; taxonRank: family; scientificNameAuthorship: Latreille, 1825; **Location:** waterBody: Pacific; stateProvince: Clarion Clipperton Zone; locality: Ocean Mineral Singapore exploration claim Stratum A; verbatimLocality: OMS Stratum A; maximumDepthInMeters: 4141; locationRemarks: Deployment BC25; at Station S11; from R/V Thomas G. Thompson Cruise no. TN319; verbatimLatitude: 12'00.559; verbatimLongitude: 117'22.818; decimalLatitude: 12.00932; decimalLongitude: -117.3803; geodeticDatum: WGS84; **Identification:** identifiedBy: Helena Wiklund | Lenka Neal | Thomas Dahlgren | Adrian Glover | Madeleine Brasier | Regan Drennan | Eva Stewart; dateIdentified: 2021-04-20; identificationRemarks: identified by DNA and morphology; **Event:** eventID: OMS1_AB02_BC25; samplingProtocol: USNEL Box Core; eventDate: 2015-03-15; eventTime: 13:10; habitat: Abyssal plain; fieldNotes: Collected from 0-2 cm layer of box core using a 300 micron sieve; **Record Level:** language: en; institutionCode: NHMUK; collectionCode: ZOO; datasetName: ABYSSLINE; basisOfRecord: PreservedSpecimen

##### Distribution

Eastern Clarion-Clipperton Zone, central Pacific Ocean.

##### Diagnosis

**Figure 112.**
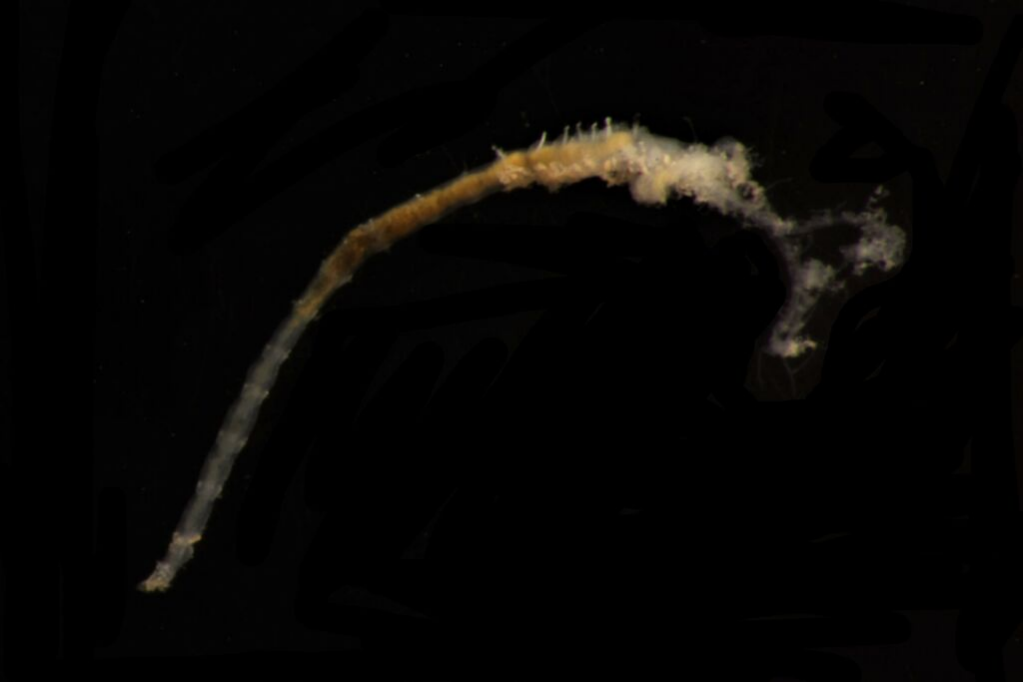
Sabellidae sp. (NHM_1991), live specimen NHM_1991 with damaged radiolar crown attached.

Damaged specimen (Fig. 112) consistent with placement within family Sabellidae, based on morphology and DNA.

### Sigalionidae Kinberg, 1856

#### Pholoinae sp. (NHM_366)

D44B9258-764E-5B7C-96FF-70C93D5D7000

##### Materials

- **Type status:** Other material. **Occurrence:** catalogNumber: NHMUK ANEA 2023.568; recordNumber: NHM_2094; recordedBy: Adrian Glover | Helena Wiklund | Thomas Dahlgren | Madeleine Brasier; individualCount: 1; preparations: specimen stored in 80% non-denatured ethanol aqueous solution | DNA voucher stored in buffer; otherCatalogNumbers: 0174126749; associatedSequences: OQ746750 (16S) | OQ738605 (COI); occurrenceID: FD491414-878C-5445-9F23-D5AC39B1814E; **Taxon:** taxonConceptID: Pholoinae sp. (NHM_366); scientificName: Pholoinae; kingdom: Animalia; phylum: Annelida; class: Polychaeta; order: Phyllodocida; family: Sigalionidae; taxonRank: family; scientificNameAuthorship: Kinberg, 1858; **Location:** waterBody: Pacific; stateProvince: Clarion Clipperton Zone; locality: Area of Particular Interest APEI-6; verbatimLocality: APEI-6; maximumDepthInMeters: 4026; locationRemarks: Deployment EB13; at Station APEI; from R/V Thomas G. Thompson Cruise no. TN319; verbatimLatitude: 19 27.874; verbatimLongitude: 120 01.525; decimalLatitude: 19.46457; decimalLongitude: -120.02542; geodeticDatum: WGS84; **Identification:** identifiedBy: Helena Wiklund | Lenka Neal | Thomas Dahlgren | Adrian Glover | Madeleine Brasier | Regan Drennan | Eva Stewart; dateIdentified: 2021-04-20; identificationRemarks: identified by DNA and morphology; **Event:** eventID: APEI6_AB02_EB13; samplingProtocol: Brenke Epibenthic Sledge; eventDate: 2015-03-20; eventTime: 16:12; habitat: Abyssal plain; fieldNotes: Collected from epi net (on the epibenthic sledge); **Record Level:** language: en; institutionCode: NHMUK; collectionCode: ZOO; datasetName: ABYSSLINE; basisOfRecord: PreservedSpecimen
- **Type status:** Other material. **Occurrence:** catalogNumber: NHMUK ANEA 2023.565; recordNumber: NHM_0366; recordedBy: Adrian Glover | Helena Wiklund | Thomas Dahlgren | Magdalena Georgieva; individualCount: 1; preparations: specimen stored in 80% non-denatured ethanol aqueous solution | DNA voucher stored in buffer; otherCatalogNumbers: 0174127327; associatedSequences: OQ746506 (16S) | OQ746823 (18S) | OQ738516 (COI); occurrenceID: 918FE2B5-F123-563E-BA10-099CCD0291A7; **Taxon:** taxonConceptID: Pholoinae sp. (NHM_366); scientificName: Pholoinae; kingdom: Animalia; phylum: Annelida; class: Polychaeta; order: Phyllodocida; family: Sigalionidae; taxonRank: family; scientificNameAuthorship: Kinberg, 1858; **Location:** waterBody: Pacific; stateProvince: Clarion Clipperton Zone; locality: UK Seabed Resources Ltd exploration area UK-1 Stratum A; verbatimLocality: UK1 Stratum A; maximumDepthInMeters: 4182; locationRemarks: Deployment EB05; at Station H-J; from R/V Melville Cruise no. MV1313; verbatimLatitude: 13°55.984; verbatimLongitude: 116°42.977; decimalLatitude: 13.93307; decimalLongitude: -116.72378; geodeticDatum: WGS84; **Identification:** identifiedBy: Helena Wiklund | Lenka Neal | Thomas Dahlgren | Adrian Glover | Madeleine Brasier | Regan Drennan | Eva Stewart; dateIdentified: 2021-04-20; identificationRemarks: identified by DNA and morphology; **Event:** eventID: UK1_AB01_EB05; samplingProtocol: Brenke Epibenthic Sledge; eventDate: 2013-10-19; eventTime: 12:16; habitat: Abyssal plain; fieldNotes: Collected from epi net (on the epibenthic sledge); **Record Level:** language: en; institutionCode: NHMUK; collectionCode: ZOO; datasetName: ABYSSLINE; basisOfRecord: PreservedSpecimen
- **Type status:** Other material. **Occurrence:** catalogNumber: NHMUK ANEA 2023.567; recordNumber: NHM_1680; recordedBy: Adrian Glover | Helena Wiklund | Thomas Dahlgren | Madeleine Brasier; individualCount: 1; preparations: specimen stored in 80% non-denatured ethanol aqueous solution | DNA voucher stored in buffer; otherCatalogNumbers: 0174126236; associatedSequences: OQ746706 (16S); occurrenceID: AA7D6FA1-56B3-572D-8AB7-D73EEFCD3316; **Taxon:** taxonConceptID: Pholoinae sp. (NHM_366); scientificName: Pholoinae; kingdom: Animalia; phylum: Annelida; class: Polychaeta; order: Phyllodocida; family: Sigalionidae; taxonRank: family; scientificNameAuthorship: Kinberg, 1858; **Location:** waterBody: Pacific; stateProvince: Clarion Clipperton Zone; locality: UK Seabed Resources Ltd exploration area UK-1 Stratum B; verbatimLocality: UK1 Stratum B; maximumDepthInMeters: 4233; locationRemarks: Deployment EB09; at Station U1; from R/V Thomas G. Thompson Cruise no. TN319; verbatimLatitude: 12'21.81; verbatimLongitude: 116'40.86; decimalLatitude: 12.3635; decimalLongitude: -116.681; geodeticDatum: WGS84; **Identification:** identifiedBy: Helena Wiklund | Lenka Neal | Thomas Dahlgren | Adrian Glover | Madeleine Brasier | Regan Drennan | Eva Stewart; dateIdentified: 2021-04-20; identificationRemarks: identified by DNA and morphology; **Event:** eventID: UK1_AB02_EB09; samplingProtocol: Brenke Epibenthic Sledge; eventDate: 2015-03-10; eventTime: 10:46; habitat: Abyssal plain; fieldNotes: Collected from epi net (on the epibenthic sledge); **Record Level:** language: en; institutionCode: NHMUK; collectionCode: ZOO; datasetName: ABYSSLINE; basisOfRecord: PreservedSpecimen
- **Type status:** Other material. **Occurrence:** catalogNumber: NHMUK ANEA 2023.566; recordNumber: NHM_0590; recordedBy: Adrian Glover | Helena Wiklund | Thomas Dahlgren | Madeleine Brasier; individualCount: 1; preparations: specimen stored in 80% non-denatured ethanol aqueous solution | DNA voucher stored in buffer; otherCatalogNumbers: 0174126552; associatedSequences: OQ746533 (16S); occurrenceID: ABE6EC0B-BA88-5D6F-B52B-AC23A2ACA87D; **Taxon:** taxonConceptID: Pholoinae sp. (NHM_366); scientificName: Pholoinae; kingdom: Animalia; phylum: Annelida; class: Polychaeta; order: Phyllodocida; family: Sigalionidae; taxonRank: family; scientificNameAuthorship: Kinberg, 1858; **Location:** waterBody: Pacific; stateProvince: Clarion Clipperton Zone; locality: UK Seabed Resources Ltd exploration area UK-1 Stratum B; verbatimLocality: UK1 Stratum B; maximumDepthInMeters: 4202; locationRemarks: Deployment EB01; at Station U2; from R/V Thomas G. Thompson Cruise no. TN319; verbatimLatitude: 12'23.17456; verbatimLongitude: 116'32.92021; decimalLatitude: 12.38624; decimalLongitude: -116.54867; geodeticDatum: WGS84; **Identification:** identifiedBy: Helena Wiklund | Lenka Neal | Thomas Dahlgren | Adrian Glover | Madeleine Brasier | Regan Drennan | Eva Stewart; dateIdentified: 2021-04-20; identificationRemarks: identified by DNA and morphology; **Event:** eventID: UK1_AB02_EB01; samplingProtocol: Brenke Epibenthic Sledge; eventDate: 2015-02-17; eventTime: 05:15; habitat: Abyssal plain; fieldNotes: Collected from epi net (on the epibenthic sledge); **Record Level:** language: en; institutionCode: NHMUK; collectionCode: ZOO; datasetName: ABYSSLINE; basisOfRecord: PreservedSpecimen

##### Distribution

Eastern Clarion-Clipperton Zone, central Pacific Ocean.

##### Diagnosis

**Figure 113.**
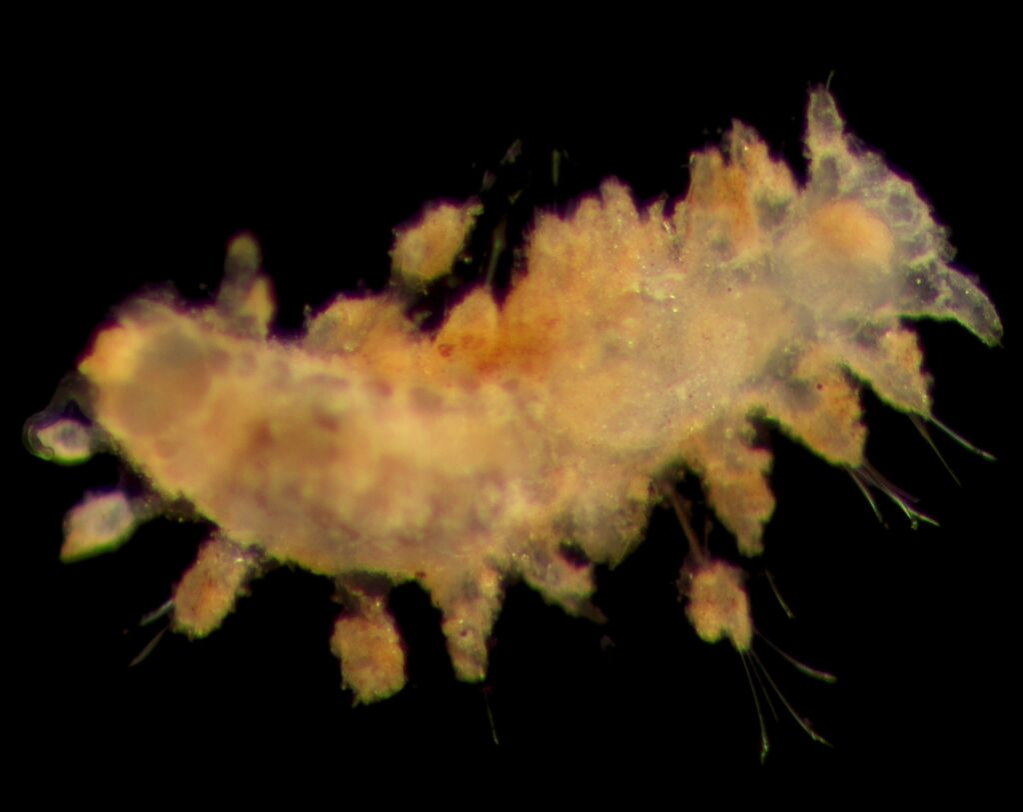
Pholoinae sp. (NHM_366), anterior fragment of live specimen NHM_2094 in dorsal view.

Damaged specimens (Fig. 113) consistent with placement within subfamily Pholoinae, based on morphology and DNA.

#### Sigalionidae sp. (NHM_342)

E63D2AD4-3108-5DE5-8445-1E4D0163BBC4

##### Materials

- **Type status:** Other material. **Occurrence:** catalogNumber: NHMUK ANEA 2023.575; recordNumber: NHM_1316; recordedBy: Adrian Glover | Helena Wiklund | Thomas Dahlgren | Madeleine Brasier; individualCount: 1; preparations: specimen stored in 80% non-denatured ethanol aqueous solution | DNA voucher stored in buffer; otherCatalogNumbers: 0174126586; associatedSequences: OQ746658 (16S) | OQ738577 (COI); occurrenceID: D86069F3-7E9C-5522-9F1B-EFCB3A000270; **Taxon:** taxonConceptID: Sigalionidae sp. (NHM_342); scientificName: Sigalionidae; kingdom: Animalia; phylum: Annelida; class: Polychaeta; order: Phyllodocida; family: Sigalionidae; taxonRank: family; scientificNameAuthorship: Kinberg, 1856; **Location:** waterBody: Pacific; stateProvince: Clarion Clipperton Zone; locality: Ocean Mineral Singapore exploration claim Stratum A; verbatimLocality: OMS Stratum A; maximumDepthInMeters: 4302; locationRemarks: Deployment EB06; at Station S5; from R/V Thomas G. Thompson Cruise no. TN319; verbatimLatitude: 12'15.44; verbatimLongitude: 117'18.13; decimalLatitude: 12.25733; decimalLongitude: -117.30217; geodeticDatum: WGS84; **Identification:** identifiedBy: Helena Wiklund | Lenka Neal | Thomas Dahlgren | Adrian Glover | Madeleine Brasier | Regan Drennan | Eva Stewart; dateIdentified: 2021-04-20; identificationRemarks: identified by DNA and morphology; **Event:** eventID: OMS1_AB02_EB06; samplingProtocol: Brenke Epibenthic Sledge; eventDate: 2015-03-01; eventTime: 04:02; habitat: Abyssal plain; fieldNotes: Collected from epi net (on the epibenthic sledge); **Record Level:** language: en; institutionCode: NHMUK; collectionCode: ZOO; datasetName: ABYSSLINE; basisOfRecord: PreservedSpecimen
- **Type status:** Other material. **Occurrence:** catalogNumber: NHMUK ANEA 2023.578; recordNumber: NHM_1947H; recordedBy: Adrian Glover | Helena Wiklund | Thomas Dahlgren | Madeleine Brasier; individualCount: 1; preparations: specimen stored in 80% non-denatured ethanol aqueous solution | DNA voucher stored in buffer; otherCatalogNumbers: 0174126186; associatedSequences: OQ746737 (16S); occurrenceID: B266112D-6C5E-52B1-BC98-11607941F1B8; **Taxon:** taxonConceptID: Sigalionidae sp. (NHM_342); scientificName: Sigalionidae; kingdom: Animalia; phylum: Annelida; class: Polychaeta; order: Phyllodocida; family: Sigalionidae; taxonRank: family; scientificNameAuthorship: Kinberg, 1856; **Location:** waterBody: Pacific; stateProvince: Clarion Clipperton Zone; locality: Ocean Mineral Singapore exploration claim Stratum A; verbatimLocality: OMS Stratum A; maximumDepthInMeters: 4094; locationRemarks: Deployment EB11; at Station S10; from R/V Thomas G. Thompson Cruise no. TN319; verbatimLatitude: 12°02.49’; verbatimLongitude: 117°13.03’; decimalLatitude: 12.0415; decimalLongitude: -117.21717; geodeticDatum: WGS84; **Identification:** identifiedBy: Helena Wiklund | Lenka Neal | Thomas Dahlgren | Adrian Glover | Madeleine Brasier | Regan Drennan | Eva Stewart; dateIdentified: 2021-04-20; identificationRemarks: identified by DNA and morphology; **Event:** eventID: OMS1_AB02_EB11; samplingProtocol: Brenke Epibenthic Sledge; eventDate: 2015-03-13; habitat: Abyssal plain; fieldNotes: Collected from epi net (on the epibenthic sledge); **Record Level:** language: en; institutionCode: NHMUK; collectionCode: ZOO; datasetName: ABYSSLINE; basisOfRecord: PreservedSpecimen
- **Type status:** Other material. **Occurrence:** catalogNumber: NHMUK ANEA 2023.574; recordNumber: NHM_1164D; recordedBy: Adrian Glover | Helena Wiklund | Thomas Dahlgren | Madeleine Brasier; individualCount: 1; preparations: specimen stored in 80% non-denatured ethanol aqueous solution | DNA voucher stored in buffer; otherCatalogNumbers: 0174126584; associatedSequences: OQ746633 (16S) | OQ738565 (COI); occurrenceID: 1BCDD598-8AA2-5451-B4D8-40E80FAD294D; **Taxon:** taxonConceptID: Sigalionidae sp. (NHM_342); scientificName: Sigalionidae; kingdom: Animalia; phylum: Annelida; class: Polychaeta; order: Phyllodocida; family: Sigalionidae; taxonRank: family; scientificNameAuthorship: Kinberg, 1856; **Location:** waterBody: Pacific; stateProvince: Clarion Clipperton Zone; locality: Ocean Mineral Singapore exploration claim Stratum A; verbatimLocality: OMS Stratum A; maximumDepthInMeters: 4100; locationRemarks: Deployment EB05; at Station S2; from R/V Thomas G. Thompson Cruise no. TN319; verbatimLatitude: 12'06.93; verbatimLongitude: 117'09.87; decimalLatitude: 12.1155; decimalLongitude: -117.1645; geodeticDatum: WGS84; **Identification:** identifiedBy: Helena Wiklund | Lenka Neal | Thomas Dahlgren | Adrian Glover | Madeleine Brasier | Regan Drennan | Eva Stewart; dateIdentified: 2021-04-20; identificationRemarks: identified by DNA and morphology; **Event:** eventID: OMS1_AB02_EB05; samplingProtocol: Brenke Epibenthic Sledge; eventDate: 2015-02-26; eventTime: 21:29; habitat: Abyssal plain; fieldNotes: Collected from epi net (on the epibenthic sledge); **Record Level:** language: en; institutionCode: NHMUK; collectionCode: ZOO; datasetName: ABYSSLINE; basisOfRecord: PreservedSpecimen
- **Type status:** Other material. **Occurrence:** catalogNumber: NHMUK ANEA 2023.576; recordNumber: NHM_1478; recordedBy: Adrian Glover | Helena Wiklund | Thomas Dahlgren | Madeleine Brasier; individualCount: 1; preparations: specimen stored in 80% non-denatured ethanol aqueous solution | DNA voucher stored in buffer; otherCatalogNumbers: 0174126756; associatedSequences: OQ746683 (16S) | OQ738586 (COI); occurrenceID: B73A340D-71B4-5201-BB20-746A3519B408; **Taxon:** taxonConceptID: Sigalionidae sp. (NHM_342); scientificName: Sigalionidae; kingdom: Animalia; phylum: Annelida; class: Polychaeta; order: Phyllodocida; family: Sigalionidae; taxonRank: family; scientificNameAuthorship: Kinberg, 1856; **Location:** waterBody: Pacific; stateProvince: Clarion Clipperton Zone; locality: UK Seabed Resources Ltd exploration area UK-1 Stratum B; verbatimLocality: UK1 Stratum B; maximumDepthInMeters: 4137; locationRemarks: Deployment EB07; at Station U7; from R/V Thomas G. Thompson Cruise no. TN319; verbatimLatitude: 12'27.26; verbatimLongitude: 116'36.77; decimalLatitude: 12.45433; decimalLongitude: -116.61283; geodeticDatum: WGS84; **Identification:** identifiedBy: Helena Wiklund | Lenka Neal | Thomas Dahlgren | Adrian Glover | Madeleine Brasier | Regan Drennan | Eva Stewart; dateIdentified: 2021-04-20; identificationRemarks: identified by DNA and morphology; **Event:** eventID: UK1_AB02_EB07; samplingProtocol: Brenke Epibenthic Sledge; eventDate: 2015-03-03; eventTime: 20:40; habitat: Abyssal plain; fieldNotes: Collected from epi net (on the epibenthic sledge); **Record Level:** language: en; institutionCode: NHMUK; collectionCode: ZOO; datasetName: ABYSSLINE; basisOfRecord: PreservedSpecimen
- **Type status:** Other material. **Occurrence:** catalogNumber: NHMUK ANEA 2023.577; recordNumber: NHM_1479; recordedBy: Adrian Glover | Helena Wiklund | Thomas Dahlgren | Madeleine Brasier; individualCount: 1; preparations: specimen stored in 80% non-denatured ethanol aqueous solution | DNA voucher stored in buffer; otherCatalogNumbers: 0174126191; associatedSequences: OQ746684 (16S) | OQ738587 (COI); occurrenceID: F8FFC88C-7351-54D4-9429-A7F49757B676; **Taxon:** taxonConceptID: Sigalionidae sp. (NHM_342); scientificName: Sigalionidae; kingdom: Animalia; phylum: Annelida; class: Polychaeta; order: Phyllodocida; family: Sigalionidae; taxonRank: family; scientificNameAuthorship: Kinberg, 1856; **Location:** waterBody: Pacific; stateProvince: Clarion Clipperton Zone; locality: UK Seabed Resources Ltd exploration area UK-1 Stratum B; verbatimLocality: UK1 Stratum B; maximumDepthInMeters: 4137; locationRemarks: Deployment EB07; at Station U7; from R/V Thomas G. Thompson Cruise no. TN319; verbatimLatitude: 12'27.26; verbatimLongitude: 116'36.77; decimalLatitude: 12.45433; decimalLongitude: -116.61283; geodeticDatum: WGS84; **Identification:** identifiedBy: Helena Wiklund | Lenka Neal | Thomas Dahlgren | Adrian Glover | Madeleine Brasier | Regan Drennan | Eva Stewart; dateIdentified: 2021-04-20; identificationRemarks: identified by DNA and morphology; **Event:** eventID: UK1_AB02_EB07; samplingProtocol: Brenke Epibenthic Sledge; eventDate: 2015-03-03; eventTime: 20:40; habitat: Abyssal plain; fieldNotes: Collected from epi net (on the epibenthic sledge); **Record Level:** language: en; institutionCode: NHMUK; collectionCode: ZOO; datasetName: ABYSSLINE; basisOfRecord: PreservedSpecimen
- **Type status:** Other material. **Occurrence:** catalogNumber: NHMUK ANEA 2023.573; recordNumber: NHM_0917F; recordedBy: Adrian Glover | Helena Wiklund | Thomas Dahlgren | Madeleine Brasier; individualCount: 1; preparations: specimen stored in 80% non-denatured ethanol aqueous solution | DNA voucher stored in buffer; otherCatalogNumbers: 0174126570; associatedSequences: OQ746588 (16S) | OQ738546 (COI); occurrenceID: 1240810A-A434-5608-B59B-06E7064DB3D6; **Taxon:** taxonConceptID: Sigalionidae sp. (NHM_342); scientificName: Sigalionidae; kingdom: Animalia; phylum: Annelida; class: Polychaeta; order: Phyllodocida; family: Sigalionidae; taxonRank: family; scientificNameAuthorship: Kinberg, 1856; **Location:** waterBody: Pacific; stateProvince: Clarion Clipperton Zone; locality: UK Seabed Resources Ltd exploration area UK-1 Stratum B; verbatimLocality: UK1 Stratum B; maximumDepthInMeters: 4198; locationRemarks: Deployment EB03; at Station U4; from R/V Thomas G. Thompson Cruise no. TN319; verbatimLatitude: 12'34.28; verbatimLongitude: 116'36.63; decimalLatitude: 12.57133; decimalLongitude: -116.6105; geodeticDatum: WGS84; **Identification:** identifiedBy: Helena Wiklund | Lenka Neal | Thomas Dahlgren | Adrian Glover | Madeleine Brasier | Regan Drennan | Eva Stewart; dateIdentified: 2021-04-20; identificationRemarks: identified by DNA and morphology; **Event:** eventID: UK1_AB02_EB03; samplingProtocol: Brenke Epibenthic Sledge; eventDate: 2015-02-23; eventTime: 05:39; habitat: Abyssal plain; fieldNotes: Collected from epi net (on the epibenthic sledge); **Record Level:** language: en; institutionCode: NHMUK; collectionCode: ZOO; datasetName: ABYSSLINE; basisOfRecord: PreservedSpecimen
- **Type status:** Other material. **Occurrence:** catalogNumber: NHMUK ANEA 2023.572; recordNumber: NHM_0695; recordedBy: Adrian Glover | Helena Wiklund | Thomas Dahlgren | Madeleine Brasier; individualCount: 1; preparations: specimen stored in 80% non-denatured ethanol aqueous solution | DNA voucher stored in buffer; otherCatalogNumbers: 0174127382; associatedSequences: OQ746541 (16S) | OQ738532 (COI); occurrenceID: F9A1A721-461C-5D26-B858-D54D89D9BDAC; **Taxon:** taxonConceptID: Sigalionidae sp. (NHM_342); scientificName: Sigalionidae; kingdom: Animalia; phylum: Annelida; class: Polychaeta; order: Phyllodocida; family: Sigalionidae; taxonRank: family; scientificNameAuthorship: Kinberg, 1856; **Location:** waterBody: Pacific; stateProvince: Clarion Clipperton Zone; locality: UK Seabed Resources Ltd exploration area UK-1 Stratum B; verbatimLocality: UK1 Stratum B; maximumDepthInMeters: 4425; locationRemarks: Deployment EB02; at Station U5; from R/V Thomas G. Thompson Cruise no. TN319; verbatimLatitude: 12'32.23; verbatimLongitude: 116'36.25; decimalLatitude: 12.53717; decimalLongitude: -116.60417; geodeticDatum: WGS84; **Identification:** identifiedBy: Helena Wiklund | Lenka Neal | Thomas Dahlgren | Adrian Glover | Madeleine Brasier | Regan Drennan | Eva Stewart; dateIdentified: 2021-04-20; identificationRemarks: identified by DNA and morphology; **Event:** eventID: UK1_AB02_EB02; samplingProtocol: Brenke Epibenthic Sledge; eventDate: 2015-02-20; eventTime: 06:24; habitat: Abyssal plain; fieldNotes: Collected from epi net (on the epibenthic sledge); **Record Level:** language: en; institutionCode: NHMUK; collectionCode: ZOO; datasetName: ABYSSLINE; basisOfRecord: PreservedSpecimen
- **Type status:** Other material. **Occurrence:** catalogNumber: NHMUK ANEA 2023.571; recordNumber: NHM_0605; recordedBy: Adrian Glover | Helena Wiklund | Thomas Dahlgren | Madeleine Brasier; individualCount: 1; preparations: specimen stored in 80% non-denatured ethanol aqueous solution | DNA voucher stored in buffer; otherCatalogNumbers: 0174126622; associatedSequences: OQ746535 (16S) | OQ738529 (COI); occurrenceID: 0D130D40-E5B1-5382-B308-31E97B88FB49; **Taxon:** taxonConceptID: Sigalionidae sp. (NHM_342); scientificName: Sigalionidae; kingdom: Animalia; phylum: Annelida; class: Polychaeta; order: Phyllodocida; family: Sigalionidae; taxonRank: family; scientificNameAuthorship: Kinberg, 1856; **Location:** waterBody: Pacific; stateProvince: Clarion Clipperton Zone; locality: UK Seabed Resources Ltd exploration area UK-1 Stratum B; verbatimLocality: UK1 Stratum B; maximumDepthInMeters: 4202; locationRemarks: Deployment EB01; at Station U2; from R/V Thomas G. Thompson Cruise no. TN319; verbatimLatitude: 12'23.17456; verbatimLongitude: 116'32.92021; decimalLatitude: 12.38624; decimalLongitude: -116.54867; geodeticDatum: WGS84; **Identification:** identifiedBy: Helena Wiklund | Lenka Neal | Thomas Dahlgren | Adrian Glover | Madeleine Brasier | Regan Drennan | Eva Stewart; dateIdentified: 2021-04-20; identificationRemarks: identified by DNA and morphology; **Event:** eventID: UK1_AB02_EB01; samplingProtocol: Brenke Epibenthic Sledge; eventDate: 2015-02-17; eventTime: 05:15; habitat: Abyssal plain; fieldNotes: Collected from epi net (on the epibenthic sledge); **Record Level:** language: en; institutionCode: NHMUK; collectionCode: ZOO; datasetName: ABYSSLINE; basisOfRecord: PreservedSpecimen
- **Type status:** Other material. **Occurrence:** catalogNumber: NHMUK ANEA 2023.570; recordNumber: NHM_0342; recordedBy: Adrian Glover | Helena Wiklund | Thomas Dahlgren | Magdalena Georgieva; individualCount: 1; preparations: specimen stored in 80% non-denatured ethanol aqueous solution | DNA voucher stored in buffer; otherCatalogNumbers: 0174127316; associatedSequences: OQ746500 (16S) | OQ746818 (18S) | OQ738514 (COI); occurrenceID: 22BA6487-4EC2-5947-91DB-5B291C670FC3; **Taxon:** taxonConceptID: Sigalionidae sp. (NHM_342); scientificName: Sigalionidae; kingdom: Animalia; phylum: Annelida; class: Polychaeta; order: Phyllodocida; family: Sigalionidae; taxonRank: family; scientificNameAuthorship: Kinberg, 1856; **Location:** waterBody: Pacific; stateProvince: Clarion Clipperton Zone; locality: UK Seabed Resources Ltd exploration area UK-1 Stratum A; verbatimLocality: UK1 Stratum A; maximumDepthInMeters: 4128; locationRemarks: Deployment EB04; at Station G-I; from R/V Melville Cruise no. MV1313; verbatimLatitude: 13°45.21N; verbatimLongitude: 116°29.12W; decimalLatitude: 13.75583; decimalLongitude: -116.48667; geodeticDatum: WGS84; **Identification:** identifiedBy: Helena Wiklund | Lenka Neal | Thomas Dahlgren | Adrian Glover | Madeleine Brasier | Regan Drennan | Eva Stewart; dateIdentified: 2021-04-20; identificationRemarks: identified by DNA and morphology; **Event:** eventID: UK1_AB01_EB04; samplingProtocol: Brenke Epibenthic Sledge; eventDate: 2013-10-17; eventTime: 01:50; habitat: Abyssal plain; fieldNotes: Collected from epi net (on the epibenthic sledge); **Record Level:** language: en; institutionCode: NHMUK; collectionCode: ZOO; datasetName: ABYSSLINE; basisOfRecord: PreservedSpecimen

##### Distribution

Eastern Clarion-Clipperton Zone, central Pacific Ocean.

##### Diagnosis

**Figure 114.**
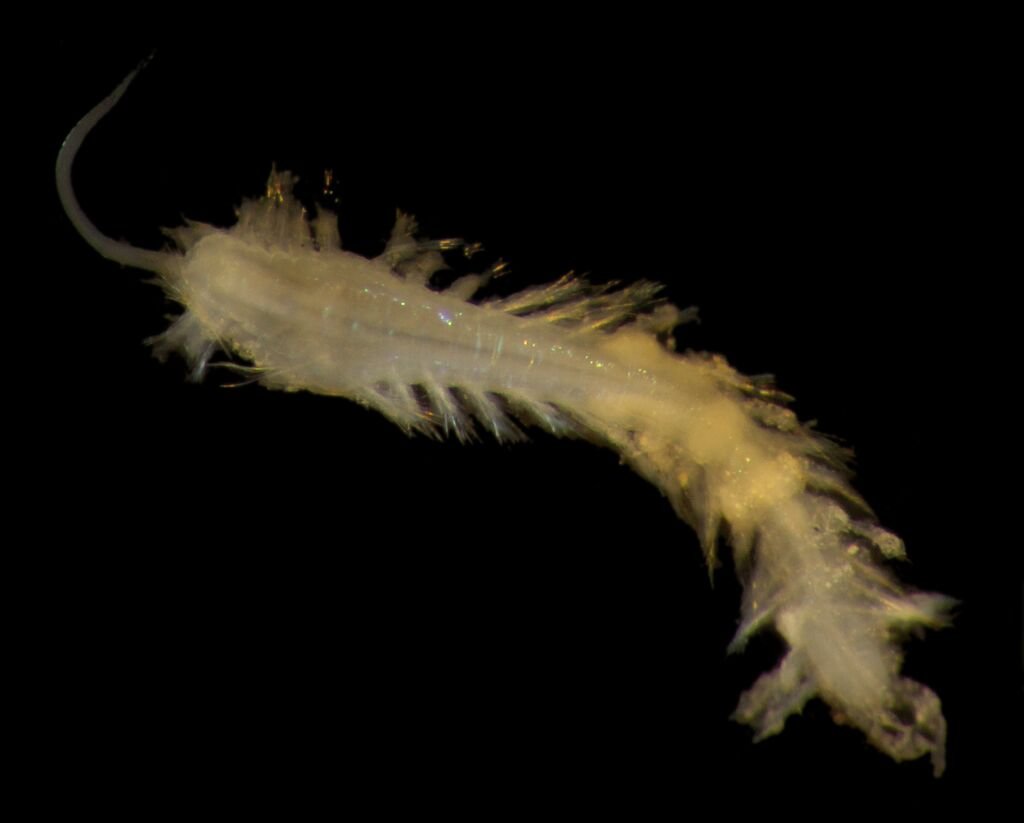
Sigalioniodae sp. (NHM_342), live specimen NHM_605 in dorsal view.

Damaged specimens (Fig. 114) consistent with placement within family Sigalionidae, based on morphology and DNA.

#### Sigalionidae sp. (NHM_881)

7AACFD49-4CB4-5A58-8920-4D710005C423

##### Materials

- **Type status:** Other material. **Occurrence:** catalogNumber: NHMUK ANEA 2023.580; recordNumber: NHM_1386; recordedBy: Adrian Glover | Helena Wiklund | Thomas Dahlgren | Madeleine Brasier; individualCount: 1; preparations: specimen stored in 80% non-denatured ethanol aqueous solution | DNA voucher stored in buffer; otherCatalogNumbers: 0174127357; associatedSequences: OQ738582 (COI); occurrenceID: 4A552BFD-FCBD-5014-92CA-F1B6215C25F7; **Taxon:** taxonConceptID: Sigalionidae sp. (NHM_881); scientificName: Sigalionidae; kingdom: Animalia; phylum: Annelida; class: Polychaeta; order: Phyllodocida; family: Sigalionidae; taxonRank: family; scientificNameAuthorship: Kinberg, 1856; **Location:** waterBody: Pacific; stateProvince: Clarion Clipperton Zone; locality: Ocean Mineral Singapore exploration claim Stratum A; verbatimLocality: OMS Stratum A; maximumDepthInMeters: 4044; locationRemarks: Deployment BC12; at Station S6; from R/V Thomas G. Thompson Cruise no. TN319; verbatimLatitude: 12'08.695; verbatimLongitude: 117'19.526; decimalLatitude: 12.14492; decimalLongitude: -117.32543; geodeticDatum: WGS84; **Identification:** identifiedBy: Helena Wiklund | Lenka Neal | Thomas Dahlgren | Adrian Glover | Madeleine Brasier | Regan Drennan | Eva Stewart; dateIdentified: 2021-04-20; identificationRemarks: identified by DNA and morphology; **Event:** eventID: OMS1_AB02_BC12; samplingProtocol: USNEL Box Core; eventDate: 2015-03-02; eventTime: 02:20; habitat: Abyssal plain; fieldNotes: Collected from 0-2 cm layer of box core using a 300 micron sieve; **Record Level:** language: en; institutionCode: NHMUK; collectionCode: ZOO; datasetName: ABYSSLINE; basisOfRecord: PreservedSpecimen
- **Type status:** Other material. **Occurrence:** catalogNumber: NHMUK ANEA 2023.579; recordNumber: NHM_0881; recordedBy: Adrian Glover | Helena Wiklund | Thomas Dahlgren | Madeleine Brasier; individualCount: 1; preparations: specimen stored in 80% non-denatured ethanol aqueous solution | DNA voucher stored in buffer; otherCatalogNumbers: 0174127378; associatedSequences: OQ746863 (18S) | OQ738541 (COI); occurrenceID: ED77ABD5-4C7D-59B2-ABD2-AD46F45415AA; **Taxon:** taxonConceptID: Sigalionidae sp. (NHM_881); scientificName: Sigalionidae; kingdom: Animalia; phylum: Annelida; class: Polychaeta; order: Phyllodocida; family: Sigalionidae; taxonRank: family; scientificNameAuthorship: Kinberg, 1856; **Location:** waterBody: Pacific; stateProvince: Clarion Clipperton Zone; locality: UK Seabed Resources Ltd exploration area UK-1 Stratum B; verbatimLocality: UK1 Stratum B; maximumDepthInMeters: 4198; locationRemarks: Deployment EB03; at Station U4; from R/V Thomas G. Thompson Cruise no. TN319; verbatimLatitude: 12'34.28; verbatimLongitude: 116'36.63; decimalLatitude: 12.57133; decimalLongitude: -116.6105; geodeticDatum: WGS84; **Identification:** identifiedBy: Helena Wiklund | Lenka Neal | Thomas Dahlgren | Adrian Glover | Madeleine Brasier | Regan Drennan | Eva Stewart; dateIdentified: 2021-04-20; identificationRemarks: identified by DNA and morphology; **Event:** eventID: UK1_AB02_EB03; samplingProtocol: Brenke Epibenthic Sledge; eventDate: 2015-02-23; eventTime: 05:39; habitat: Abyssal plain; fieldNotes: Collected from epi net (on the epibenthic sledge); **Record Level:** language: en; institutionCode: NHMUK; collectionCode: ZOO; datasetName: ABYSSLINE; basisOfRecord: PreservedSpecimen

##### Distribution

Eastern Clarion-Clipperton Zone, central Pacific Ocean.

##### Diagnosis

Damaged specimens (Fig. 115) consistent with placement within family Sigalionidae, based on morphology and DNA.

#### Sigalionidae sp. (NHM_1093)

E2F4944C-69B2-5C43-8769-27ED9EC1847E

##### Materials

- **Type status:** Other material. **Occurrence:** catalogNumber: NHMUK ANEA 2023.569; recordNumber: NHM_1093; recordedBy: Adrian Glover | Helena Wiklund | Thomas Dahlgren | Madeleine Brasier; individualCount: 1; preparations: specimen stored in 80% non-denatured ethanol aqueous solution | DNA voucher stored in buffer; otherCatalogNumbers: 0174126787; associatedSequences: OQ746615 (16S) | OQ746875 (18S); occurrenceID: 9C1DA962-69F9-554E-BCA3-0F65FE4C4914; **Taxon:** taxonConceptID: Sigalionidae sp. (NHM_1093); scientificName: Sigalionidae; kingdom: Animalia; phylum: Annelida; class: Polychaeta; order: Phyllodocida; family: Sigalionidae; taxonRank: family; scientificNameAuthorship: Kinberg, 1856; **Location:** waterBody: Pacific; stateProvince: Clarion Clipperton Zone; locality: Ocean Mineral Singapore exploration claim Stratum A; verbatimLocality: OMS Stratum A; maximumDepthInMeters: 4100; locationRemarks: Deployment EB05; at Station S2; from R/V Thomas G. Thompson Cruise no. TN319; verbatimLatitude: 12'06.93; verbatimLongitude: 117'09.87; decimalLatitude: 12.1155; decimalLongitude: -117.1645; geodeticDatum: WGS84; **Identification:** identifiedBy: Helena Wiklund | Lenka Neal | Thomas Dahlgren | Adrian Glover | Madeleine Brasier | Regan Drennan | Eva Stewart; dateIdentified: 2021-04-20; identificationRemarks: identified by DNA and morphology; **Event:** eventID: OMS1_AB02_EB05; samplingProtocol: Brenke Epibenthic Sledge; eventDate: 2015-02-26; eventTime: 21:29; habitat: Abyssal plain; fieldNotes: Collected from epi net (on the epibenthic sledge); **Record Level:** language: en; institutionCode: NHMUK; collectionCode: ZOO; datasetName: ABYSSLINE; basisOfRecord: PreservedSpecimen

##### Distribution

Eastern Clarion-Clipperton Zone, central Pacific Ocean.

##### Diagnosis

**Figure 116.**
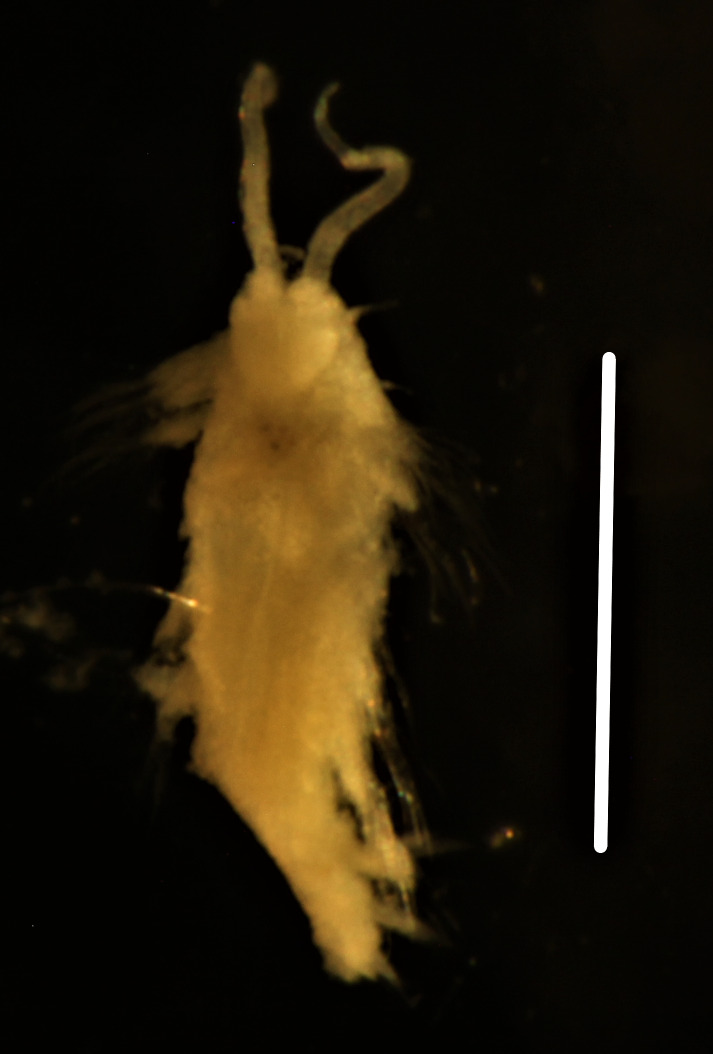
Sigalionidae sp. (NHM_1093), anterior end of preserved specimen NHM_1093 in dorsal view.

Damaged specimen (Fig. 116) consistent with placement within family Sigalionidae, based on morphology and DNA.

### Sipuncula Stephen, 1964

#### Sipuncula sp. (NHM_126)

71EB8A75-DD30-5CEB-9134-8E4439A8E0E4

##### Materials

- **Type status:** Other material. **Occurrence:** catalogNumber: NHMUK ANEA 2023.590; recordNumber: NHM_1265; recordedBy: Adrian Glover | Helena Wiklund | Thomas Dahlgren | Madeleine Brasier; individualCount: 1; preparations: specimen stored in 80% non-denatured ethanol aqueous solution | DNA voucher stored in buffer; otherCatalogNumbers: 0174126797; associatedSequences: OQ746647 (16S); occurrenceID: 17DF65A3-BBDB-5CEB-869C-ED8024613204; **Taxon:** taxonConceptID: Sipuncula sp. (NHM_126); scientificName: Sipuncula; kingdom: Animalia; phylum: Annelida; order: Sipuncula; taxonRank: family; scientificNameAuthorship: Stephen, 1965; **Location:** waterBody: Pacific; stateProvince: Clarion Clipperton Zone; locality: Ocean Mineral Singapore exploration claim Stratum A; verbatimLocality: OMS Stratum A; maximumDepthInMeters: 4302; locationRemarks: Deployment EB06; at Station S5; from R/V Thomas G. Thompson Cruise no. TN319; verbatimLatitude: 12'15.44; verbatimLongitude: 117'18.13; decimalLatitude: 12.25733; decimalLongitude: -117.30217; geodeticDatum: WGS84; **Identification:** identifiedBy: Helena Wiklund | Lenka Neal | Thomas Dahlgren | Adrian Glover | Madeleine Brasier | Regan Drennan | Eva Stewart; dateIdentified: 2021-04-20; identificationRemarks: identified by DNA and morphology; **Event:** eventID: OMS1_AB02_EB06; samplingProtocol: Brenke Epibenthic Sledge; eventDate: 2015-03-01; eventTime: 04:02; habitat: Abyssal plain; fieldNotes: Collected from epi net (on the epibenthic sledge); **Record Level:** language: en; institutionCode: NHMUK; collectionCode: ZOO; datasetName: ABYSSLINE; basisOfRecord: PreservedSpecimen
- **Type status:** Other material. **Occurrence:** catalogNumber: NHMUK ANEA 2023.592; recordNumber: NHM_2189; recordedBy: Adrian Glover | Helena Wiklund | Thomas Dahlgren | Madeleine Brasier; individualCount: 1; preparations: specimen stored in 80% non-denatured ethanol aqueous solution | DNA voucher stored in buffer; otherCatalogNumbers: 0174126736; associatedSequences: OQ746761 (16S); occurrenceID: 16D7FAF4-7CA5-5F07-9503-B127D99E7176; **Taxon:** taxonConceptID: Sipuncula sp. (NHM_126); scientificName: Sipuncula; kingdom: Animalia; phylum: Annelida; order: Sipuncula; taxonRank: family; scientificNameAuthorship: Stephen, 1965; **Location:** waterBody: Pacific; stateProvince: Clarion Clipperton Zone; locality: Ocean Mineral Singapore exploration claim Stratum A; verbatimLocality: OMS Stratum A; maximumDepthInMeters: 4122; locationRemarks: Deployment EB04; at Station S1; from R/V Thomas G. Thompson Cruise no. TN319; verbatimLatitude: 12'08.02; verbatimLongitude: 117'17.52; decimalLatitude: 12.13367; decimalLongitude: -117.292; geodeticDatum: WGS84; **Identification:** identifiedBy: Helena Wiklund | Lenka Neal | Thomas Dahlgren | Adrian Glover | Madeleine Brasier | Regan Drennan | Eva Stewart; dateIdentified: 2021-04-20; identificationRemarks: identified by DNA and morphology; **Event:** eventID: OMS1_AB02_EB04; samplingProtocol: Brenke Epibenthic Sledge; eventDate: 2015-02-24; eventTime: 19:10; habitat: Abyssal plain; fieldNotes: Collected from supra net (on the epibenthic sledge); **Record Level:** language: en; institutionCode: NHMUK; collectionCode: ZOO; datasetName: ABYSSLINE; basisOfRecord: PreservedSpecimen
- **Type status:** Other material. **Occurrence:** catalogNumber: NHMUK ANEA 2023.589; recordNumber: NHM_1128; recordedBy: Adrian Glover | Helena Wiklund | Thomas Dahlgren | Madeleine Brasier; individualCount: 1; preparations: specimen stored in 80% non-denatured ethanol aqueous solution | DNA voucher stored in buffer; otherCatalogNumbers: 0174126789; associatedSequences: OQ746619 (16S); occurrenceID: BB6FF6ED-F840-5391-AE1E-F8CFEE6727B9; **Taxon:** taxonConceptID: Sipuncula sp. (NHM_126); scientificName: Sipuncula; kingdom: Animalia; phylum: Annelida; order: Sipuncula; taxonRank: family; scientificNameAuthorship: Stephen, 1965; **Location:** waterBody: Pacific; stateProvince: Clarion Clipperton Zone; locality: Ocean Mineral Singapore exploration claim Stratum A; verbatimLocality: OMS Stratum A; maximumDepthInMeters: 4100; locationRemarks: Deployment EB05; at Station S2; from R/V Thomas G. Thompson Cruise no. TN319; verbatimLatitude: 12'06.93; verbatimLongitude: 117'09.87; decimalLatitude: 12.1155; decimalLongitude: -117.1645; geodeticDatum: WGS84; **Identification:** identifiedBy: Helena Wiklund | Lenka Neal | Thomas Dahlgren | Adrian Glover | Madeleine Brasier | Regan Drennan | Eva Stewart; dateIdentified: 2021-04-20; identificationRemarks: identified by DNA and morphology; **Event:** eventID: OMS1_AB02_EB05; samplingProtocol: Brenke Epibenthic Sledge; eventDate: 2015-02-26; eventTime: 21:29; habitat: Abyssal plain; fieldNotes: Collected from epi net (on the epibenthic sledge); **Record Level:** language: en; institutionCode: NHMUK; collectionCode: ZOO; datasetName: ABYSSLINE; basisOfRecord: PreservedSpecimen
- **Type status:** Other material. **Occurrence:** catalogNumber: NHMUK ANEA 2023.587; recordNumber: NHM_0126; recordedBy: Adrian Glover | Helena Wiklund | Thomas Dahlgren | Magdalena Georgieva; individualCount: 1; preparations: specimen stored in 80% non-denatured ethanol aqueous solution | DNA voucher stored in buffer; otherCatalogNumbers: 0174127349; associatedSequences: OQ746478 (16S) | OQ746799 (18S); occurrenceID: 4CA6611D-73F7-55C5-98C4-8DB040B82159; **Taxon:** taxonConceptID: Sipuncula sp. (NHM_126); scientificName: Sipuncula; kingdom: Animalia; phylum: Annelida; order: Sipuncula; taxonRank: family; scientificNameAuthorship: Stephen, 1965; **Location:** waterBody: Pacific; stateProvince: Clarion Clipperton Zone; locality: UK Seabed Resources Ltd exploration area UK-1 Stratum A; verbatimLocality: UK1 Stratum A; maximumDepthInMeters: 4080; locationRemarks: Deployment EB02; at Station C; from R/V Melville Cruise no. MV1313; verbatimLatitude: 13°45.500; verbatimLongitude: 116°41.911; decimalLatitude: 13.75833; decimalLongitude: -116.69852; geodeticDatum: WGS84; **Identification:** identifiedBy: Helena Wiklund | Lenka Neal | Thomas Dahlgren | Adrian Glover | Madeleine Brasier | Regan Drennan | Eva Stewart; dateIdentified: 2021-04-20; identificationRemarks: identified by DNA and morphology; **Event:** eventID: UK1_AB01_EB02; samplingProtocol: Brenke Epibenthic Sledge; eventDate: 2013-10-11; eventTime: 10:32; habitat: Abyssal plain; fieldNotes: Collected from epi net (on the epibenthic sledge); **Record Level:** language: en; institutionCode: NHMUK; collectionCode: ZOO; datasetName: ABYSSLINE; basisOfRecord: PreservedSpecimen
- **Type status:** Other material. **Occurrence:** catalogNumber: NHMUK ANEA 2023.591; recordNumber: NHM_1450; recordedBy: Adrian Glover | Helena Wiklund | Thomas Dahlgren | Madeleine Brasier; individualCount: 1; preparations: specimen stored in 80% non-denatured ethanol aqueous solution | DNA voucher stored in buffer; otherCatalogNumbers: 0174126799; associatedSequences: OQ746680 (16S); occurrenceID: 1C7C7494-15C2-5540-8404-72AD3EA2EBFE; **Taxon:** taxonConceptID: Sipuncula sp. (NHM_126); scientificName: Sipuncula; kingdom: Animalia; phylum: Annelida; order: Sipuncula; taxonRank: family; scientificNameAuthorship: Stephen, 1965; **Location:** waterBody: Pacific; stateProvince: Clarion Clipperton Zone; locality: UK Seabed Resources Ltd exploration area UK-1 Stratum B; verbatimLocality: UK1 Stratum B; maximumDepthInMeters: 4137; locationRemarks: Deployment EB07; at Station U7; from R/V Thomas G. Thompson Cruise no. TN319; verbatimLatitude: 12'27.26; verbatimLongitude: 116'36.77; decimalLatitude: 12.45433; decimalLongitude: -116.61283; geodeticDatum: WGS84; **Identification:** identifiedBy: Helena Wiklund | Lenka Neal | Thomas Dahlgren | Adrian Glover | Madeleine Brasier | Regan Drennan | Eva Stewart; dateIdentified: 2021-04-20; identificationRemarks: identified by DNA and morphology; **Event:** eventID: UK1_AB02_EB07; samplingProtocol: Brenke Epibenthic Sledge; eventDate: 2015-03-03; eventTime: 20:40; habitat: Abyssal plain; fieldNotes: Collected from epi net (on the epibenthic sledge); **Record Level:** language: en; institutionCode: NHMUK; collectionCode: ZOO; datasetName: ABYSSLINE; basisOfRecord: PreservedSpecimen
- **Type status:** Other material. **Occurrence:** catalogNumber: NHMUK ANEA 2023.588; recordNumber: NHM_0745; recordedBy: Adrian Glover | Helena Wiklund | Thomas Dahlgren | Madeleine Brasier; individualCount: 1; preparations: specimen stored in 80% non-denatured ethanol aqueous solution | DNA voucher stored in buffer; otherCatalogNumbers: 0174126811; associatedSequences: OQ746547 (16S); occurrenceID: D8A3814B-3B8E-5088-A493-3E0BEAA8EC94; **Taxon:** taxonConceptID: Sipuncula sp. (NHM_126); scientificName: Sipuncula; kingdom: Animalia; phylum: Annelida; order: Sipuncula; taxonRank: family; scientificNameAuthorship: Stephen, 1965; **Location:** waterBody: Pacific; stateProvince: Clarion Clipperton Zone; locality: UK Seabed Resources Ltd exploration area UK-1 Stratum B; verbatimLocality: UK1 Stratum B; maximumDepthInMeters: 4425; locationRemarks: Deployment EB02; at Station U5; from R/V Thomas G. Thompson Cruise no. TN319; verbatimLatitude: 12'32.23; verbatimLongitude: 116'36.25; decimalLatitude: 12.53717; decimalLongitude: -116.60417; geodeticDatum: WGS84; **Identification:** identifiedBy: Helena Wiklund | Lenka Neal | Thomas Dahlgren | Adrian Glover | Madeleine Brasier | Regan Drennan | Eva Stewart; dateIdentified: 2021-04-20; identificationRemarks: identified by DNA and morphology; **Event:** eventID: UK1_AB02_EB02; samplingProtocol: Brenke Epibenthic Sledge; eventDate: 2015-02-20; eventTime: 06:24; habitat: Abyssal plain; fieldNotes: Collected from epi net (on the epibenthic sledge); **Record Level:** language: en; institutionCode: NHMUK; collectionCode: ZOO; datasetName: ABYSSLINE; basisOfRecord: PreservedSpecimen

##### Distribution

Eastern Clarion-Clipperton Zone, central Pacific Ocean.

##### Diagnosis

Specimens (Fig. 117) consistent with placement within Sipuncula, based on morphology and DNA.

#### Sipuncula sp. (NHM_148)

35D02AF1-0A36-5E58-A8FD-728C8CD3BCE3

##### Materials

- **Type status:** Other material. **Occurrence:** catalogNumber: NHMUK ANEA 2023.595; recordNumber: NHM_0148; recordedBy: Adrian Glover | Helena Wiklund | Thomas Dahlgren | Magdalena Georgieva; individualCount: 1; preparations: specimen stored in 80% non-denatured ethanol aqueous solution | DNA voucher stored in buffer; otherCatalogNumbers: 0174127363; associatedSequences: OQ746801 (18S); occurrenceID: B03973D6-070C-5BA2-9272-0E5076CA1FED; **Taxon:** taxonConceptID: Sipuncula sp. (NHM_148); scientificName: Sipuncula; kingdom: Animalia; phylum: Annelida; order: Sipuncula; taxonRank: family; scientificNameAuthorship: Stephen, 1965; **Location:** waterBody: Pacific; stateProvince: Clarion Clipperton Zone; locality: UK Seabed Resources Ltd exploration area UK-1 Stratum A; verbatimLocality: UK1 Stratum A; maximumDepthInMeters: 4080; locationRemarks: Deployment EB02; at Station C; from R/V Melville Cruise no. MV1313; verbatimLatitude: 13°45.500; verbatimLongitude: 116°41.911; decimalLatitude: 13.75833; decimalLongitude: -116.69852; geodeticDatum: WGS84; **Identification:** identifiedBy: Helena Wiklund | Lenka Neal | Thomas Dahlgren | Adrian Glover | Madeleine Brasier | Regan Drennan | Eva Stewart; dateIdentified: 2021-04-20; identificationRemarks: identified by DNA and morphology; **Event:** eventID: UK1_AB01_EB02; samplingProtocol: Brenke Epibenthic Sledge; eventDate: 2013-10-11; eventTime: 10:32; habitat: Abyssal plain; fieldNotes: Collected from epi net (on the epibenthic sledge); **Record Level:** language: en; institutionCode: NHMUK; collectionCode: ZOO; datasetName: ABYSSLINE; basisOfRecord: PreservedSpecimen

##### Distribution

Eastern Clarion-Clipperton Zone, central Pacific Ocean.

##### Diagnosis

**Figure 118.**
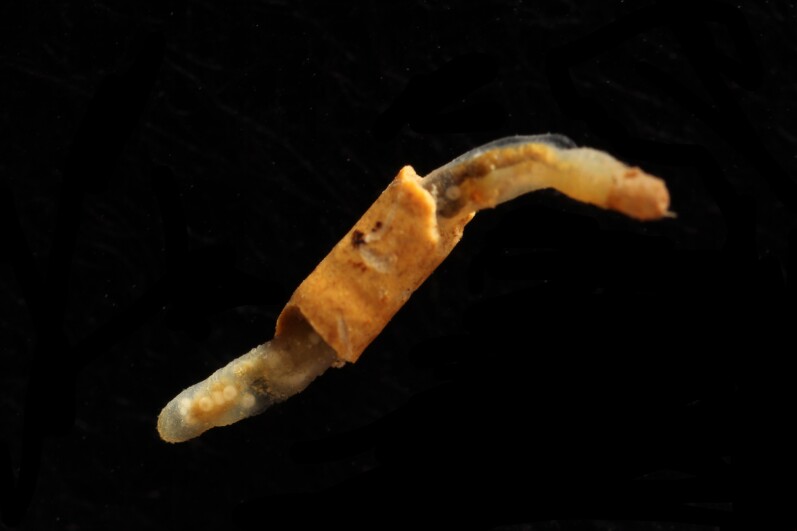
Sipuncula sp. (NHM_148), live specimen NHM_148, with remnants of the tube.

Specimen (Fig. 118) consistent with placement within Sipuncula, based on morphology and DNA.

#### Sipuncula sp. (NHM_715)

484D7F07-A6F8-556C-BAE2-6290B49EEAB6

##### Materials

- **Type status:** Other material. **Occurrence:** catalogNumber: NHMUK ANEA 2023.605; recordNumber: NHM_2191; recordedBy: Adrian Glover | Helena Wiklund | Thomas Dahlgren | Madeleine Brasier; individualCount: 1; preparations: specimen stored in 80% non-denatured ethanol aqueous solution | DNA voucher stored in buffer; otherCatalogNumbers: 0174126800; associatedSequences: OQ746762 (16S); occurrenceID: 0AF0D541-6860-56C7-91B5-90B2D2465F30; **Taxon:** taxonConceptID: Sipuncula sp. (NHM_715); scientificName: Sipuncula; kingdom: Animalia; phylum: Annelida; order: Sipuncula; taxonRank: family; scientificNameAuthorship: Stephen, 1965; **Location:** waterBody: Pacific; stateProvince: Clarion Clipperton Zone; locality: Ocean Mineral Singapore exploration claim Stratum A; verbatimLocality: OMS Stratum A; maximumDepthInMeters: 4302; locationRemarks: Deployment EB06; at Station S5; from R/V Thomas G. Thompson Cruise no. TN319; verbatimLatitude: 12'15.44; verbatimLongitude: 117'18.13; decimalLatitude: 12.25733; decimalLongitude: -117.30217; geodeticDatum: WGS84; **Identification:** identifiedBy: Helena Wiklund | Lenka Neal | Thomas Dahlgren | Adrian Glover | Madeleine Brasier | Regan Drennan | Eva Stewart; dateIdentified: 2021-04-20; identificationRemarks: identified by DNA and morphology; **Event:** eventID: OMS1_AB02_EB06; samplingProtocol: Brenke Epibenthic Sledge; eventDate: 2015-03-01; eventTime: 04:02; habitat: Abyssal plain; fieldNotes: Collected from supra net (on the epibenthic sledge); **Record Level:** language: en; institutionCode: NHMUK; collectionCode: ZOO; datasetName: ABYSSLINE; basisOfRecord: PreservedSpecimen
- **Type status:** Other material. **Occurrence:** catalogNumber: NHMUK ANEA 2023.603; recordNumber: NHM_1126; recordedBy: Adrian Glover | Helena Wiklund | Thomas Dahlgren | Madeleine Brasier; individualCount: 1; preparations: specimen stored in 80% non-denatured ethanol aqueous solution | DNA voucher stored in buffer; otherCatalogNumbers: 0174126739; associatedSequences: OQ746618 (16S) | OQ738557 (COI); occurrenceID: 4D70E954-4C0F-5841-8E83-2377EF0871BF; **Taxon:** taxonConceptID: Sipuncula sp. (NHM_715); scientificName: Sipuncula; kingdom: Animalia; phylum: Annelida; order: Sipuncula; taxonRank: family; scientificNameAuthorship: Stephen, 1965; **Location:** waterBody: Pacific; stateProvince: Clarion Clipperton Zone; locality: Ocean Mineral Singapore exploration claim Stratum A; verbatimLocality: OMS Stratum A; maximumDepthInMeters: 4100; locationRemarks: Deployment EB05; at Station S2; from R/V Thomas G. Thompson Cruise no. TN319; verbatimLatitude: 12'06.93; verbatimLongitude: 117'09.87; decimalLatitude: 12.1155; decimalLongitude: -117.1645; geodeticDatum: WGS84; **Identification:** identifiedBy: Helena Wiklund | Lenka Neal | Thomas Dahlgren | Adrian Glover | Madeleine Brasier | Regan Drennan | Eva Stewart; dateIdentified: 2021-04-20; identificationRemarks: identified by DNA and morphology; **Event:** eventID: OMS1_AB02_EB05; samplingProtocol: Brenke Epibenthic Sledge; eventDate: 2015-02-26; eventTime: 21:29; habitat: Abyssal plain; fieldNotes: Collected from epi net (on the epibenthic sledge); **Record Level:** language: en; institutionCode: NHMUK; collectionCode: ZOO; datasetName: ABYSSLINE; basisOfRecord: PreservedSpecimen
- **Type status:** Other material. **Occurrence:** catalogNumber: NHMUK ANEA 2023.604; recordNumber: NHM_1130; recordedBy: Adrian Glover | Helena Wiklund | Thomas Dahlgren | Madeleine Brasier; individualCount: 1; preparations: specimen stored in 80% non-denatured ethanol aqueous solution | DNA voucher stored in buffer; otherCatalogNumbers: 0174126724; associatedSequences: OQ746620 (16S) | OQ738558 (COI); occurrenceID: 5B179B3F-4858-5C5C-AD20-829401E6EB69; **Taxon:** taxonConceptID: Sipuncula sp. (NHM_715); scientificName: Sipuncula; kingdom: Animalia; phylum: Annelida; order: Sipuncula; taxonRank: family; scientificNameAuthorship: Stephen, 1965; **Location:** waterBody: Pacific; stateProvince: Clarion Clipperton Zone; locality: Ocean Mineral Singapore exploration claim Stratum A; verbatimLocality: OMS Stratum A; maximumDepthInMeters: 4100; locationRemarks: Deployment EB05; at Station S2; from R/V Thomas G. Thompson Cruise no. TN319; verbatimLatitude: 12'06.93; verbatimLongitude: 117'09.87; decimalLatitude: 12.1155; decimalLongitude: -117.1645; geodeticDatum: WGS84; **Identification:** identifiedBy: Helena Wiklund | Lenka Neal | Thomas Dahlgren | Adrian Glover | Madeleine Brasier | Regan Drennan | Eva Stewart; dateIdentified: 2021-04-20; identificationRemarks: identified by DNA and morphology; **Event:** eventID: OMS1_AB02_EB05; samplingProtocol: Brenke Epibenthic Sledge; eventDate: 2015-02-26; eventTime: 21:29; habitat: Abyssal plain; fieldNotes: Collected from epi net (on the epibenthic sledge); **Record Level:** language: en; institutionCode: NHMUK; collectionCode: ZOO; datasetName: ABYSSLINE; basisOfRecord: PreservedSpecimen
- **Type status:** Other material. **Occurrence:** catalogNumber: NHMUK ANEA 2023.599; recordNumber: NHM_0923; recordedBy: Adrian Glover | Helena Wiklund | Thomas Dahlgren | Madeleine Brasier; individualCount: 1; preparations: specimen stored in 80% non-denatured ethanol aqueous solution | DNA voucher stored in buffer; otherCatalogNumbers: 0174126748; associatedSequences: OQ746590 (16S) | OQ738548 (COI); occurrenceID: 6C893635-05C6-5D8B-889C-7D47A34416AC; **Taxon:** taxonConceptID: Sipuncula sp. (NHM_715); scientificName: Sipuncula; kingdom: Animalia; phylum: Annelida; order: Sipuncula; taxonRank: family; scientificNameAuthorship: Stephen, 1965; **Location:** waterBody: Pacific; stateProvince: Clarion Clipperton Zone; locality: UK Seabed Resources Ltd exploration area UK-1 Stratum B; verbatimLocality: UK1 Stratum B; maximumDepthInMeters: 4198; locationRemarks: Deployment EB03; at Station U4; from R/V Thomas G. Thompson Cruise no. TN319; verbatimLatitude: 12'34.28; verbatimLongitude: 116'36.63; decimalLatitude: 12.57133; decimalLongitude: -116.6105; geodeticDatum: WGS84; **Identification:** identifiedBy: Helena Wiklund | Lenka Neal | Thomas Dahlgren | Adrian Glover | Madeleine Brasier | Regan Drennan | Eva Stewart; dateIdentified: 2021-04-20; identificationRemarks: identified by DNA and morphology; **Event:** eventID: UK1_AB02_EB03; samplingProtocol: Brenke Epibenthic Sledge; eventDate: 2015-02-23; eventTime: 05:39; habitat: Abyssal plain; fieldNotes: Collected from epi net (on the epibenthic sledge); **Record Level:** language: en; institutionCode: NHMUK; collectionCode: ZOO; datasetName: ABYSSLINE; basisOfRecord: PreservedSpecimen
- **Type status:** Other material. **Occurrence:** catalogNumber: NHMUK ANEA 2023.600; recordNumber: NHM_0923A; recordedBy: Adrian Glover | Helena Wiklund | Thomas Dahlgren | Madeleine Brasier; individualCount: 1; preparations: specimen stored in 80% non-denatured ethanol aqueous solution | DNA voucher stored in buffer; otherCatalogNumbers: 0174126772; associatedSequences: OQ746589 (16S) | OQ738547 (COI); occurrenceID: 4E580A92-7CF4-5149-9CAF-F9DF09E5EFE4; **Taxon:** taxonConceptID: Sipuncula sp. (NHM_715); scientificName: Sipuncula; kingdom: Animalia; phylum: Annelida; order: Sipuncula; taxonRank: family; scientificNameAuthorship: Stephen, 1965; **Location:** waterBody: Pacific; stateProvince: Clarion Clipperton Zone; locality: UK Seabed Resources Ltd exploration area UK-1 Stratum B; verbatimLocality: UK1 Stratum B; maximumDepthInMeters: 4198; locationRemarks: Deployment EB03; at Station U4; from R/V Thomas G. Thompson Cruise no. TN319; verbatimLatitude: 12'34.28; verbatimLongitude: 116'36.63; decimalLatitude: 12.57133; decimalLongitude: -116.6105; geodeticDatum: WGS84; **Identification:** identifiedBy: Helena Wiklund | Lenka Neal | Thomas Dahlgren | Adrian Glover | Madeleine Brasier | Regan Drennan | Eva Stewart; dateIdentified: 2021-04-20; identificationRemarks: identified by DNA and morphology; **Event:** eventID: UK1_AB02_EB03; samplingProtocol: Brenke Epibenthic Sledge; eventDate: 2015-02-23; eventTime: 05:39; habitat: Abyssal plain; fieldNotes: Collected from epi net (on the epibenthic sledge); **Record Level:** language: en; institutionCode: NHMUK; collectionCode: ZOO; datasetName: ABYSSLINE; basisOfRecord: PreservedSpecimen
- **Type status:** Other material. **Occurrence:** catalogNumber: NHMUK ANEA 2023.601; recordNumber: NHM_0935; recordedBy: Adrian Glover | Helena Wiklund | Thomas Dahlgren | Madeleine Brasier; individualCount: 1; preparations: specimen stored in 80% non-denatured ethanol aqueous solution | DNA voucher stored in buffer; otherCatalogNumbers: 0174126763; associatedSequences: OQ746591 (16S) | OQ738549 (COI); occurrenceID: 92BB4D24-F341-5B7B-98AC-1062912B42B7; **Taxon:** taxonConceptID: Sipuncula sp. (NHM_715); scientificName: Sipuncula; kingdom: Animalia; phylum: Annelida; order: Sipuncula; taxonRank: family; scientificNameAuthorship: Stephen, 1965; **Location:** waterBody: Pacific; stateProvince: Clarion Clipperton Zone; locality: UK Seabed Resources Ltd exploration area UK-1 Stratum B; verbatimLocality: UK1 Stratum B; maximumDepthInMeters: 4198; locationRemarks: Deployment EB03; at Station U4; from R/V Thomas G. Thompson Cruise no. TN319; verbatimLatitude: 12'34.28; verbatimLongitude: 116'36.63; decimalLatitude: 12.57133; decimalLongitude: -116.6105; geodeticDatum: WGS84; **Identification:** identifiedBy: Helena Wiklund | Lenka Neal | Thomas Dahlgren | Adrian Glover | Madeleine Brasier | Regan Drennan | Eva Stewart; dateIdentified: 2021-04-20; identificationRemarks: identified by DNA and morphology; **Event:** eventID: UK1_AB02_EB03; samplingProtocol: Brenke Epibenthic Sledge; eventDate: 2015-02-23; eventTime: 05:39; habitat: Abyssal plain; fieldNotes: Collected from epi net (on the epibenthic sledge); **Record Level:** language: en; institutionCode: NHMUK; collectionCode: ZOO; datasetName: ABYSSLINE; basisOfRecord: PreservedSpecimen
- **Type status:** Other material. **Occurrence:** catalogNumber: NHMUK ANEA 2023.602; recordNumber: NHM_0938; recordedBy: Adrian Glover | Helena Wiklund | Thomas Dahlgren | Madeleine Brasier; individualCount: 1; preparations: specimen stored in 80% non-denatured ethanol aqueous solution | DNA voucher stored in buffer; otherCatalogNumbers: 0174126810; associatedSequences: OQ746592 (16S) | OQ738550 (COI); occurrenceID: F554899C-1665-50FE-951E-C6DD7885E316; **Taxon:** taxonConceptID: Sipuncula sp. (NHM_715); scientificName: Sipuncula; kingdom: Animalia; phylum: Annelida; order: Sipuncula; taxonRank: family; scientificNameAuthorship: Stephen, 1965; **Location:** waterBody: Pacific; stateProvince: Clarion Clipperton Zone; locality: UK Seabed Resources Ltd exploration area UK-1 Stratum B; verbatimLocality: UK1 Stratum B; maximumDepthInMeters: 4198; locationRemarks: Deployment EB03; at Station U4; from R/V Thomas G. Thompson Cruise no. TN319; verbatimLatitude: 12'34.28; verbatimLongitude: 116'36.63; decimalLatitude: 12.57133; decimalLongitude: -116.6105; geodeticDatum: WGS84; **Identification:** identifiedBy: Helena Wiklund | Lenka Neal | Thomas Dahlgren | Adrian Glover | Madeleine Brasier | Regan Drennan | Eva Stewart; dateIdentified: 2021-04-20; identificationRemarks: identified by DNA and morphology; **Event:** eventID: UK1_AB02_EB03; samplingProtocol: Brenke Epibenthic Sledge; eventDate: 2015-02-23; eventTime: 05:39; habitat: Abyssal plain; fieldNotes: Collected from epi net (on the epibenthic sledge); **Record Level:** language: en; institutionCode: NHMUK; collectionCode: ZOO; datasetName: ABYSSLINE; basisOfRecord: PreservedSpecimen
- **Type status:** Other material. **Occurrence:** catalogNumber: NHMUK ANEA 2023.597; recordNumber: NHM_0715; recordedBy: Adrian Glover | Helena Wiklund | Thomas Dahlgren | Madeleine Brasier; individualCount: 1; preparations: specimen stored in 80% non-denatured ethanol aqueous solution | DNA voucher stored in buffer; otherCatalogNumbers: 0174126723; associatedSequences: OQ746543 (16S) | OQ738533 (COI); occurrenceID: 4738002E-6CAC-5F59-BB22-F69769F0E798; **Taxon:** taxonConceptID: Sipuncula sp. (NHM_715); scientificName: Sipuncula; kingdom: Animalia; phylum: Annelida; order: Sipuncula; taxonRank: family; scientificNameAuthorship: Stephen, 1965; **Location:** waterBody: Pacific; stateProvince: Clarion Clipperton Zone; locality: UK Seabed Resources Ltd exploration area UK-1 Stratum B; verbatimLocality: UK1 Stratum B; maximumDepthInMeters: 4425; locationRemarks: Deployment EB02; at Station U5; from R/V Thomas G. Thompson Cruise no. TN319; verbatimLatitude: 12'32.23; verbatimLongitude: 116'36.25; decimalLatitude: 12.53717; decimalLongitude: -116.60417; geodeticDatum: WGS84; **Identification:** identifiedBy: Helena Wiklund | Lenka Neal | Thomas Dahlgren | Adrian Glover | Madeleine Brasier | Regan Drennan | Eva Stewart; dateIdentified: 2021-04-20; identificationRemarks: identified by DNA and morphology; **Event:** eventID: UK1_AB02_EB02; samplingProtocol: Brenke Epibenthic Sledge; eventDate: 2015-02-20; eventTime: 06:24; habitat: Abyssal plain; fieldNotes: Collected from epi net (on the epibenthic sledge); **Record Level:** language: en; institutionCode: NHMUK; collectionCode: ZOO; datasetName: ABYSSLINE; basisOfRecord: PreservedSpecimen
- **Type status:** Other material. **Occurrence:** catalogNumber: NHMUK ANEA 2023.598; recordNumber: NHM_0816; recordedBy: Adrian Glover | Helena Wiklund | Thomas Dahlgren | Madeleine Brasier; individualCount: 1; preparations: specimen stored in 80% non-denatured ethanol aqueous solution | DNA voucher stored in buffer; otherCatalogNumbers: 0174126796; associatedSequences: OQ746563 (16S) | OQ738536 (COI); occurrenceID: E2E843DA-E68D-55C5-92BF-E66FC8B970B8; **Taxon:** taxonConceptID: Sipuncula sp. (NHM_715); scientificName: Sipuncula; kingdom: Animalia; phylum: Annelida; order: Sipuncula; taxonRank: family; scientificNameAuthorship: Stephen, 1965; **Location:** waterBody: Pacific; stateProvince: Clarion Clipperton Zone; locality: UK Seabed Resources Ltd exploration area UK-1 Stratum B; verbatimLocality: UK1 Stratum B; maximumDepthInMeters: 4425; locationRemarks: Deployment EB02; at Station U5; from R/V Thomas G. Thompson Cruise no. TN319; verbatimLatitude: 12'32.23; verbatimLongitude: 116'36.25; decimalLatitude: 12.53717; decimalLongitude: -116.60417; geodeticDatum: WGS84; **Identification:** identifiedBy: Helena Wiklund | Lenka Neal | Thomas Dahlgren | Adrian Glover | Madeleine Brasier | Regan Drennan | Eva Stewart; dateIdentified: 2021-04-20; identificationRemarks: identified by DNA and morphology; **Event:** eventID: UK1_AB02_EB02; samplingProtocol: Brenke Epibenthic Sledge; eventDate: 2015-02-20; eventTime: 06:24; habitat: Abyssal plain; fieldNotes: Collected from epi net (on the epibenthic sledge); **Record Level:** language: en; institutionCode: NHMUK; collectionCode: ZOO; datasetName: ABYSSLINE; basisOfRecord: PreservedSpecimen
- **Type status:** Other material. **Occurrence:** catalogNumber: NHMUK ANEA 2023.596; recordNumber: NHM_0603; recordedBy: Adrian Glover | Helena Wiklund | Thomas Dahlgren | Madeleine Brasier; individualCount: 1; preparations: specimen stored in 80% non-denatured ethanol aqueous solution | DNA voucher stored in buffer; otherCatalogNumbers: 0174126784; associatedSequences: OQ746847 (18S) | OQ738528 (COI); occurrenceID: B7B494A2-A040-5F16-84AE-356BC1E1F237; **Taxon:** taxonConceptID: Sipuncula sp. (NHM_715); scientificName: Sipuncula; kingdom: Animalia; phylum: Annelida; order: Sipuncula; taxonRank: family; scientificNameAuthorship: Stephen, 1965; **Location:** waterBody: Pacific; stateProvince: Clarion Clipperton Zone; locality: UK Seabed Resources Ltd exploration area UK-1 Stratum B; verbatimLocality: UK1 Stratum B; maximumDepthInMeters: 4202; locationRemarks: Deployment EB01; at Station U2; from R/V Thomas G. Thompson Cruise no. TN319; verbatimLatitude: 12'23.17456; verbatimLongitude: 116'32.92021; decimalLatitude: 12.38624; decimalLongitude: -116.54867; geodeticDatum: WGS84; **Identification:** identifiedBy: Helena Wiklund | Lenka Neal | Thomas Dahlgren | Adrian Glover | Madeleine Brasier | Regan Drennan | Eva Stewart; dateIdentified: 2021-04-20; identificationRemarks: identified by DNA and morphology; **Event:** eventID: UK1_AB02_EB01; samplingProtocol: Brenke Epibenthic Sledge; eventDate: 2015-02-17; eventTime: 05:15; habitat: Abyssal plain; fieldNotes: Collected from epi net (on the epibenthic sledge); **Record Level:** language: en; institutionCode: NHMUK; collectionCode: ZOO; datasetName: ABYSSLINE; basisOfRecord: PreservedSpecimen

##### Distribution

Eastern Clarion-Clipperton Zone, central Pacific Ocean.

##### Diagnosis

Specimens (Fig. 119) consistent with placement within Sipuncula, based on morphology and DNA.

#### Sipuncula sp. (NHM_1298)

B94B8C1F-2745-5263-B27B-A831C2B4B463

##### Materials

- **Type status:** Other material. **Occurrence:** catalogNumber: NHMUK ANEA 2023.593; recordNumber: NHM_1298; recordedBy: Adrian Glover | Helena Wiklund | Thomas Dahlgren | Madeleine Brasier; individualCount: 1; preparations: specimen stored in 80% non-denatured ethanol aqueous solution | DNA voucher stored in buffer; otherCatalogNumbers: 0174126786; associatedSequences: OQ746652 (16S) | OQ746886 (18S) | OQ738576 (COI); occurrenceID: A125CE6C-CD71-5D4D-8B09-EFB8A4EC6174; **Taxon:** taxonConceptID: Sipuncula sp. (NHM_1298); scientificName: Sipuncula; kingdom: Animalia; phylum: Annelida; order: Sipuncula; taxonRank: family; scientificNameAuthorship: Stephen, 1965; **Location:** waterBody: Pacific; stateProvince: Clarion Clipperton Zone; locality: Ocean Mineral Singapore exploration claim Stratum A; verbatimLocality: OMS Stratum A; maximumDepthInMeters: 4302; locationRemarks: Deployment EB06; at Station S5; from R/V Thomas G. Thompson Cruise no. TN319; verbatimLatitude: 12'15.44; verbatimLongitude: 117'18.13; decimalLatitude: 12.25733; decimalLongitude: -117.30217; geodeticDatum: WGS84; **Identification:** identifiedBy: Helena Wiklund | Lenka Neal | Thomas Dahlgren | Adrian Glover | Madeleine Brasier | Regan Drennan | Eva Stewart; dateIdentified: 2021-04-20; identificationRemarks: identified by DNA and morphology; **Event:** eventID: OMS1_AB02_EB06; samplingProtocol: Brenke Epibenthic Sledge; eventDate: 2015-03-01; eventTime: 04:02; habitat: Abyssal plain; fieldNotes: Collected from epi net (on the epibenthic sledge); **Record Level:** language: en; institutionCode: NHMUK; collectionCode: ZOO; datasetName: ABYSSLINE; basisOfRecord: PreservedSpecimen
- **Type status:** Other material. **Occurrence:** catalogNumber: NHMUK ANEA 2023.594; recordNumber: NHM_2501; recordedBy: Adrian Glover | Helena Wiklund | Thomas Dahlgren | Madeleine Brasier; individualCount: 1; preparations: specimen stored in 80% non-denatured ethanol aqueous solution | DNA voucher stored in buffer; otherCatalogNumbers: 0174126287; associatedSequences: OQ738614 (COI); occurrenceID: F314AF5F-E15E-552E-A9AA-7291DE027791; **Taxon:** taxonConceptID: Sipuncula sp. (NHM_1298); scientificName: Sipuncula; kingdom: Animalia; phylum: Annelida; order: Sipuncula; taxonRank: family; scientificNameAuthorship: Stephen, 1965; **Location:** waterBody: Pacific; stateProvince: Clarion Clipperton Zone; locality: UK Seabed Resources Ltd exploration area UK-1 Stratum B; verbatimLocality: UK1 Stratum B; maximumDepthInMeters: 4202; locationRemarks: Deployment EB01; at Station U2; from R/V Thomas G. Thompson Cruise no. TN319; verbatimLatitude: 12'23.17456; verbatimLongitude: 116'32.92021; decimalLatitude: 12.38624; decimalLongitude: -116.54867; geodeticDatum: WGS84; **Identification:** identifiedBy: Helena Wiklund | Lenka Neal | Thomas Dahlgren | Adrian Glover | Madeleine Brasier | Regan Drennan | Eva Stewart; dateIdentified: 2021-04-20; identificationRemarks: identified by DNA and morphology; **Event:** eventID: UK1_AB02_EB01; samplingProtocol: Brenke Epibenthic Sledge; eventDate: 2015-02-17; eventTime: 05:15; habitat: Abyssal plain; fieldNotes: Collected from supra net (on the epibenthic sledge); **Record Level:** language: en; institutionCode: NHMUK; collectionCode: ZOO; datasetName: ABYSSLINE; basisOfRecord: PreservedSpecimen

##### Distribution

Eastern Clarion-Clipperton Zone, central Pacific Ocean.

##### Diagnosis

**Figure 120.**
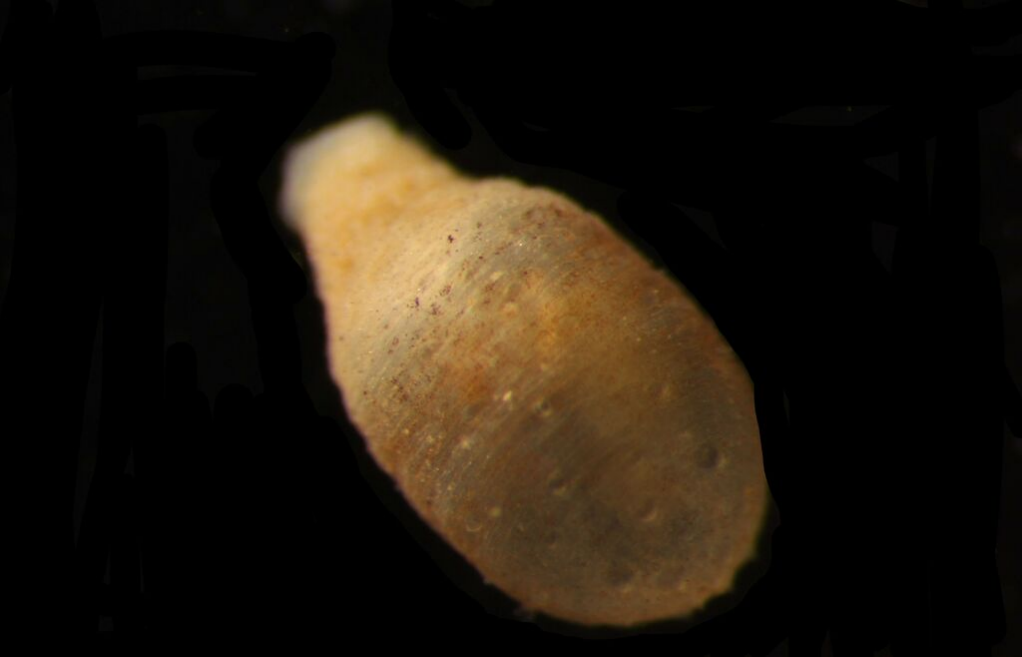
Sipuncula sp. (NHM_1298), live specimen NHM_1298.

Specimen (Fig. 120) consistent with placement within Sipuncula, based on morphology and DNA.

### Sphaerodoridae Malmgren, 1867

#### Sphaerodoridae sp. (NHM_1003)

102DAB64-E772-57E9-A32C-DAB9D446322B

##### Materials

- **Type status:** Other material. **Occurrence:** catalogNumber: NHMUK ANEA 2023.581; recordNumber: NHM_1003; recordedBy: Adrian Glover | Helena Wiklund | Thomas Dahlgren | Madeleine Brasier; individualCount: 1; preparations: specimen stored in 80% non-denatured ethanol aqueous solution | DNA voucher stored in buffer; otherCatalogNumbers: 0174126751; associatedSequences: OQ746870 (18S); occurrenceID: EF51AD2B-AD14-5733-BD71-4BC011307B01; **Taxon:** taxonConceptID: Sphaerodoridae sp. (NHM_1003); scientificName: Sphaerodoridae; kingdom: Animalia; phylum: Annelida; class: Polychaeta; order: Phyllodocida; family: Sphaerodoridae; taxonRank: family; scientificNameAuthorship: Malmgren, 1867; **Location:** waterBody: Pacific; stateProvince: Clarion Clipperton Zone; locality: Ocean Mineral Singapore exploration claim Stratum A; verbatimLocality: OMS Stratum A; maximumDepthInMeters: 4122; locationRemarks: Deployment EB04; at Station S1; from R/V Thomas G. Thompson Cruise no. TN319; verbatimLatitude: 12'08.02; verbatimLongitude: 117'17.52; decimalLatitude: 12.13367; decimalLongitude: -117.292; geodeticDatum: WGS84; **Identification:** identifiedBy: Helena Wiklund | Lenka Neal | Thomas Dahlgren | Adrian Glover | Madeleine Brasier | Regan Drennan | Eva Stewart; dateIdentified: 2021-04-20; identificationRemarks: identified by DNA and morphology; **Event:** eventID: OMS1_AB02_EB04; samplingProtocol: Brenke Epibenthic Sledge; eventDate: 2015-02-24; eventTime: 19:10; habitat: Abyssal plain; fieldNotes: Collected from epi net (on the epibenthic sledge); **Record Level:** language: en; institutionCode: NHMUK; collectionCode: ZOO; datasetName: ABYSSLINE; basisOfRecord: PreservedSpecimen

##### Distribution

Eastern Clarion-Clipperton Zone, central Pacific Ocean.

##### Diagnosis

**Figure 121.**
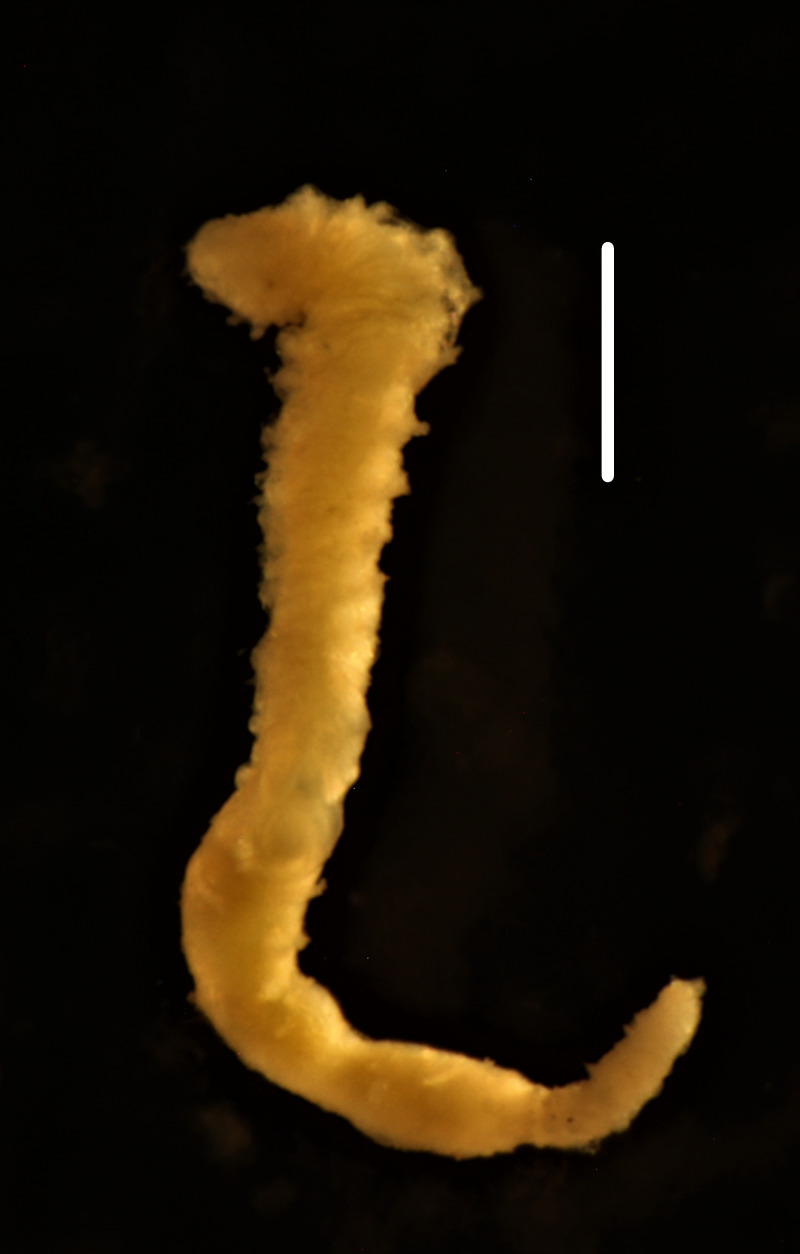
Sphaerodoridae sp. (NHM_1003), complete preserved specimen NHM_1003. Scale bar 1 mm.

Damaged specimen (Fig. 121) consistent with placement within family Sphaerodoridae, based on morphology and DNA.

#### Sphaerodoridae sp. (NHM_2177)

C5B3AA40-C8E5-526C-B34E-BB5111433314

##### Materials

- **Type status:** Other material. **Occurrence:** catalogNumber: NHMUK ANEA 2023.582; recordNumber: NHM_2177; recordedBy: Adrian Glover | Helena Wiklund | Thomas Dahlgren | Madeleine Brasier; individualCount: 1; preparations: specimen stored in 80% non-denatured ethanol aqueous solution | DNA voucher stored in buffer; otherCatalogNumbers: 0174126232; associatedSequences: OQ746912 (18S); occurrenceID: 606B9581-C249-5E0D-8616-121B842BA00F; **Taxon:** taxonConceptID: Sphaerodoridae sp. (NHM_2177); scientificName: Sphaerodoridae; kingdom: Animalia; phylum: Annelida; class: Polychaeta; order: Phyllodocida; family: Sphaerodoridae; taxonRank: family; scientificNameAuthorship: Malmgren, 1867; **Location:** waterBody: Pacific; stateProvince: Clarion Clipperton Zone; locality: Area of Particular Interest APEI-6; verbatimLocality: APEI-6; maximumDepthInMeters: 4141; locationRemarks: Deployment BC28; at Station A01; from R/V Thomas G. Thompson Cruise no. TN319; verbatimLatitude: 19 27.998; verbatimLongitude: 120 00.172; decimalLatitude: 19.46663; decimalLongitude: 120.00287; geodeticDatum: WGS84; **Identification:** identifiedBy: Helena Wiklund | Lenka Neal | Thomas Dahlgren | Adrian Glover | Madeleine Brasier | Regan Drennan | Eva Stewart; dateIdentified: 2021-04-20; identificationRemarks: identified by DNA and morphology; **Event:** eventID: APEI6_AB02_BC28; samplingProtocol: USNEL Box Core; eventDate: 2015-03-21; eventTime: 19:50; habitat: Abyssal plain; fieldNotes: Collected from 0-2 cm layer of box core using a 300 micron sieve; **Record Level:** language: en; institutionCode: NHMUK; collectionCode: ZOO; datasetName: ABYSSLINE; basisOfRecord: PreservedSpecimen

##### Distribution

Eastern Clarion-Clipperton Zone, central Pacific Ocean.

##### Diagnosis

**Figure 122.**
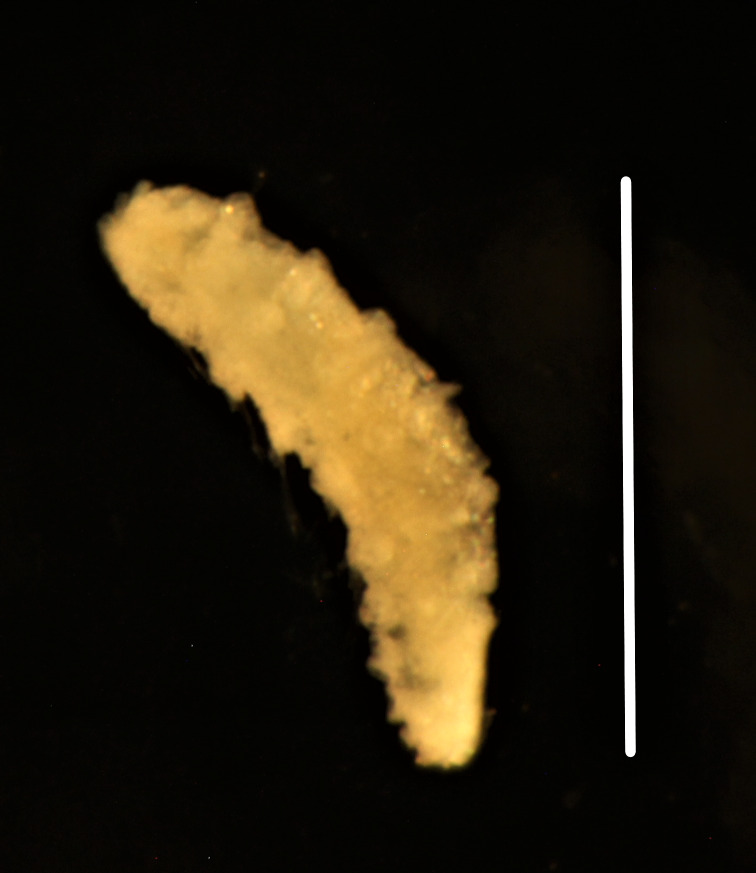
Sphaerodoridae sp. (NHM_2177), preserved specimen NHM_2177 in dorsal view. Scale bar 1 mm.

Damaged specimen (Fig. 122) consistent with placement within family Sphaerodoridae, based on morphology and DNA.

#### Sphaerodoridae sp. (NHM_2532)

BEED596F-65A4-5C1C-9CF1-4F9AB2B06D3A

##### Materials

- **Type status:** Other material. **Occurrence:** catalogNumber: NHMUK ANEA 2023.583; recordNumber: NHM_2532; recordedBy: Adrian Glover | Helena Wiklund | Thomas Dahlgren | Madeleine Brasier; individualCount: 1; preparations: specimen stored in 80% non-denatured ethanol aqueous solution | DNA voucher stored in buffer; otherCatalogNumbers: 0174126200; associatedSequences: OQ746778 (16S) | OQ738615 (COI); occurrenceID: 1AB54D75-1F53-55E0-A803-1B66FE2B70E1; **Taxon:** taxonConceptID: Sphaerodoridae sp. (NHM_2532); scientificName: Sphaerodoridae; kingdom: Animalia; phylum: Annelida; class: Polychaeta; order: Phyllodocida; family: Sphaerodoridae; taxonRank: family; scientificNameAuthorship: Malmgren, 1867; **Location:** waterBody: Pacific; stateProvince: Clarion Clipperton Zone; locality: UK Seabed Resources Ltd exploration area UK-1 Stratum B; verbatimLocality: UK1 Stratum B; maximumDepthInMeters: 4137; locationRemarks: Deployment EB07; at Station U7; from R/V Thomas G. Thompson Cruise no. TN319; verbatimLatitude: 12'27.26; verbatimLongitude: 116'36.77; decimalLatitude: 12.45433; decimalLongitude: -116.61283; geodeticDatum: WGS84; **Identification:** identifiedBy: Helena Wiklund | Lenka Neal | Thomas Dahlgren | Adrian Glover | Madeleine Brasier | Regan Drennan | Eva Stewart; dateIdentified: 2021-04-20; identificationRemarks: identified by DNA and morphology; **Event:** eventID: UK1_AB02_EB07; samplingProtocol: Brenke Epibenthic Sledge; eventDate: 2015-03-03; eventTime: 20:40; habitat: Abyssal plain; fieldNotes: Collected from supra net (on the epibenthic sledge); **Record Level:** language: en; institutionCode: NHMUK; collectionCode: ZOO; datasetName: ABYSSLINE; basisOfRecord: PreservedSpecimen

##### Distribution

Eastern Clarion-Clipperton Zone, central Pacific Ocean.

##### Diagnosis

**Figure 123.**
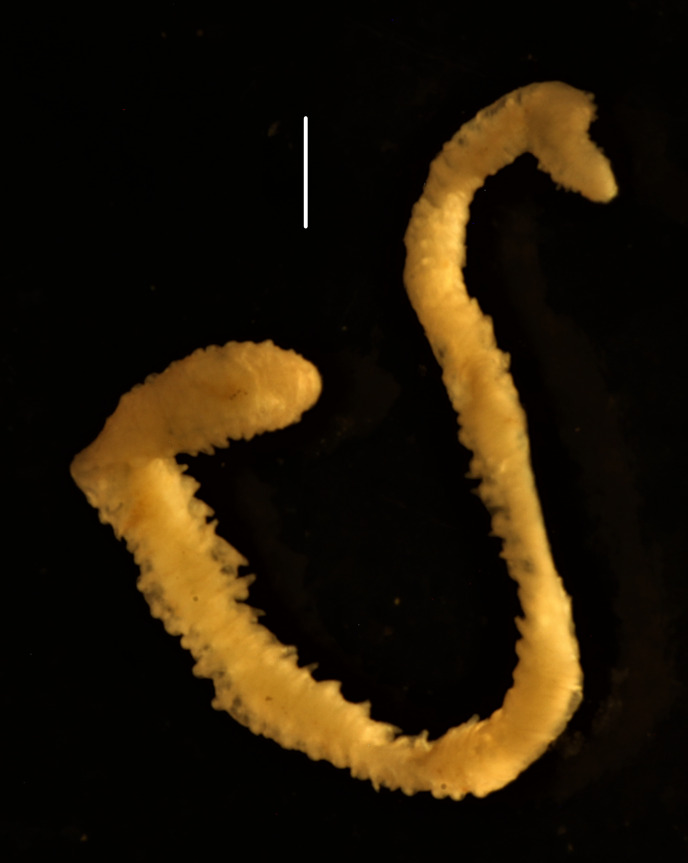
Sphaerodoridae sp. (NHM_2532), complete preserved specimen NHM_2532. Scale bar 1 mm.

Damaged specimen (Fig. 123) consistent with placement within family Sphaerodoridae, based on morphology and DNA.

### Terebellidae Johnston, 1846

#### Terebellidae sp. (NHM_018)

2DBAD3B7-185D-5DA4-A959-4D15F6D09042

##### Materials

- **Type status:** Other material. **Occurrence:** catalogNumber: NHMUK ANEA 2023.584; recordNumber: NHM_0018; recordedBy: Adrian Glover | Helena Wiklund | Thomas Dahlgren | Magdalena Georgieva; individualCount: 1; preparations: specimen stored in 80% non-denatured ethanol aqueous solution | DNA voucher stored in buffer; otherCatalogNumbers: 0174127321; associatedSequences: OQ746467 (16S) | OQ746790 (18S); occurrenceID: A45B54E5-1A80-5AD6-9D20-561594EB62CB; **Taxon:** taxonConceptID: Terebellidae sp. (NHM_018); scientificName: Terebellidae; kingdom: Animalia; phylum: Annelida; class: Polychaeta; order: Terebellida; family: Terebellidae; taxonRank: family; scientificNameAuthorship: Johnston, 1846; **Location:** waterBody: Pacific; stateProvince: Clarion Clipperton Zone; locality: UK Seabed Resources Ltd exploration area UK-1 Stratum A; verbatimLocality: UK1 Stratum A; maximumDepthInMeters: 4336; locationRemarks: Deployment EB01; at Station B-K-E; from R/V Melville Cruise no. MV1313; verbatimLatitude: 13°50.232; verbatimLongitude: 116°33.506; decimalLatitude: 13.8372; decimalLongitude: -116.55843; geodeticDatum: WGS84; **Identification:** identifiedBy: Helena Wiklund | Lenka Neal | Thomas Dahlgren | Adrian Glover | Madeleine Brasier | Regan Drennan | Eva Stewart; dateIdentified: 2021-04-20; identificationRemarks: identified by DNA and morphology; **Event:** eventID: UK1_AB01_EB01; samplingProtocol: Brenke Epibenthic Sledge; eventDate: 2013-10-09; eventTime: 10:26; habitat: Abyssal plain; fieldNotes: Collected from epi net (on the epibenthic sledge); **Record Level:** language: en; institutionCode: NHMUK; collectionCode: ZOO; datasetName: ABYSSLINE; basisOfRecord: PreservedSpecimen

##### Distribution

Eastern Clarion-Clipperton Zone, central Pacific Ocean.

##### Diagnosis

**Figure 124.**
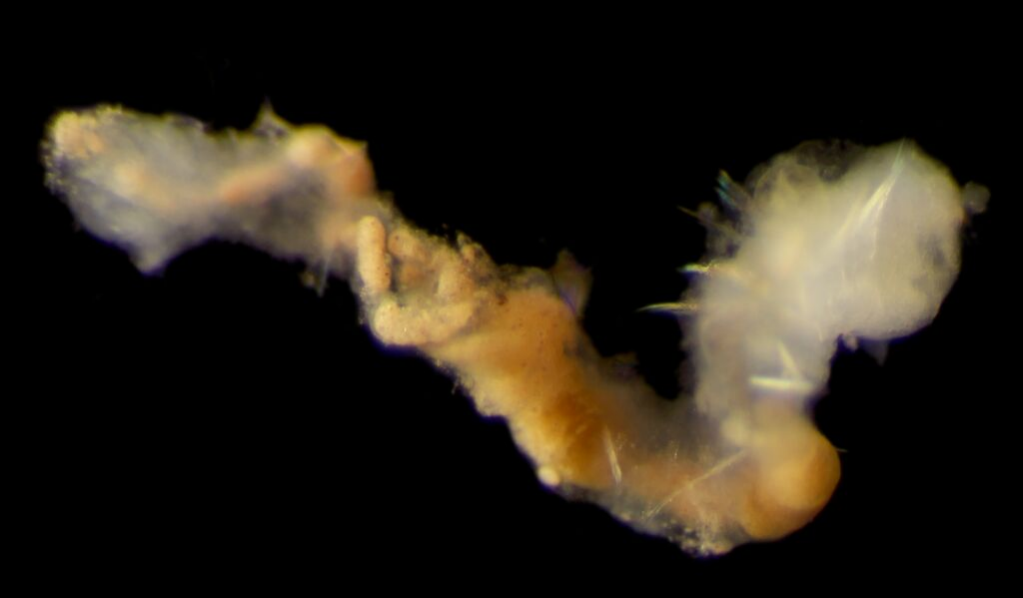
Terebellidae sp. (NHM_018), damaged live specimen NHM_018 in dorsolateral view.

Damaged specimen (Fig. 124) consistent with placement within family Terebellidae, based on morphology and DNA.

### Trichobranchidae Malmgren, 1866

#### Terebellides sp. (NHM_167)

C15E0FB7-85F4-5D1D-9D43-9E37332EC57B

##### Materials

- **Type status:** Other material. **Occurrence:** catalogNumber: NHMUK ANEA 2023.585; recordNumber: NHM_0167; recordedBy: Adrian Glover | Helena Wiklund | Thomas Dahlgren | Magdalena Georgieva; individualCount: 1; preparations: specimen stored in 80% non-denatured ethanol aqueous solution | DNA voucher stored in buffer; otherCatalogNumbers: 0174127348; associatedSequences: OQ746482 (16S); occurrenceID: 05E83A05-F47E-53C6-9074-5A3DDB7594F5; **Taxon:** taxonConceptID: Terebellides sp. (NHM_167); scientificName: Terebellides; kingdom: Animalia; phylum: Annelida; class: Polychaeta; order: Terebellida; family: Trichobranchidae; genus: Terebellides; taxonRank: genus; scientificNameAuthorship: Sars, 1835; **Location:** waterBody: Pacific; stateProvince: Clarion Clipperton Zone; locality: UK Seabed Resources Ltd exploration area UK-1 Stratum A; verbatimLocality: UK1 Stratum A; maximumDepthInMeters: 4084; locationRemarks: Deployment BC06; at Station D; from R/V Melville Cruise no. MV1313; verbatimLatitude: 13°57.794; verbatimLongitude: 116°34.093; decimalLatitude: 13.96323; decimalLongitude: -116.56822; geodeticDatum: WGS84; **Identification:** identifiedBy: Helena Wiklund | Lenka Neal | Thomas Dahlgren | Adrian Glover | Madeleine Brasier | Regan Drennan | Eva Stewart; dateIdentified: 2021-04-20; identificationRemarks: identified by DNA and morphology; **Event:** eventID: UK1_AB01_BC06; samplingProtocol: USNEL Box Core; eventDate: 2013-10-12; eventTime: 23:01:00; habitat: Abyssal plain; fieldNotes: Collected from 0-2 cm layer of box core using a 300 micron sieve; **Record Level:** language: en; institutionCode: NHMUK; collectionCode: ZOO; datasetName: ABYSSLINE; basisOfRecord: PreservedSpecimen

##### Distribution

Eastern Clarion-Clipperton Zone, central Pacific Ocean.

##### Diagnosis

Damaged specimen (Fig. 125) consistent with placement within genus *Terebellides*, based on morphology and DNA.

#### Terebellides sp. (NHM_406)

86F129AD-53E8-5755-9CC4-6AEA46360C37

##### Materials

- **Type status:** Other material. **Occurrence:** catalogNumber: NHMUK ANEA 2023.586; recordNumber: NHM_0406; recordedBy: Adrian Glover | Helena Wiklund | Thomas Dahlgren | Magdalena Georgieva; individualCount: 1; preparations: specimen stored in 80% non-denatured ethanol aqueous solution | DNA voucher stored in buffer; otherCatalogNumbers: 0174127322; associatedSequences: OQ746509 (16S) | OQ746826 (18S) | OQ738517 (COI); occurrenceID: B277B1D4-E96D-58C4-AADE-DB50AA58011A; **Taxon:** taxonConceptID: Terebellides sp. (NHM_406); scientificName: Terebellides; kingdom: Animalia; phylum: Annelida; class: Polychaeta; order: Terebellida; family: Trichobranchidae; genus: Terebellides; taxonRank: genus; scientificNameAuthorship: Sars, 1835; **Location:** waterBody: Pacific; stateProvince: Clarion Clipperton Zone; locality: UK Seabed Resources Ltd exploration area UK-1 Stratum A; verbatimLocality: UK1 Stratum A; maximumDepthInMeters: 4500; locationRemarks: Deployment BC12; at Station K; from R/V Melville Cruise no. MV1313; decimalLatitude: 13.86328; decimalLongitude: -116.54885; geodeticDatum: WGS84; **Identification:** identifiedBy: Helena Wiklund | Lenka Neal | Thomas Dahlgren | Adrian Glover | Madeleine Brasier | Regan Drennan | Eva Stewart; dateIdentified: 2021-04-20; identificationRemarks: identified by DNA and morphology; **Event:** eventID: UK1_AB01_BC12; samplingProtocol: USNEL Box Core; eventDate: 2013-10-20; eventTime: 03:39; habitat: Abyssal plain; fieldNotes: Collected from 0-2 cm layer of box core using a 300 micron sieve; **Record Level:** language: en; institutionCode: NHMUK; collectionCode: ZOO; datasetName: ABYSSLINE; basisOfRecord: PreservedSpecimen

##### Distribution

Eastern Clarion-Clipperton Zone, central Pacific Ocean.

##### Diagnosis

**Figure 126.**
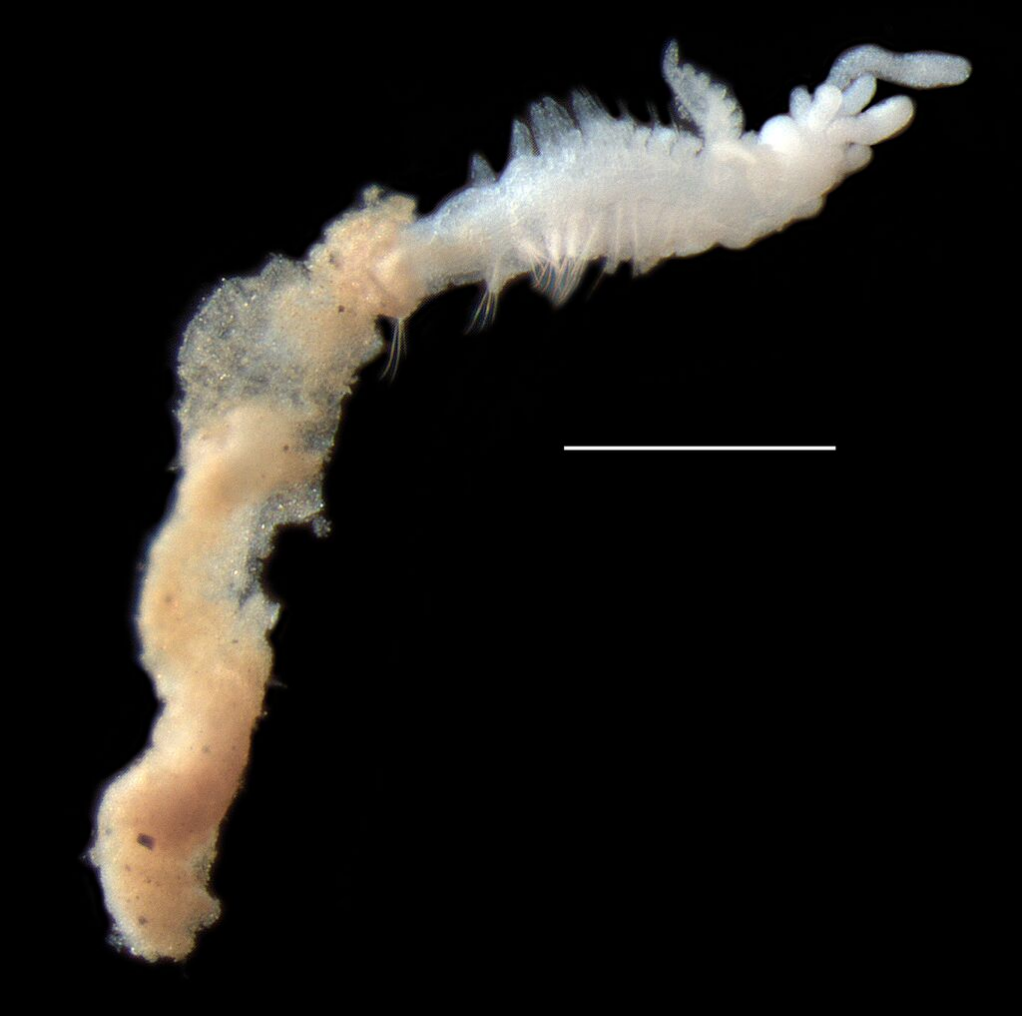
*Terebellides* sp. (NHM_406), live specimen NHM_406 in lateral view. Scale bar 500 μm.

Damaged specimen (Fig. 126) consistent with placement within genus *Terebellides*, based on morphology and DNA.

## Discussion

Species checklists are critical tools in biodiversity and conservation that can be used to underpin our understanding of biological change and appropriate policy measures (Reyserhove et al. 2020, Rabone et al. 2023b). However, checklists are of limited value if the data are not freely available. Therefore, the emphasis of this publication is on open data following the FAIR principles (Wilkinson et al. 2016). Recent years have seen an increased emphasis on FAIR data, resulting in a resurgence in the public availability of checklists (Rabone et al. 2023a, Rabone et al. 2023b), also exemplified by the success of the World Register of Marine Species.

More often than not, ecological papers describing biodiversity do not include a list of all the species and specimens used to make the broader ecological inferences and even more rarely make the specimens and all associated metadata available in a FAIR way (Bianchi and Gonçalves 2021). In this study, we have made a significant and time-consuming attempt to do this, in a region of the global oceans where critical policy decisions are being made that could impact the way humanity obtains its resources and manages its environment in a sustainable way.

## Supplementary Material

DAAB5E9D-EE6F-5B62-9359-80D5CD8B76E4 10.3897/BDJ.11.e86921.suppl1 Supplementary material 1 Genetic Matches from GenBank Data type genetic Brief description Table of COI genetic matches obtained by BLAST search from GenBank. Only matches > 98% were considered. File: oo_851458.xlsx https://binary.pensoft.net/file/851458 Lenka Neal, Lucas King

5728CF70-111E-5C19-8EE5-A5008AB6953C 10.3897/BDJ.11.e86921.suppl2 Supplementary material 2 Combined Darwin Core from taxon treatments in checklist Data type occurrences Brief description Table of all Darwin Core taxon treatments included in the checklist herein. File: oo_852817.csv https://binary.pensoft.net/file/852817 Muriel Rabone

## Figures and Tables

**Figure 2a.**
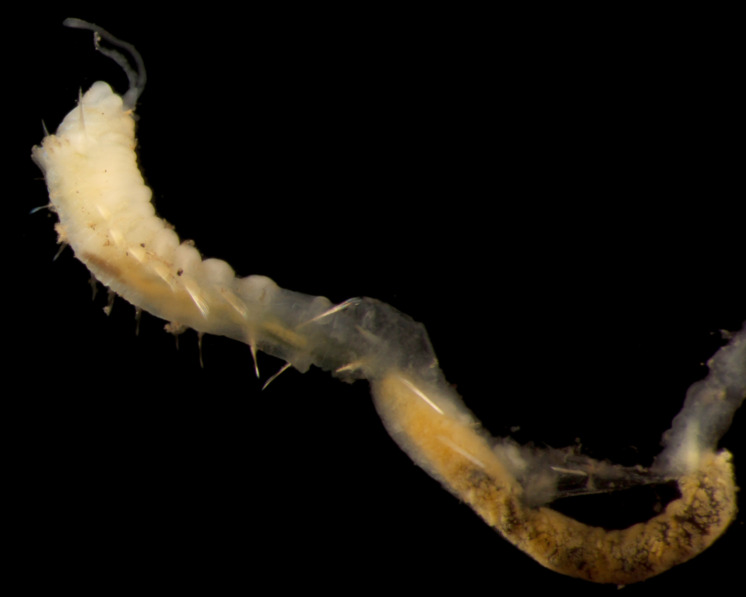
Anterior fragment of live specimen NHM_1893;

**Figure 2b.**
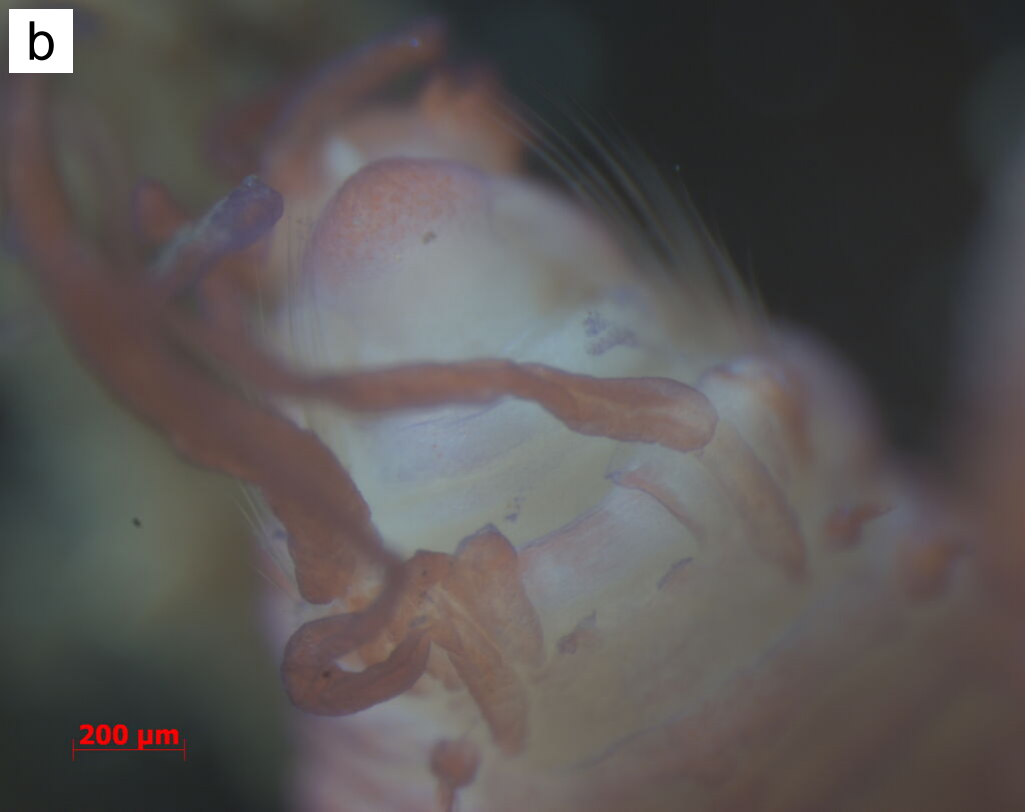
Dorsal view of prostomium with branchiae, preserved specimen NHM_015;

**Figure 2c.**
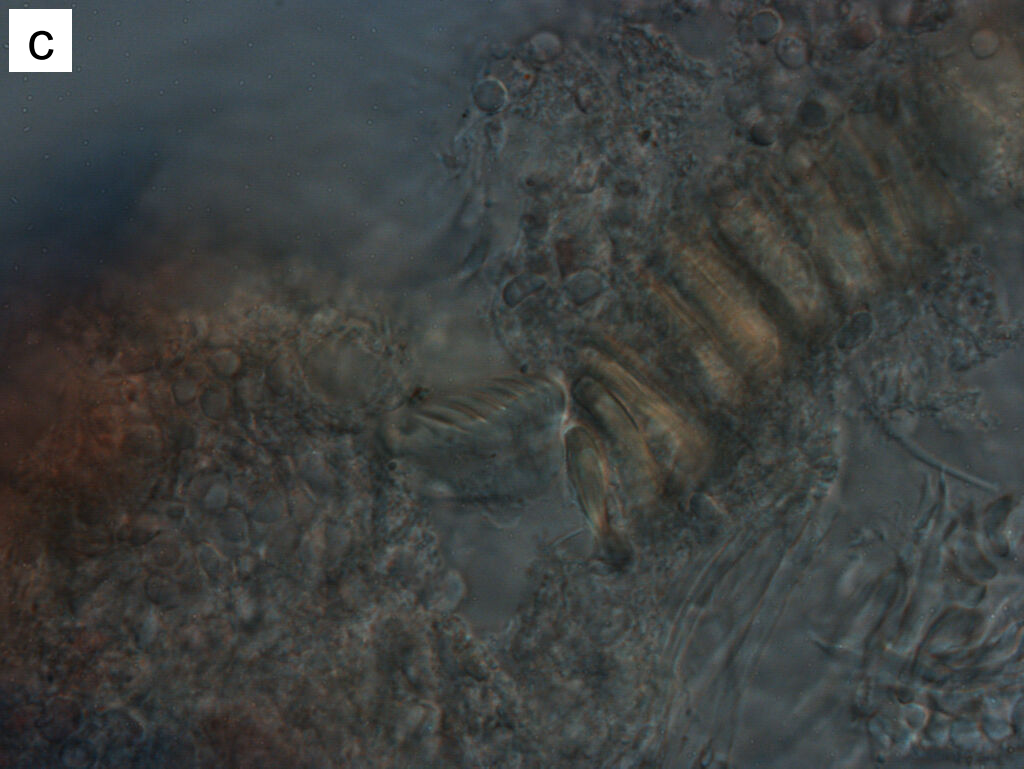
Thoracic uncini, specimen NHM_015;

**Figure 2d.**
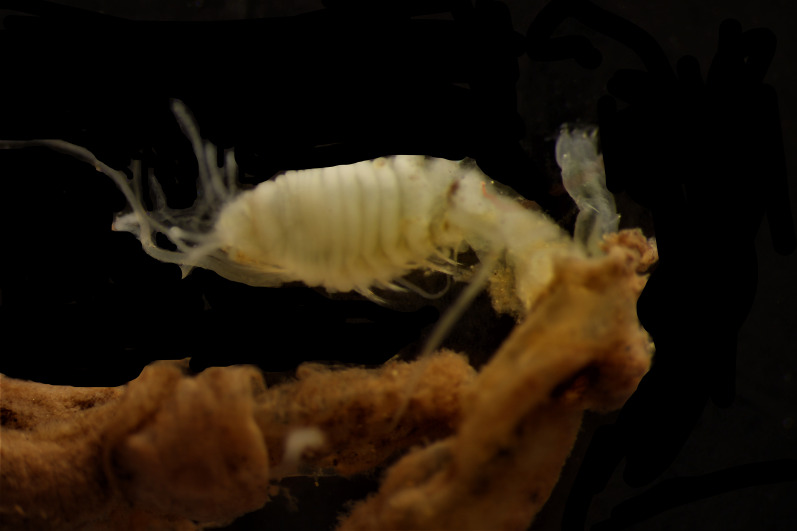
Live specimen NHM_015 in ventral view, partly inside the tube.

**Figure 3a.**
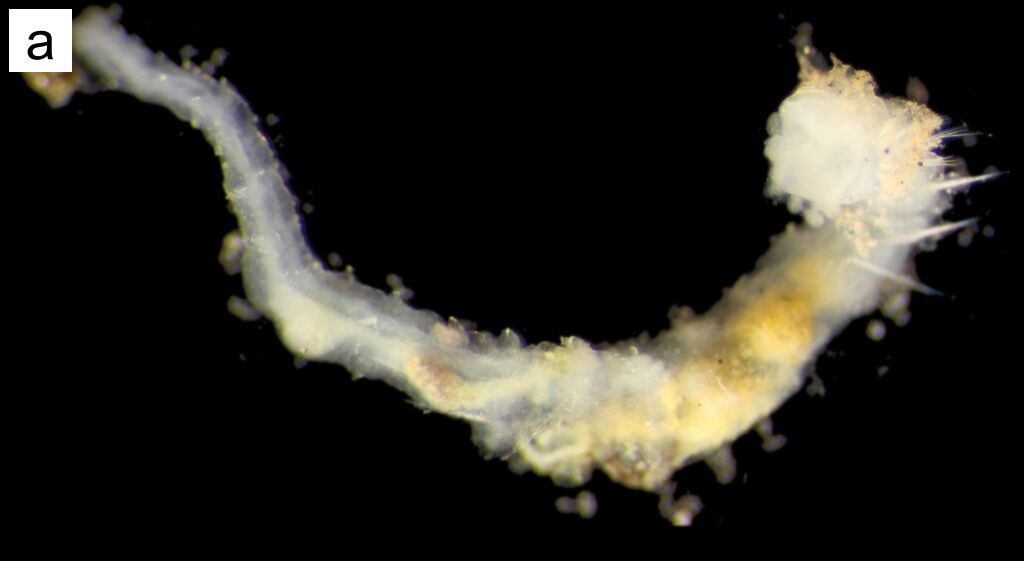
Specimen NHM_044, overview;

**Figure 3b.**
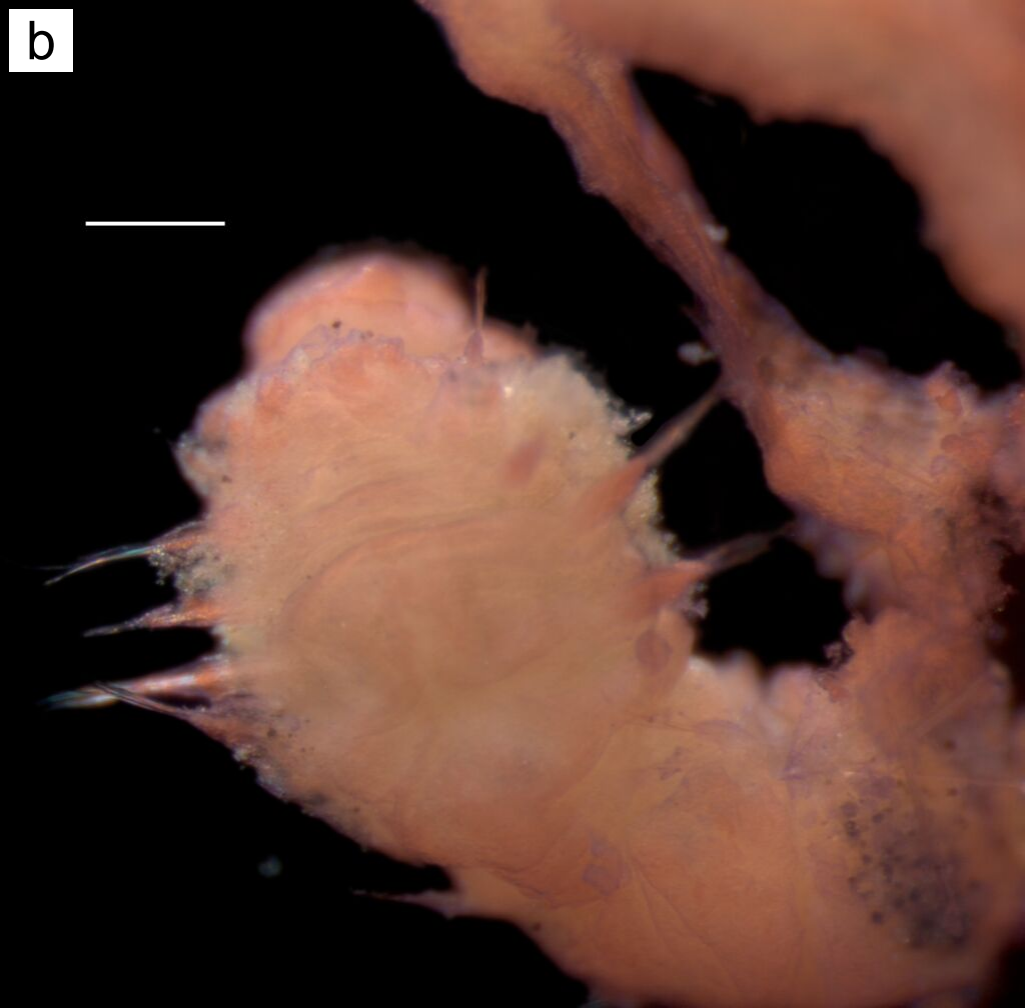
Specimen NHM_044, dorsal view of anterior end, scale bar 200 µm.

**Figure 18a.**
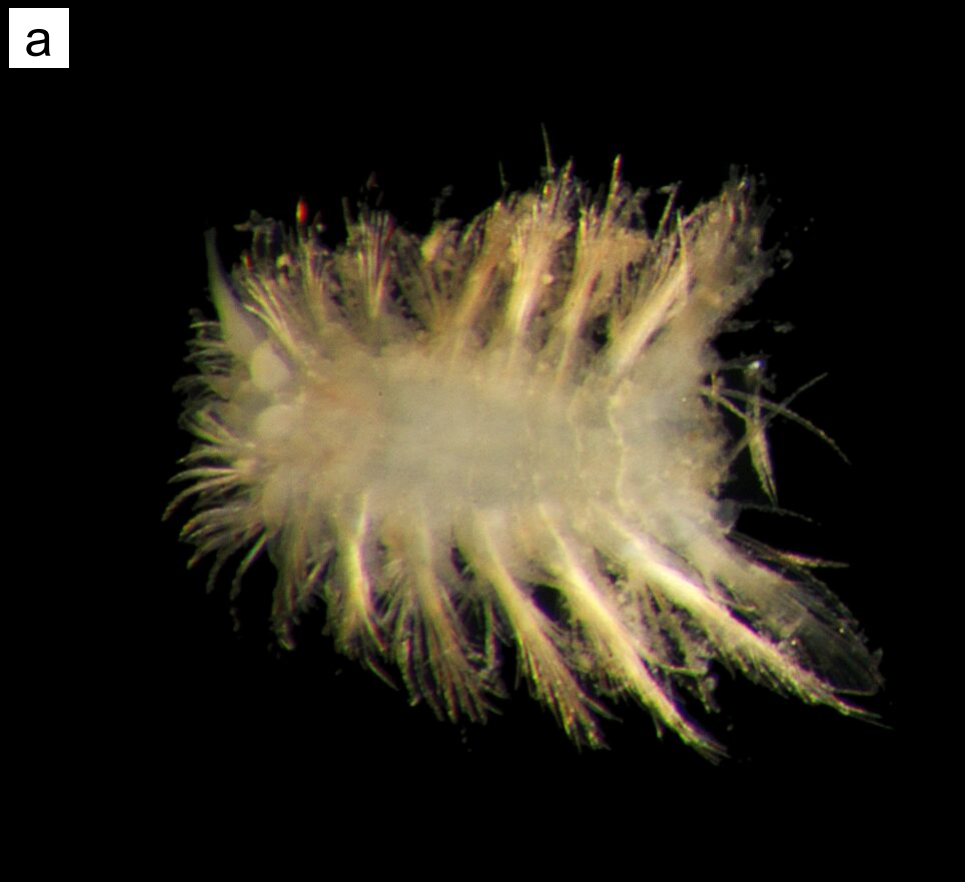
Anterior fragment of live specimen NHM_1303 in ventral view;

**Figure 18b.**
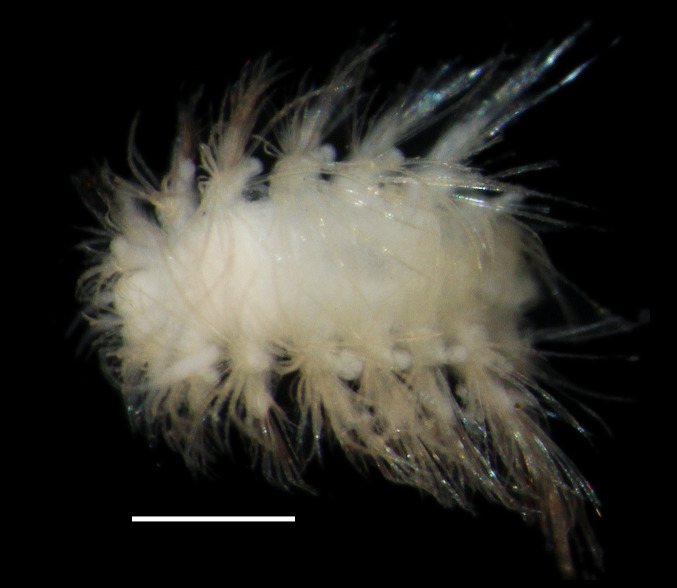
Anterior fragment of preserved specimen NHM_1303 in dorsal view, scale bar 0.5 mm.

**Figure 43a.**
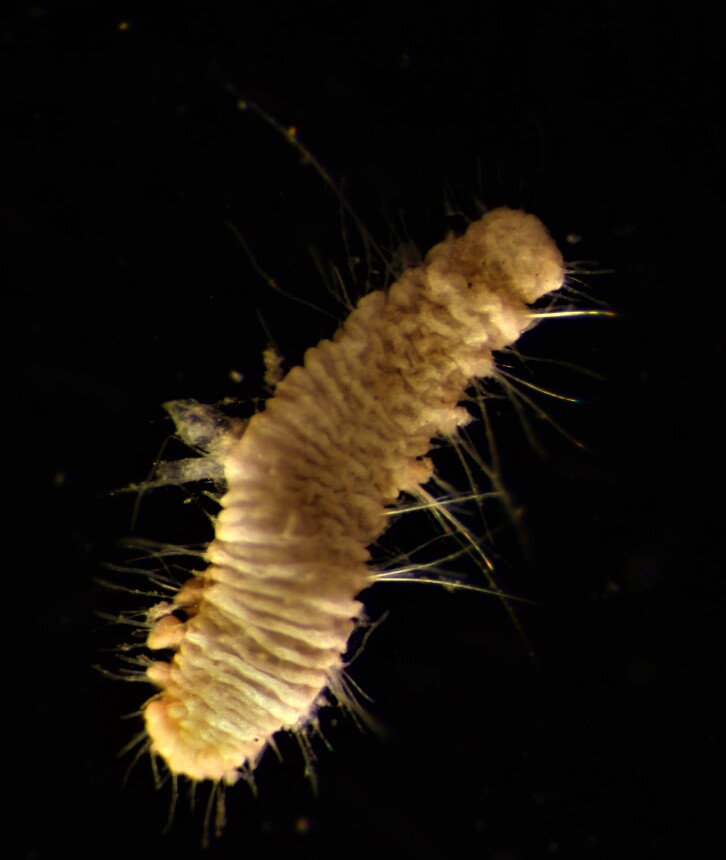
Fragment of a live specimen NHM_045;

**Figure 43b.**
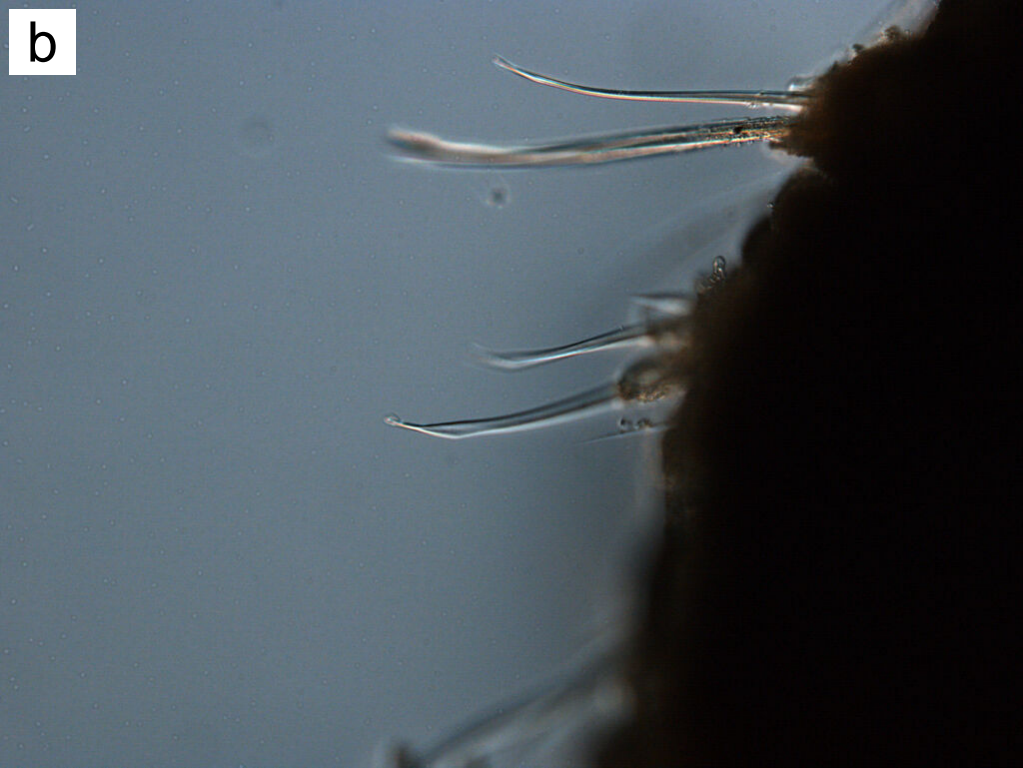
Example of chaetae from specimen NHM_045.

**Figure 58a.**
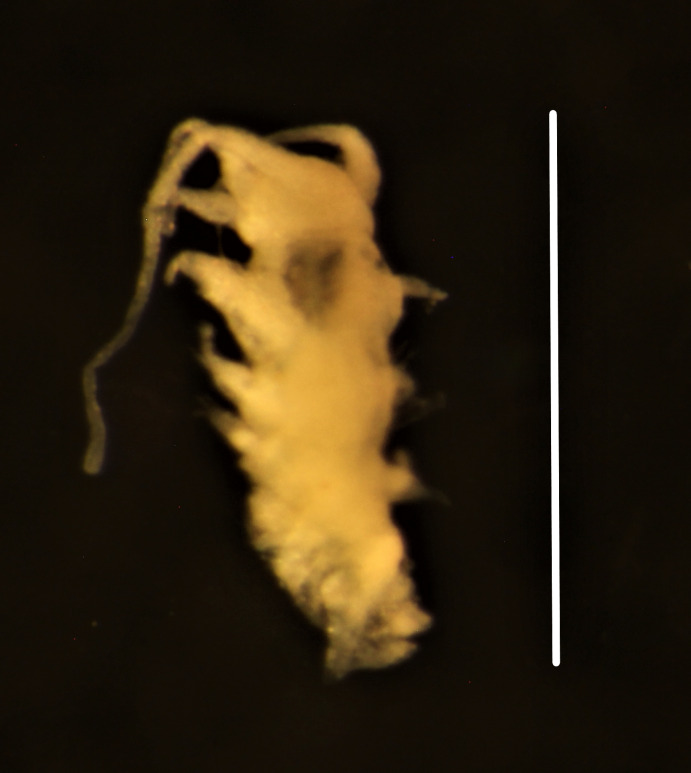
Anterior fragment of specimen NHM_2430 in dorsal view. Scale bar 1 mm;

**Figure 58b.**
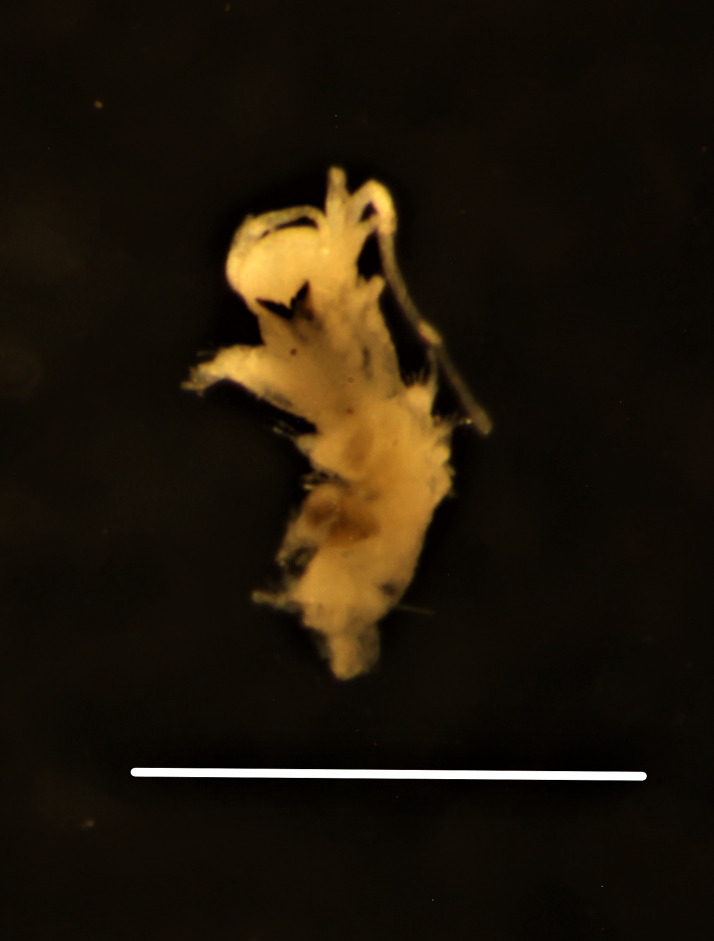
Anterior fragment of specimen NHM_2430 in ventral view. Scale bar 1 mm.

**Figure 62a.**
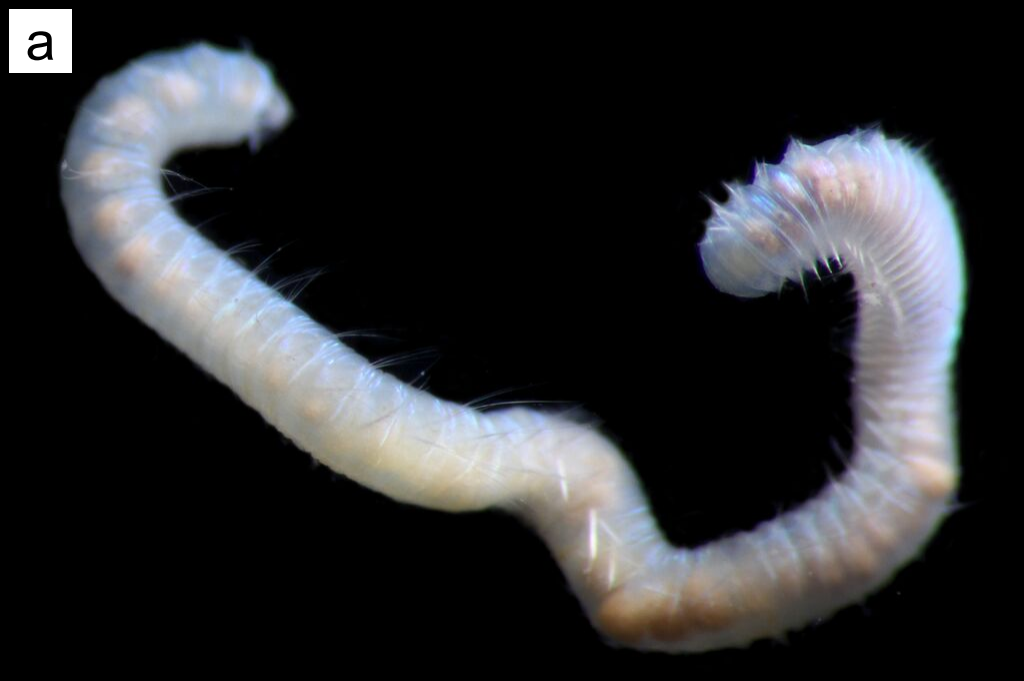
Fragment of live specimen NHM_754;

**Figure 62b.**
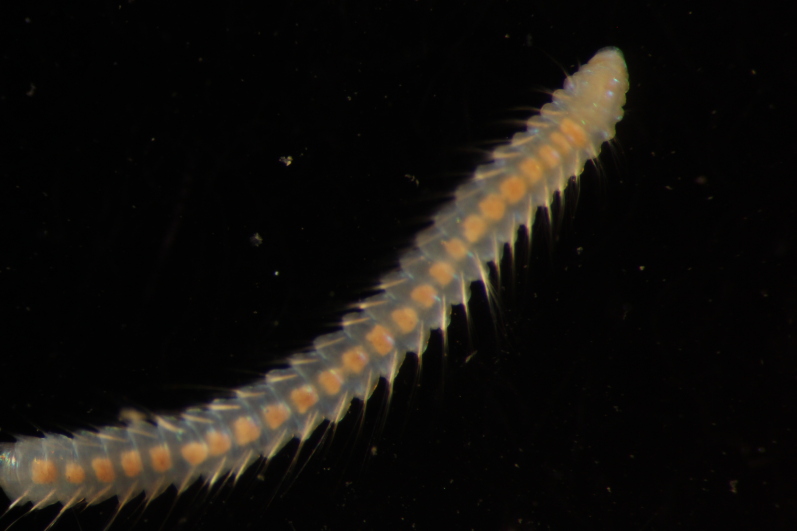
Live specimen NHM_1071.

**Figure 73a.**
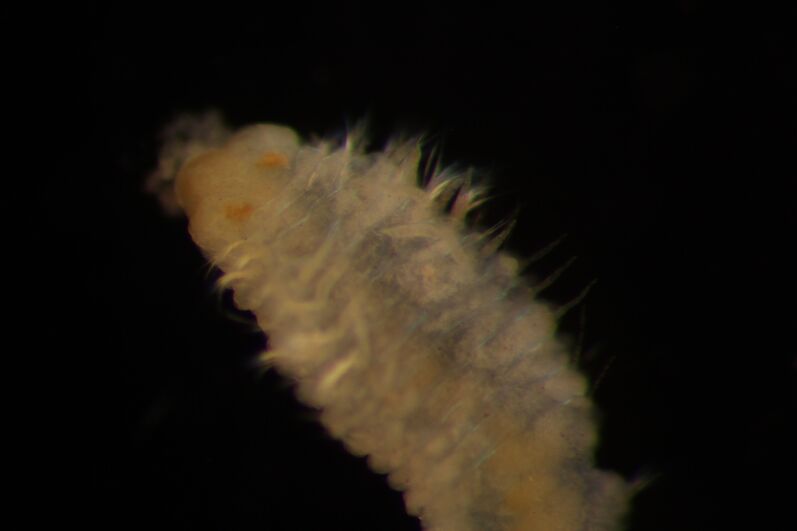
Anterior fragment of live specimen NHM_1452 in dorsal view;

**Figure 73b.**
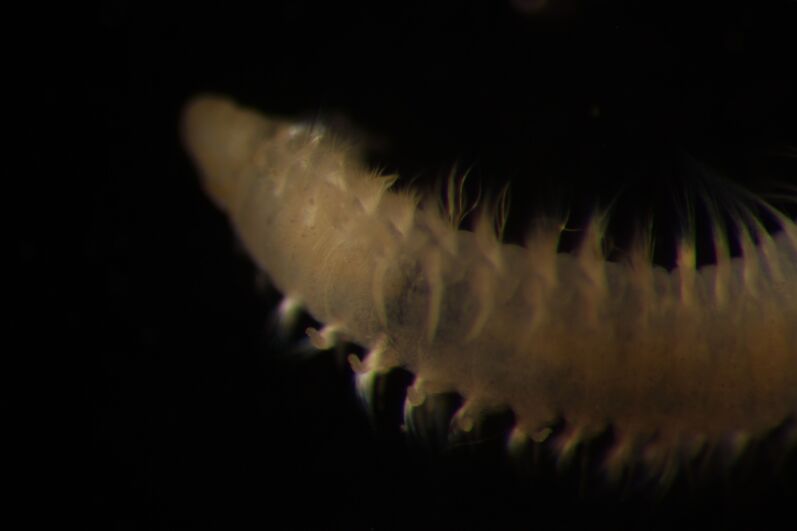
Anterior fragment of live specimen NHM_1774 in dorsolateral view.

**Figure 77a.**
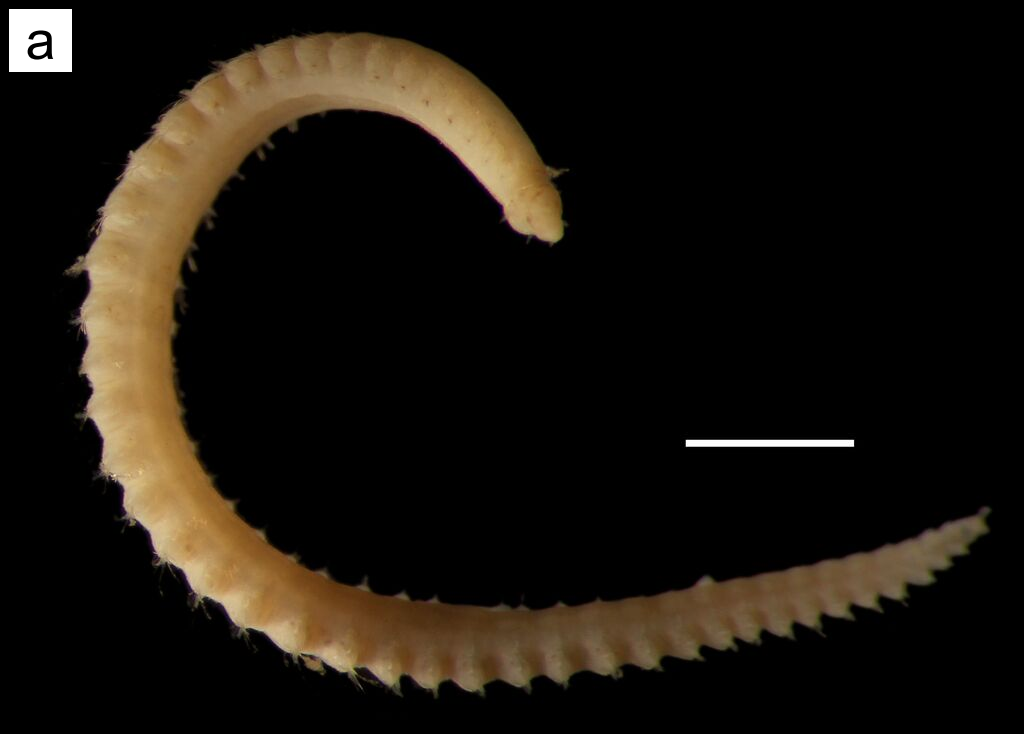
Complete specimen NHM_1385 in ventrolateral view. Scale bar 1 mm;

**Figure 77b.**
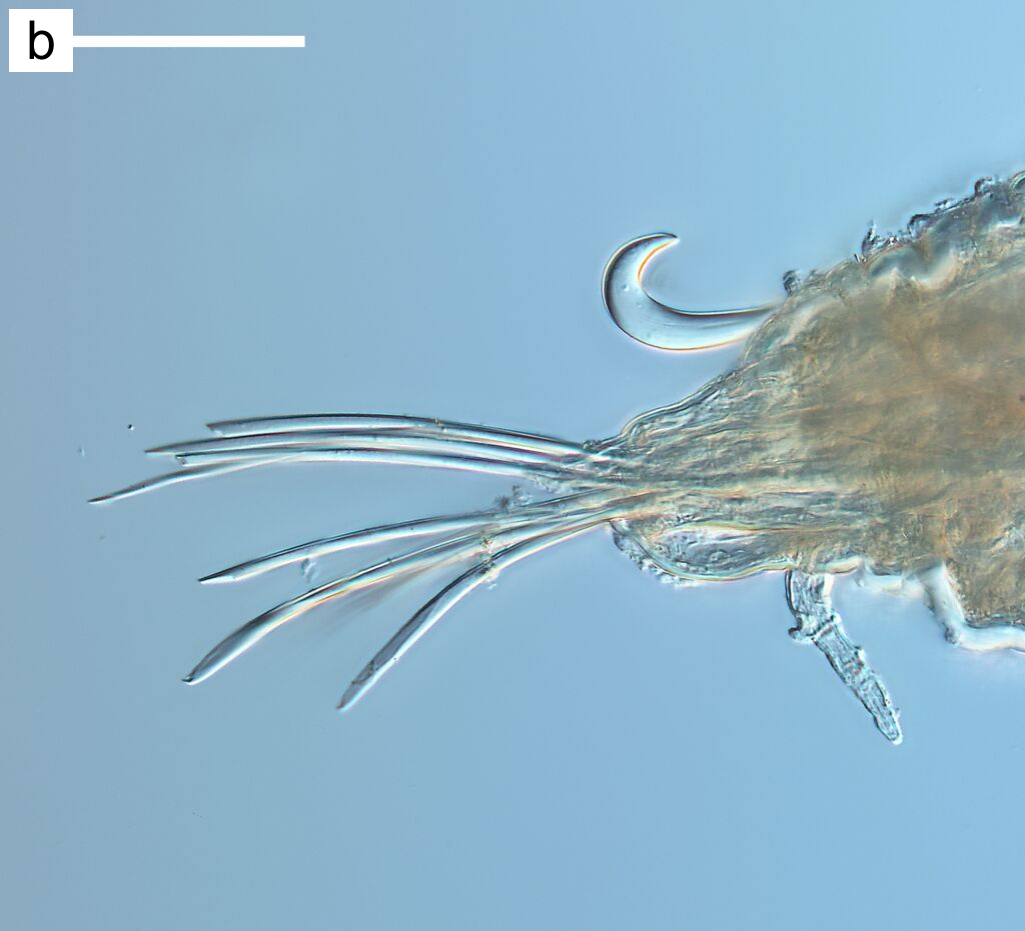
Specimen NHM_1424, detail of mid-body parapodium. Scale bar 100 μm.

**Figure 80a.**
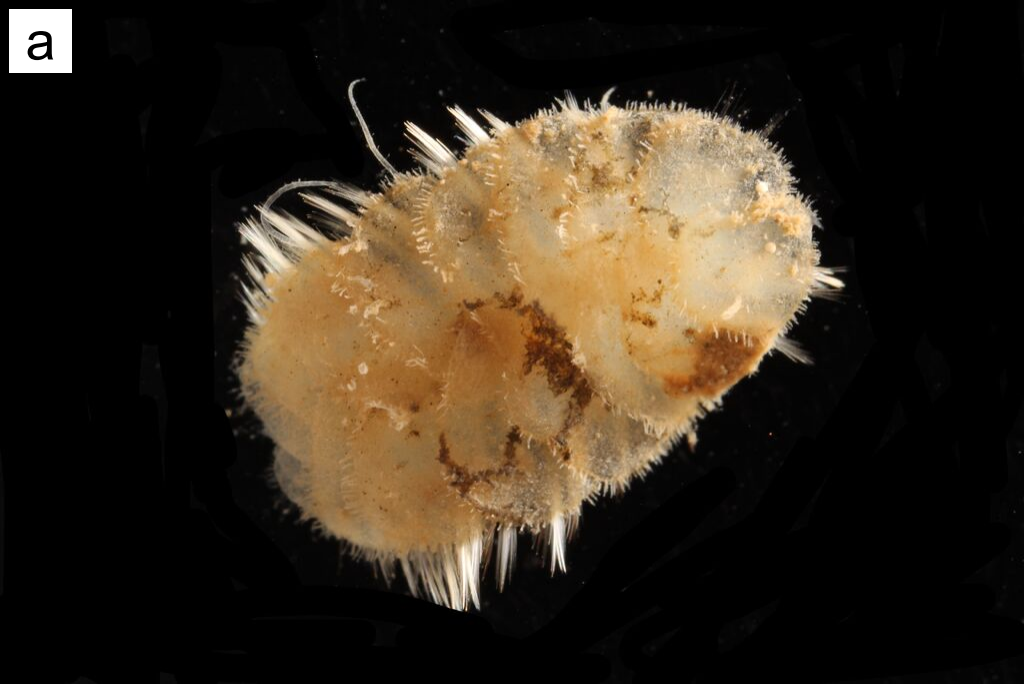
Complete live specimen NHM_235 in dorsal view;

**Figure 80b.**
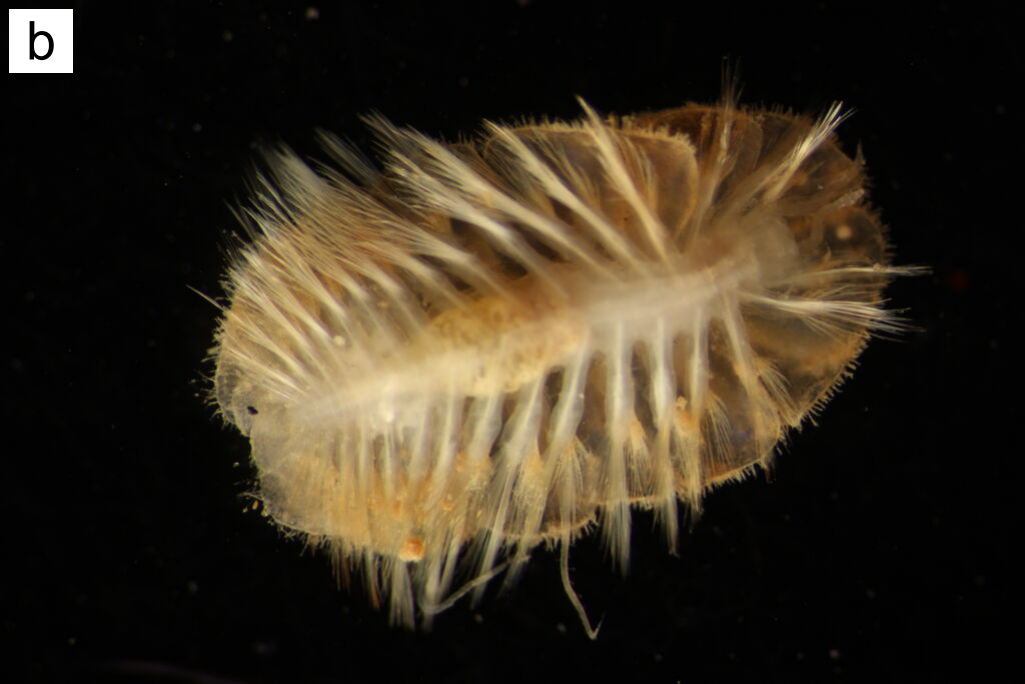
Complete live specimen NHM_235 in ventral view.

**Figure 115a.**
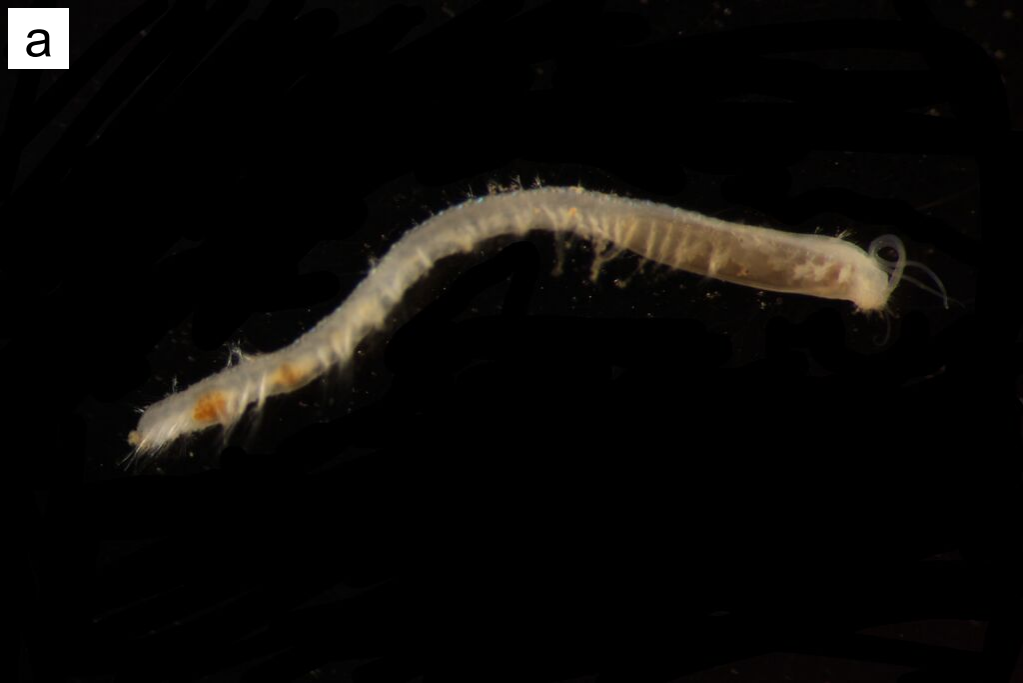
Live specimen NHM_881 in ventrolateral view;

**Figure 115b.**
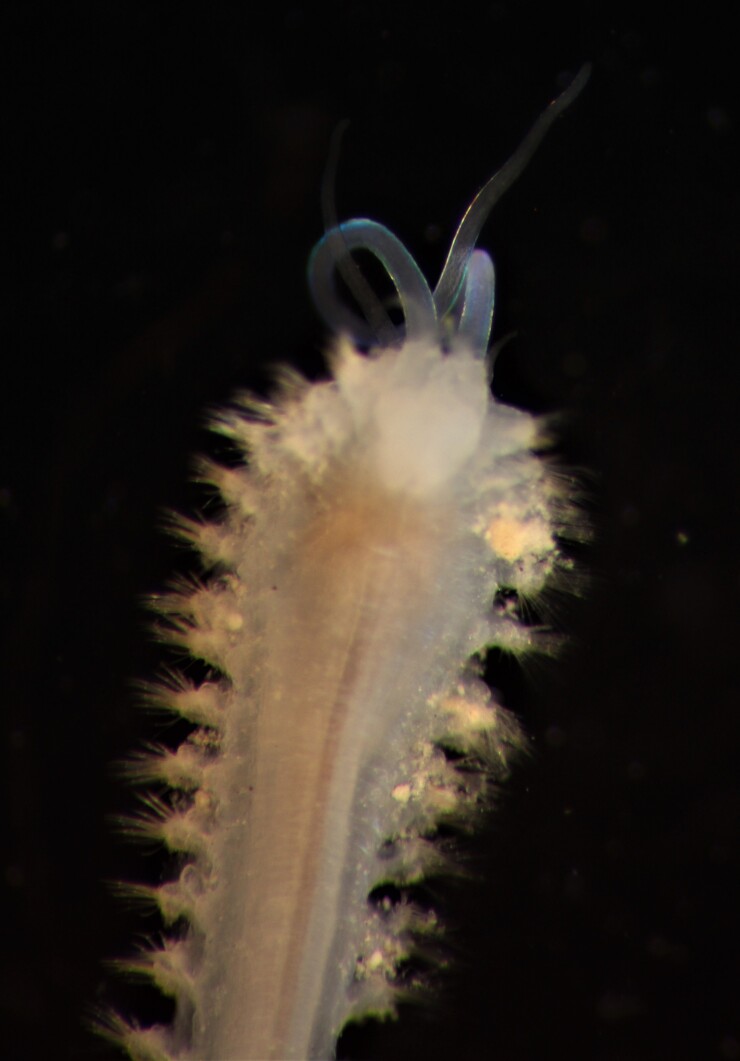
Anterior end of live specimen NHM_881 in dorsal view.

**Figure 117a.**
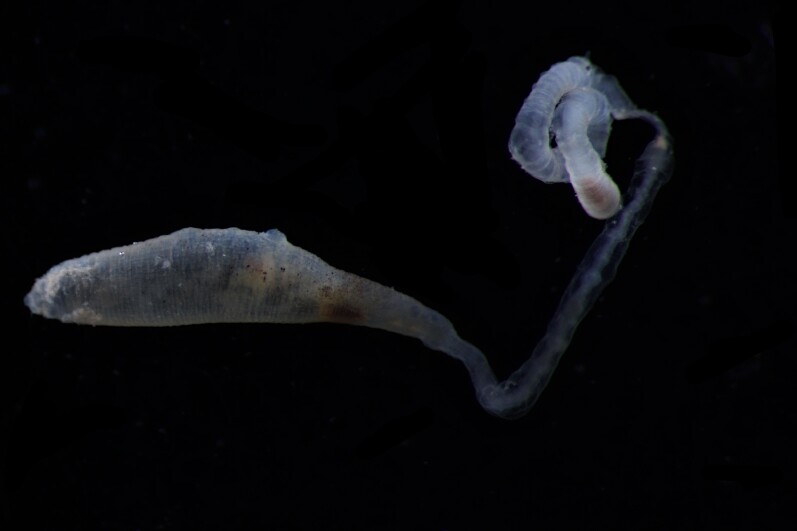
Live specimen NHM_126;

**Figure 117b.**
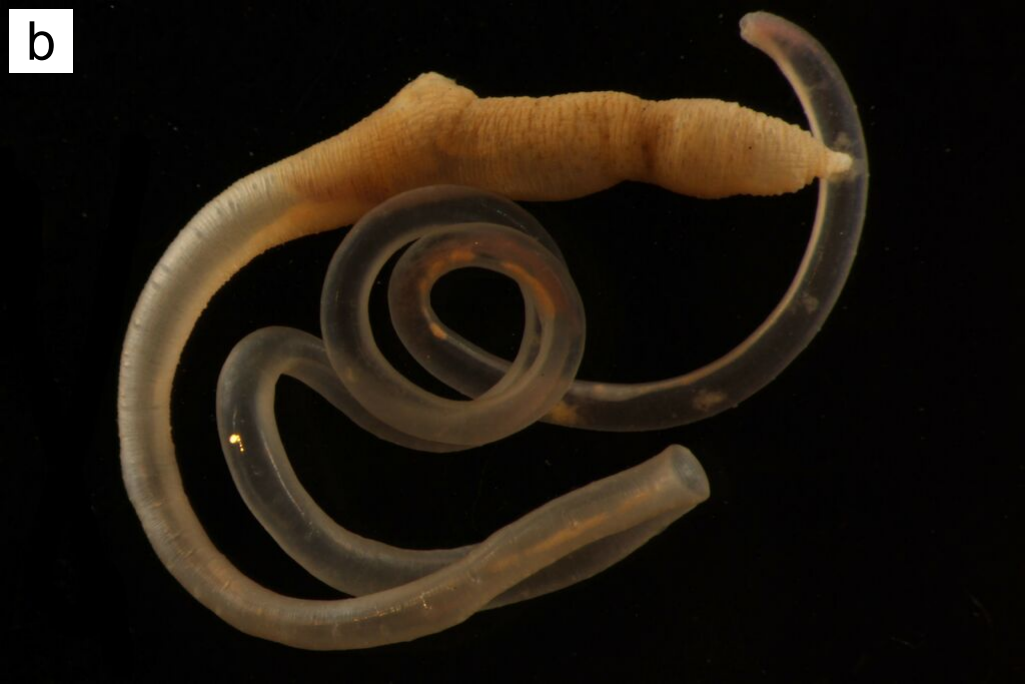
Live specimen NHM_1128.

**Figure 119a.**
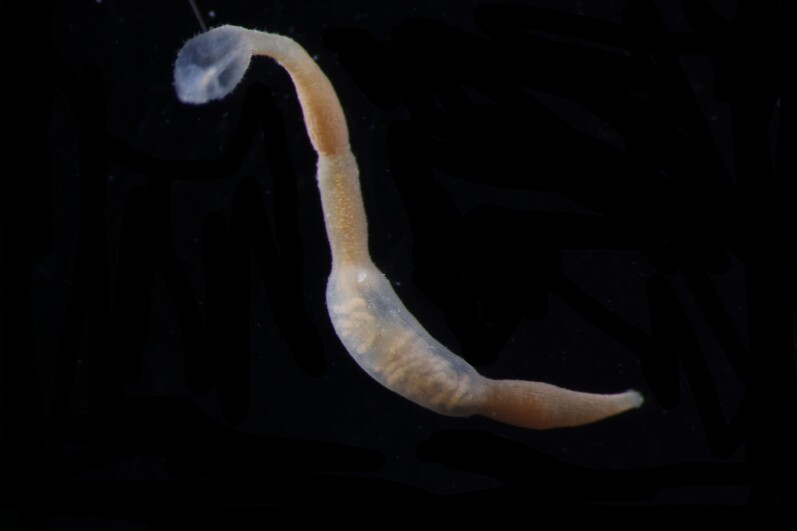
Live specimen NHM_715;

**Figure 119b.**
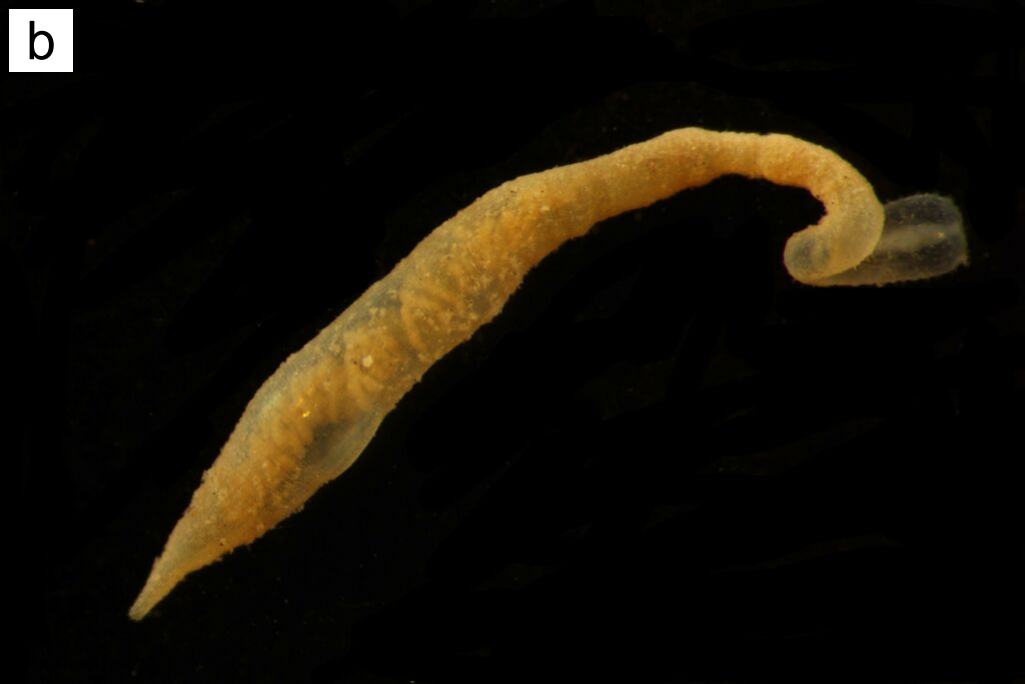
Live specimen NHM_603.

**Figure 125a.**
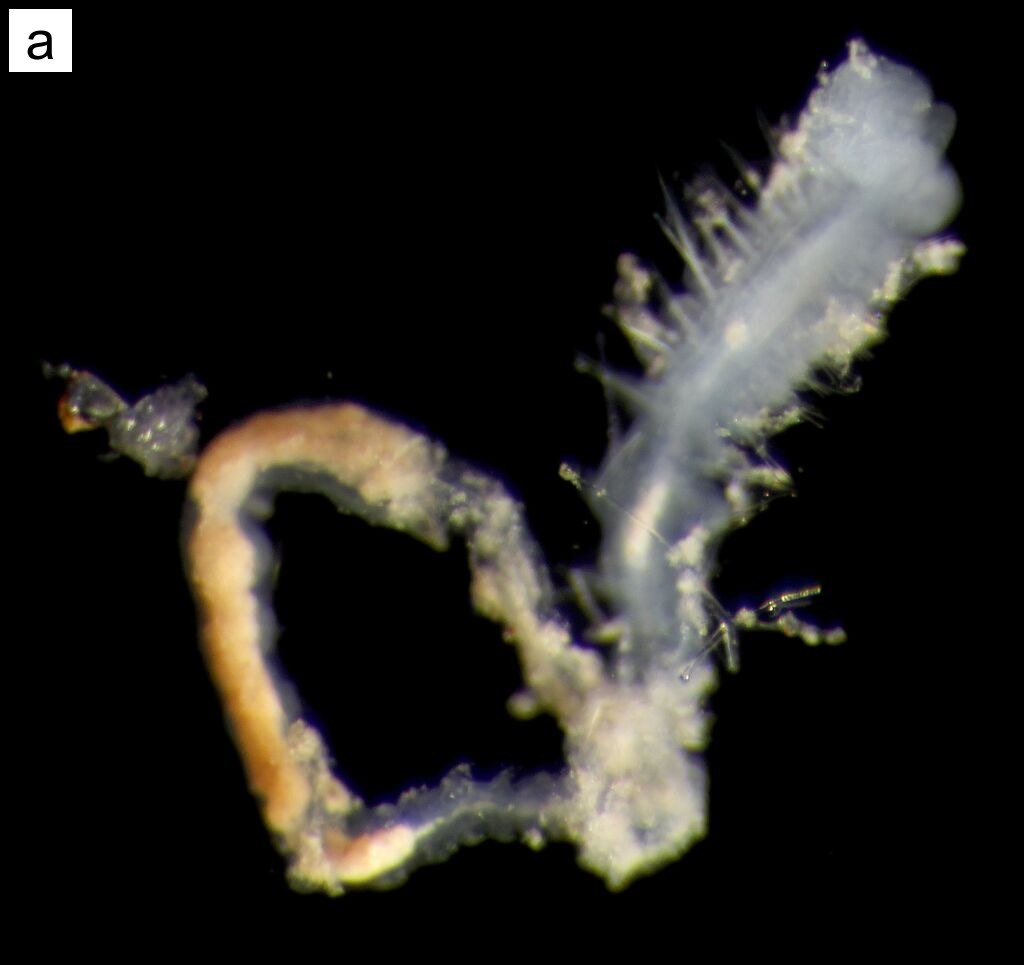
Live specimen NHM_167 in dorsal view

**Figure 125b.**
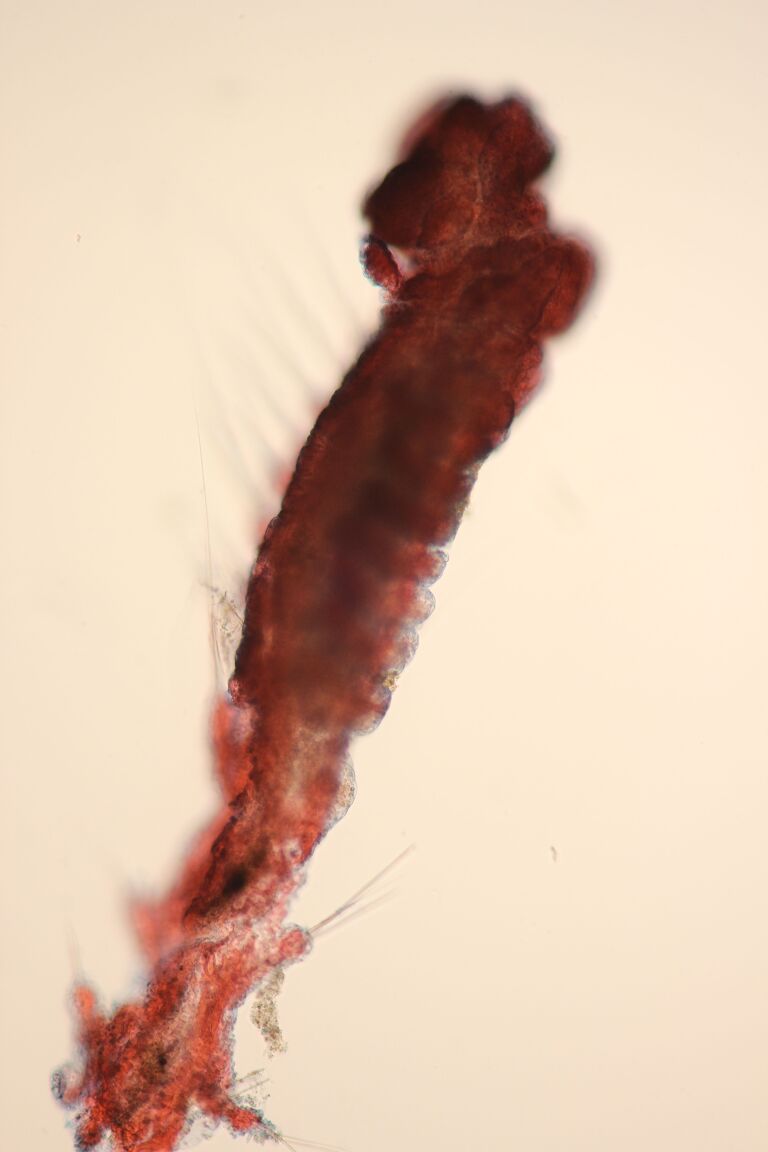
Preserved specimen NHM_167 in lateral view, branchiae visible.

**Table 1.** List of species included in this study, including the number of records per species.

**Family/Order**	**Species**	# **Records**
Ampharetidae	Ampharetidae sp. (NHM_015)	2
	Ampharetidae sp. (NHM_044)	3
	Ampharetidae sp. (NHM_062)	2
	Ampharetidae sp. (NHM_1082)	1
	Ampharetidae sp. (NHM_1161)	1
	Ampharetidae sp. (NHM_1338)	3
	Ampharetidae sp. (NHM_1578)	2
	Ampharetidae sp. (NHM_1599)	2
	Ampharetidae sp. (NHM_163)	3
	Ampharetidae sp. (NHM_265)	1
	Ampharetidae sp. (NHM_292)	4
	Ampharetidae sp. (NHM_774)	1
	Ampharetidae sp. (NHM_789)	4
Chaetopteridae	Chaetopteridae sp. (NHM_331)	3
Chrysopetalidae	Chrysopetalidae sp. NHM (1303)	2
	Chrysopetalidae sp. (NHM_1550)	1
	Chrysopetalidae sp. (NHM_2026)	1
	Chrysopetalidae sp. (NHM_410)	1
	Chrysopetalidae sp. (NHM_748A)	1
Cirratulidae	Cirratulidae sp. (NHM_172)	4
	Cirratulidae sp. (NHM_1001)	1
	Cirratulidae sp. (NHM_1235)	3
	Cirratulidae sp. (NHM_1429)	1
	Cirratulidae sp. (NHM_1518)	1
	Cirratulidae sp. (NHM_165)	1
	Cirratulidae sp. (NHM_2093)	1
	Cirratulidae sp. (NHM_2163)	4
	Cirratulidae sp. (NHM_269)	4
	Cirratulidae sp. (NHM_340)	5
	Cirratulidae sp. (NHM_530)	3
	Cirratulidae sp. (NHM_734)	1
	Cirratulidae sp. (NHM_904)	2
	Cirratulidae sp. (NHM_915G)	4
	Cirratulidae sp. (NHM_945C)	1
Flabelligeridae	Flabelligeridae sp. (NHM_045)	3
	Flabelligeridae sp. (NHM_1274)	2
	Flabelligeridae sp. (NHM_1313)	3
	Flabelligeridae sp. (NHM_1638)	2
	Flabelligeridae sp. (NHM_2124)	1
	Flabelligeridae sp. (NHM_555)	1
	Flabelligeridae sp. (NHM_630A)	1
	Flabelligeridae sp. (NHM_738)	3
	Flabelligeridae sp. (NHM_955)	7
Glyceridae	Glyceridae sp. (NHM_1242)	3
	Glyceridae sp. (NHM_207)	13
	Glyceridae sp. (NHM_2089)	2
Goniadidae	Goniadidae sp. (NHM_1512)	2
Lacydoniidae	Lacydoniidae sp. (NHM_1355C)	1
	Lacydoniidae sp. (NHM_1797D)	1
	Lacydoniidae sp. (NHM_898)	1
Magelonidae	Magelonidae sp. (NHM_1340)	2
Maldanidae	Maldanidae sp. (NHM_026)	5
	Maldanidae sp. (NHM_1170)	1
	Maldanidae sp. (NHM_1178)	2
	Maldanidae sp. (NHM_836)	5
	Maldanidae sp. (NHM_900)	1
Onuphidae	Onuphidae sp. (NHM_1010)	2
	Onuphidae sp. (NHM_2430)	1
Orbiniidae	Orbiniidae sp. (NHM_050)	4
	Orbiniidae sp. (NHM_102)	4
	Orbiniidae sp. (NHM_1947G)	1
	Orbiniidae sp. (NHM_264)	2
	Orbiniidae sp. (NHM_458)	2
	Orbiniidae sp. (NHM_754)	6
	Orbiniidae sp. (NHM_791)	2
	Orbiniidae sp. (NHM_824)	7
Paraonidae	Paraonidae sp. (NHM_059)	7
	Paraonidae sp. (NHM_1139)	1
	Paraonidae sp. (NHM_177)	2
	Paraonidae sp. (NHM_2118)	1
	Paraonidae sp. (NHM_332)	6
	Paraonidae sp. (NHM_363)	5
	Paraonidae sp. (NHM_412)	3
	Paraonidae sp. (NHM_434)	3
	Paraonidae sp. (NHM_584)	2
	Paraonidae sp. (NHM_902)	4
Pilargidae	*Ancistrosyllis* sp. (NHM_765)	9
Polynoidae	*Bathyeliasonamariaae*	2
	*Bathyfauveliaglacigena*	9
	*Bathyfauveliaignigena*	1
	*Polaruschakovlamellae*	1
	Polynoidae sp. (NGM_1074)	1
	Polynoidae sp. (NHM_034)	1
	Polynoidae sp. (NHM_1000)	1
	Polynoidae sp. (NHM_128)	8
	Polynoidae sp. (NHM_1655)	2
	Polynoidae sp. (NHM_1763)	1
	Polynoidae sp. (NHM_2097)	1
	Polynoidae sp. (NHM_2099)	1
	Polynoidae sp. (NHM_2101)	2
	Polynoidae sp. (NHM_2122)	1
	Polynoidae sp. (NHM_2195)	2
	Polynoidae sp. (NHM_583)	2
	Polynoidae sp. (NHM_588)	2
	Polynoidae sp. (NHM_589)	1
	Polynoidae sp. (NHM_595)	8
	Polynoidae sp. (NHM_679)	8
	Polynoidae sp. (NHM_690)	1
	Polynoidae sp. (NHM_747A)	1
	Polynoidae sp. (NHM_747D)	5
	Polynoidae sp. (NHM_756)	2
	Polynoidae sp. (NHM_773B)	7
	Polynoidae sp. (NHM_834)	1
Sabellariidae	Sabellariidae sp. (NHM_1167B)	4
Sabellidae	Sabellidae sp. (NHM_1613)	1
	Sabellidae sp. (NHM_1859A)	1
	Sabellidae sp. (NHM_1879)	1
	Sabellidae sp. (NHM_189)	1
	Sabellidae sp. (NHM_1991)	1
	Sabellidae sp. (NHM_351)	1
	Sabellidae sp. (NHM_370)	1
	Sabellidae sp. (NHM_472)	1
	Sabellidae sp. (NHM_535)	1
	Sabellidae sp. (NHM_647)	1
	Sabellidae sp. (NHM_915D)	2
Sigalionidae	Pholoinae sp. (NHM_366)	4
	Sigalionidae sp. (NHM_1093)	1
	Sigalionidae sp. (NHM_342)	9
	Sigalionidae sp. (NHM_881)	2
Sphaerodoridae	Sphaerodoridae sp. (NHM_1003)	1
	Sphaerodoridae sp. (NHM_2177)	1
	Sphaerodoridae sp. (NHM_2532)	1
Terebellidae	Terebellidae sp. (NHM_018)	1
Trichobranchidae	Terebellides sp. (NHM_167)	1
	Terebellides sp. (NHM_406)	1
Sipuncula	Sipuncula sp. (NHM_126)	6
	Sipuncula sp. (NHM_1298)	2
	Sipuncula sp. (NHM_148)	1
	Sipuncula sp. (NHM_715)	10

**Table 2.** Primers used for PCR and sequencing of COI and 16S.

Primer	Sequence 5'-3'	Reference
COI		
LCO1490	GGTCAACAAATCATAAAGATATTGG	Folmer et al. (1994)
HCO2198	TAAACTTCAGGGTGACCAAAAAATCA	Folmer et al. (1994)
COI-E	TATACTTCTGGGTGTCCGAAGAATCA	Bely and Wray (2004)
polyLCO	GAYTATWTTCAACAAATCATAAAGATATTGG	Carr et al. (2011)
polyHCO	TAMACTTCWGGGTGACCAAARAATCA	Carr et al. 2011
16S		
ann16SF	GCGGTATCCTGACCGTRCWAAGGTA	Sjölin et al. (2005)
16SbrH	CCGGTCTGAACTCAGATCACGT	Palumbi (1996)
